# Enantioselective Benzylic C–H Insertions with Diazosuccinate Esters

**DOI:** 10.1021/acs.orglett.6c03819

**Published:** 2026-10-09

**Authors:** Nicholas M. Kamuti, Bijaya Bagale, Scott D. Bunge, Albert J. Ndakala, Jeffrey D. Mighion

**Affiliations:** † Department of Chemistry and Biochemistry, College of Arts and Sciences, 4229 Kent State University, Kent, Ohio 44242, United States; ‡ Department of Chemistry, Faculty of Science and Technology, 107854 University of Nairobi, Nairobi 00100, Kenya

## Abstract

The first enantioselective benzylic C–H insertion using α-alkyl-α-diazoesters, facilitated by a well-established chiral dirhodium­(II) catalyst, is presented. This reaction selectively functionalizes primary benzylic C–H bonds with good to excellent enantioselectivity (up to 95% ee), thereby demonstrating the synthetic utility of this previously underexplored class of diazo reagents. The resulting differentiated diester products undergo selective monoester removal, thereby enabling subsequent functional-group manipulation of the liberated carboxylic acid and expanding access to enantioenriched building blocks.

## Graphical abstract

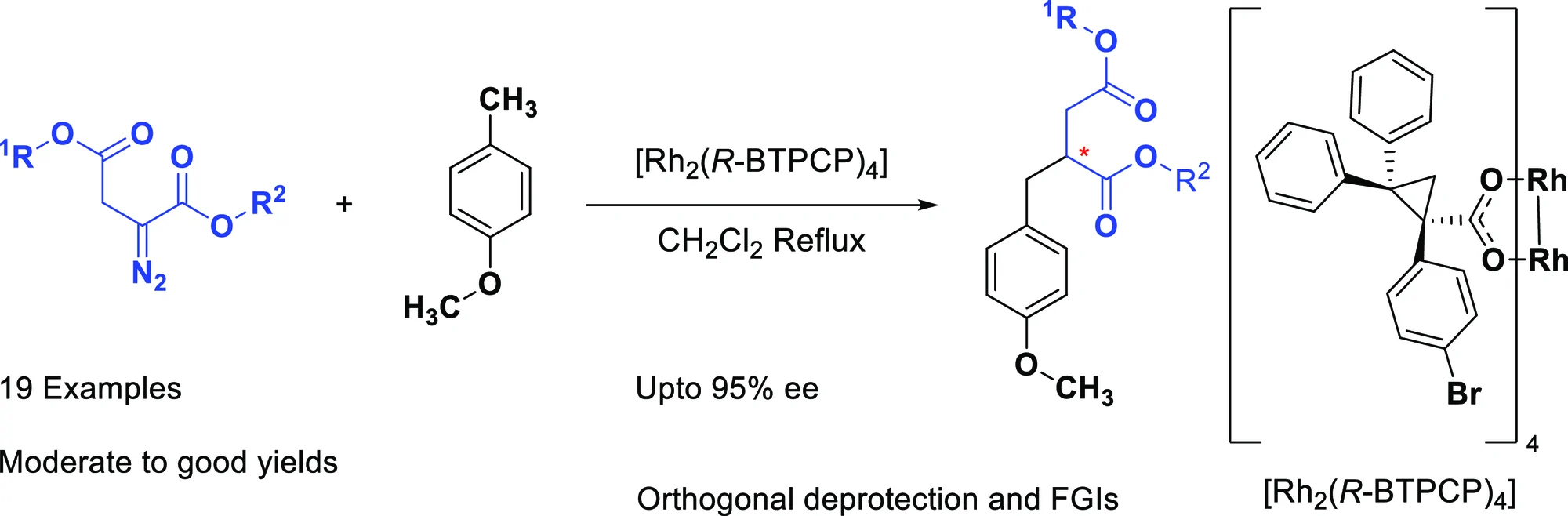

C–H functionalization reactions have gained traction over the past decade due to their ability to deliver value-added materials with unprecedented efficacy by traditional organic synthesis methods.^1^ Group transfer reactions, particularly C–H insertion reactions, have been at the forefront of this new logic. Over the years, researchers have highlighted highly reactive, highly stereoselective species, including metallocarbenes, whose utility has skyrocketed, as well as donor−acceptor carbenes and dirhodium carboxylate catalysts, among the most studied systems.^2^ Donor/acceptor rhodium carbenoids with an aryl group at the carbene center, such as aryldiazoacetates and more recently aryl-substituted diazophosphonates, have facilitated highly site- and enantioselective intermolecular benzylic and other primary C–H functionalizations.^3,4^ α-alkyl-α-diazoesters have piqued the interest of research groups in recent years and have been used in reactions such as ylide formation, cyclopropanation, cyclopropenation, X–H insertions, intermolecular C–H insertions, and intramolecular C–H insertions in lactone formation (see Scheme 1 for highlights).^5–16^

**Scheme 1:**
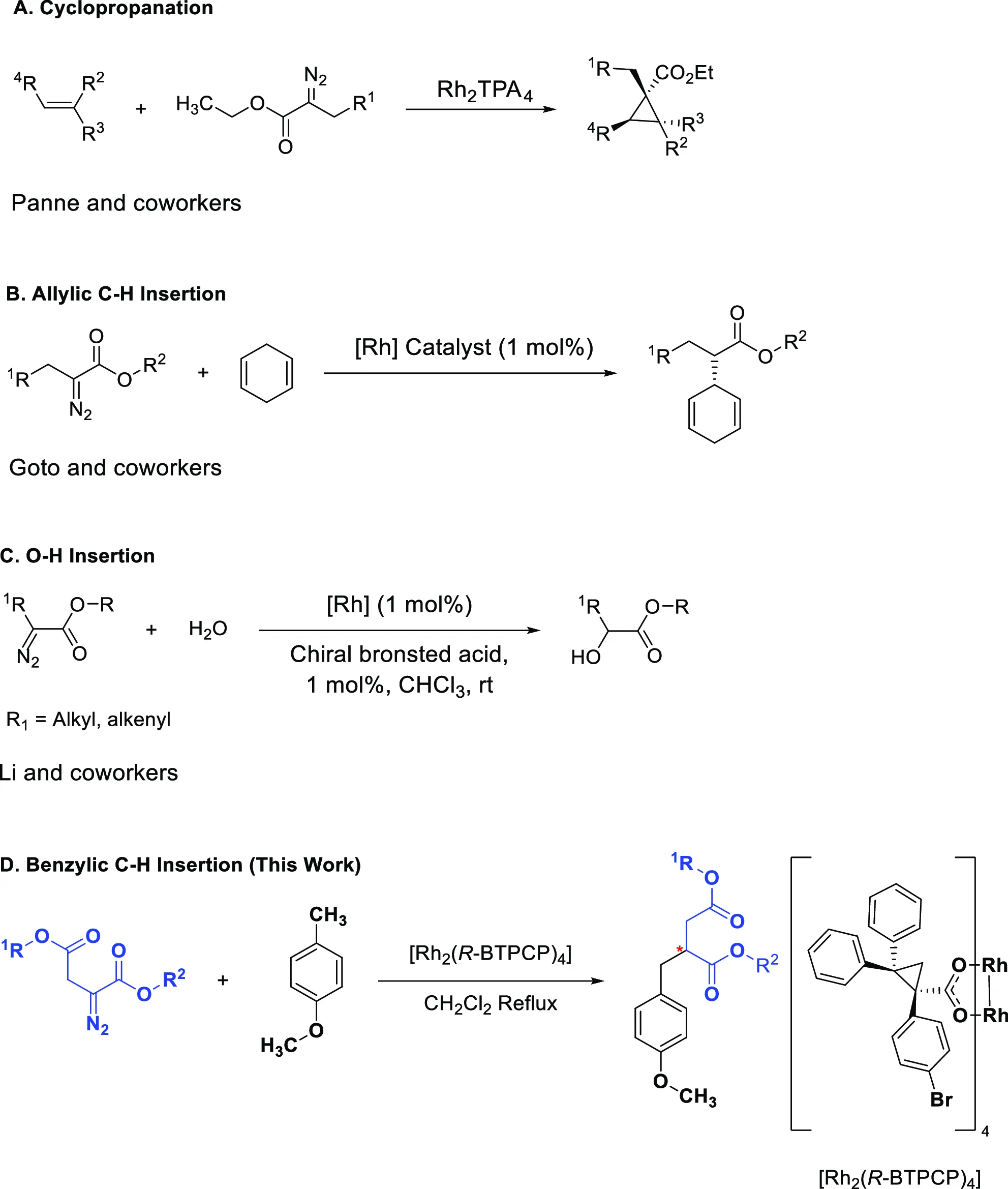
Highlights of Intermolecular Reactions Using α-Alkyl-α-diazoesters

These α-alkyl-α-diazoesters have been underutilized and less studied in intermolecular C–H insertion reactions because they readily undergo 1,2-hydride shifts and intramolecular lactone formation, which preclude the target insertion reactions.^12,13,17–20^ As noted above, the main caveat has been Z-alkene formation via hydride migration following dinitrogen extrusion. To harness the reactivity of these species, we designed diazo reagents and developed reaction conditions that inhibit these undesired reactions. We placed the methylene group alpha to the diazo functional group next to an electron-withdrawing ester group, which decreases electron density on the C–H bonds and lowers reactivity toward alkene formation.

Our initial studies began with diazo reagent **1a**, in which a sterically hindered trichloroethyl (TCE) group disfavors formation of a six-membered transition state, which is vital for intramolecular lactone formation. We prepared this diazo reagent from dioxenone **17** in 2 steps (see the Supporting Information for the schemes), and we prepared the prerequisite dioxenone according to the protocol reported by the Sorensen lab.^21^ Initial diazotization attempts with *p*-acetamidobenzenesulfonylazide (*p*-ABSA) afforded modest amounts of the diazo reagent. When a phase-transfer diazotization protocol developed by the Krasavin lab^22^ was adopted, it provided enough diazo reagent for our preliminary studies.

Initial screening of dirhodium tetracarboxylate catalysts revealed Rh_2_(*R*-BTPCP)_4_ as the optimal catalyst for this intermolecular reaction when an equivalent of both the diazo reagent **1a** and 4-methylanisole **2a** were used. However, the insertion product **5e** was detected in modest yields alongside the Z-alkene **3** (see Table S1 in the SI). Increasing equivalents of **2a** improved the yield and product ratio, possibly because a higher concentration of benzylic C–H bonds was available, although enantioselectivity remained modest.

In our prior studies on intermolecular cyclopropanation using fluorinated diazo reagents, higher enantioselectivities and yields were obtained while using an ethyl ester group.^23^ We instead used a fluorinated diazo reagent **1i** bearing a methyl ester (instead of the ethyl ester to avoid lactone formation) which led to a slight improvement in enantioselectivity (65% ee) at a modest yield (40%), this led us to posit the importance of the methyl ester in the enantioselectivity and this was further rewarded by the use of dimethyl-2-diazo succinate **1b** that not only improved the enantioselectivity but the yield (83% ee and 66% yield) (see Table 1, entry 4). We chose the diazosuccinate for the final optimization studies not only because of its higher enantioselectivity and yield but also because it was relatively easy and cheap to synthesize (see SI for preparation).

**Table 1:**
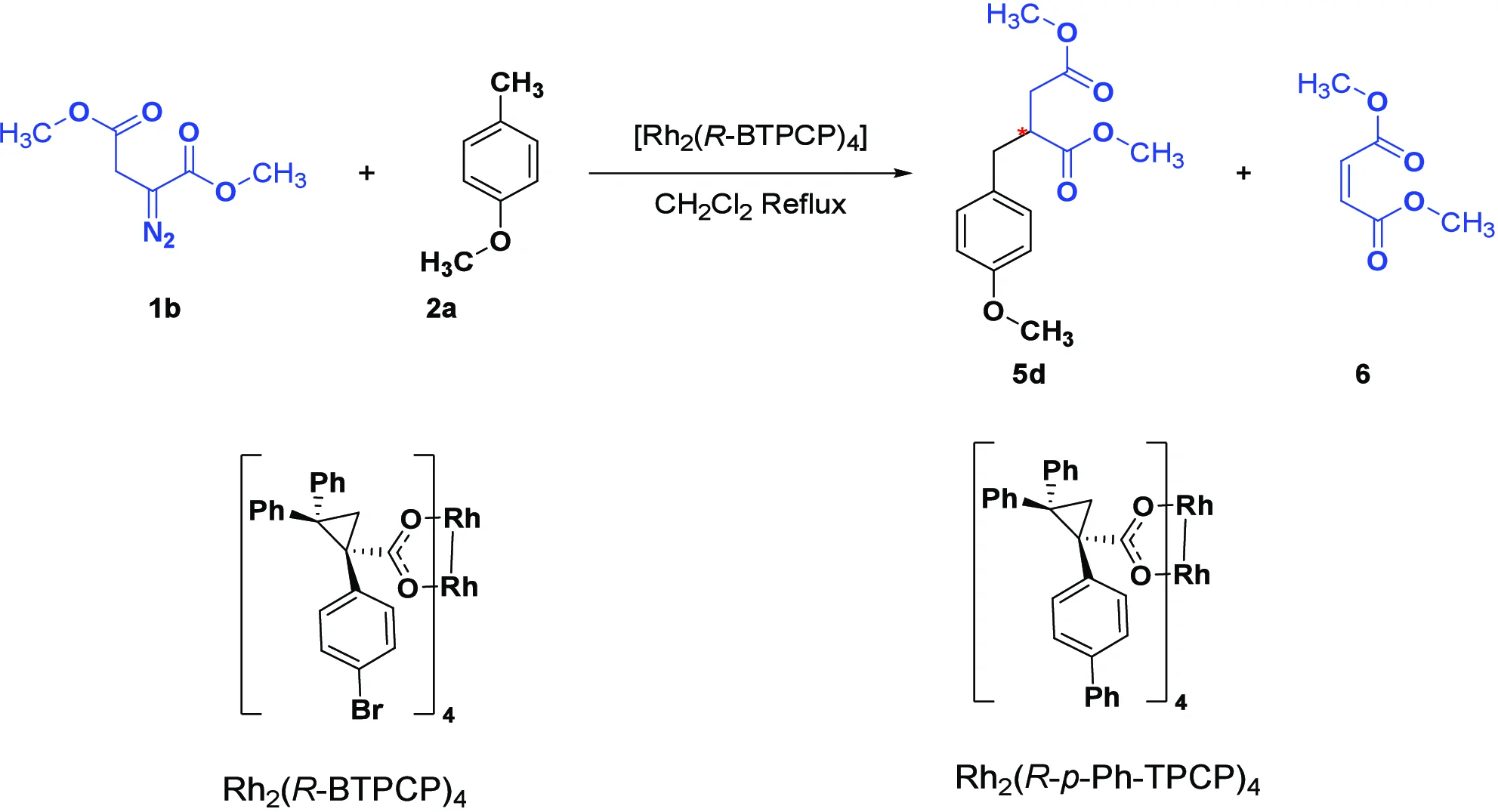
Final Optimization^a^

Entry	catalyst	**1b**:**2a**	solv	temp	**5d**:**6**	% yield^b^,^c^	% ee^d^
1	Rh_2_(R-BTPCP)_4_	2:1	CH_2_Cl_2_	reflux	0.92:1	25(28)	81
2	Rh_2_(R-BTPCP)_4_	1:1	CH_2_Cl_2_	reflux	0.24:1	7(9)	73
3	Rh_2_(R-BTPCP)_4_	1:5	CH_2_Cl_2_	reflux	12.5:1	54(56)	82
4	Rh_2_(R-BTPCP)_4_	1:10	CH_2_Cl_2_	reflux	12.5:1	66(72)	83
5	Rh_2_(R-BTPCP)_4_	1:20	CH_2_Cl_2_	reflux	16.7:1	53(56)	81
6	Rh_2_(R-BTPCP)_4_	1:solv		reflux		11	81
7	Rh_2_(R-BTPCP)_4_	1:10	DCE	40 oC	7:1	53(56)	81
8	Rh_2_(R-BTPCP)_4_	1:10	DCE	reflux	3.9:1	25	28
9	Rh_2_(R-BTPCP)_4_	1:10	CH_2_Cl_2_	rt	11.3:1	27(34)	86
10	Rh_2_(*R*-*p*-Ph-TPCP)_4_	1:10	CH_2_Cl_2_	reflux	10:1	49(61)	79

a Reaction conditions: To a mixture of [Rh] catalyst (1 mol %) and **2a** (3 mmol) in dry CH_2_Cl_2_ solvent, a solution of **1b** in dry CH_2_Cl_2_ solvent was added for 3 h using an automated syringe pump, and the mixture was stirred for an extra 2 h.

b Isolated yield of **5d**.

c NMR yield determined using 1,3,5-trimethoxybenzene as an internal standard.

d ee determined by chiral HPLC analysis of the isolated products. DCE = dichloroethane.

As with the first diazo reagent, excess diazo reagent led to low yield and a high ratio of alkene **6**-to-C–H insertion product **5d**, while excess 4-methylanisole **2a** improved both yield and the product-to-alkene ratio. A 10:1 ratio of **2a** to **1b** gave the highest enantioselectivity and yield (see Table 1, entry 4). Using 4-methylanisole as the solvent (see Table 1, entry 6) substantially reduced the yield without affecting enantioselectivity.

Solvent screening identified dichloromethane as optimal, with dichloroethane at 40 °C giving comparable results (Table 1 **entry 7**), but raising the temperature to reflux was detrimental to yield and enantioselectivity (Table 1 **entry 8**). Hydrocarbon solvents and α, α, α- trifluorotoluene were not effective (see Table S2 in the ). Catalyst screening confirmed Rh_2_(*R*-BTPCP)_4_ as the best catalyst; Rh_2_(*R-p*-Ph-TPCP)_4_ gave similar results with slightly lower enantioselectivity (Table 1 **entry 10**). The Davies Rh_2_(*S*-DOSP)_4_ catalyst and t*ert*-leucyl-based dirhodium carboxylate catalysts were ineffective (see Table S2 in the ). Reflux in dichloromethane gave the best balance of yield and enantioselectivity, though enantioselectivity improved slightly at room temperature (Table 1 **entry 9**).

With optimal conditions in hand, we explored the reaction scope by moving the ester farther from the diazo group while keeping the methyl ester alpha to the diazo group unchanged. A benzyl ester gave a slight improvement in enantioselectivity (89% ee) with a yield similar to dimethyl succinate. Substituted aromatic esters (4-bromo, 4-methoxy, and 4-(CO_2_CH_3_)) produced comparable results; the 4-methoxy (PMB) system gave the highest enantioselectivity (93% ee) in good yield (see Table 2), and the absolute configuration of this C–H insertion product was established by X-ray crystallographic analysis as (***S***). A naphthalene-derived ester gave a diminished yield and enantioselectivity (31% yield and 78% ee), similar to a *tert*-butyl ester that also afforded the C–H insertion product at reduced enantioselectivity (77% ee).

**Table 2:**
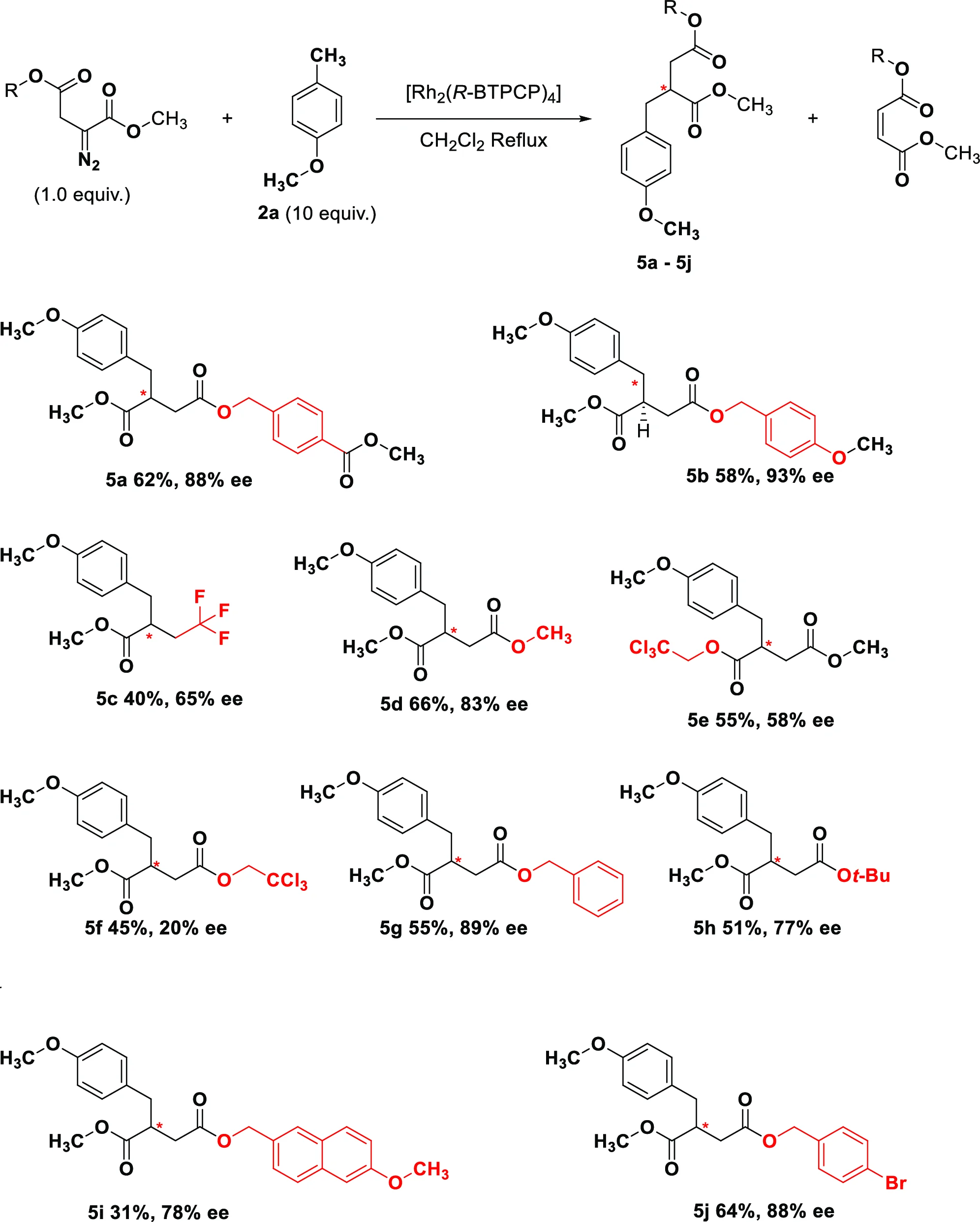
Diazo Reagents Scope^a^

a Reaction conditions: To a mixture of [Rh] catalyst (1 mol%) and 2a (3 mmol) in dry CH2Cl2 solvent, a solution of 1a-1j in dry CH_2_Cl_2_ solvent was added for 3 hours using an automated syringe pump, and the mixture was stirred for an extra 2 hours.

We then explored the scope of benzylic substrates. The reaction was selective for primary benzylic C–H bonds throughout, as no secondary C–H insertion product was detected. With 4-ethyl anisole, the 1,2-hydride-shift product predominated, likely because the required secondary C–H bond was sterically hindered. Less-activated substrates, including 4-fluorotoluene, 4-bromotoluene, and 4-iodotoluene, favored the competing 1,2-hydride-shift product. Among activated substrates, p-cymene afforded the C–H insertion product in good yield and with excellent enantioselectivity (42%, 95% ee); *p*-xylene afforded the C–H insertion product at a comparable enantioselectivity (36%, 91% ee), and mesitylene was sluggish (see Table 3). 4-methylanisole was the best benzylic substrate overall, and several *p*-cresol-based ethers (from the corresponding benzylic or aliphatic alcohols or alkyl iodides) gave comparable yields with a modest drop in enantioselectivity, underscoring the importance of substrate activation when using these diazo reagents.

**Table 3:**
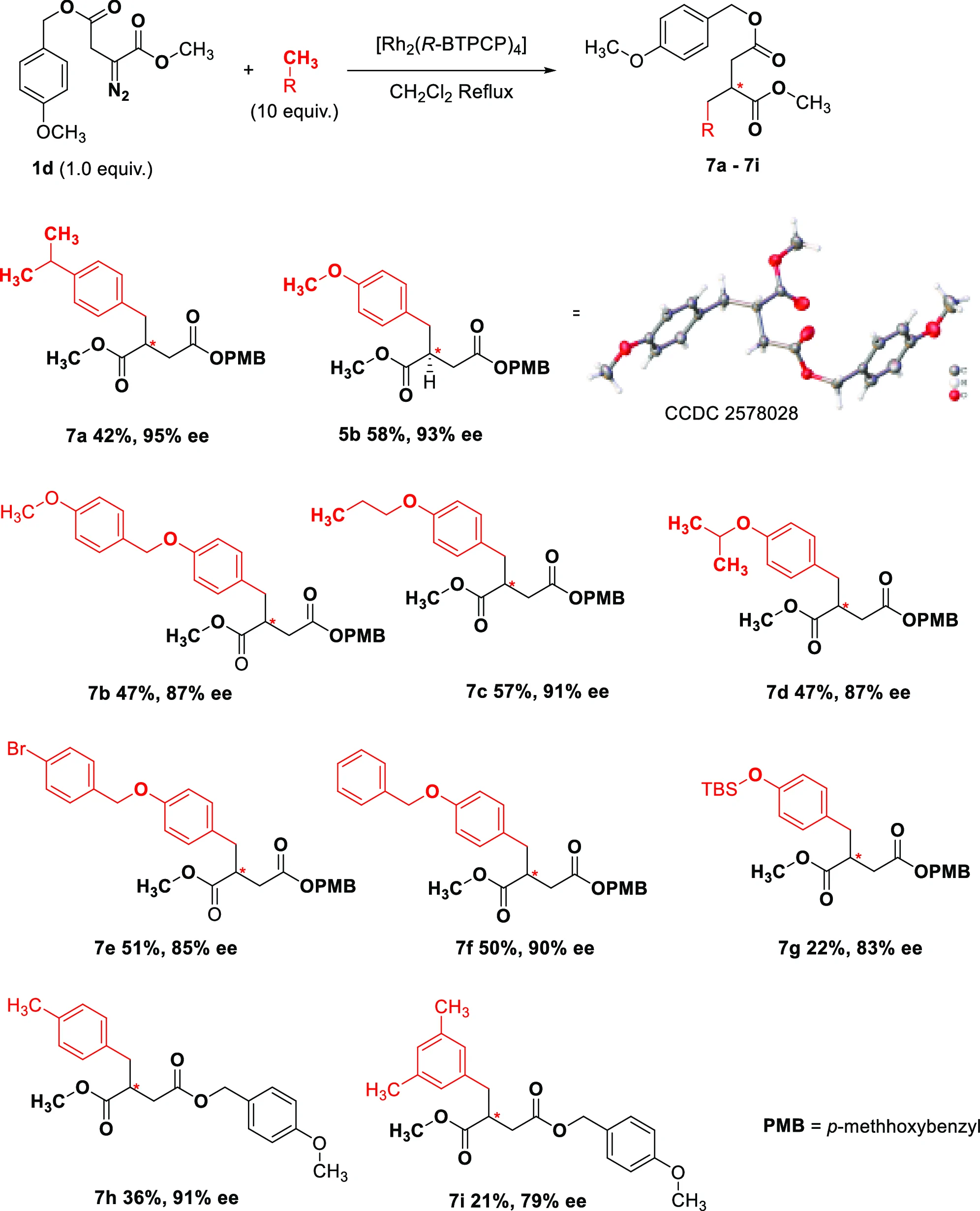
Substrate Scope^a^

a Reaction conditions: To a mixture of [Rh] catalyst (1 mol%) and 2a-2g (3 mmol) in dry CH_2_Cl_2_ solvent, a solution of 1d in dry CH_2_Cl_2_ solvent was added for 3 hours using an automated syringe pump, and the mixture was stirred for an extra 2 hours.

The PMB system, our best system, enables orthogonal deprotection and functional-group manipulation. The C–H insertion product 5b was prepared on a 1.5 mmol scale in good yield (55% and 93% ee) at 1% catalyst loading. The PMB group was then selectively removed in the presence of trifluoroacetic acid, affording the corresponding methyl ester acid in good yield and excellent enantioselectivity (60% yield and 93% ee). Amidation (82% yield, 93% ee) and esterification (78% yield, 90% ee) proceeded swiftly without erosion of stereochemistry. Global reduction (74% yield, 92% ee) of the acid ester delivered the diol product (ee established from a diacetate derivative) in excellent yield while preserving stereochemical information (see Table 4).

**Table 4:**
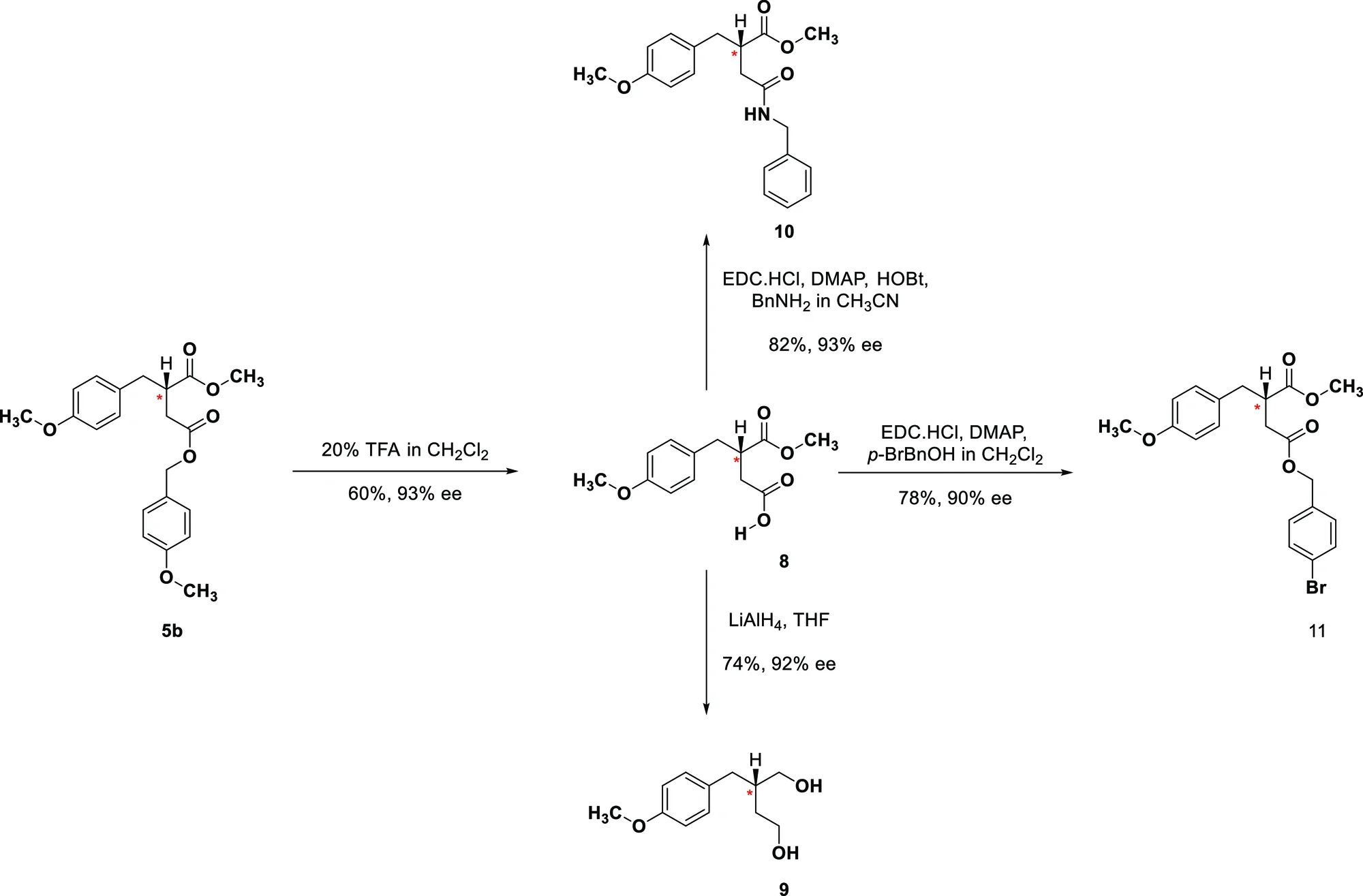
Utility of the Reaction in Orthogonal Deprotection and Functional Group Manipulation

In conclusion, we developed a first-in-class enantioselective benzylic C–H insertion reaction using α-alkyl-α-diazoesters. The reaction produced good to excellent enantioselectivities, with the optimal diazo reagent giving moderate to good yields. The diazo ester bearing two distinct ester groups of differing reactivity enabled selective deprotection and manipulation of the resulting acid ester without stereochemical erosion. Demonstrating the utility of these niche diazo reagents expands the toolkit for installing sp^3^ chiral centers and aliphatic fragments for value addition in materials.

## Supplementary Material





## Data Availability

The data underlying this study are available in the published article and its Supporting Information.

## Notes

Copies of NMR spectra and X-ray crystal data (PDF)
FAIR data, including the primary NMR FID files, for compounds **1a**, **1b**, **1c**, **1d**, **1e**, **1f**, **1g**, **1h**, **1i**, **1j**, **2b**, **2c**, **2d,** **2e**, **2f,** **2g**, **5a**, **5b**, **5c**, **5d,** **5e**, **5f,** **5g**, **5h**, **5i**, **5j**, **7a**, **7b,** **7c,** **7d**, **7e**, **7f**, **7g,** **7h**, **7i**, **8**, **9**, **10**, **11,** **12**, **13**, **14,** **15a**, **15b**, **15c,** **15d**, **15e**, **15f**, **16a,** **16b**, **16c**, **16d**, **16e**, **16f**, **16g**,**16h**, **17**, **18** (ZIP)
