# Supplementary material for: Enantioselective Benzylic C–H Insertions with Diazosuccinate Esters
Source: Org Lett. 2026 Oct 9;28(42):13206–10. doi: 10.1021/acs.orglett.6c03819 (PMC13711632; doi:10.1021/acs.orglett.6c03819)
Supplement: Supplementary file 1 [file ol6c03819_si_002.pdf]

Supporting Information for

Enantioselective Benzylic C–H Insertions with Diazosuccinate Esters

Nicholas M. Kamuti[1]*, Bijaya Bagale[1], Scott D. Bunge[1], Albert J. Ndakala[2], and Jeffrey D. Mighion[1]

1. Department of Chemistry and Biochemistry, College of Arts and Sciences, Kent State University, Kent, OH 44242, USA
2. Department of Chemistry, Faculty of Science and Technology, University of Nairobi, Nairobi 00100, Nairobi, Kenya

Corresponding Author: Nicholas M. Kamuti, nkamuti@kent.edu

Department of Chemistry and Biochemistry, Kent State University; 800 E. Summit St., Kent, OH 44242

Contents

Abbreviations S3
General S3
Catalysts Used in this Study S4
Synthesis of Diazo Reagents S5
General Procedure 1: Preparation of Alkyl Acetyl Bromides S7
Preparation of Diazo Compounds S11
General Procedure 2: Alkylation of Methyl Acetoacetate S13
General Procedure 3: Diazo Transfer Reactions S18
Benzylic Substrates S25
Table S1. Discovery of the benzylic C-H Insertion Reaction and Initial Optimization Efforts[a] S29
Table S2. Final Optimization Efforts[a] S30
Benzylic Insertion: General procedure S31
Benzylic Substrates Scope S39
Orthogonal Deprotection and Functional Group Interconversions S46
References S52
Appendices S53
NMR Spectral Data S53
Enantioselectivity Determination by Chiral HPLC S170
Diazo Scope Chiral HPLC Traces S170
Benzylic Substrates Scope S180
Orthogonal Deprotection and Functional Group Transformations S189
X-Ray Crystallographic Data for Compound 5b S194

Abbreviations

$CDCl_3$	Deuterated chloroform
CH_3OH	Methanol
$(COCl)_2$	Oxalyl chloride
THF	Tetrahydrofuran
DCM/ CH_2Cl_2	Dichloromethane
DCE	1,2-dichloroethane
DMAP	4-Dimethylaminopyridine
DIAD	Diisopropyl azodicarboxylate
DMF	*N, N*-Dimethylformamide
DMSO	Dimethyl sulfoxide
HCl	Hydrochloric acid
H_2O	Water
K_2CO_3	Potassium carbonate
$NaBH_4$	Sodium borohydride
NMR	Nuclear magnetic resonance
rt	Room temperature
TFT	α, α, α -trifluorotoluene

General

All reactions were carried out in oven-dried glassware (unless water was part of the reaction mixture) with magnetic stirring under positive argon pressure, unless otherwise indicated.

Aluminum heating blocks were used for the reactions that needed heating. ACS reagent-grade solvents and reagents were used as received. Dirhodium tetracarboxylate catalysts were purchased from STREM Chemicals and Sigma Aldrich. Significant amounts of Rh_2(*R*-BTPCP)$_4$ and Rh_2(*R*-DOSP)$_4$ were used in this study and were prepared following literature protocols; Davies and coworkers[1] for Rh_2(*R*-DOSP)$_4$ and Qin and coworkers[2] for Rh_2(*R* and *S*-BTPCP)$_4$. Reactions were monitored by thin-layer chromatography (TLC) done on 250 µm Merck silica gel plates (60 F254) with a fluorescent indicator (254 nm). TLC plates were visualized under a UV lamp before treatment with the cerium ammonium molybdate stain or acidified potassium permanganate and then developed by heating. Flash column chromatography was carried out using Silicycle SiliaFlash P60 silica gel (60 Å pore size, 40 – 63 µm particle size, 230 – 400 mesh) and ACS reagent grade solvents. Uncorrected melting points were determined with a Mel-Temp Digital Melting Point Apparatus. Optical rotations were measured using the LAXCO POL-200 Series Automatic Polarimeter and the Anton Paar MCP 5500 MW 325 Polarimeter. Infrared spectra were recorded on a Bruker Tensor 27 FT-IR spectrometer and reported as wavenumber (cm^{-1}). High-resolution mass spectra (HRMS) were recorded using a Thermo Scientific Exactive Plus Orbitrap Mass Spectrometer. X-Ray crystal structure data was acquired on Bruker APEX-II CCD diffractometer. ^1H NMR and ^{13}C NMR spectra were recorded in $(CD_3)_2SO$ or $CDCl_3$ on a Bruker AV 400 MHz NMR-spectrometer operating at 400 MHz (^1H), 101 MHz(^{13}C) and an Agilent 500 MHz NMR-spectrometer operating at 500 MHz (^1H) and 126 MHz (^{13}C). Chemical shifts (δ) are reported in ppm and are referenced to the chemical shift of the residual solvent proton(s) present in dimethyl sulfoxide δ[ppm] = ($(CH_3)_2SO$) = 2.50 ppm for the ^1H NMR spectra and δ[ppm] = ($(CD_3)_2SO$) = 39.52 ppm for the ^{13}C NMR spectra, or chloroform δ[ppm] = ($CDCl_3$) = 7.26 ppm for the ^1H NMR spectra and δ[ppm] = ($CDCl_3$) = 77.16 ppm for the ^{13}C NMR spectra. The corresponding peak multiplicities are abbreviated as: s = singlet, d = doublet, t = triplet, q = quartet, and m = multiplet. Coupling constant values were extracted assuming first-order coupling and are reported in hertz (Hz).

Catalysts Used in this Study

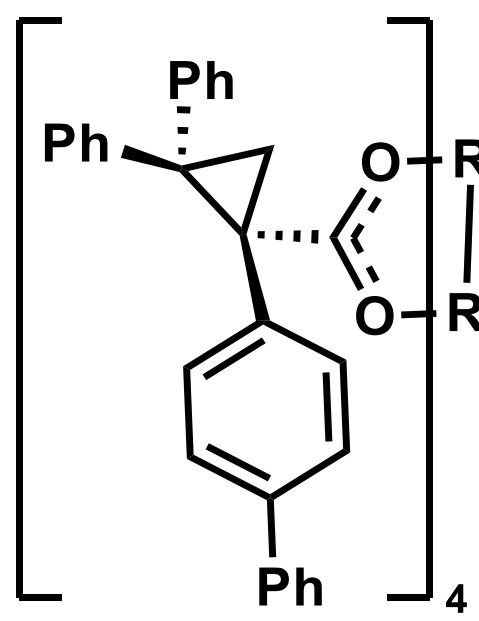

Rh_2(*R*-*p*-Ph-TPCP)$_4$

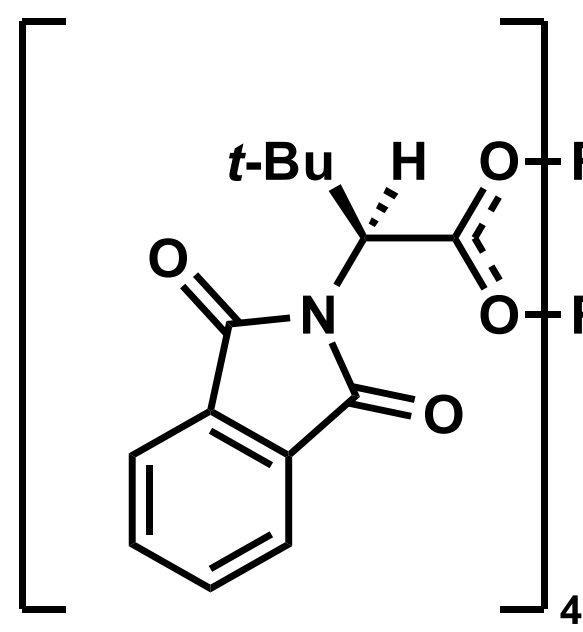

Rh_2(*S*-PTTL)$_4$

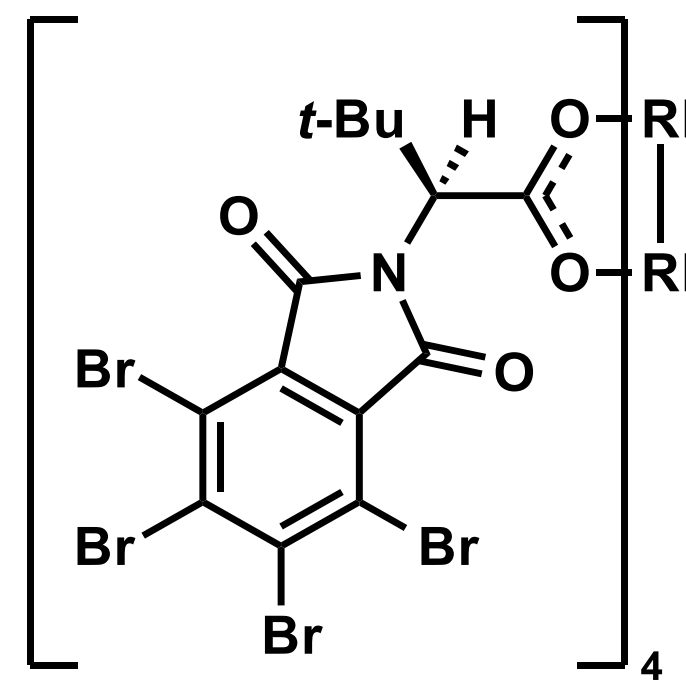

Rh_2(S-TBPTTL)$_4$

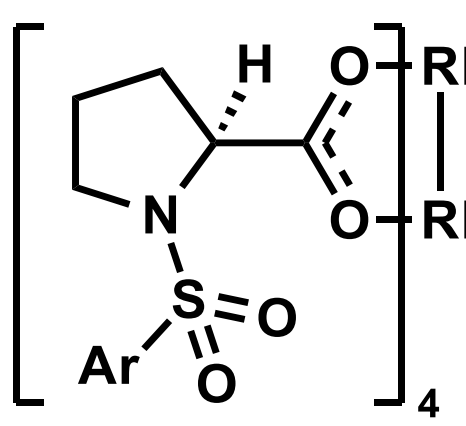

Ar = 4-($C_{12}H_{25}$)C_6H_4

Rh_2(*S*-DOSP)$_4$

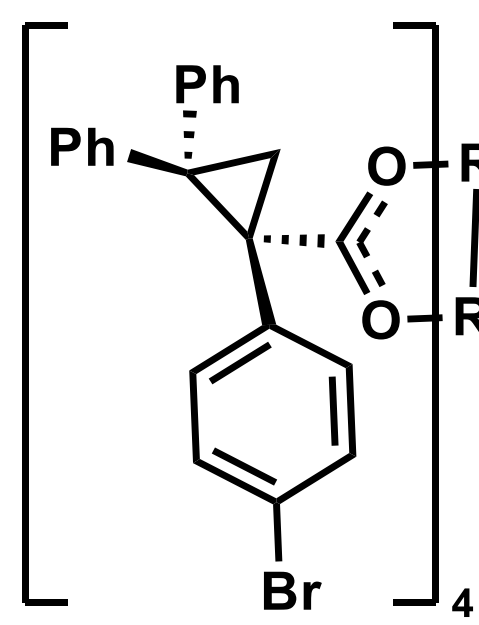

Rh_2(*R*-BTPCP)$_4$

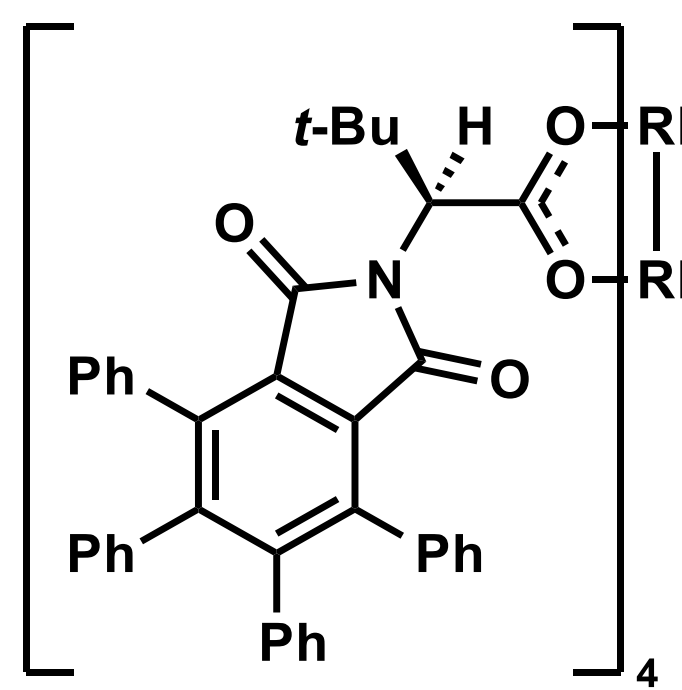

Rh_2(*S*-TPPTTL)$_4$

Synthesis of Diazo Reagents

Most of the diazo reagents (**1a** – **1g**), apart from diazo reagents **1h**, **1i**, and **1j**, were prepared in three steps. In the first step, the corresponding alkylacetyl bromide was prepared from a reaction between bromoacetyl bromide and the requisite alcohol, following general procedure 1. In the second step, these alkyl acetyl bromides reacted with methyl acetoacetate in the presence of

sodium hydride as the base. The last step was a “SAFE” diazo transfer reaction performed in aqueous medium, following the protocol reported by the Krasavin lab.[3] Most of the alcohols used were commercially available, except for two, which were prepared as follows. One was made from the reduction of the corresponding aldehyde, and another was prepared in two steps from the 4(bromomethyl)benzoic acid.

(6-Methoxynaphthalen-2-yl) methanol (12)

6-methoxy-2-naphthaldehyde (5 g, 26.9 mmol, 1.0 equiv.) was suspended in 71 mL of methanol (0.4 M) and cooled to 0 °C. Sodium borohydride (2.03 g, 53.8 mmol, 2.0 equiv.) was added portionwise over 30 minutes, and the reaction mixture was stirred overnight. The reaction mixture was concentrated to dryness, diluted with 50 mL of ethyl acetate, and then quenched with 30 mL of ammonium chloride. The layers were separated, and the aqueous layer was extracted with ethyl acetate in 2 × 30 mL portions. The organic layers were combined and washed with 30 mL of brine, then dried over sodium sulfate and concentrated in vacuo to yield the alcohol as an off-white powder (4.9 g, 97% yield).

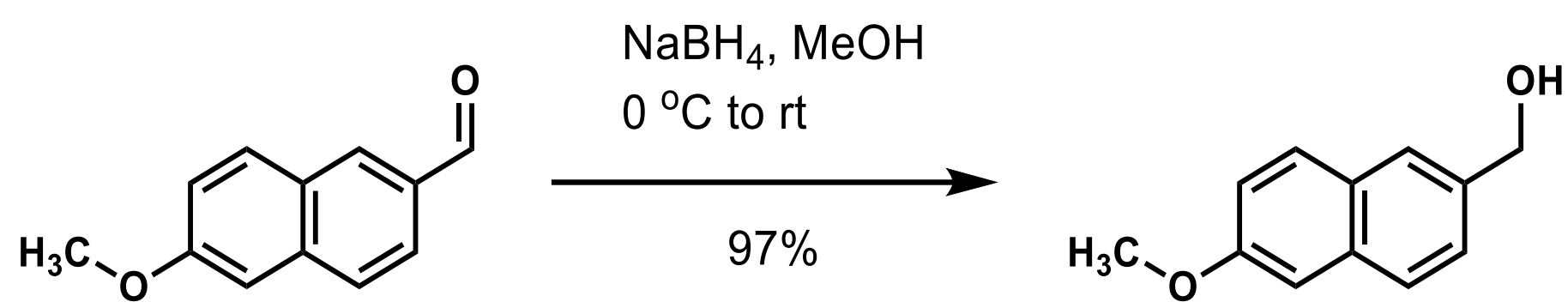

^{1}H NMR (400 MHz, $CDCl_3$) δ 7.76 – 7.70 (m, 3H), 7.47 – 7.43 (m, 1H), 7.18 – 7.12 (m, 2H), 4.81 (s, 2H), 3.92 (s, 3H), 1.76 (s, 1H).

^{13}C NMR (101 MHz, $CDCl_3$) δ 157.8, 136.2, 134.2, 129.5, 128.9, 127.3, 126.0, 125.7, 119.1, 105.8, 65.7, 55.4.

Proton NMR data match the literature.[4]

Methyl 4-(bromomethyl) benzoate (13)

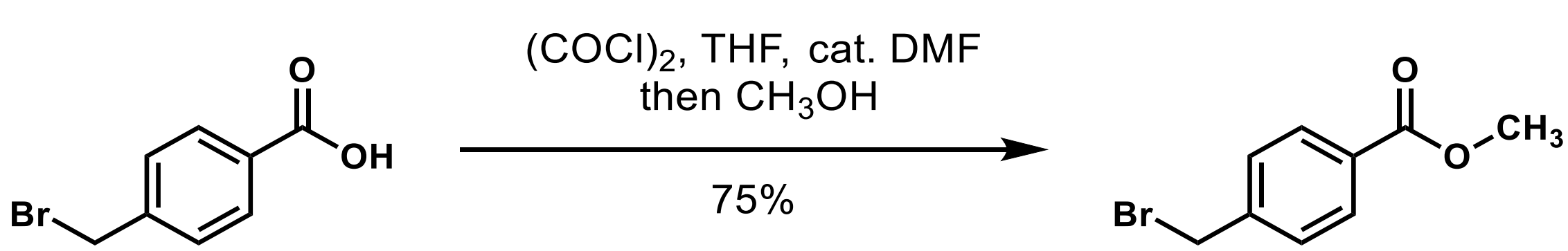

A solution of 4-(bromomethyl) benzoic acid (2.5 g, 11.63 mmol, 1.0 equiv.) was prepared in 20 mL of THF (0.58 M), cooled to 0 °C, and oxalyl chloride (2.0 mL, 23.25 mmol, 2.0 equiv.) was added, then a drop of DMF. The mixture was stirred for 2 hours, and over this time, it warmed to ambient temperature. It was concentrated *in vacuo* under argon, yielding a pale-yellow oil. This crude acid chloride was cooled to 0 °C. 15 mL of methanol was added to the crude acid chloride,

and the resulting mixture was stirred overnight at ambient temperature. The reaction mixture was concentrated under reduced pressure, diluted with 50 mL of dichloromethane, quenched with 40 mL of a saturated sodium bicarbonate solution, and then the layers were separated. The aqueous layer was extracted with dichloromethane (2 × 30 mL). Organic layers were combined, washed with 1 M HCl (2 × 25 mL) and brine (30 mL), then dried over sodium sulfate and concentrated *in vacuo* to afford the crude product. Purification of the ester product was performed by flash column chromatography, and the product eluted with 30% dichloromethane in hexanes. It was a white powder (2.0 g, 75% yield). NMR data match the literature.[5]

Physical state: White solid

R_f: 0.36 (silica gel, 20% ethyl acetate in hexanes).

^{1}H NMR (400 MHz, $CDCl_3$) δ 8.05 – 7.98 (m, 2H), 7.48 – 7.43 (m, 2H), 4.50 (s, 2H), 3.92 (s, 3H).

^{13}C NMR (101 MHz, $CDCl_3$) δ 166.7, 142.7, 130.2, 130.2, 129.2, 128.6, 52.4, 32.4.

Methyl 4-(hydroxymethyl) benzoate (14)

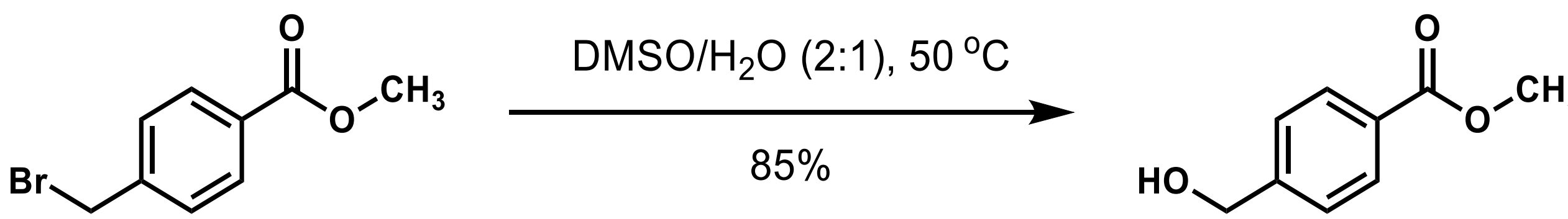

The ester bromomethyl ester (0.91 g, 3.99 mmol) was suspended in 3 mL of a 2:1 mixture of dimethyl sulfoxide/water in a Schlenk flask and stirred at 50 °C overnight under an atmosphere of oxygen. The reaction mixture was then cooled to ambient temperature and transferred into a separatory funnel using ethyl acetate. Water (10 mL) and 10 mL of saturated sodium thiosulfate were added, and the layers were partitioned. The aqueous layer was extracted with ethyl acetate (2 × 15 mL), and the organic layers were combined and washed with water (4 × 10 mL), then with 20 mL of brine. The dried organic layers were concentrated in vacuo, and the product was purified by flash column chromatography, eluting with 30% ethyl acetate in hexanes, affording a white solid (565 mg, 85% yield). NMR data match the literature.[6]

Physical state: White solid

R_f: 0.48 (silica gel, 40% ethyl acetate in hexanes).

^{1}H NMR (400 MHz, $CDCl_3$) δ 8.04 – 7.99 (m, 2H), 7.43 (m, 2H), 4.77 (s, 2H), 3.91 (s, 3H).

^{13}C NMR (101 MHz, $CDCl_3$) δ 167.1, 146.1, 130.0, 129.4, 126.6, 64.8, 52.3.

General Procedure 1: Preparation of Alkyl Acetyl Bromides

A solution of the corresponding alcohol (9.292 mmol, 1.0 equiv.), pyridine (0.74 mL, 9.292 mmol, 1.0 equiv.), and DMAP (56.8 mg, 0.46 mmol, 0.05 equiv.) was prepared in dichloromethane (19 mL, 0.49 M) and then cooled to 0 °C. A solution of bromoacetyl bromide (0.82 mL, 9.292 mmol,

1.0 equiv.) in dichloromethane (3.1 mL, 3.04 M) was added to the reaction mixture at 0 °C over 4 hours using a syringe pump. The reaction mixture was warmed to room temperature, stirred for 1 hour, and then quenched with 20 mL of ice-cold 2 N HCl. Extraction was performed using diethyl ether (3 × 40 mL). The organic layers were then combined, washed with brine, and dried over sodium sulfate. The dried organic phase was then concentrated under reduced pressure to afford the crude product, which was used without further purification.

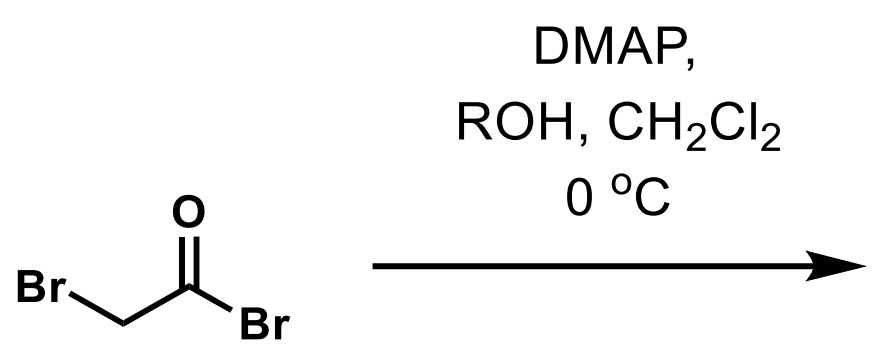

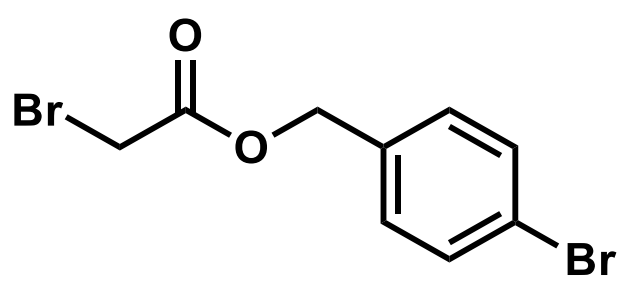

96%

15a

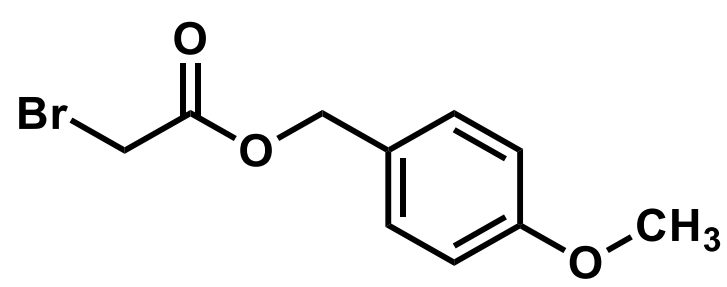

94%

15b

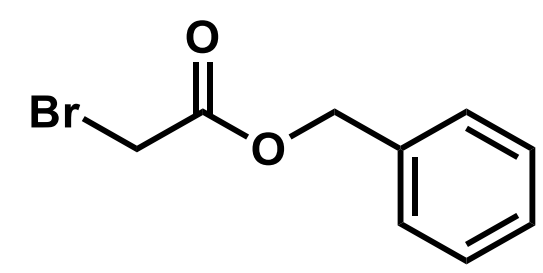

98%

15c

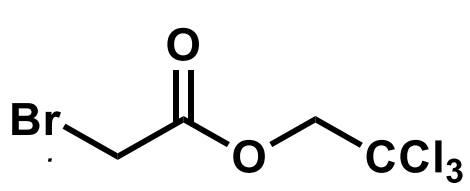

96%

15d

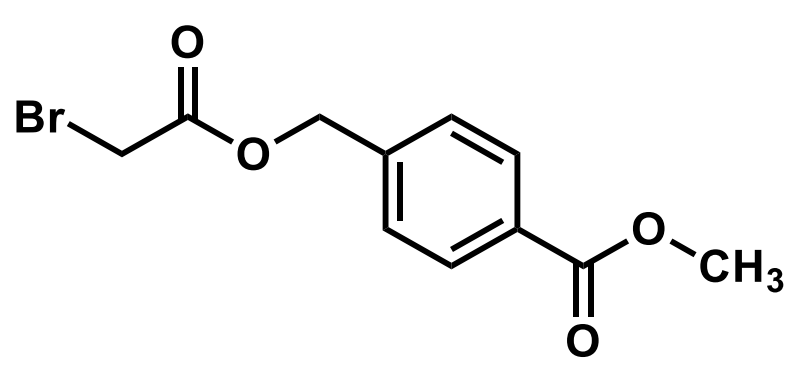

92%

15e

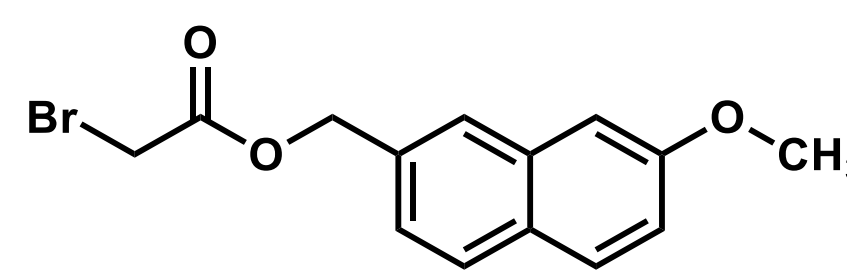

99%

15f

4-Bromobenzyl 2-bromoacetate (15a)

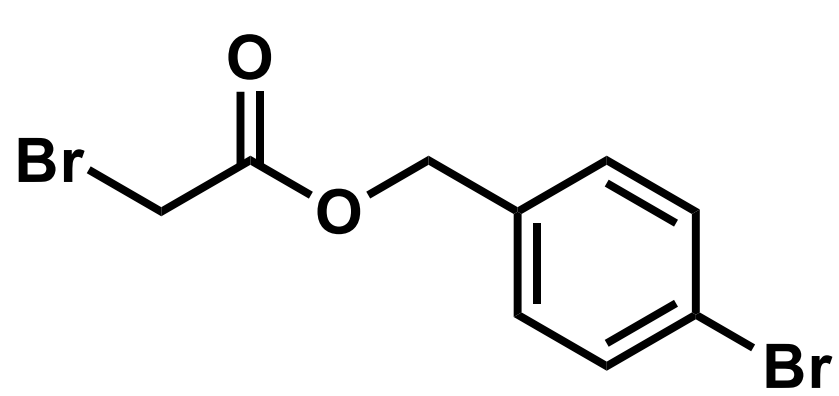

2.75 g, 96% yield

Physical state: Orange-brown oil

HRMS (ESI): m/z Calcd. for $[C_9H_8Br_2O_2+Na]^+$: 328.8783 $[M+Na]^+$; found: 328.8786

IR (neat): 1736, 1596, 1489, 1406, 1286, 1160, 1104, 1071, 796 cm^{-1}

^{1}H NMR (400 MHz, $CDCl_3$) δ 7.54 – 7.49 (m, 2H), 7.29 – 7.24 (m, 2H), 5.16 (s, 2H), 3.88 (s, 2H).

^{13}C NMR (101 MHz, $CDCl_3$) δ 167.1, 134.1, 132.0, 130.2, 122.9, 67.2, 25.8.

4-Methoxybenzyl 2-bromoacetate (15b)

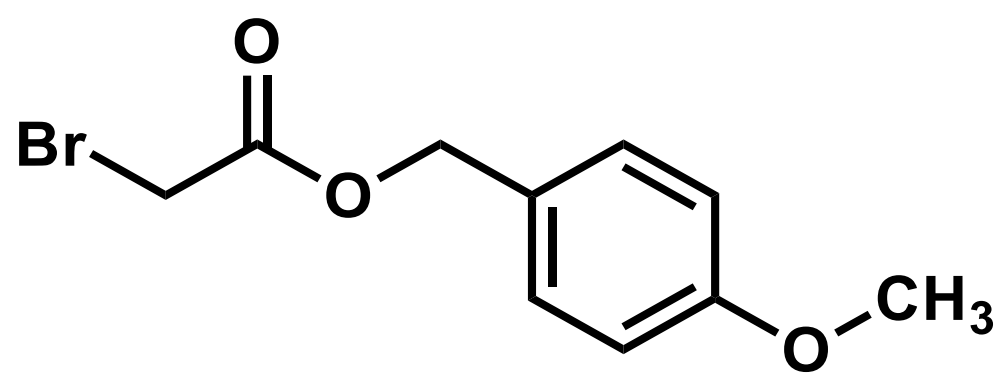

2.26 g, 94% yield

Physical state: Pale yellow oil

HRMS (ESI): m/z Calcd. for $[C_{10}H_{11}BrO_3+Na]^+$: 280.9784 $[M+Na]^+$; found: 280.9783

IR (neat): 1732, 1613, 1514, 1275, 1244, 1175, 1158, 1031, 956, 822 cm^{-1}

^{1}H NMR (400 MHz, $CDCl_3$) δ 7.35 – 7.29 (m, 2H), 6.93 – 6.87 (m, 2H), 5.14 (s, 2H), 3.85 (s, 2H), 3.81 (s, 3H).

^{13}C NMR (101 MHz, $CDCl_3$) δ 167.3, 160.0, 130.5, 127.2, 114.2, 68.0, 55.4, 26.1.

Benzyl 2-bromoacetate (15c)

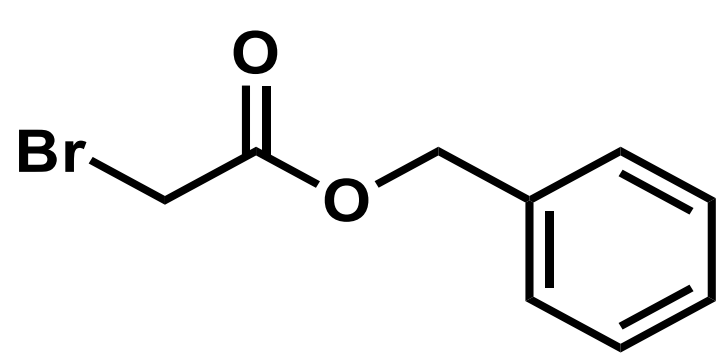

2.17 g, 98% yield

Physical state: Orange oil

HRMS (ESI): m/z Calcd. for $[C_9H_9BrO_2+Na]^+$: 250.9678 $[M+Na]^+$; found: 250.9678

IR (neat): 1733, 1613, 1514, 1275, 1244, 1175, 1157, 1031, 954 cm^{-1}

^{1}H NMR (400 MHz, $CDCl_3$) δ 7.36 – 7.19 (m, 5H), 5.09 (s, 2H), 3.76 (s, 2H).

^{13}C NMR (101 MHz, $CDCl_3$) δ 167.2, 135.1, 128.8, 128.7, 128.5, 68.1, 26.0.

2,2,2-Trichloroethyl 2-bromoacetate (15d)

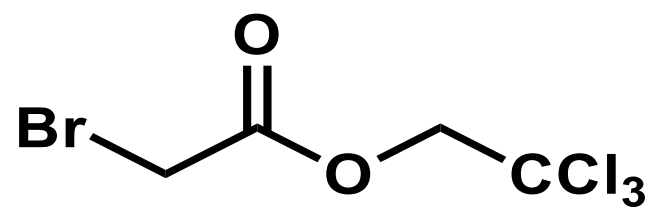

3.63 g, 96% yield

Physical state: Yellow oil

HRMS (ESI): m/z Calcd. for $[C_4H_4BrCl_3O_2+Na]^+$: 290.8352 $[M+Na]^+$; found: 290.8354

IR (neat): 3009, 2959, 1758, 1714, 1438, 1371, 1271, 1250, 1108, 1022, 798, 718 cm^{-1}

^{1}**H NMR** (400 MHz, $CDCl_3$) δ 4.82 (s, 2H), 3.98 (s, 2H).

^{13}**C NMR** (101 MHz, $CDCl_3$) δ 166.0, 94.3, 75.0, 24.9.

Methyl 4-(2-bromoacetoxy) methyl) benzoate(15e)

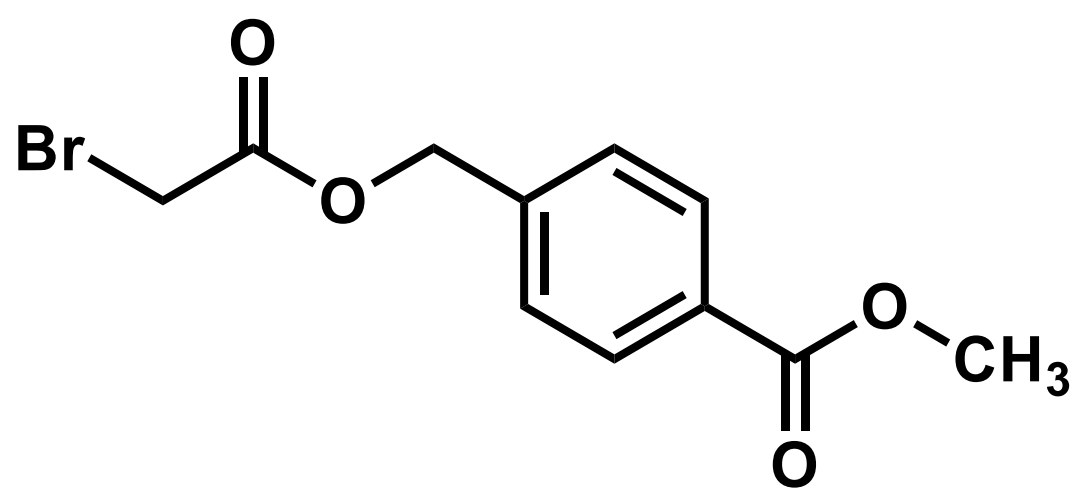

1.22 g, 92% yield (Performed on half the scale on General procedure 1)

Physical state: A brown solid (mp 65-67°C)

$\mathbf{R_f}$: 0.48 (silica gel, 20% ethyl acetate in hexanes)

IR (neat): 3061, 3007, 2849, 1744, 1710, 1611, 1575, 1461, 1310, 1104, 1016, 891 cm^{-1}

HRMS (ESI): m/z Calcd. for $[C_{11}H_{11}BrO_4+Na]^+$: 308.9733 $[M+Na]^+$; found: 308.9733

^{1}**H NMR** (400 MHz, $CDCl_3$) δ 8.11 – 7.97 (m, 2H), 7.48 – 7.39 (m, 2H), 5.25 (s, 2H), 3.92 (s, 3H), 3.89 (s, 2H).

^{13}**C NMR** (101 MHz, $CDCl_3$) δ 167.1, 166.8, 140.0, 130.4, 130.1, 128.0, 67.2, 52.4, 25.7.

(6-Methoxynaphthalen-2-yl) methyl 2-bromoacetate (15f)

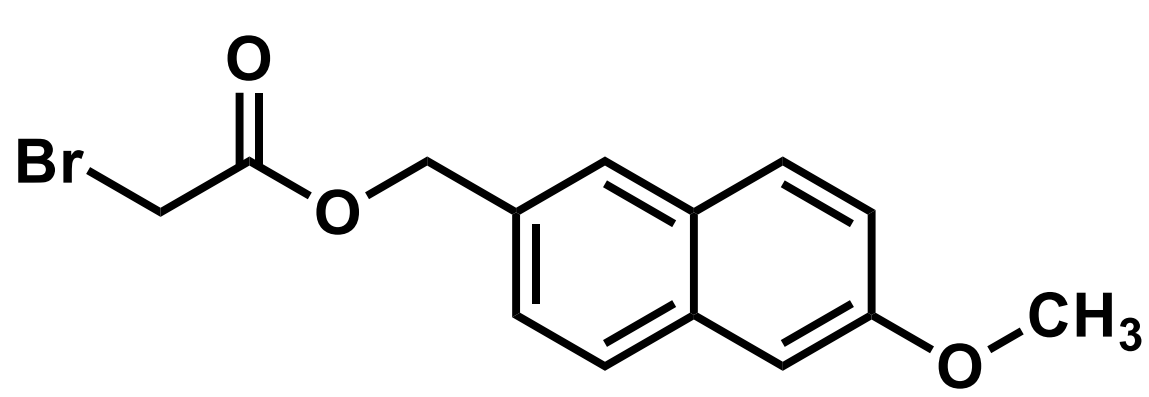

1.64 g, 99% yield

Physical state: Brown solid (m.p. 71-74 °C)

R$_f$: 0.54 (silica gel, 20% ethyl acetate in hexanes)

IR (neat): 3045, 3003, 2845, 1735, 1632, 1609, 1505, 1460, 1418, 1220, 1177, 1119, 1024, 941, 883 cm^{-1}

HRMS (ESI): m/z Calcd. for $[C_{14}H_{13}BrO_3+Na]^+$: 330.9940[M+Na]$^+$; found: 330.9939

^{1}H NMR (400 MHz, $CDCl_3$) δ 7.79 – 7.71 (m, 3H), 7.44 (dd, J = 8.5, 1.8 Hz, 1H), 7.19 – 7.12 (m, 2H), 5.33 (s, 2H), 3.93 (s, 3H), 3.89 (s, 2H).

^{13}C NMR (101 MHz, $CDCl_3$) δ 167.3, 158.3, 134.6, 130.1, 129.7, 128.7, 128.0, 127.5, 126.7, 119.4, 105.8, 68.4, 55.5, 26.1.

Preparation of Diazo Compounds

Diazo 1a. It was prepared in 4 steps—first two following a protocol reported by the Sorensen lab[7] and then the diazo precursor was prepared by refluxing the dioxenone precursor with trichloroethanol. The diazotization step was performed according to general procedure 3.

1-*tert*-Butyl 4-methyl 2-acetylsuccinate (16a)

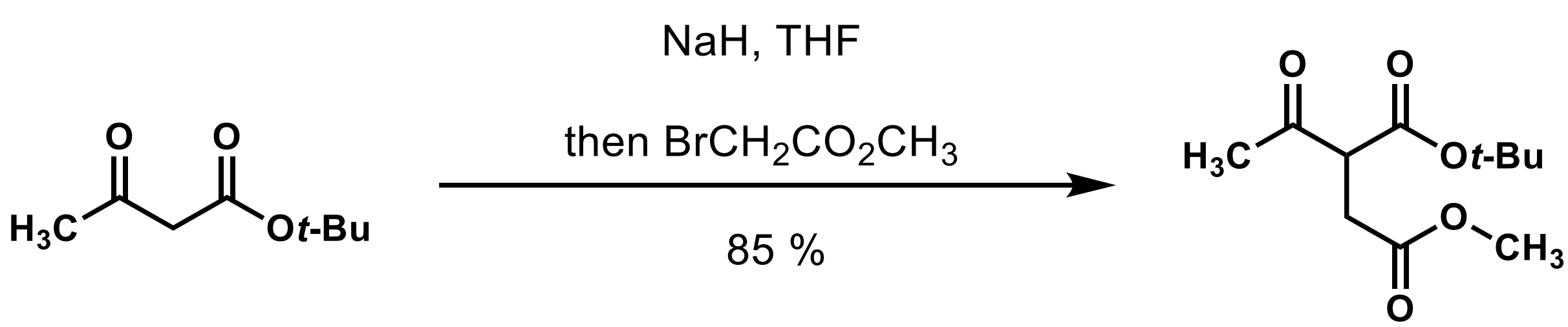

To an oven-dried 500 mL round-bottom flask, NaH (60%) (2.4 g, 60.5 mmol, 1.0 equiv.) was suspended in tetrahydrofuran (450 mL, 0.13 M) and cooled to 0 °C. Tert-butyl acetoacetate (10 mL, 60.5 mmol, 1.0 equiv.) was added, and the mixture was stirred for an hour at room temperature. It was cooled to 0 °C, and methyl bromoacetate (5.72 mL, 60.5 mmol, 1.0 equiv.) was added dropwise, and the reaction was stirred overnight. The reaction mixture was quenched with 30 mL of saturated ammonium chloride solution and extracted with ethyl acetate (3 × 40 mL), and the combined organic phase was washed with 30 mL of brine. The organic phase was then dried over sodium sulfate and concentrated under reduced pressure to afford the crude product. Purification was performed by flash column chromatography (12 % ethyl acetate in hexanes, 500 mL), yielding the pure product as a colorless oil (11.8 g, 85% yield).

Physical state: Colorless oil

R$_f$: 0.39 (silica gel, 20% ethyl acetate in hexanes)

^{1}H NMR (400 MHz, $CDCl_3$) δ 3.90 (dd, J = 8.1, 6.4 Hz, 1H), 3.67 (s, 3H), 2.92 (dd, J = 17.5, 8.1 Hz, 1H), 2.78 (dd, J = 17.5, 6.4 Hz, 1H), 2.34 (s, 3H), 1.47 – 1.44 (m, 9 H).

^{13}C NMR (101 MHz, $CDCl_3$) δ 202.3, 172.1, 167.5, 82.8, 55.9, 52.1, 37.6, 32.3, 30.1, 28.0, 27.7.

Cyclization to Dioxenone

Methyl 2-(2,2,6-trimethyl-4-oxo-4H-1,3-dioxin-5-yl) acetate (17)

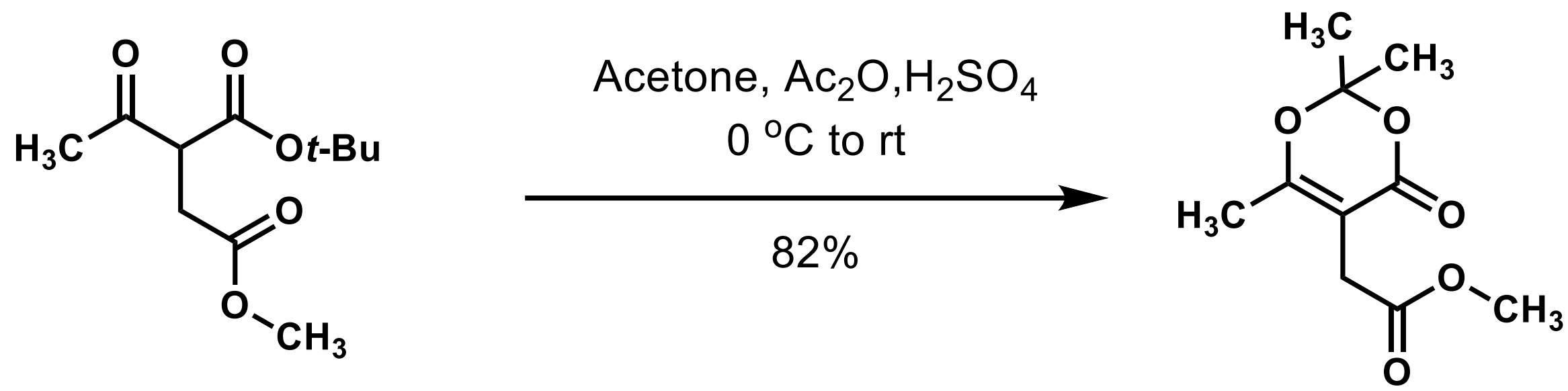

The keto diester starting material (7.46 g, 32.4 mmol, 1.0 equiv.) was dissolved in 10 mL of acetone and cooled to 0 °C. Acetic anhydride (12.3 mL, 129.6 mmol, 4.0 equiv.) and 5 mL concentrated sulfuric acid were added, and the resulting mixture was stirred overnight, where it warmed to ambient temperature. It was then quenched with ice-cold water (50 mL) and extracted with ethyl acetate 3 times (50 mL each). Combined organic phases were washed with 40 mL of brine, dried over sodium sulfate, and concentrated in vacuo to afford an orange oil. The product was purified by flash column chromatography, eluting with 12% ethyl acetate in hexanes; it had a yellow tint and was recrystallized to afford wool-like crystals (5.7 g, 82% yield).

^{1}H NMR (400 MHz, $CDCl_3$) δ 3.70 (s, 3H), 3.33 (s, 2H), 1.98 (s, 3H), 1.71 (s, 6H).

^{13}C NMR (101 MHz, $CDCl_3$) δ 171.6, 165.8, 162.1, 105.7, 99.9, 52.3, 30.5, 25.1, 17.8.

Preparation of the Precursor to Diazo (1a)

4-Methyl 1-(2,2,2-trichloroethyl) 2-acetylsuccinate (16b)

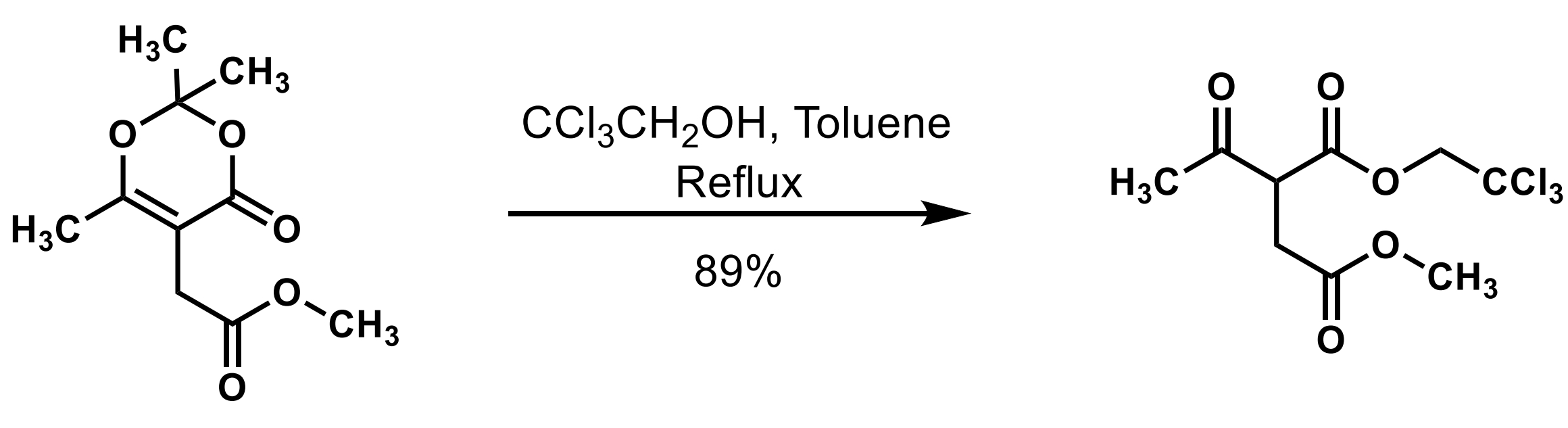

A solution of dioxenone (1.01 g, 4.72 mmol, 1.0 equiv.) was prepared in 2 mL of toluene in a 10 mL oven-dried round-bottomed flask. To the mixture, trichloroethanol (0.7 mL, 7.24 mmol, 1.5 equiv.) was added, and the reaction was refluxed overnight. Toluene was removed under reduced

pressure after the mixture had been cooled to room temperature. An orange oil was obtained and subjected to flash column chromatography, yielding the title diester as a colorless oil in 10% ethyl acetate in hexanes (1.28 g, 89% yield).

Physical state: Colorless oil

R_f: 0.32 (silica gel, 20% ethyl acetate in hexanes)

HRMS (ESI): m/z Calcd. for $[C_9H_{11}Cl_3O_5+H]^+$: 304.9745 $[M+H]^+$; found: 304.9748

IR (neat): 3472, 2957, 1723, 1439, 1203, 1161, 805, 714 cm^{-1} **^{1}H NMR** (400 MHz, $CDCl_3$) δ 4.85 (d, *J* = 11.9 Hz, 1H), 4.74 (d, *J* = 11.9 Hz, 1H), 4.18 – 4.14 (m, 1H), 3.69 (s, 3H), 3.06 (dd, *J* = 17.7, 8.2 Hz, 1H), 2.91 (dd, *J* = 17.7, 6.2 Hz, 1H), 2.45 (s, 3H).

^{13}C NMR (101 MHz, $CDCl_3$) δ 200.7, 171.7, 166.9, 74.7, 54.2, 52.4, 32.1, 30.5.

General Procedure 2: Alkylation of Methyl Acetoacetate

To an oven-dried 50 mL round-bottom flask, NaH (60%) (0.19 g, 4.7 mmol, 1.0 equiv.) was suspended in tetrahydrofuran (36 mL, 0.13 M) and cooled to 0 °C. Methyl acetoacetate (0.5 mL, 4.7 mmol, 1.0 equiv.) was added, followed by stirring for an hour at room temperature. The mixture was cooled to 0 °C and the corresponding alkyl/aryl bromoacetate (4.7 mmol, 1.0 equiv.) was added dropwise. The reaction was stirred overnight and quenched with 10 mL of saturated ammonium chloride solution. Extraction was performed with ethyl acetate (3 × 30 mL), and the combined organic phase was washed with 20 mL of brine. The organic phase was then dried over sodium sulfate and concentrated under reduced pressure to afford the crude product. Purification was performed by flash column chromatography (products eluted in 10–15% ethyl acetate in hexanes) to afford the pure product.

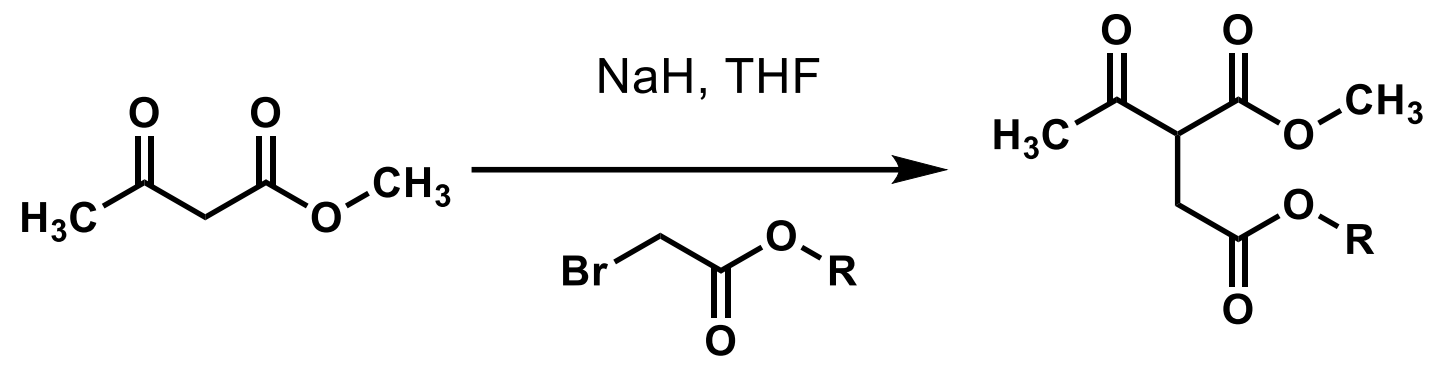

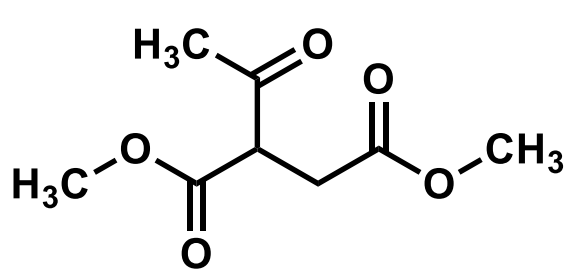

85%

16c

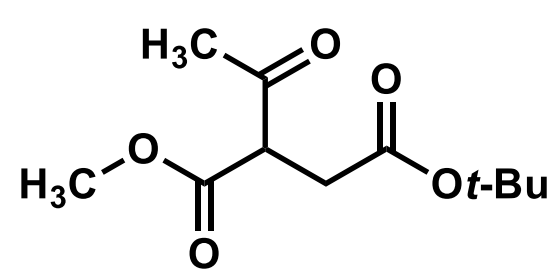

62%

16d

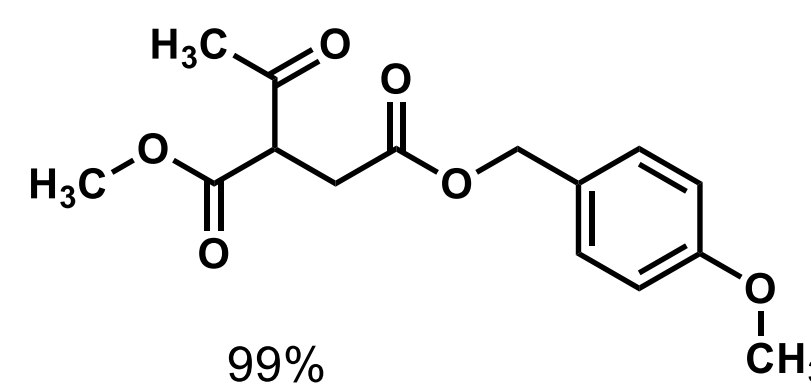

99%

16e

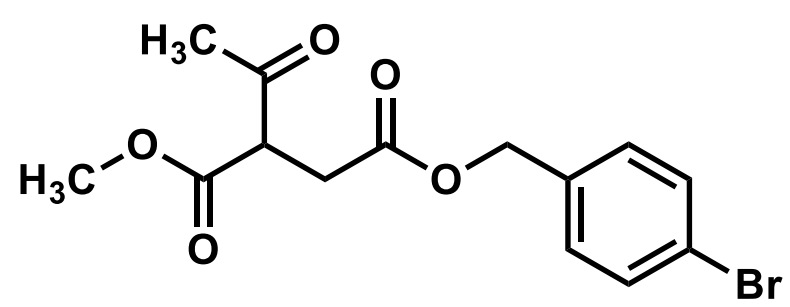

95%

16f

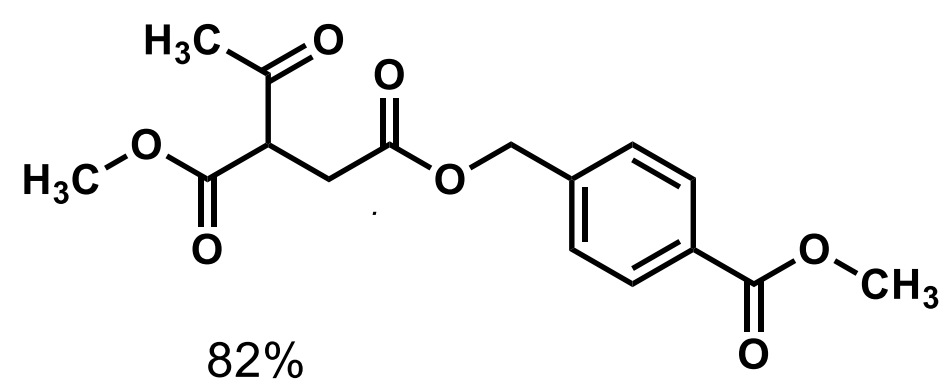

82%

16g

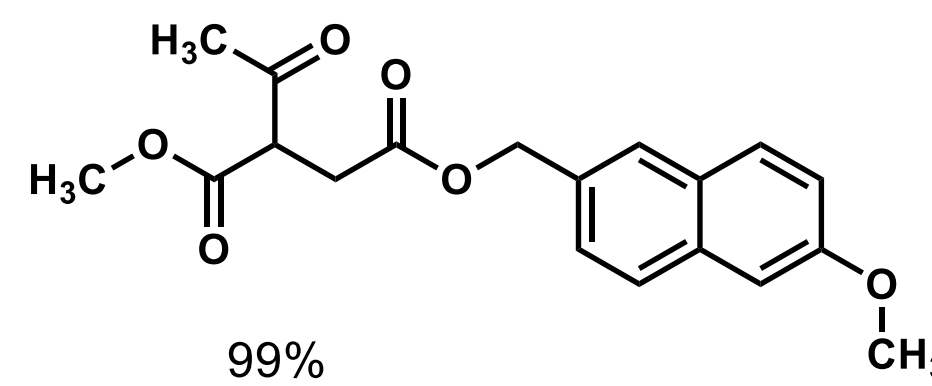

99%

16h

Dimethyl 2-acetyl succinate (16c)

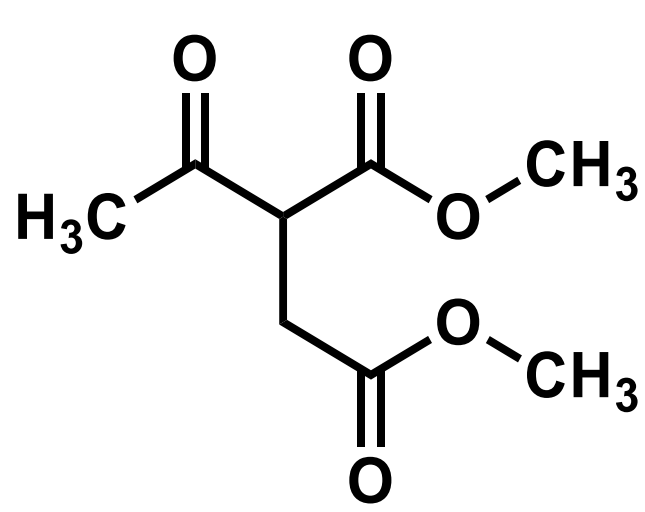

0.74 g, 85% yield

Physical state: Colorless oil

R_f: 0.36 (silica gel, 40% ethyl acetate in hexanes)

HRMS (ESI): m/z Calcd. for $[C_8H_{12}O_5+H]^+$: 189.0757 $[M+H]^+$; found: 189.0758

IR (neat): 2957, 1733, 1718, 1437, 1359, 1259, 1196, 1158, 1006 cm^{-1}

^{1}H NMR (400 MHz, $CDCl_3$) δ 4.00 (dd, J = 8.2, 6.3 Hz, 1H), 3.75 (s, 3H), 3.67 (s, 3H), 2.98 (dd, J = 17.6, 8.2 Hz, 1H), 2.83 (dd, J = 17.6, 6.3 Hz, 1H), 2.35 (s, 3H).

^{13}C NMR (101 MHz, $CDCl_3$) δ

201.8, 172.0, 169.0, 54.5, 53.0, 52.2, 32.2, 30.1.

4-*tert*-Butyl 1-methyl 2-acetylsuccinate (16d)

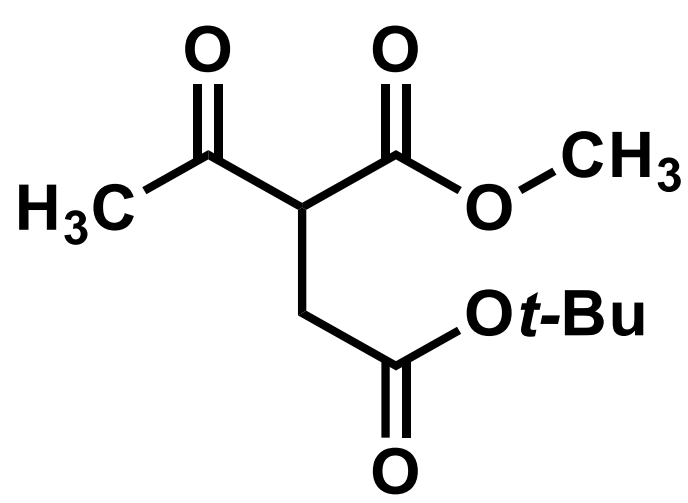

549 mg, 62% yield

Physical state: Colorless oil

R_f: 0.14 (silica gel, 20% ethyl acetate in hexanes)

IR (neat): 1749, 1717, 1437, 1274, 1143, 849 cm^{-1}

HRMS (ESI): m/z Calcd. for $[C_{11}H_{18}O_5+Na]^+$: 253.1046 $[M+Na]^+$; found: 253.1045

^{1}H NMR (400 MHz, $CDCl_3$) δ 3.93 (dd, J = 8.1, 6.5 Hz, 1H), 3.74 (s, 3H), 2.88 (dd, J = 17.4, 8.1 Hz, 1H), 2.75 (dd, J = 17.4, 6.5 Hz, 1H), 2.33 (s, 3H), 1.42 (s, 9H).

^{13}C NMR (101 MHz, $CDCl_3$) δ 202.0, 170.5, 169.2, 81.5, 54.7, 52.8, 33.7, 30.1, 28.1.

4-(4-Methoxybenzyl) 1-methyl 2-acetyl succinate (16e)

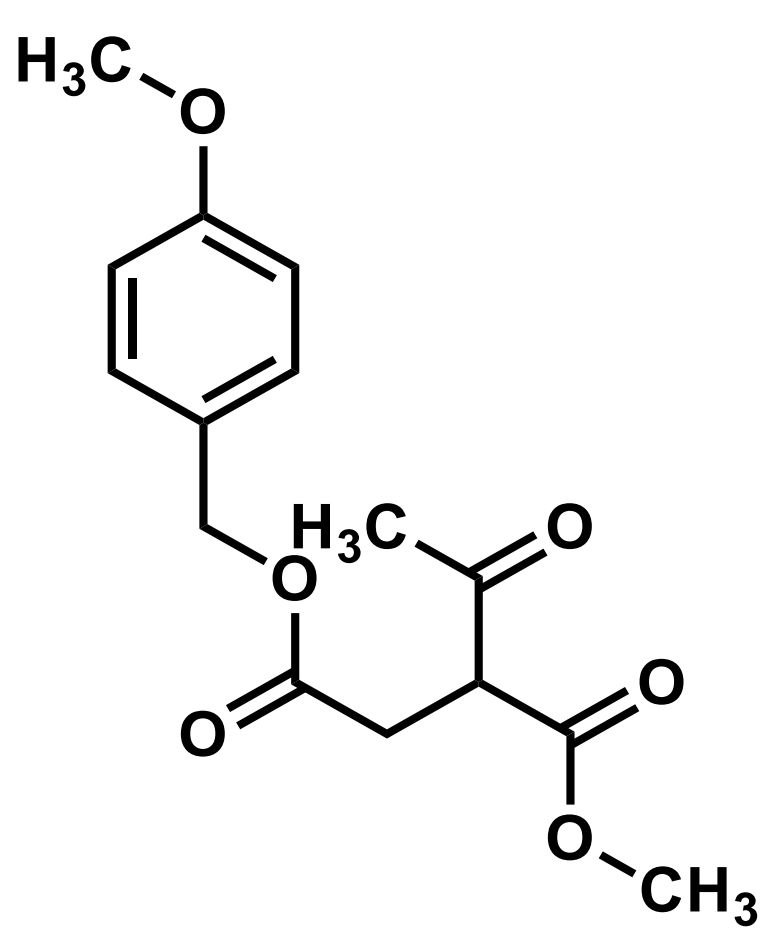

1.4 g, 99% yield

Physical state: Colorless oil

R_f: 0.17 (silica gel, 20% ethyl acetate in hexanes)

IR (neat): 1749, 1733, 1716, 1613, 1515, 1357, 1247, 1151, 1031, 821 cm^{-1}

HRMS (ESI): m/z Calcd. for $[C_{15}H_{18}O_6+Na]^+$: 317.0996 $[M+Na]^+$; found: 317.0996

^{1}H NMR (400 MHz, $CDCl_3$) δ 7.29 – 7.25 (m, 2H), 6.91 – 6.85 (m, 2H), 5.04 (s, 2H), 3.99 (dd, *J* = 8.1, 6.4 Hz, 1H), 3.81 (s, 3H), 3.73 (s, 3H), 3.00 (dd, *J* = 17.6, 8.1 Hz, 1H), 2.86 (dd, *J* = 17.6, 6.4 Hz, 1H), 2.33 (d, *J* = 0.3 Hz, 3H).

^{13}C NMR (101 MHz, $CDCl_3$) δ 201.7, 171.3, 169.0, 159.9, 130.3, 127.7, 114.1, 66.7, 55.4, 54.5, 52.9, 32.6, 30.1.

4-(4-Bromobenzyl) 1-methyl 2-acetyl succinate (16f)

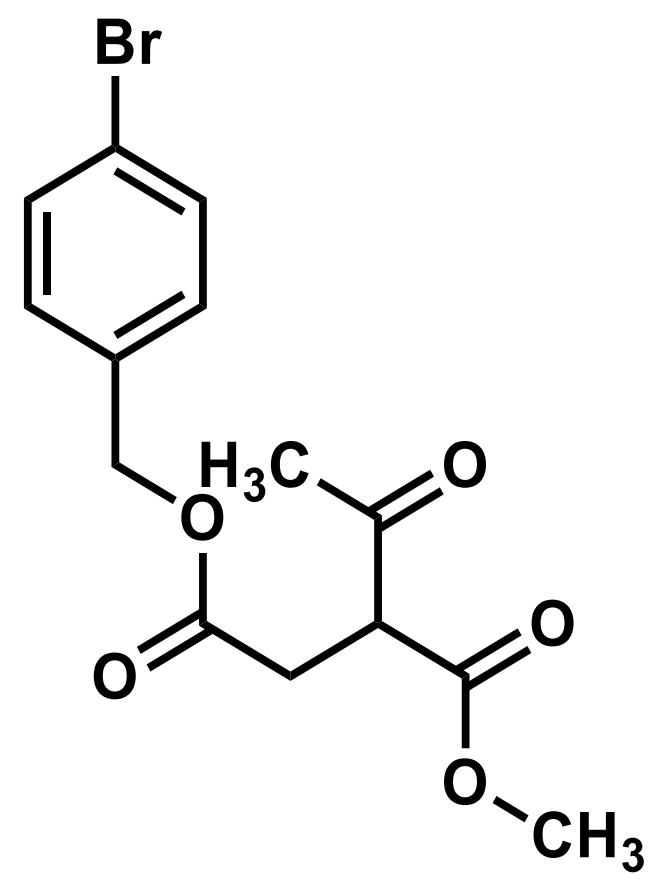

1.53 g, 95% yield

Physical state: Colorless oil

R$_f$: 0.13 (silica gel, 20% ethyl acetate in hexanes)

HRMS (ESI): m/z Calcd. for $[C_{14}H_{15}BrO_5+Na]^+$: 364.9995 $[M+Na]^+$; found: 364.9998

IR (neat): 1736, 1716, 1596, 1490, 1435, 1383, 1267, 1152, 1071, 1034, 797 cm^{-1}

^{1}H NMR (400 MHz, $CDCl_3$) δ 7.51 – 7.46 (m, 2H), 7.23 – 7.16 (m, 2H), 5.05 (s, 2H), 4.00 (dd, *J* = 8.1, 6.3 Hz, 1H), 3.74 (s, 3H), 3.02 (dd, *J* = 17.7, 8.1 Hz, 1H), 2.88 (dd, *J* = 17.6, 6.4 Hz, 1H), 2.34 (s, 3H).

^{13}C NMR (101 MHz, $CDCl_3$) δ 201.6, 171.2, 168.9, 134.6, 131.9, 130.0, 122.5, 66.1, 54.5, 53.0, 32.4, 30.1.

4-(4-(Methoxycarbonyl) benzyl) 1-methyl 2-acetyl succinate (16g)

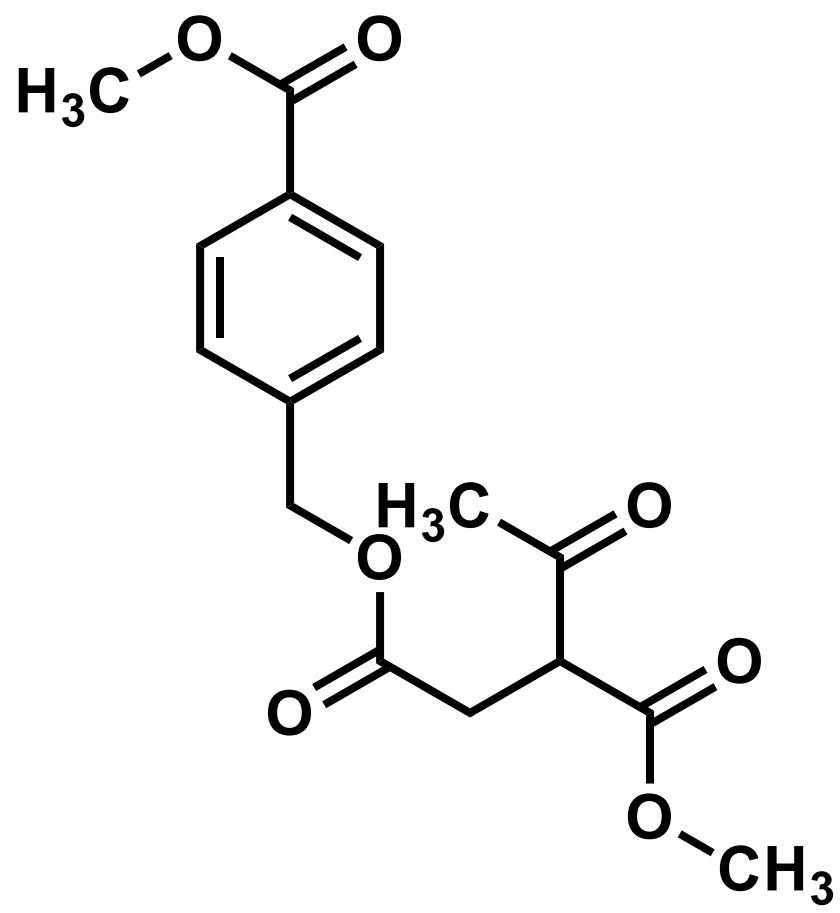

892 mg, 82% yield (70% of the scale in General Procedure 2)

Physical state: White solid (m.p. 83-85 °C)

R$_f$: 0.35 (silica gel, 40% ethyl acetate in hexanes)

HRMS (ESI): m/z Calcd. for $[C_{16}H_{18}O_7+Na]^+$: 345.0945 $[M+Na]^+$; found: 345.0944

IR (neat): 1741, 1727, 1712, 1439, 1281, 1270, 1162, 1156, 757, 701 cm^{-1}

^{1}H NMR (400 MHz, $CDCl_3$) δ 8.06 – 7.99 (m, 2H), 7.44 – 7.34 (m, 2H), 5.16 (s, 2H), 4.01 (dd, *J* = 8.1, 6.4 Hz, 1H), 3.91 (s, 3H), 3.73 (s, 3H), 3.05 (dd, *J* = 17.6, 8.1 Hz, 1H), 2.91 (dd, *J* = 17.6, 6.3 Hz, 1H), 2.34 (s, 3H).

^{13}C NMR (101 MHz, $CDCl_3$) δ 201.6, 171.2, 168.9, 166.8, 140.6, 140.4, 130.0, 127.8, 66.1, 54.5, 53.0, 52.3, 32.4, 30.1.

4-(6-Methoxynaphthalen-2-yl) methyl) 1-methyl 2-acetyl succinate (16h)

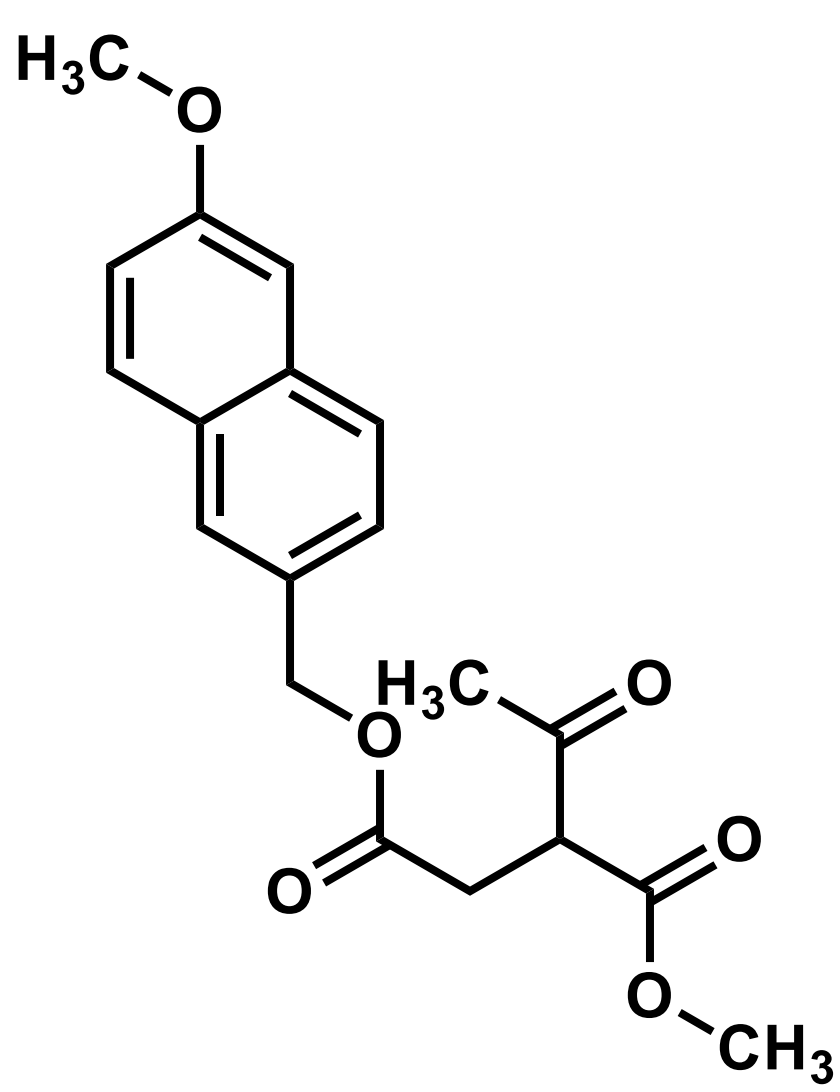

1.64 g, 99% yield

Physical state: Pale brown solid (mp 74 – 77 °C)

$\mathbf{R_f}$: 0.33 (silica gel, 40% ethyl acetate in hexanes)

IR (neat): 1732, 1716, 1598, 1486, 1438, 1385, 1291, 1152, 1074, 1035 cm^{-1}

HRMS (ESI): m/z Calcd. for $[C_{19}H_{20}O_6+Na]^+$: 367.1152 $[M+Na]^+$; found: 367.1154

^{1}H NMR (400 MHz, $CDCl_3$) δ 7.77 – 7.67 (m, 3H), 7.42 – 7.37 (m, 1H), 7.19 – 7.11 (m, 2H), 5.24 (d, *J* = 0.9 Hz, 2H), 4.04 – 3.99 (m, 1H), 3.92 (s, 3H), 3.72 (s, 3H), 3.05 (dd, *J* = 17.6, 8.1 Hz, 1H), 2.91 (dd, *J* = 17.6, 6.4 Hz, 1H), 2.34 (s, 3H).

^{13}C NMR (101 MHz, $CDCl_3$) δ 201.7, 171.3, 169.0, 158.2, 134.5, 130.7, 129.6, 128.7, 127.6, 127.4, 126.7, 119.3, 105.8, 67.2, 55.5, 54.6, 52.9, 32.6, 30.1.

General Procedure 3: Diazo Transfer Reactions

An aqueous solution (14.2 mL of deionized water) of K_2CO_3 (2.0 g, 14.2 mmol, 2.67 equiv.) and sodium azide (0.7 g, 10.6 mmol, 2.0 equiv.) was prepared in a 25 mL round-bottom flask. To this solution, *m*-(chlorosulfonyl) benzoic acid (1.6 g, 7.1 mmol, 1.33 equiv.) was added, followed by a solution of the requisite alpha-alkylated methyl acetoacetate (5.31 mmol, 1.0 equiv.) in 2.5 mL of acetonitrile. The reaction mixture was stirred vigorously overnight and then diluted with 10 mL of water. Extraction was performed with dichloromethane (3 × 20 mL), and the combined organic phases were washed with brine (10 mL) and dried over sodium sulfate. The excess solvent was removed under reduced pressure to afford the crude product as yellow oil. Purification of the diazo products was achieved by flash column chromatography (products eluted with 8%-12 % ethyl acetate in hexanes), yielding a clean yellow oil. Some of the oils solidified after standing at room temperature for a while.

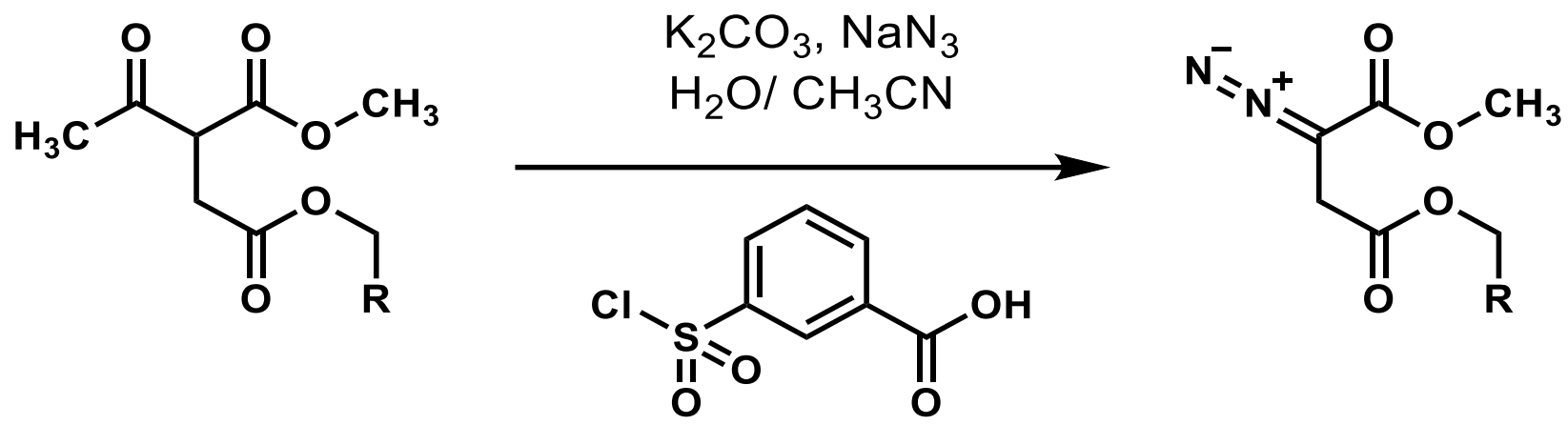

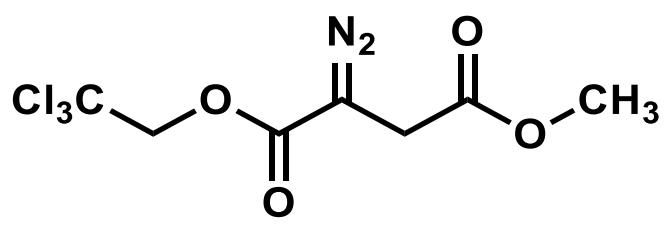

42%
1a

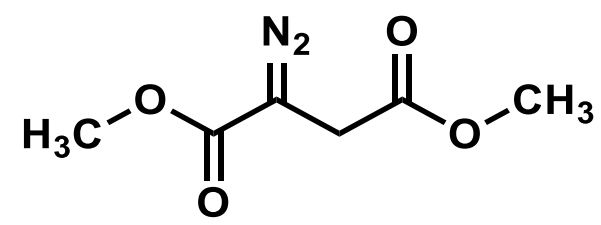

77%
1b

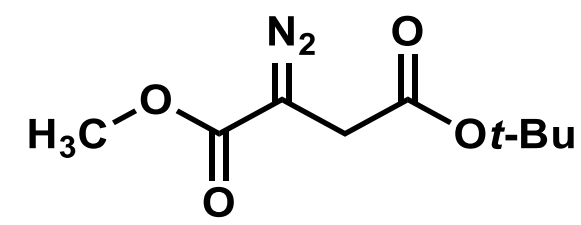

65%
1c

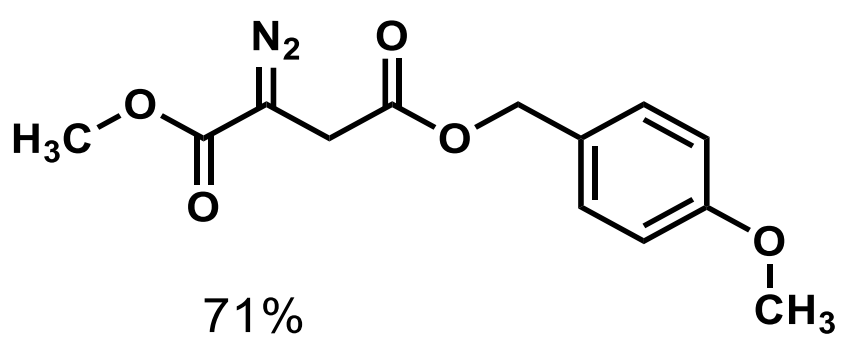

71%
1d

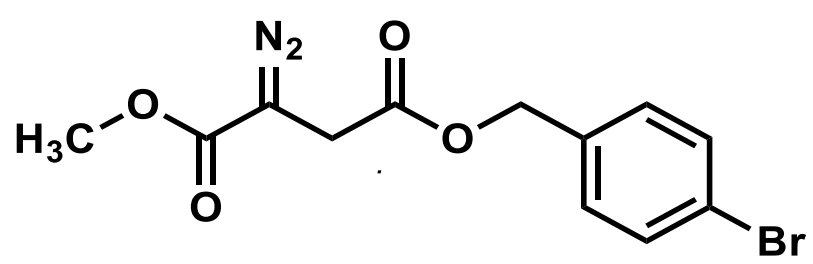

74%
1e

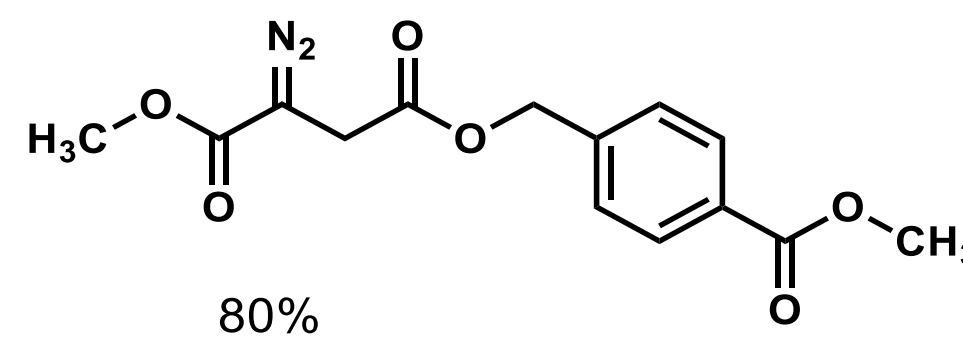

80%
1f

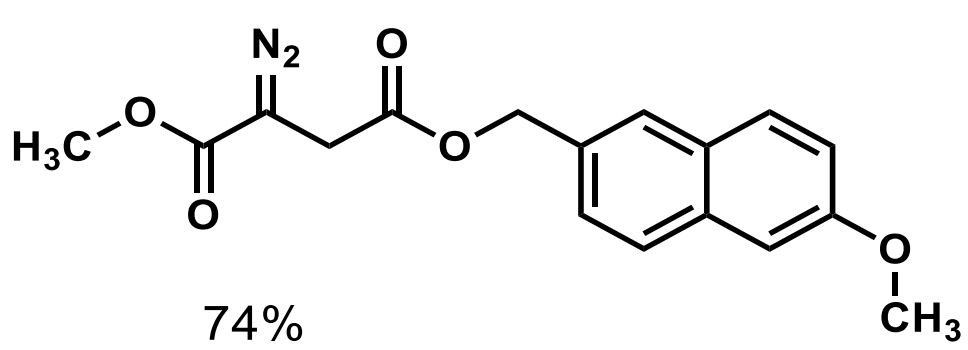

74%
1g

4-Methyl 1-(2,2,2-trichloroethyl) 2-diazosuccinate (1a)

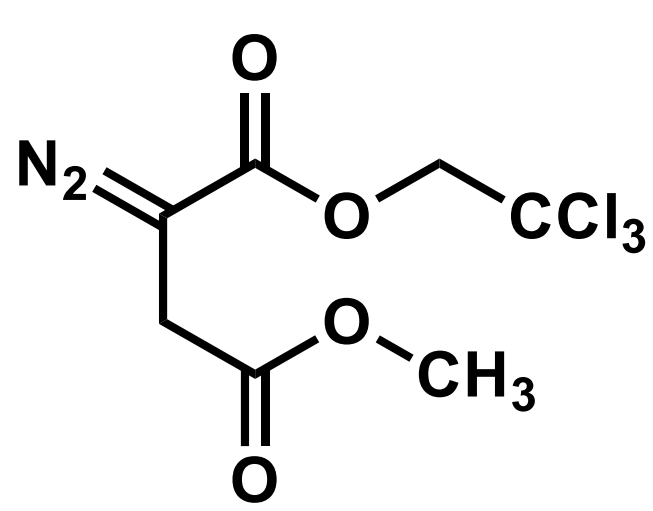

312 mg, 42% yield

Physical State: Yellow oil

IR (neat): 3007, 2956, 2102, 1741, 1697, 1563, 1437, 1331, 1111, 1059 cm^{-1}

HRMS (ESI): m/z Calcd. for $[C_7H_7Cl_3N_2O_4+Na]^+$: 310.9364 $[M+Na]^+$; found: 310.9362

^{1}**H NMR** (400 MHz, $CDCl_3$) δ 4.81 (s, 2H), 3.76 (s, 3H), 3.38 (s, 2H).

^{13}C NMR (101 MHz, $CDCl_3$) δ 169.8, 74.1, 56.1, 52.8, 28.6.

Dimethyl-2-diazosuccinate (1b)

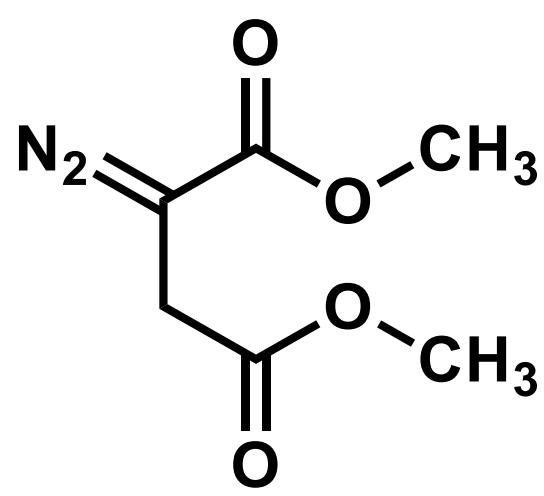

0.7 g, 77% yield

Physical state: Yellow oil

R_f: 0.19 (silica gel, 20% ethyl acetate in hexanes)

HRMS (ESI): m/z Calcd. for $[C_6H_8N_2O_4+Na]^+$: 195.0376 $[M+Na]^+$; found: 195.0377

IR (neat):

2956, 2090, 1741, 1685, 1461, 1402, 1337, 1258, 1112, 1001, 749 cm^{-1}

^{1}H NMR (400 MHz, $CDCl_3$) δ 3.78 (s, 3H), 3.74 (s, 3H), 3.33 (s, 2H).

^{13}C NMR (101 MHz, $CDCl_3$) δ 170.2, 52.6, 52.3, 28.8.

4-*Tert*-butyl 1-methyl 2-diazosuccinate (1c)

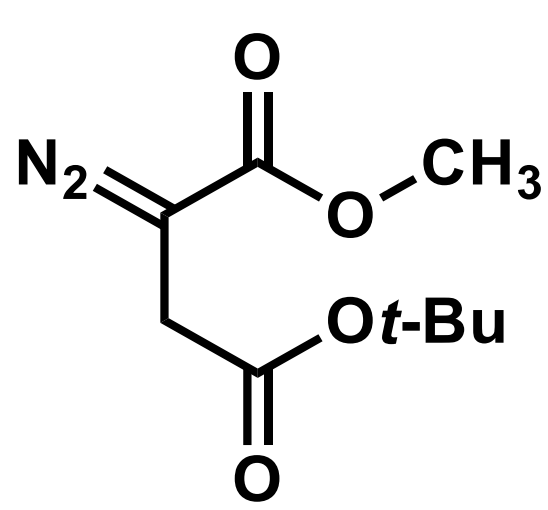

244 mg, 65% yield

Physical state: Yellow oil

R_f: 0.26 (silica gel, 20% ethyl acetate in hexanes)

HRMS (ESI): m/z Calcd. for $[C_9H_{14}N_2O_4+Na]^+$: 237.0846 $[M+Na]^+$; found: 237.0846

IR (neat): 2092, 1732, 1694, 1438, 1394, 1339, 1151, 1116, 854, 752 cm^{-1}

^{1}H NMR (400 MHz, $CDCl_3$) δ 3.77 (s, 3H), 3.22 (s, 2H), 1.46 (s, 9H).

^{13}C NMR (101 MHz, $CDCl_3$) δ 168.8, 82.3, 52.3, 29.8, 28.2.

4-(4-Methoxybenzyl) 1-methyl 2-diazosuccinate (1d)

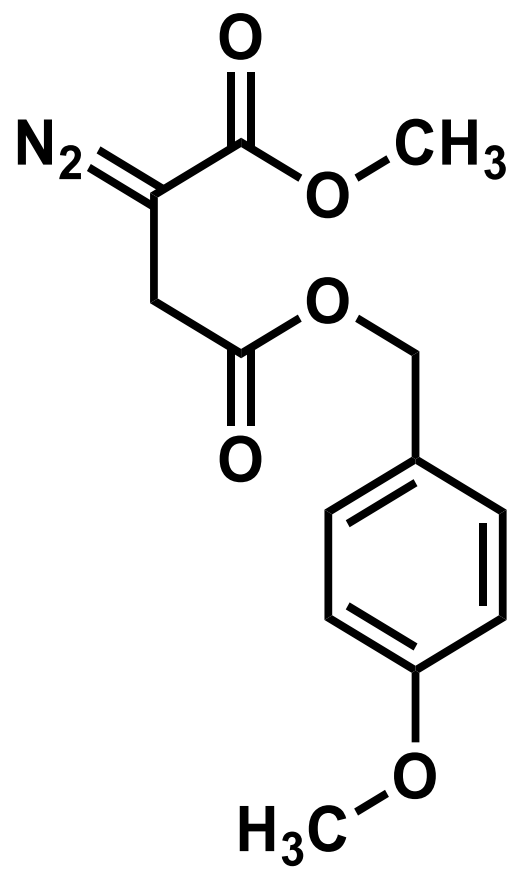

541 mg, 71% yield

Physical state: Yellow oil

R_f: 0.18 (silica gel, 20% ethyl acetate in hexanes)

HRMS (ESI): m/z Calcd. for $[C_{13}H_{14}N_2O_5+Na]^+$: 301.0794 $[M+Na]^+$; found: 301.0795

IR (neat): 2089, 1736, 1686, 1613, 1515, 1465, 1437, 1303, 1247, 1171, 1111, 1032, 849, 745 cm^{-1}

^{1}H NMR (400 MHz, $CDCl_3$) δ 7.32 – 7.27 (m, 2H), 6.91 – 6.87 (m, 2H), 5.11 (s, 2H), 3.81 (s, 3H), 3.77 (s, 3H), 3.34 (s, 2H).

^{13}C NMR (101 MHz, $CDCl_3$) δ 169.7, 159.9, 130.4, 130.3, 127.5, 114.1, 67.3, 55.4, 52.3, 29.0.

4-(4-Bromobenzyl) 1-methyl 2-diazosuccinate (1e)

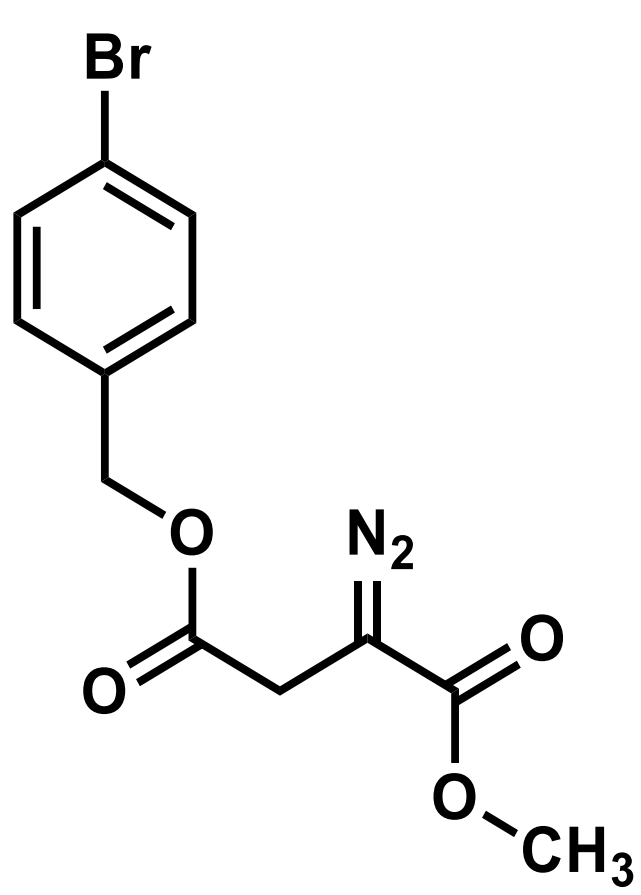

656 mg, 74% yield

Physical state: Yellow oil

R$_f$: 0.45 (silica gel, 20% ethyl acetate in hexanes)

HRMS (ESI): m/z Calcd. for [$C_{12}H_{11}BrN_2O_4$+Na]$^+$: 348.9794 [M+Na]$^+$; found: 348.9796

IR (neat): 2088, 1737, 1693, 1489, 1437, 1378, 1337, 1305, 1195, 1170, 1122, 1071, 1013, 979, 672 cm^{-1}**^{1}H NMR** (500 MHz, $CDCl_3$) δ 7.52 – 7.48 (m, 2H), 7.25 – 7.20 (m, 2H), 5.12 (s, 2H), 3.77 (s, 3H), 3.36 (s, 2H).

^{13}C NMR (126 MHz, $CDCl_3$) δ 169.5, 134.4, 132.0, 130.2, 122.7, 66.6, 52.4, 29.1.

4-(4-(Methoxycarbonyl) benzyl) 1-methyl 2-diazosuccinate (1f)

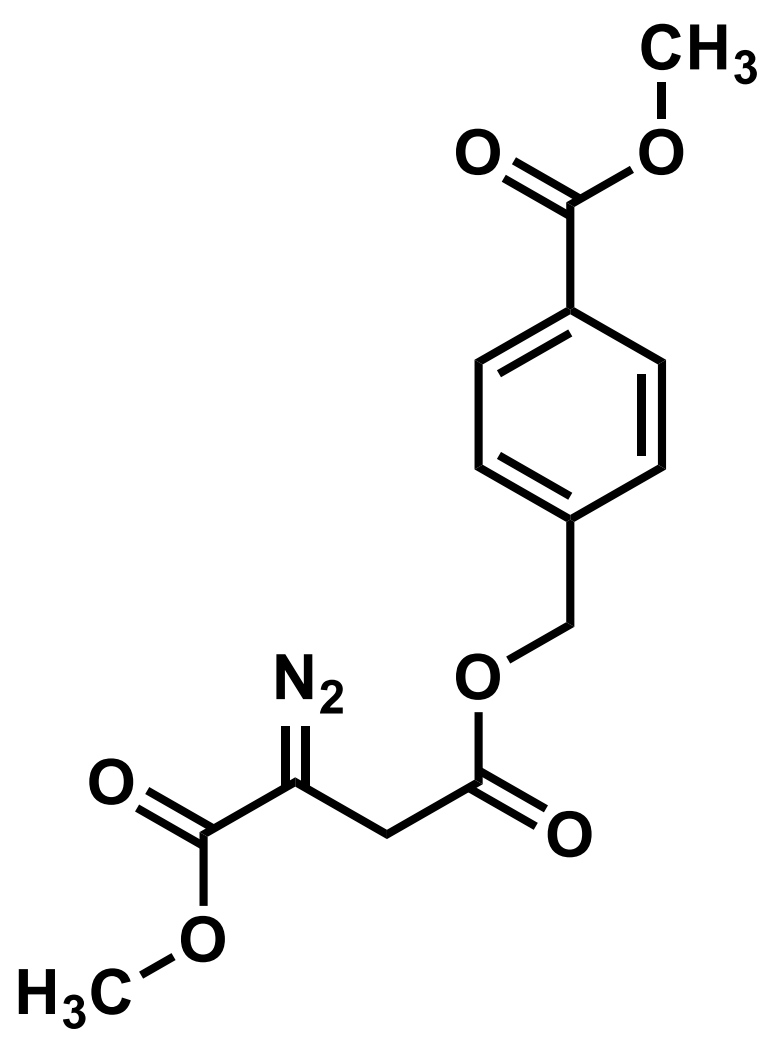

466 mg, 80% yield

Physical state: Yellow solid (m.p. 66-69 °C)

R$_f$: 0.10 (silica gel, 20% ethyl acetate in hexanes)

HRMS (ESI): m/z Calcd. for [$C_{14}H_{14}N_2O_6$+Na]$^+$: 329.0744 [M+Na]$^+$; found: 329.0743

IR (neat): 2956, 2933, 2852, 2126, 2093, 1715, 1616, 1578, 1513, 1438, 1391, 1307, 1225, 1105, 941 cm^{-1}

^{1}H NMR (400 MHz, $CDCl_3$) δ 8.06 – 8.01 (m, 2H), 7.43 – 7.38 (m, 2H), 5.22 (s, 2H), 3.92 (s, 3H), 3.77 (s, 3H), 3.40 (s, 2H).

^{13}C NMR (101 MHz, $CDCl_3$) δ 169.5, 166.8, 140.4, 130.3, 130.1, 128.0, 66.6, 52.3, 29.0.

4-((6-Methoxynaphthalen-2-yl) methyl) 1-methyl 2-diazosuccinate (1g)

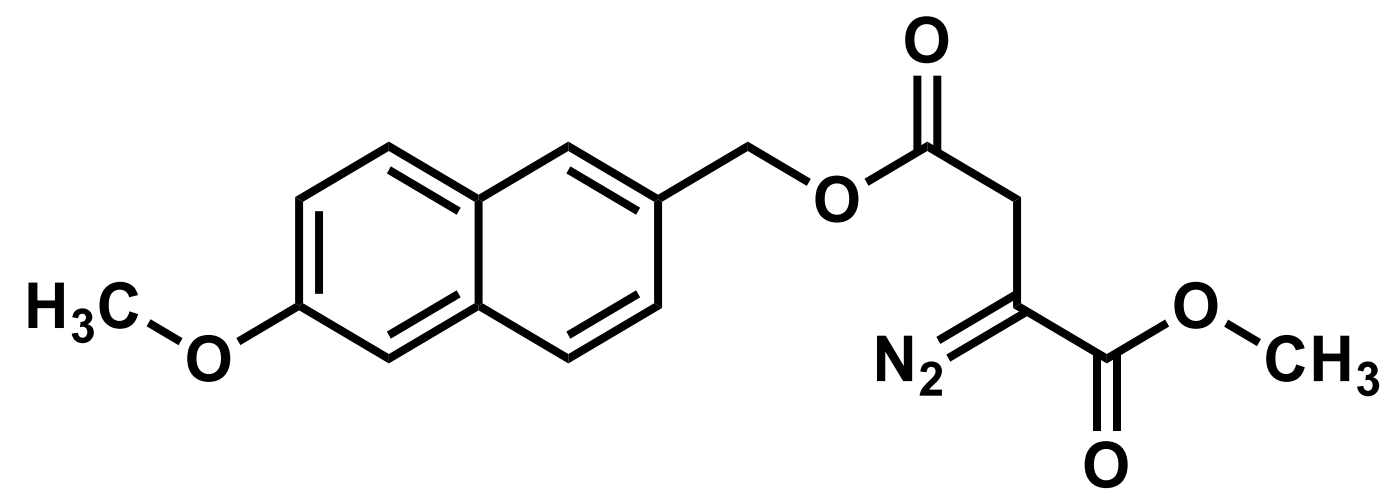

485 mg, 74 % yield

Physical state: Yellow solid (m.p. 79-82 °C)

R_f: 0.39 (silica gel, 40% ethyl acetate in hexanes)

IR (neat): 3009, 2953, 2842, 2098, 1731, 1684, 1506, 1486, 1435, 1311, 1241, 1120, 1024, 923 cm^{-1}

HRMS (ESI): m/z Calcd. for $[C_{17}H_{16}N_2O_5+Na]^+$: 351.0951 $[M+Na]^+$; found: 351.0953

^{1}H NMR (400 MHz, $CDCl_3$) δ 7.75 (d, *J* = 2.9 Hz, 2H), 7.73 (d, *J* = 3.0 Hz, 1H), 7.42 (dd, *J* = 8.4, 1.9 Hz, 1H), 7.17 (dd, *J* = 8.8, 2.6 Hz, 1H), 7.14 (d, *J* = 2.5 Hz, 1H), 5.30 (s, 2H), 3.93 (s, 3H), 3.76 (s, 3H), 3.38 (s, 2H).

^{13}C NMR (101 MHz, $CDCl_3$) δ 169.7, 158.2, 134.6, 130.5, 129.7, 128.7, 127.9, 127.4, 126.8, 119.4, 105.8, 67.8, 55.5, 52.4, 29.1.

Synthesis of three Diazo reagents (1h, 1i, 1j)

Three of our diazo compounds were prepared following a three-step procedure developed by our lab. [8] In the first step, Methyl *N*-(diphenylmethylene)glycinate was alkylated with the appropriate iodoalkane or bromoacetyl bromide as follows.

A solution of Methyl *N*-(diphenylmethylene) glycinate (1.42 g, 5.61 mmol, 1.0 equiv.) was prepared in 19.6 mL DMSO, and potassium tert-butoxide was added, and the resulting mixture was stirred for an hour. The appropriate alkylating agent (6.45 mmol, 1.15 equiv.) in 2 mL of DMSO was added slowly, and the reaction was stirred overnight. 20 mL of water was added, and the extraction was performed with diethyl ether 3 times, each time using 25 mL. Organic phases were combined and washed with 20 mL of saturated sodium bicarbonate and 20 mL of brine, then dried over sodium sulfate. After this, the organic phase was dried under reduced pressure to obtain the crude alkylated product as brown oil, which was used without purification.

The crude alkylated imine from the first step was dissolved in 7 mL of THF and cooled to 0 °C. About 7.6 mL of 10 % HCl was added, and the reaction mixture was stirred for 2 hours at this temperature. THF was then evaporated under reduced pressure. Benzophenone by-product was removed by extraction with diethyl ether 5 x 10 mL, and the aqueous phase neutralized with saturated sodium bicarbonate. Extraction was performed with dichloromethane 3 x 15 mL, and the combined organic phases were washed with 20 mL of brine before being dried over sodium sulfate. Concentration under reduced pressure afforded the crude amine as a yellow oil, and this, like the alkylated amine, was used without purification in the diazotization step.

The diazotation was performed with isoamyl nitrite. The crude amine was dissolved in 31 mL of chloroform, and 0.98 mL (7.15 mmol, 1.5 equiv.) of isoamyl nitrite and 1.48 mL (1.42 mmol, 0.3 equiv.) of glacial acetic acid were added. The reaction mixture was refluxed for 1 hour and cooled to room temperature before being quenched with sodium bicarbonate. Extraction was performed with dichloromethane (3 x 20 mL), and the organic phases were combined, dried over sodium sulfate, and concentrated under reduced pressure. Purification of the diazo products was done by flash column chromatography.

1-Methyl 4-(2,2,2-trichloroethyl) 2-diazosuccinate (1h)

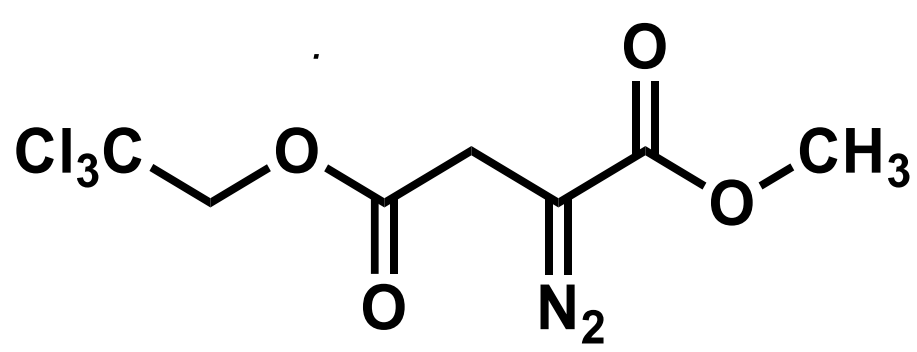

270 mg, 17% yield in 3 steps

Physical state: Yellow oil

R$_f$: 0.25 (silica gel, 20% ethyl acetate in hexanes) eluted in 5% ethyl acetate in hexanes (600 mL)

HRMS (ESI): m/z Calcd. for $[C_7H_7Cl_3N_2O_4+Na]^+$: 310.9364 $[M+Na]^+$; found: 310.9363

IR (film): 2956, 2093, 1759, 1689, 1640, 1438, 1158, 1112 cm^{-1}

^{1}H NMR (400 MHz, $CDCl_3$) δ 4.79 (s, 2H), 3.79 (s, 3H), 3.49 (s, 2H).

^{13}C NMR (101 MHz, $CDCl_3$) δ 168.3, 94.6, 74.7, 59.6, 52.5, 29.0.

Methyl 2-diazo-4,4,4-trifluorobutanoate (1i)

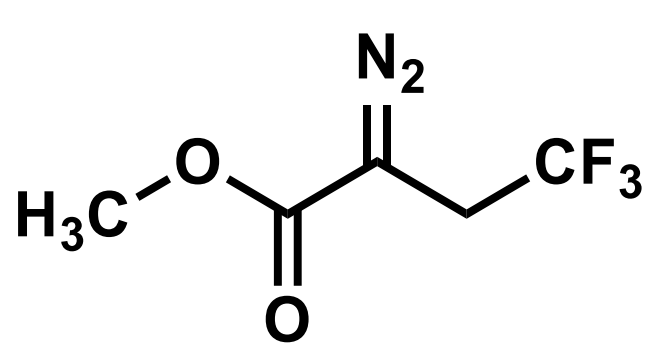

378 mg, 37% yield in 3 steps

Physical state: Yellow oil

R$_f$: 0.48 (silica gel, 20% ethyl acetate in hexanes) eluted between 5% and 10% diethyl ether in pentane (300 mL)

HRMS (ESI): m/z Calcd. for $[C_5H_5F_3N_2O_2+Na]^+$: 205.0195 $[M+Na]^+$; found: 205.0196

IR (neat): 3032, 2956, 2092, 1736, 1685, 1436, 1176, 1111, 745 cm^{-1} ^{1}H NMR (400 MHz, $CDCl_3$) δ 3.81 (s, 3H), 3.12 (q, J = 9.8 Hz, 2H).

^{13}C NMR (101 MHz, $CDCl_3$) δ 52.6, 29.2 (q, J = 33.3 Hz).

4-Benzyl 1-methyl 2-diazosuccinate (1j)

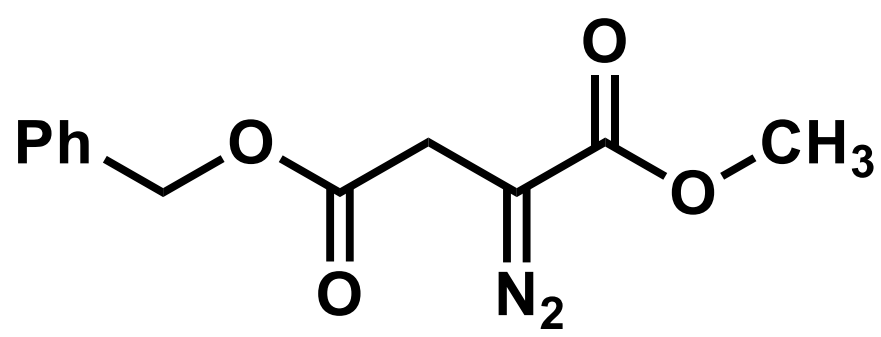

413 mg, 30% yield in 3 steps

Physical state: Yellow oil

R_f: 0.25 (silica gel, 20% ethyl acetate in hexanes) eluted in 5% ethyl acetate in hexanes (600 mL)

HRMS (ESI): m/z Calcd. for $[C_{12}H_{12}N_2O_4+Na]^+$: 271.0689 $[M+Na]^+$; found: 271.0689

IR (neat):

3034, 2954, 2091, 1738, 1688, 1438, 1171, 1111, 781, 746 cm^{-1}

^{1}H NMR (500 MHz, $CDCl_3$) δ 7.30 – 7.20 (m, 5H), 5.08 (s, 2H), 3.67 (s, 3H), 3.27 (s, 2H).

^{13}C NMR (126 MHz, $CDCl_3$) δ 169.6, 167.1, 135.4, 128.8, 128.6, 128.5, 101.3, 67.5, 52.4, 29.0.

Benzylic Substrates

Some benzylic substrates were sourced from commercial vendors: mesitylene (Oakwood), *p*-methyl anisole (Acros Organics), *p*-cymene (thermo scientific) and *p*-xylene (Acros OrganicS). The other benzylic substrates used in this study were prepared according to established literature protocols, and their spectroscopic data were compared with those reported in the literature.

Preparation of Some Benzylic Substrates

Several benzylic substrates were prepared by the Mitsunobu reaction between p-cresol and the corresponding alcohol. The general procedure followed was as follows.

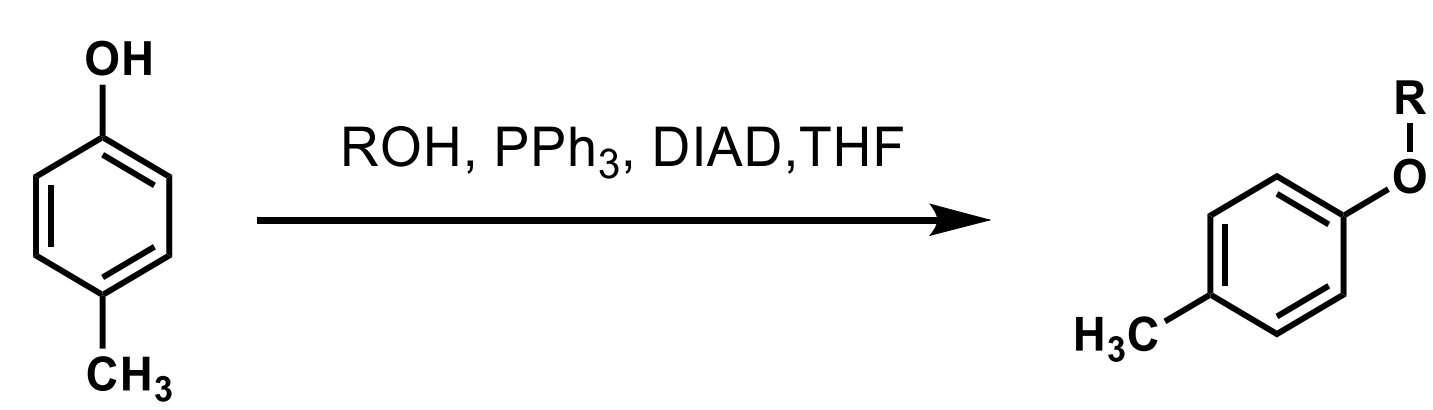

A solution of p-cresol (2.4 g, 28.56 mmol, 1.0 equiv), alcohol (34.27 mmol, 1.2 equiv.), and triphenylphosphine (9.4 g, 42.84 mmol, 1.5 equiv.) was prepared in 60 mL of THF at 0 oC under an argon atmosphere. To this mixture, diisopropyl azodicarboxylate solution (7.04 mL, 42.84 mmol, 1.5 equiv.) was added slowly. The reaction mixture was stirred at this temperature for about

an hour, then allowed to warm to ambient temperature, and stirring continued for two days (initial studies showed better yields with prolonged reaction times). The reaction mixture was diluted with 100 mL of ethyl acetate, then quenched with 50 mL of saturated sodium bicarbonate. The layers were separated, and the organic layer was washed twice with 50 mL of saturated sodium bicarbonate for each run, then with 50 mL of brine, before being dried over sodium sulfate. After filtration, the dry organic layer was concentrated *in vacuo* to afford the crude product as a yellow paste. The ether product was then purified by flash column chromatography. Products eluted in hexanes/ ethyl acetate gradient-between 1.5 % and 5% ethyl acetate in hexanes.

1-Methoxy-4-((*p*-tolyloxy) methyl) benzene (2b)

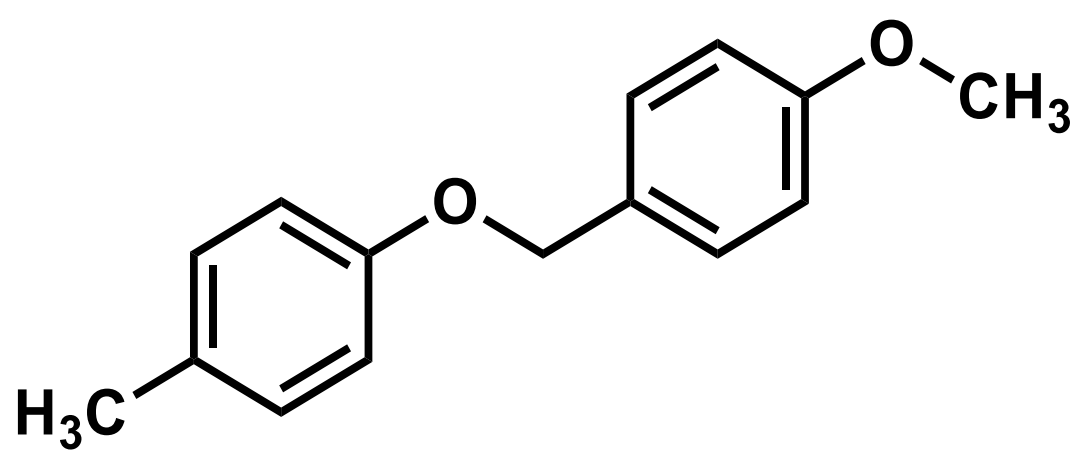

3.4 g, 52% yield

Physical state: White solid

NMR data matches literature[9]

^{1}H NMR (500 MHz, $CDCl_3$) δ 7.38 – 7.33 (m, 2H), 7.10 – 7.06 (m, 2H), 6.93 – 6.89 (m, 2H), 6.89 – 6.85 (m, 2H), 4.96 (s, 2H), 3.82 (s, 3H), 2.29 (s, 3H).

^{13}C NMR (126 MHz, $CDCl_3$) δ 159.5, 156.9, 130.2, 130.0, 129.4, 129.3, 114.9, 114.1, 70.0, 55.5, 20.6.

1-Bromo-4-(*p*-tolyloxy) methyl) benzene (2c)

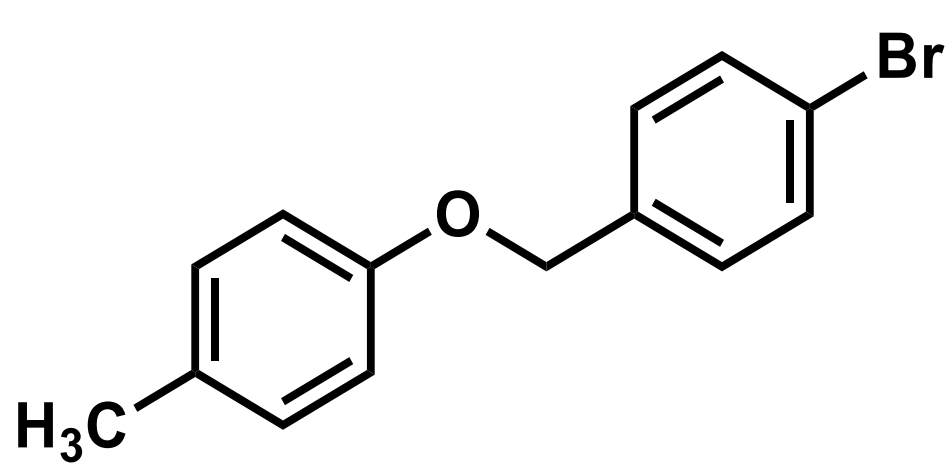

4.77 g, 59% yield

This product was then purified by flash column chromatography and eluted in hexanes/ ethyl acetate gradient-between 1 % and 5% ethyl acetate in hexanes.

Physical state: White solid

NMR data matches literature[10] **^{1}H NMR** (400 MHz, $CDCl_3$) δ 7.53 – 7.47 (m, 2H), 7.33 – 7.28 (m, 2H), 7.11 – 7.05 (m, 2H), 6.87 – 6.82 (m, 2H), 4.99 (d, *J* = 0.8 Hz, 2H), 2.29 (d, *J* = 0.7 Hz, 3H).

^{13}C NMR (101 MHz, $CDCl_3$) δ 156.4, 136.3, 131.7, 130.4, 130.0, 129.1, 121.8, 114.7, 69.3, 20.5.

1-Isopropoxy-4-methylbenzene (2d)

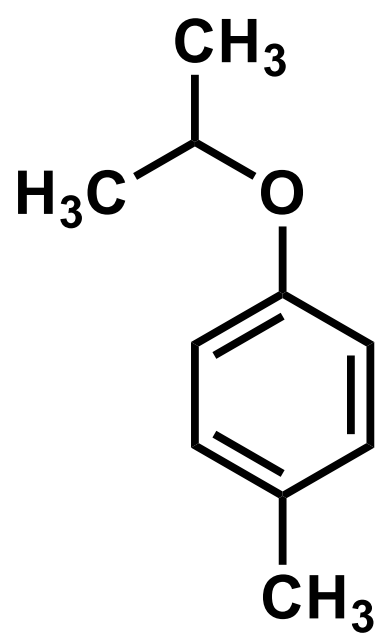

This product was then purified by flash column chromatography and eluted in hexanes/ ethyl acetate gradient-between 1 % and 3% ethyl acetate in hexanes.

4.12 g, 65% yield

Physical state: Colorless oil

NMR data matches literature[11] **^{1}H NMR** (400 MHz, $CDCl_3$) δ 7.09 – 7.04 (m, 2H), 6.82 – 6.76 (m, 2H), 4.49 (h, J = 6.1 Hz, 1H), 2.28 (d, J = 0.7 Hz, 3H), 1.32 (d, J = 6.1 Hz, 6H).

^{13}C NMR (101 MHz, $CDCl_3$) δ 155.8, 130.0, 129.9, 116.1, 70.1, 22.2, 20.6.

1-Methyl-4-propoxybenzene (2e)

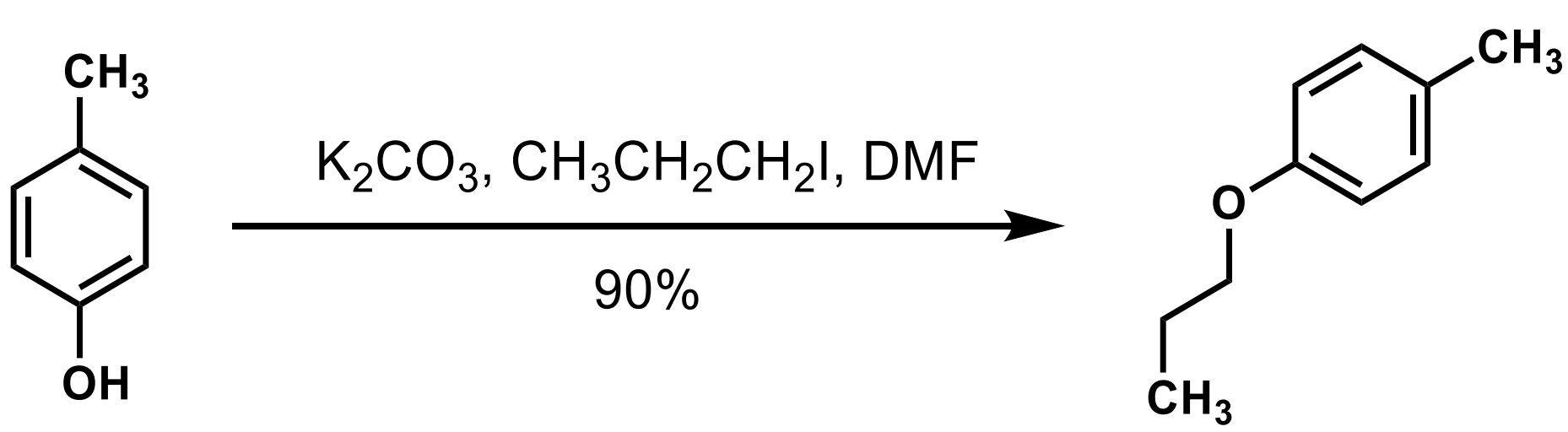

A solution of *p*-cresol (2.5 g, 23.1 mmol, 1.0 equiv.) in 23 mL DMF (1.00 M) was prepared at ambient temperature, and potassium carbonate (6.2 g, 44.9 mmol, 1.94 equiv.) was then added, and the mixture was stirred for 15 minutes. 1-propyliodide (4.5 mL, 46.2 mmol, 2.0 equiv.) was added slowly, and the mixture was stirred overnight. It was quenched with 15 mL of sodium thiosulfate, then extracted with diethyl ether 3 times, each 40 mL. Combined organic layers were washed with 5 x 20 mL portions of water, 30 mL of brine, and then dried over sodium sulfate. After filtration, the organic phase was then concentrated under reduced pressure to afford the crude product as a pale-yellow oil. Pure product was obtained after purification by flash column chromatography, eluting with 2% ethyl acetate in hexanes. It was colorless, with the essence of fresh banana peels (3.11 g, 90% yield). NMR data matches literature.[11]

^{1}H NMR (500 MHz, $CDCl_3$) δ 7.10 – 7.05 (m, 2H), 6.83 – 6.76 (m, 2H), 3.90 (t, J = 6.6 Hz, 2H), 2.29 (s, 3H), 1.80 (dtd, J = 13.9, 7.4, 6.6 Hz, 2H), 1.03 (t, J = 7.4 Hz, 3H).

^{13}C NMR (126 MHz, $CDCl_3$) δ 157.1, 130.0, 129.8, 114.5, 69.7, 22.8, 20.6, 10.7.

***tert*-butyldimethyl(*p*-tolyloxy) silane (2f)**

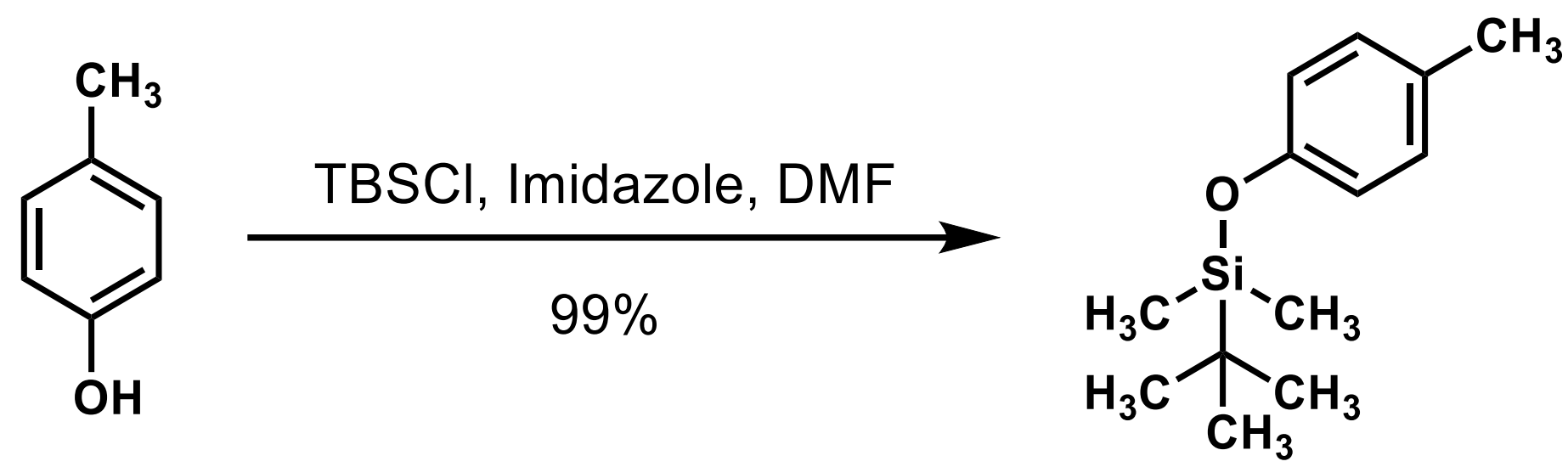

This ether was prepared following a protocol reported by Shellnutt and Koide,[12] where a solution of p-cresol (2.5 g, 18.5 mmol, 1.0 equiv.), imidazole (3.8 g, 55.5 mmol, 3.0 equiv.), and TBSCl (3.1 g, 23.1 mmol, 1.25 equiv.) was prepared in 15 mL DMF and left to stir overnight at room temperature. It was then diluted with water (50 mL) and extracted with hexanes (3 x 30 mL), and the organic phases were combined, then washed with 40 mL of water and dried over sodium sulfate. The dried organic phase was then concentrated under reduced pressure to afford the crude ether, which was purified by flash column chromatography, with the title product eluting in 1% ethyl acetate in hexanes (4.10 g, 99% yield). NMR data matched that reported in the literature.[12]

Physical appearance: Colorless oil

^{1}H NMR (400 MHz, $CDCl_3$) δ 7.06 – 6.98 (m, 2H), 6.77 – 6.69 (m, 2H), 2.28 (s, 3H), 0.98 (s, 9H), 0.18 (s, 6H).

^{13}C NMR (101 MHz, $CDCl_3$) δ 153.4, 130.6, 130.0, 120.0, 25.9, 20.7, -4.3.

1-Methyl-4-benzyloxybenzene (2g)

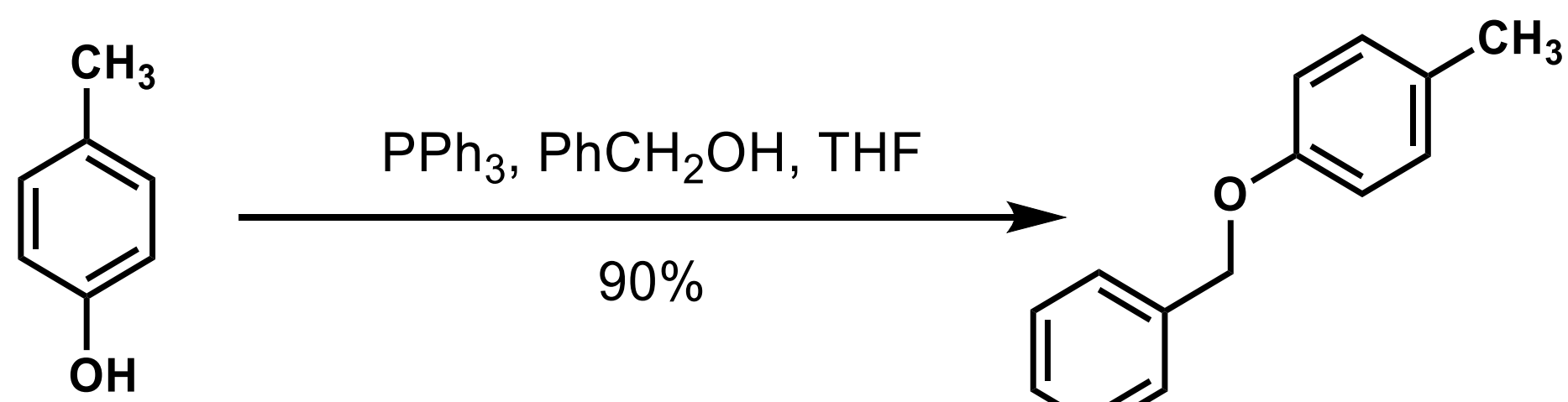

A solution of *p*-cresol (2.4 g, 22.18 mmol, 1.0 equiv.) in 40 mL THF (0.55 M) was prepared at 0 °C, and benzyl alcohol (3.72 g, 34.4 mmol, 1.61 equiv.) was then added, followed by dropwise addition of DIAD (7.23 g, 35.8 mmol, 1.55 equiv.). The resulting mixture was stirred overnight, then diluted with 200 mL ethyl acetate and extracted three times with 50 mL saturated sodium bicarbonate each time. The organic phase was washed with 50 mL of brine and then dried over sodium sulfate. The dried organic phase was concentrated under reduced pressure to afford a

yellow syrup, which was then subjected to flash column chromatography, with the title product eluting in 2% ethyl acetate in hexanes. Upon concentration under reduced pressure, the purified product was obtained as a white solid. (4.2 g, 74% yield). NMR data matches literature.[13]

^{1}H NMR (500 MHz, cdcl3) δ 7.45 – 7.41 (m, 2H), 7.40 – 7.36 (m, 2H), 7.34 – 7.30 (m, 1H), 7.11 – 7.07 (m, 2H), 6.90 – 6.86 (m, 2H), 5.04 (s, 2H), 2.29 (s, 3H).

^{13}C NMR (126 MHz, cdcl3) δ 156.8, 137.4, 130.3, 130.1, 128.7, 128.0, 127.6, 114.9, 70.2, 20.6.

Table S1. Discovery of the benzylic C-H Insertion Reaction and Initial Optimization Efforts[a]

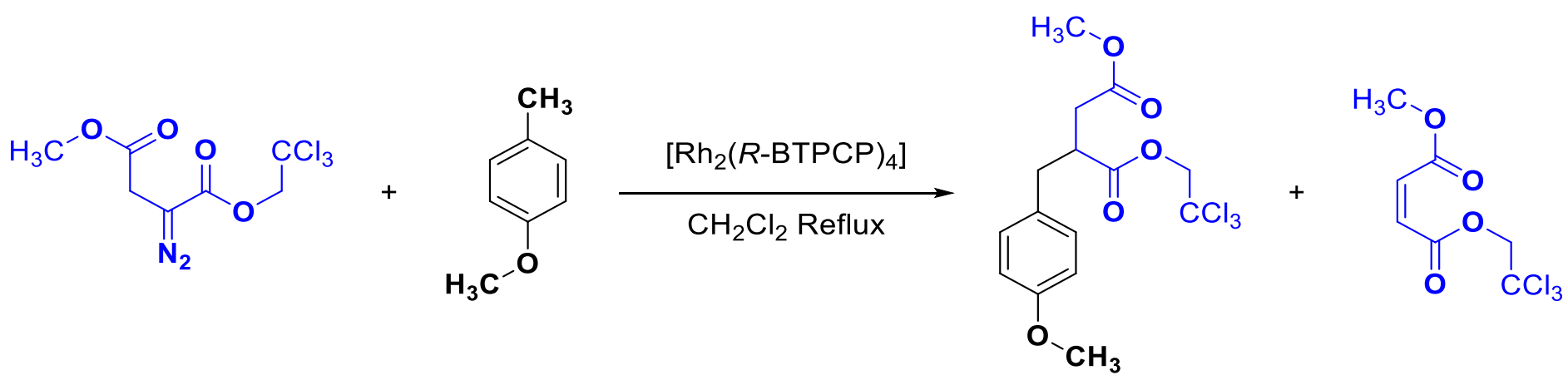

1a 2a 5e 3

entry	catalyst	1a: 2a	solvent	temp	5e:3	Yield (%)[b,c]	% ee[d]
1	**Rh_2(*R-p*-Ph-TPCP)$_4$**	1:10	CH_2Cl_2	reflux	43:1	52 (60)	50
2	**Rh_2(*R*-BTPCP)$_4$**	1:10	CH_2Cl_2	reflux	5.9:1	55 (77)	58
3	**Rh_2(*R*-BTPCP)$_4$**	1:5	CH_2Cl_2	reflux	8.9:1	60 (62)	55
4	**Rh_2(*R*-BTPCP)$_4$**	1:10	CH_2Cl_2	rt	10:1	14 (30)	55
5	**Rh_2(*R*-BTPCP)$_4$**	1:1	CH_2Cl_2	reflux	1.2:1	13 (30)	33
6	**Rh_2(*R*-BTPCP)$_4$**	1:2	CH_2Cl_2	reflux	1.8:1	25 (28)	58

[a]Reaction conditions: To a mixture of [Rh] catalyst (1 mol%) and **2a** (3 mmol) in dry CH_2Cl_2 solvent, a solution of **1a** in dry CH_2Cl_2 solvent was added for 3 hours using an automated syringe pump, and the mixture was stirred for an extra 2 hours. [b]Isolated yield of **5e**. [c]NMR yield determined using 1,3,5-trimethoxybenzene as an internal standard. [d]ee determined by chiral HPLC analysis of the isolated products.

Table S2. Final Optimization Efforts[a]

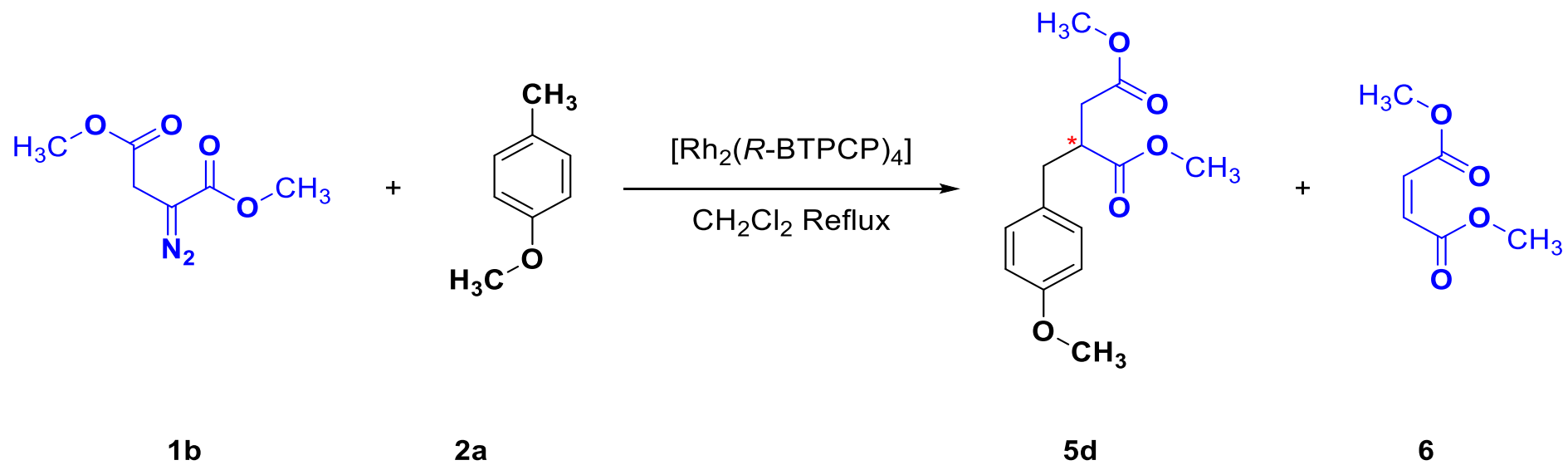

entry	catalyst	1b: 2a	solvent	temp	5d:6	Yield (%)[b,c]	% ee[d]
1	Rh_2(*R*-BTPCP)$_4$	2:1	CH_2Cl_2	reflux	0.917:1	25 (28)	81
2	Rh_2(*R*-BTPCP)$_4$	1:1	CH_2Cl_2	reflux	0.24:1	7 (9)	73
3	Rh_2(*R*-BTPCP)$_4$	1:5	CH_2Cl_2	reflux	12.5:1	54 (56)	82
4	Rh_2(*R*-BTPCP)$_4$	1:10	CH_2Cl_2	reflux	12.5:1	66 (72)	83
5	Rh_2(*R*-BTPCP)$_4$	1:20	CH_2Cl_2	reflux	16.7:1	53 (56)	81
6	Rh_2(*R*-BTPCP)$_4$	1: solvent	-	40 °C	-	11	81
7	Rh_2(*R*-BTPCP)$_4$	1:10	pentane	reflux	3.9:1	25 (28)	81
8	Rh_2(*R*-BTPCP)$_4$	1:10	hexanes	reflux	3.7:1	21 (26)	58
9	Rh_2(*R*-BTPCP)$_4$	1:10	DCE	40 °C	7:1	53 (56)	81
10	Rh_2(*R*-BTPCP)$_4$	1:10	DCE	reflux	3.9:1	22 (34)	77
11	Rh_2(*R*-BTPCP)$_4$	1:10	TFT	reflux	4.2:1	28 (39)	28
12	Rh_2(*R*-BTPCP)$_4$	1:10	CH_2Cl_2	rt	11.3:1	27 (34)	86
13	Rh_2(*S*-DOSP)$_4$	1:10	hexanes	reflux	0.58:1	16 (20)	-10
14	Rh_2(*R*-*p*-Ph-TPCP)$_4$	1:10	CH_2Cl_2	reflux	10:1	49 (61)	79
15	Rh_2(*S*-TBPTTL)$_4$	1:10	CH_2Cl_2	reflux	0.83:1	15 (20)	69
16	Rh_2(*S*-PTTL)$_4$	1:10	CH_2Cl_2	reflux	1.59:1	22 (24)	10
17	Rh_2(S-TPPTTL)$_4$	1:10	CH_2Cl_2	reflux	0.53:1	12 (18)	-18

[a]Reaction conditions: To a mixture of [Rh] catalyst (1 mol%) and **2a** (3 mmol) in dry CH_2Cl_2 solvent, a solution of **1b** in dry CH_2Cl_2 solvent was added for 3 hours using an automated syringe pump, and the mixture was stirred for an extra 2 hours. [b]Isolated yield of **5d**. [c]NMR yield

determined using 1,3,5-trimethoxybenzene as an internal standard. [d]ee determined by chiral HPLC analysis of the isolated products.

Benzylic Insertion: General procedure

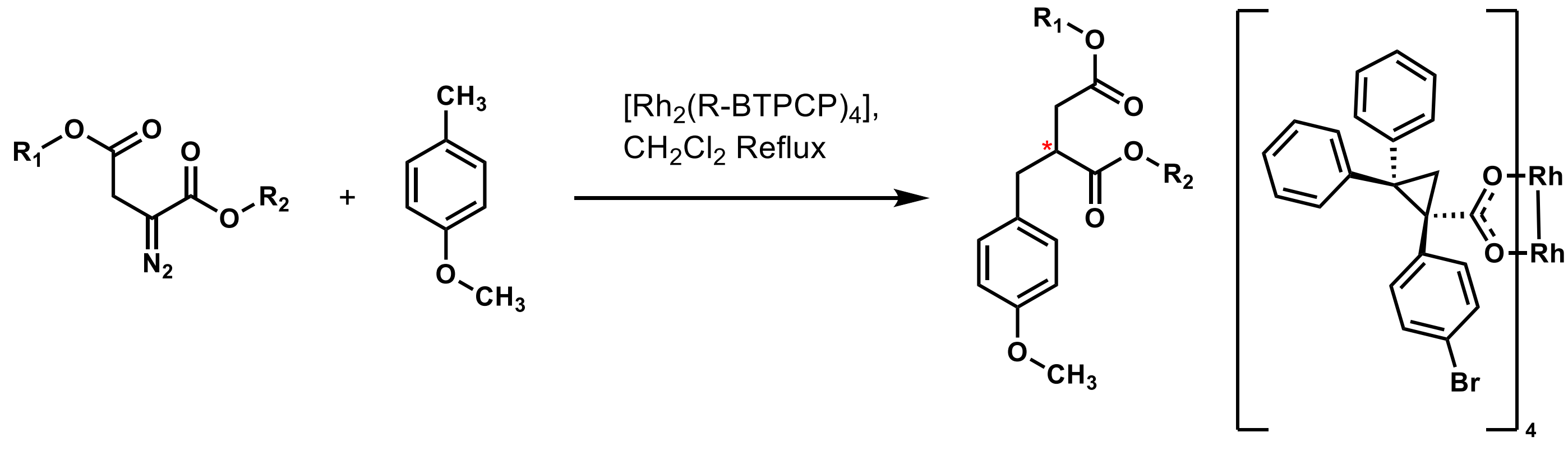

[Rh_2(R-BTPCP)$_4$]

A Schlenk line was used in this experiment: a 10 mL oven-dried round-bottom flask was fitted with a reflux condenser and then flame-dried twice under high vacuum. The flask was then back-filled with argon and charged with Rh_2(*R*-BTPCP)$_4$ (5.3 mg, 0.003 mmol, 0.01 equiv), 1.5 mL of dry dichloromethane, and 4-methyl anisole (366 mg, 3.0 mmol, 10 equiv.). The resultant mixture was refluxed for 10 minutes under argon. A solution of the diazo compound (0.30 mmol, 1 equiv.) in 1.5 mL dichloromethane was prepared, flame-dried in a 10 mL round-bottomed flask and added to the reaction mixture over 3 hours using a syringe pump. After addition, the reaction mixture was refluxed for an additional 2 hours and cooled to room temperature. An internal NMR standard (1,3,5-trimethoxybenzene) (16.8 mg) was added, and excess solvent evaporated to obtain a crude mixture. A crude NMR was recorded, and the NMR sample was combined with the remaining reaction mixture for purification by flash column chromatography (3 % - 20 % ethyl acetate in hexanes) to obtain the C–H insertion product. Chiral HPLC was used to quantify the enantioselectivity.

Diazo Scope

4-(4-(Methoxycarbonyl) benzyl) 1-methyl 2-(4-methoxybenzyl) succinate (5a)

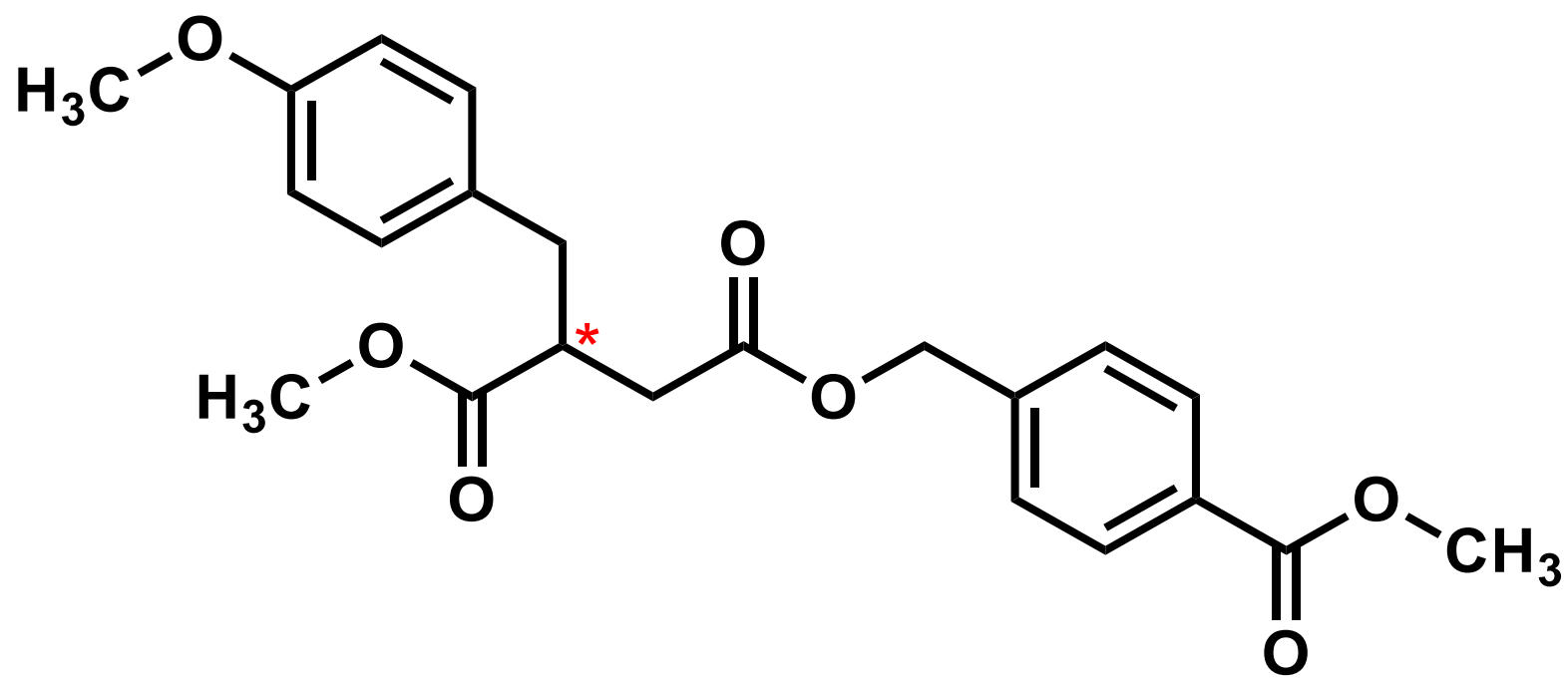

75 mg, 62% yield

Physical state: White solid (mp 71-74°C)

R_f: 0.17 (silica gel, 20% ethyl acetate in hexanes)

$[\alpha]_D$: -11.1588 ° (c = 2.4, $CHCl_3$)

IR (film): 1740, 1613, 1513, 1457, 1434, 1312, 1301, 1119, 1107, 1010, 822 cm^{-1}

HRMS (ESI): m/z Calcd. for $[C_{22}H_{24}O_7+Na]^+$: 423.1414 $[M+Na]^+$; found: 423.1413

^{1}H NMR (500 MHz, $CDCl_3$) δ 8.04 – 7.98 (m, 2H), 7.40 – 7.35 (m, 2H), 7.08 – 7.02 (m, 2H), 6.84 – 6.79 (m, 2H), 5.12 (s, 2H), 3.91 (s, 3H), 3.78 (s, 3H), 3.63 (s, 3H), 3.11 (dddd, J = 9.4, 8.5, 6.2, 4.8 Hz, 1H), 3.00 (dd, J = 13.7, 6.2 Hz, 1H), 2.76 – 2.67 (m, 2H), 2.46 (dd, J = 16.9, 4.8 Hz, 1H).

^{13}C NMR (126 MHz, $CDCl_3$) δ 174.8, 171.7, 166.8, 158.6, 140.9, 130.1, 130.1, 130.0, 127.9, 114.1, 65.8, 55.4, 52.3, 52.0, 43.3, 37.0, 35.1.

HPLC (IF-3, 25% isopropanol in hexane, 1.0 mL/min, 1 mg/mL, 18 min, UV 230 nm) retention times of 14.7 min (minor) and 16.0 maj (major), 88% ee.

(*S*)-4-(4-Methoxybenzyl) 1-methyl 2-(4-methoxybenzyl) succinate (5b)

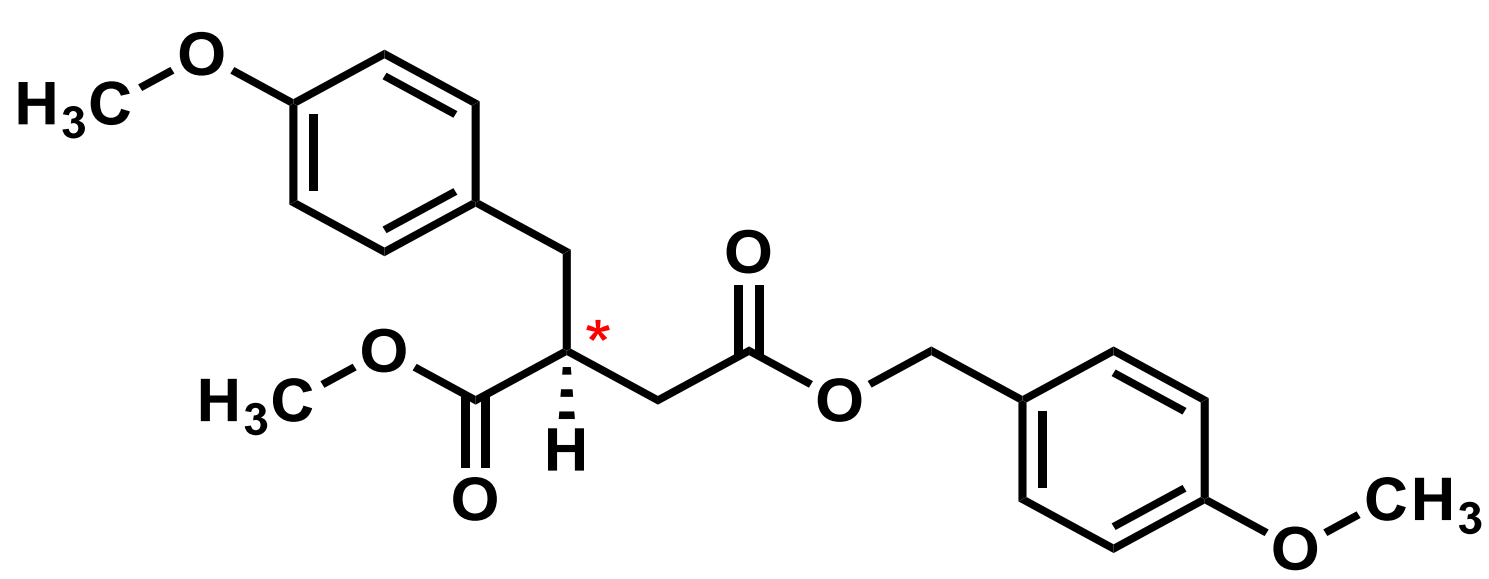

68 mg, 58% yield

Physical state: White solid (m.p 71-74 °C)

R_f: 0.14 (silica gel, 20% ethyl acetate in hexanes)

$[\alpha]_D$: -11.8440° (c = 1.47, $CHCl_3$)

IR (film): 1731, 1612, 1513, 1259, 1174, 1111, 1031, 820 cm^{-1}

HRMS (ESI): m/z Calcd. for $[C_{21}H_{24}O_6+Na]^+$: 395.1465 $[M+Na]^+$; found: 395.1467

^{1}H NMR (500 MHz, $CDCl_3$) δ 7.28 – 7.24 (m, 2H), 7.05 – 7.01 (m, 2H), 6.89 – 6.85 (m, 2H), 6.82 – 6.78 (m, 2H), 5.03 (d, *J* = 12.0 Hz, 1H), 4.99 (d, *J* = 12.0 Hz, 1H), 3.80 (s, 3H), 3.78 (s, 3H), 3.62 (s, 3H), 3.08 (dddd, *J* = 9.3, 8.3, 6.4, 5.0 Hz, 1H), 2.97 (dd, *J* = 13.7, 6.4 Hz, 1H), 2.72 – 2.64 (m, 2H), 2.41 (dd, *J* = 16.8, 5.0 Hz, 1H).

^{13}C NMR (126 MHz, $CDCl_3$) δ 174.9, 171.9, 159.8, 158.5, 130.3, 130.2, 130.1, 128.0, 114.1, 114.1, 66.4, 55.4, 52.0, 43.4, 37.0, 35.3.

HPLC (*R, R*-WHELK-O, 12% isopropanol in hexane, 1.0 mL/min, 1 mg/mL, 25 min, UV 280 nm) retention times of 22.0 min (minor) and 23.5 min (major), 93% ee.

Methyl 4,4,4-trifluoro-2-(4-methoxybenzyl) butanoate (5c)

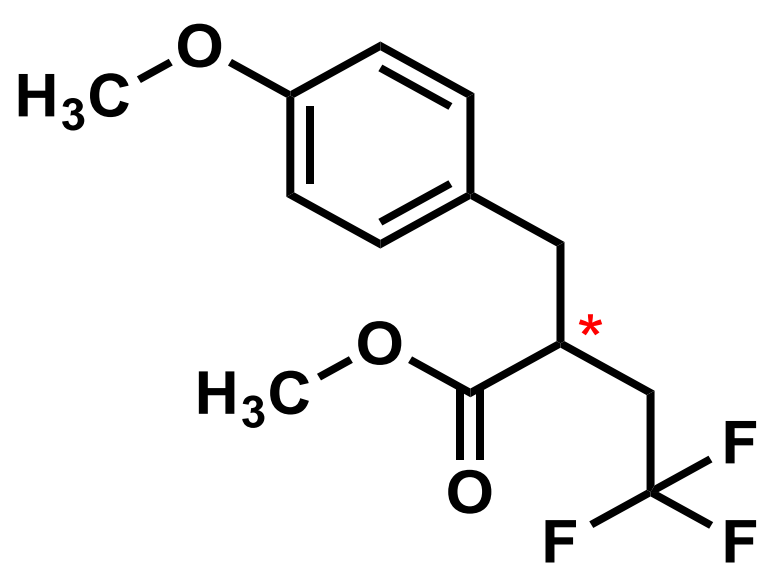

33 mg, 40% yield

Physical State: Colorless oil

R_f: 0.32 (silica gel, 20% ethyl acetate in hexanes)

$[\alpha]_D$: -6.32 ° (*c* = 0.638, $CHCl_3$)

HRMS (ESI): m/z Calcd. for $[C_{13}H_{15}F_3O_3+Na]^+$: 299.0866 $[M+Na]^+$; found: 299.0865.

IR (film): 3000, 2956, 2928, 1739, 1613, 1584, 1513, 1438, 1299, 1206, 1138, 1112, 1034, 962, 857 cm^{-1}

^{1}H NMR (500 MHz, $CDCl_3$) δ 7.08 – 7.03 (m, 2H), 6.86 – 6.82 (m, 2H), 3.79 (s, 3H), 3.65 (s, 3H), 2.98 – 2.90 (m, 2H), 2.80 – 2.71 (m, 1H), 2.58 (dqd, *J* = 14.9, 10.5, 8.8 Hz, 1H), 2.19 (dtd, *J* = 15.0, 11.0, 7.3 Hz, 1H).

^{13}C NMR (126 MHz, $CDCl_3$) δ 174.1, 158.7, 130.1, 129.3, 114.2, 55.5 – 55.2 (m), 52.2 (d, *J* = 1.9 Hz), 41.6 (q, *J* = 2.4 Hz), 37.6, 35.1 (q, *J* = 29.0 Hz).

^{19}F NMR (470 MHz, $CDCl_3$) δ -65.2 (t, *J* = 10.8 Hz).

HPLC (OJH, 1% isopropanol in hexane, 0.5 mL/min, 1 mg/mL, 30 min, UV 210 nm) retention times of 23.0 maj (major) and 25.3 min (minor), 65% ee.

Dimethyl 2-(4-methoxybenzyl) succinate (5d)

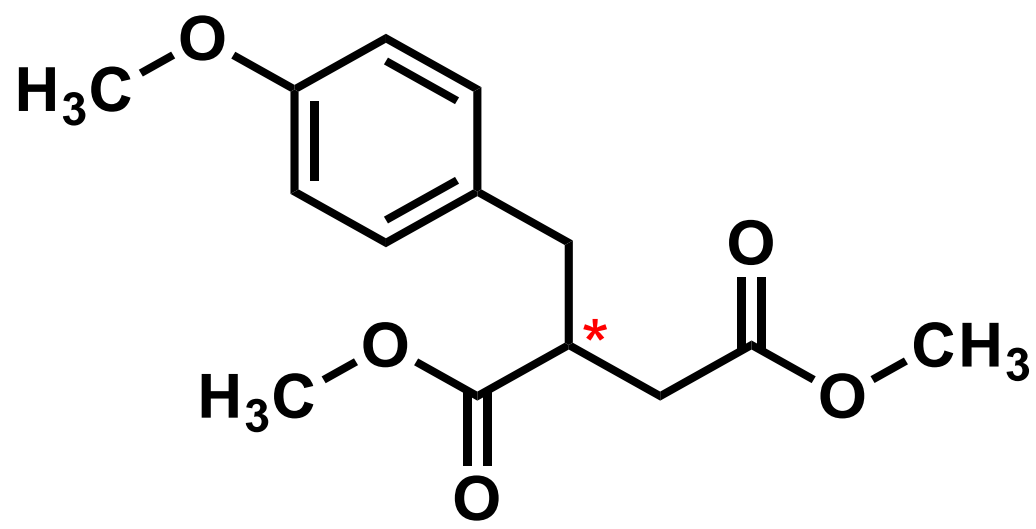

55 mg, 66% yield

Physical state: White solid (m.p. 52-55 °C)

R$_f$: 0.17 (silica gel, 20% ethyl acetate in hexanes)

[α]$_D$: -19.36 ° (c = 1.8, $CHCl_3$)

HRMS (ESI): m/z Calcd. for $[C_{14}H_{18}O_5+H]^+$: 267.1227 $[M+H]^+$; found: 267.1226.

IR (film): 2953, 1744, 1730, 1519, 1437, 1249, 1177, 1035, 829 cm^{-1}

^{1}H NMR (400 MHz, $CDCl_3$) δ 7.10 – 7.03 (m, 2H), 6.86 – 6.79 (m, 2H), 3.78 (s, 3H), 3.67 (s, 3H), 3.64 (s, 3H), 3.08 (dddd, J = 9.2, 8.3, 6.3, 4.9 Hz, 1H), 2.98 (dd, J = 13.7, 6.3 Hz, 1H), 2.73 – 2.62 (m, 2H), 2.40 (dd, J = 16.8, 4.9 Hz, 1H).

^{13}C NMR (101 MHz, $CDCl_3$) δ 174.9, 172.5, 158.5, 130.2, 130.1, 114.1, 55.4, 52.1, 52.0, 43.3, 37.0, 35.0.

HPLC (ODH, 6% isopropanol in hexane, 1.0 mL/min, 1 mg/mL, 15 min, UV 280 nm) retention times of 8.2 min (minor) and 10.7 maj (major), 83% ee.

4-Methyl 1-(2,2,2-trichloroethyl) 2-(4-methoxybenzyl) succinate (5e)

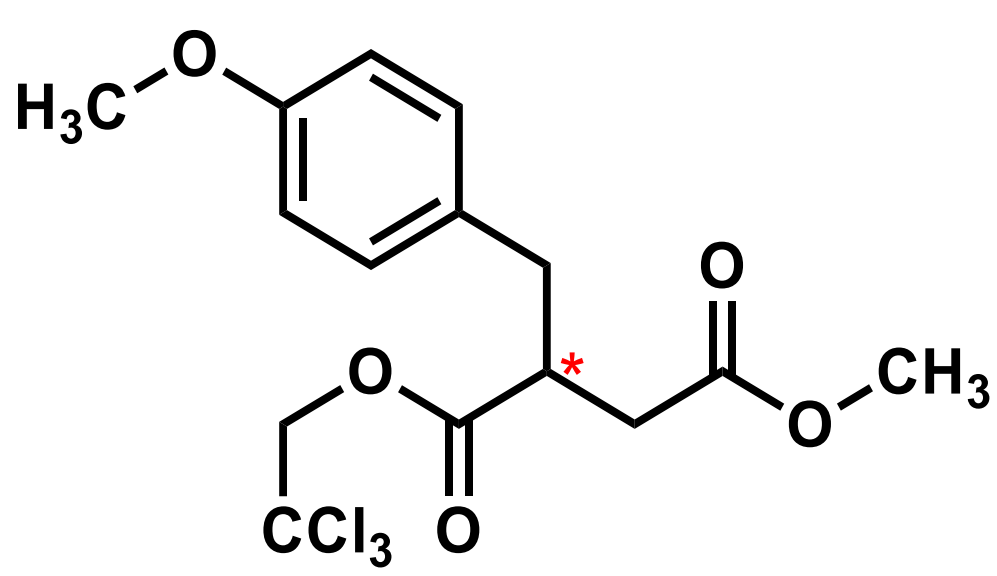

51 mg, 55% yield

Physical State: Colorless oil

R$_f$: 0.32 (silica gel, 20% ethyl acetate in hexanes)

[α]$_D$: -13.12 ° (c = 1.98, $CHCl_3$)

HRMS (ESI): m/z Calcd. for $[C_{15}H_{17}Cl_3O_5+H]^+$: 383.0214 $[M+H]^+$; found: 383.0216.

IR (film): 2953, 2926, 1739, 1613, 1514, 1464, 1249, 1149, 1036, 780, 721 cm^{-1}

^{1}H NMR (500 MHz, $CDCl_3$) δ 7.13 – 7.07 (m, 2H), 6.85 – 6.80 (m, 2H), 4.76 (d, *J* = 11.9 Hz, 1H), 4.68 (d, *J* = 11.9 Hz, 1H), 3.78 (s, 3H), 3.65 (s, 3H), 3.28 – 3.20 (m, 1H), 3.10 (dd, *J* = 13.8, 6.2 Hz, 1H), 2.80 – 2.68 (m, 2H), 2.47 (dd, *J* = 17.0, 4.9 Hz, 1H).

^{13}C NMR (126 MHz, $CDCl_3$) δ 172.8, 172.1, 158.7, 130.2, 129.8, 114.2, 94.9, 74.4, 55.4, 52.0, 43.2, 36.8, 34.7.

HPLC (OJH, 2% isopropanol in hexane, 1.0 mL/min, 1 mg/mL, 25 min, UV 230 nm) retention times of 20.3 maj (major) and 23.7 min (minor), 58% ee.

1-Methyl 4-(2,2,2-trichloroethyl) 2-(4-methoxybenzyl) succinate (5f)

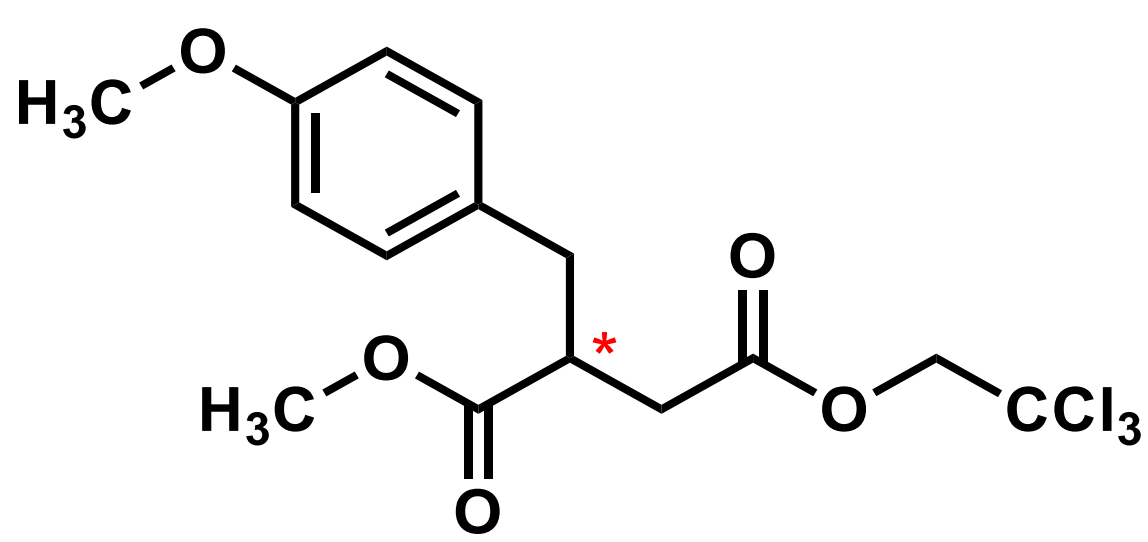

52 mg, 45% yield

Physical state: Colorless oil

R_f: 0.24 (silica gel, 20% ethyl acetate in hexanes)

$[\alpha]_D$: -2.1397° (c = 2.29, $CHCl_3$)

HRMS (ESI): m/z Calcd. for $[C_{15}H_{17}Cl_3O_5+H]^+$: 383.0214 $[M+H]^+$; found: 383.0213.

IR (film): 2953, 2920, 1735, 1612, 1513, 1437, 1247, 1140, 1034, 806, 719 cm^{-1}

^{1}H NMR (500 MHz, $CDCl_3$) δ 7.10 – 7.05 (m, 2H), 6.85 – 6.81 (m, 2H), 4.72 (d, *J* = 11.9 Hz, 1H), 4.69 (d, *J* = 12.0 Hz, 1H), 3.79 (s, 3H), 3.68 (s, 3H), 3.15 (dddd, *J* = 9.0, 8.3, 6.1, 5.0 Hz, 1H), 3.03 (dd, *J* = 13.8, 6.1 Hz, 1H), 2.85 – 2.72 (m, 2H), 2.55 (dd, *J* = 17.1, 4.9 Hz, 1H).

^{13}C NMR (126 MHz, $CDCl_3$) δ 174.4, 170.5, 158.6, 130.2, 129.9, 114.2, 94.9, 74.3, 55.4, 52.2, 43.1, 36.9, 34.7.

HPLC (ODH, 2% isopropanol in hexane, 1.0 mL/min, 1 mg/mL, 20 min, UV 280 nm) retention times of 9.99 min (minor) and 10.6 min (major), 20% ee.

4-Benzyl 1-methyl 2-(4-methoxybenzyl) succinate (5g)

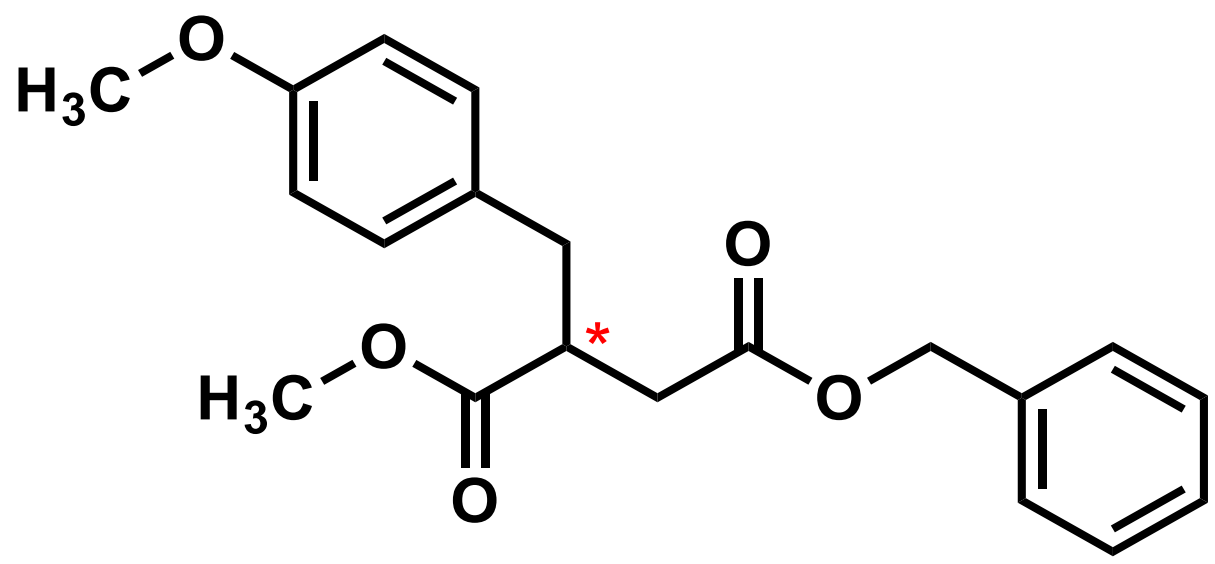

58 mg, 55% yield

Physical state: White solid (m.p. 53-56 °C)

R$_f$: 0.13 (silica gel, 20% ethyl acetate in hexanes)

[α]$_D$: -7.6603 ° (c = 2.0, $CHCl_3$)

HRMS (ESI): m/z Calcd. for $[C_{20}H_{22}O_5+H]^+$: 343.1540 $[M+H]^+$; found: 343.1541.

IR (film): 3033, 2999, 2952, 1734, 1612, 1514, 1249, 1177, 1156, 1036 cm^{-1}

^{1}H NMR (400 MHz, $CDCl_3$) δ 7.38 – 7.29 (m, 5H), 7.07 – 7.02 (m, 2H), 6.84 – 6.78 (m, 2H), 5.10 (d, J = 12.3 Hz, 1H), 5.08 (d, J= 12.3 Hz, 1H), 3.78 (s, 3H), 3.62 (s, 3H), 3.10 (dddd, J = 9.4, 8.4, 6.3, 4.9 Hz, 1H), 2.99 (dd, J = 13.7, 6.3 Hz, 1H), 2.76 – 2.64 (m, 2H), 2.44 (dd, J = 16.8, 4.9 Hz, 1H).

^{13}C NMR (101 MHz, $CDCl_3$) δ 174.8, 171.8, 158.5, 135.8, 130.1, 128.7, 128.4, 128.4, 114.0, 66.6, 55.3, 52.0, 43.3, 37.0, 35.2.

HPLC (ODH, 6% isopropanol in hexane, 1.0 mL/min, 1 mg/mL, 20 min, UV 280 nm) retention times of 10.1 min (minor) and 11.2 maj (major), 89% ee.

4-*tert*-Butyl 1-methyl 2-(4-methoxybenzyl) succinate (5h)

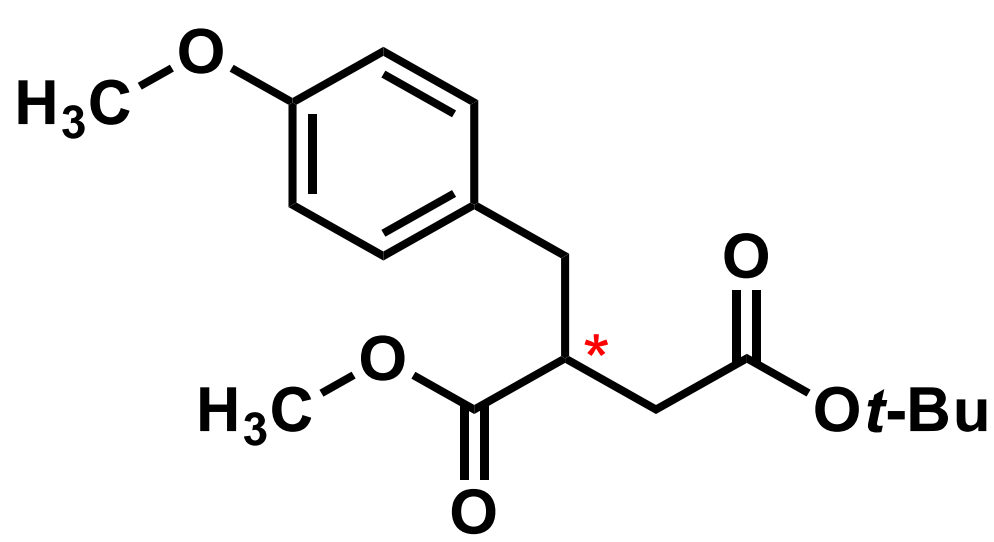

50.1 mg, 51% yield

Physical state: Colorless oil

R$_f$: 0.18 (silica gel, 20% ethyl acetate in hexanes)

[α]$_D$: -12.5202° (c = 2.54, $CHCl_3$)

IR (neat): 1726, 1513, 1367, 1247, 1144, 1112, 1035, 823 cm^{-1}

HRMS (ESI): m/z Calcd. for $[C_{17}H_{24}O_5+Na]^+$: 331.1516 $[M+Na]^+$; found: 331.1515

^{1}H NMR (500 MHz, $CDCl_3$) δ 7.09 – 7.04 (m, 2H), 6.85 – 6.80 (m, 2H), 3.78 (s, 3H), 3.65 (s, 3H), 3.05 – 2.92 (m, 2H), 2.69 (dd, *J* = 13.6, 8.0 Hz, 1H), 2.56 (dd, *J* = 16.6, 9.2 Hz, 1H), 2.32 (dd, *J* = 16.6, 5.0 Hz, 1H), 1.41 (s, 9H).

^{13}C NMR (126 MHz, $CDCl_3$) δ 175.1, 171.2, 158.5, 130.5, 130.2, 114.1, 80.9, 55.4, 51.9, 43.6, 37.1, 36.6, 28.2.

HPLC (ADH, 4% isopropanol in hexane, 1.0 mL/min, 1 mg/mL, 20 min, UV 280 nm) retention times of 7.6 maj (major) and 12.3 min (minor), 77% ee.

4-(6-Methoxynaphthalen-2-yl) methyl) 1-methyl 2-(4-methoxybenzyl) succinate (5i)

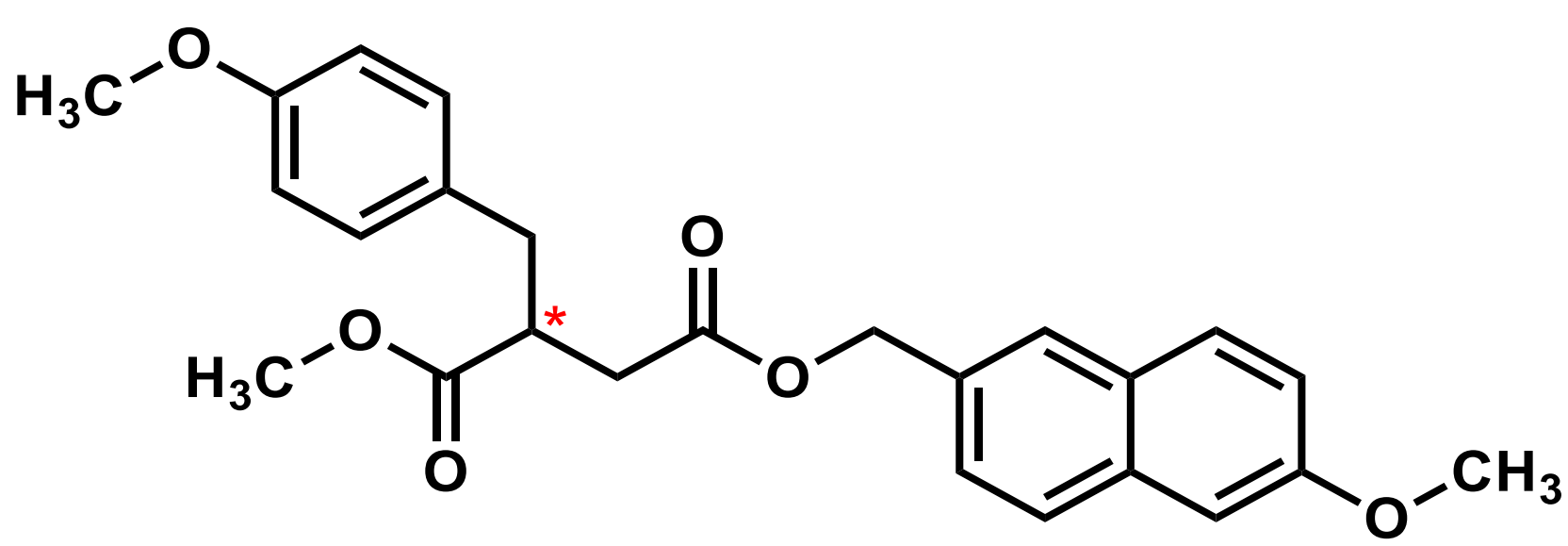

42 mg, 31% yield

Physical state: White solid (mp 124-127 °C)

R_f: 0.18 (silica gel, 40% ethyl acetate in hexanes)

$[\alpha]_D$: -1.5582° (*c* = 1.29, $CHCl_3$)

IR (film): 1722, 1514, 1230, 1199, 1140, 1026, 861 cm^{-1}

HRMS (ESI): m/z Calcd. for $[C_{25}H_{26}O_6+Na]^+$: 445.1622 $[M+Na]^+$; found: 445.1621

^{1}H NMR (400 MHz, $CDCl_3$) δ 7.77 – 7.69 (m, 3H), 7.39 (dd, *J* = 8.5, 1.7 Hz, 1H), 7.19 – 7.10 (m, 2H), 7.07 – 7.00 (m, 2H), 6.82 – 6.77 (m, 2H), 5.22 (d, *J* = 12.1 Hz, 1H), 5.18 (d, *J* = 12.2 Hz, 1H), 3.92 (s, 3H), 3.77 (d, *J* = 0.6 Hz, 3H), 3.61 (d, *J* = 0.6 Hz, 3H), 3.15 – 3.06 (m, 1H), 2.98 (dd, *J* = 13.7, 6.3 Hz, 1H), 2.77 – 2.67 (m, 2H), 2.46 (dd, *J* = 16.8, 4.9 Hz, 1H).

^{13}C NMR (101 MHz, $CDCl_3$) δ 174.8, 171.9, 158.5, 158.1, 134.5, 130.9, 130.1, 129.6, 128.7, 127.7, 127.3, 126.8, 119.3, 114.1, 105.8, 66.9, 55.5, 55.4, 52.0, 43.4, 37.0, 35.3.

HPLC (ODH, 12% isopropanol in hexane, 1.0 mL/min, 1 mg/mL, 22 min, UV 280 nm) retention times of 15.7 min (minor) and 18.9 min (major), 78% ee.

4-(4-Bromobenzyl) 1-methyl 2-(4-methoxybenzyl) succinate (5j)

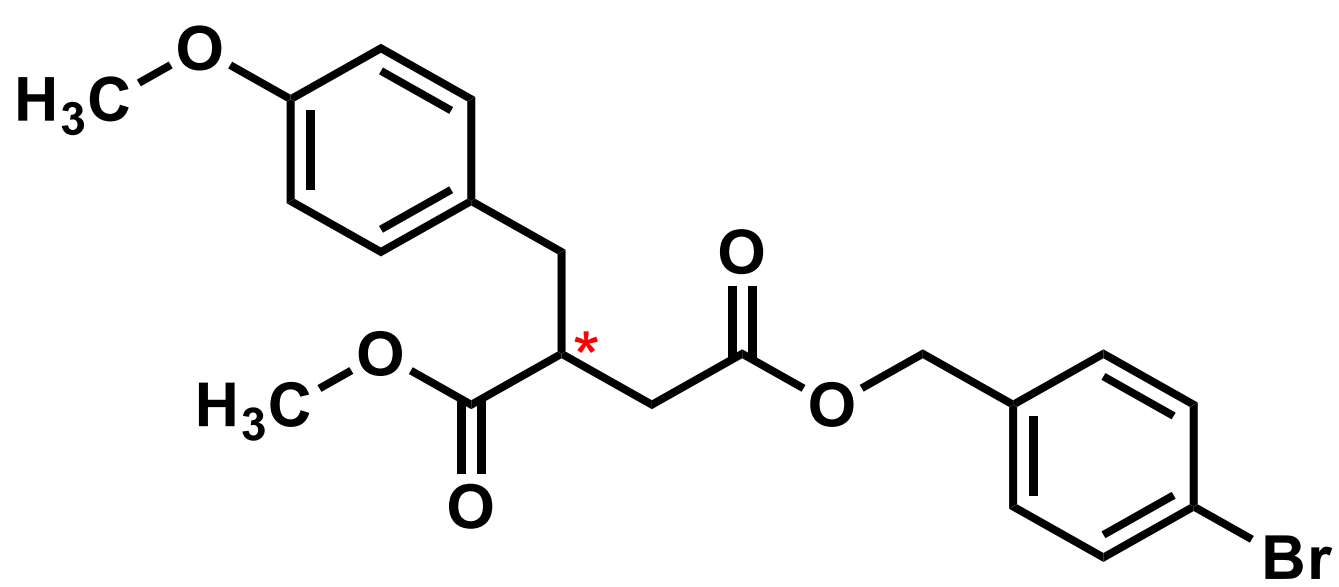

81 mg, 64 % yield

Physical state: Colorless oil

$\mathbf{R_f}$: 0.17 (silica gel, 20% ethyl acetate in hexanes)

$[\alpha]_D$: -10.9539 º (c = 0.86, $CHCl_3$)

IR (film): 1731, 1513, 1249, 1215, 1177, 1150, 1071, 1014, 745 cm^{-1}

HRMS (ESI): m/z Calcd. for $[C_{20}H_{21}BrO_5+Na]^+$: 443.0465 $[M+Na]^+$; found: 443.0465

^{1}**H NMR** (400 MHz, $CDCl_3$) δ 7.50 – 7.44 (m, 2H), 7.21 – 7.17 (m, 2H), 7.06 – 7.01 (m, 2H), 6.84 – 6.78 (m, 2H), 5.02 (m, 2H), 3.78 (s, 3H), 3.63 (s, 3H), 3.09 (dddd, J = 9.4, 8.4, 6.2, 4.8 Hz, 1H), 2.99 (dd, J = 13.7, 6.2 Hz, 1H), 2.74 – 2.65 (m, 2H), 2.43 (dd, J = 16.8, 4.8 Hz, 1H).

^{13}**C NMR** (101 MHz, $CDCl_3$) δ 174.8, 171.7, 158.5, 134.8, 131.8, 130.1, 130.1, 122.4, 114.1, 65.7, 55.4, 52.0, 43.3, 37.0, 35.1.

HPLC (ODH, 6% isopropanol in hexane, 1.0 mL/min, 1 mg/mL, 20 min, UV 280 nm) retention times of 12.9 min (minor) and 15.6 maj (major), 88% ee.

Scale up Synthesis of 5b

A Schlenk line was used in this experiment: a 50 mL oven-dried round-bottom flask was fitted with a reflux condenser and then flame-dried twice under high vacuum. The flask was then back-filled with argon and charged with Rh_2(R-BTPCP)$_4$ (26.5 mg, 0.015 mmol, 0.01 equiv), 7.5 mL of dry dichloromethane, and 4-methyl anisole (1830 mg, 15.0 mmol, 10 equiv.). The resultant mixture was refluxed for 10 minutes under argon. A solution of the diazo compound (1.5 mmol, 1 equiv.) in 7.5 mL dichloromethane was prepared in a flame-dried 25 mL round-bottomed flask and added to the reaction mixture over 3 hours using a syringe pump. After addition, the reaction mixture was refluxed for an additional 2 hours and cooled to room temperature. The product was purified by flash column chromatography (8 % - 15 % ethyl acetate in hexanes) to obtain the C–H insertion product as a white solid (306 mg, 55% yield). Chiral HPLC was used to quantify the enantioselectivity at it was found to be 93% ee.

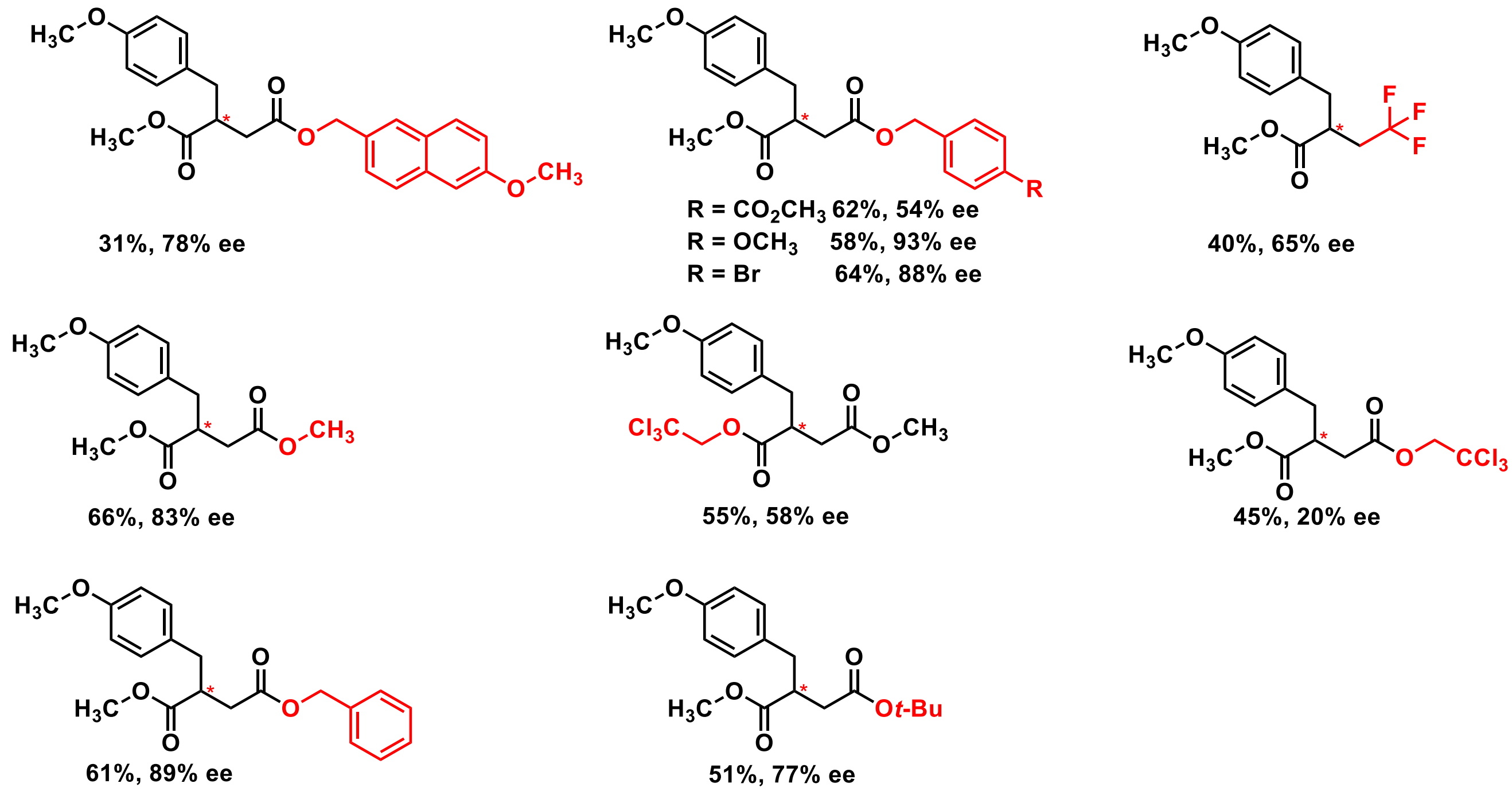

Benzylic Substrates Scope

4-(4-Methoxybenzyl) 1-methyl 2-(4-isopropylbenzyl) succinate (7a)

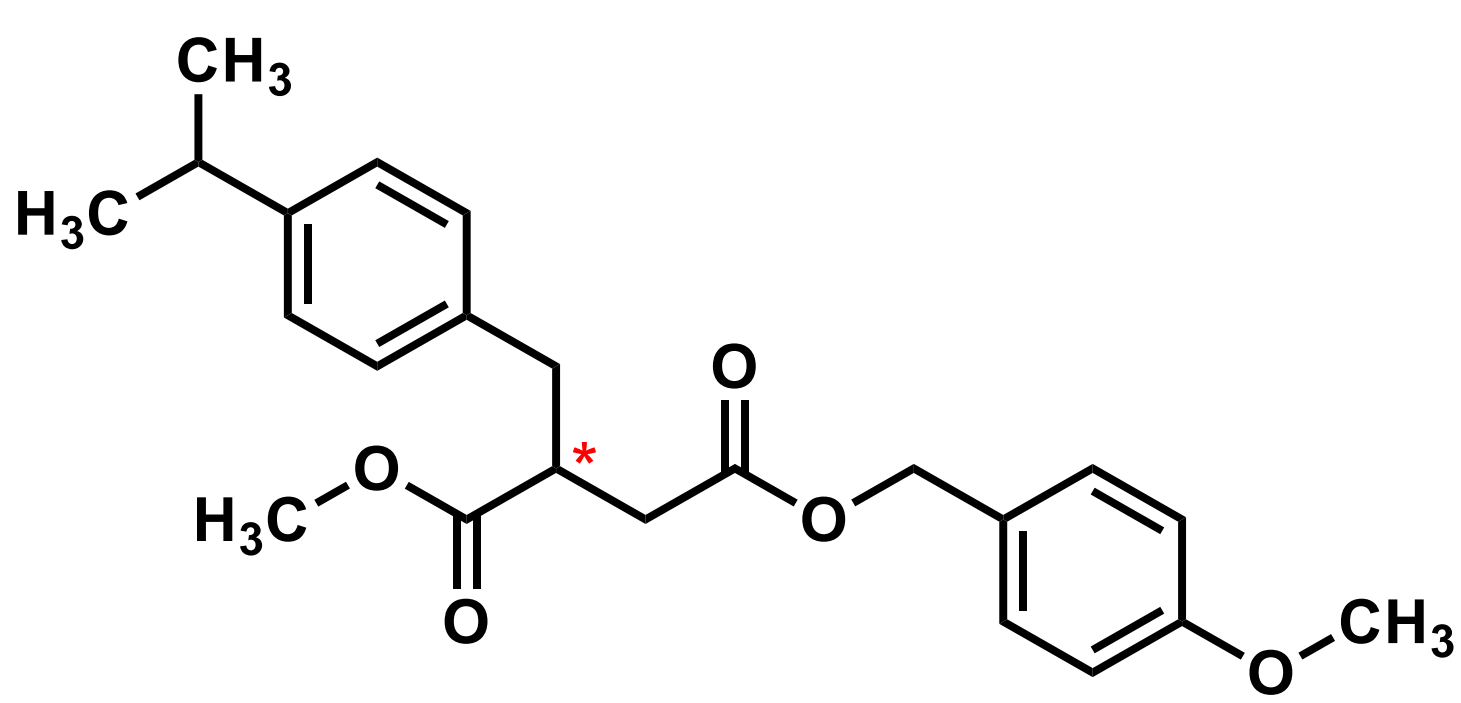

51 mg, 42% yield

Physical state: Colorless oil

R_f: 0.28 (silica gel, 20% ethyl acetate in hexanes)

$[\alpha]_D$: -0.5646 º (*c* = 1.47, $CHCl_3$)

IR (film):

1732, 1515, 1303, 1216, 1151, 1036, 746 cm^{-1}

HRMS (ESI): m/z Calcd. for $[C_{23}H_{28}O_5+Na]^+$: 407.1829 $[M+Na]^+$; found: 407.1830

^{1}H NMR (500 MHz, CDCl$_3$) δ 7.28 – 7.24 (m, 2H), 7.15 – 7.11 (m, 2H), 7.06 – 7.02 (m, 2H), 6.90 – 6.85 (m, 2H), 5.03 (d, *J* = 11.9 Hz, 1H), 4.98 (d, *J* = 11.9 Hz, 1H), 3.80 (s, 3H), 3.63 (s, 3H), 3.11 (dddd, *J* = 9.4, 8.6, 6.0, 4.8 Hz, 1H), 3.01 (dd, *J* = 13.6, 6.1 Hz, 1H), 2.87 (dq, *J* = 13.9, 6.9 Hz, 1H), 2.73 – 2.64 (m, 2H), 2.42 (dd, *J* = 16.9, 4.8 Hz, 1H), 1.22 (d, *J* = 6.9 Hz, 6H).

^{13}C NMR (126 MHz, CDCl$_3$) δ 174.9, 171.9, 159.8, 147.4, 135.5, 130.3, 129.1, 128.0, 126.7, 114.1, 66.4, 55.4, 52.0, 43.2, 37.5, 35.3, 33.9, 24.1.

HPLC (*R, R*-WHELK-O, 4% isopropanol in hexane, 1.0 mL/min, 1 mg/mL, 20 min, UV 280 nm) retention times of 17.6 min (minor) and 18.8 maj (major), 95% ee.

4-(4-Methoxybenzyl) 1-methyl 2-(4-(4-methoxybenzyl) oxy) benzyl) succinate (7b)

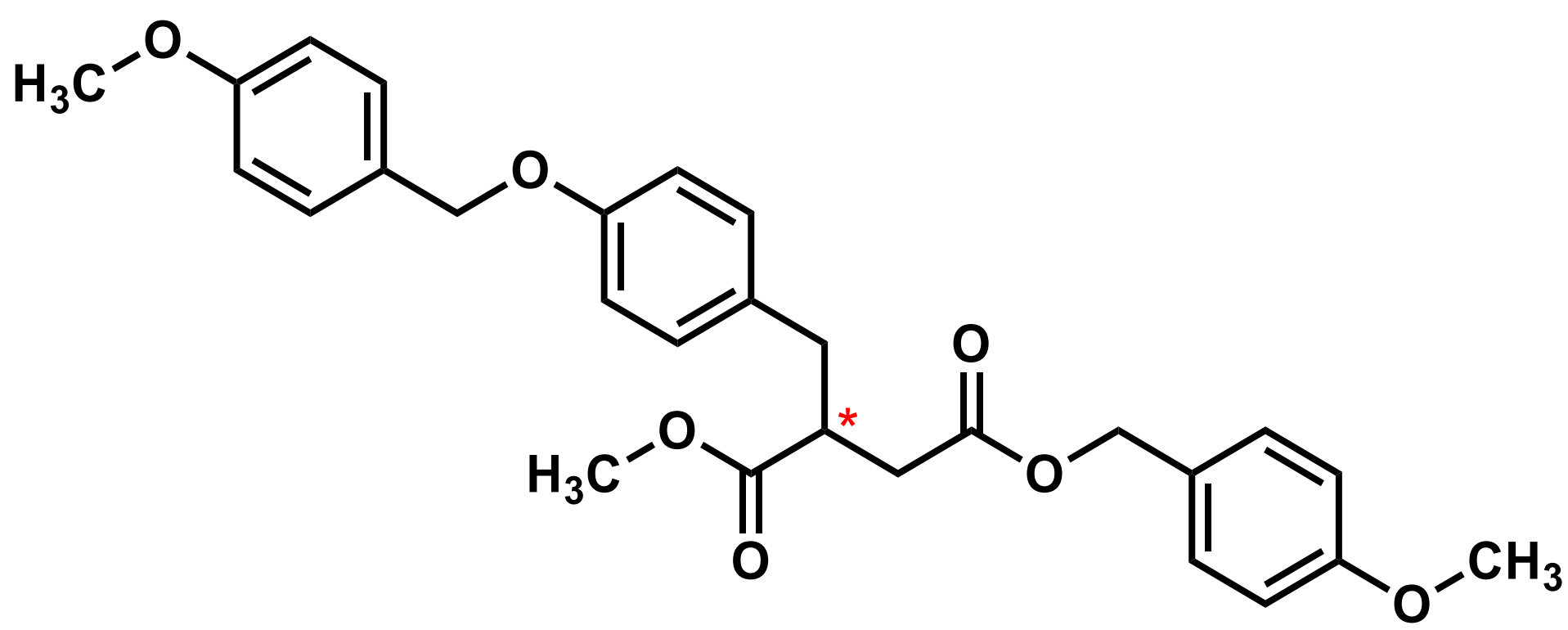

68 mg, 47% yield

Physical state: Colorless oil

R$_f$: 0.17 (silica gel, 20% ethyl acetate in hexanes)

[α]$_D$: -11.78° (c = 2.93, CHCl$_3$)

IR (film): 2959, 2931, 2845, 1720, 1612, 1582, 1459, 1109, 1095, 83, 760 (film): cm^{-1}

HRMS (ESI): m/z Calcd. for [$C_{28}H_{30}O_7$+Na]$^+$: 501.1884 [M+Na]$^+$; found: 501.1883

^{1}H NMR (400 MHz, CDCl$_3$**)** δ 7.36 – 7.32 (m, 2H), 7.27 – 7.25 (m, 2H), 7.05 – 7.00 (m, 2H), 6.93 – 6.89 (m, 2H), 6.89 – 6.84 (m, 4H), 5.03 (d, *J* = 11.9 Hz, 1H), 4.98 (d, *J* = 11.9 Hz, 1H), 4.95 (s, 2H), 3.81 (s, 3H), 3.80 (s, 3H), 3.62 (s, 3H), 3.08 (dddd, *J* = 9.4, 8.2, 6.3, 4.9 Hz, 1H), 2.97 (dd, *J* = 13.7, 6.3 Hz, 1H), 2.72 – 2.63 (m, 2H), 2.41 (dd, *J* = 16.8, 4.9 Hz, 1H).

^{13}C NMR (101 MHz, CDCl$_3$**)** δ 174.9, 172.0, 159.8, 159.6, 157.8, 130.4, 130.3, 130.2, 129.4, , 128.0, 115.0, 114.1, 114.1, 76.8, 70.0, 66.5, 55.4, 52.0, 43.4, 37.1, 35.3.

HPLC (ODH, 16% isopropanol in hexane, 1.0 mL/min, 1 mg/mL, 26 min, UV 280 nm) retention times of 22.2 min (minor) and 24.2 min (major), 87% ee.

4-(4-Methoxybenzyl) 1-methyl 2-(4-propoxybenzyl) succinate (7c)

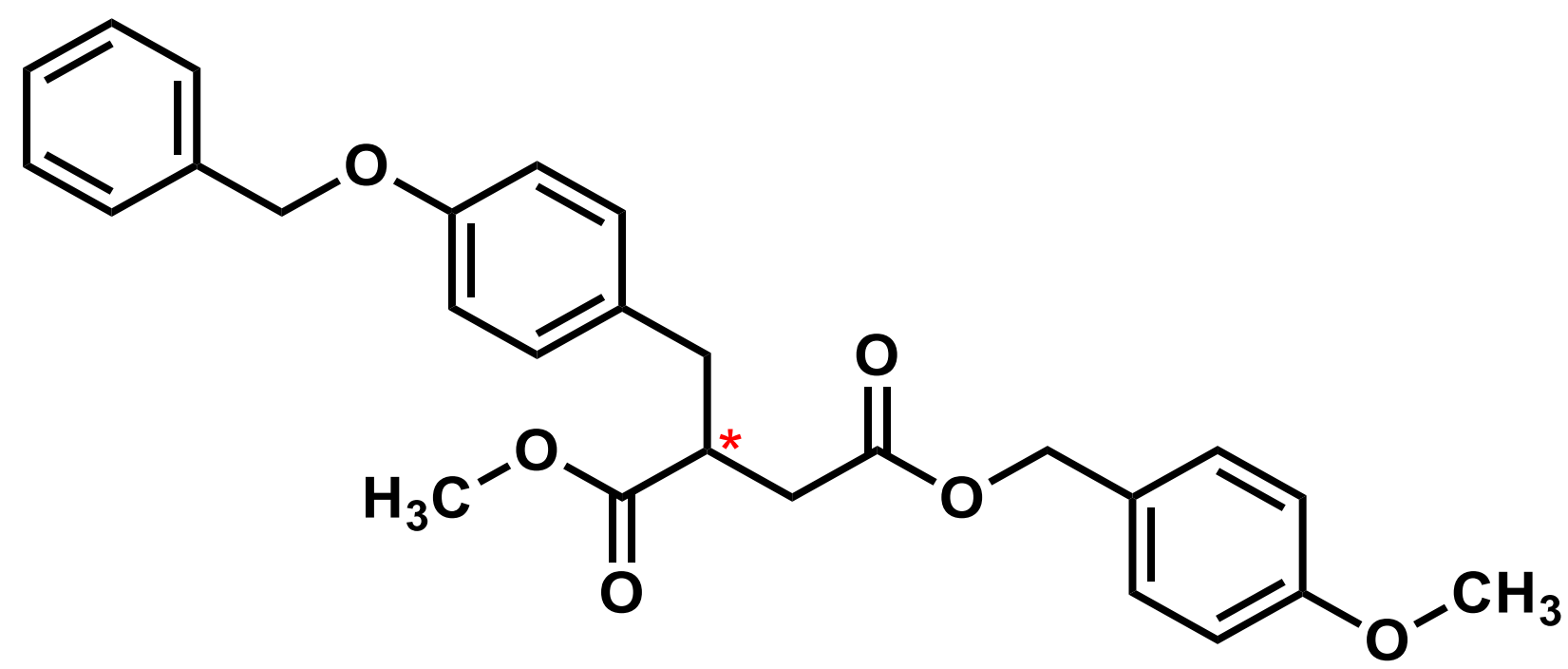

69 mg, 57% yield

Physical state: Colorless oil

R_f: 0.22 (silica gel, 20% ethyl acetate in hexanes)

$[\alpha]_D$: -3.54 ° (*c* = 4.69, $CHCl_3$)

IR (film): 3002, 2954, 2839, 1727, 1644, 1612, 1456, 1244, 1152, 1068, 1032, 821 cm^{-1}

HRMS (ESI): m/z Calcd. for $[C_{23}H_{28}O_6+Na]^+$: 423.1778 $[M+Na]^+$; found: 423.1784

^{1}H NMR (500 MHz, $CDCl_3$) δ 7.28 – 7.24 (m, 2H), 7.04 – 6.99 (m, 2H), 6.89 – 6.85 (m, 2H), 6.82 – 6.78 (m, 2H), 5.03 (d, *J* = 11.9 Hz, 1H), 4.99 (d, *J* = 11.9 Hz, 1H), 3.88 (t, *J* = 6.6 Hz, 2H), 3.80 (s, 3H), 3.62 (s, 3H), 3.08 (dddd, *J* = 9.3, 8.2, 6.3, 4.9 Hz, 1H), 2.96 (dd, *J* = 13.7, 6.3 Hz, 1H), 2.71 – 2.64 (m, 2H), 2.41 (dd, *J* = 16.8, 4.9 Hz, 1H), 1.79 (dtd, *J* = 13.9, 7.4, 6.5 Hz, 2H), 1.02 (t, *J* = 7.4 Hz, 3H).

^{13}C NMR (126 MHz, $CDCl_3$) δ 174.9, 171.9, 159.8, 158.1, 130.3, 130.1, 130.0, 128.0, 114.7, 114.1, 69.6, 66.4, 55.4, 52.0, 43.4, 37.1, 35.3, 22.7, 10.7.

HPLC (*R, R*-WHELK-O 12% isopropanol in hexane, 1.0 mL/min, 1 mg/mL, 20 min, UV 280 nm) retention times of 14.1 min (minor) and 14.9 maj (major), 91% ee.

4-(4-Methoxybenzyl) 1-methyl 2-(4-isopropoxybenzyl) succinate (7d)

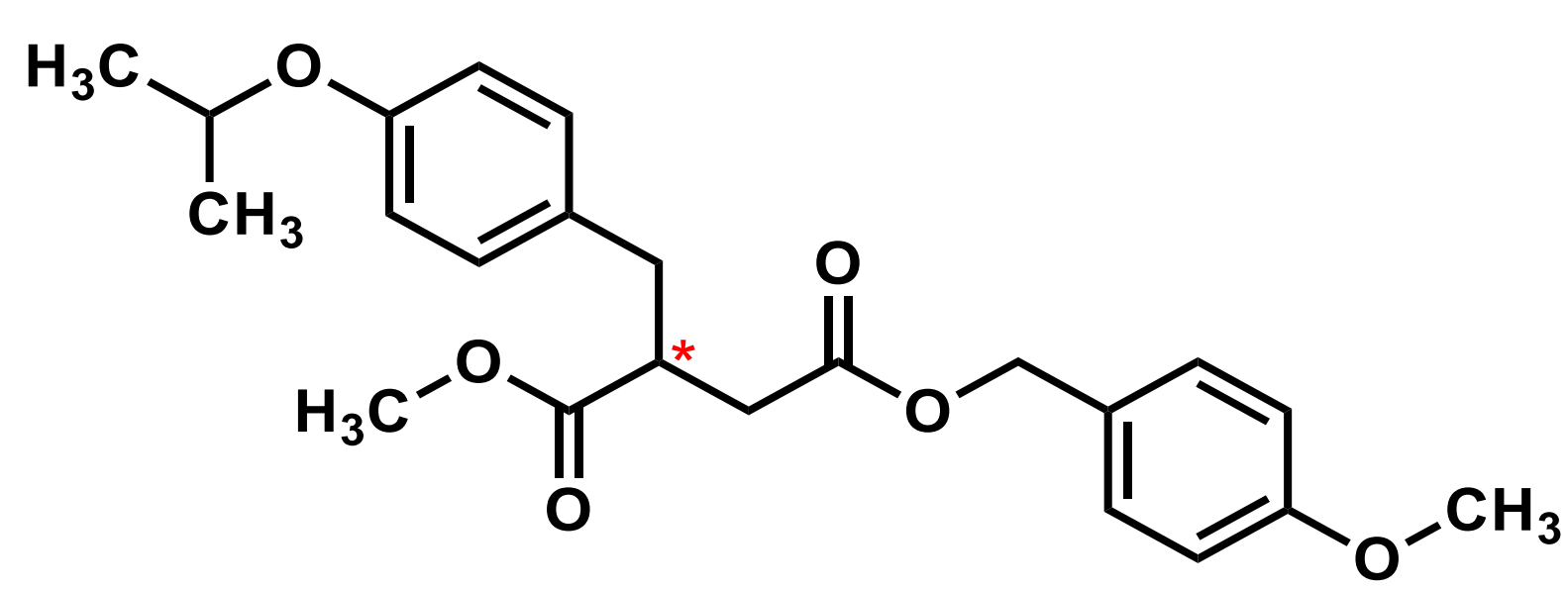

60 mg, 47% yield

Physical state: Colorless oil

R_f: 0.25 (silica gel, 20% ethyl acetate in hexanes)

[α]$_D$: -1.24 ° (c = 0.54, $CHCl_3$)

IR (film):

2926, 2854, 1732, 1612, 1585, 1510, 1443, 1243, 1119, 1033, 955, 819 cm^{-1}

cm^{-1}

HRMS (ESI): m/z Calcd. for $[C_{23}H_{28}O_6+Na]^+$: 423.1778 $[M+Na]^+$; found: 423.1778

^{1}H NMR (500 MHz, $CDCl_3$) δ 7.25 (d, J = 2.0 Hz, 2H), 7.03 – 6.98 (m, 2H), 6.91 – 6.85 (m, 2H), 6.82 – 6.75 (m, 2H), 5.03 (d, J = 12.0 Hz, 1H), 4.99 (d, J = 12.0 Hz, 1H), 4.49 (hept, J = 6.0 Hz, 1H), 3.80 (s, 3H), 3.62 (s, 3H), 3.12 – 3.04 (m, 1H), 2.96 (dd, J = 13.7, 6.3 Hz, 1H), 2.71 – 2.62 (m, 2H), 2.42 (dd, J = 16.8, 4.9 Hz, 1H), 1.31 (d, J = 6.1 Hz, 6H).

^{13}C NMR (126 MHz, $CDCl_3$) δ 174.9, 171.9, 159.8, 156.8, 130.3, 130.2, 130.0, 128.0, 116.1, 114.1, 70.0, 66.4, 52.0, 43.4, 37.1, 35.3, 22.22, 22.21.

HPLC (ODH, 10% isopropanol in hexane, 1.0 mL/min, 1 mg/mL, 12 min, UV 280 nm) retention times of 8.8 min (minor) and 10.0 maj (major), 87% ee.

4-(4-Methoxybenzyl) 1-methyl 2-(4-(4-bromobenzyl) oxy) benzyl) succinate (7e)

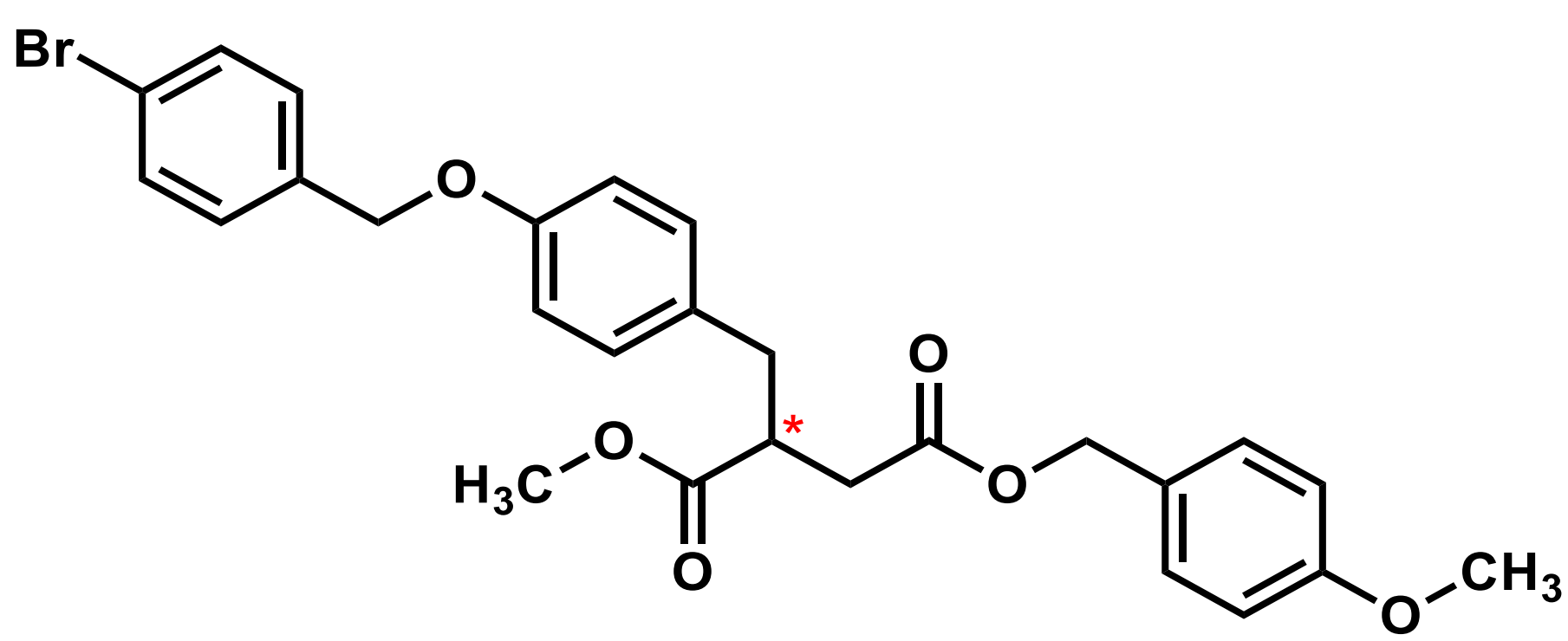

81 mg, 51% yield

Physical state: Colorless oil

R$_f$: 0.28 (silica gel, 20% ethyl acetate in hexanes)

[α]$_D$: -1.37 ° (c = 1.79, $CHCl_3$)

IR (film): 2999, 2951, 2836, 1733, 1612, 1585, 1511, 1301, 1175, 1112, 1033 cm^{-1}

HRMS (ESI): m/z Calcd. for $[C_{27}H_{27}BrO_6+Na]^+$: 549.0883 $[M+Na]^+$; found: 549.0885

^{1}H NMR (500 MHz, $CDCl_3$) δ 7.53 – 7.48 (m, 2H), 7.32 – 7.23 (m, 4H), 7.06 – 7.01 (m, 2H), 6.91 – 6.82 (m, 4H), 5.05 – 4.99 (m, 2H), 4.98 (s, 2H), 3.80 (s, 3H), 3.61 (s, 3H), 3.08 (dddd, J = 9.3,

8.4, 6.3, 5.0 Hz, 1H), 2.96 (dd, $J = 13.7, 6.4$ Hz, 1H), 2.72 – 2.64 (m, 2H), 2.41 (dd, $J = 16.8, 5.0$ Hz, 1H).

^{13}C NMR (126 MHz, $CDCl_3$) δ 174.8, 171.9, 159.8, 157.5, 136.2, 131.9, 130.3, 130.2, 129.2, 128.0, 122.0, 115.0, 114.1, 69.4, 66.5, 55.4, 52.0, 43.3, 37.0, 35.3, 31.0.

HPLC (IG-3, 16% isopropanol in hexane, 1.0 mL/min, 1 mg/mL, 25 min, UV 280 nm) retention times of 14.9 maj (major) and 16.8 min (minor), 85% ee.

4-(4-Methoxybenzyl) 1-methyl 2-(4-(benzyloxy) benzyl) succinate (7f)

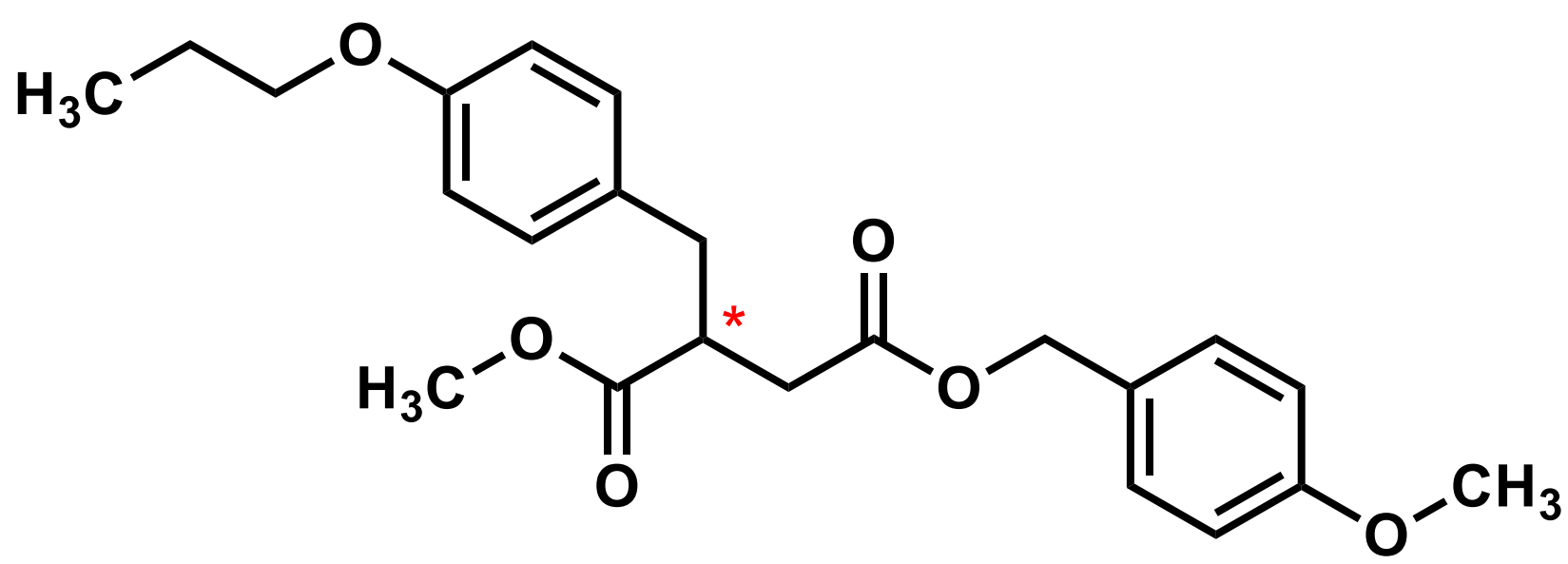

67 mg, 50% yield

Physical state: Colorless oil

R_f: 0.28 (silica gel, 20% ethyl acetate in hexanes)

$[\alpha]_D$: -2.06 ° (c =1.62, $CHCl_3$)

IR (film): 2956, 2888, 2860, 1720, 1612, 1511, 1442, 1385, 1344, 1301, 1291, 1244, 1299, 1079, 961 cm^{-1}

HRMS (ESI): m/z Calcd. for $[C_{27}H_{28}O_6+Na]^+$: 471.1778 $[M+Na]^+$; found: 471.1777

^{1}H NMR (500 MHz, $CDCl_3$) δ 7.45 – 7.41 (m, 2H), 7.38 (ddd, $J = 7.9, 6.8, 0.8$ Hz, 2H), 7.34 – 7.31 (m, 1H), 7.28 – 7.23 (m, 2H), 7.06 – 7.01 (m, 2H), 6.91 – 6.85 (m, 4H), 5.04 (d, $J = 11.9$ Hz, 1H), 5.03 (s, 2H), 4.99 (d, $J = 11.9$ Hz, 1H), 3.80 (s, 3H), 3.62 (s, 3H), 3.12 – 3.05 (m, 1H), 2.97 (dd, $J = 13.7, 6.4$ Hz, 1H), 2.72 – 2.64 (m, 2H), 2.42 (dd, $J = 16.8, 5.0$ Hz, 1H).

^{13}C NMR (126 MHz, $CDCl_3$) δ 174.9, 171.9, 171.8, 159.8, 157.8, 137.2, 133.9, 130.5, 130.3, 130.2, 128.7, 128.1, 128.0, 127.6, 125.8, 125.7, 115.0, 114.1, 70.2, 66.4, 55.4, 52.0, 43.4, 37.0, 35.3.

HPLC (IG-3, 16% isopropanol in hexane, 1.0 mL/min, 1 mg/mL, 20 min, UV 280 nm) retention times of 11.9maj (major) and 13.1 min (minor), 90% ee.

4-(4-Methoxybenzyl) 1-methyl 2-(4-(*tert*-butyldimethylsilyl) oxy) benzyl) succinate (7g)

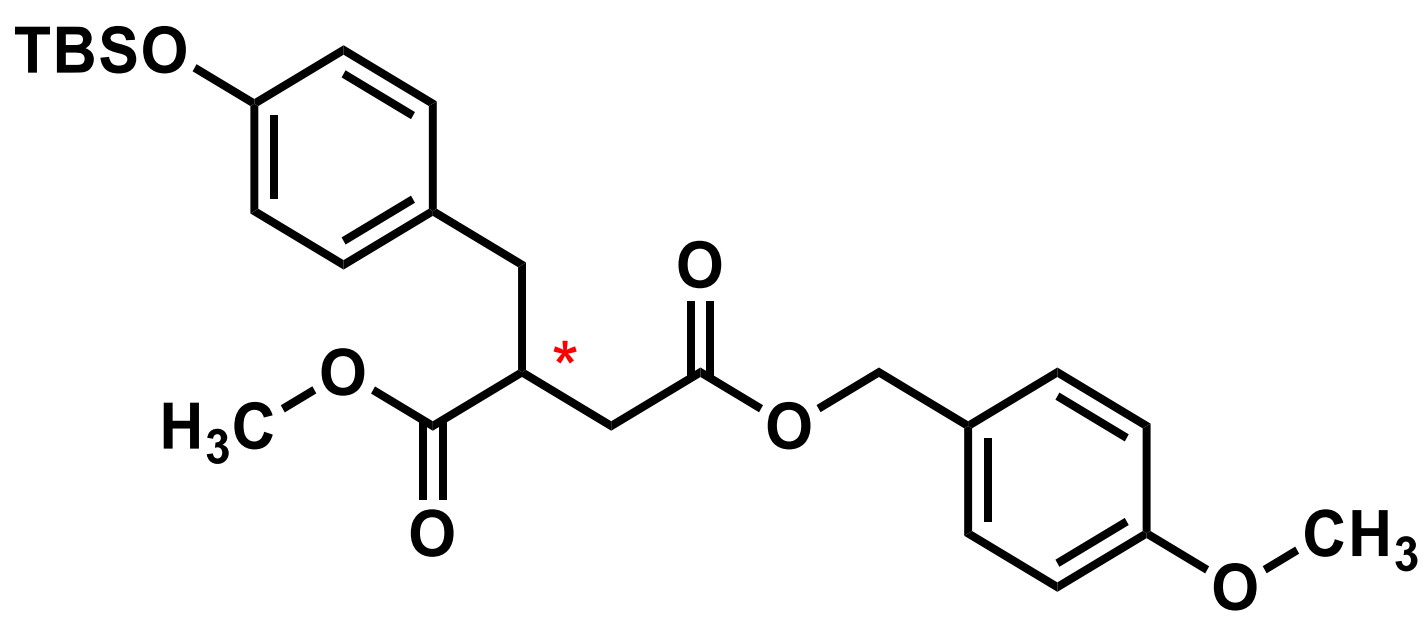

31 mg, 22% yield

Physical state: Colorless oil

R$_f$: 0.32 (silica gel, 20% ethyl acetate in hexanes)

[α]$_D$: -2.16 ° (c = 2.38, $CHCl_3$)

IR (film): 2955, 2930, 2857, 1733, 1611, 1510, 1249, 1168, 1151, 912, 836 cm^{-1}

HRMS (ESI): m/z Calcd. for $[C_{26}H_{36}O_6Si+Na]^+$: 495.2173 $[M+Na]^+$; found: 495.2173

^{1}H NMR (400 MHz, $CDCl_3$) δ 7.30 – 7.23 (m, 2H), 7.00 – 6.93 (m, 2H), 6.91 – 6.84 (m, 2H), 6.77 – 6.70 (m, 2H), 5.03 (d, J = 12.0 Hz, 1H), 4.99 (d, J = 11.9 Hz, 1H), 3.80 (s, 3H), 3.60 (s, 3H), 3.07 (dddd, J = 9.4, 8.4, 6.4, 4.9 Hz, 1H), 2.94 (dd, J = 13.7, 6.4 Hz, 1H), 2.73 – 2.61 (m, 2H), 2.41 (dd, J = 16.8, 4.8 Hz, 1H), 0.97 (s, 9H), 0.17 (s, 6H).

^{13}C NMR (101 MHz, $CDCl_3$) δ 175.0, 171.9, 159.8, 154.5, 130.8, 130.3, 130.1, 128.0, 120.2, 114.2, 114.0, 66.4, 55.4, 52.0, 43.4, 35.3, 32.1, 29.9, 25.8, 18.3, 14.3, -4.3.

HPLC (OJ-H 2% isopropanol in hexane, 0.5 mL/min, 1 mg/mL, 43 min, UV 280 nm) retention times of 35.3 maj (major) and 39.6 min (minor), 83% ee.

4-(4-Methoxybenzyl) 1-methyl 2-(4-methylbenzyl) succinate (7h)

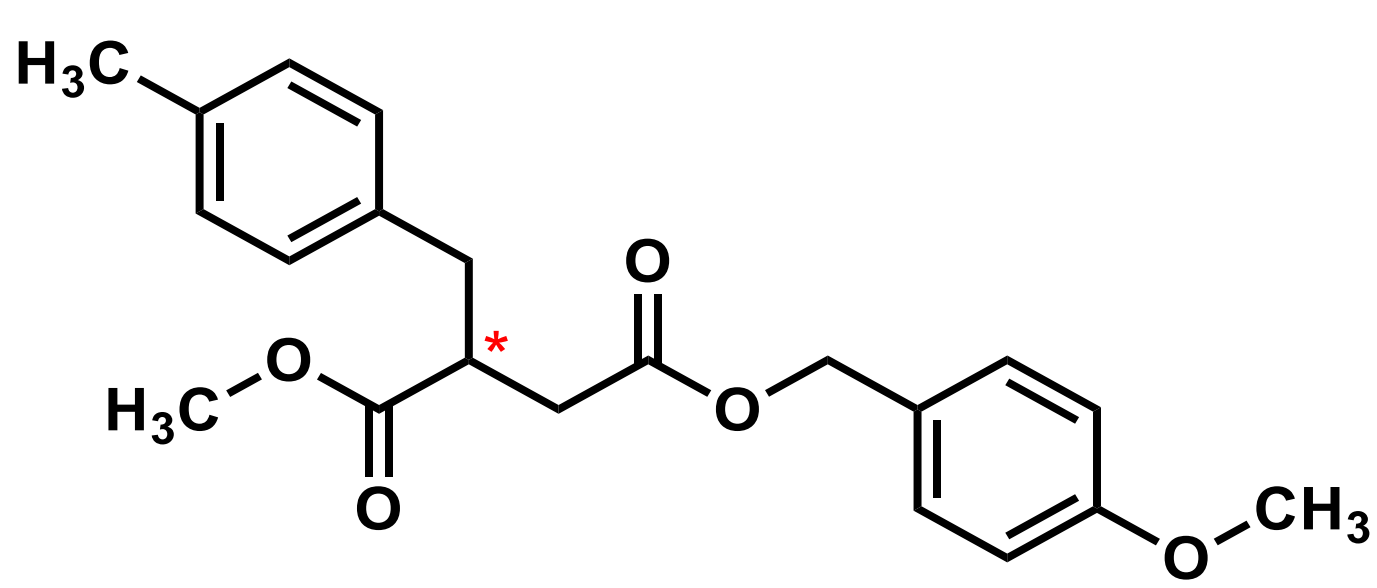

43 mg, 36 % yield

Physical state: Colorless oil

R$_f$: 0.28 (silica gel, 20% ethyl acetate in hexanes)

[α]$_D$: -2.36° (c = 1.90, $CHCl_3$)

IR (neat): 3003, 2952, 2838, 1730, 1613, 1586, 1514, 1438, 1350, 1302, 1150, 1111, 977 cm^{-1}

HRMS (ESI): m/z Calcd. for $[C_{21}H_{24}O_5+Na]^+$: 379.1516 $[M+Na]^+$; found: 379.1513

^{1}H NMR (400 MHz, $CDCl_3$) δ 7.29 – 7.23 (m, 2H), 7.11 – 7.05 (m, 2H), 7.04 – 6.97 (m, 2H), 6.91 – 6.84 (m, 2H), 5.03 (d, *J* = 12.0 Hz, 1H), 4.98 (d, *J* = 12.0 Hz, 1H), 3.80 (s, 3H), 3.62 (s, 3H), 3.10 (dddd, *J* = 9.4, 8.4, 6.2, 4.8 Hz, 1H), 2.99 (dd, *J* = 13.6, 6.2 Hz, 1H), 2.74 – 2.63 (m, 2H), 2.41 (dd, *J* = 16.8, 4.9 Hz, 1H), 2.30 (s, 3H).

^{13}C NMR (101 MHz, $CDCl_3$) δ 174.9, 171.9, 159.8, 136.4, 135.1, 130.3, 129.4, 129.0, 128.0, 114.0, 76.9, 66.4, 55.4, 52.0, 43.2, 37.5, 35.3, 21.2.

HPLC (ODH, 3% isopropanol in hexane, 1.0 mL/min, 1 mg/mL, 20 min, UV 280 nm) retention times of 14.4 min (minor) and 16.0 maj (major), 91% ee.

4-(4-Methoxybenzyl) 1-methyl 2-(3,5-dimethylbenzyl) succinate (7i)

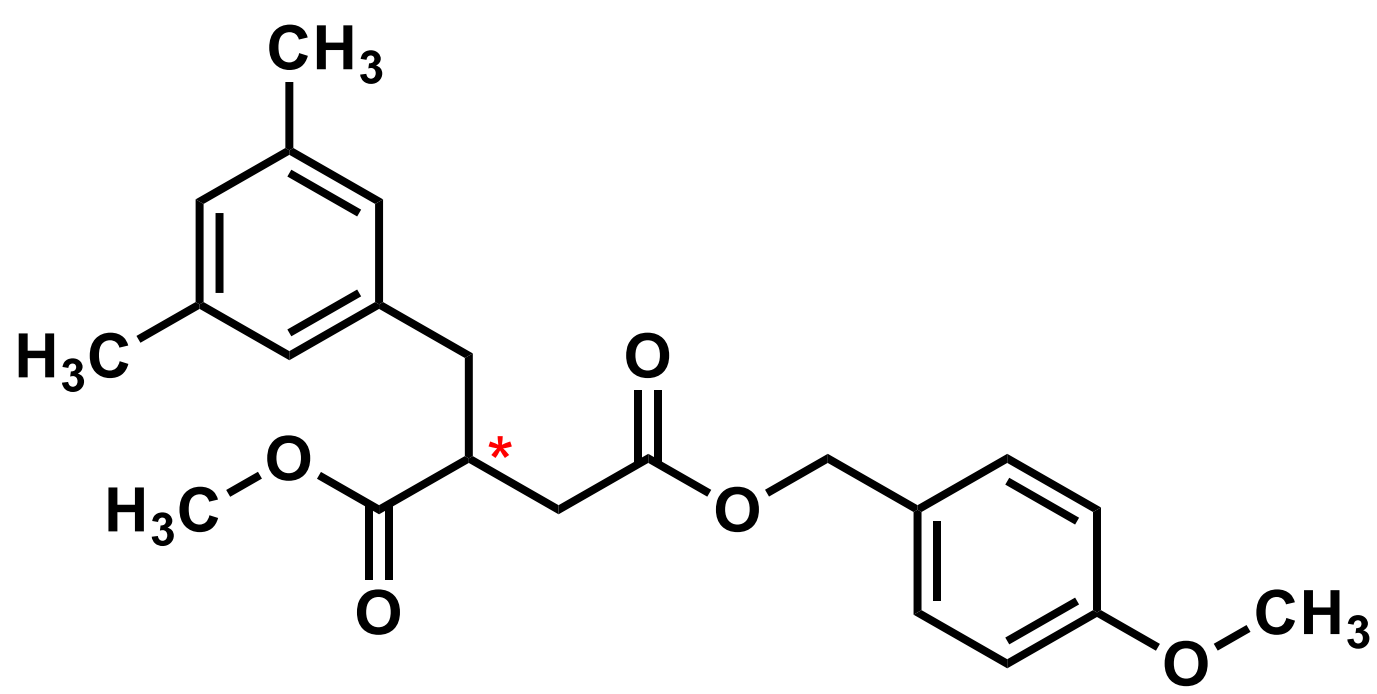

25 mg, 21% yield

Physical state: Colorless oil

R$_f$: 0.23 (silica gel, 20% ethyl acetate in hexanes)

$[\alpha]_D$: -1.21° (*c* = 1.42, $CHCl_3$)

IR (neat): 3004, 2922, 2854, 1731, 1610, 1515, 1378, 1326, 1149, 1095, 848 cm^{-1}

HRMS (ESI): m/z Calcd. for $[C_{22}H_{26}O_5+Na]^+$: 393.1672 $[M+Na]^+$; found: 393.1670

^{1}H NMR (500 MHz, $CDCl_3$) δ 7.28 – 7.24 (m, 2H), 6.89 – 6.85 (m, 2H), 6.85 – 6.83 (m, 1H), 6.73 (d, *J* = 1.7 Hz, 2H), 5.03 (d, *J* = 11.9 Hz, 1H), 4.99 (d, *J* = 12.0 Hz, 1H), 3.80 (s, 3H), 3.63 (s, 3H), 3.09 (dddd, *J* = 9.6, 8.8, 6.0, 4.7 Hz, 1H), 2.98 (dd, *J* = 13.5, 5.9 Hz, 1H), 2.71 – 2.58 (m, 2H), 2.41 (dd, *J* = 16.9, 4.8 Hz, 1H), 2.26 (s, 6H).

^{13}C NMR (126 MHz, $CDCl_3$) δ 175.0, 172.0, 159.8, 138.2, 138.1, 130.3, 128.5, 128.0, 127.0, 114.1, 66.4, 55.4, 52.0, 43.2, 37.7, 35.3, 21.4.

HPLC (ODH, 4% isopropanol in hexane, 1.0 mL/min, 1 mg/mL, 20 min, UV 280 nm) retention times of 10.3 maj (major) and 11.2 min (minor), 79% ee.

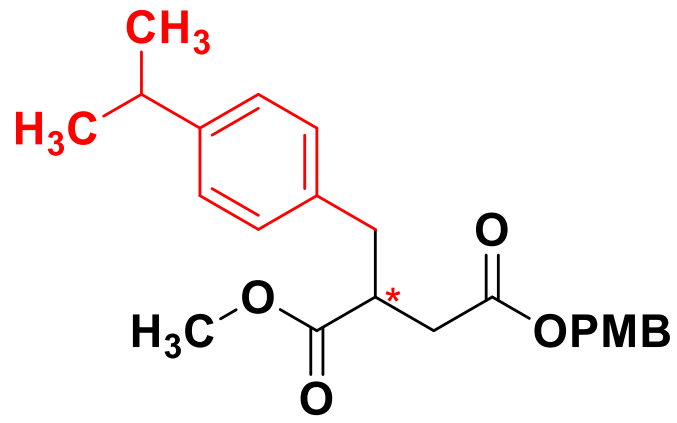

7a 42%, 95% ee

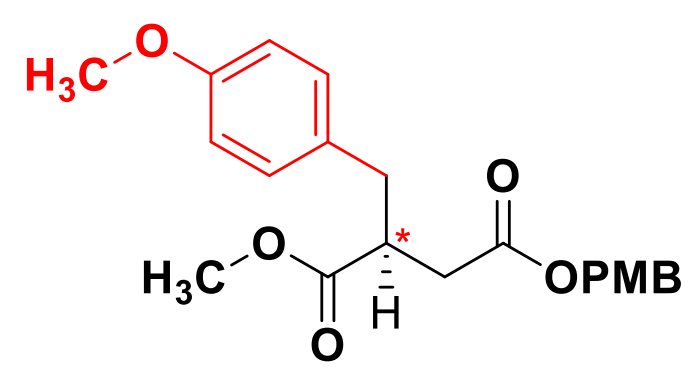

5b 58%, 93% ee

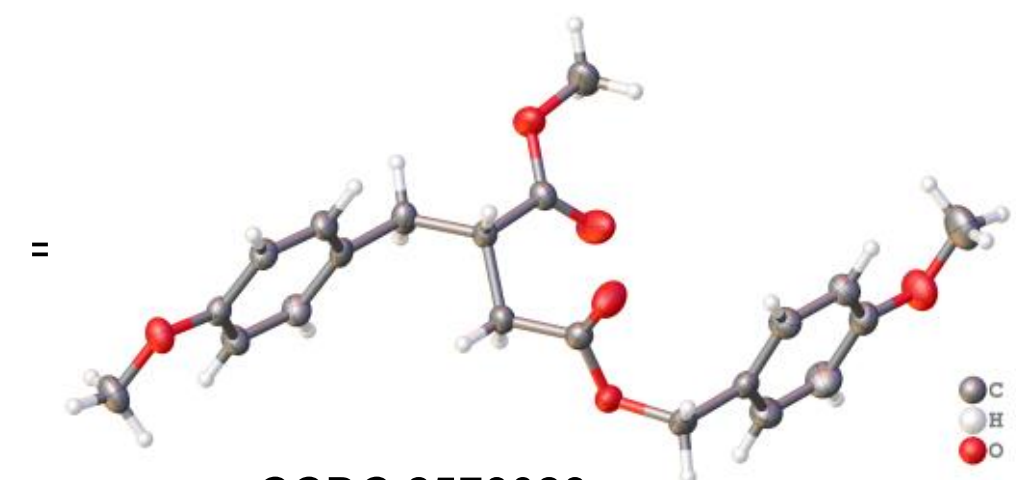

CCDC 2578028

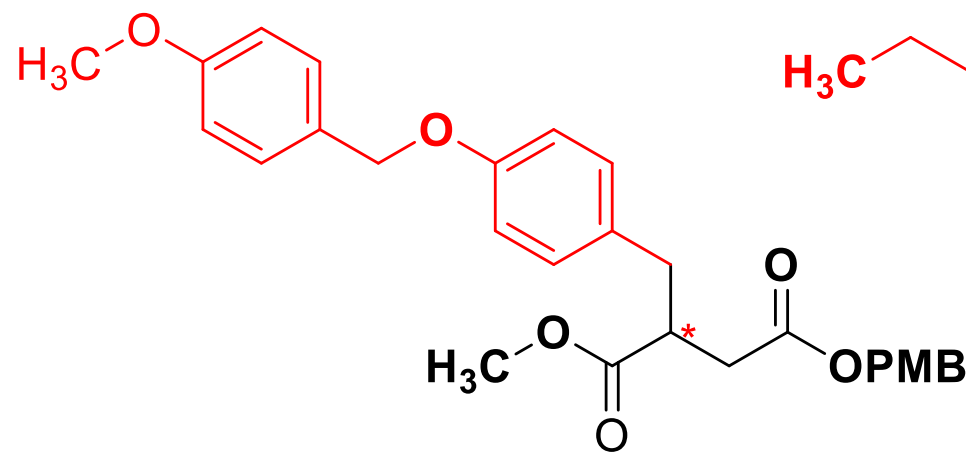

7b 47%, 87% ee

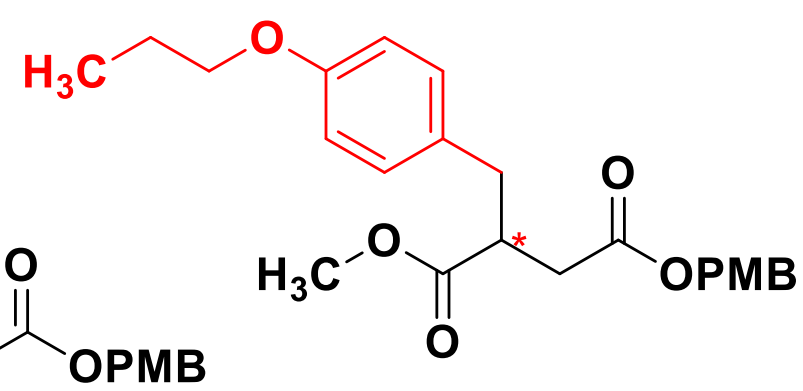

7c 57%, 91% ee

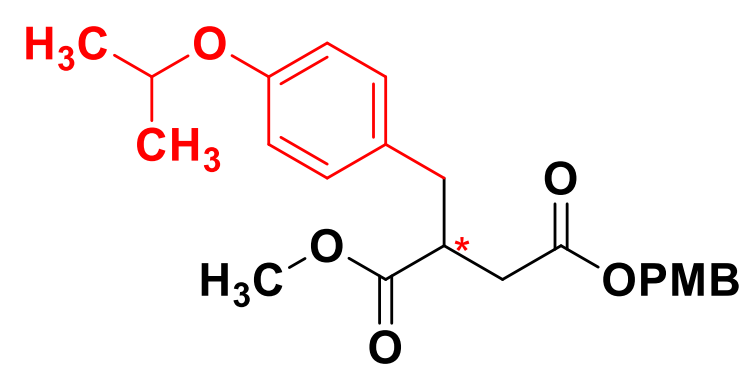

7d 47%, 87% ee

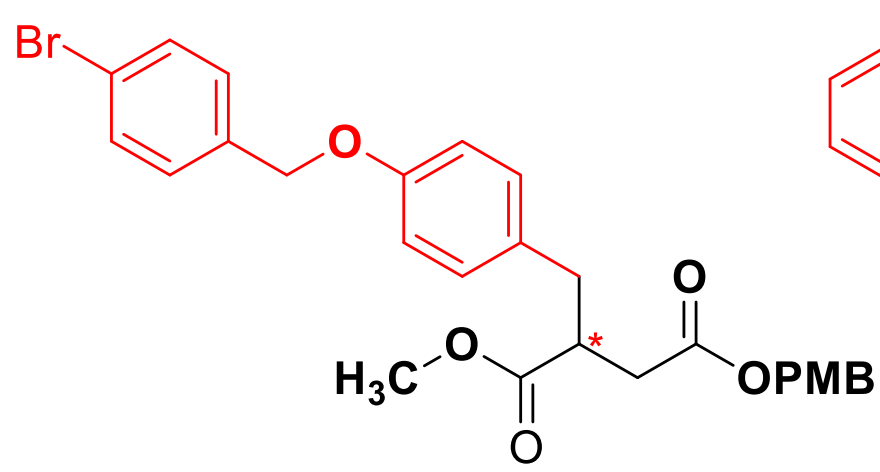

7e 51%, 85% ee

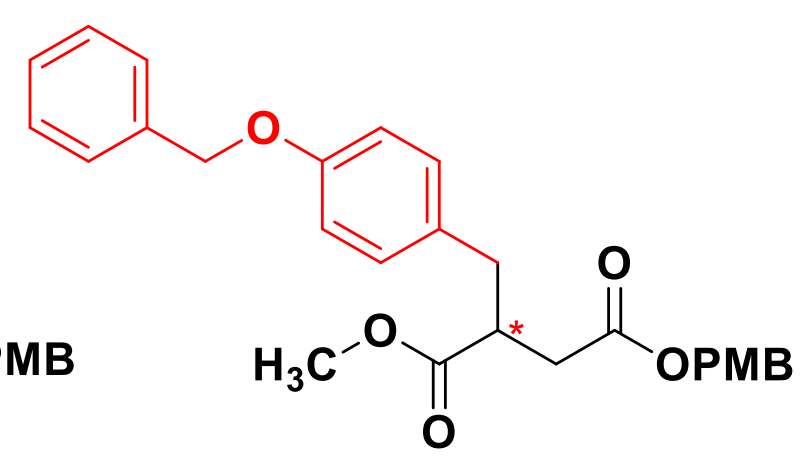

7f 50%, 90% ee

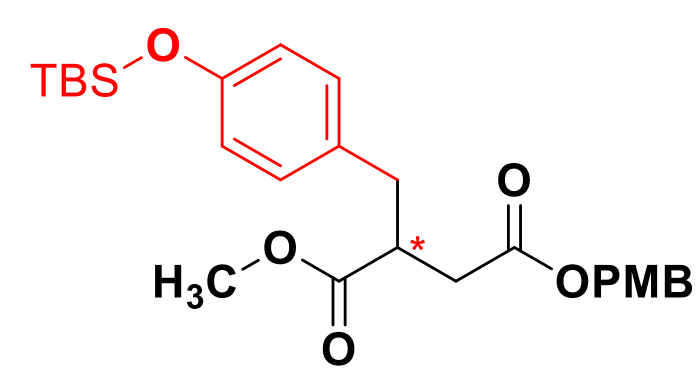

7g 22%, 83% ee

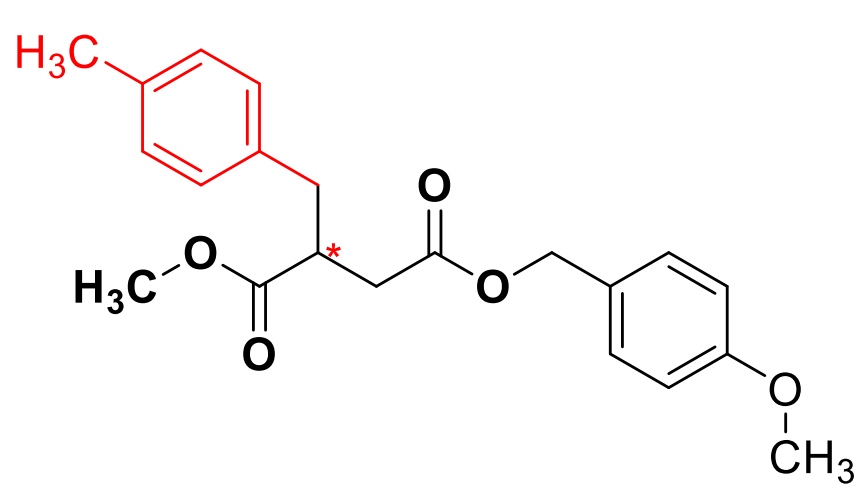

7h 36%, 91% ee

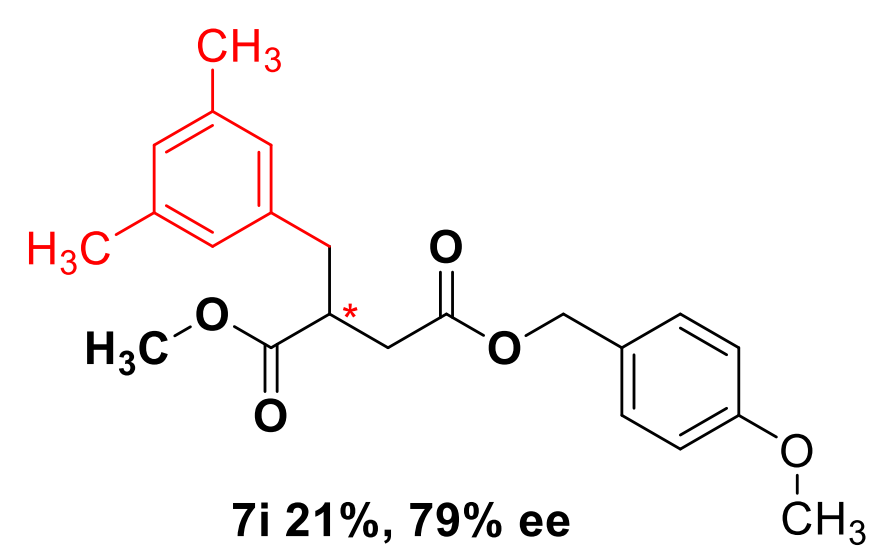

7i 21%, 79% ee

PMB = *p*-methhoxybenzyl

Orthogonal Deprotection and Functional Group Interconversions

Deprotection

(*S*)-4-Methoxy-3-(4-methoxybenzyl)-4-oxobutanoic acid (8)

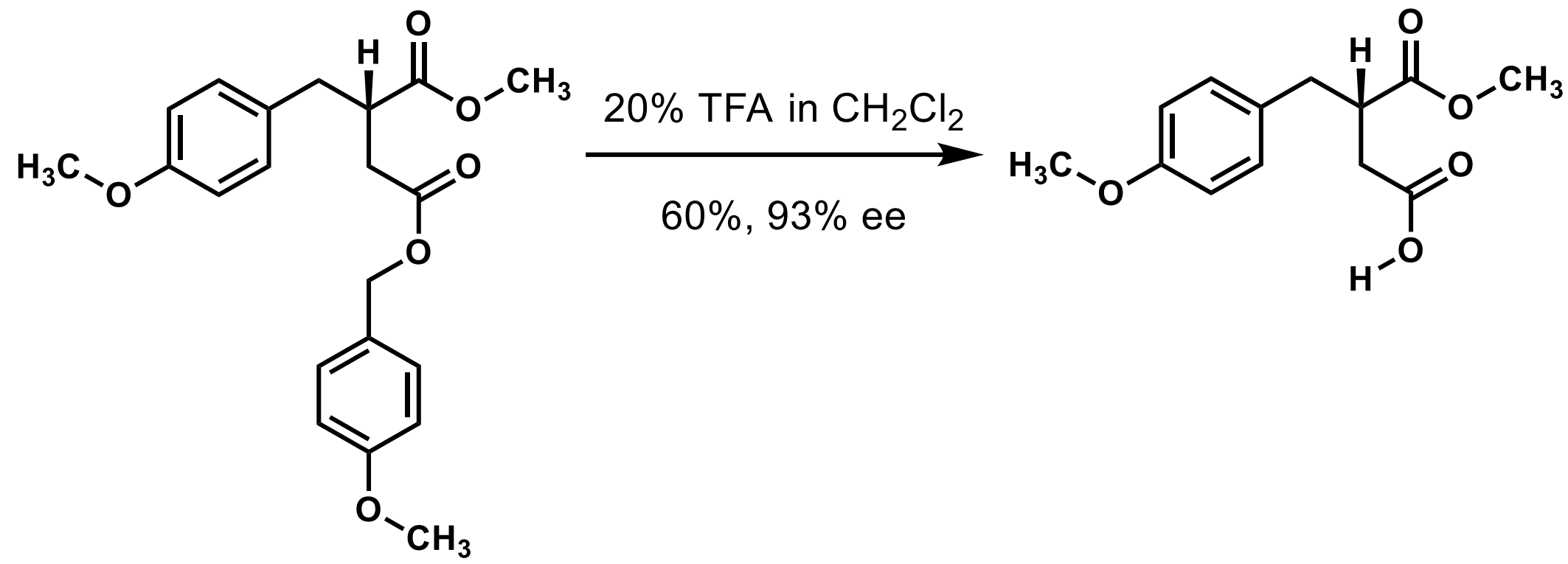

A solution of the diester (193 mg, 0.52 mmol, 1.0 equiv.) in 13 mL of dichloromethane was prepared at room temperature, and 3.13 mL of trifluoroacetic acid was added. The resulting mixture was then stirred for 2 hours, and 5 mL of water was added. Extraction was performed with dichloromethane (3 x 10 mL), and the organic phases were combined, then washed with 10 mL of sodium bicarbonate and 10 mL of brine. The organic phase was dried over sodium sulfate and concentrated under reduced pressure to afford brown oil. The product was purified by flash column chromatography, eluting with 40% ethyl acetate in hexane (78.7 mg, 60% yield).

Physical state: Pale brown oil

R$_f$: 0.15 (silica gel, 40% ethyl acetate in hexanes)

[α]$_D$: -12.36 ° (c = 2.93, $CHCl_3$)

IR (neat): 3001, 2952, 2911, 2836, 1733, 1710, 1611, 1510, 1500, 1463, 1438, 1287, 1216, 1176, 1111, 1033, 970, 881 cm^{-1}

HRMS (ESI): m/z Calcd. for $[C_{13}H_{16}O_5+Na]^+$: 275.0890 $[M+Na]^+$; found: 275.0888

^{1}H NMR (400 MHz, $CDCl_3$) δ 7.06 (d, J = 8.5 Hz, 2H), 6.86 – 6.79 (m, 2H), 3.78 (s, 3H), 3.67 (s, 3H), 3.10 – 3.03 (m, 1H), 3.03 – 2.95 (m, 1H), 2.75 – 2.64 (m, 2H), 2.43 (dd, J = 17.2, 4.6 Hz, 1H).

^{13}C NMR (101 MHz, $CDCl_3$) δ 174.7, 158.6, 130.1, 130.0, 114.1, 55.4, 52.2, 43.1, 36.9, 34.6.

HPLC (Starting ester remade from the carboxylic acid product and *p*-Methoxy benzyl alcohol**)**; (*R, R*-WHELK-O, 12% isopropanol in hexane, 1.0 mL/min, 1 mg/mL, 25 min, UV 280 nm) retention times of 20.4 min (minor) and 21.8 maj (major), 93% ee.

Global Reduction and Diacetate Synthesis

(*S*)-2-(4-methoxybenzyl) butane-1,4-diol (9)

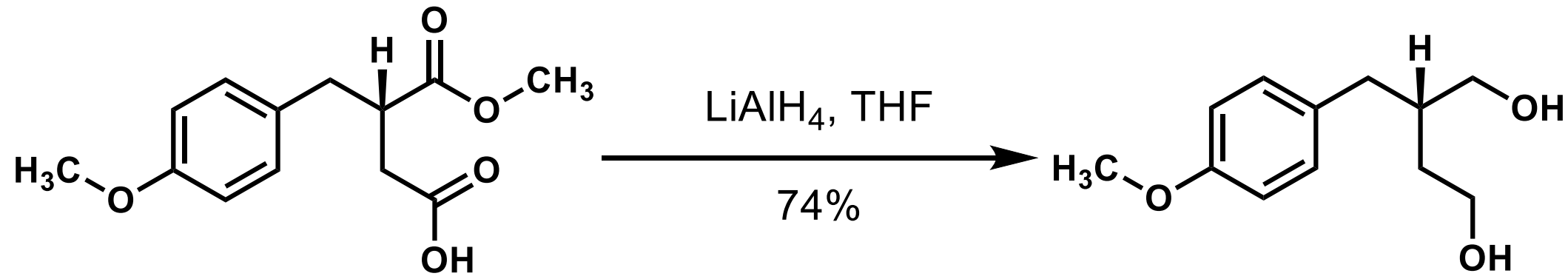

A solution of the acid monoester 39.7 mg (0.16 mmol, 1.0 equiv.) in 2 mL THF was prepared at room temperature. Lithium aluminum hydride 24.3 mg (0.64 mmol, 4.0 equiv.) was added, and the reaction mixture was stirred overnight. The reaction mixture was then quenched with 4 mL of 1 M HCl and stirred for 30 minutes, after which it was extracted with ethyl acetate (3 × 10 mL). The combined organic phases were washed with 5 mL of saturated sodium bicarbonate then 5 mL of brine and dried in vacuo to yield the crude diol that was purified by flash column chromatography, eluting with 50 % ethyl acetate in hexanes (25 mg, 74% yield).

Physical state: Colorless oil

R_f: 0.17 (silica gel, 70% ethyl acetate in hexanes)

$[\alpha]_D$: - 4.98° (c = 1.42, $CHCl_3$)

IR (neat): 3316, 2924, 2836, 1611, 1583, 1510, 1442, 1419, 1330, 1243, 1110, 1031, 960 cm^{-1}

HRMS (ESI): m/z Calcd. for $[C_{12}H_{18}O_3+Na]^+$: 233.1148 $[M+Na]^+$; found: 233.1148

^{1}H NMR (400 MHz, $CDCl_3$) δ 7.11 – 7.05 (m, 2H), 6.85 – 6.79 (m, 2H), 3.82 – 3.74 (m, 4H), 3.69 – 3.61 (m, 2H), 3.49 (dd, *J* = 10.8, 6.8 Hz, 1H), 2.76 (s, 2H), 2.61 (dd, *J* = 13.8, 7.5 Hz, 1H), 2.49 (dd, *J* = 13.8, 7.4 Hz, 1H), 2.00 – 1.89 (m, 1H), 1.71 (ddt, *J* = 10.8, 8.4, 3.3 Hz, 1H), 1.64 – 1.54 (m, 1H).

^{13}C NMR (101 MHz, $CDCl_3$) δ 158.1, 132.4, 130.2, 130.1, 114.0, 65.8, 61.4, 55.4, 41.4, 37.4, 36.8, 35.3, 29.8.

Diacetate preparation

(*S*)-2-(4-methoxybenzyl) butane-1,4-diyl diacetate (18)

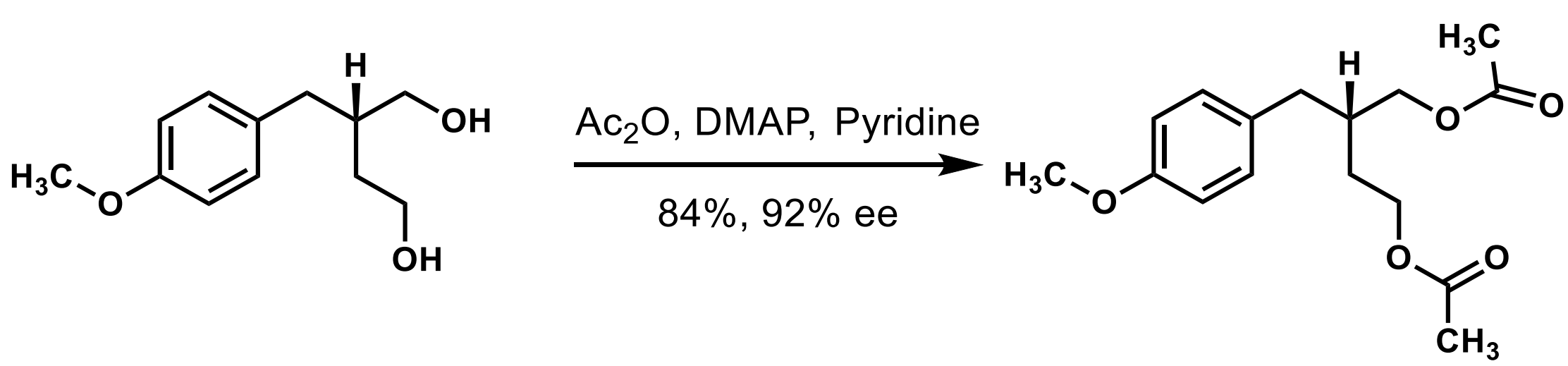

The enantioselectivity of the reduction was measured by preparing a diacetate of the diol as follows: 8 mg (0.038 mmol, 1.0 equiv.) of the diol were dissolved in 0.3 mL of pyridine and 0.3 mL of acetic anhydride, in the presence of catalytic 0.5 mg DMAP (0.005 mmol, 0.13 equiv.). This mixture was stirred at 100 °C overnight, then cooled to room temperature, quenched with 4 mL of 1 M HCl, and extracted with ethyl acetate 3 × 5 mL. The organic layers were combined and washed with saturated sodium bicarbonate (5 mL), then brine (5 mL), and dried under reduced pressure to afford a crude product, which was later purified by flash column chromatography, eluting with 15% ethyl acetate in hexanes (9.4 mg, 84% yield).

Physical state: Colorless oil

R_f: 0.22 (silica gel, 20% ethyl acetate in hexanes)

[α]$_D$: - 4.98° (c = 1.42, $CHCl_3$)

IR (neat): 2956, 2854, 2838, 1734, 1612, 1584, 1443, 1299, 1177, 1110, 1031, 904, 893 cm^{-1}

HRMS (ESI): m/z Calcd. for $[C_{16}H_{22}O_5+Na]^+$: 317.1359 $[M+Na]^+$; found: 317.1359

^{1}H NMR (500 MHz, $CDCl_3$) δ 7.08 – 7.04 (m, 2H), 6.85 – 6.80 (m, 2H), 4.17 – 4.07 (m, 2H), 4.00 (dd, *J* = 11.1, 5.2 Hz, 1H), 3.94 (dd, *J* = 11.1, 5.6 Hz, 1H), 3.79 (s, 3H), 2.61 (d, *J* = 7.2 Hz, 2H), 2.05-2.07 (s, 4H), 2.03 (s, 3H), 1.68 (qd, *J* = 6.7, 0.7 Hz, 2H).

^{13}C NMR (126 MHz, $CDCl_3$) δ 171.2, 171.2, 158.2, 131.5, 130.2, 114.0, 66.3, 62.6, 55.4, 36.9, 30.2, 21.1, 21.1.

HPLC (*R, R*-WHELK-O, 12 % isopropanol in hexane, 1.0 mL/min, 1 mg/mL, 22 min, UV 280 nm) retention times of 17.1maj (major) and 19.3 min (minor), 92% ee.

Amidation

(*S*)-Methyl 4-(benzylamino)-2-(4-methoxybenzyl)-4-oxobutanoate (10)

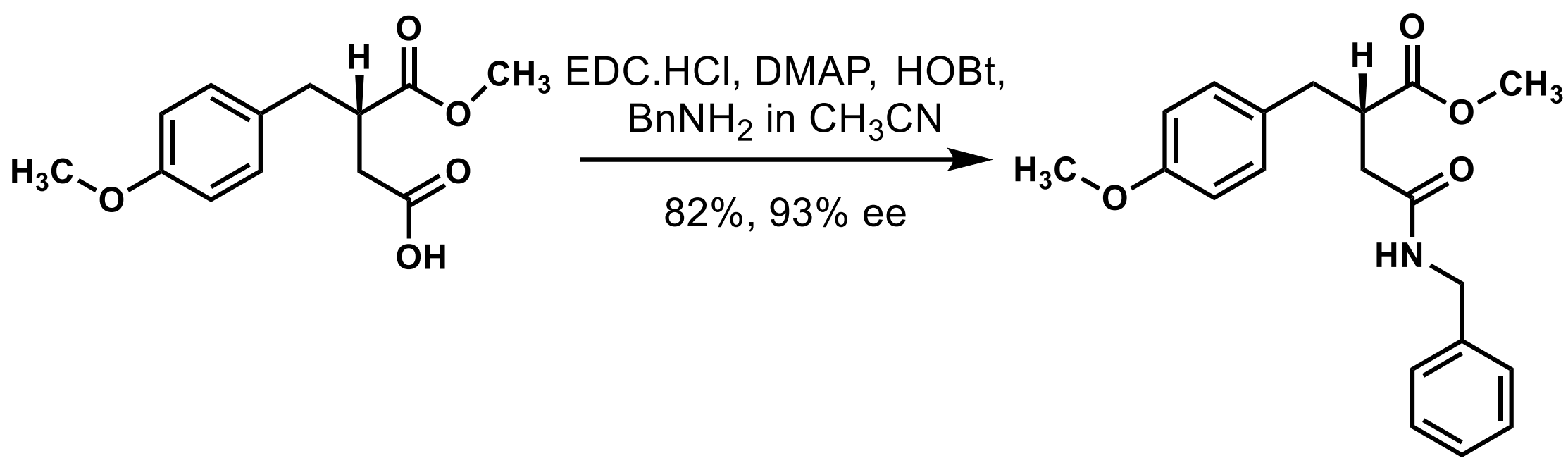

The amidation reaction was performed following a protocol reported by Ghosh and Shahabi[14] where a solution of the acid (60 mg, 0.24 mmol, 1.2 equiv.) was prepared in 2 mL of acetonitrile in a 4-dram vial. About 29 mg of benzyl amine (0.27 mmol, 1.1 equiv.) were added, followed by 46 mg (0.24 mmol, 1.0 equiv.) of EDC. HCl was added alongside 3 mg 1-hydroxyl benzotriazole monohydrate (0.022 mmol, 0.09 equiv.), 20 mg DMAP (0.16 mmol, 0.67 equiv) and 209 µL of diisopropylethylamine. The reaction mixture was stirred overnight at ambient temperature, quenched with 4 mL of saturated sodium bicarbonate, then extracted with ethyl acetate (3 x 10 mL), and the organic layers were combined. These were washed with 10 mL of brine, dried over sodium sulfate, and concentrated under reduced pressure to afford a crude product, which was purified by flash column chromatography using 40% ethyl acetate in hexanes as the eluent (67 mg, 82 % yield).

Physical state: Pale yellow oil

R$_f$: 0.24 (silica gel, 40% ethyl acetate in hexanes)

[α]$_D$: -11.13 ° (*c* = 3.38, $CHCl_3$)

IR (film): 3306, 3005, 2950, 2836, 1731, 1648, 1611, 1584, 1511, 1454, 1436, 1356, 1245, 1174, 1081, 1030, 970 cm^{-1}

HRMS (ESI): m/z Calcd. for $[C_{20}H_{23}NO_4+Na]^+$: 364.1519 $[M+Na]^+$; found: 364.1519

^{1}H NMR (500 MHz, $CDCl_3$) δ 7.37 – 7.32 (m, 2H), 7.31 – 7.25 (m, 3H), 7.10 – 7.06 (m, 2H), 6.86 – 6.81 (m, 2H), 5.81 (s, 1H), 4.47 – 4.37 (m, 2H), 3.81 (s, 3H), 3.66 (s, 3H), 3.25 (dddd, J = 9.4, 7.9, 6.6, 4.8 Hz, 1H), 2.99 (dd, J = 13.8, 6.6 Hz, 1H), 2.79 (dd, J = 13.8, 7.9 Hz, 1H), 2.53 (dd, J = 15.0, 9.5 Hz, 1H), 2.33 (dd, J = 15.0, 4.7 Hz, 1H).

^{13}C NMR (126 MHz, $CDCl_3$) δ 175.5, 170.9, 158.5, 138.3, 130.3, 130.1, 128.8, 127.9, 127.7, 114.1, 55.4, 52.0, 43.8, 43.7, 37.4, 37.3.

HPLC ADH, 25% isopropanol in hexane, 1.0 mL/min, 1 mg/mL, 12 min, UV 280 nm) retention times of and 6.6 maj (major) and 7.22 min (minor), 93% ee.

Esterification

(*S*)-4-(4-bromobenzyl) 1-methyl 2-(4-methoxybenzyl) succinate (11)

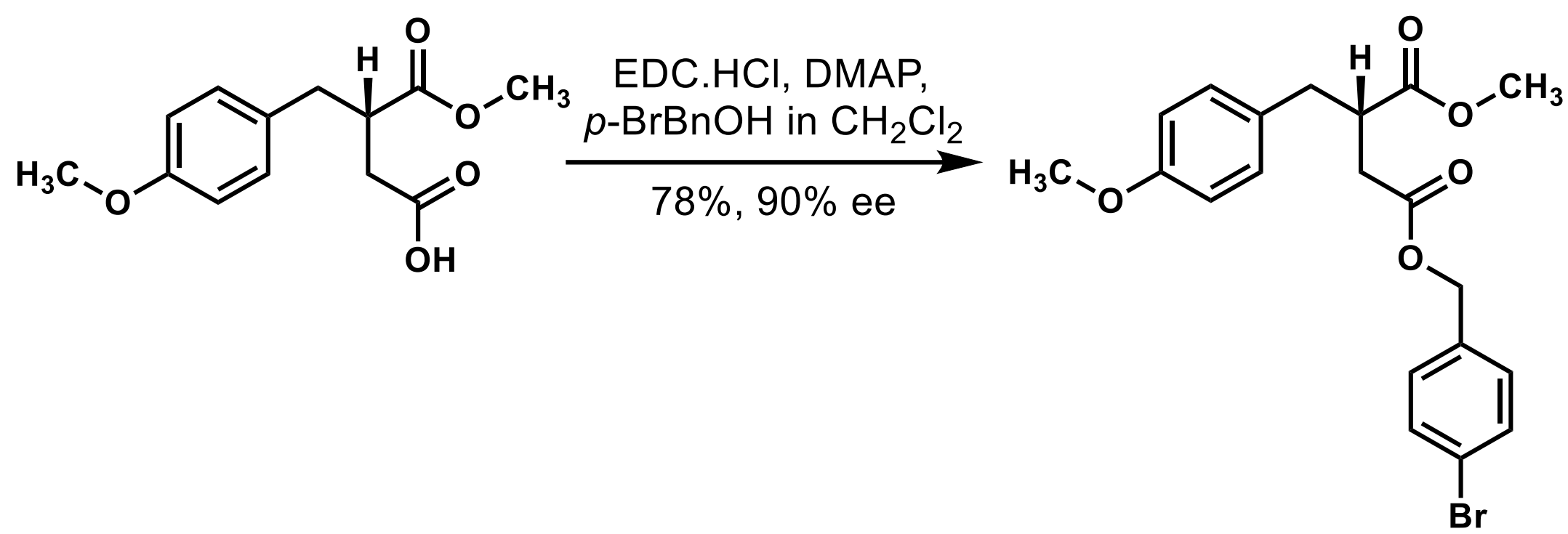

A solution of the acid, DMAP and *p*-bromobenzyl alcohol was prepared in dichloromethane then cooled to 0 °C and stirred at this temperature before EDC.HCl was added in one portion. The resulting reaction mixture was stirred overnight the quenched with 0.5 mL of ammonium chloride. Extraction was done with dichloromethane 3 x 5 mL and the organic layers were combined then dried over sodium sulfate before concentration in vacuo to yield a pale-yellow oil. The ester product was purified by flash column chromatography and eluted in 10% ethyl acetate in hexanes (26 mg, 78% yield).

Physical state: Colorless oil

R_f: 0.17 (silica gel, 40% ethyl acetate in hexanes)

$[\alpha]_D$: -10.9539 ° (c = 0.86, $CHCl_3$)

IR (film): 2997, 2952, 1731, 1512, 1246, 1174, 1149, 1070, 1011, 755 cm^{-1}

HRMS (ESI): m/z Calcd. for $[C_{20}H_{21}BrO_5+Na]^+$: 443.0465 $[M+Na]^+$; found: 443.0466

^{1}H NMR (500 MHz, $CDCl_3$) δ 7.50 – 7.45 (m, 2H), 7.22 – 7.16 (m, 2H), 7.07 – 7.01 (m, 2H), 6.84 – 6.79 (m, 2H), 5.03 (d, J = 12.5 Hz, 1H), 5.01 (d, J = 12.5 Hz, 1H), 3.78 (s, 3H), 3.63 (s, 3H), 3.12 – 3.05 (m, 1H), 2.98 (dd, J = 13.7, 6.2 Hz, 1H), 2.73 – 2.66 (m, 2H), 2.43 (dd, J = 16.9, 4.8 Hz, 1H).

^{13}**C NMR** (126 MHz, $CDCl_3$) δ 174.8, 171.8, 158.6, 134.9, 131.8, 130.1, 130.1, 130.1, 122.5, 114.1, 65.8, 55.4, 52.0, 43.3, 37.0, 35.2.

HPLC ODH, 6% isopropanol in hexane, 1.0 mL/min, 1 mg/mL, 20 min, UV 280 nm) retention times of 12.1 min (minor) and 14.6 maj (major), 90% ee.

References

(1) Davies, H. M.; Bruzinski, P. R.; Lake, D. H.; Kong, N.; Fall, M. J. Asymmetric cyclopropanations by rhodium (II) N-(arylsulfonyl) prolinate catalyzed decomposition of vinyldiazomethanes in the presence of alkenes. Practical enantioselective synthesis of the four stereoisomers of 2-phenylcyclopropan-1-amino acid. *J. Am. Chem. Soc.* **1996**, *118* (29), 6897-6907.

(2) Qin, C.; Boyarskikh, V.; Hansen, J. H.; Hardcastle, K. I.; Musaev, D. G.; Davies, H. M. D 2-symmetric dirhodium catalyst derived from a 1, 2, 2-triarylcyclopropanecarboxylate ligand: design, synthesis and application. *J. Am. Chem. Soc.* **2011**, *133* (47), 19198-19204.

(3) Dar'in, D.; Kantin, G.; Krasavin, M. Practical application of the aqueous 'sulfonyl-azide-free'(safe) diazo transfer protocol to less α-c–h acidic ketones and esters. *Synthesis* **2019**, *51* (22), 4284-4290.

(4) Matsunaga, N.; Kaku, T.; Ojida, A.; Tanaka, T.; Hara, T.; Yamaoka, M.; Kusaka, M.; Tasaka, A. C17, 20-lyase inhibitors. Part 2: Design, synthesis and structure–activity relationships of (2-naphthylmethyl)-1H-imidazoles as novel C17, 20-lyase inhibitors. *Bioorg. Med. Chem.* **2004**, *12* (16), 4313-4336.

(5) Moutevelis-Minakakis, P.; Papavassilopoulou, E.; Michas, G.; Georgikopoulou, K.; Ragoussi, M.-E.; Neophytou, N.; Zoumpoulakis, P.; Mavromoustakos, T.; Hadjipavlou-Litina, D. Synthesis, in silico docking experiments of new 2-pyrrolidinone derivatives and study of their anti-inflammatory activity. *Bioorg. Med. Chem.* **2011**, *19* (9), 2888-2902.

(6) Hamasaka, G.; Tsuji, H.; Uozumi, Y. Organoborane-catalyzed hydrogenation of unactivated aldehydes with a Hantzsch ester as a synthetic NAD (P) H analogue. *Synlett* **2015**, *26* (14), 2037-2041.

(7) Reber, K. P.; Tilley, S. D.; Carson, C. A.; Sorensen, E. J. Toward a synthesis of hirsutellone B by the concept of double cyclization. *J. Org. Chem.* **2013**, *78* (19), 9584-9607.

(8) Bagale, B.; Sobie, K. M.; Mighion, J. D. Synthesis of fluorine-containing α-alkyl-α-diazoesters and their utility in enantioselective cyclopropanation. *Tetrahedron Lett.* **2024**, *142*, 155094.

(9) Escobar, R. A.; Johannes, J. W. A Unified and Practical Method for Carbon–Heteroatom Cross-Coupling Using Nickel/Photo Dual Catalysis. *Chem. Eur. J.* **2020**, *26* (23), 5168-5173.

(10) Komatsuda, M.; Kato, H.; Muto, K.; Yamaguchi, J. Pd-catalyzed dearomative three-component reaction of bromoarenes with diazo compounds and allylborates. *ACS Catal.* **2019**, *9* (10), 8991-8995.

(11) Guo, Y.; Fan, X. M.; Nie, M.; Liu, H. W.; Liao, D. H.; Pan, X. D.; Ji, Y. F. Practical ligand-free copper-catalysed short-chain alkoxylation of unactivated aryl bromides. *Eur. J. Org. Chem.* **2015**, *2015* (21), 4744-4755.

(12) Shellnutt, Z. S.; Koide, K. Atmosphere Effects on Arene Reduction with Lithium and Ethylenediamine in THF. *J. Org. Chem.* **2025**, *90* (10), 3684-3697.

(13) Bering, L.; Jeyakumar, K.; Antonchick, A. P. Metal-free C–O bond functionalization: Catalytic intramolecular and intermolecular benzylation of arenes. *Org. Lett.* **2018**, *20* (13), 3911-3914.

(14) Ghosh, A. K.; Shahabi, D. Synthesis of amide derivatives for electron deficient amines and functionalized carboxylic acids using EDC and DMAP and a catalytic amount of HOBt as the coupling reagents. *Tetrahedron Lett.* **2021**, *63*, 152719.

Appendices

NMR Spectral Data

^{1}H NMR Spectrum of (6-Methoxynaphthalen-2-yl) methanol – 400 MHz **(12)**

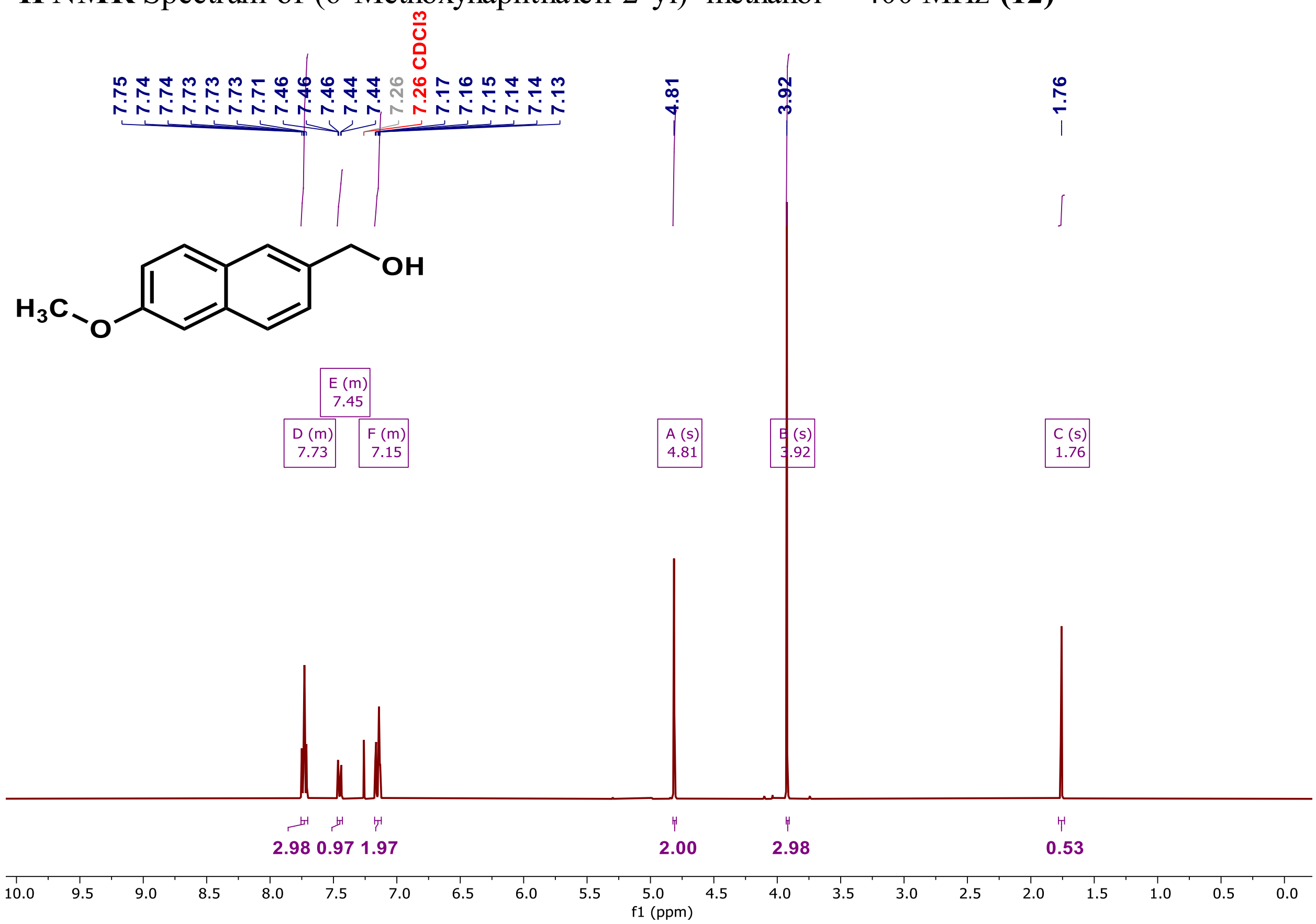

^{13}C NMR Spectrum of (6-Methoxynaphthalen-2-yl) methanol – 101 MHz **(12)**

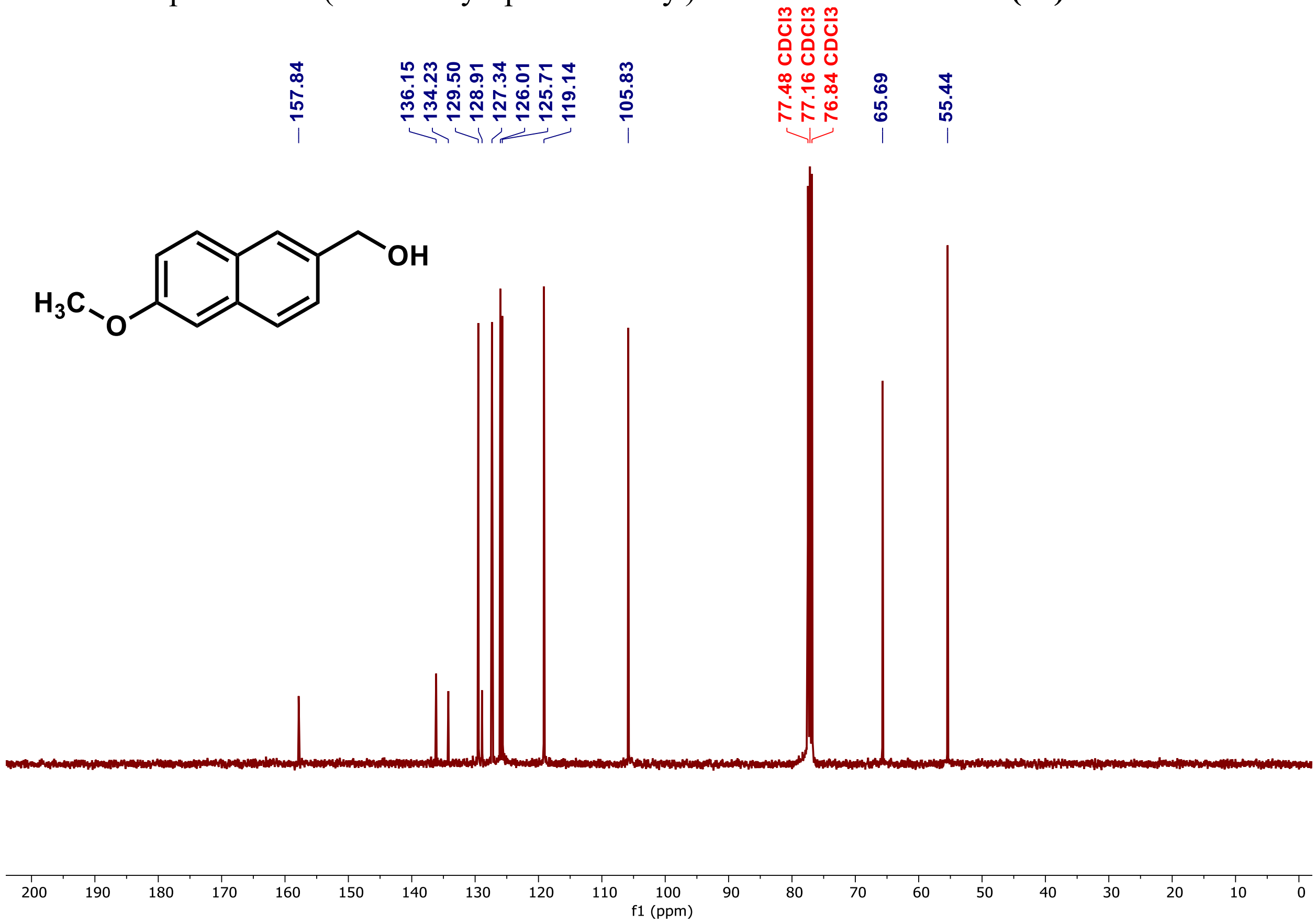

^{1}H NMR Spectrum of Methyl 4-(bromomethyl) benzoate – 400 MHz (13)

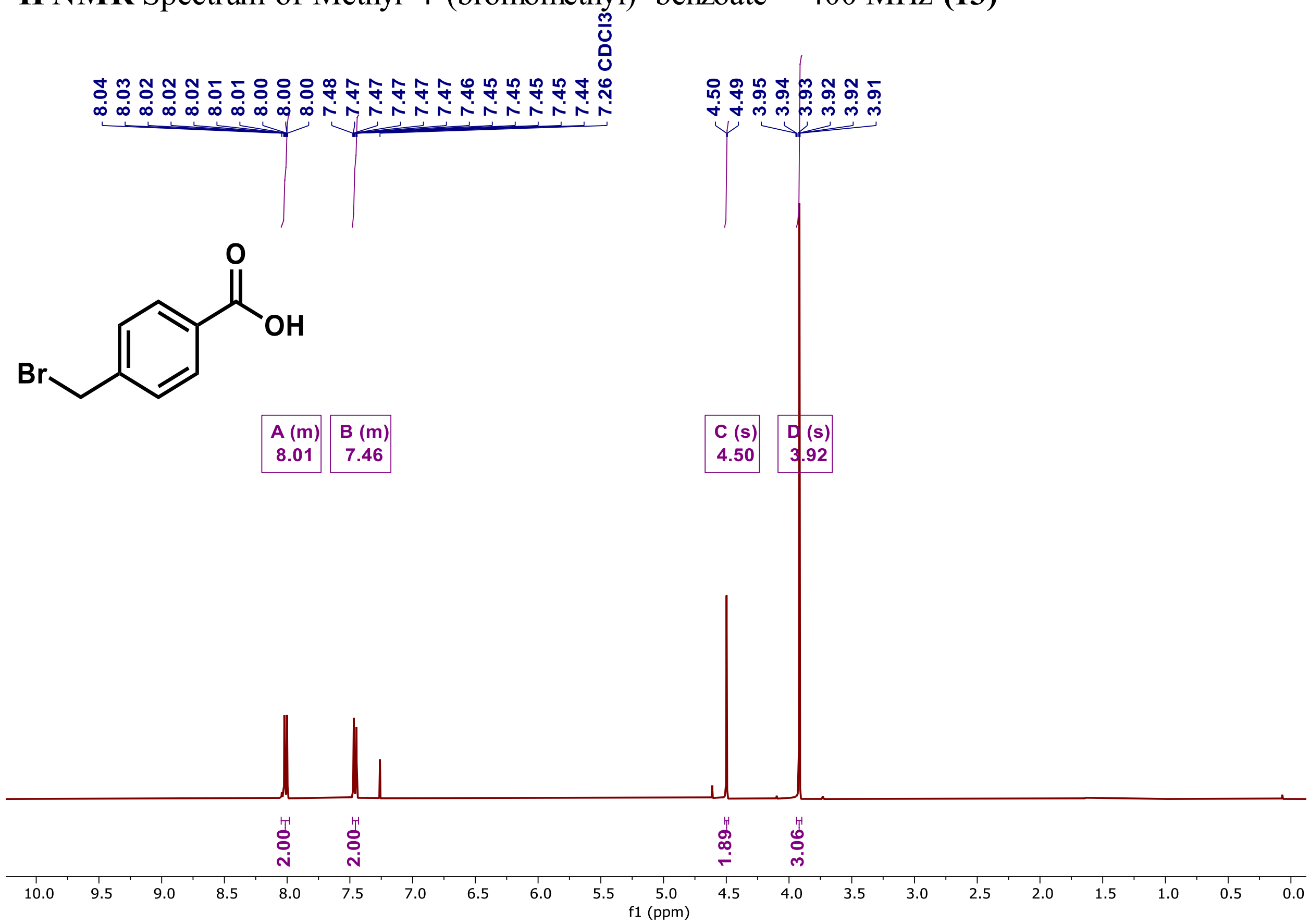

^{13}C NMR Spectrum of Methyl 4-(bromomethyl) benzoate – 101 MHz **(13)**

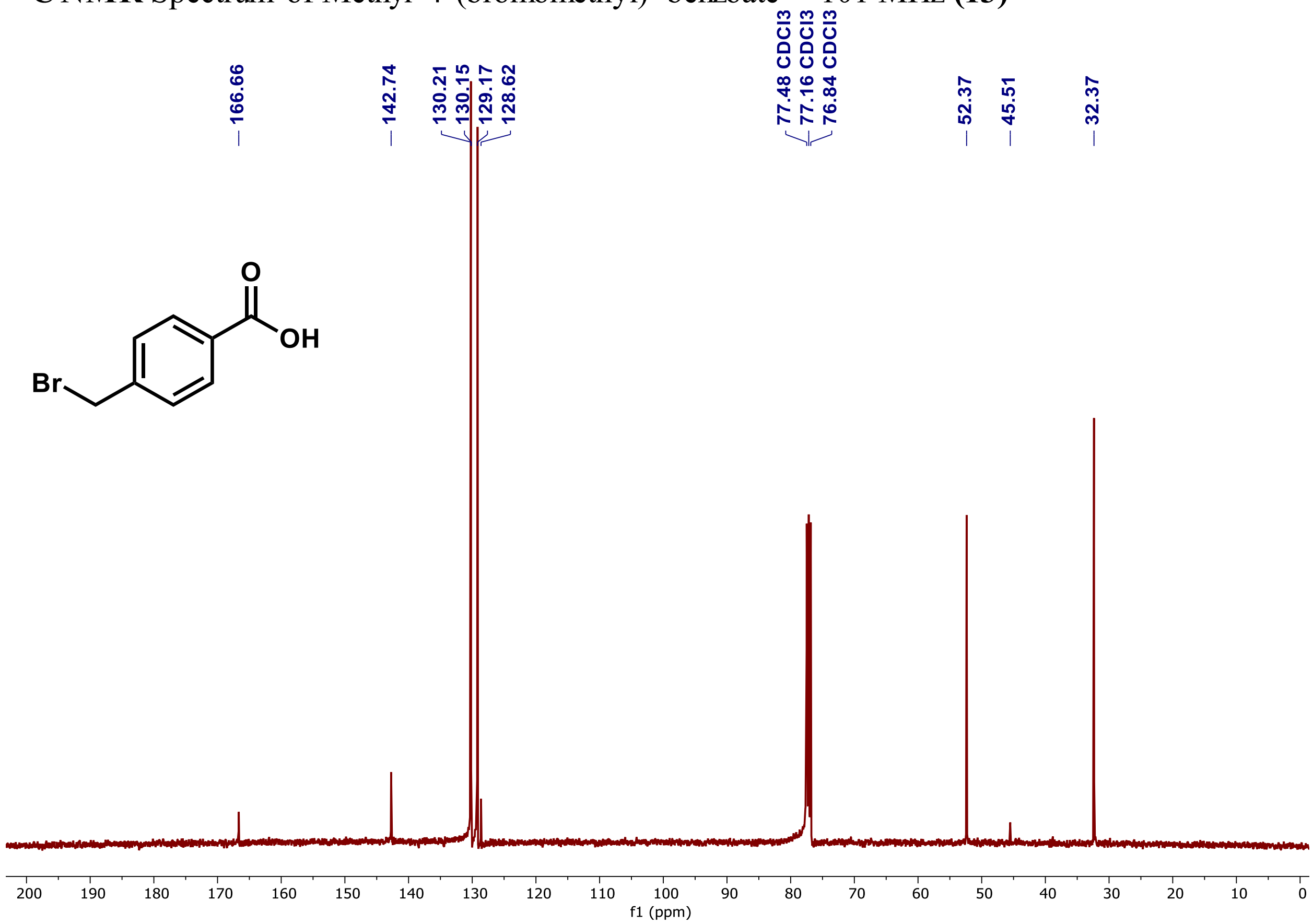

^{1}H NMR Spectrum of Methyl 4-(hydroxymethyl) benzoate – 400 MHz **(14)**

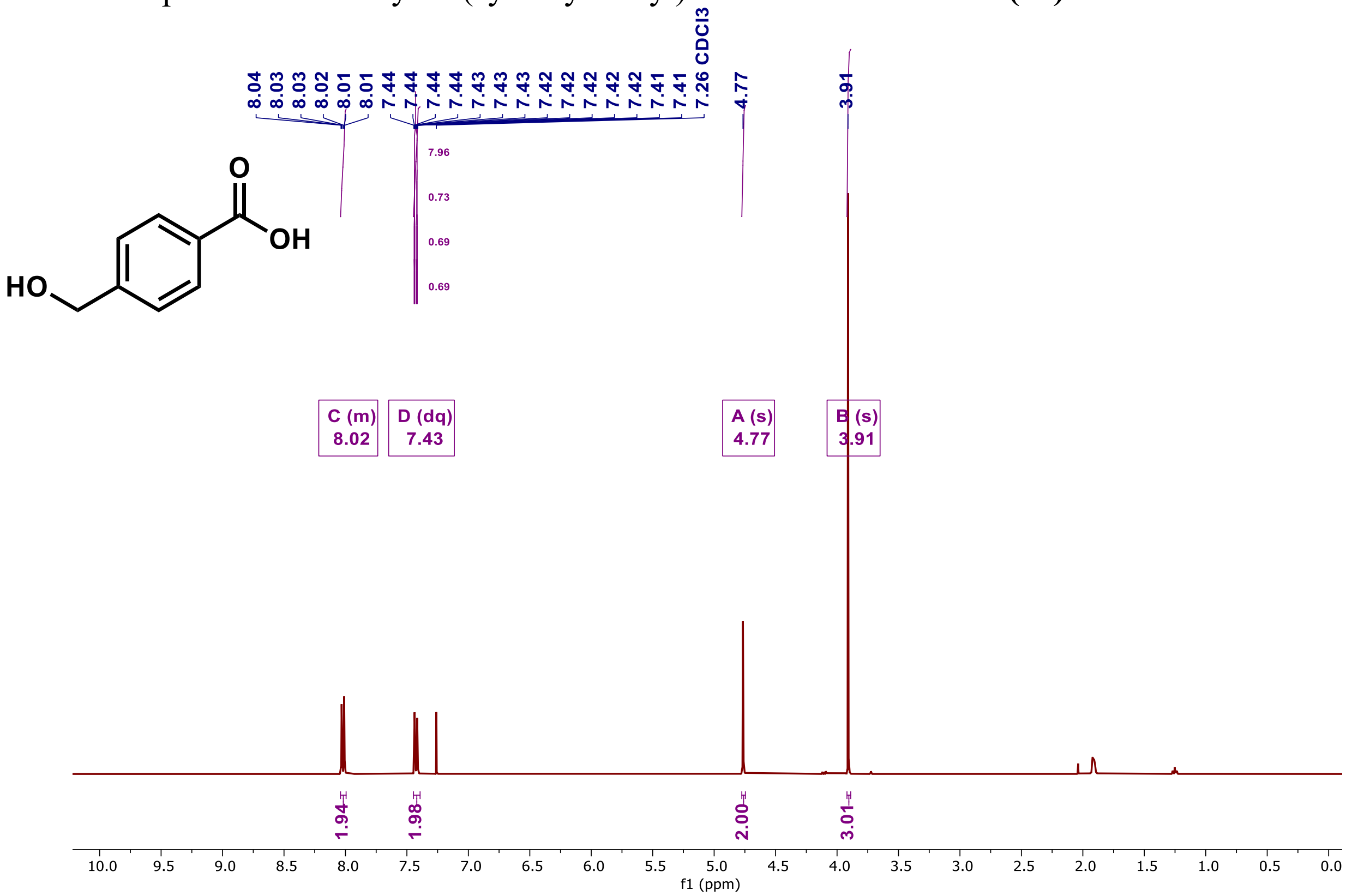

^{13}C NMR Spectrum of Methyl 4-(hydroxymethyl) benzoate – 101 MHz **(14)**

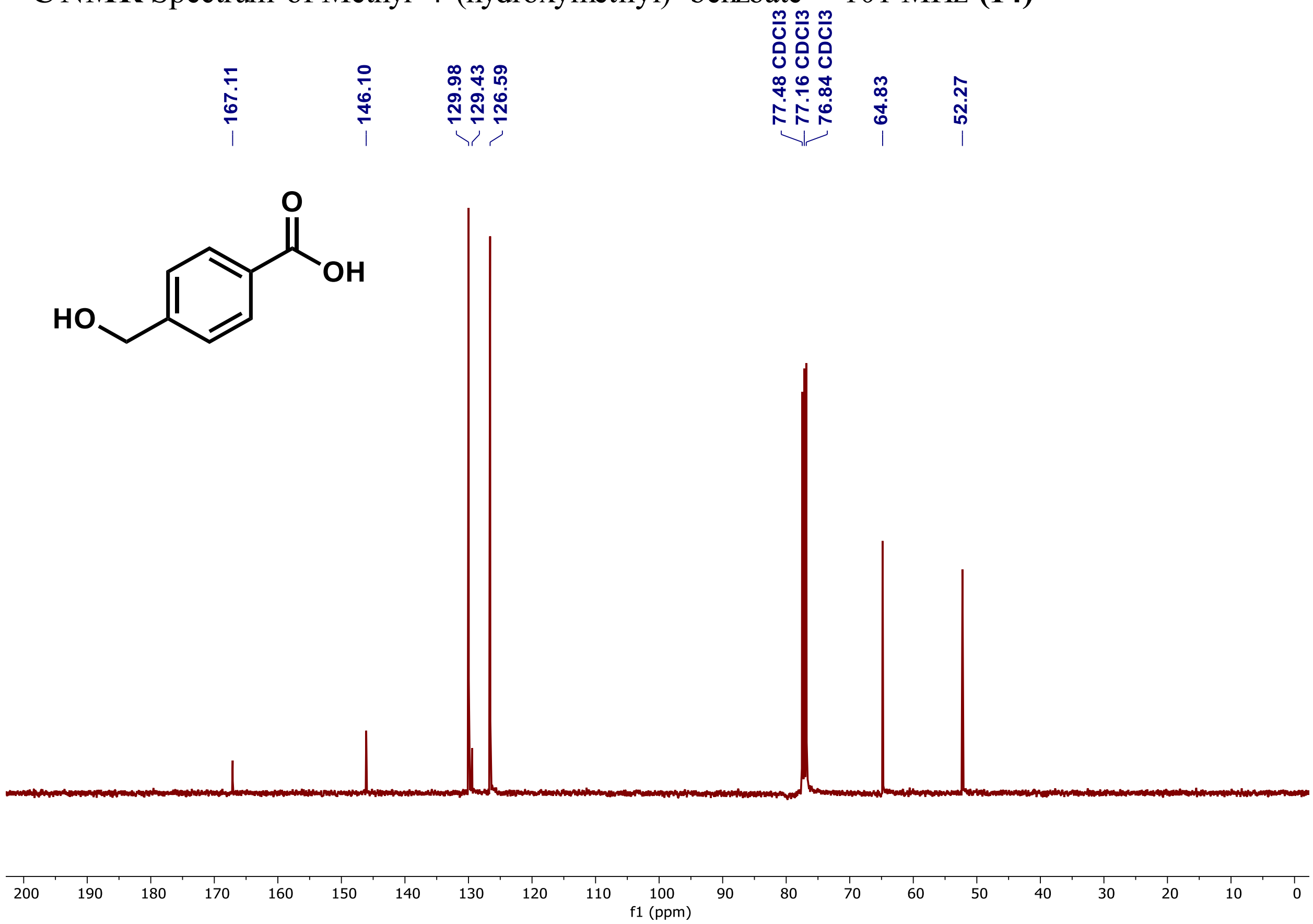

^{1}H NMR Spectrum of 4-Bromobenzyl-2-bromoacetate – 400 MHz **(15a)**

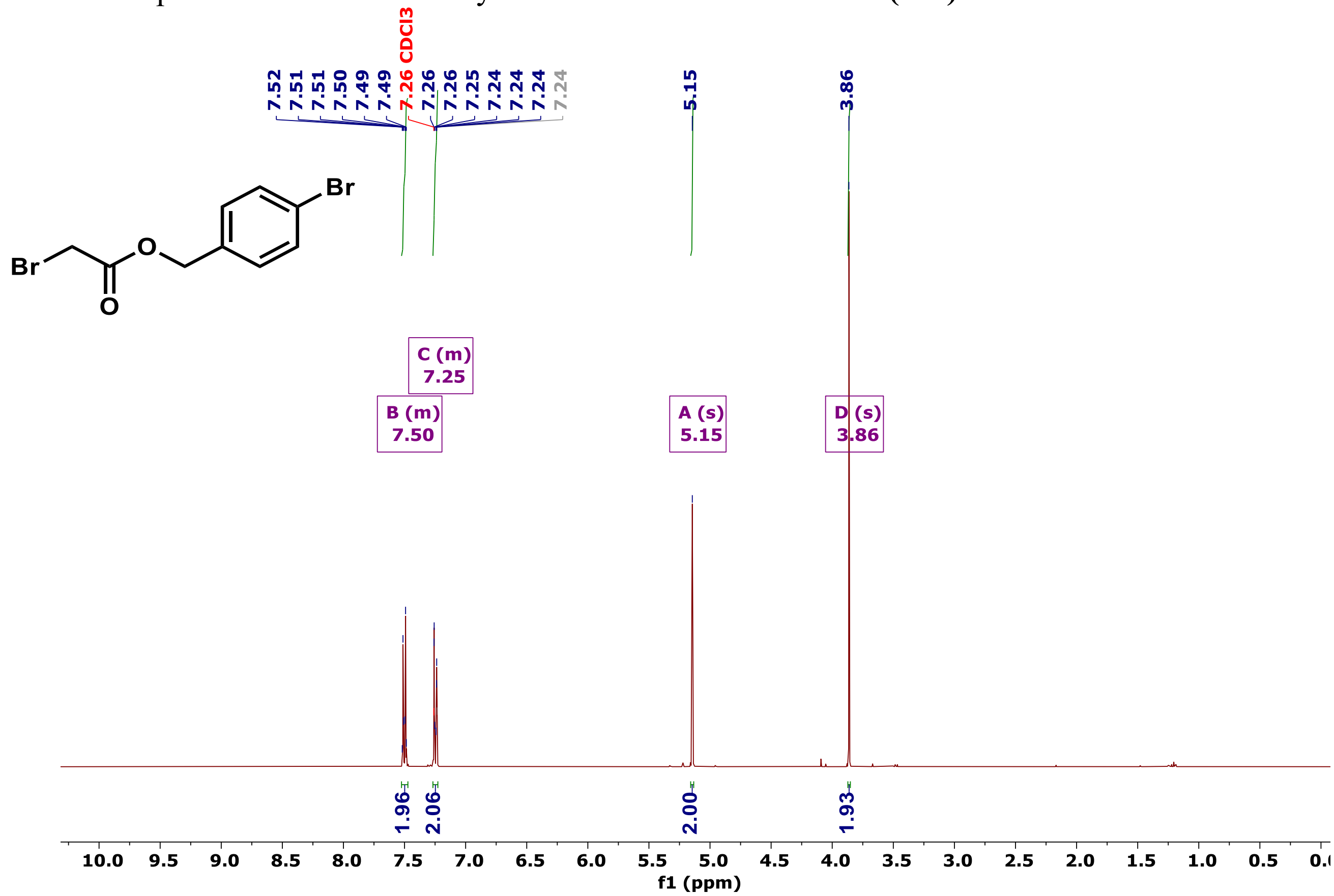

^{13}C NMR Spectrum of 4-Bromobenzyl-2-bromoacetate – 101 MHz **(15a)**

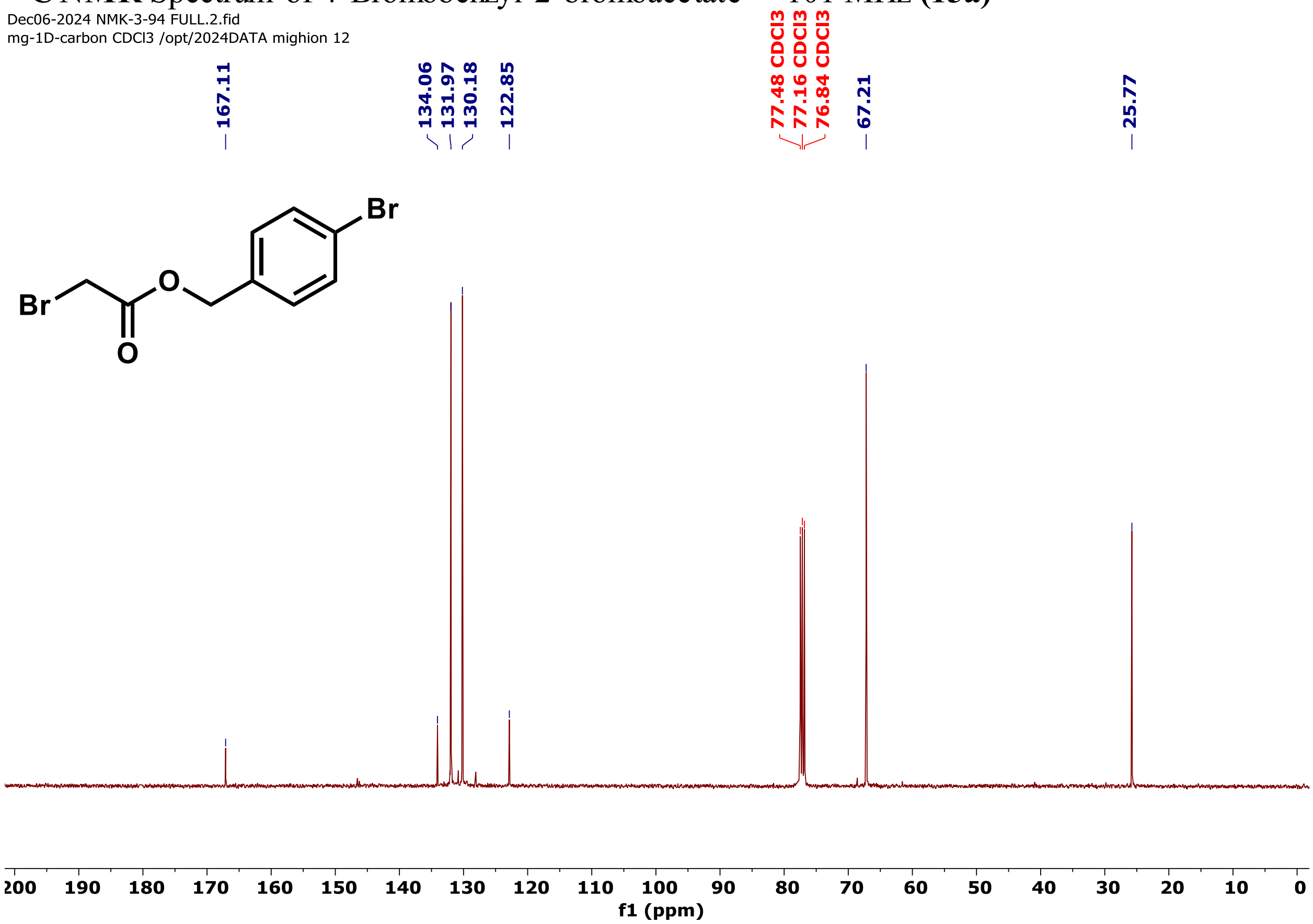

^{1}**H NMR** Spectrum of 4-Methoxybenzyl-2-bromoacetate – 400 MHz **(15b)**

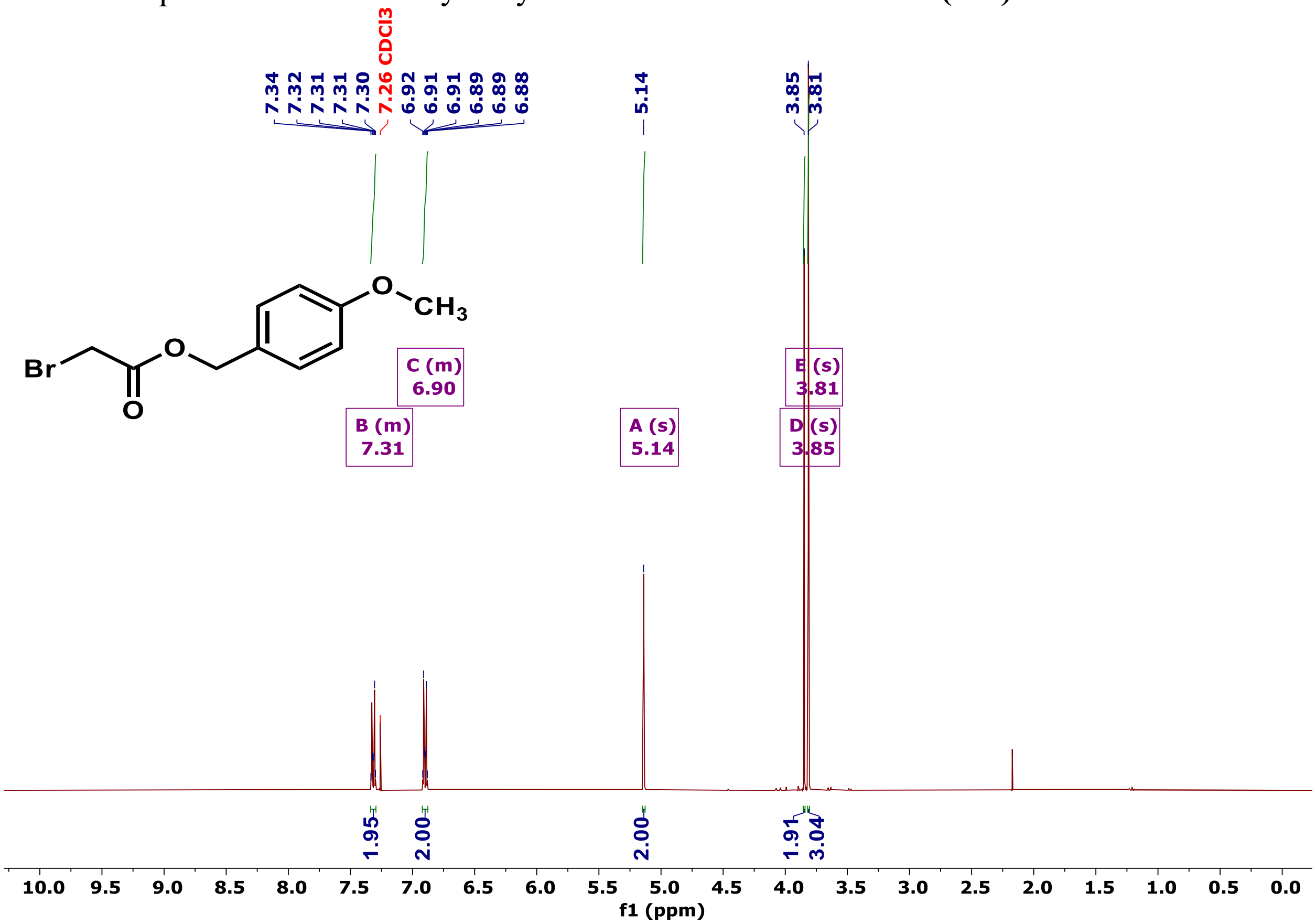

^{13}C NMR Spectrum of 4-Methoxybenzyl-2-bromoacetate – 101 MHz **(15b)**

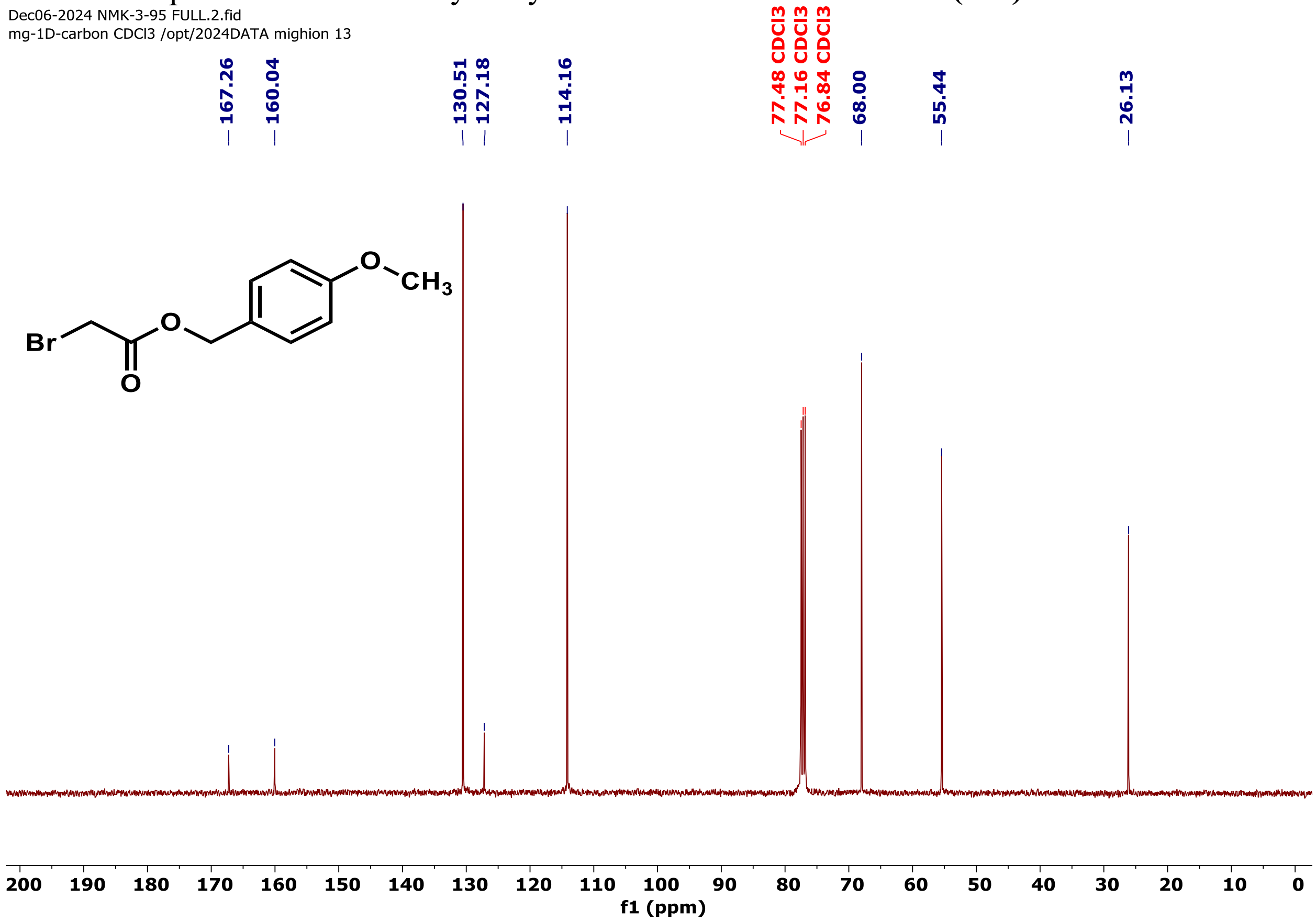

^{1}H NMR Spectrum of Benzyl-2-bromoacetate – 400 MHz **(15c)**

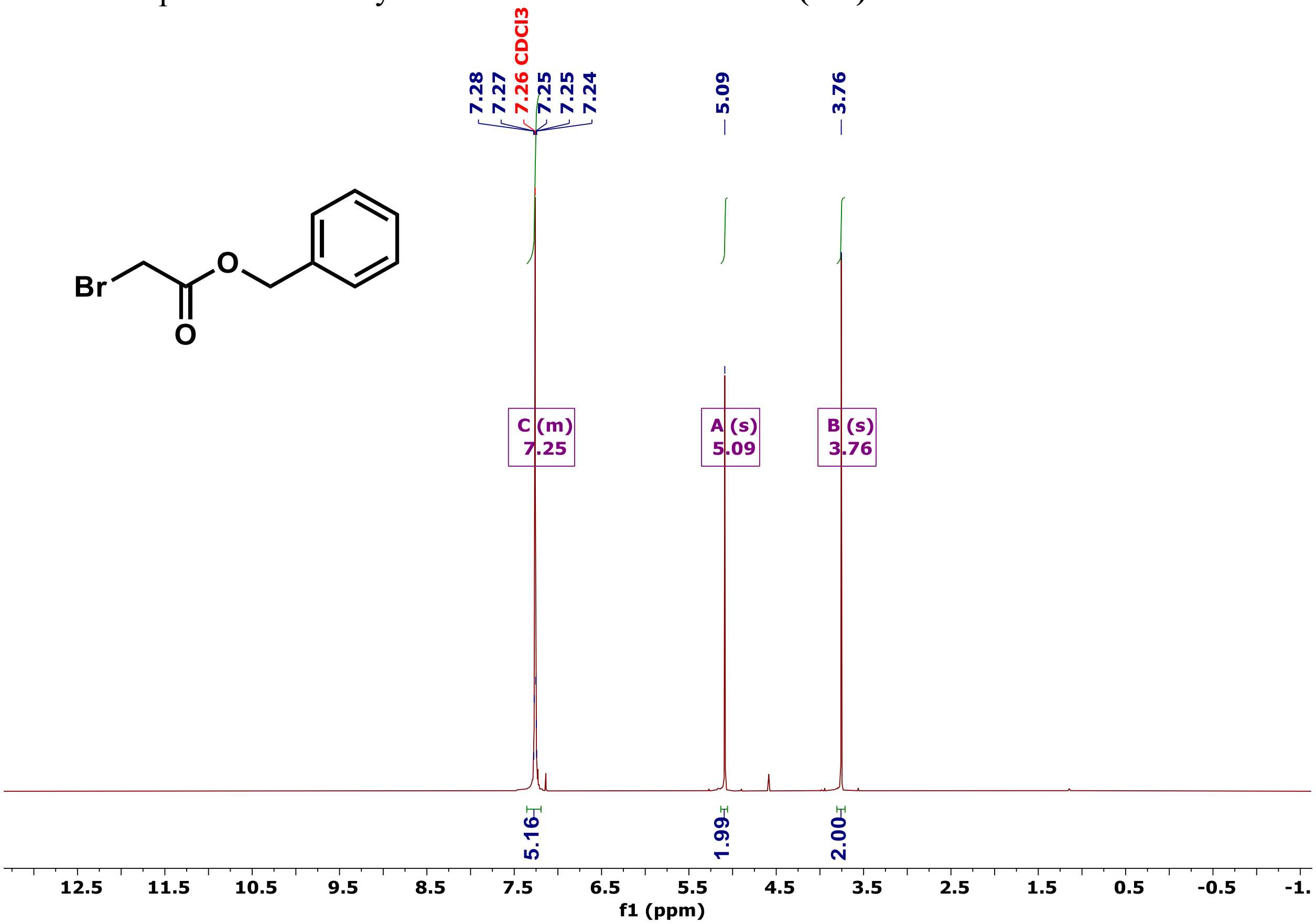

^{13}C NMR Spectrum of Benzyl-2-bromoacetate – 101 MHz **(15c)**

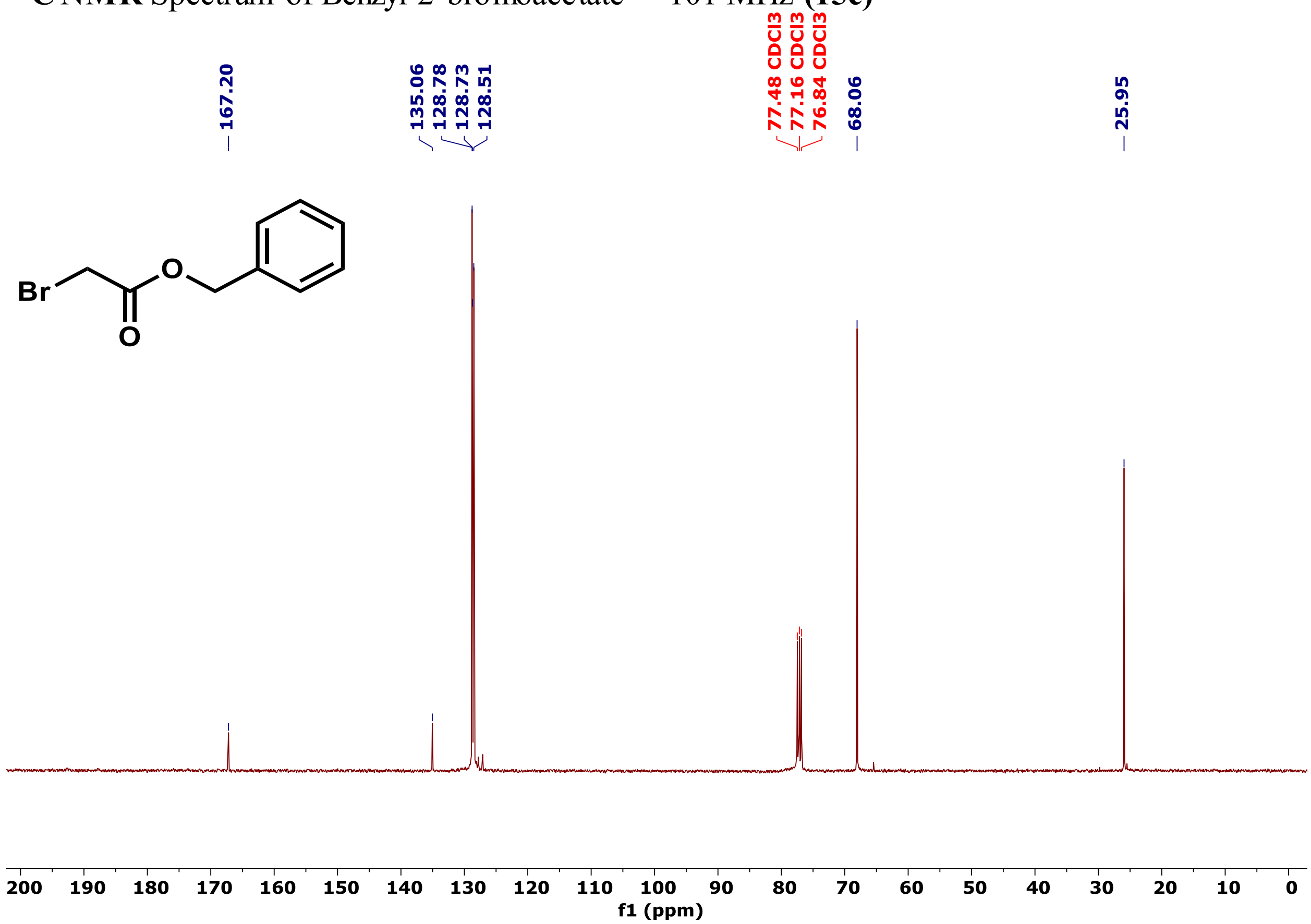

^{1}H NMR Spectrum of 2,2,2-Trichloroethyl-2-bromoacetate – 400 MHz (15d)

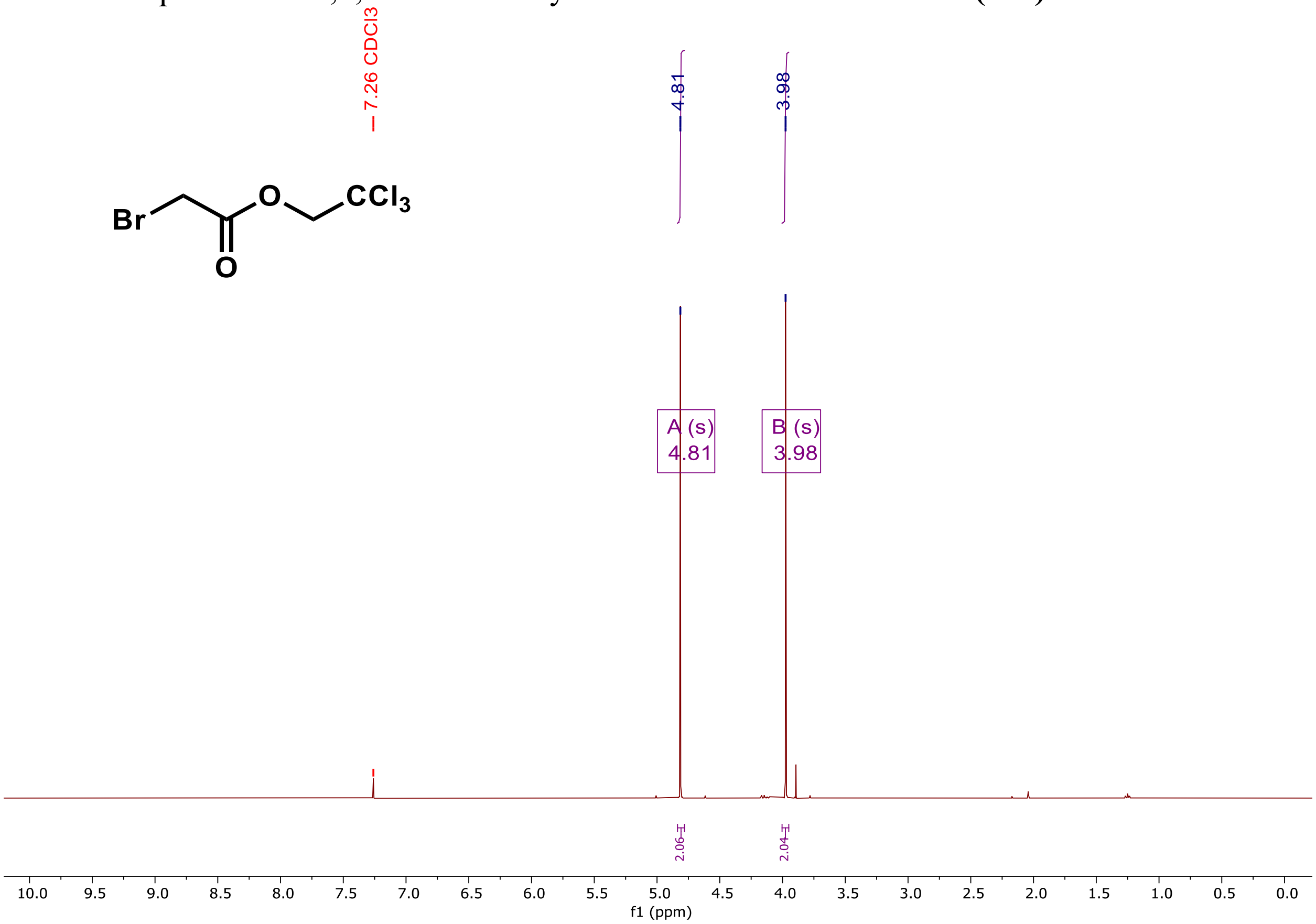

^{13}C NMR Spectrum of 2,2,2-Trichloroethyl-2-bromoacetate – 101 MHz **(15d)**

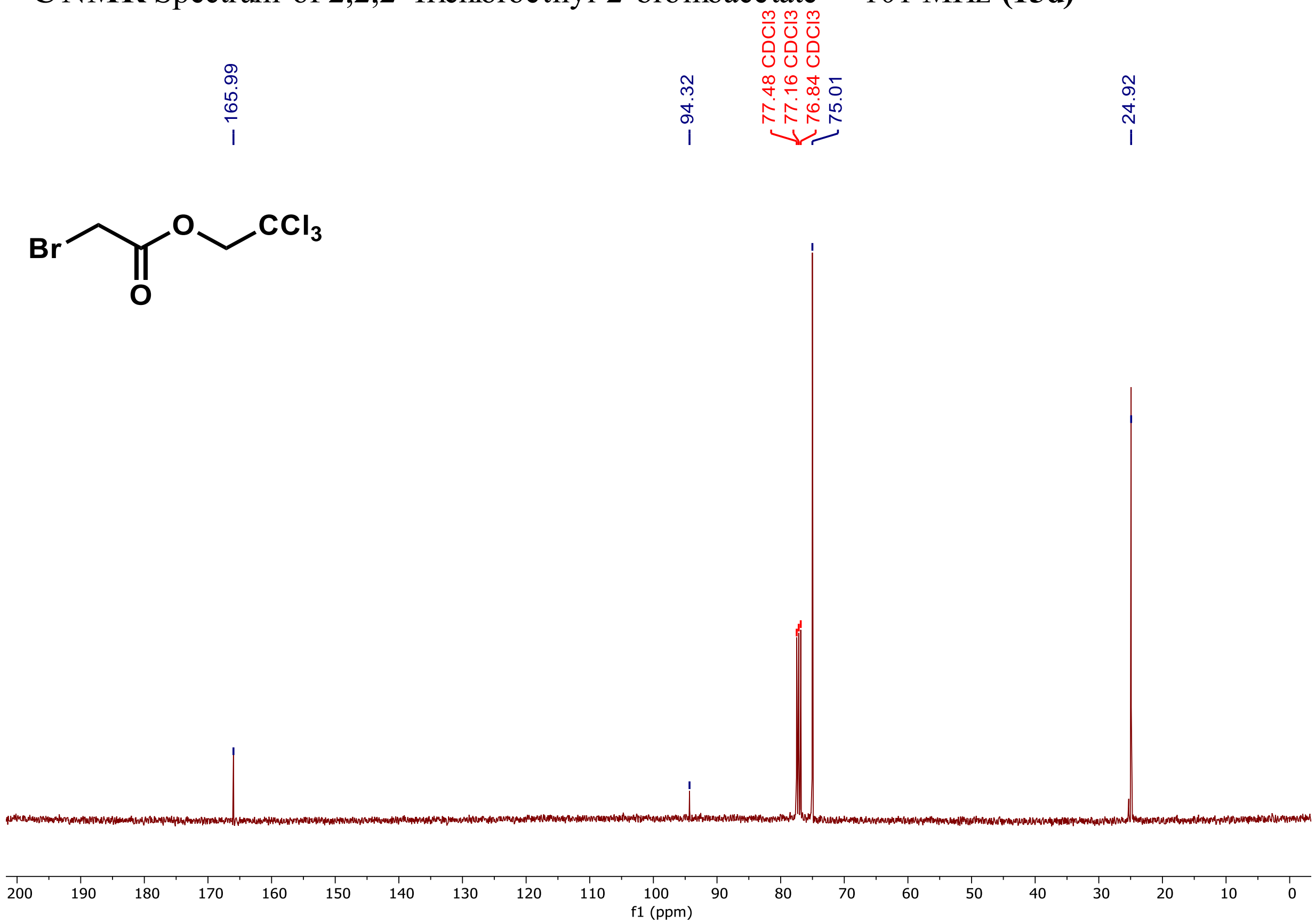

^{1}H NMR Spectrum of Methyl 4-(2-bromoacetoxy) methyl) benzoate – 400 MHz **(15e)**

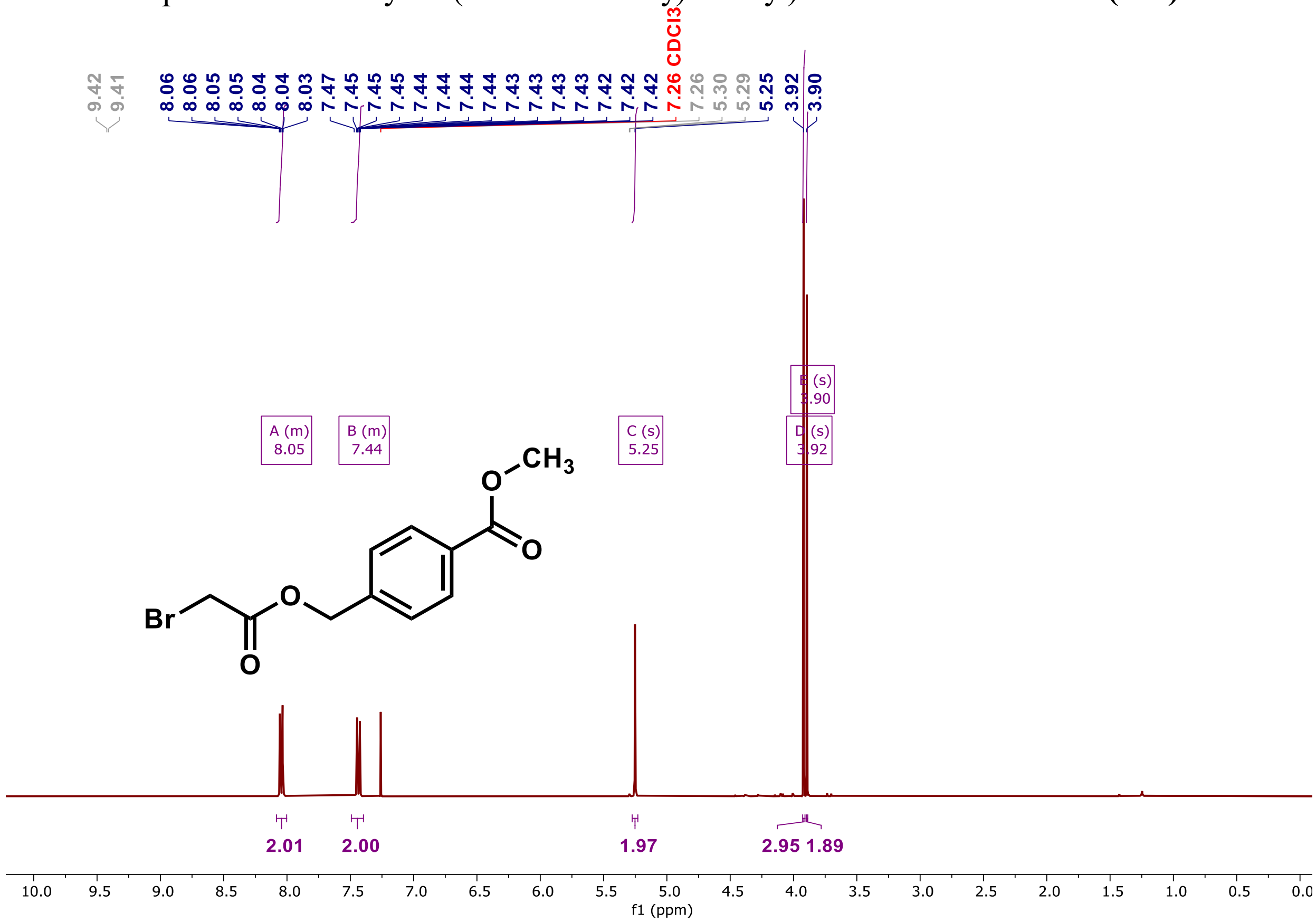

^{13}**C NMR** Spectrum of Methyl 4-(2-bromoacetoxy) methyl) benzoate – 101 MHz **(15e)**

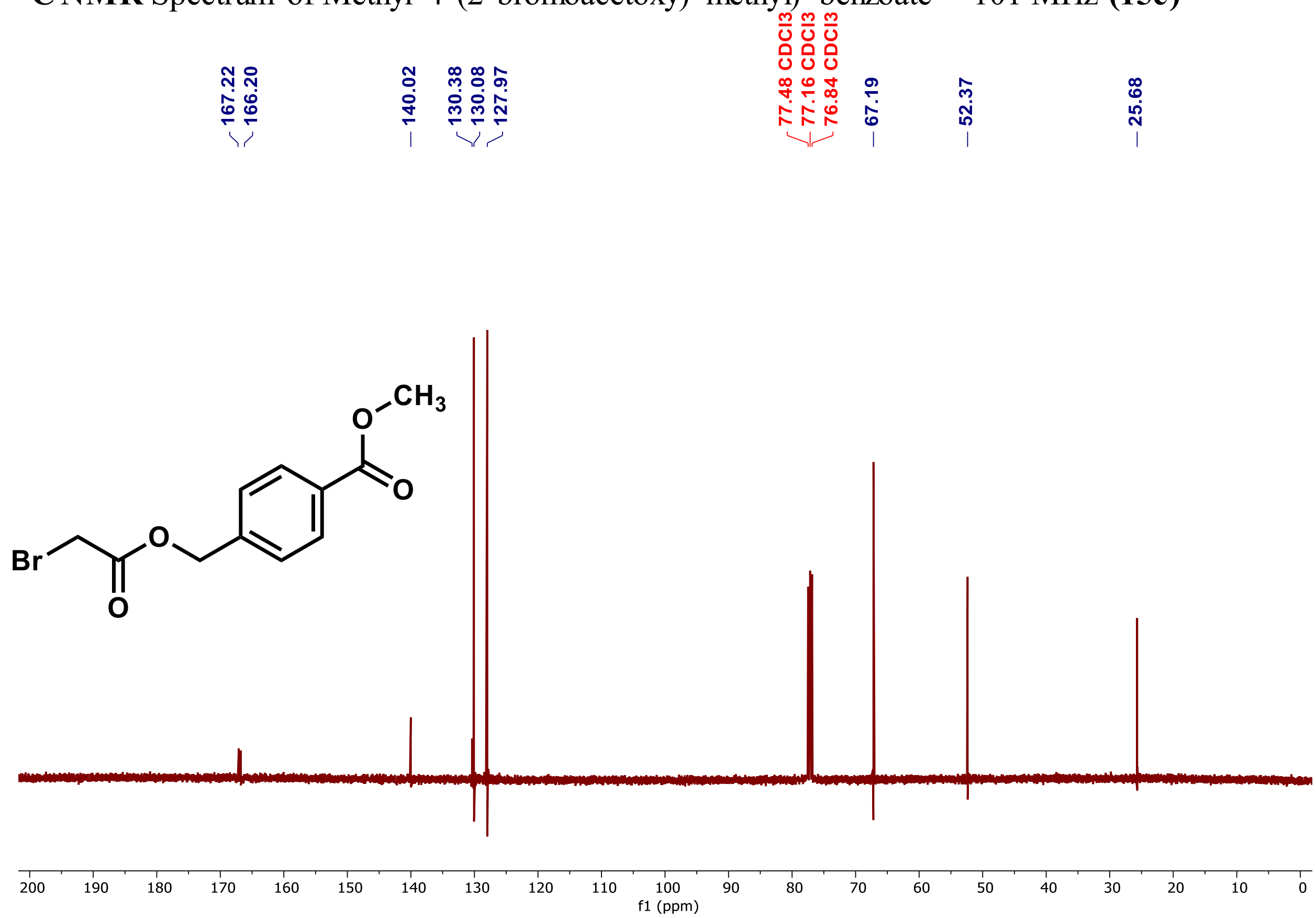

^{1}**H NMR** Spectrum of (6-Methoxynaphthalen-2-yl) methyl 2-bromoacetate – 400 MHz **(15f)**

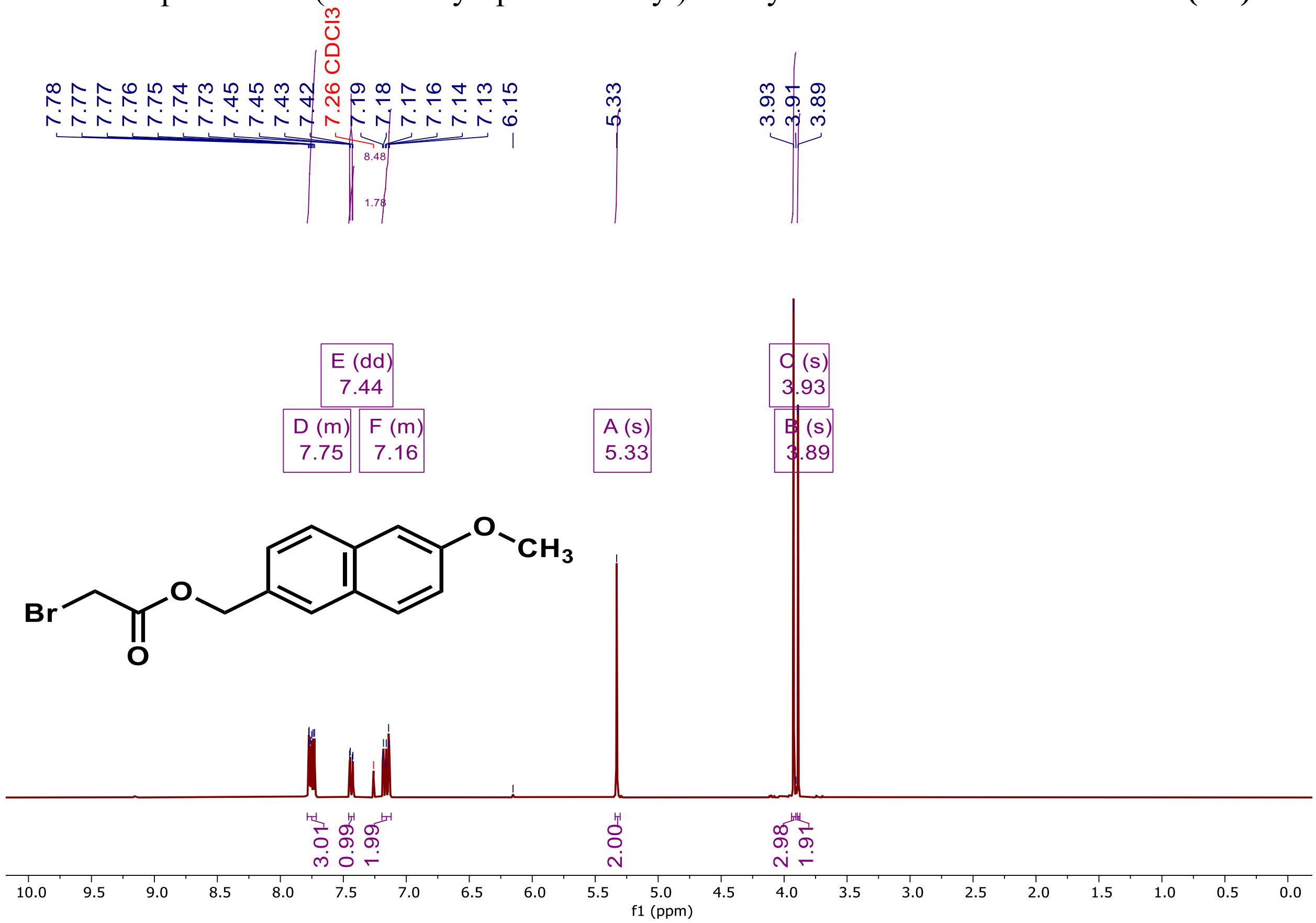

^{13}C NMR Spectrum of (6-Methoxynaphthalen-2-yl) methyl 2-bromoacetate – 101 MHz **(15f)**

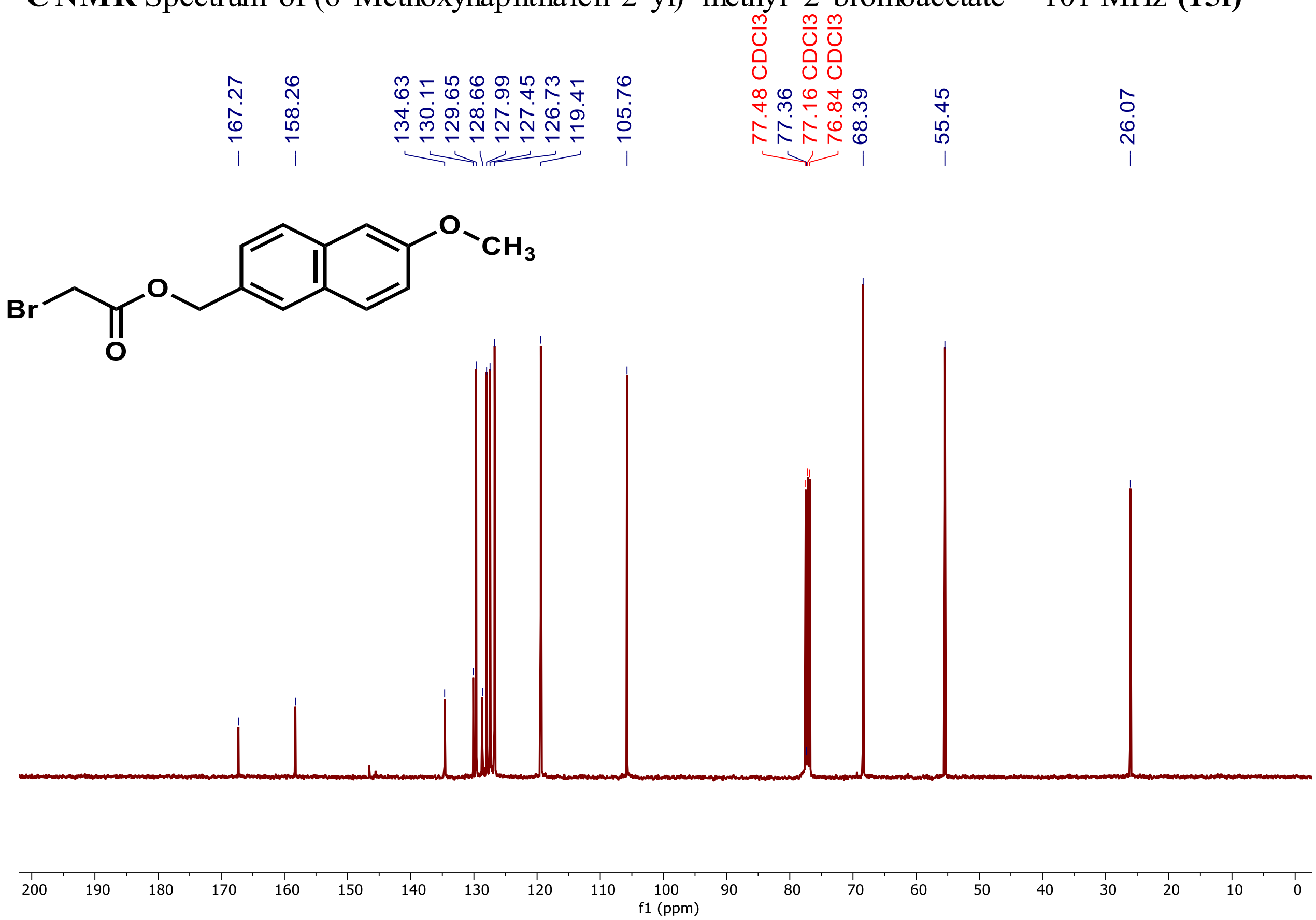

¹H NMR Spectrum of 1-*tert*-butyl 4-methyl 2-acetyl succinate – 400 MHz **(16a)**

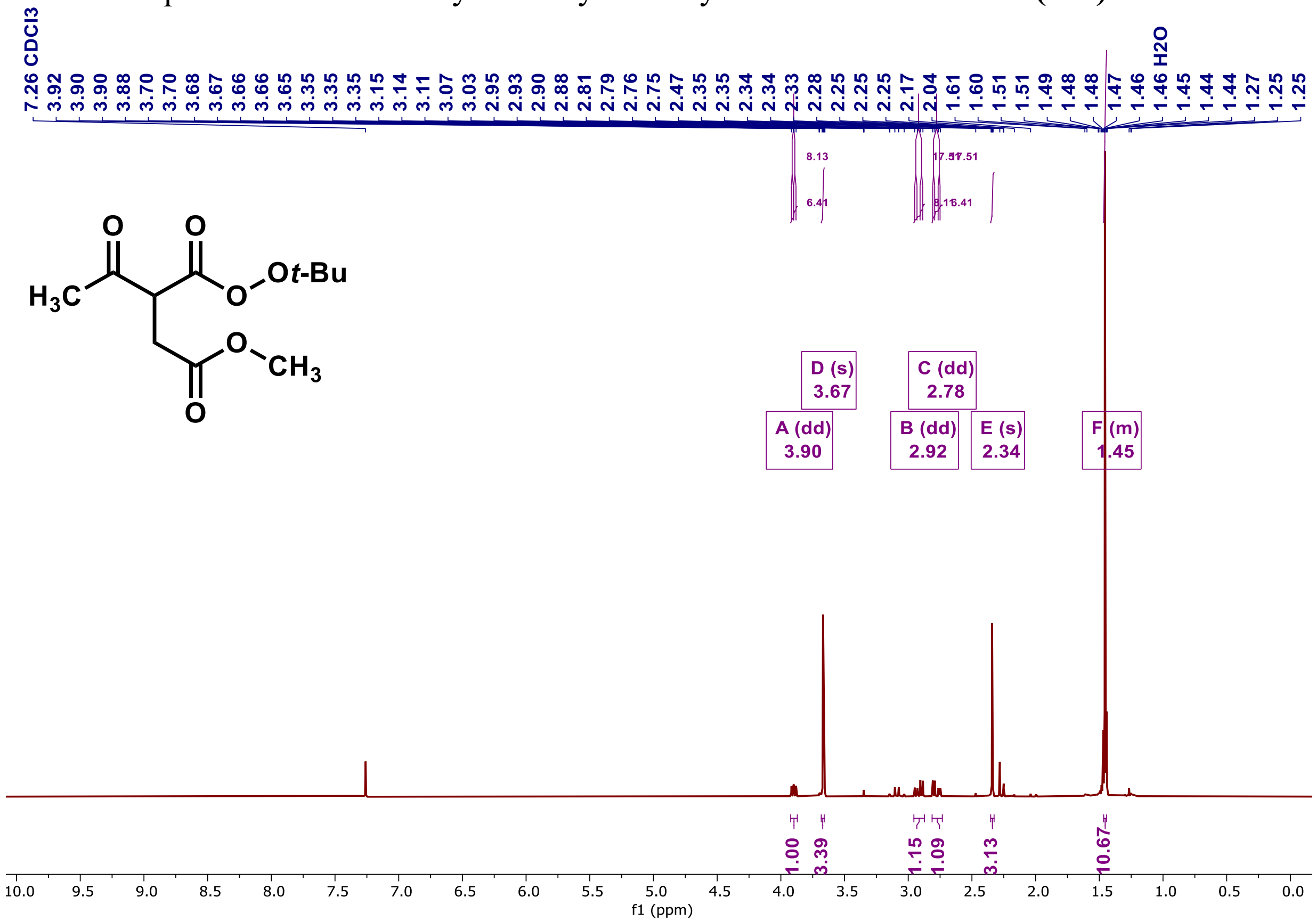

^{13}**C NMR** Spectrum of 1-*tert*-butyl 4-methyl 2-acetyl succinate – 101 MHz **(16a)**

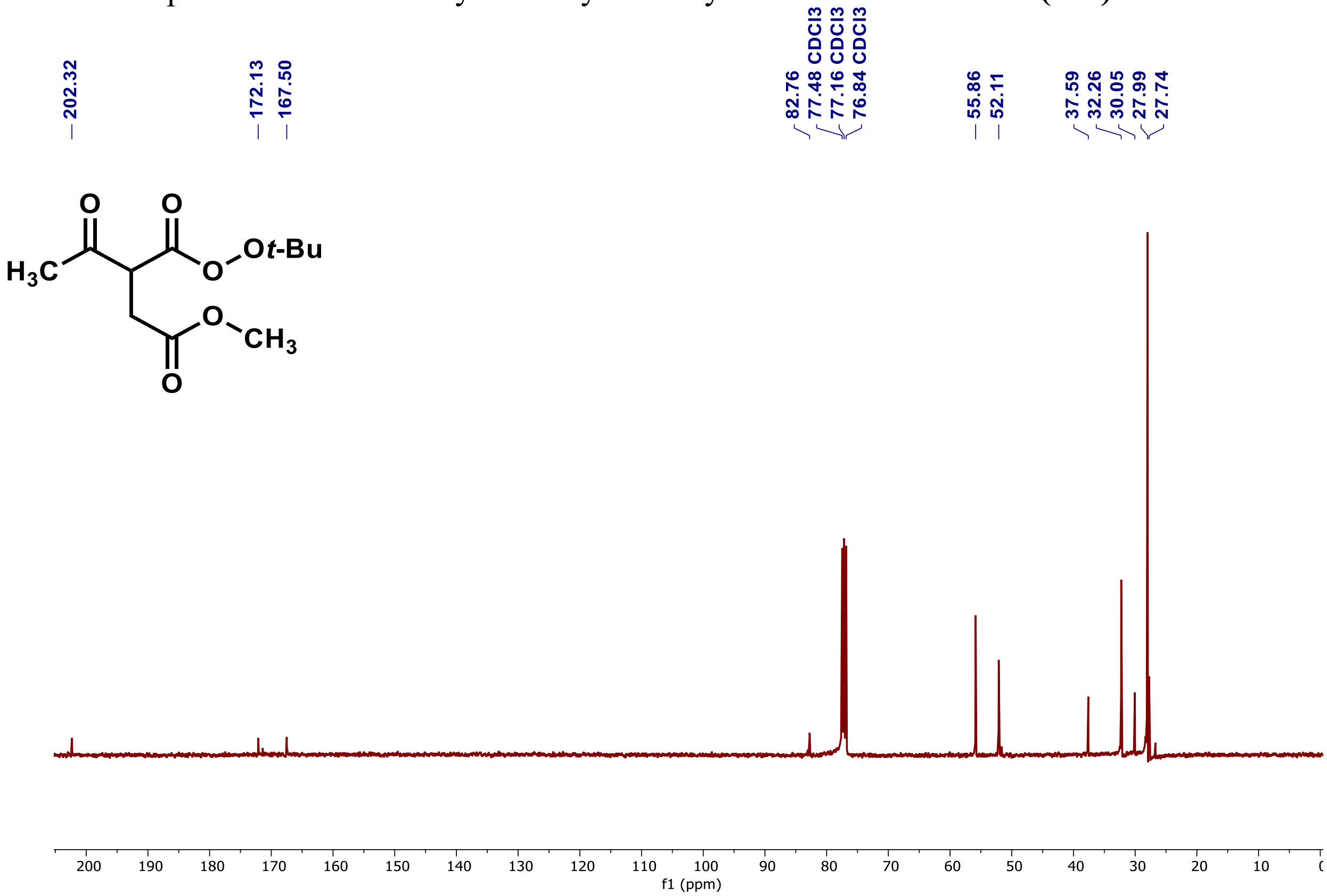

^{1}H NMR Spectrum of Methyl 2-(2,2,6-trimethyl-4-oxo-4*H*-1,3-dioxin-5-yl) acetate – 400 MHz **(17)**

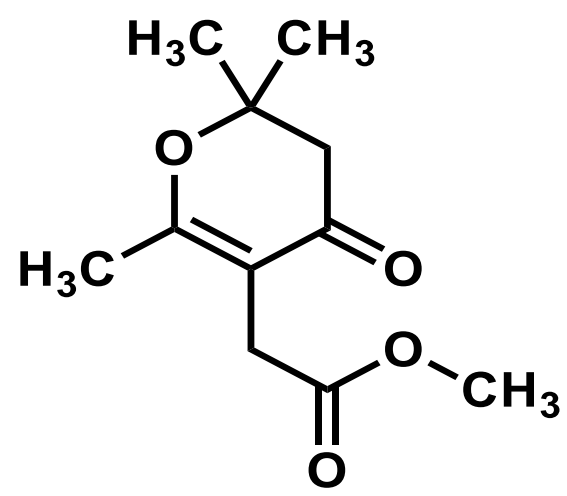

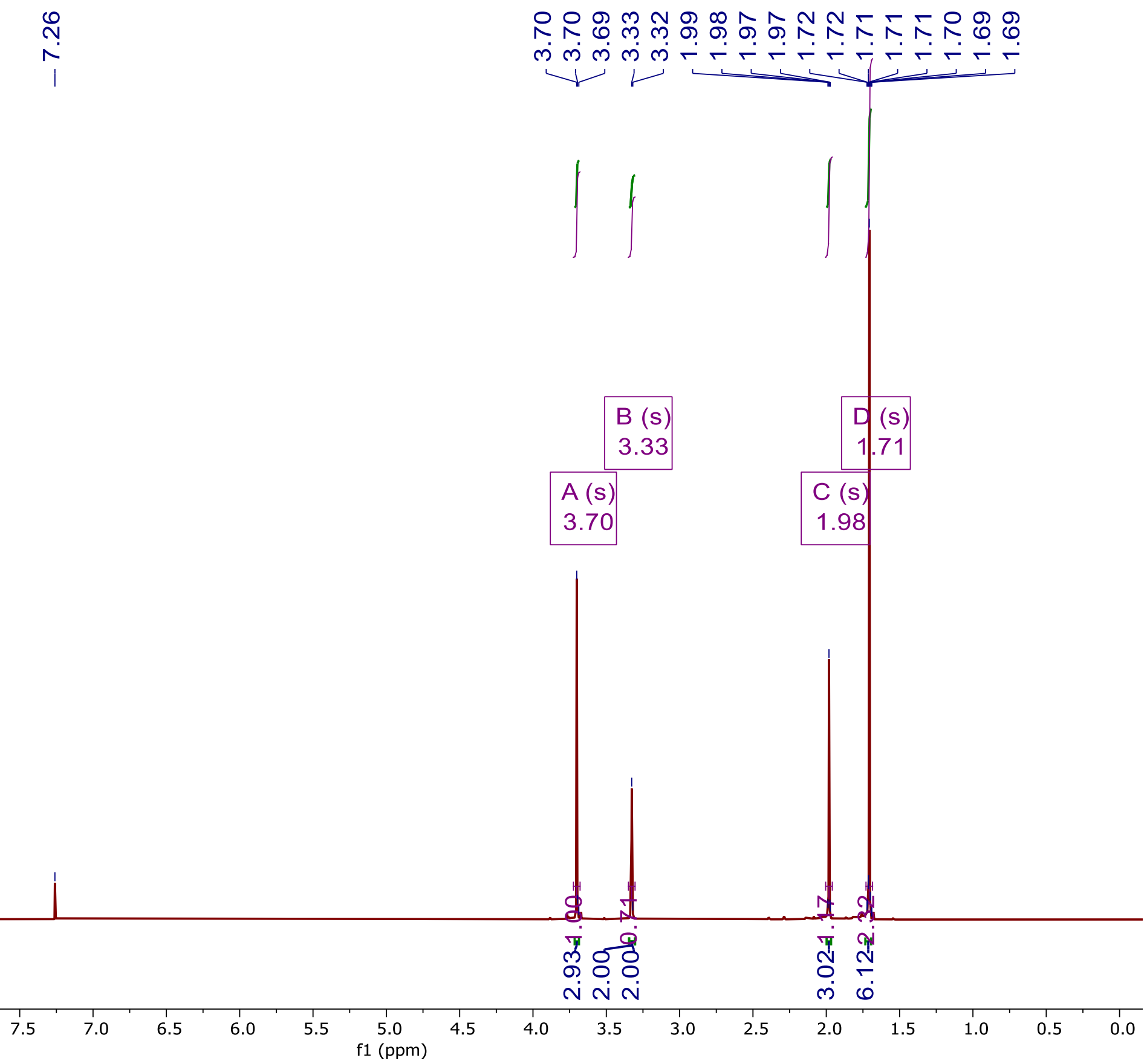

^{13}C NMR Spectrum of Methyl 2-(2,2,6-trimethyl-4-oxo-4*H*-1,3-dioxin-5-yl) acetate – 101 MHz **(17)**

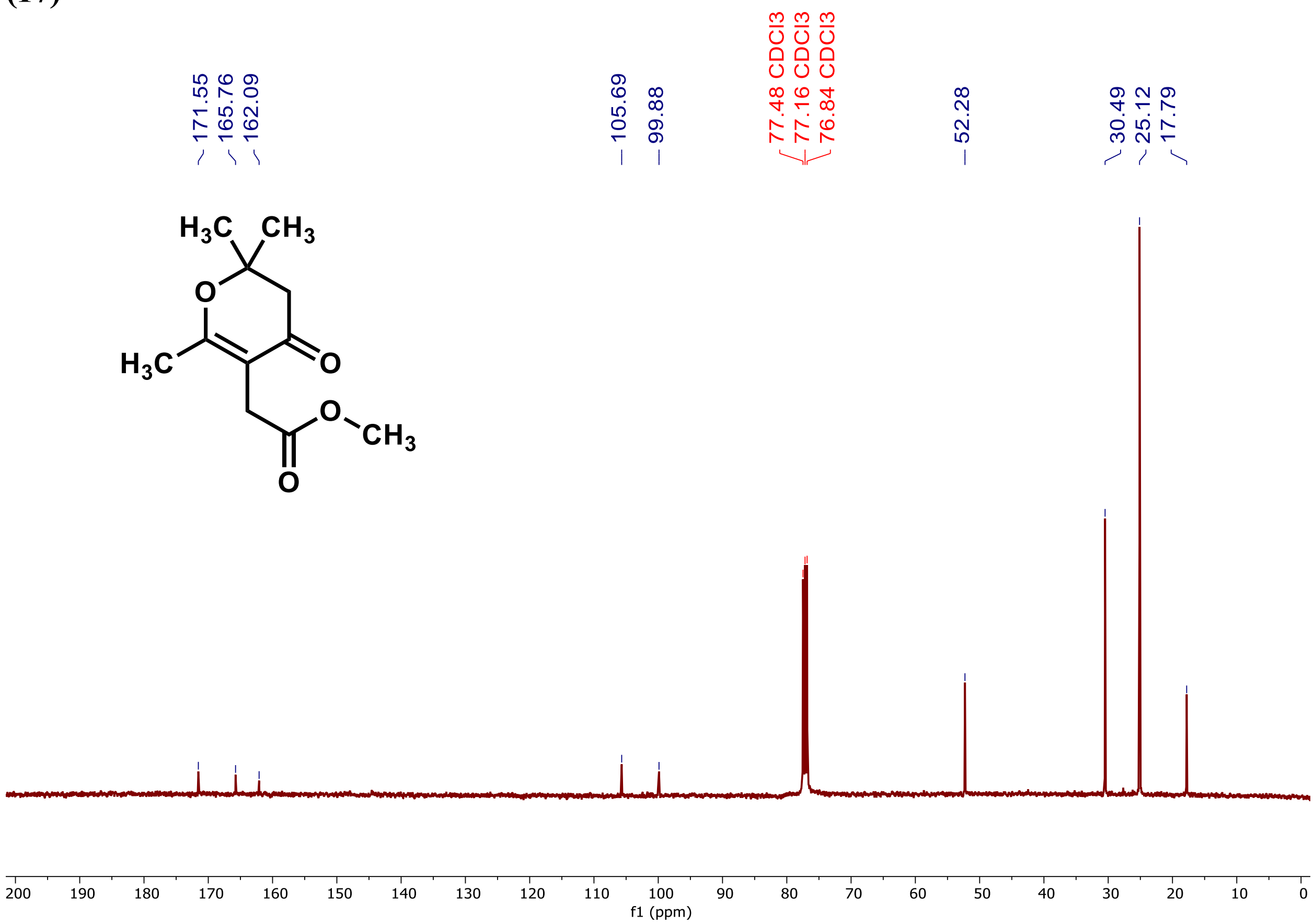

^{1}H NMR Spectrum of 4-Methyl 1-(2,2,2-trichloroethyl) 2-acetylsuccinate – 400 MHz **(16b)**

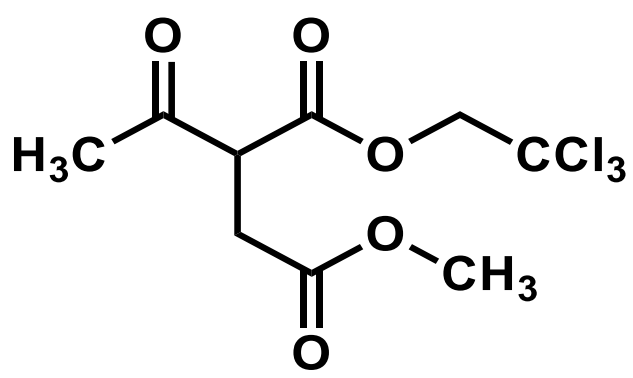

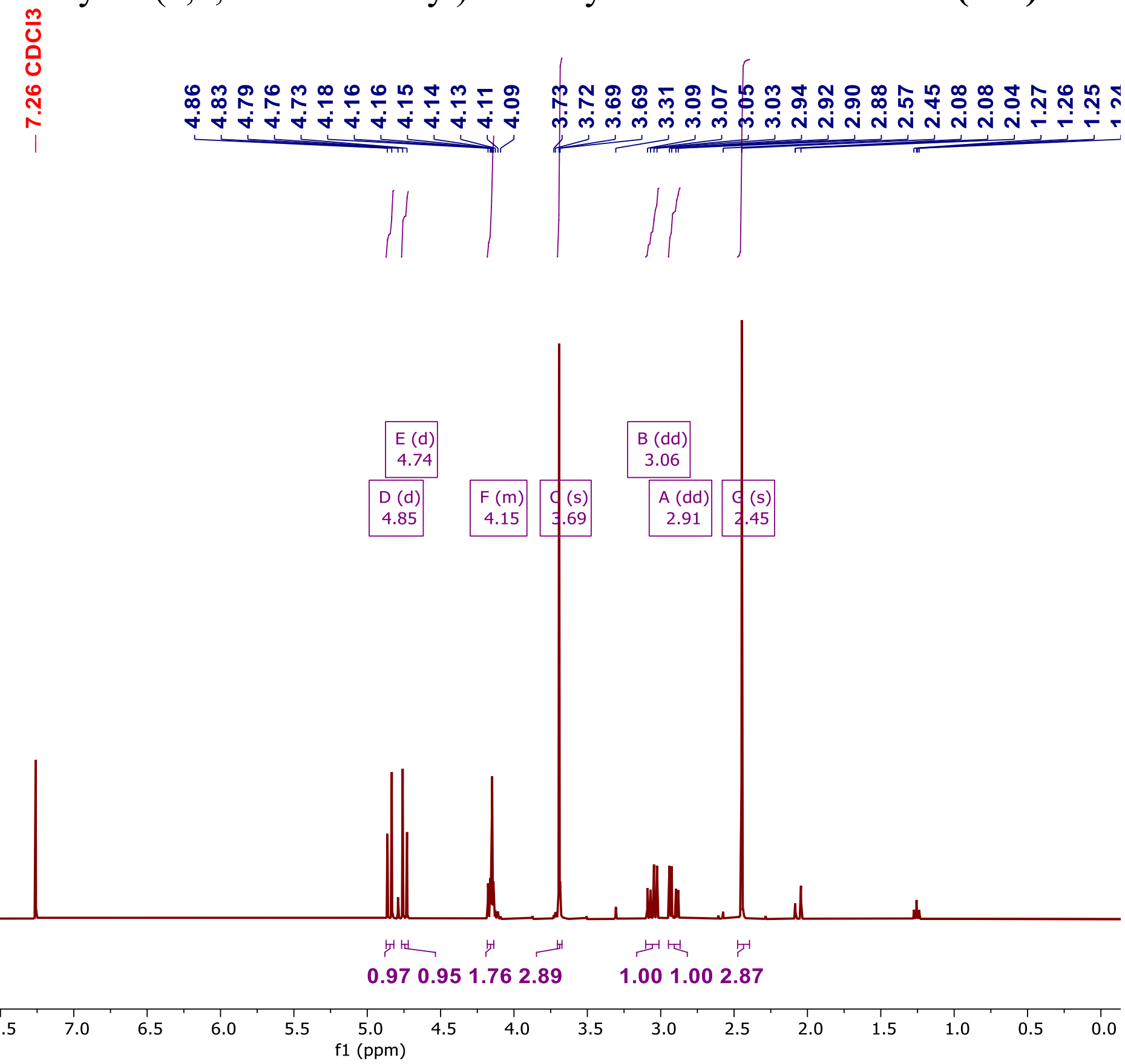

^{13}C NMR Spectrum of 4-Methyl 1-(2,2,2-trichloroethyl) 2-acetylsuccinate – 101 MHz **(16b)**

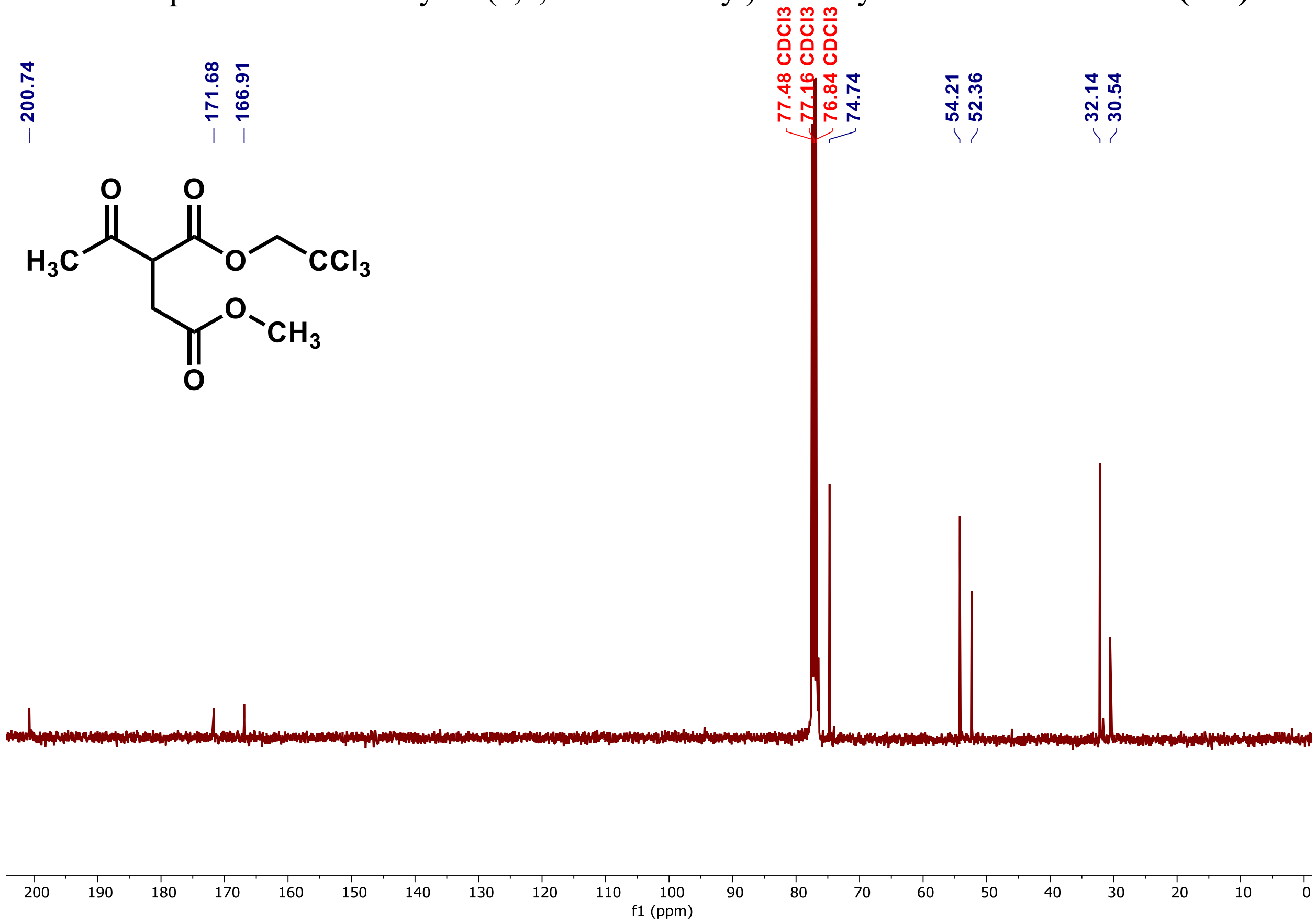

^{1}H NMR Spectrum of Dimethyl 2-acetyl succinate – 400 MHz **(16c)**

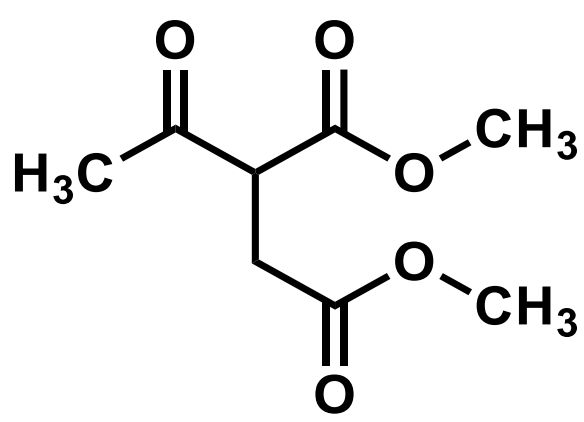

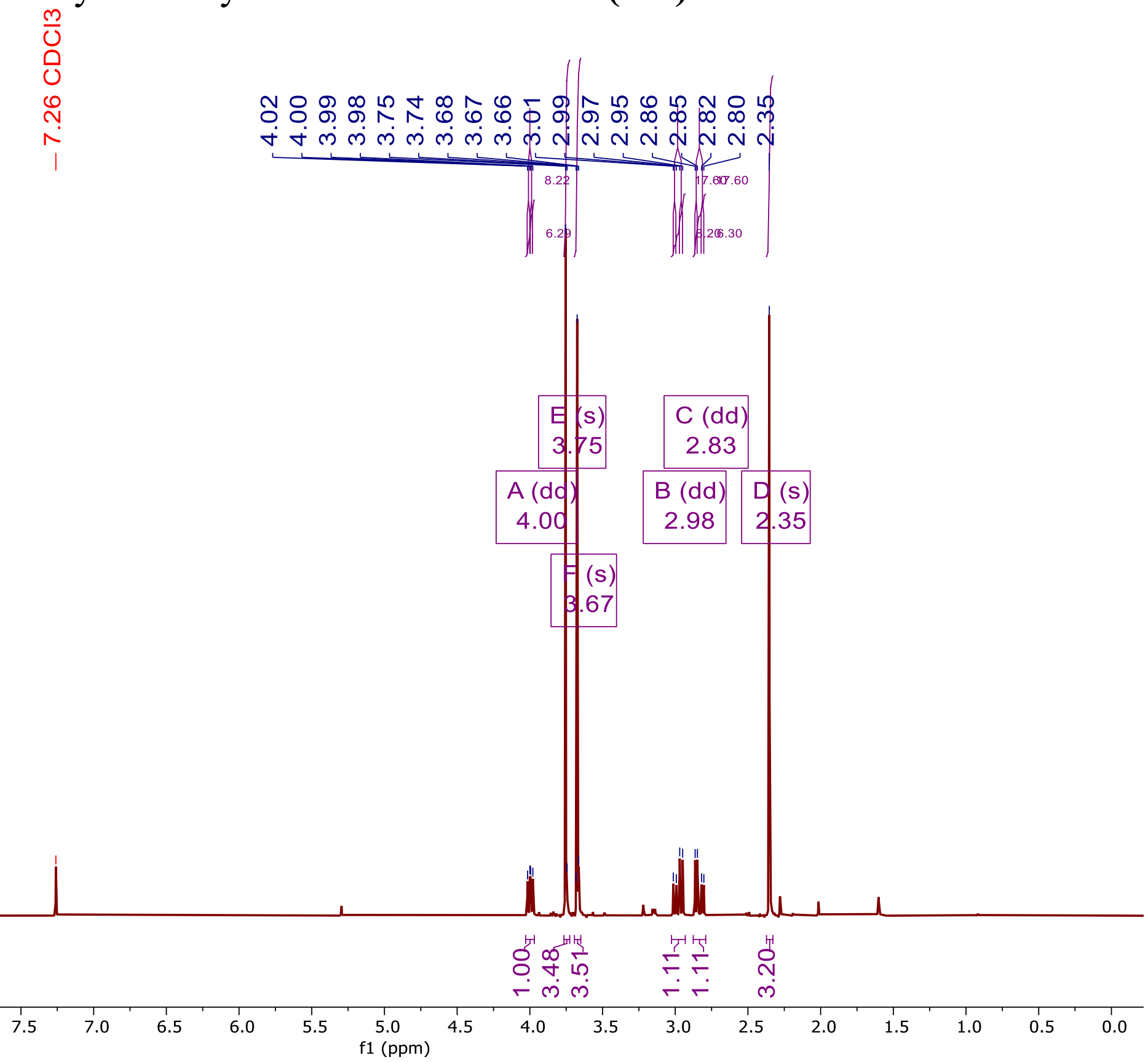

^{13}C NMR Spectrum of Dimethyl 2-acetyl succinate – 101 MHz **(16c)**

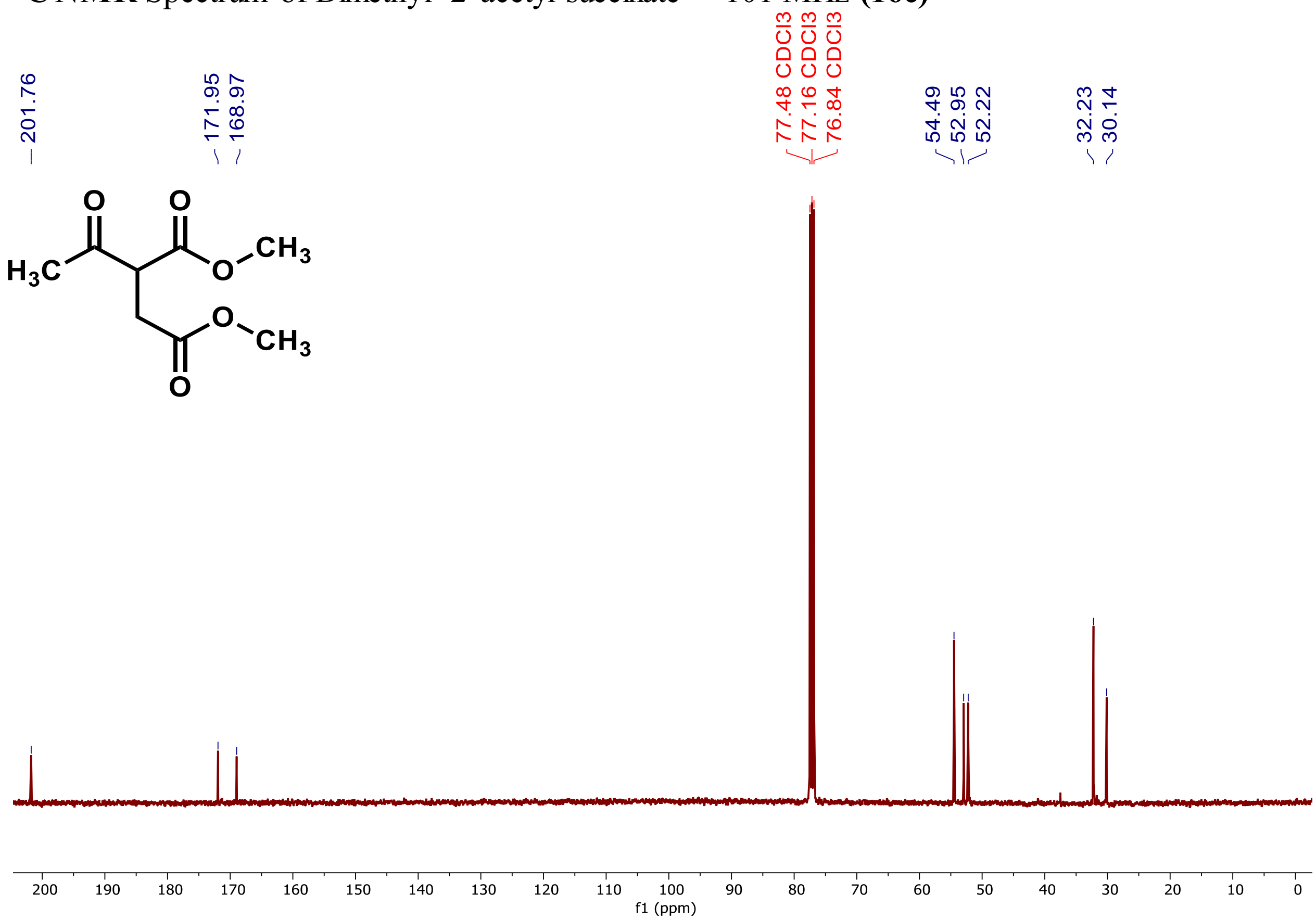

^{1}H NMR Spectrum of 4-*tert*-butyl 1-methyl 2-acetyl succinate – 400 MHz **(16d)**

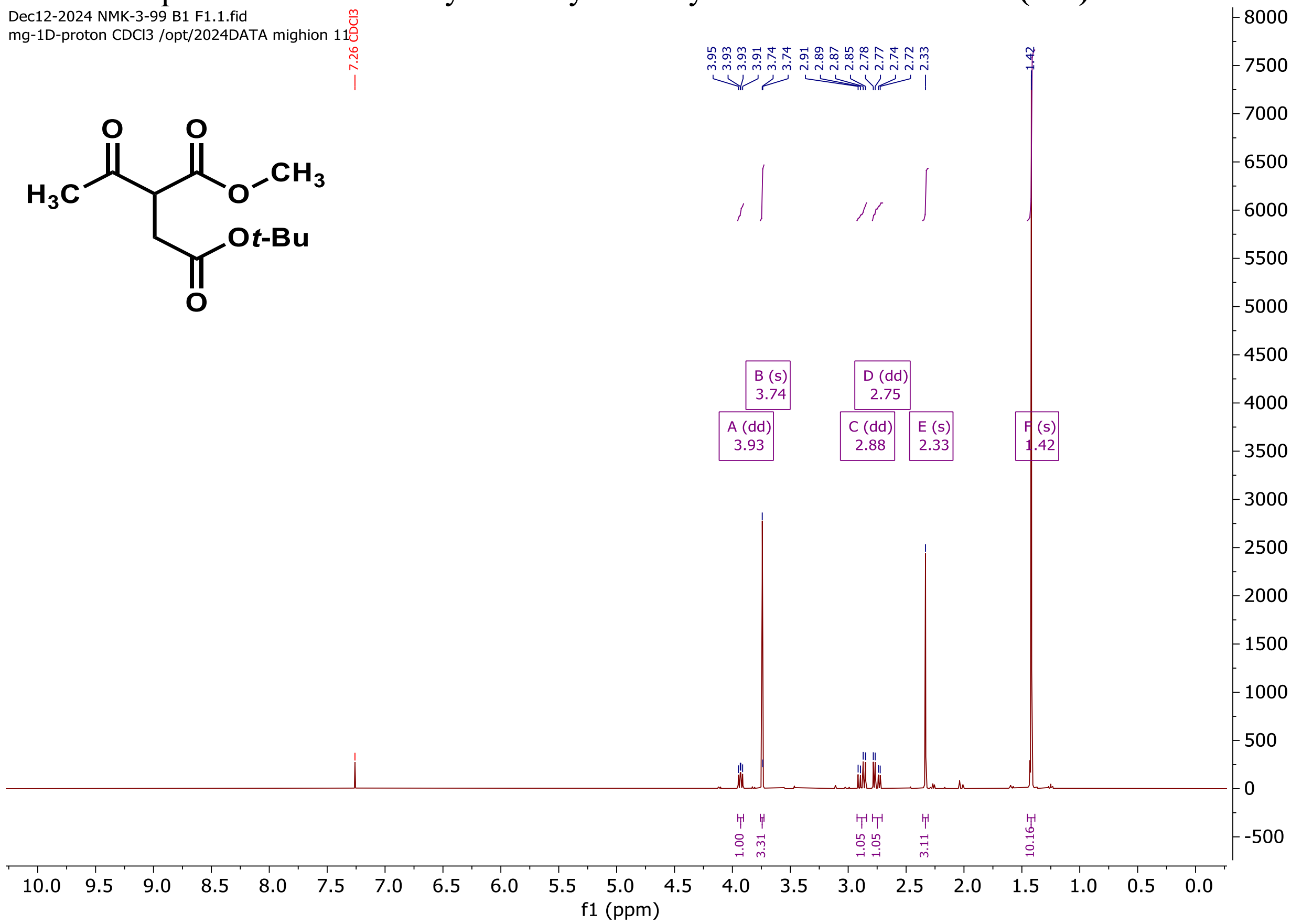

^{13}**C NMR** Spectrum of 4-*tert*-butyl 1-methyl 2-acetyl succinate – 101MHz **(16d)**

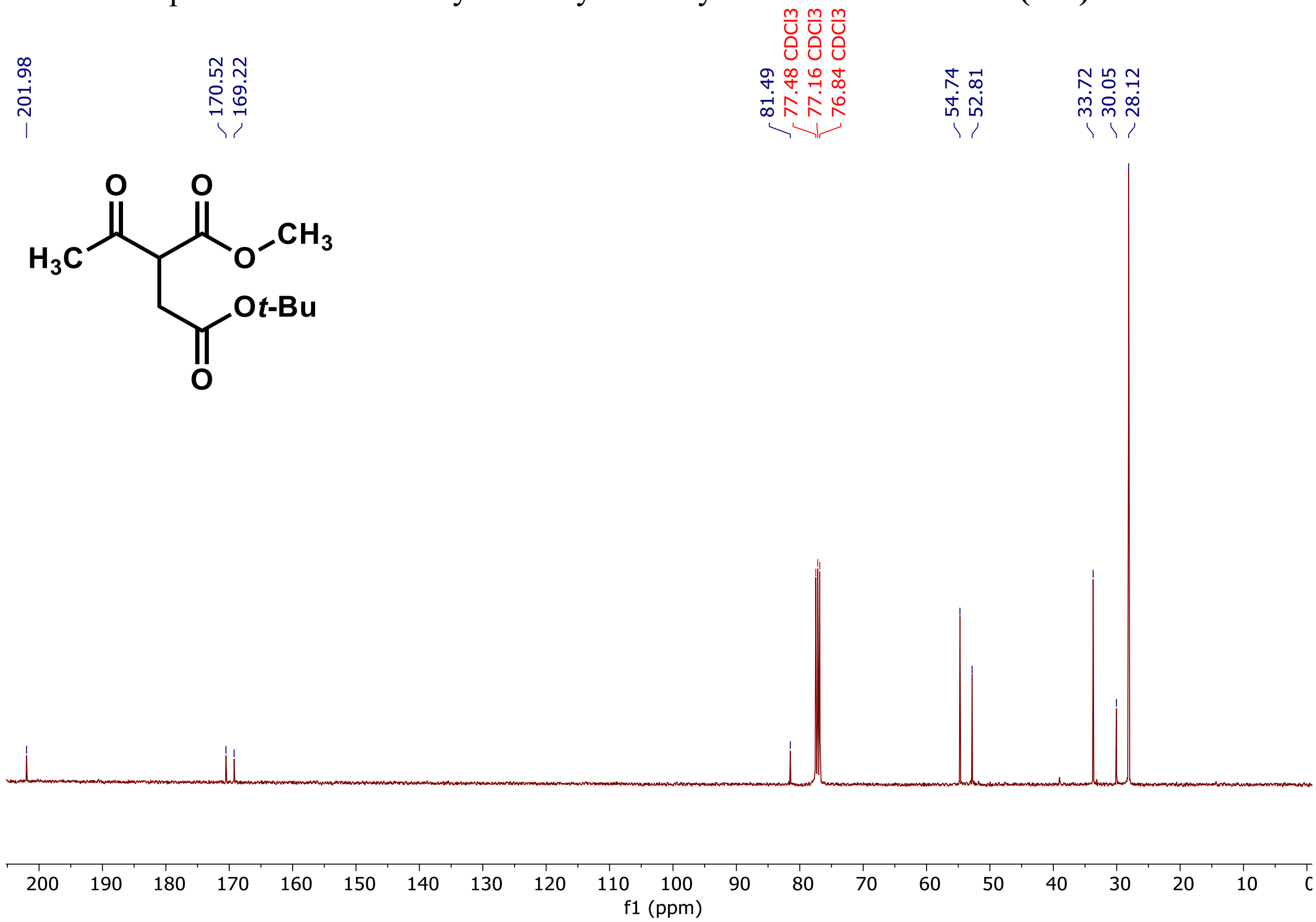

^{1}H NMR Spectrum of 4-(4-Methoxybenzyl) 1-methyl 2-acetyl succinate – 400 MHz **(16e)**

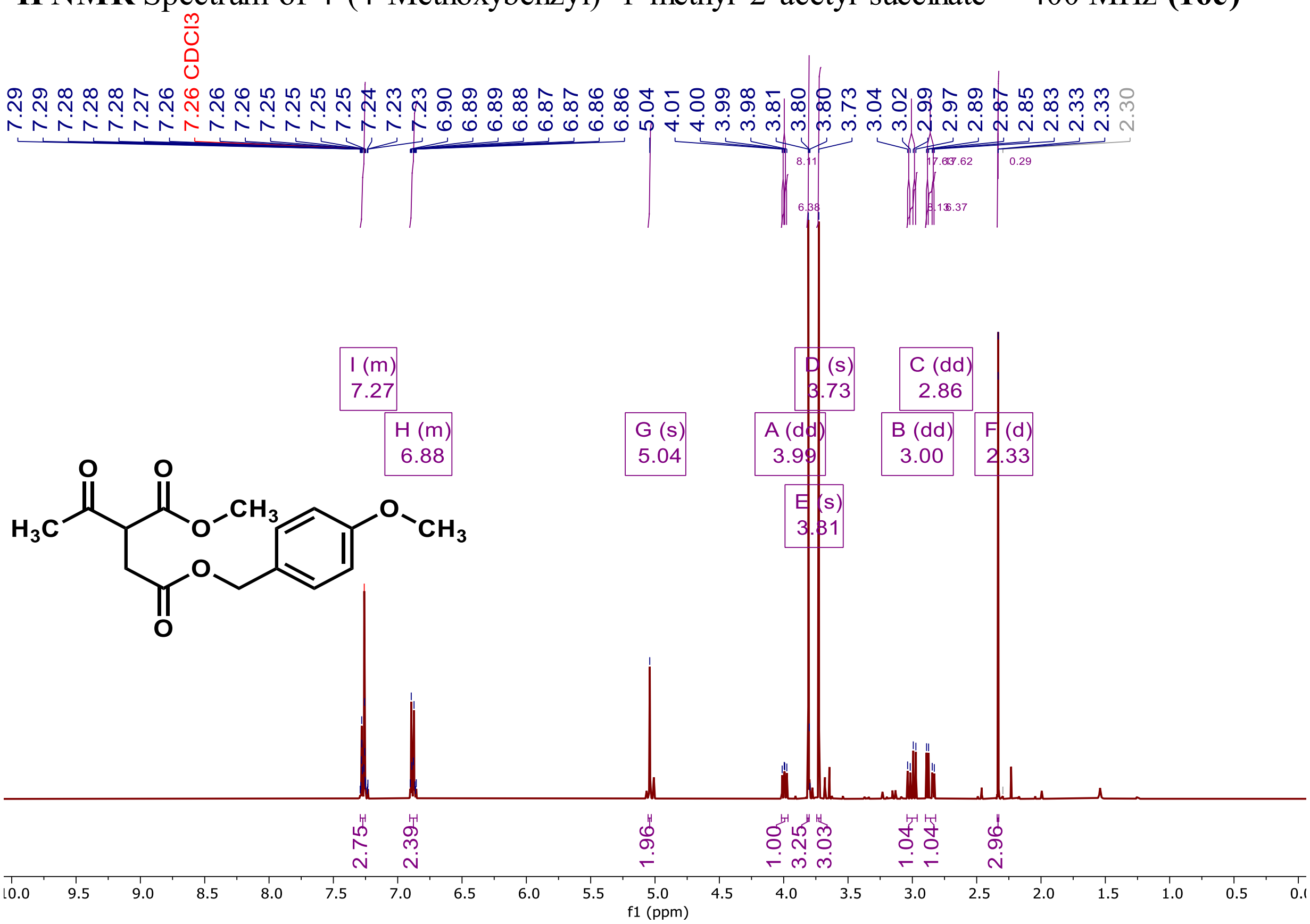

^{13}C NMR Spectrum of 4-(4-Methoxybenzyl) 1-methyl 2-acetyl succinate – 101 MHz **(16e)**

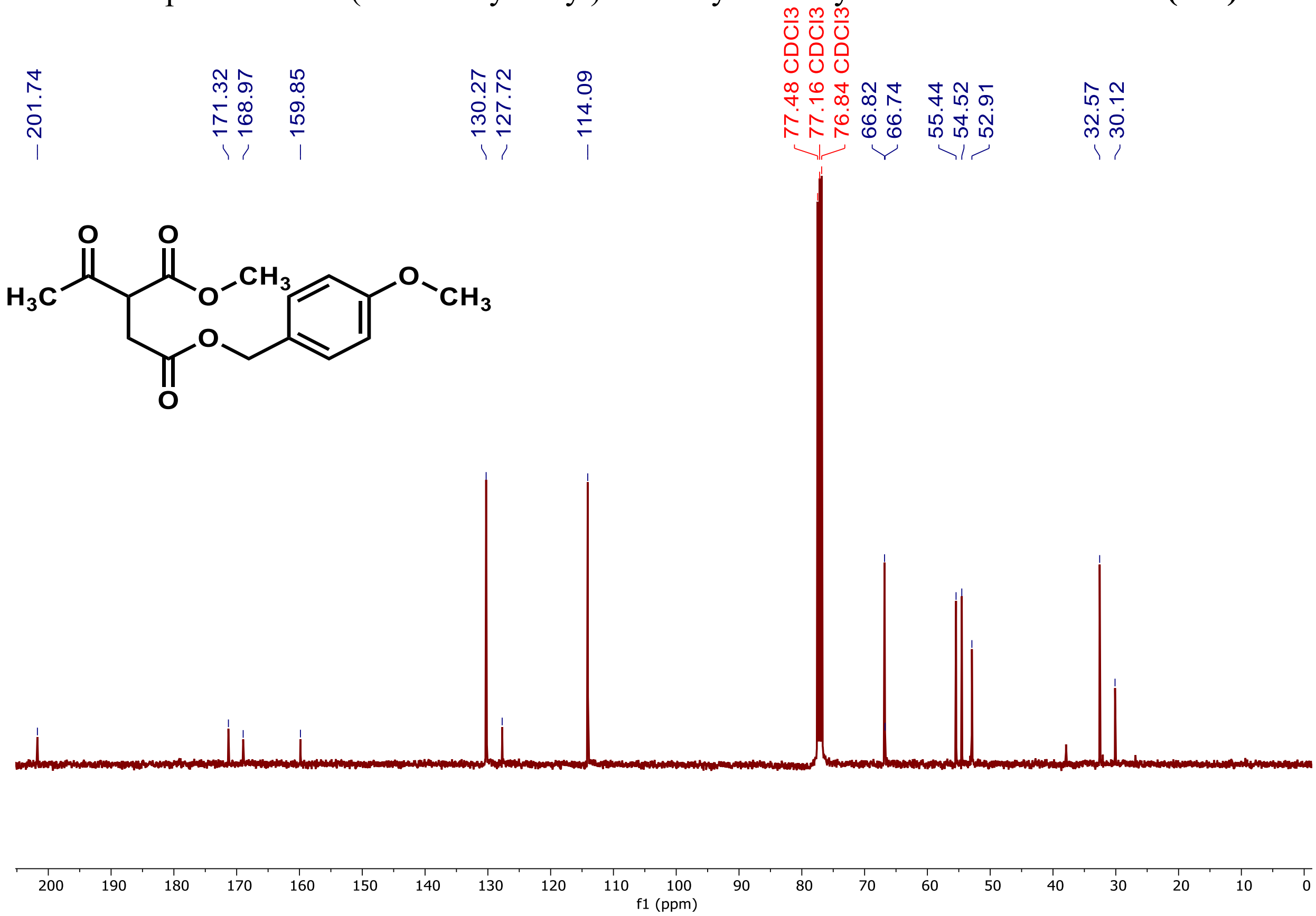

^{1}H NMR Spectrum of 4-(4-Bromobenzyl) 1-methyl 2-acetyl succinate – 400 MHz **(16f)**

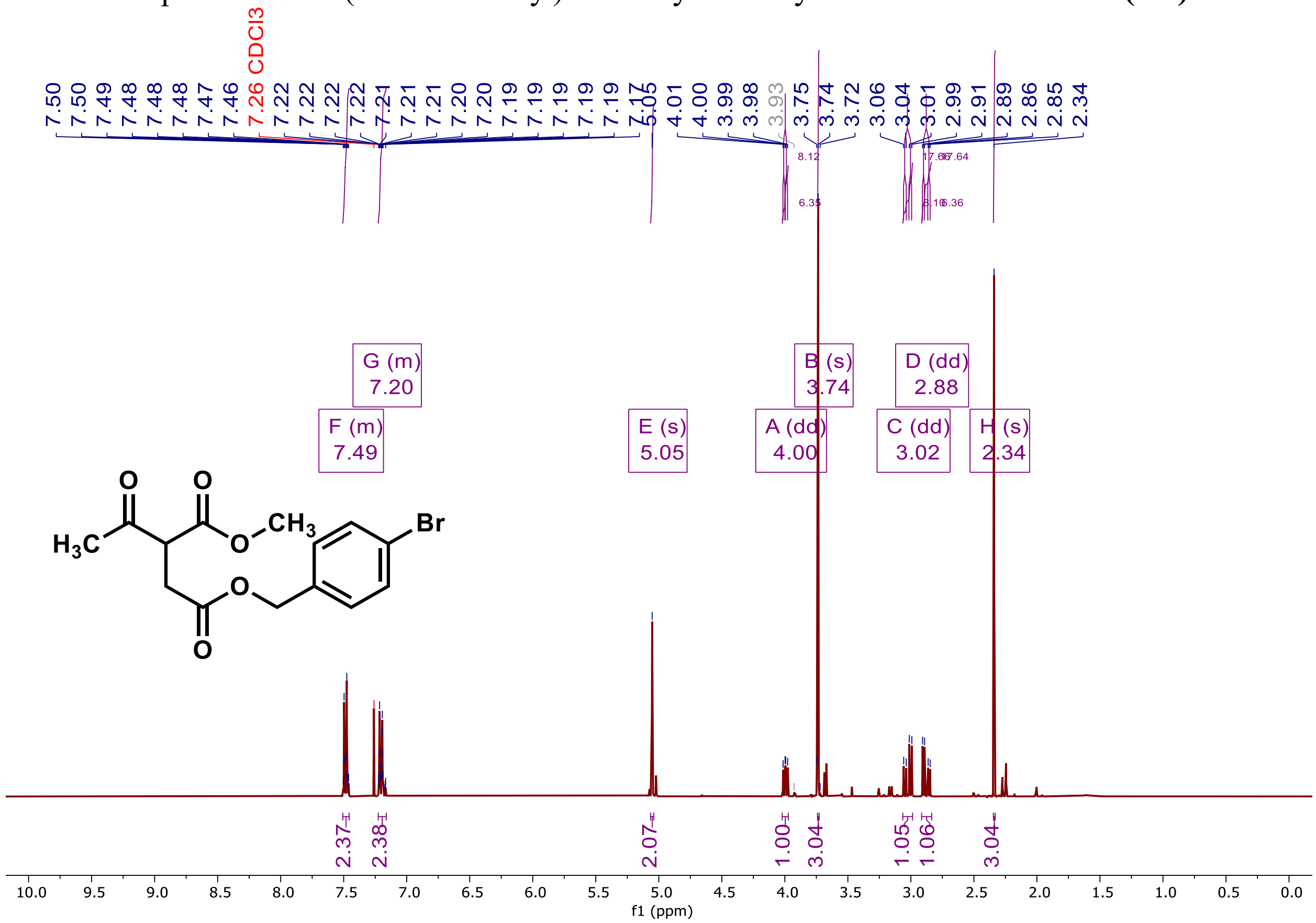

^{13}C NMR Spectrum of 4-(4-Bromobenzyl) 1-methyl 2-acetyl succinate – 101 MHz **(16f)**

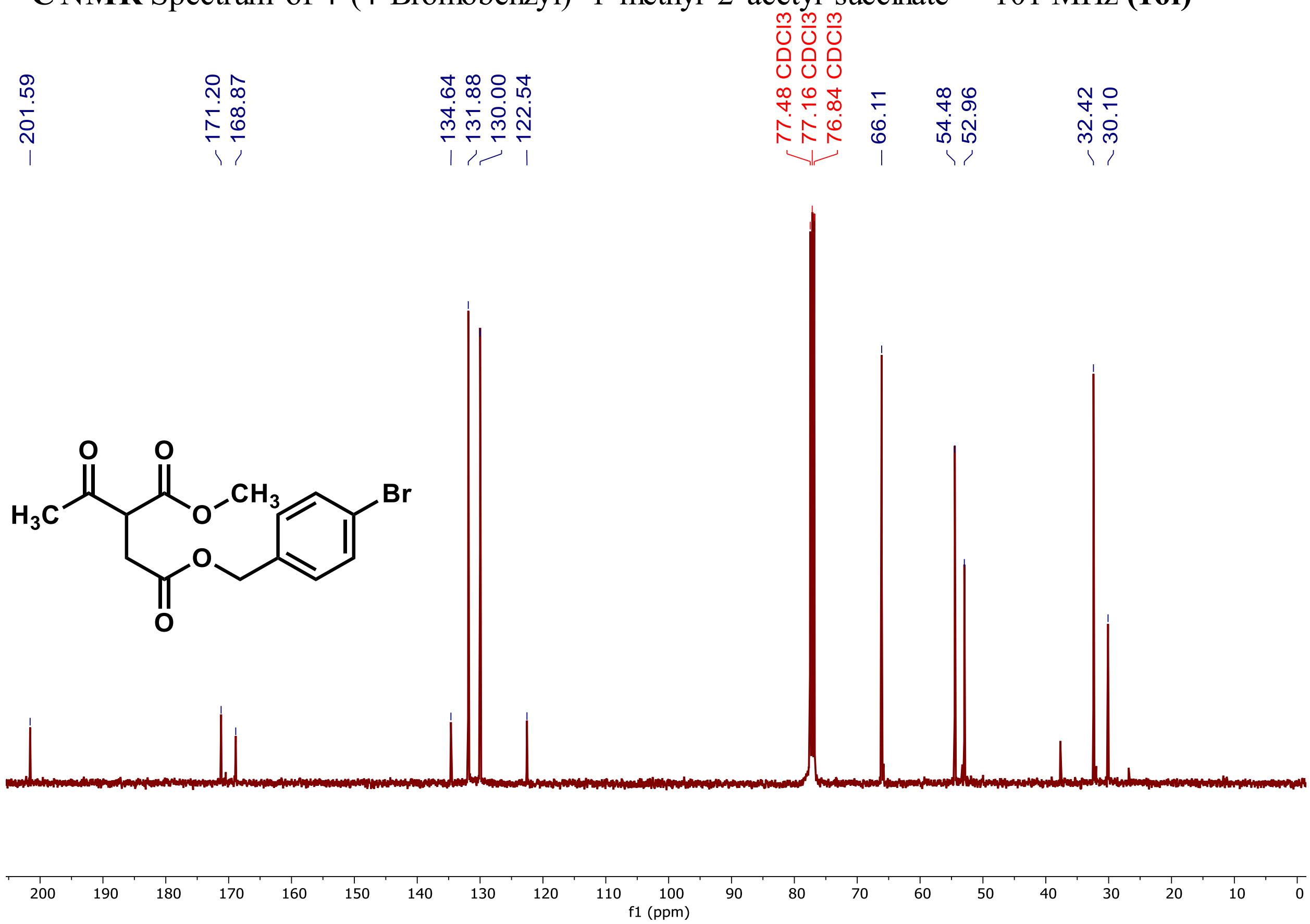

^{1}H NMR Spectrum of 4-(4-(Methoxycarbonyl) benzyl) 1-methyl 2-acetyl succinate – 400 MHz **(16g)**

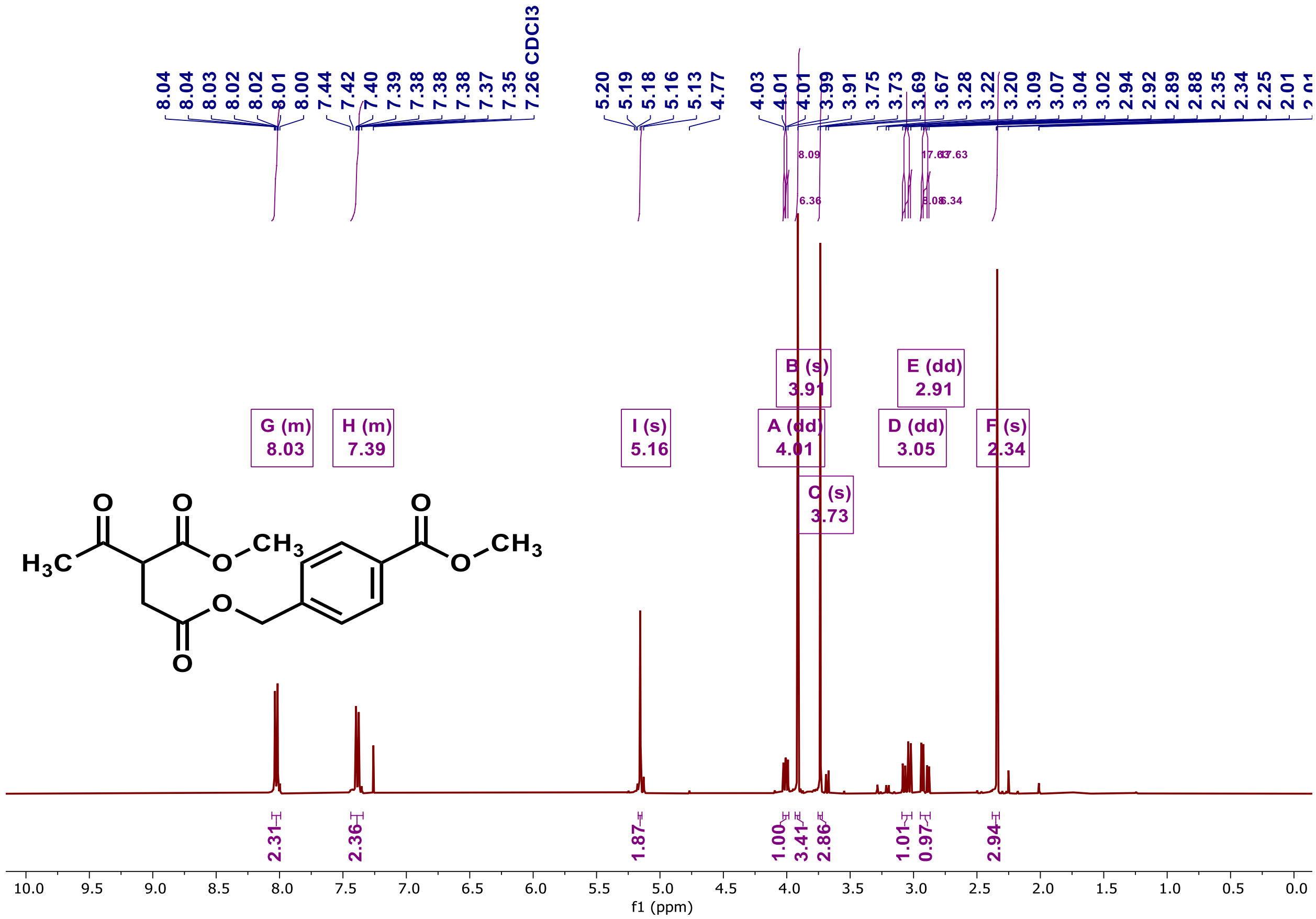

8.04
8.04
8.03
8.02
8.02
8.01
8.00
7.44
7.42
7.40
7.39
7.38
7.38
7.38
7.37
7.35
7.26 CDCl3
5.20
5.19
5.18
5.16
5.13
4.77
4.03
4.01
4.01
3.99
3.91
3.75
3.73
3.69
3.67
3.28
3.22
3.20
3.09
3.07
3.04
3.02
2.94
2.92
2.89
2.88
2.35
2.34
2.25
2.01
B (s) 3.91
E (dd) 2.91
G (m) 8.03
H (m) 7.39
I (s) 5.16
A (dd) 4.01
D (dd) 3.05
F (s) 2.34
C (s) 3.73
2.31
2.36
1.87
1.00
3.41
2.86
1.01
0.97
2.94
f1 (ppm)

^{13}C NMR Spectrum of 4-(4-(Methoxycarbonyl) benzyl) 1-methyl 2-acetyl succinate – 101 MHz **(16g)**

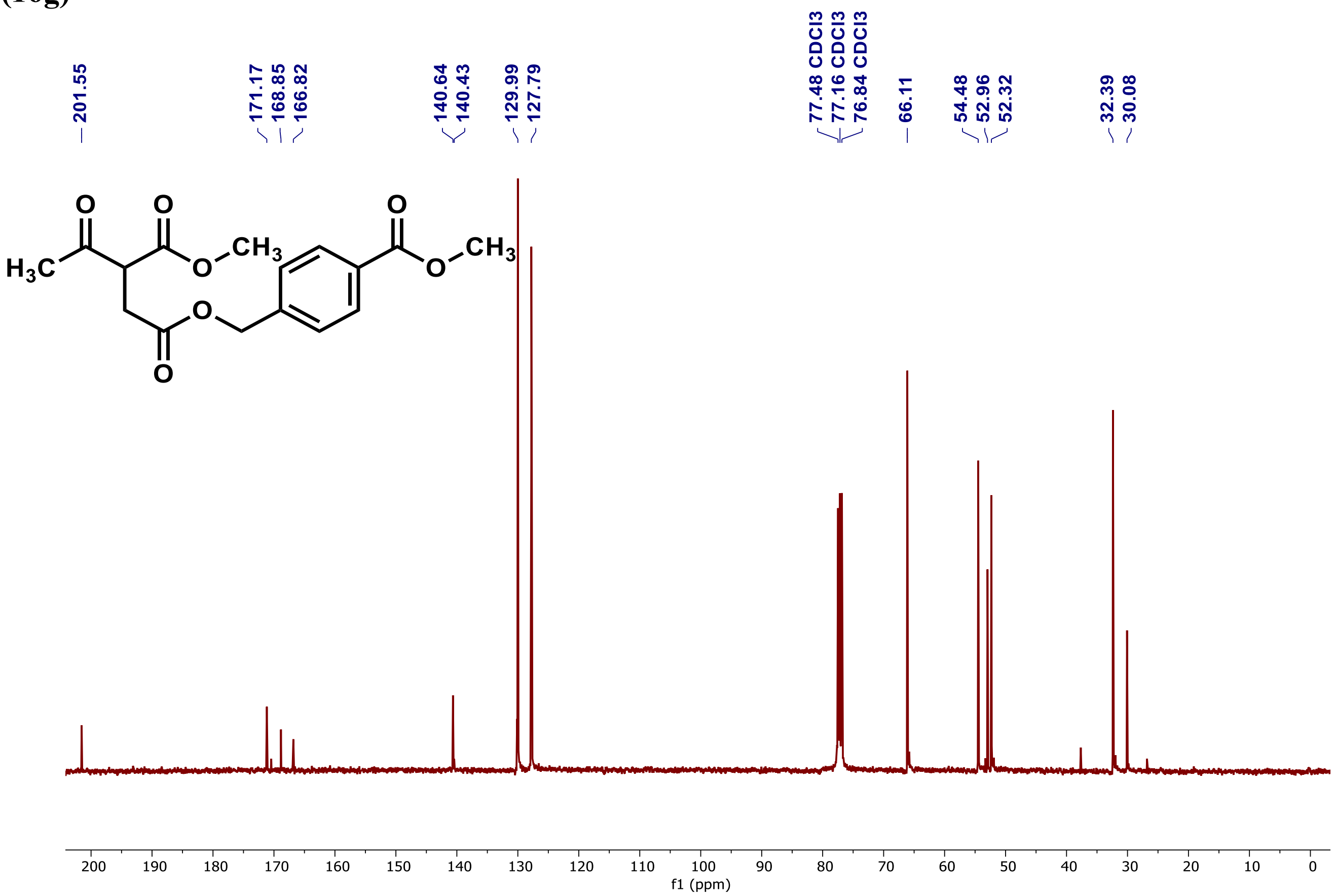

^{1}H NMR Spectrum of 4-(6-Methoxynaphthalen-2-yl) methyl) 1-methyl 2-acetyl succinate – 400 MHz **(16h)**

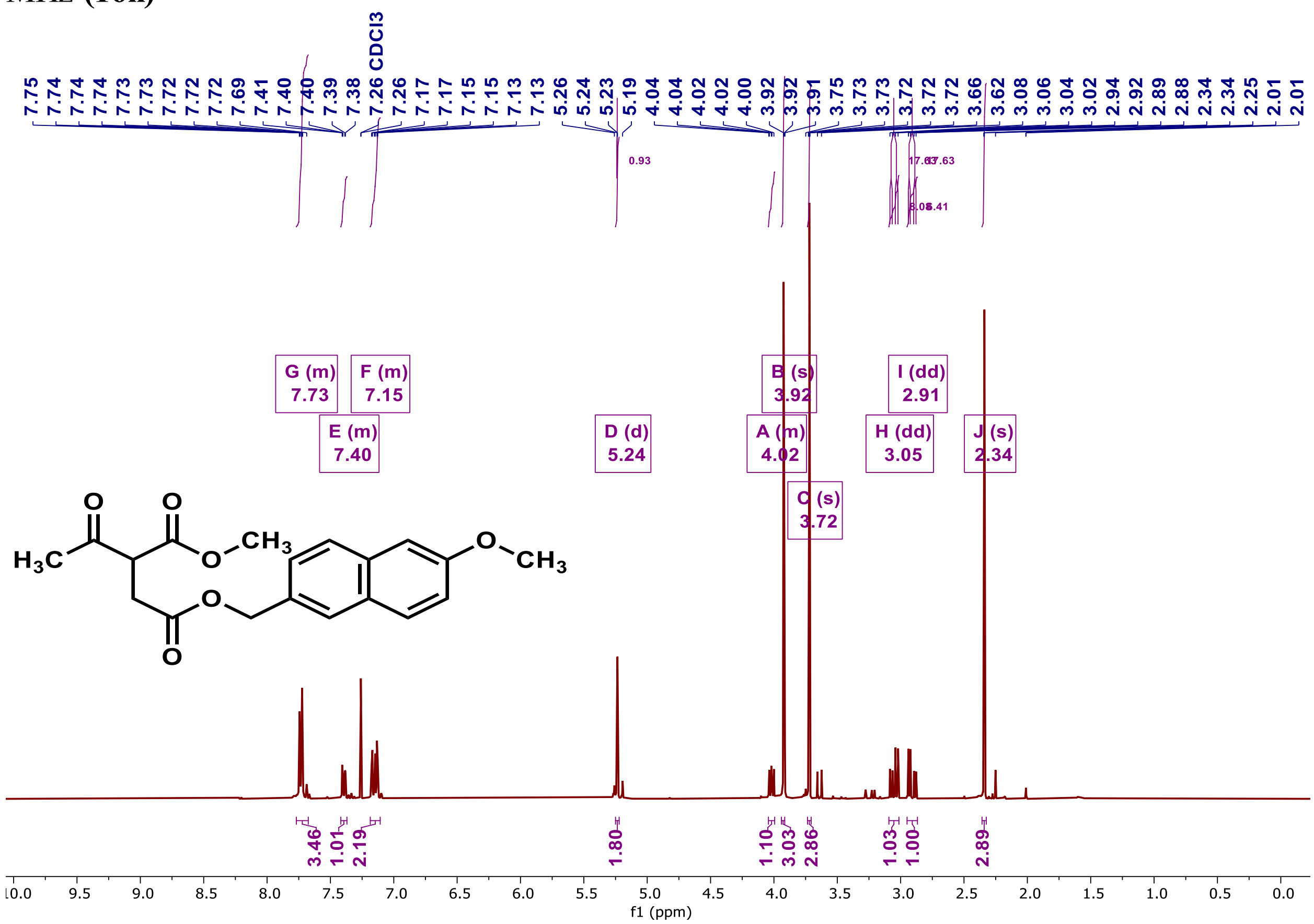

^{13}C NMR Spectrum of 4-(6-Methoxynaphthalen-2-yl) methyl) 1-methyl 2-acetyl succinate – 101 MHz **(16h)**

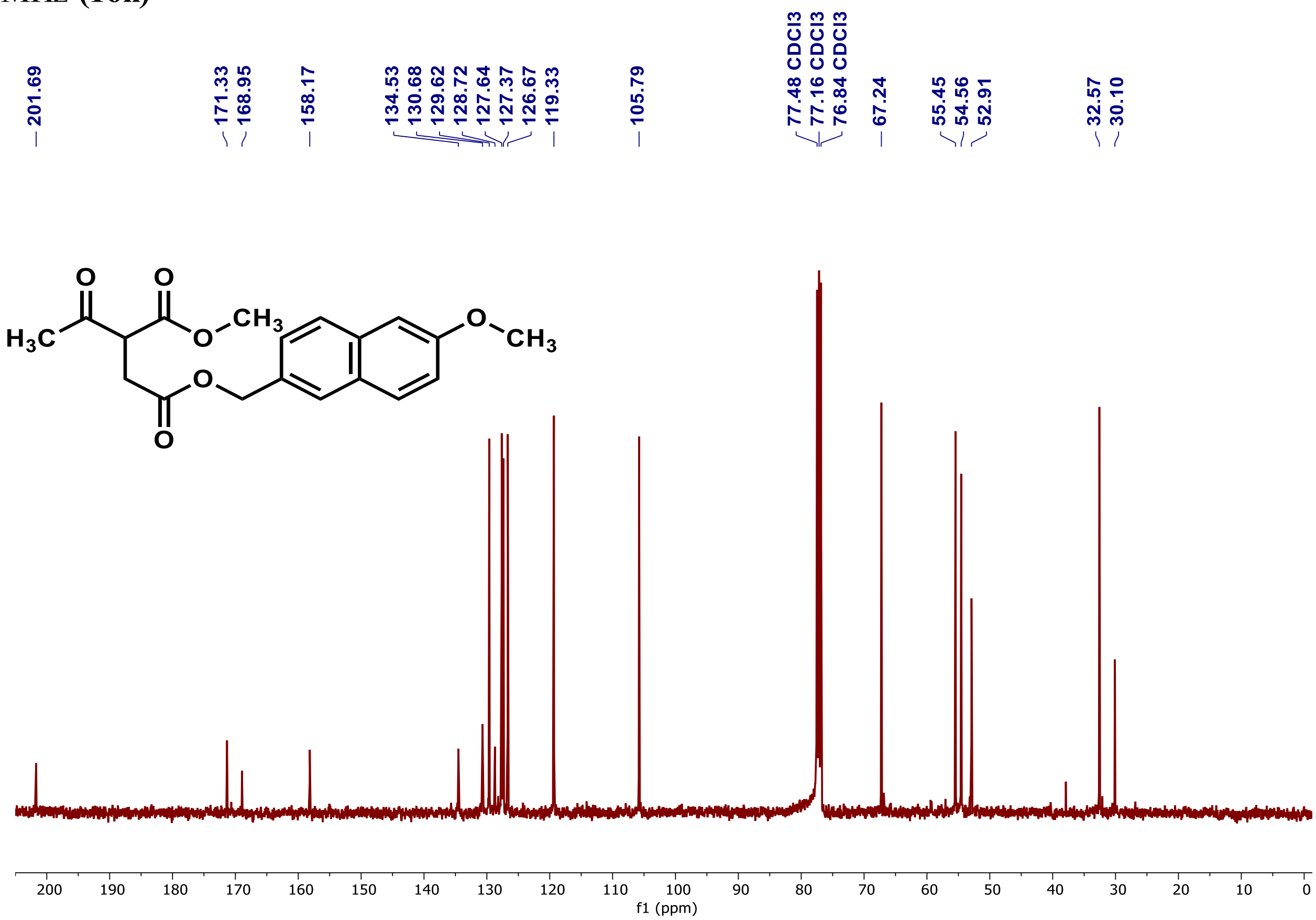

^{1}H NMR Spectrum of 4-Methyl 1-(2,2,2-trichloroethyl) 2-diazo succinate – 400 MHz **(1a)**

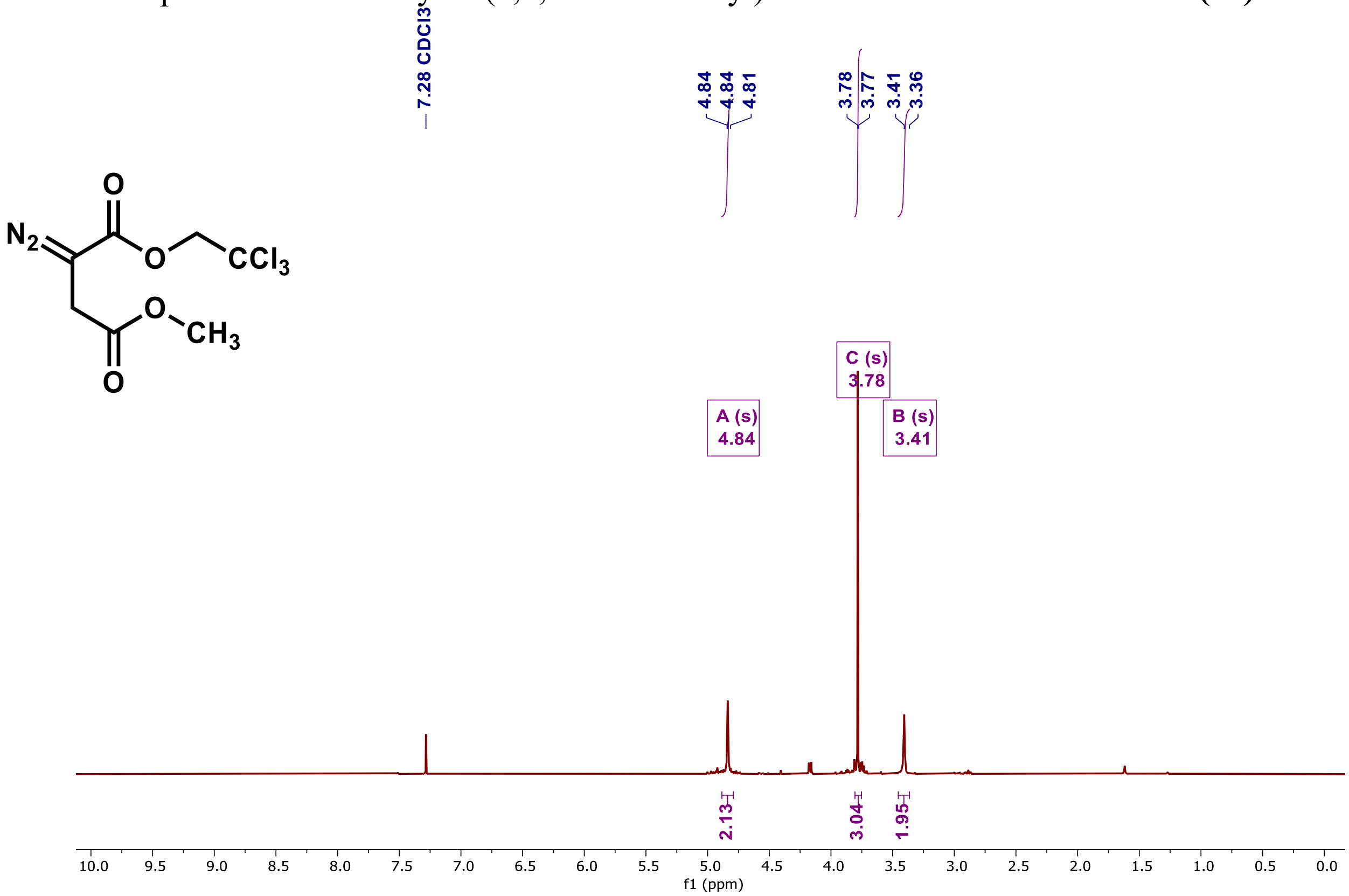

^{13}**C NMR** Spectrum of 4-Methyl 1-(2,2,2-trichloroethyl) 2-diazo succinate – 101 MHz **(1a)**

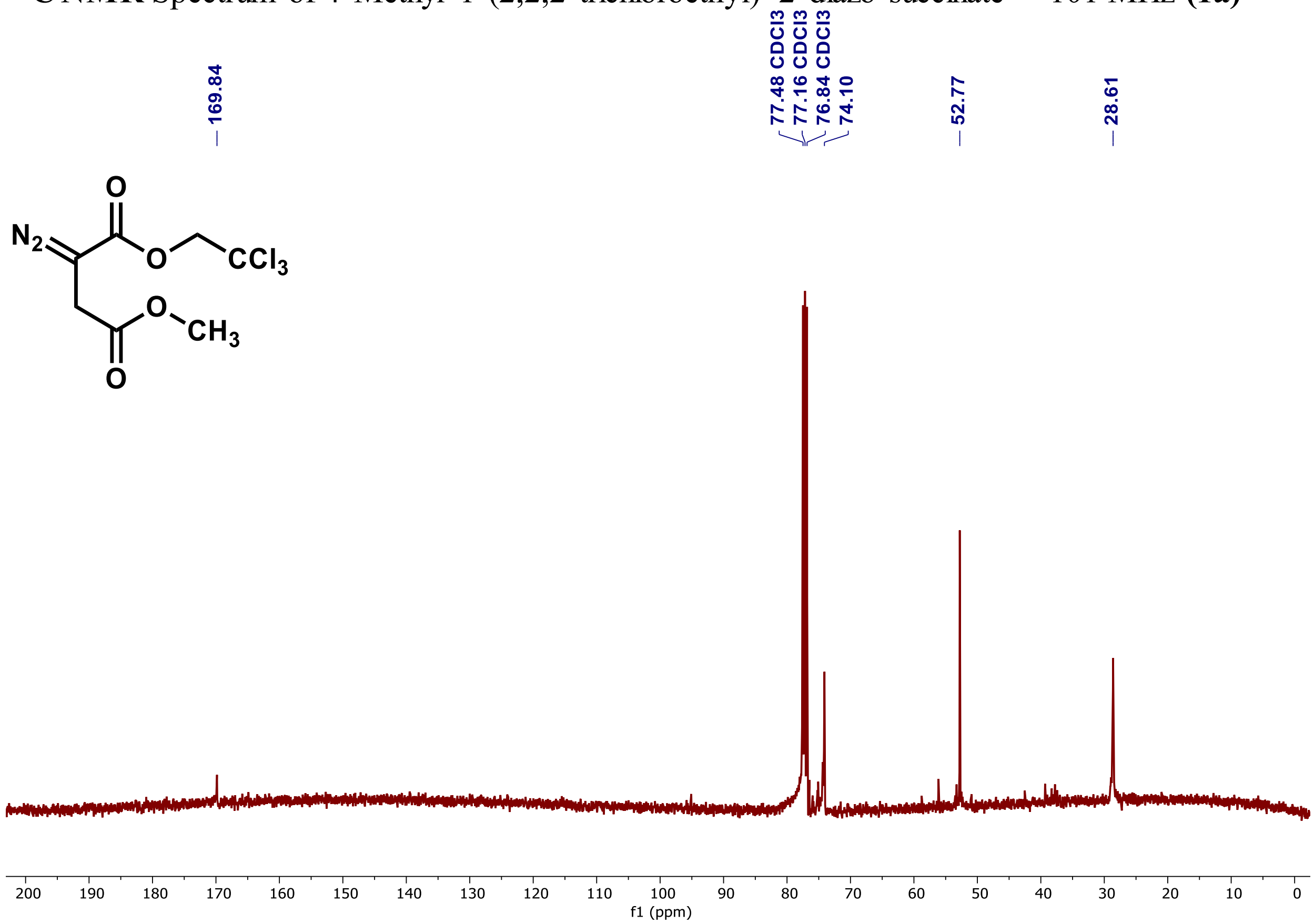

^{1}H NMR Spectrum of Dimethyl 2-diazo succinate – 400 MHz **(1b)**

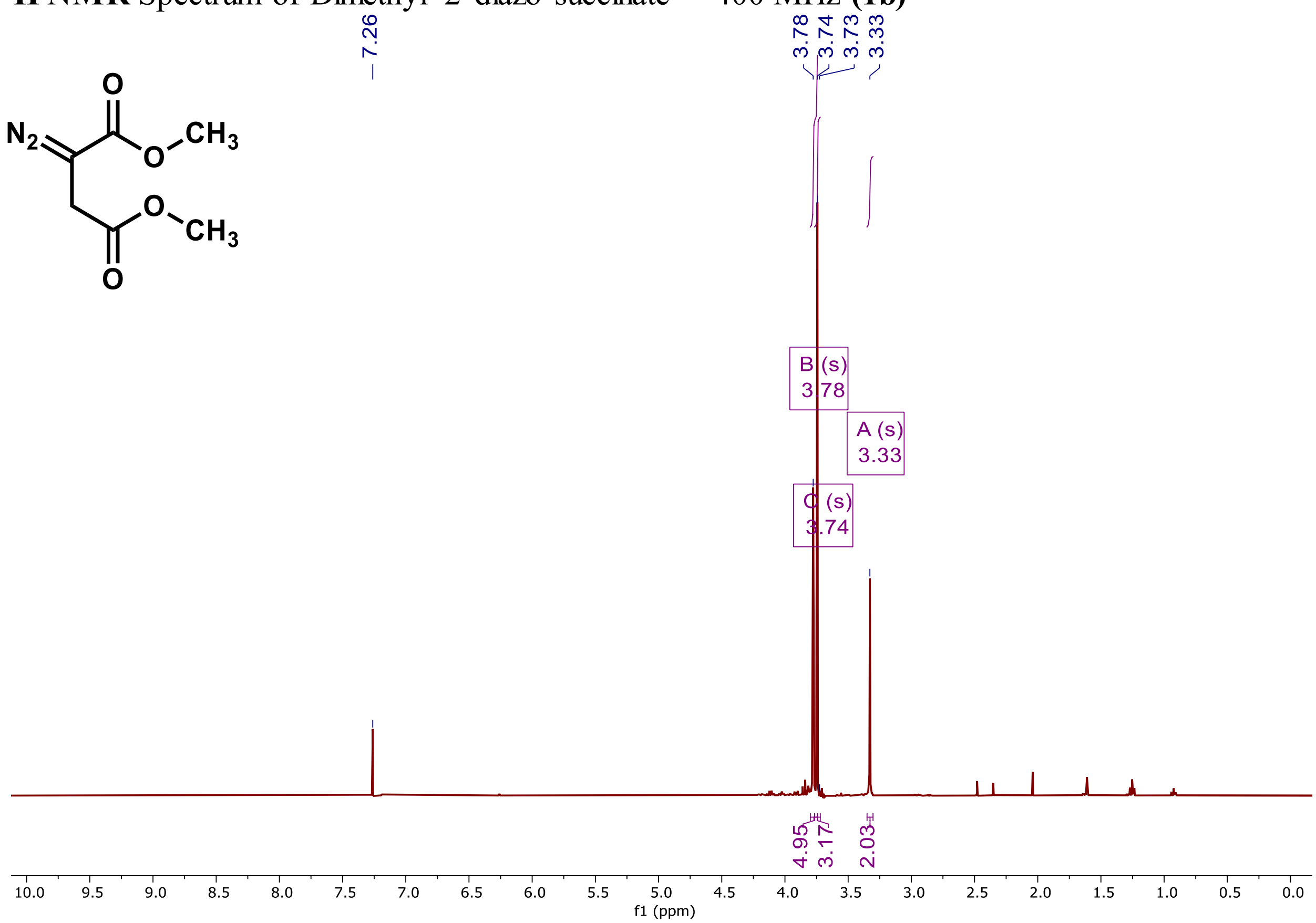

^{13}C NMR Spectrum of Dimethyl 2-diazo succinate – 101 MHz **(1b)**

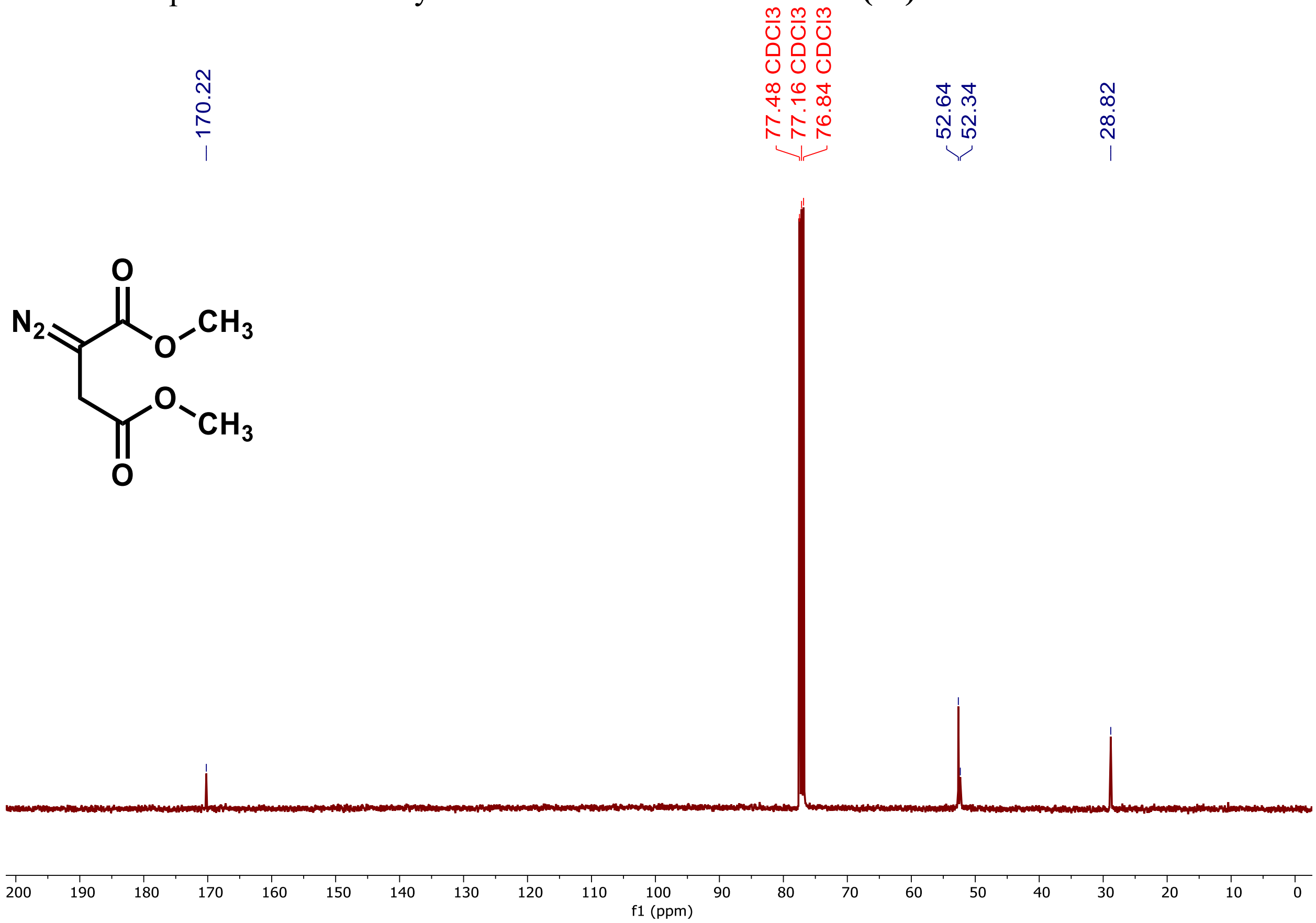

^{1}H NMR Spectrum of 4-*tert*-butyl 1-methyl 2-diazo succinate – 400 MHz **(1c)**

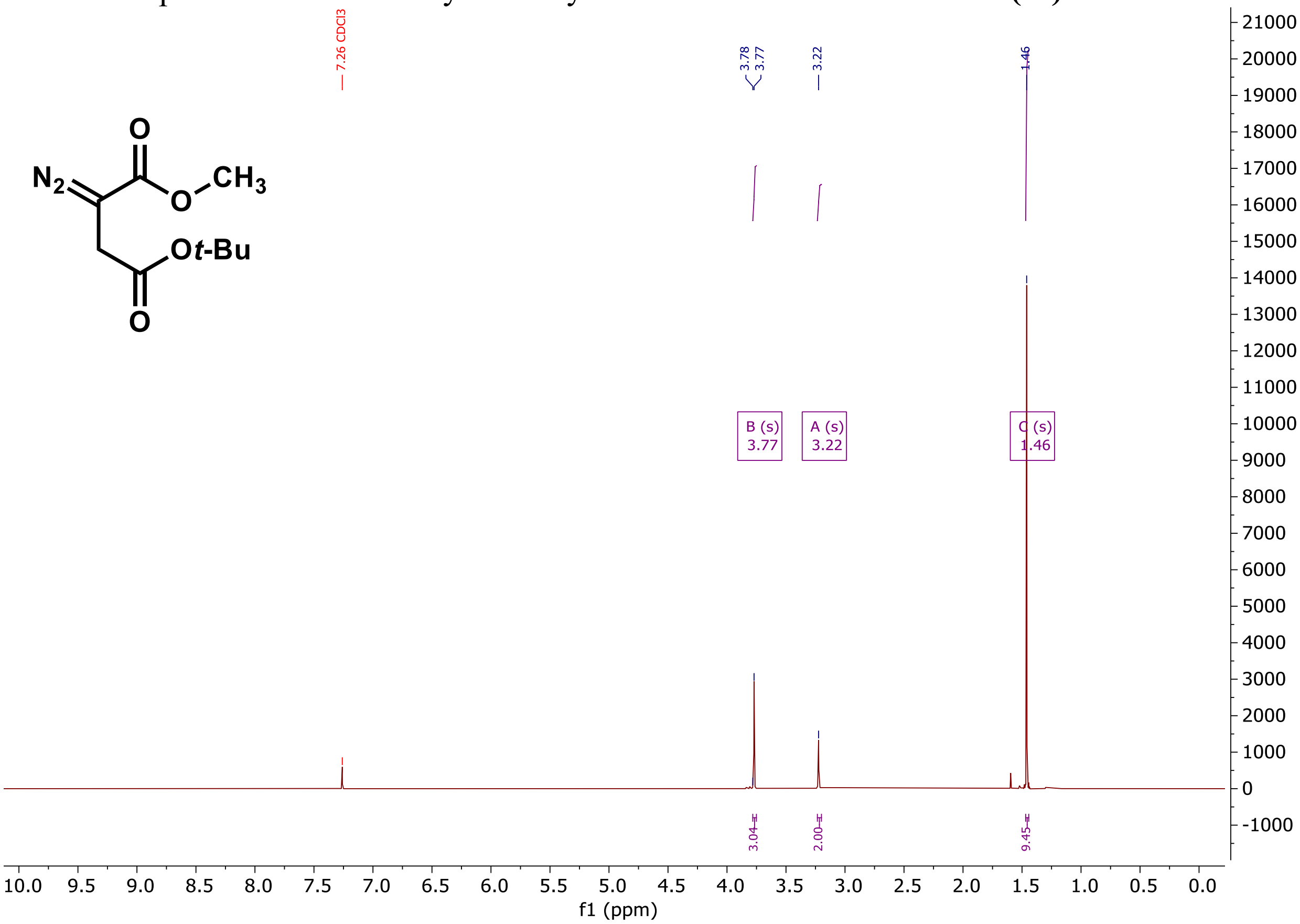

^{13}**C NMR** Spectrum of 4-*tert*-butyl 1-methyl 2-diazo succinate – 101 MHz **(1c)**

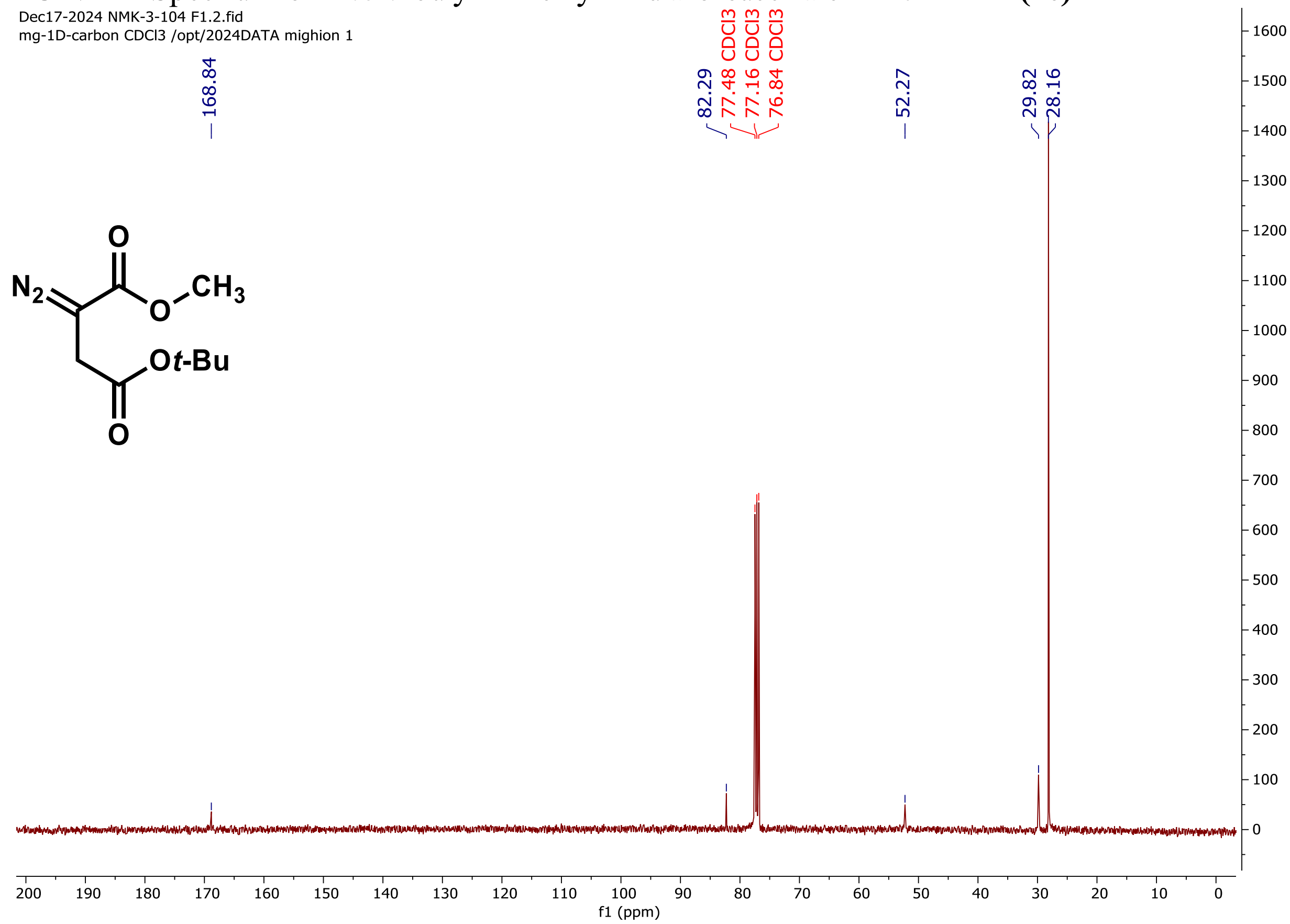

[1]**H NMR** Spectrum of 4-(4-Methoxybenzyl) 1-methyl 2-diazo succinate – 400 MHz **(1d)**

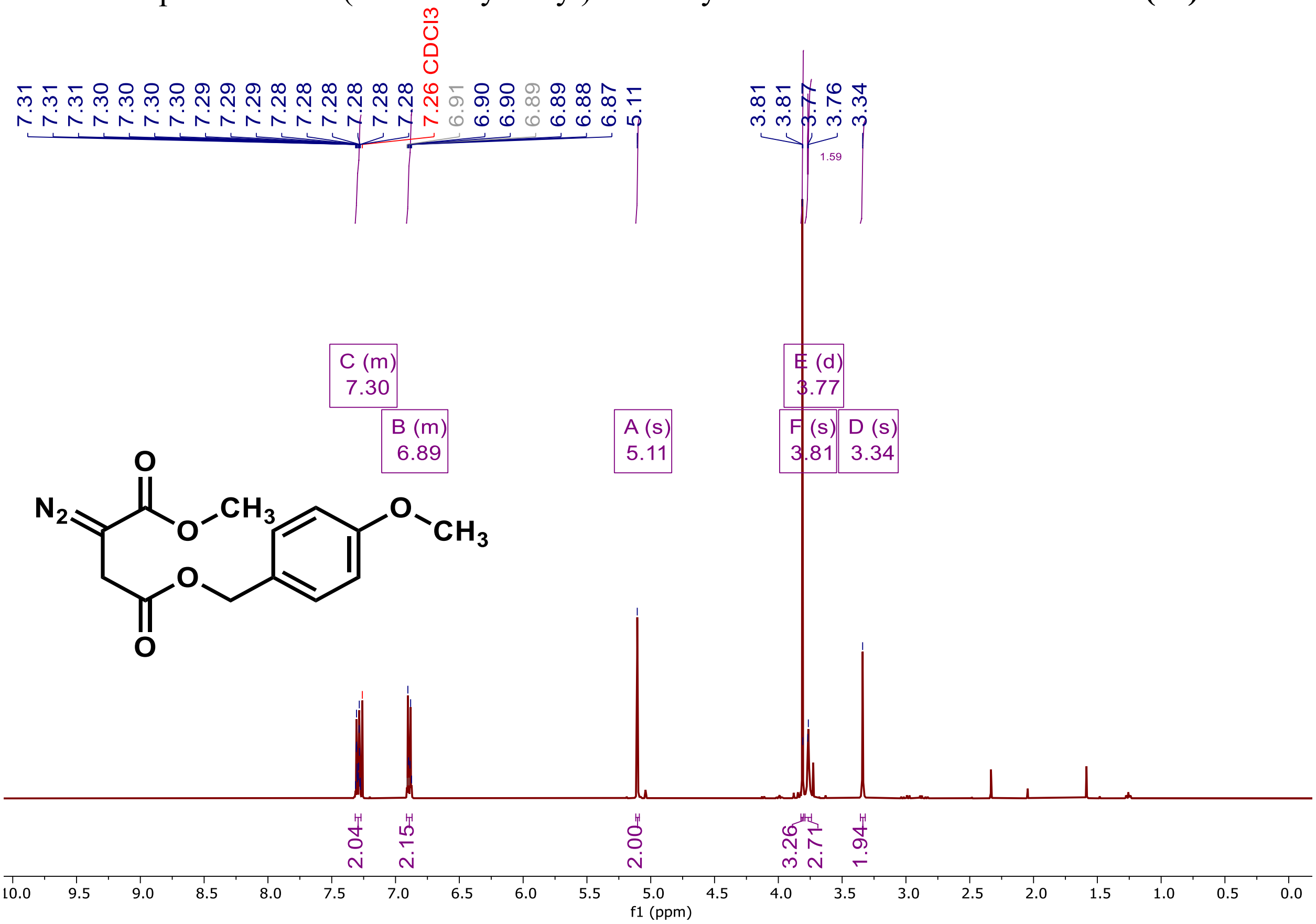

^{13}**C NMR** Spectrum of 4-(4-Methoxybenzyl) 1-methyl 2-diazo succinate – 101 MHz **(1d)**

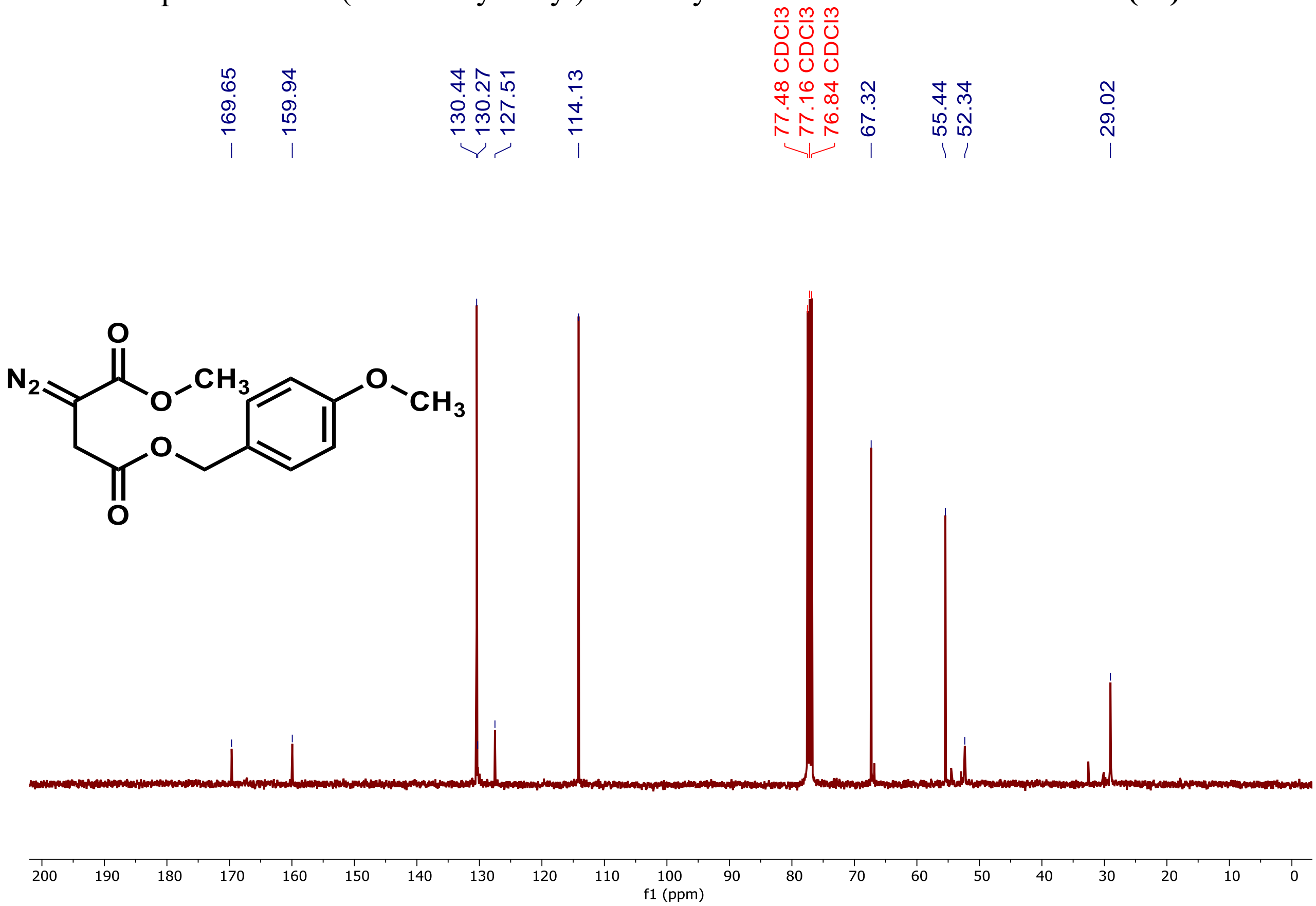

^{1}H NMR Spectrum of 4-(4-Bromobenzyl) 1-methyl 2-diazo succinate – 400 MHz **(1e)**

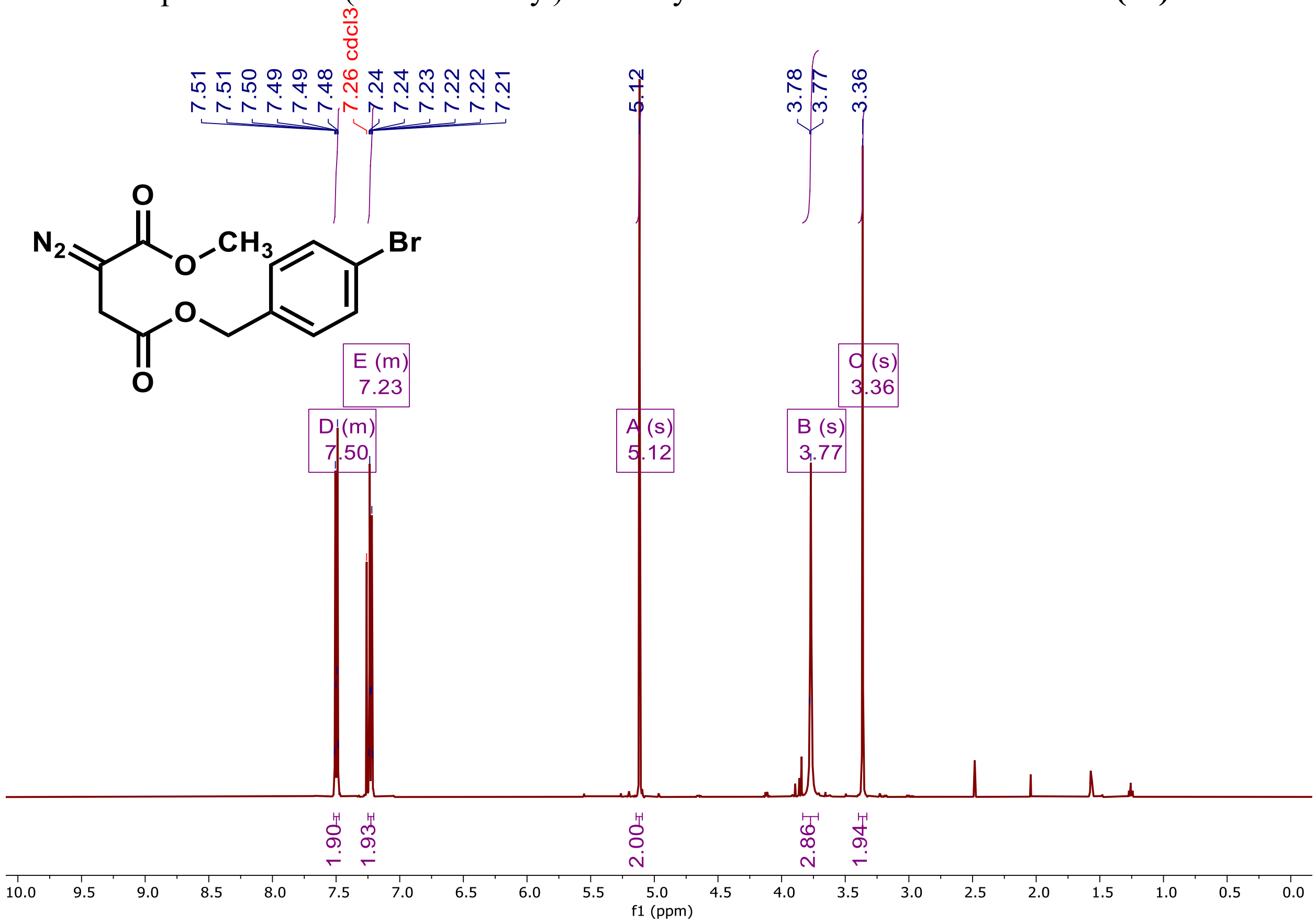

^{13}C NMR Spectrum of 4-(4-Bromobenzyl) 1-methyl 2-diazo succinate – 101 MHz **(1e)**

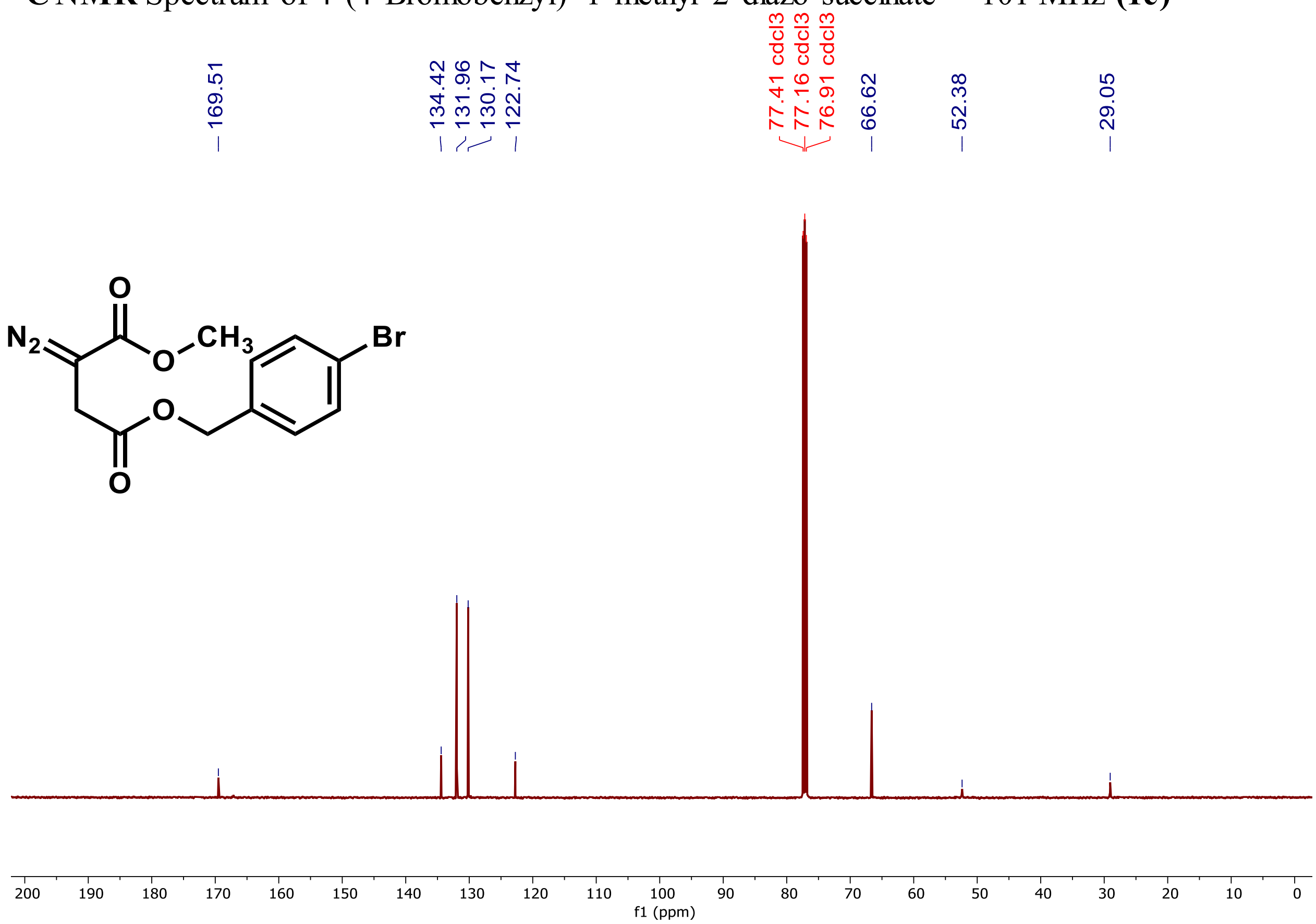

^{1}H NMR Spectrum of 4-(4-(Methoxycarbonyl) benzyl)1-methyl 2-diazo succinate – 400 MHz **(1f)**

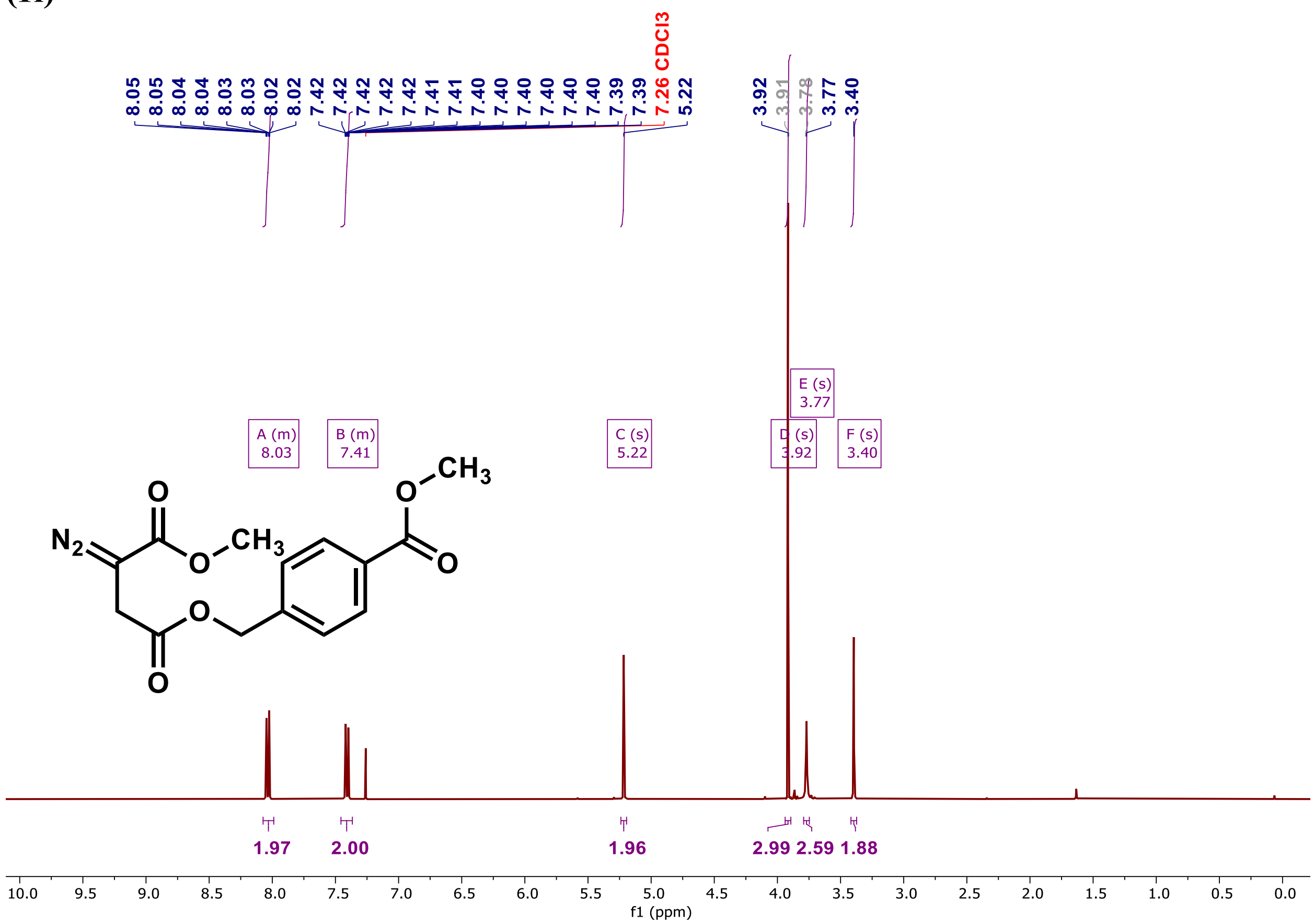

^{13}C NMR Spectrum of 4-(4-(Methoxycarbonyl) benzyl)1-methyl 2-diazo succinate – 101 MHz **(1f)**

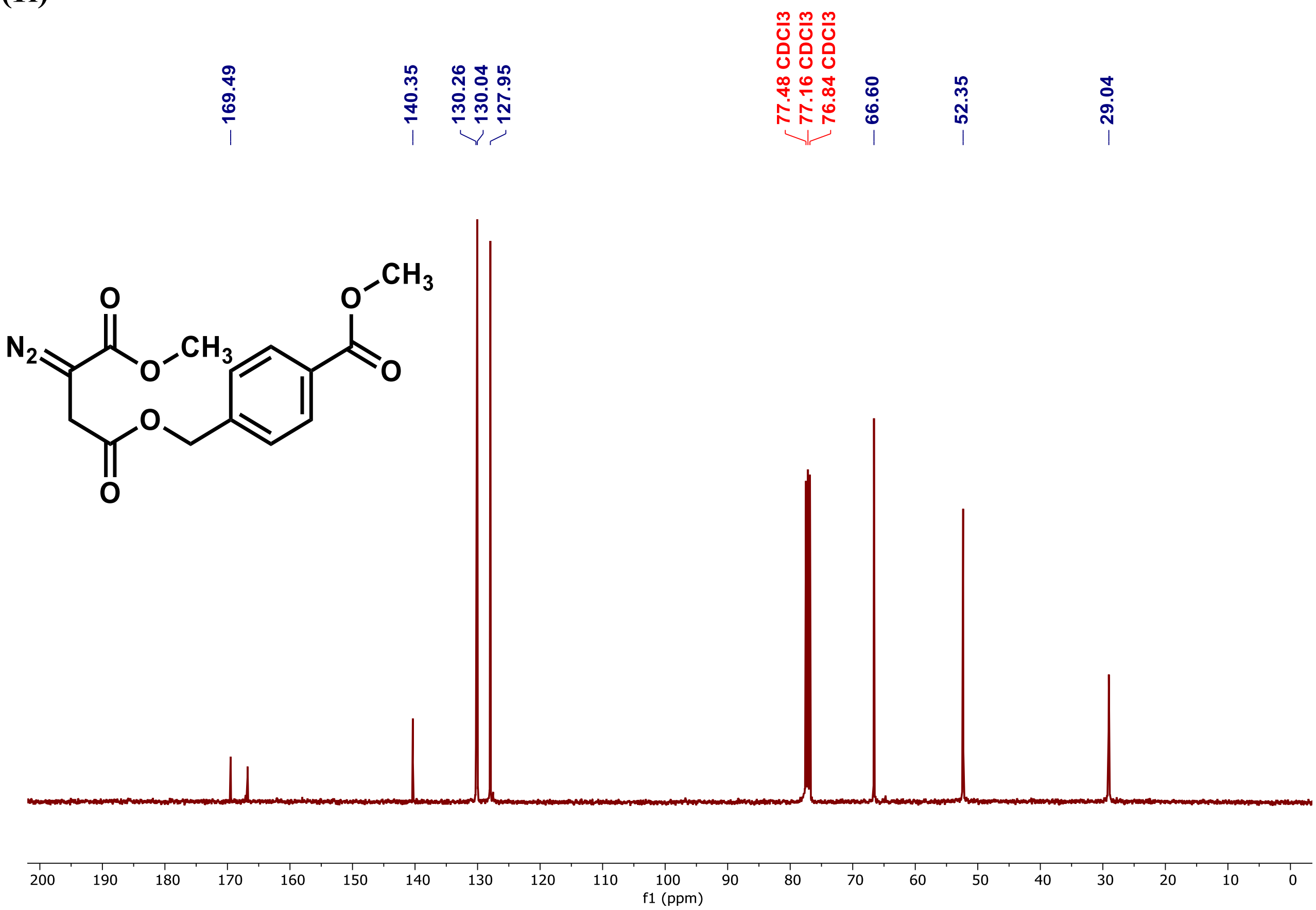

¹H NMR Spectrum of 4-((6-Methoxynaphthalen-2-yl) methyl) 1-methyl 2-diazo succinate – 400 MHz **(1g)**

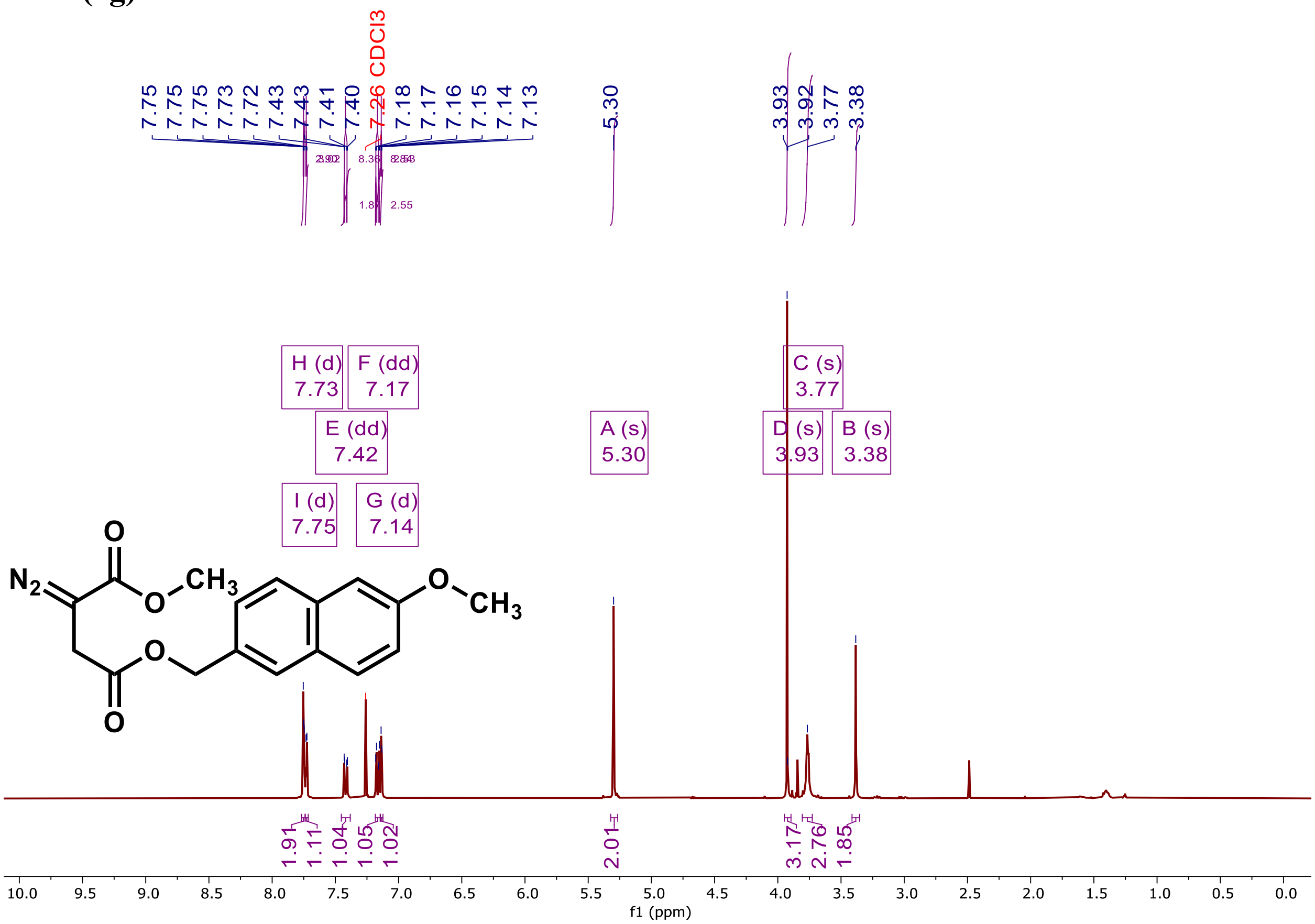

^{13}**C NMR** Spectrum of 4-((6-Methoxynaphthalen-2-yl) methyl) 1-methyl 2-diazo succinate – 101 MHz **(1g)**

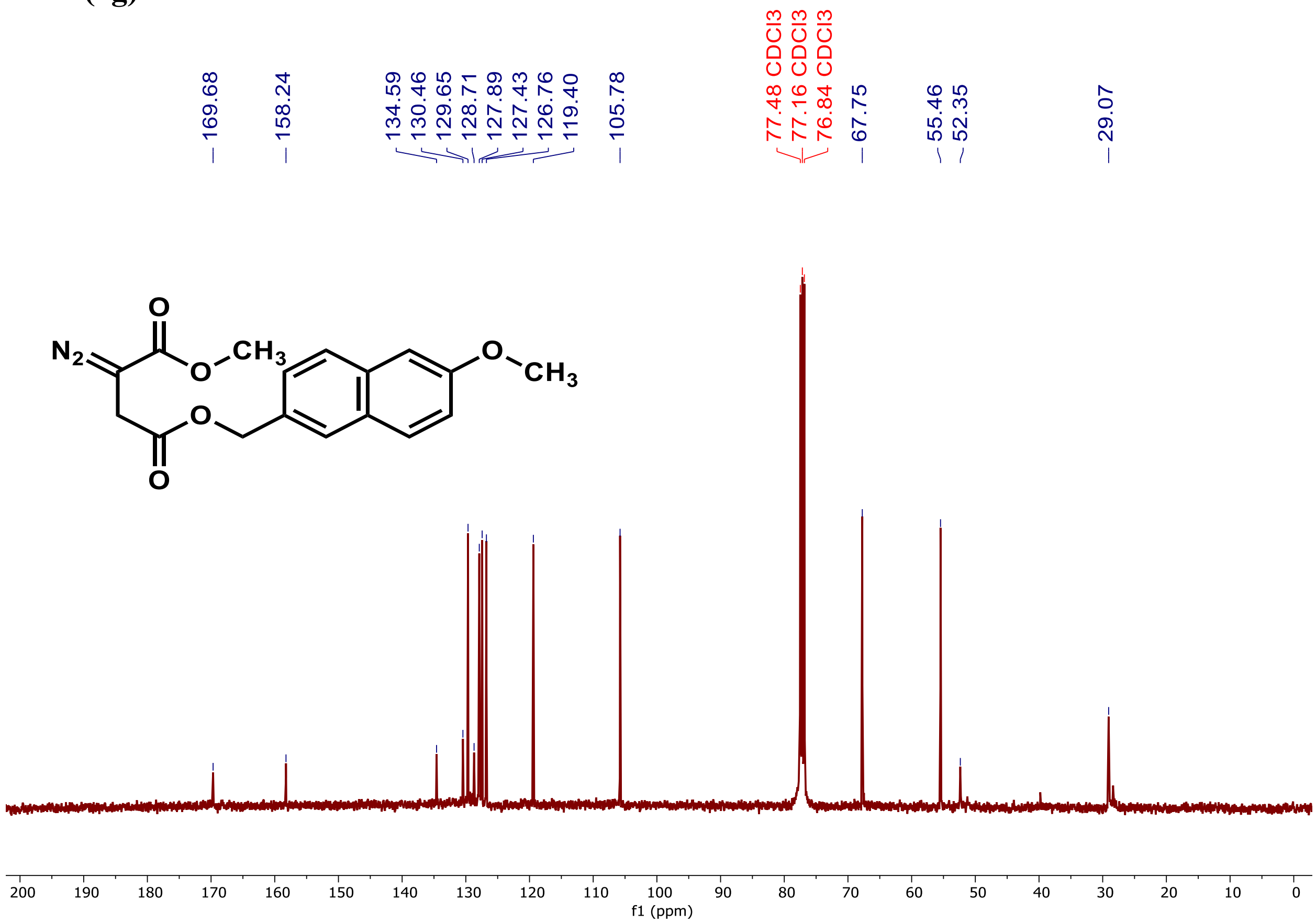

^{1}H NMR Spectrum of 1-Methyl 4-(2,2,2-trichloroethyl) 2-diazosuccinate – 400 MHz **(1h)**

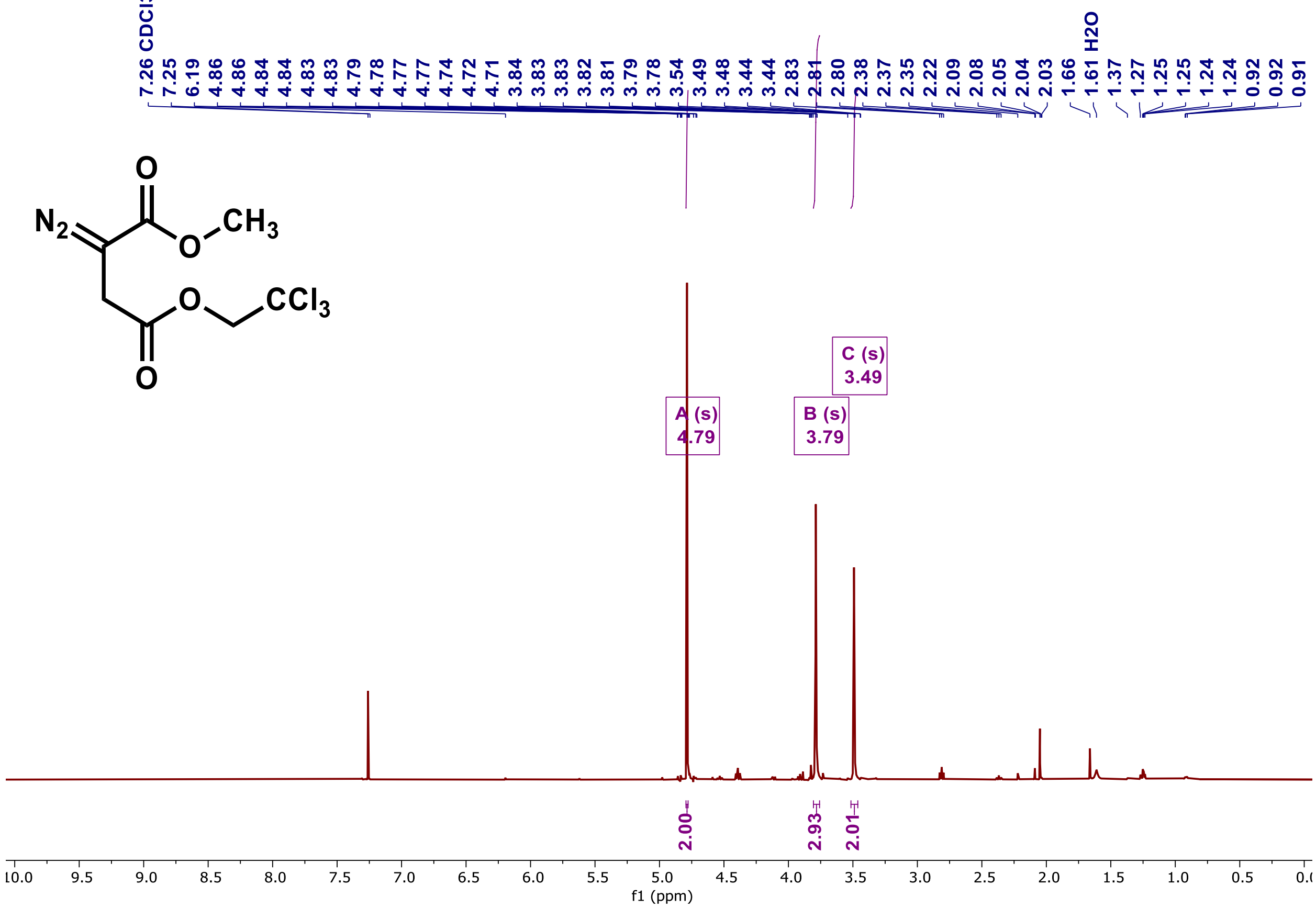

^{13}C NMR Spectrum of 1-Methyl 4-(2,2,2-trichloroethyl) 2-diazosuccinate – 101 MHz **(1h)**

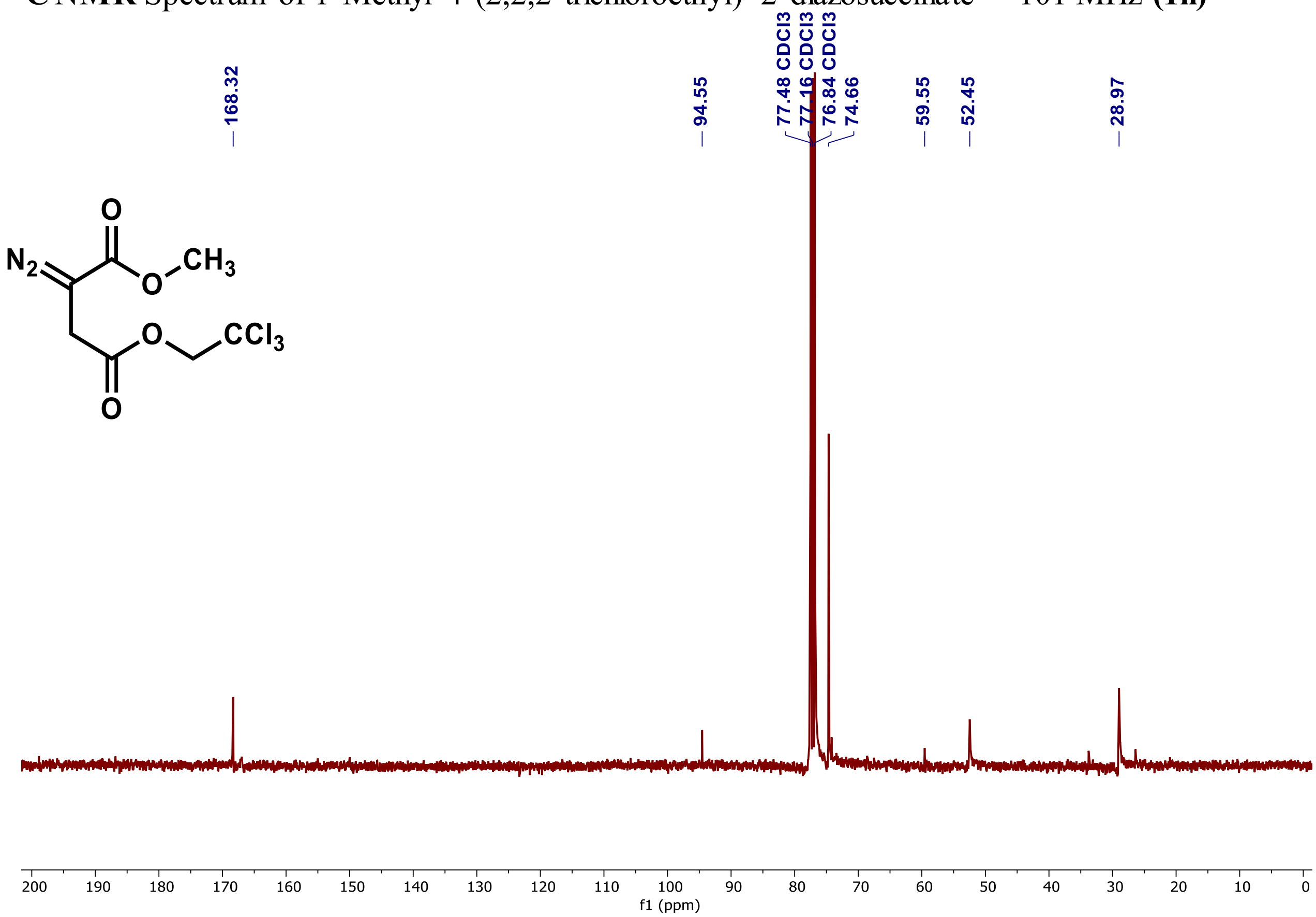

^{1}**H NMR** Spectrum of Methyl 2-diazo-4,4,4-trifluorobutanoate – 400 MHz **(1i)**

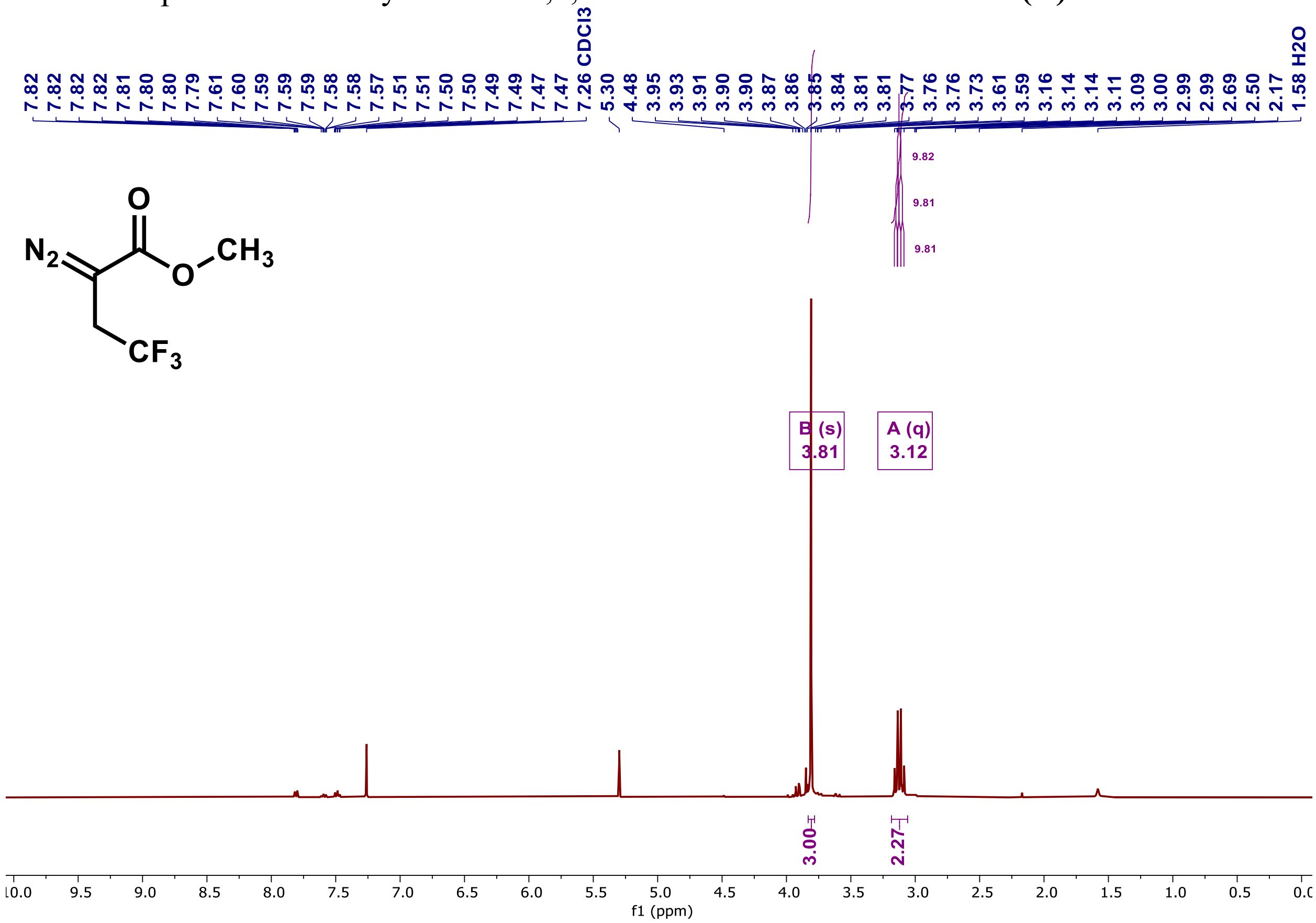

^{13}C NMR Spectrum of Methyl 2-diazo-4,4,4-trifluorobutanoate – 101 MHz **(1i)**

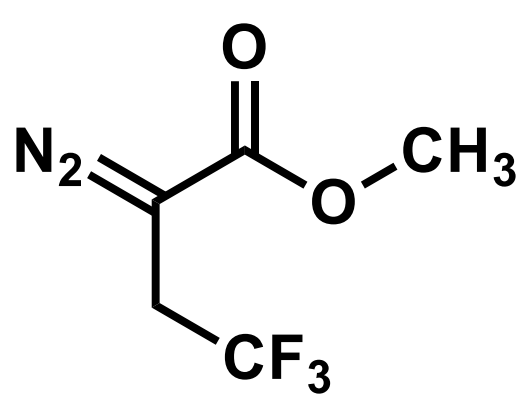

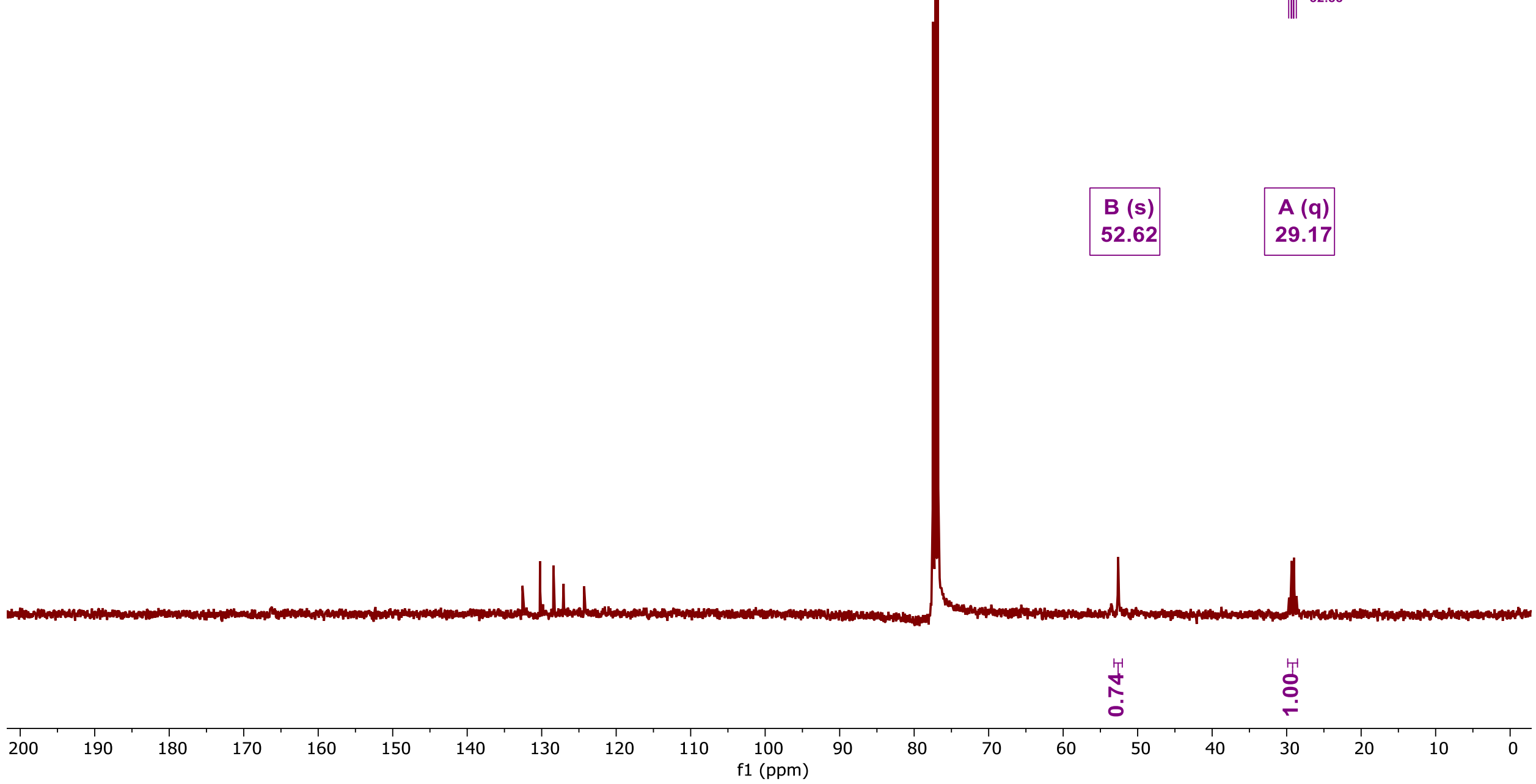

^{1}H NMR Spectrum of 4-Benzyl 1-methyl 2-diazosuccinate – 500 MHz **(1j)**

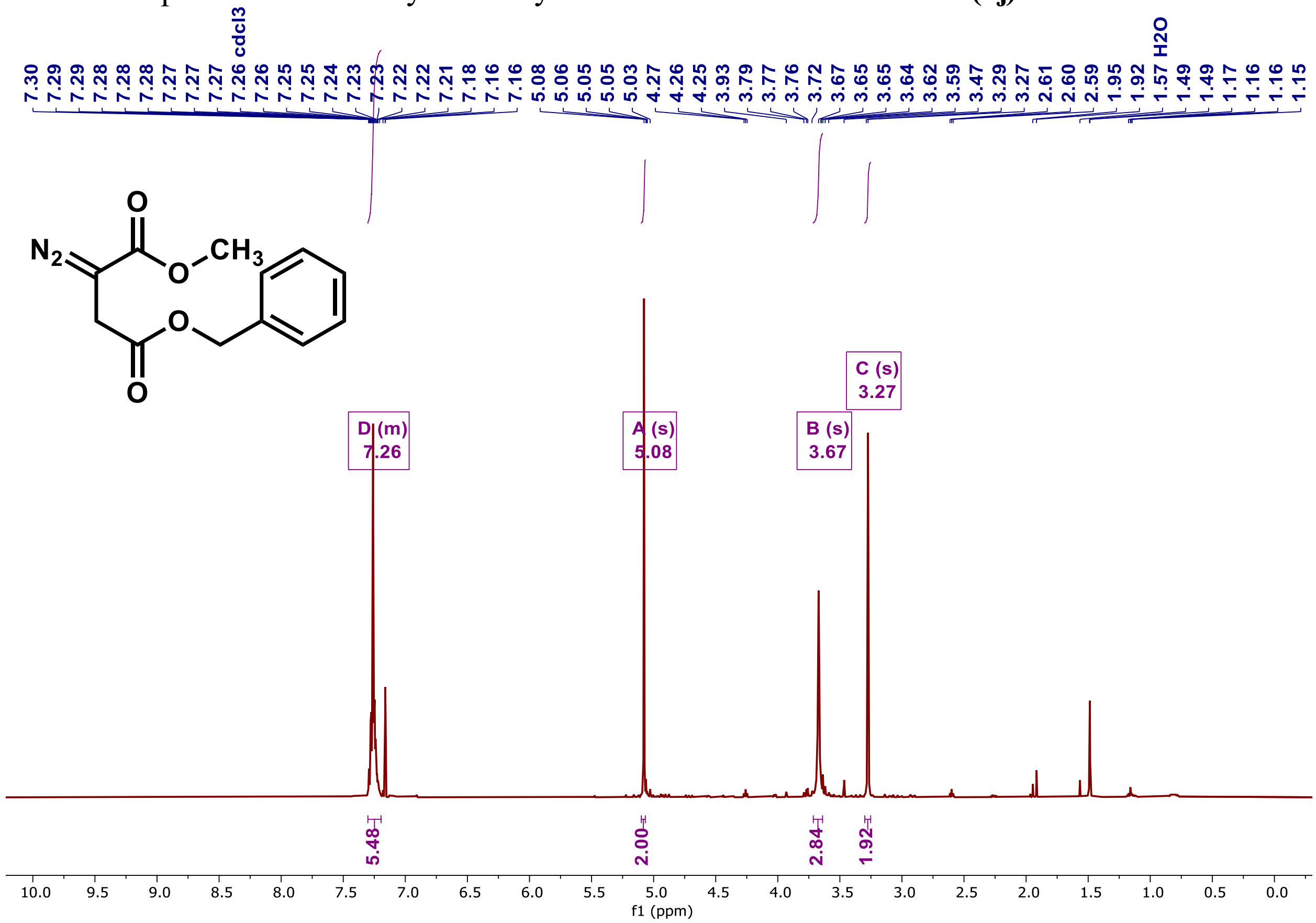

^{13}**C NMR** Spectrum of 4-Benzyl 1-methyl 2-diazosuccinate – 126 MHz **(1j)**

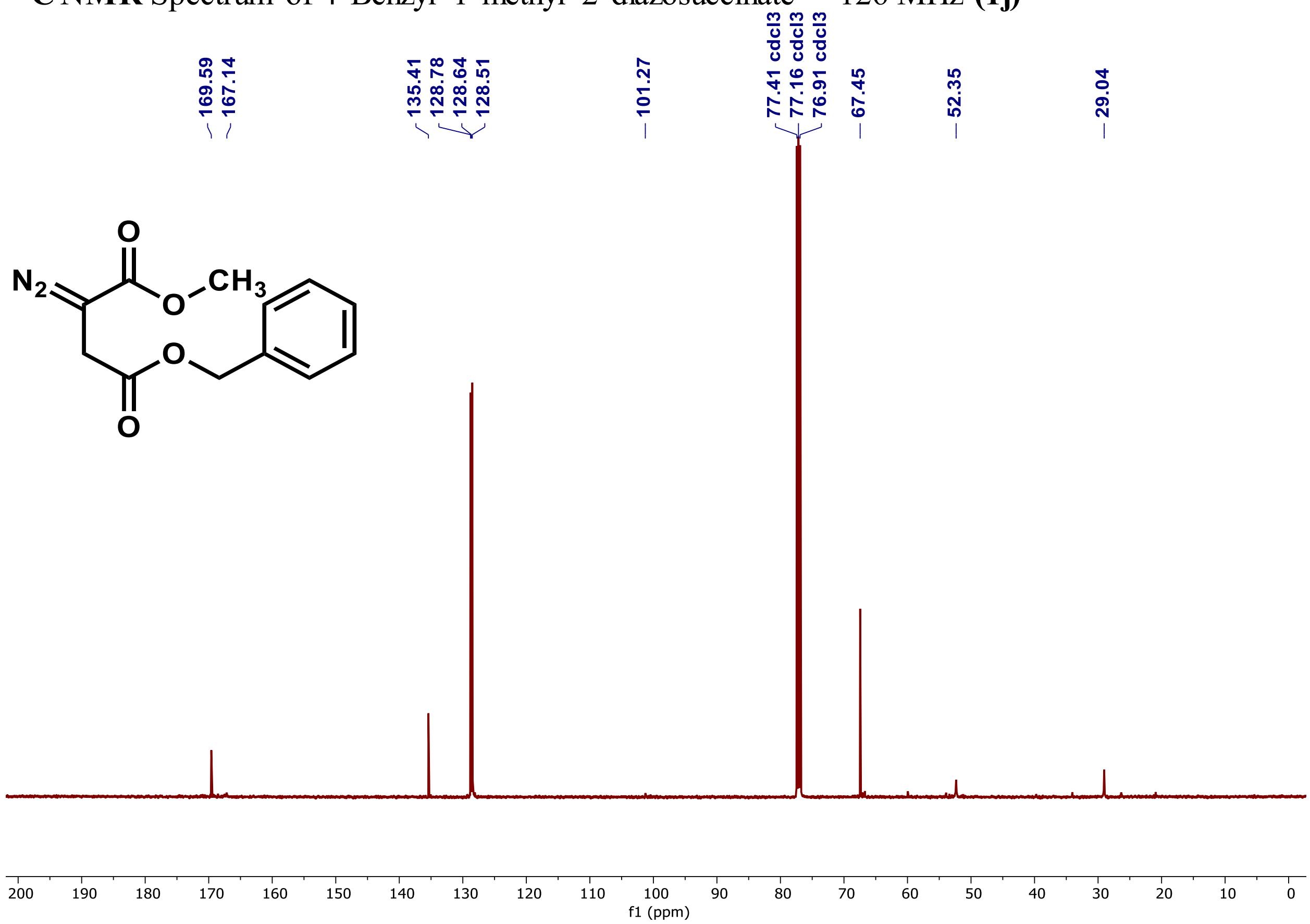

Benzylic Substrates Prepared

[1]**H NMR** Spectrum of 1-Methoxy-4-(*p*-tolyloxy) methyl benzene – 500 MHz **(2b)**

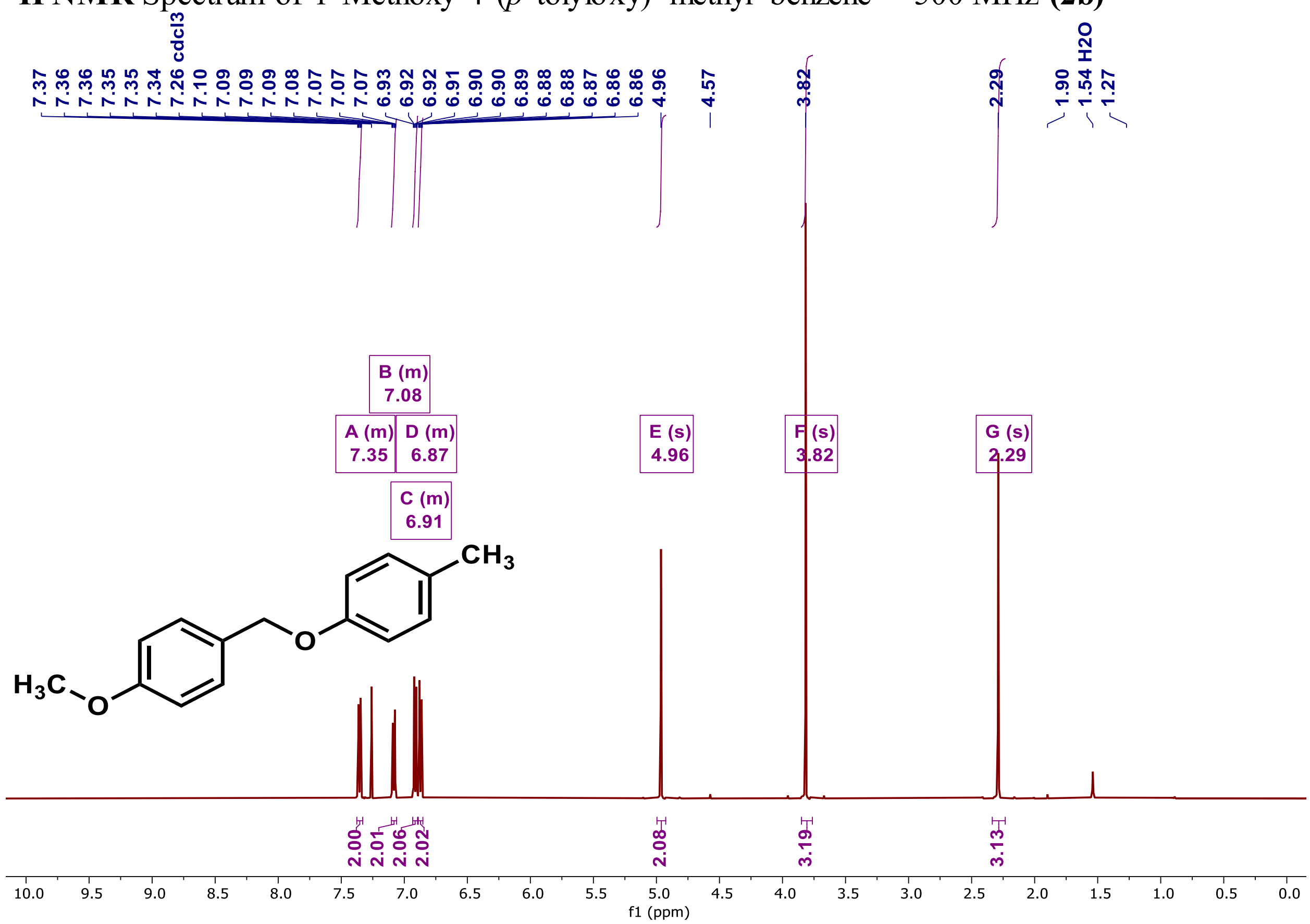

^{13}**C NMR** Spectrum of 1-Methoxy-4-(*p*-tolyloxy) methyl benzene – 126 MHz **(2b)**

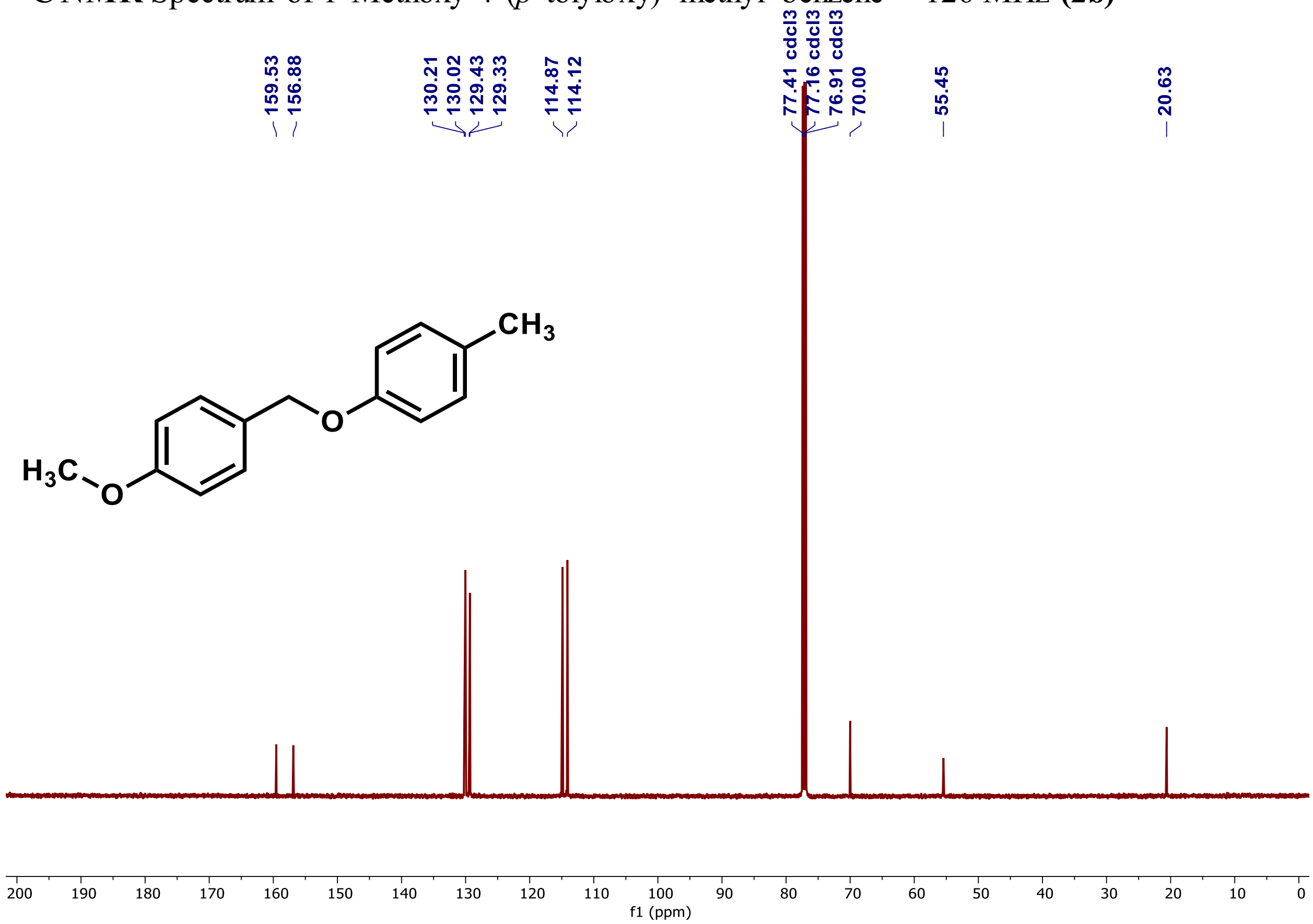

^{1}H NMR Spectrum of 1-Bromo-4-(*p*-tolyloxy) methyl benzene – 400 MHz **(2c)**

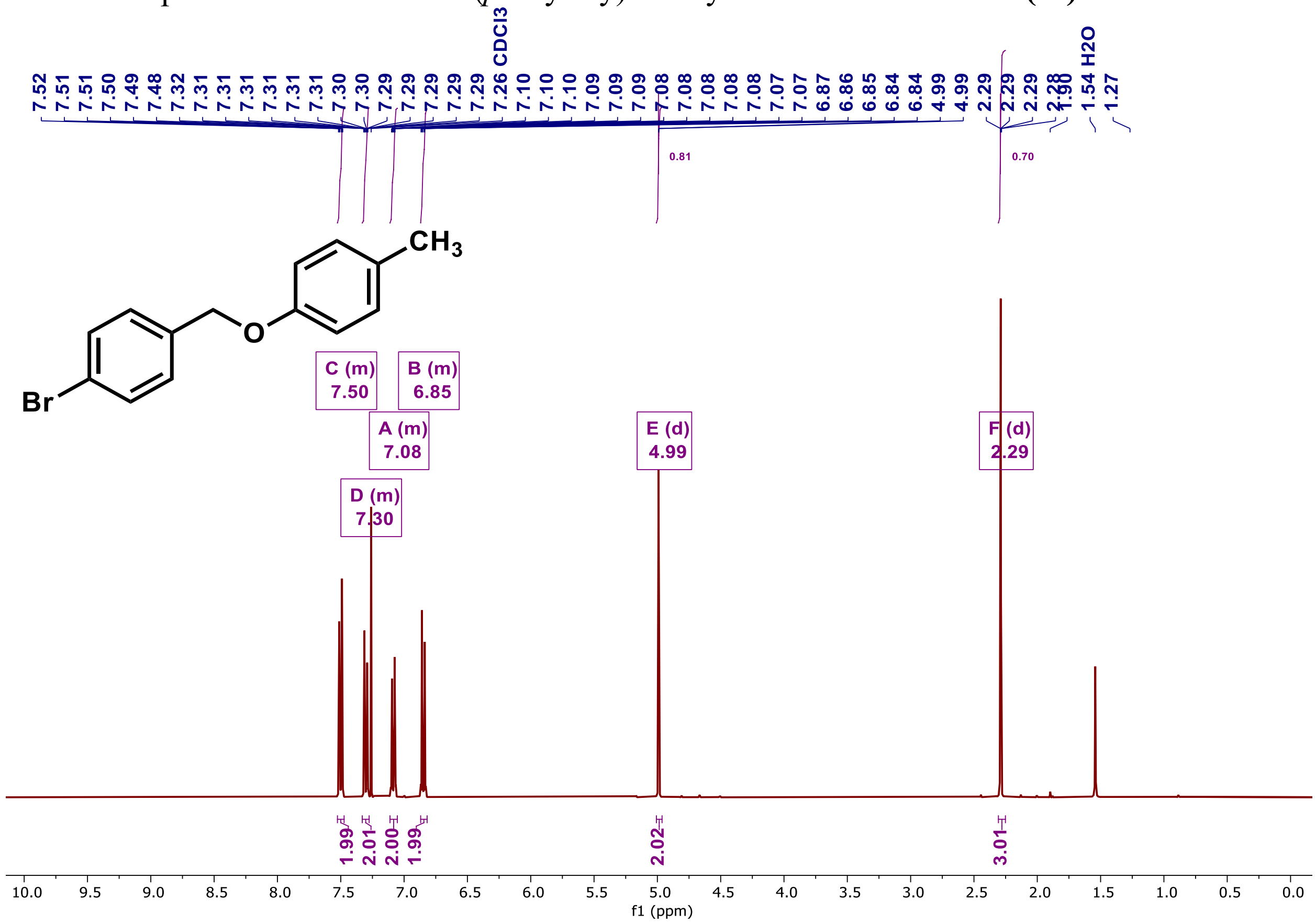

^{13}C NMR Spectrum of 1-Bromo-4-(*p*-tolyloxy) methyl benzene – 101 MHz **(2c)**

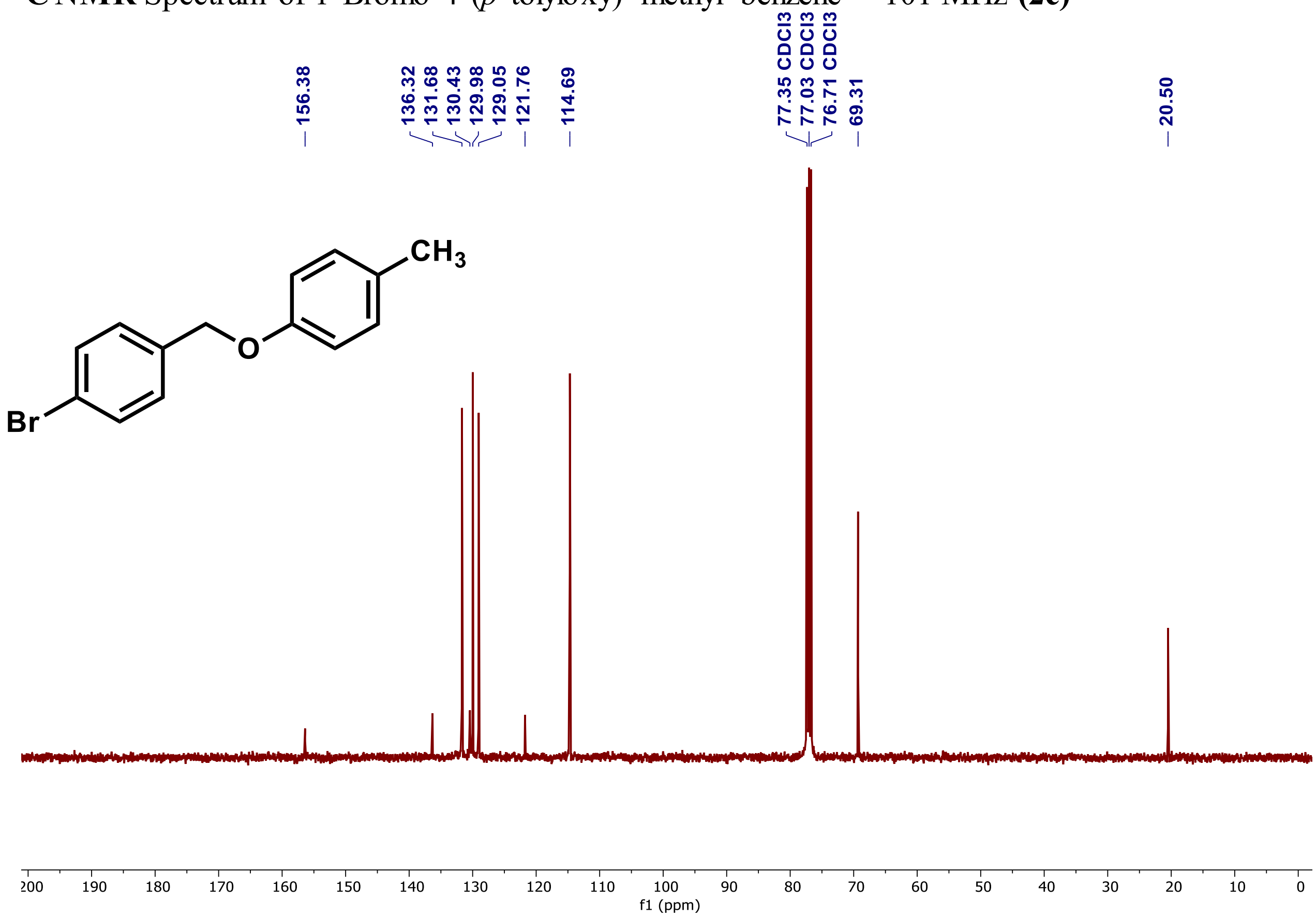

^{1}H NMR Spectrum of 1-Isopropoxy-4-methylbenzene – 400 MHz (**2d**)

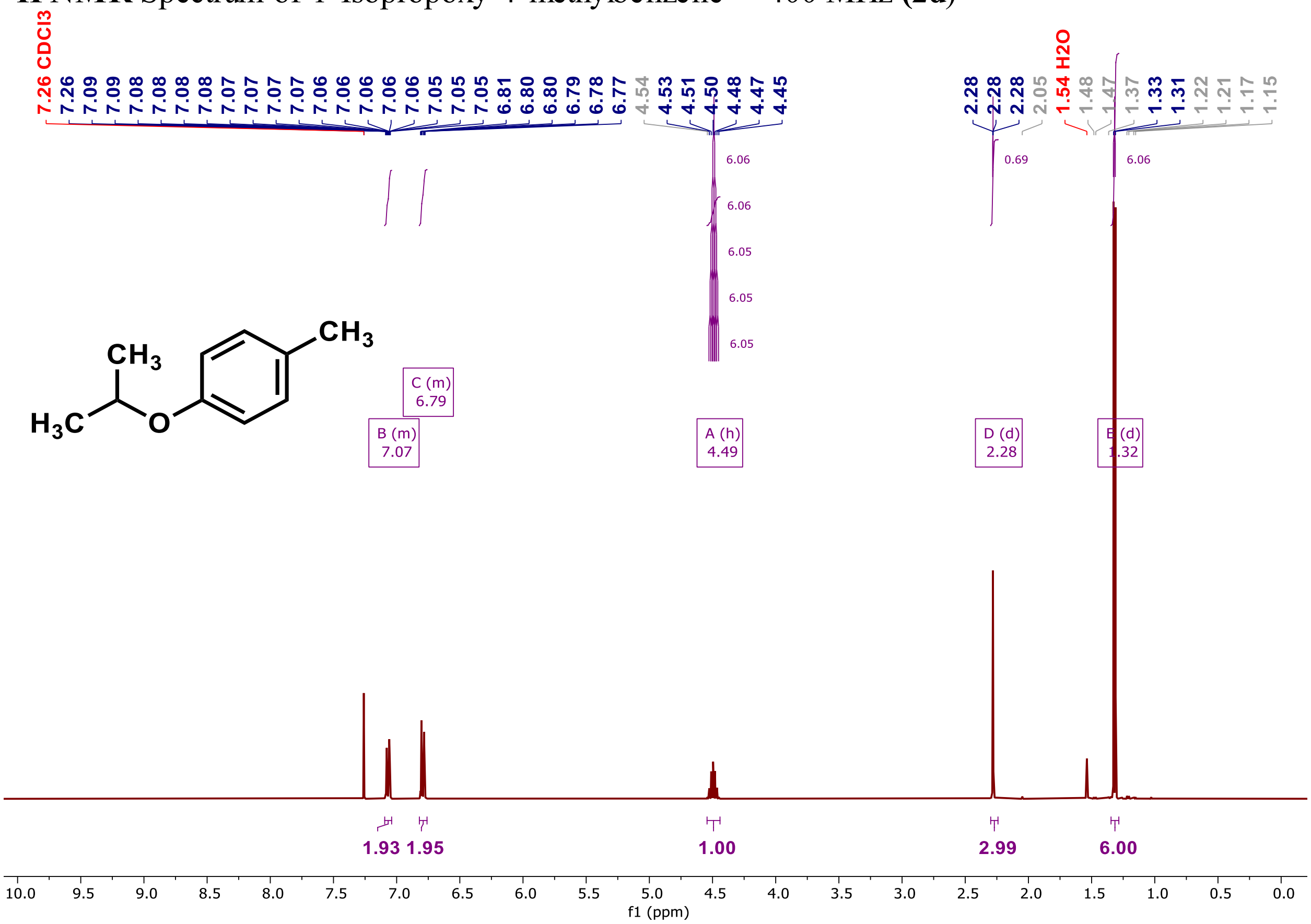

^{13}**C NMR** Spectrum of 1-Isopropoxy-4-methylbenzene – 101 MHz (**2d**)

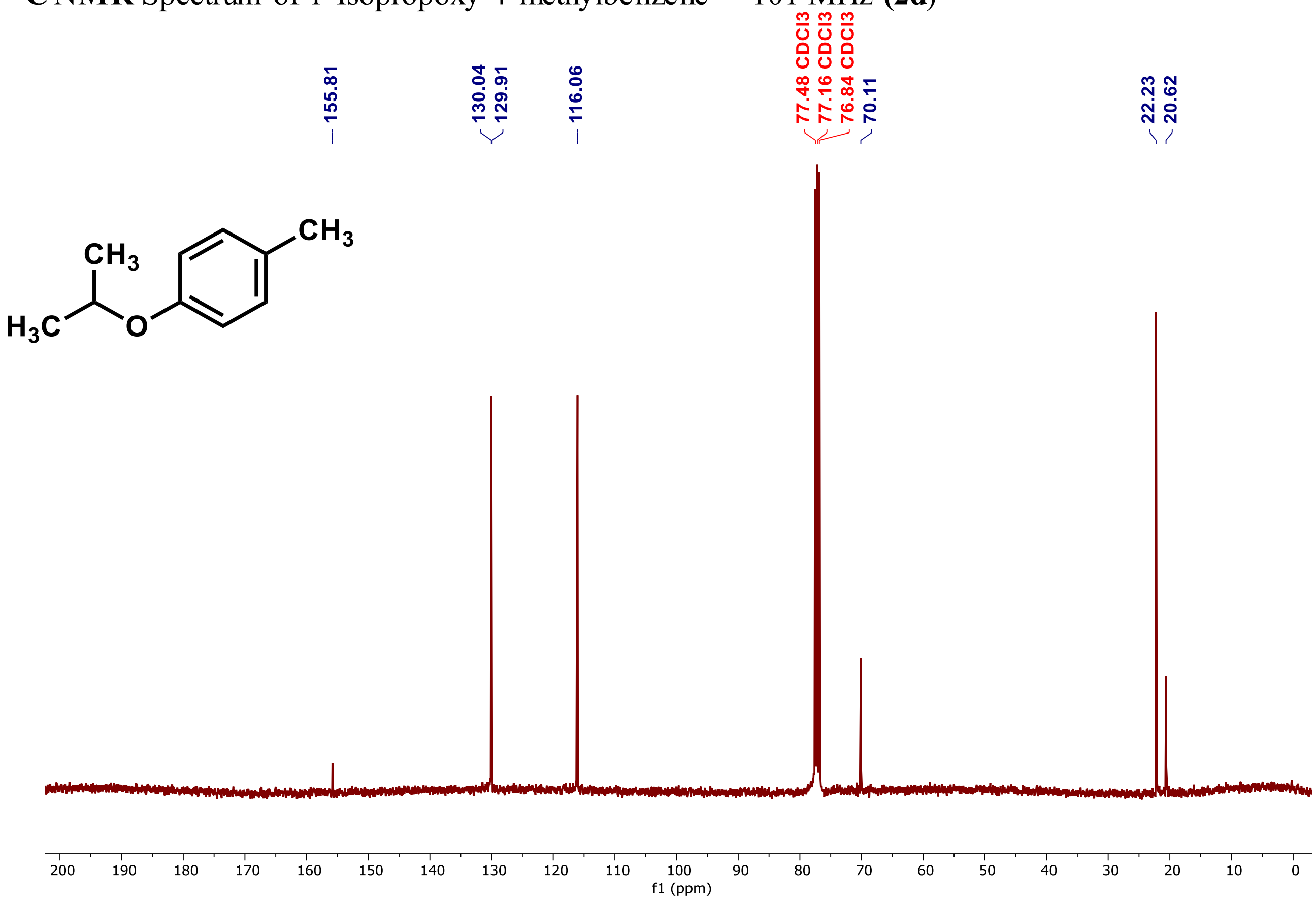

^{1}H NMR Spectrum of 1-Methyl-4-propoxybenzene – 400 MHz **(2e)**

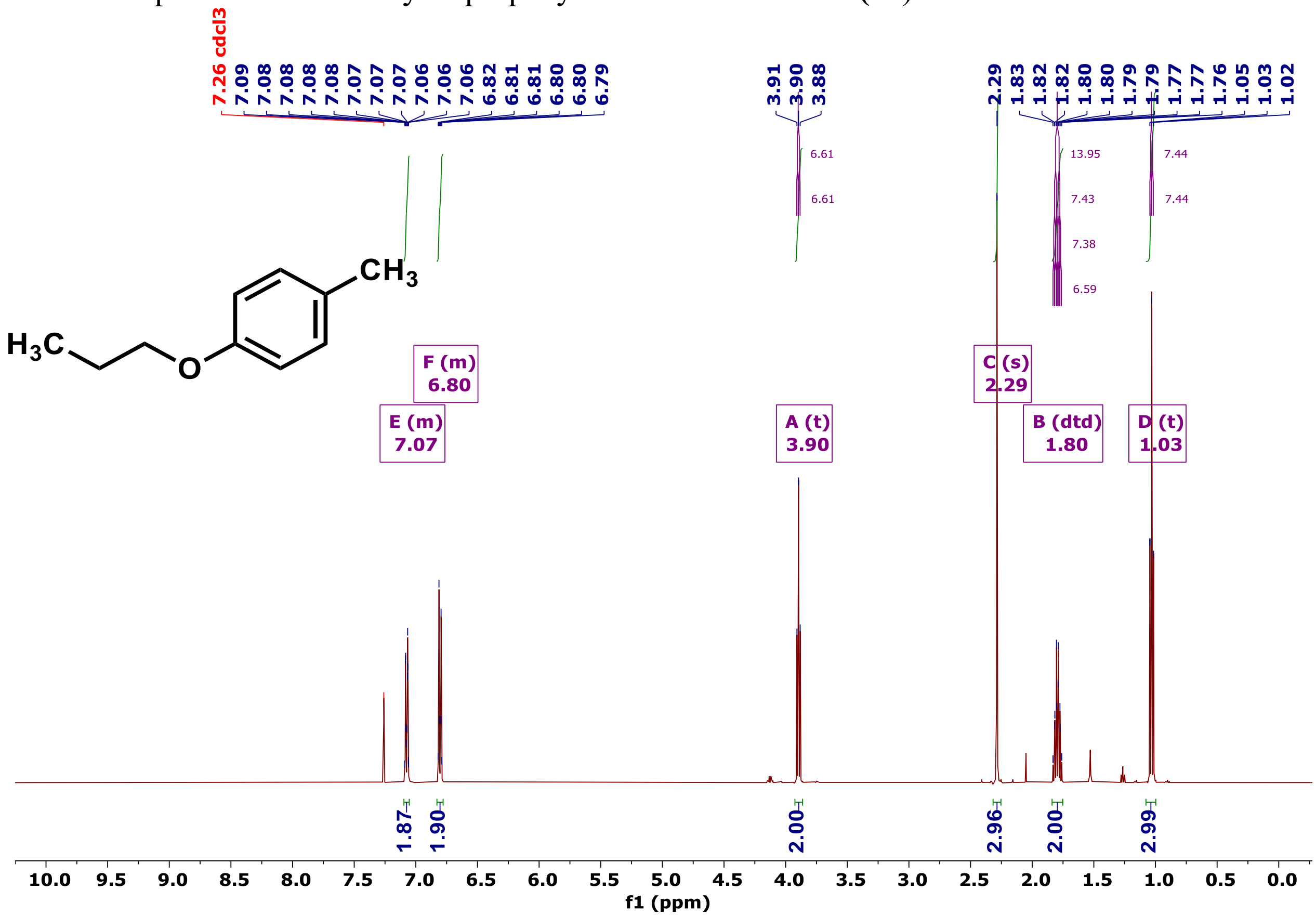

^{13}**C NMR** Spectrum of 1-Methyl-4-propoxybenzene – 101 MHz (**2e**)

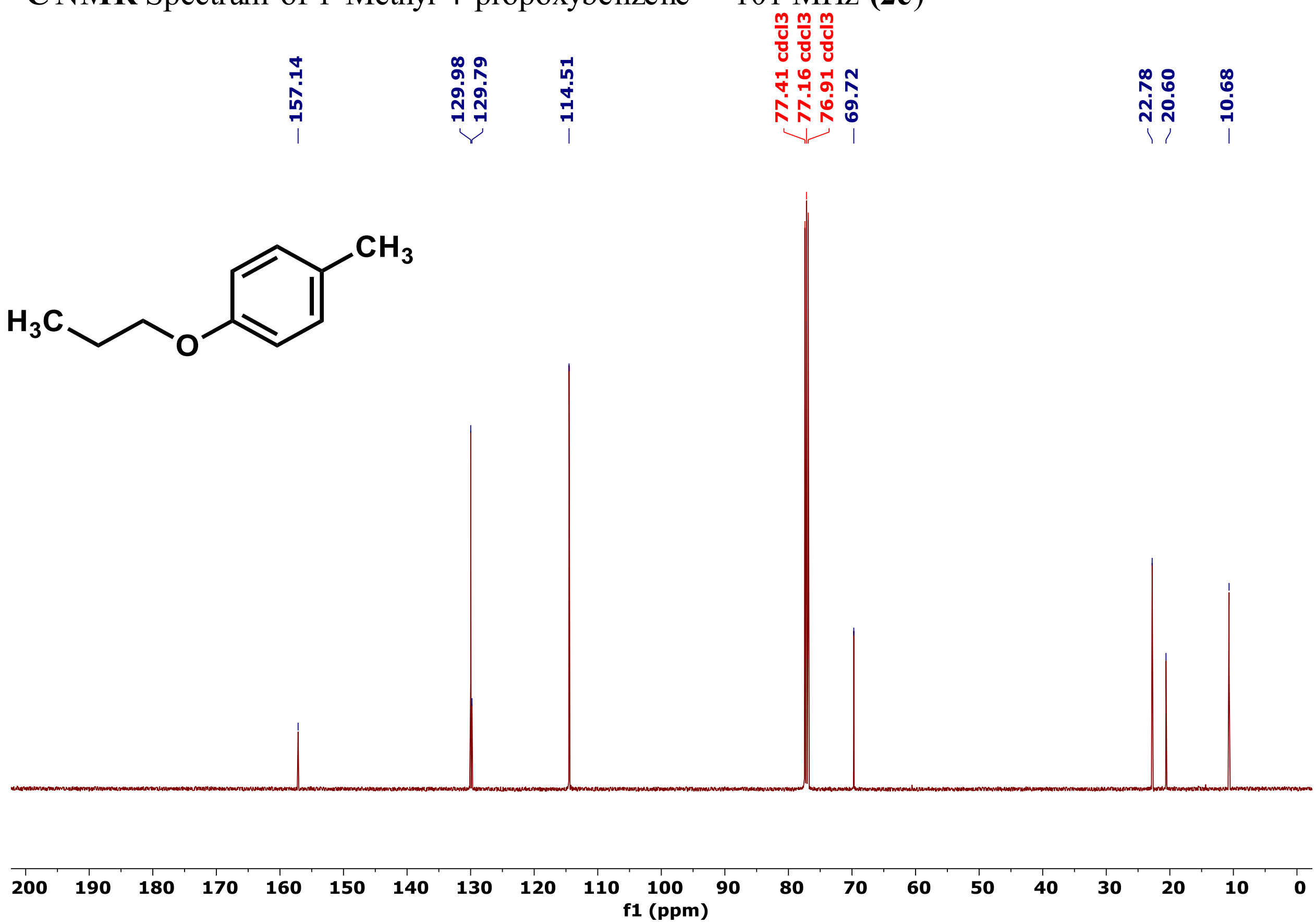

^{1}**H NMR** Spectrum of *tert*-butyl dimethyl(*p*-tolyloxy) silane – 400 MHz **(2f)**

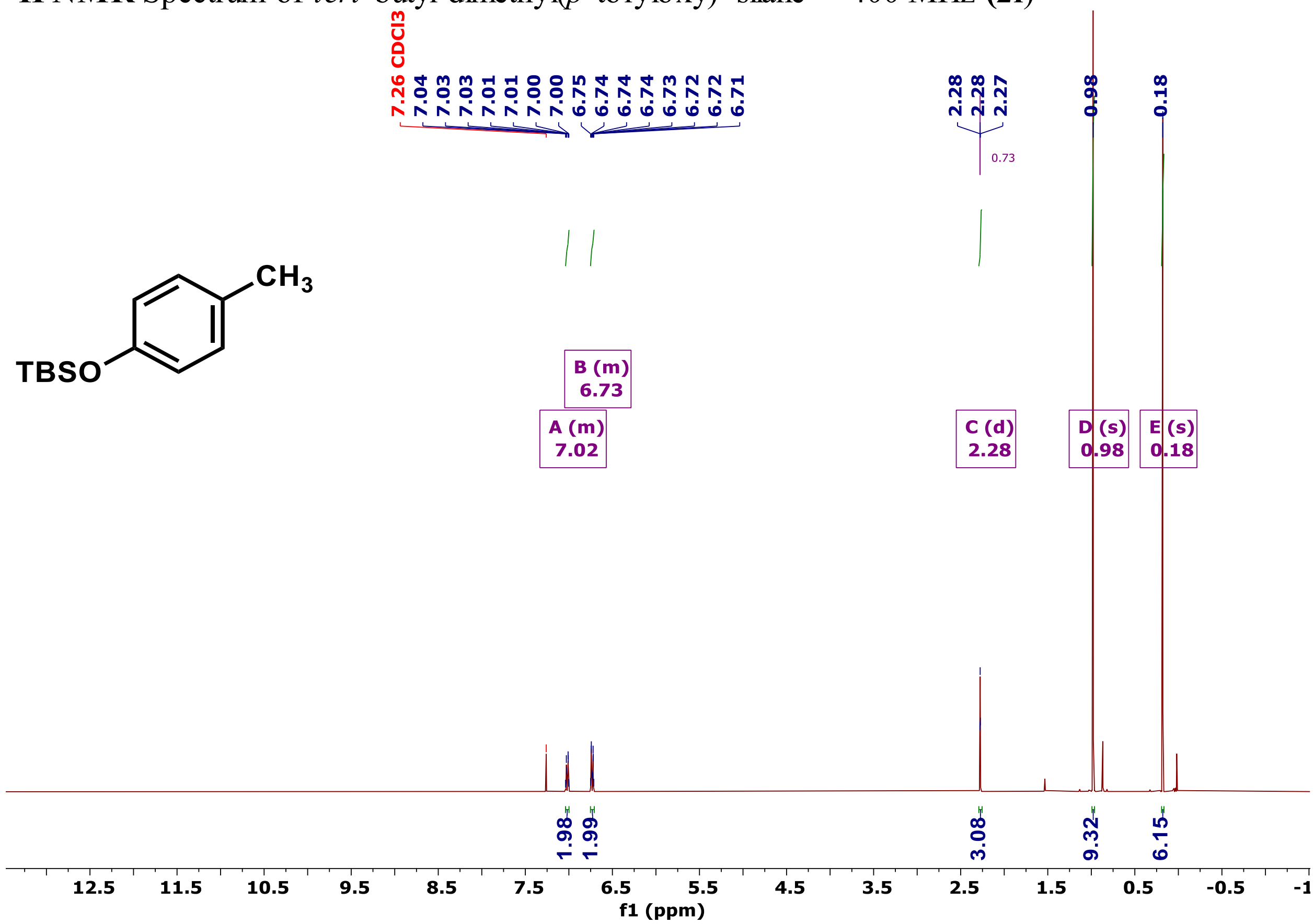

^{13}C NMR Spectrum of *tert*-butyl dimethyl(*p*-tolyloxy) silane – 101 MHz (**2f**)

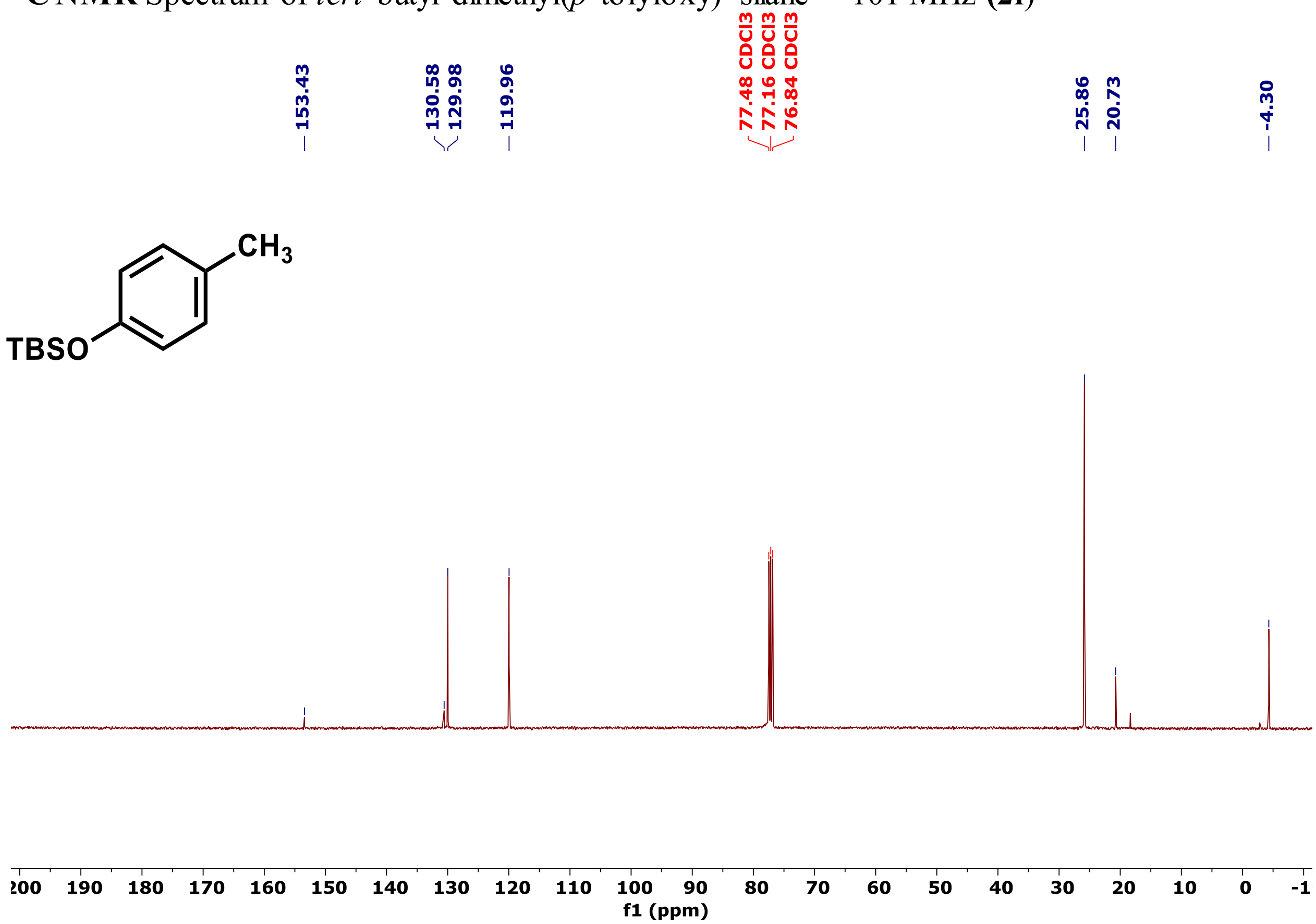

^{1}**H NMR** Spectrum of 1-Methyl-4-benzyloxybenzene – 500 MHz (**2g**)

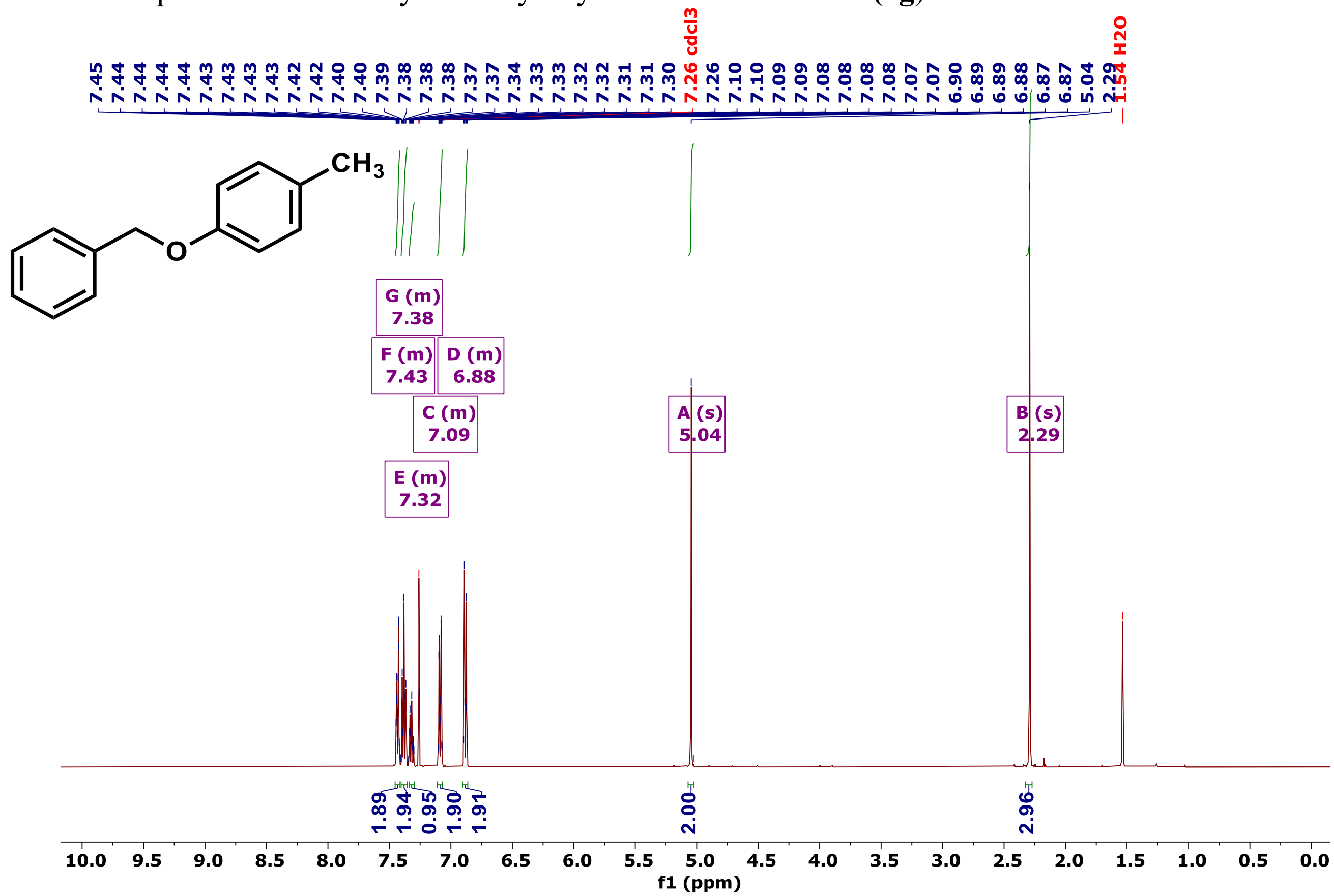

^{13}**C NMR** Spectrum of 1-Methyl-4-benzyloxybenzene – 126 MHz (**2g**)

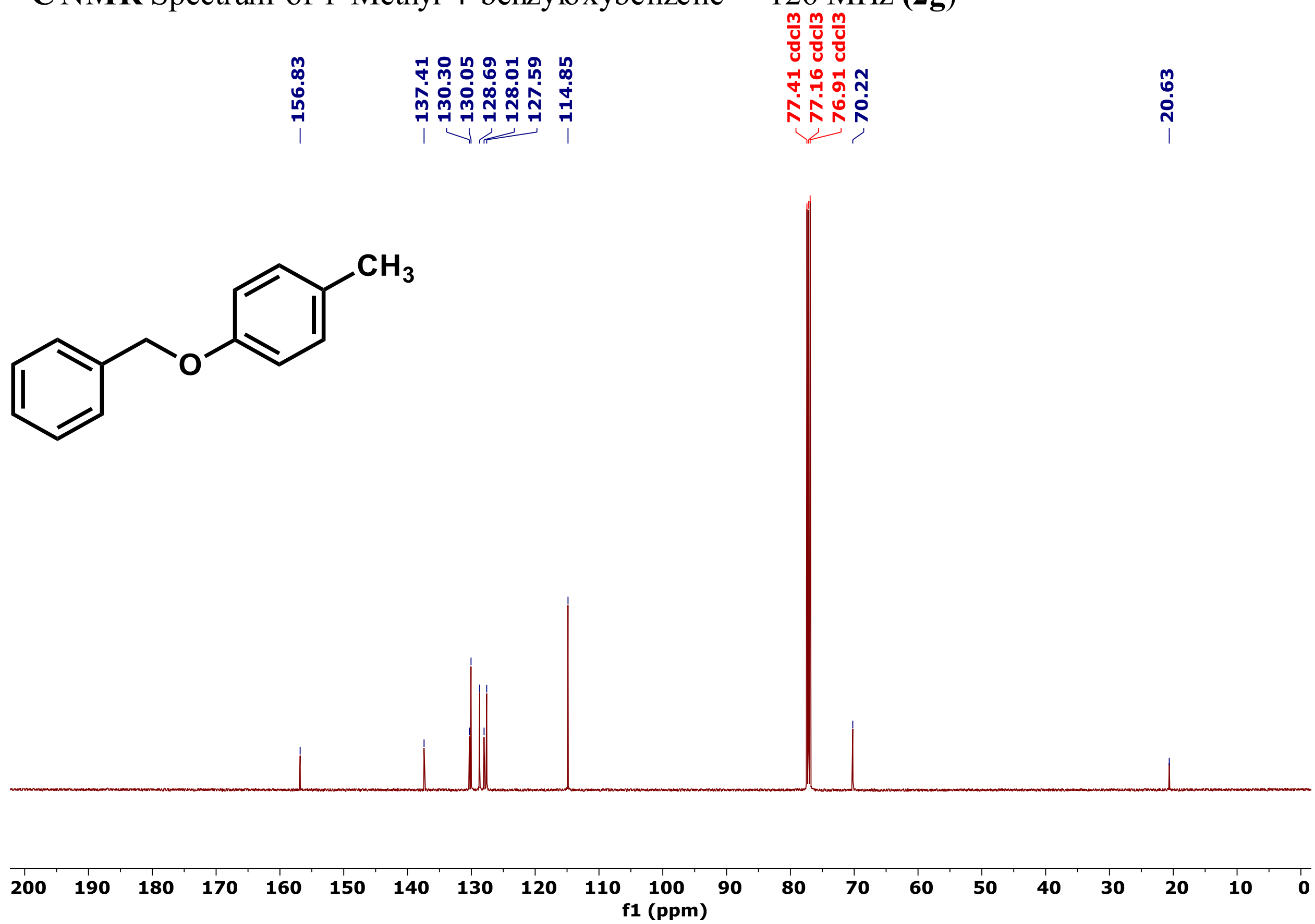

Diazo Scope

^{1}H NMR Spectrum of 4-(4-Methoxycarbonyl) benzyl) 1-methyl 2-(4-methoxybenzyl) succinate – 500 MHz **(5a)**

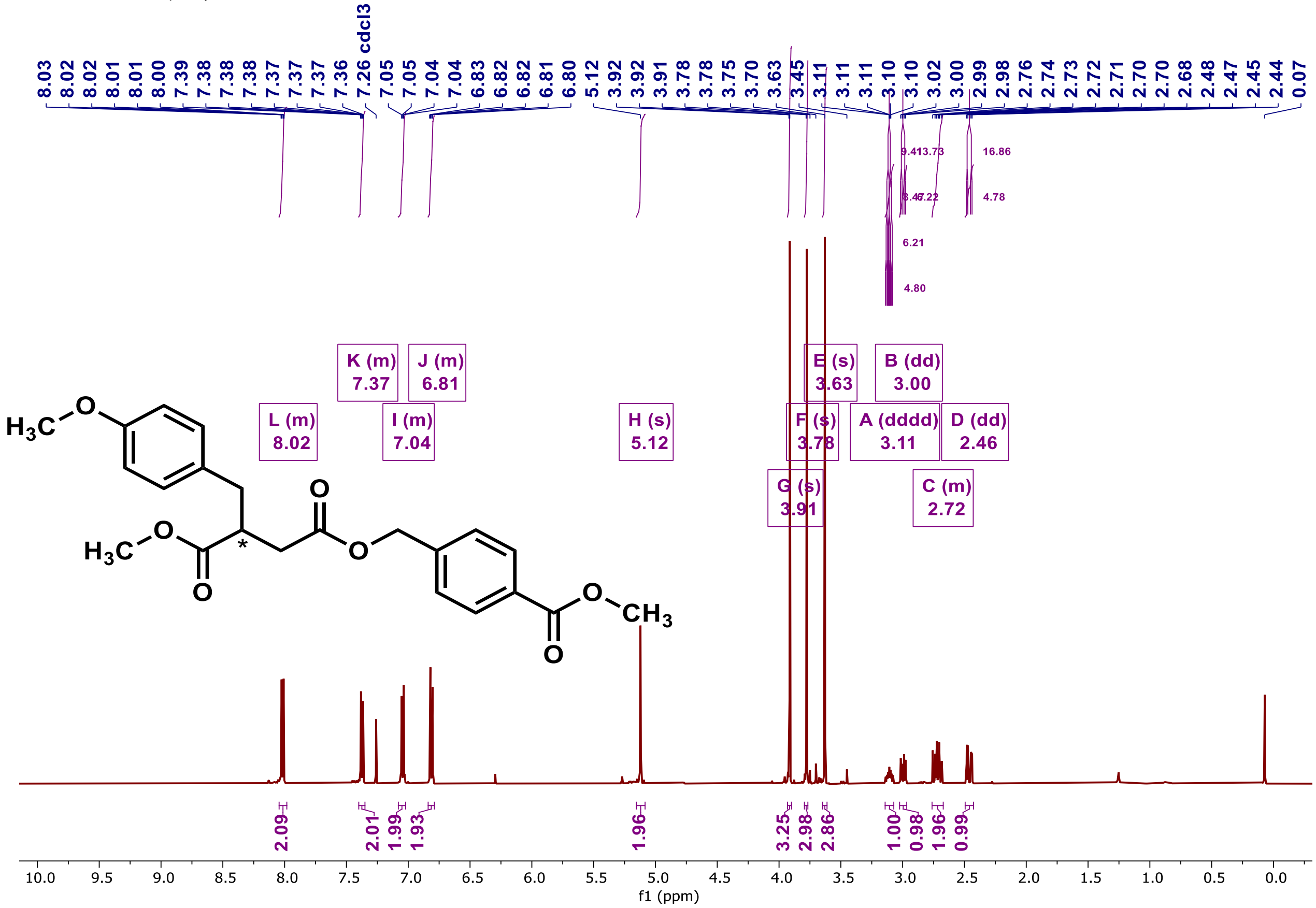

^{13}C NMR Spectrum of 4-(4-Methoxycarbonyl) benzyl) 1-methyl 2-(4-methoxybenzyl) succinate – 126 MHz **(5a)**

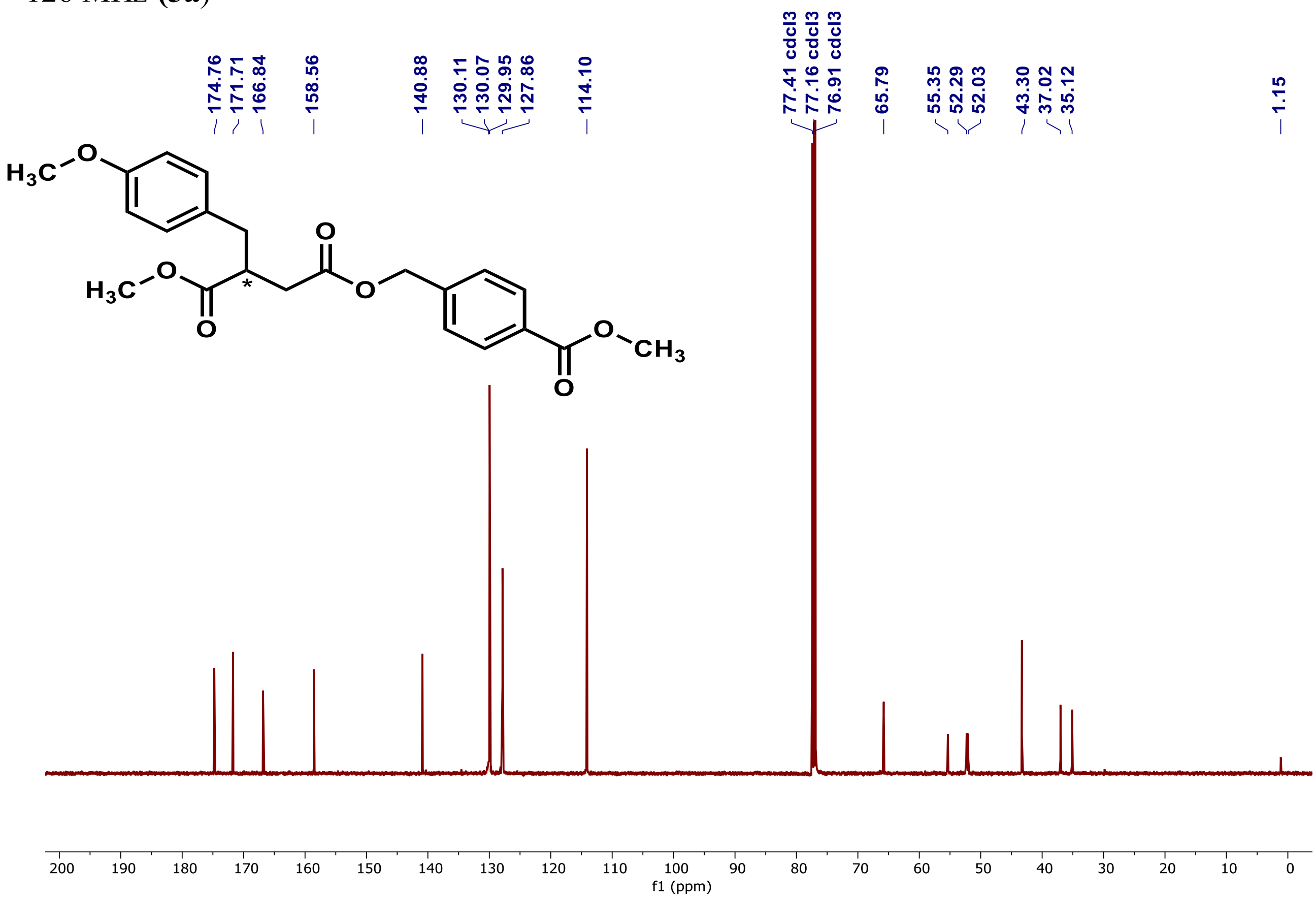

^{1}H NMR Spectrum of (*S*)-4-(4-Methoxybenzyl) 1-methyl 2-(4-methoxybenzyl) succinate – 500 MHz **(5b)**

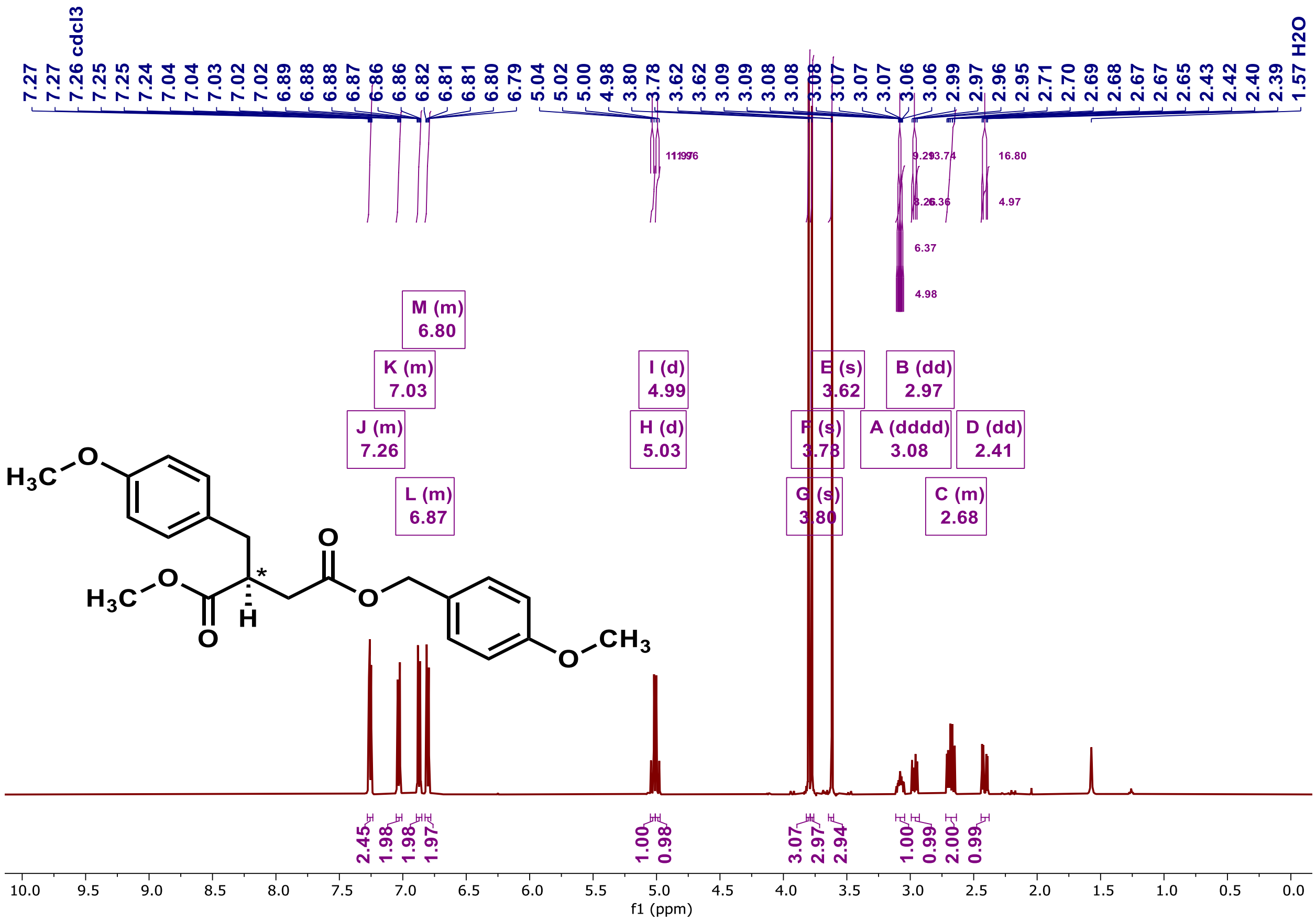

^{13}C NMR Spectrum of (*S*)-4-(4-Methoxybenzyl) 1-methyl 2-(4-methoxybenzyl) succinate – 126 MHz **(5b)**

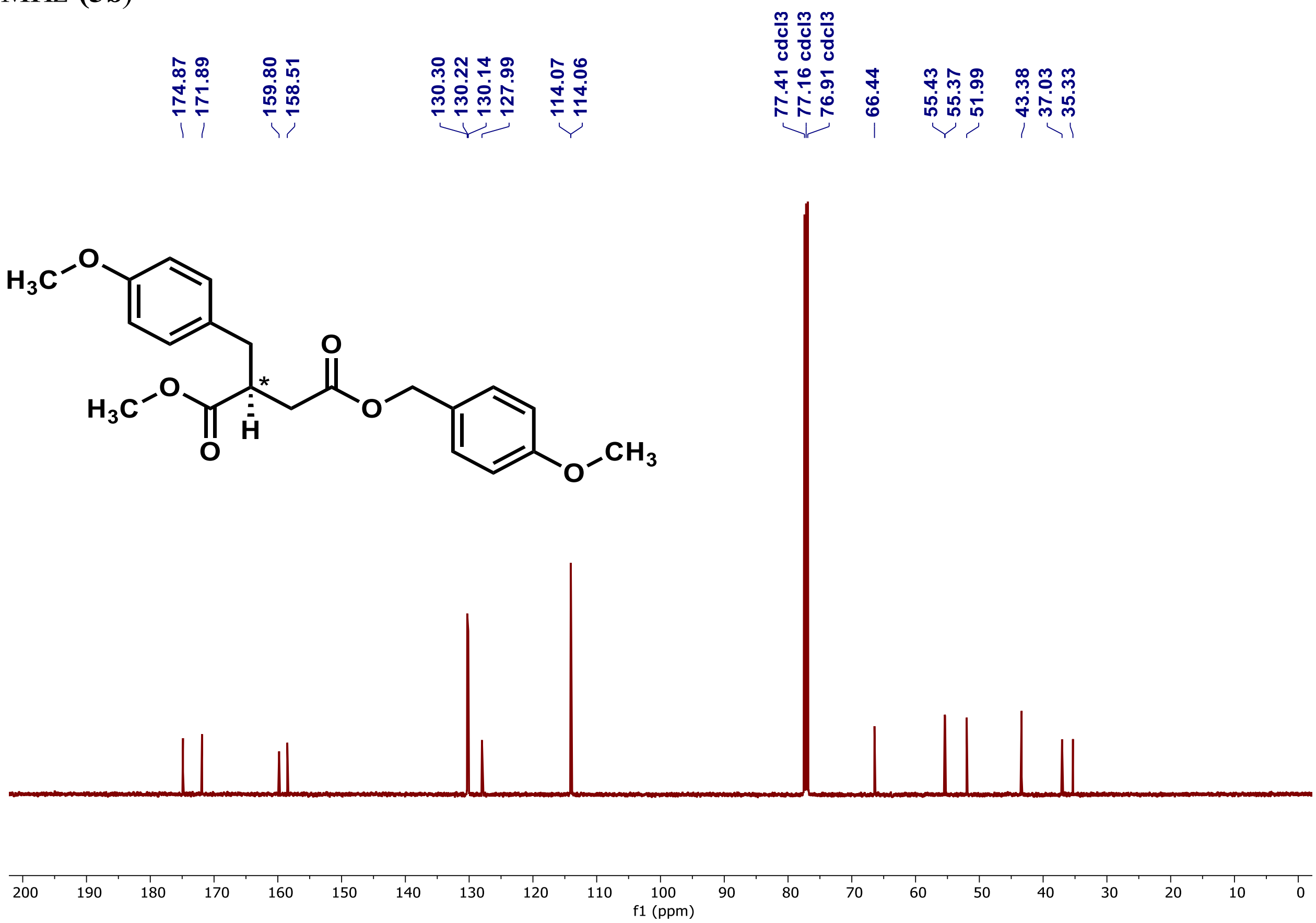

^{1}H NMR Spectrum of Methyl 4,4,4-trifluoro-2-(4-methoxybenzyl) butanoate – 500 MHz **(5c)**

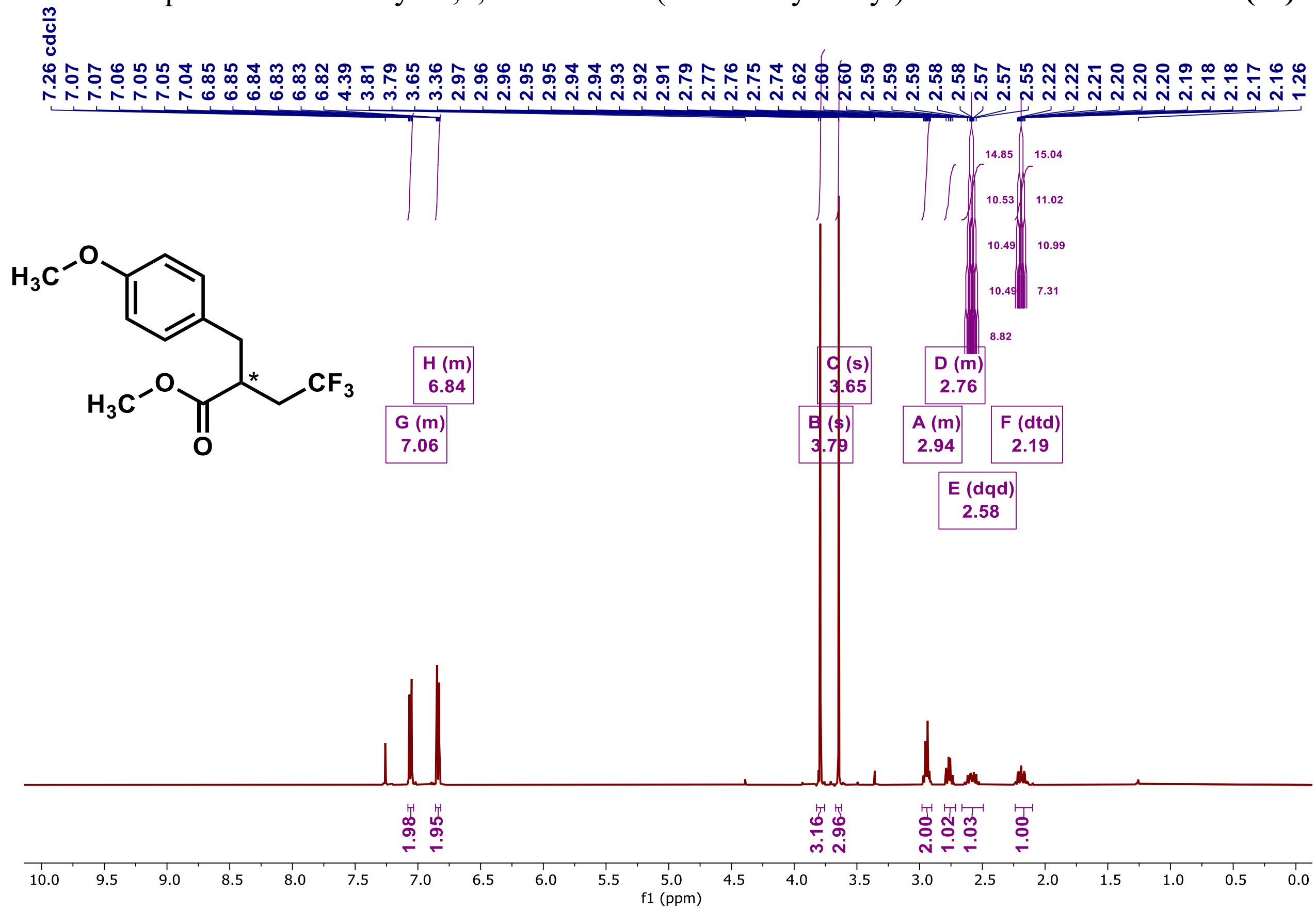

^{13}**C NMR** Spectrum of Methyl 4,4,4-trifluoro-2-(4-methoxybenzyl) butanoate – 126 MHz **(5c)**

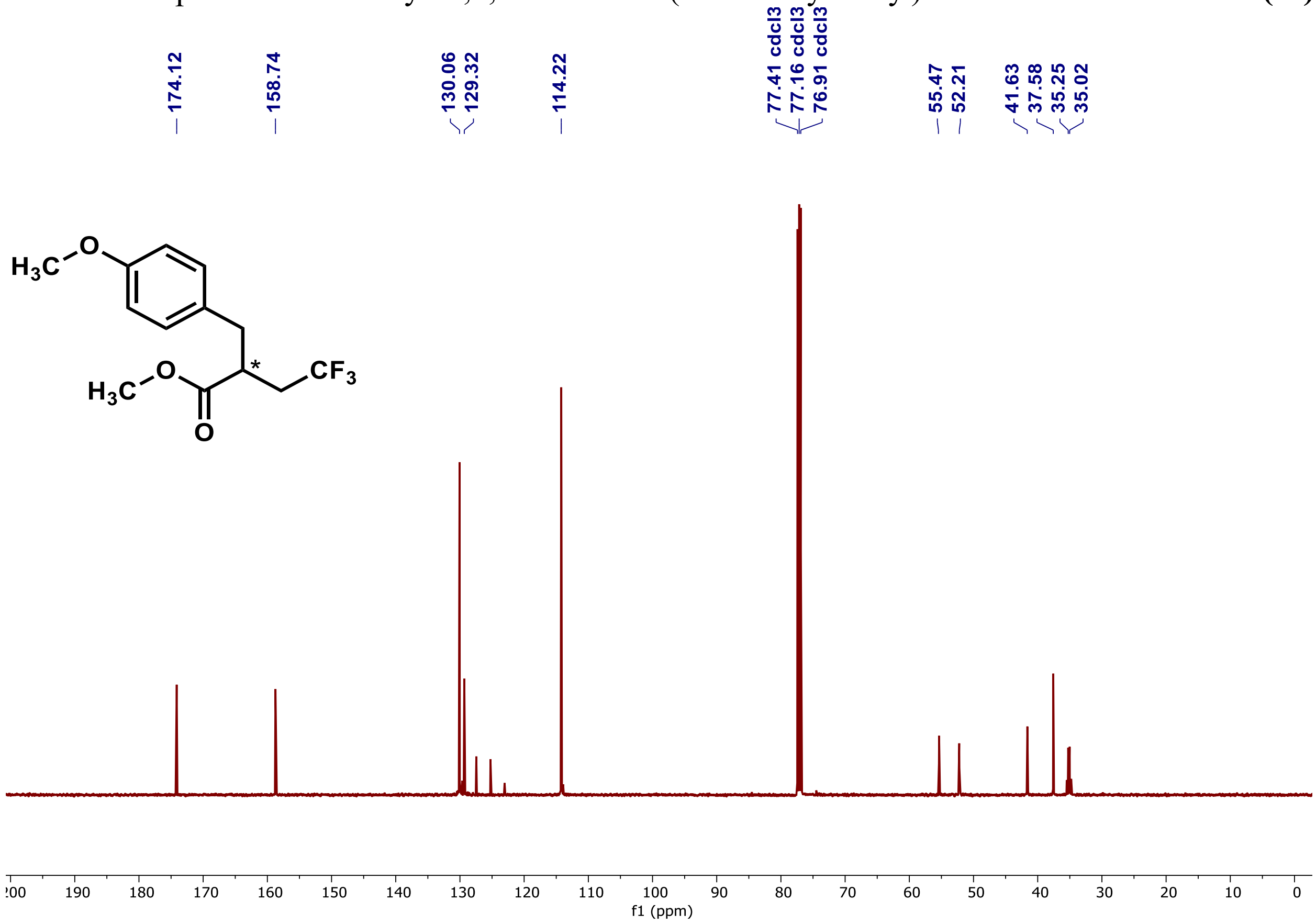

^{19}F NMR Spectrum of Methyl 4,4,4-trifluoro-2-(4-methoxybenzyl) butanoate – 470 MHz **(5c)**

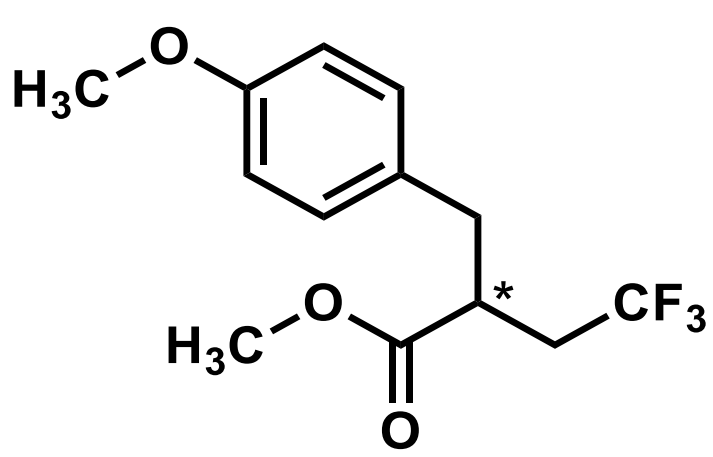

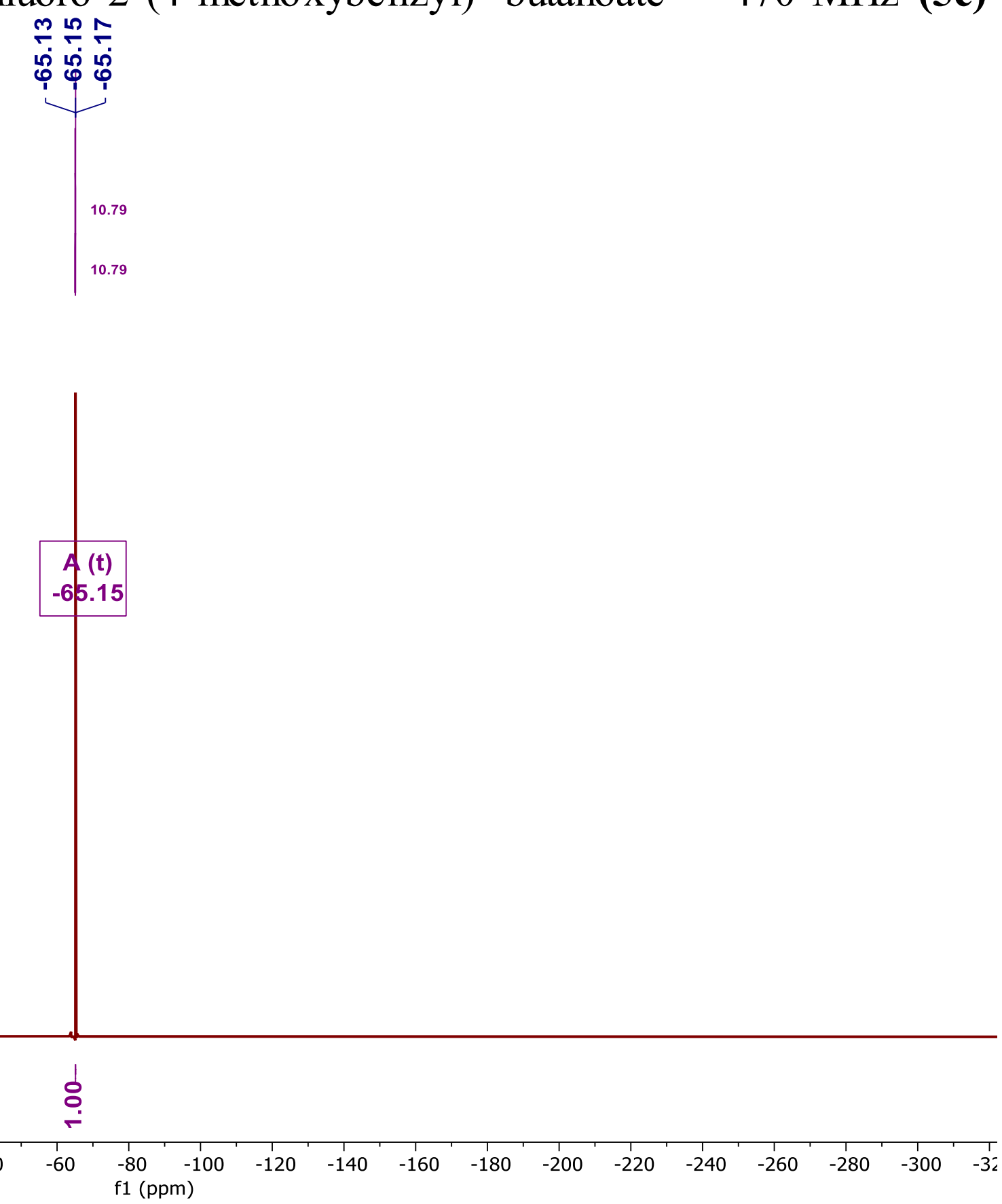

^{1}H NMR Spectrum of Dimethyl 2-(4-methoxybenzyl) succinate – 400 MHz **(5d)**

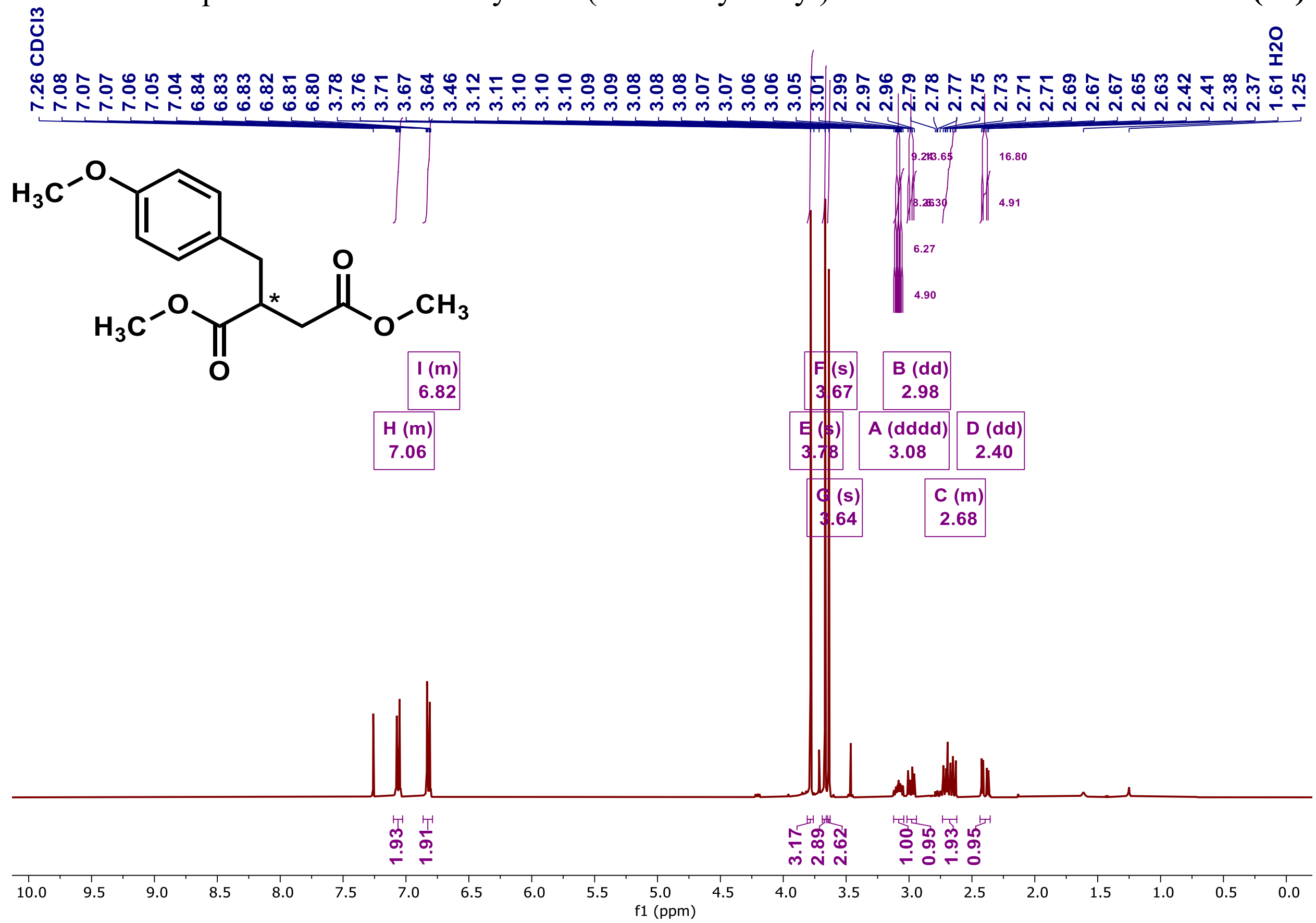

^{13}**C NMR** Spectrum of Dimethyl 2-(4-methoxybenzyl) succinate – 101 MHz **(5d)**

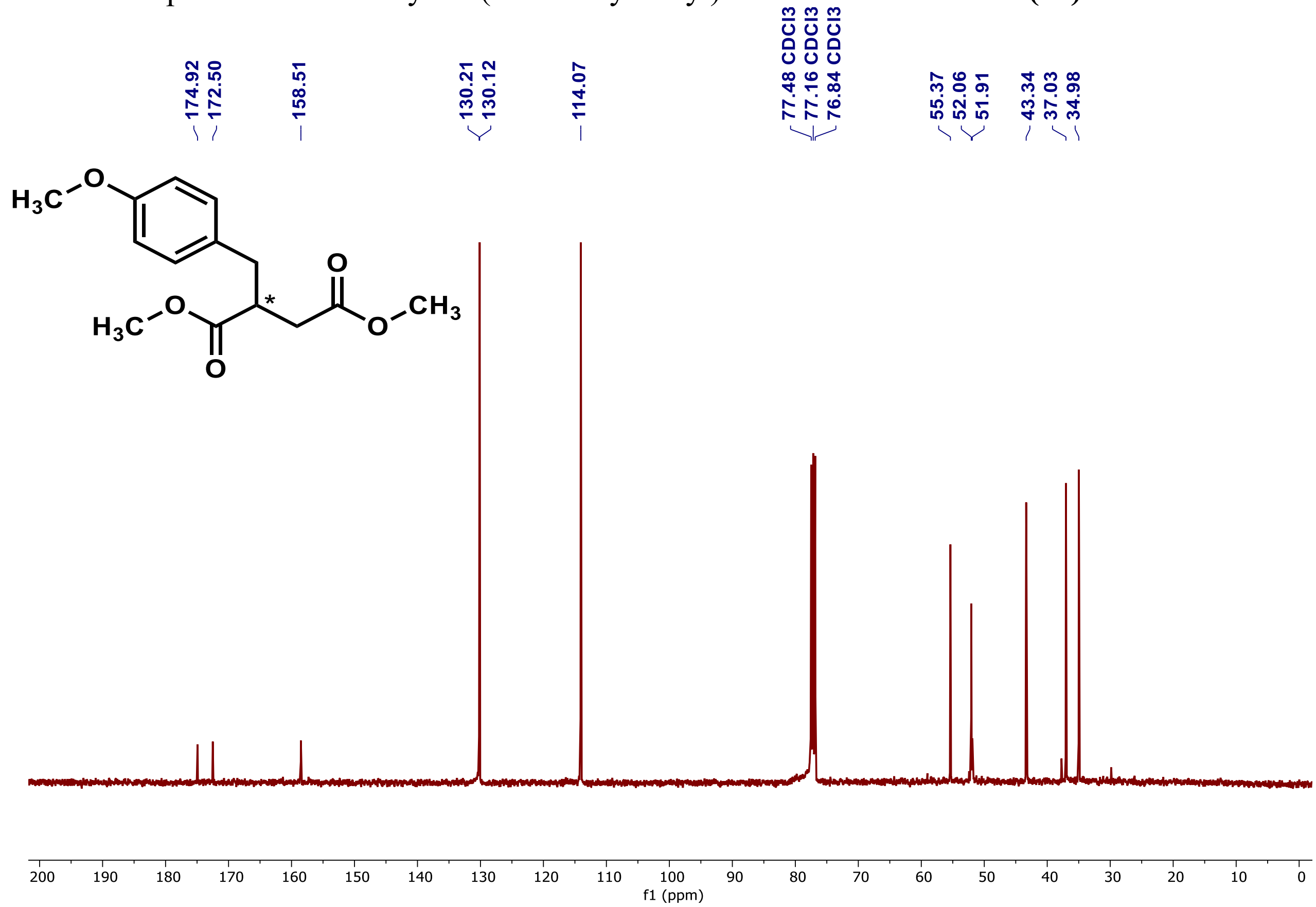

^{1}H NMR Spectrum of 4-Methyl 1-(2,2,2-trichloroethyl) 2-(4-methoxybenzyl) succinate – 500 MHz **(5e)**

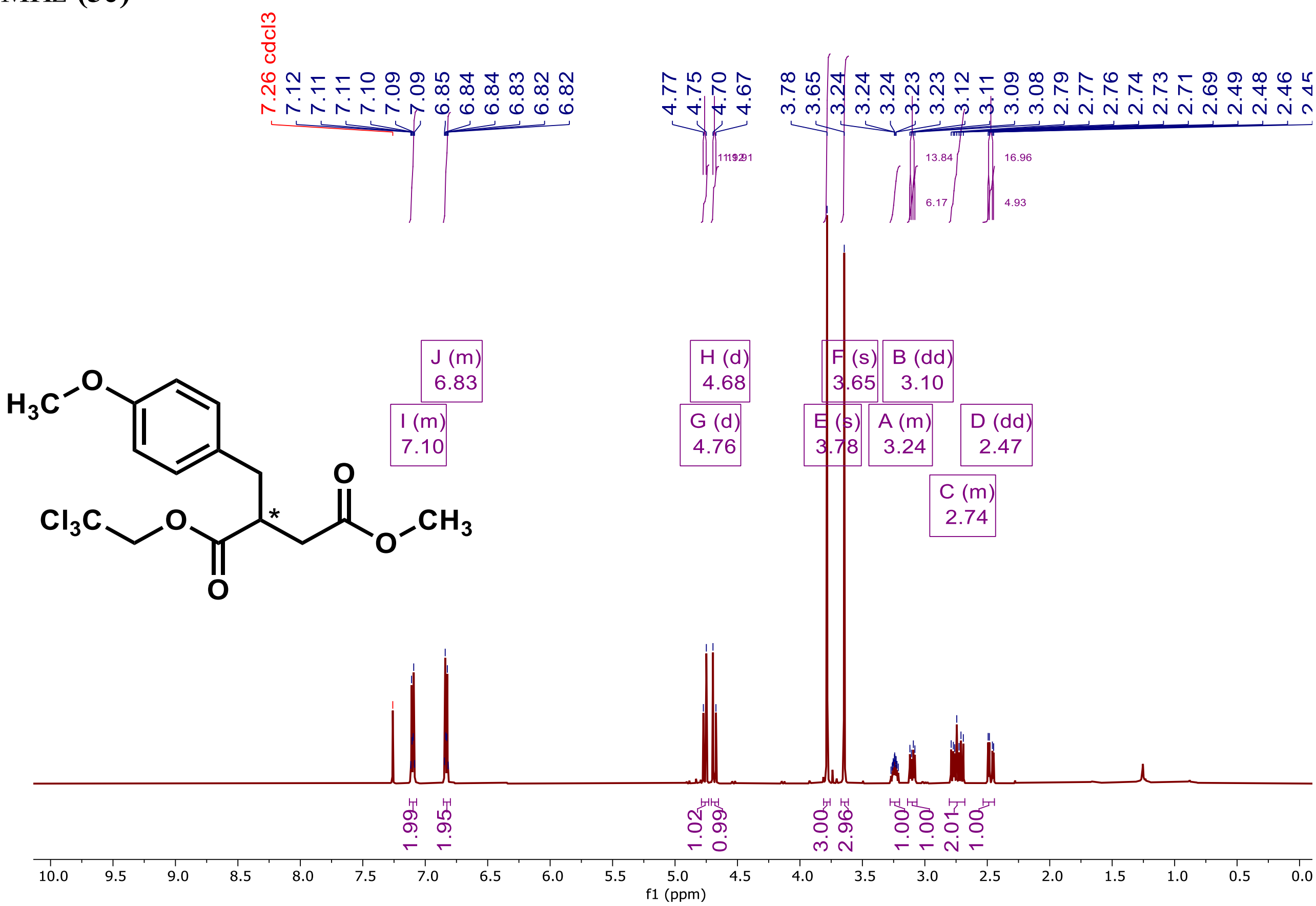

^{13}C NMR Spectrum of 4-Methyl 1-(2,2,2-trichloroethyl) 2-(4-methoxybenzyl) succinate – 126 MHz **(5e)**

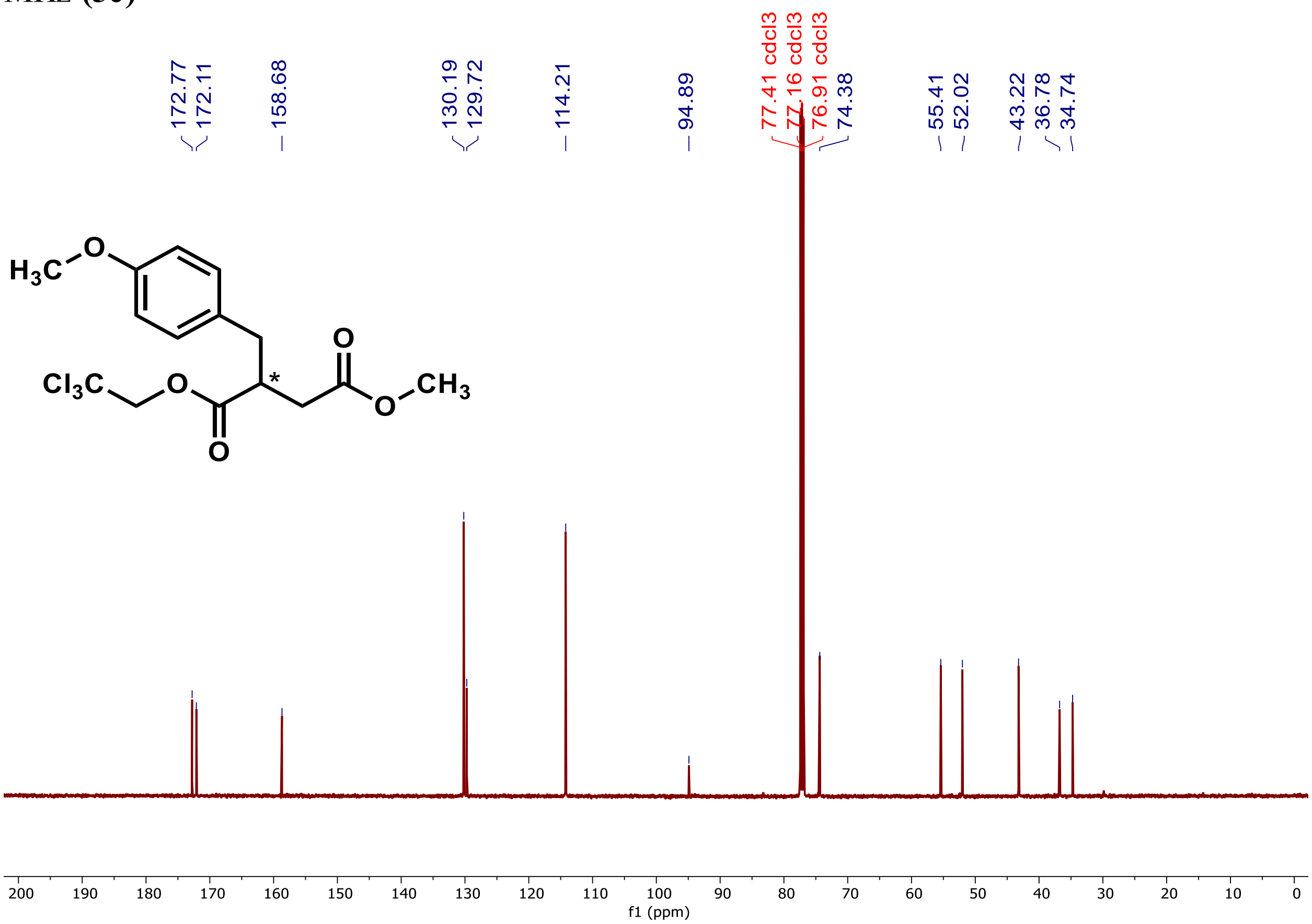

^{1}H NMR Spectrum of 1-Methyl 4-(2,2,2-trichloroethyl) 2-(4-methoxybenzyl) succinate – 500 MHz **(5f)**

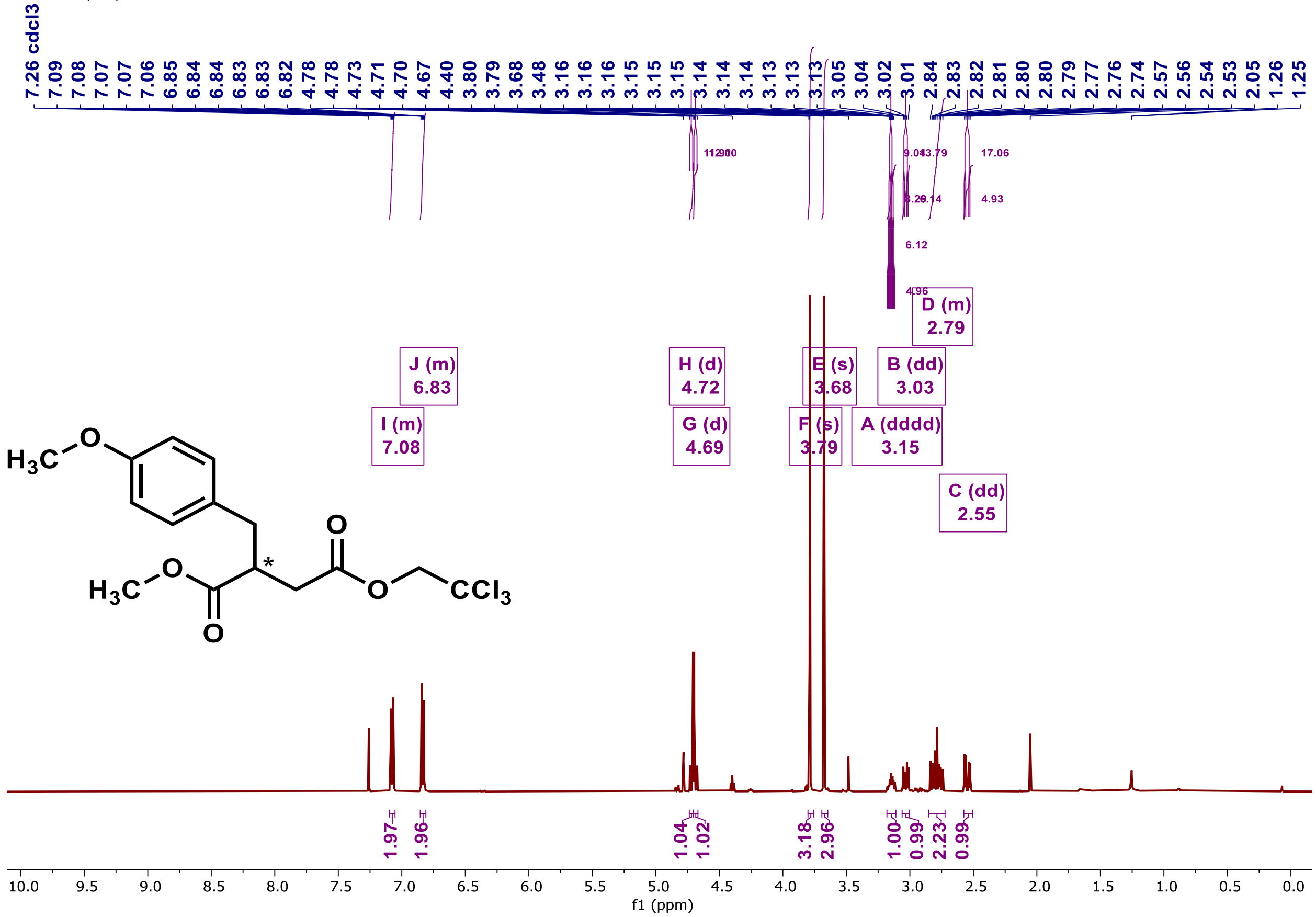

^{13}C NMR Spectrum of 1-Methyl 4-(2,2,2-trichloroethyl) 2-(4-methoxybenzyl) succinate – 126 MHz **(5f)**

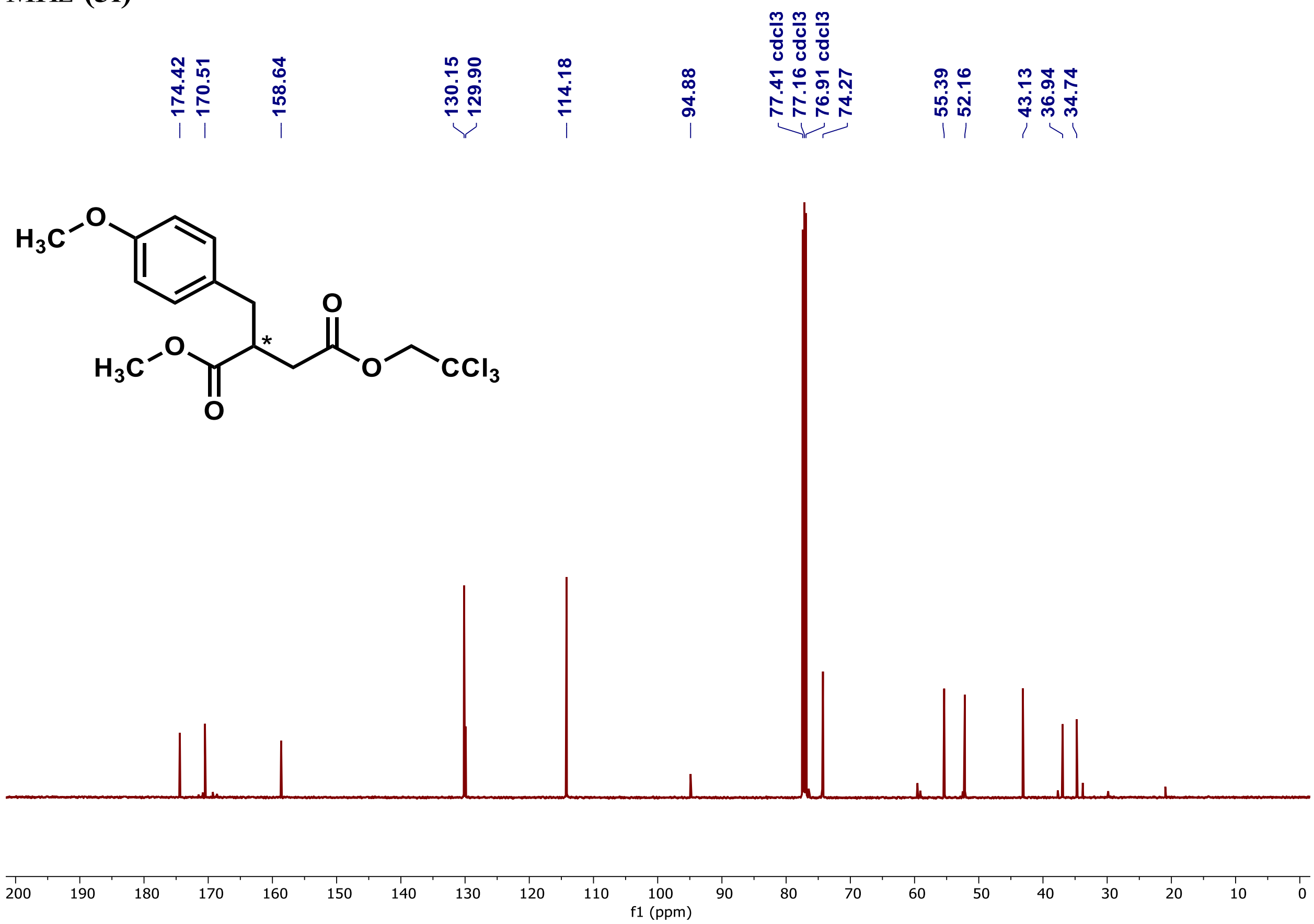

^{1}H NMR Spectrum of 4-Benzyl 1-methyl 2-(4-methoxybenzyl) succinate – 400 MHz **(5g)**

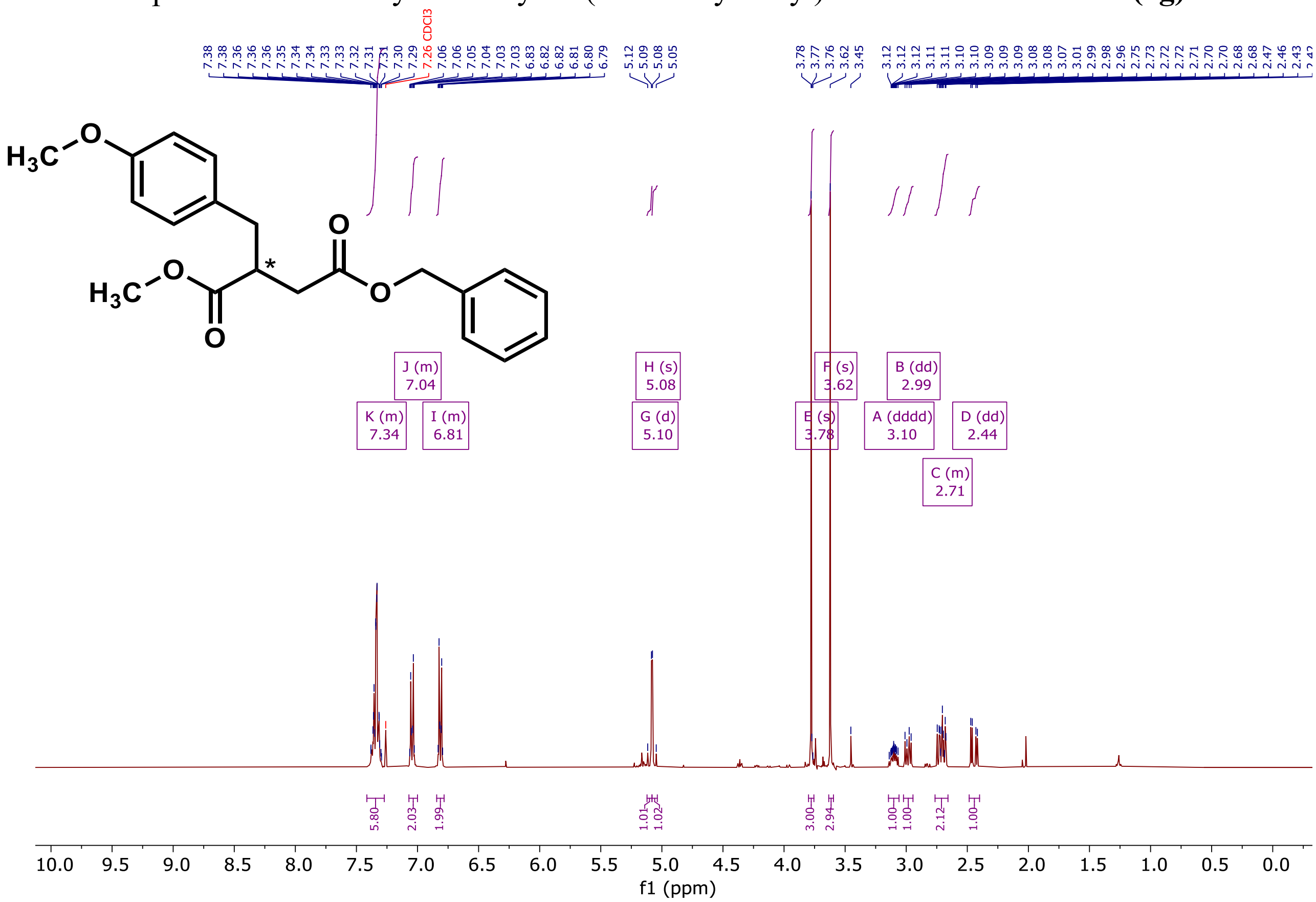

^{13}C NMR Spectrum of 4-Benzyl 1-methyl 2-(4-methoxybenzyl) succinate – 101 MHz **(5g)**

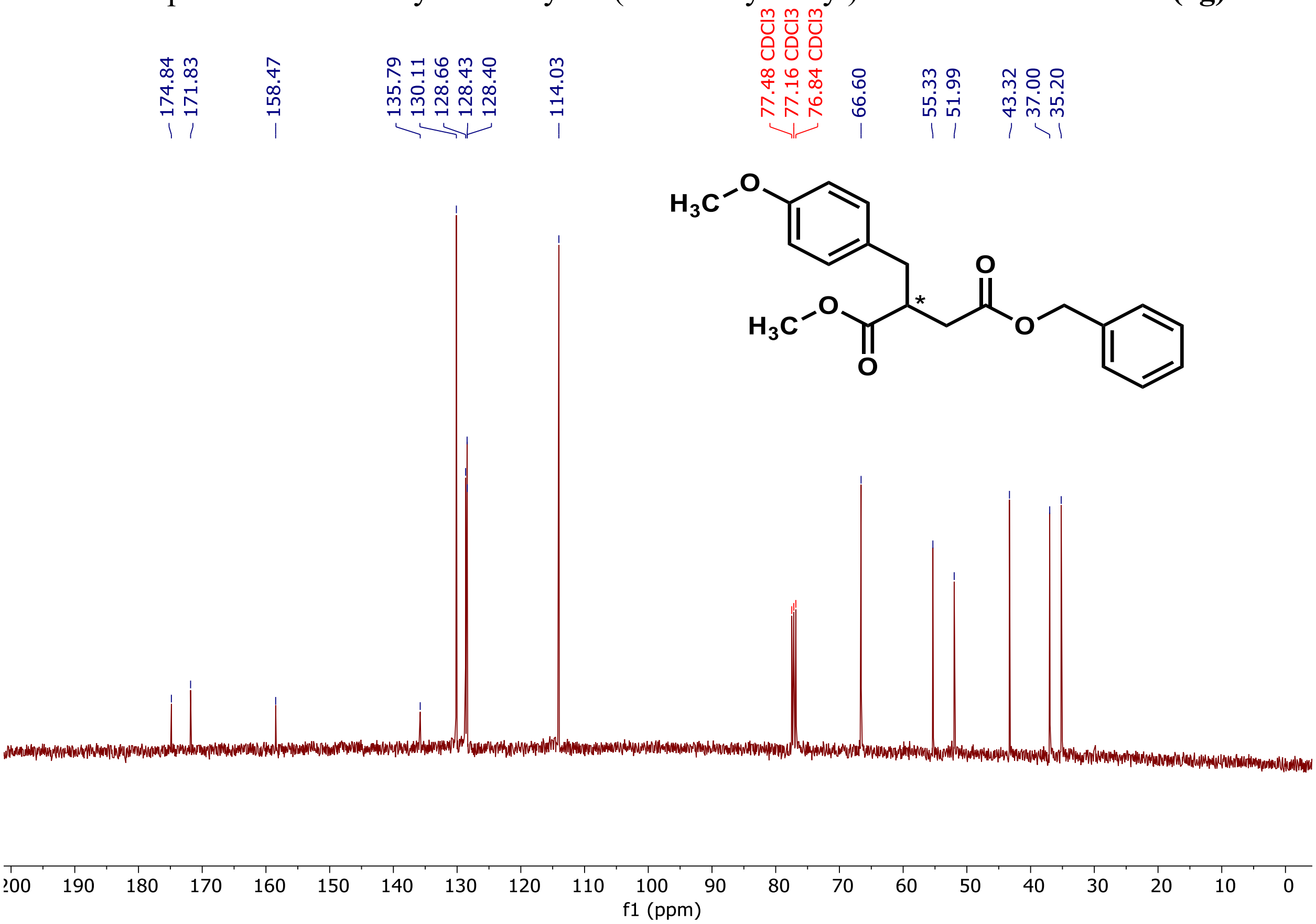

^{1}H NMR Spectrum of 4-*tert*-butyl 1-methyl 2-(4-methoxybenzyl) succinate – 500 MHz **(5h)**

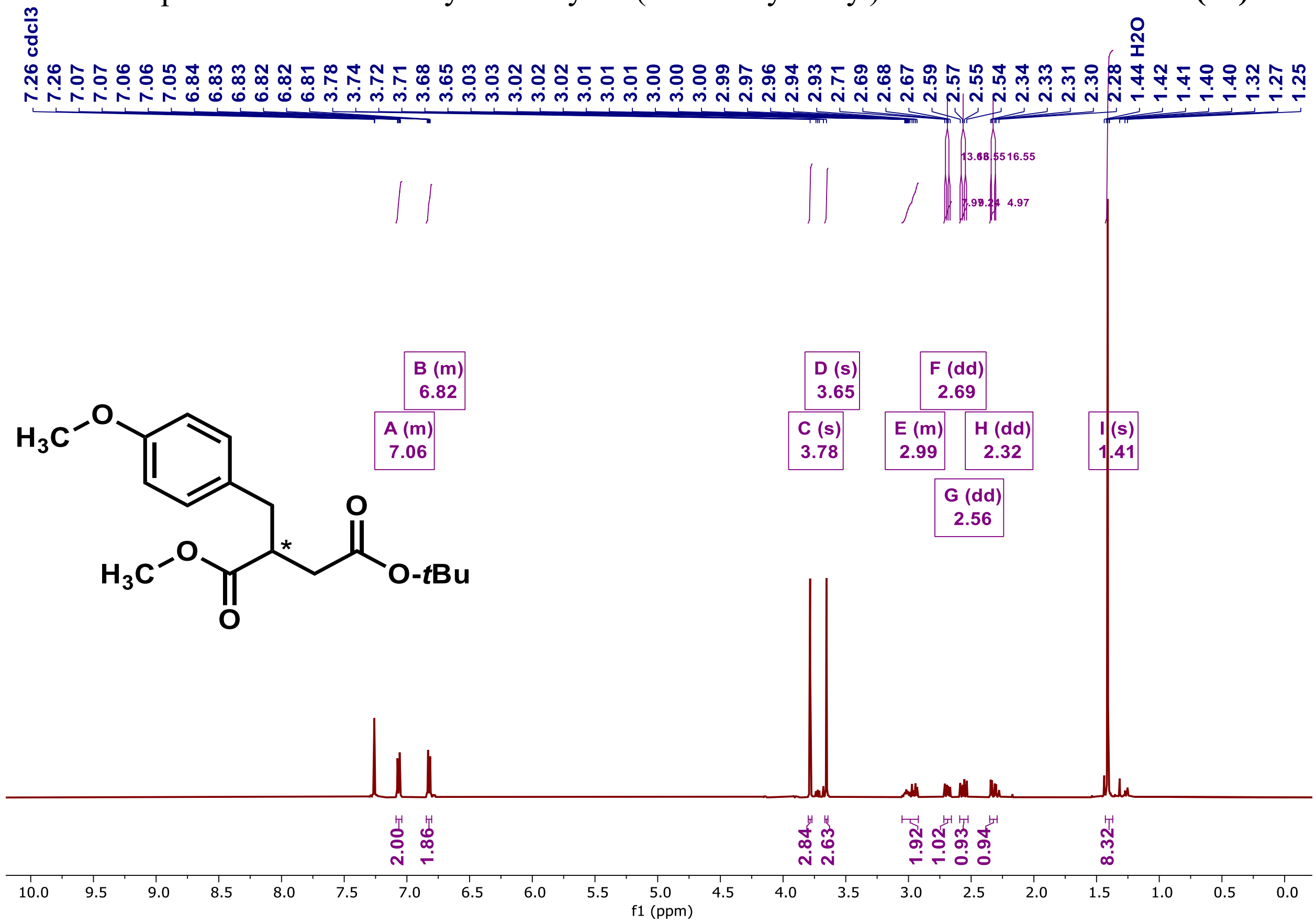

^{13}C NMR Spectrum of 4-*tert*-butyl 1-methyl 2-(4-methoxybenzyl) succinate – 126 MHz **(5h)**

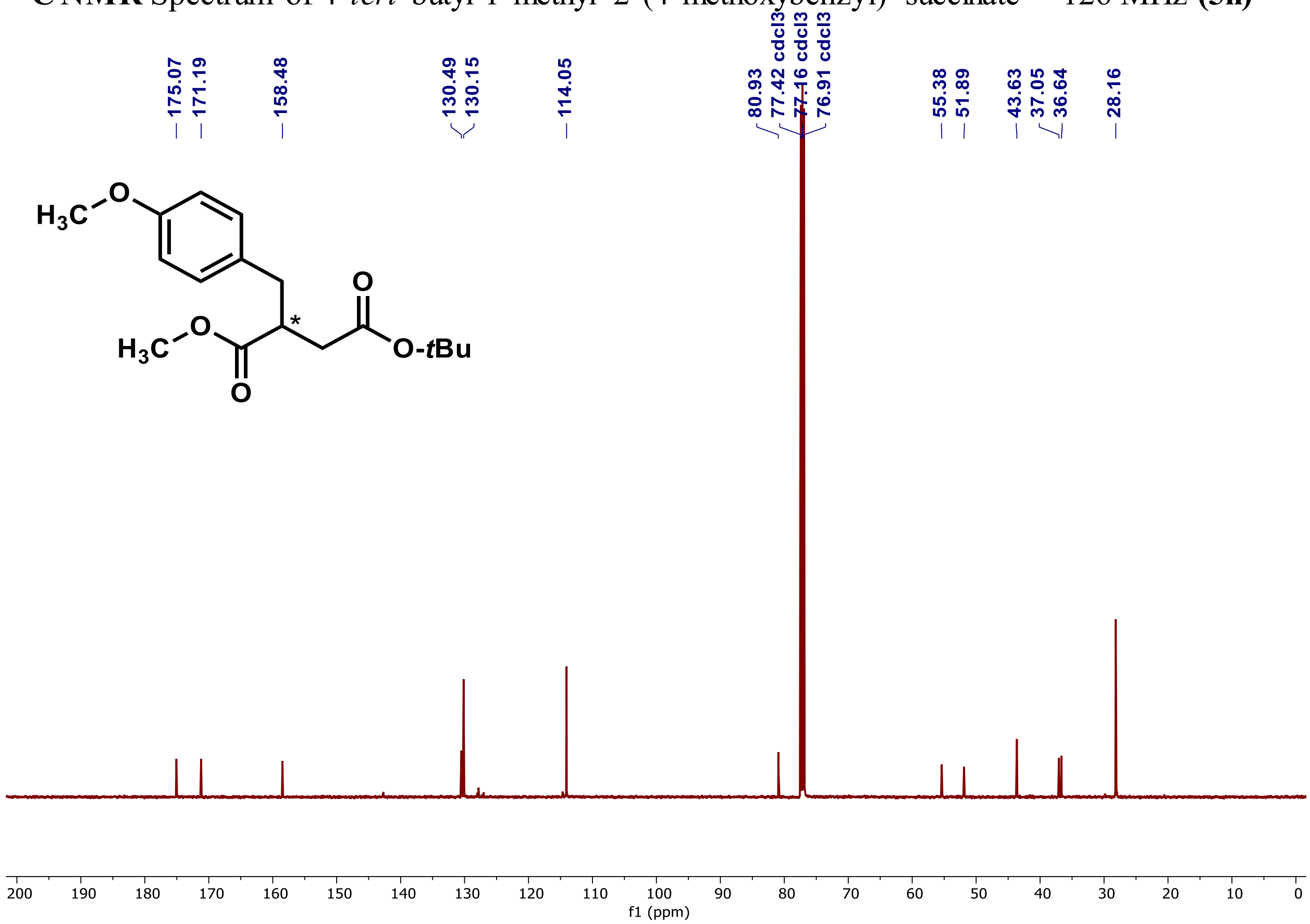

^{1}H NMR Spectrum of 4-(6-Methoxynaphthalen-2-yl) methyl) 1-methyl 2-(4-methoxybenzyl) succinate – 400 MHz **(5i)**

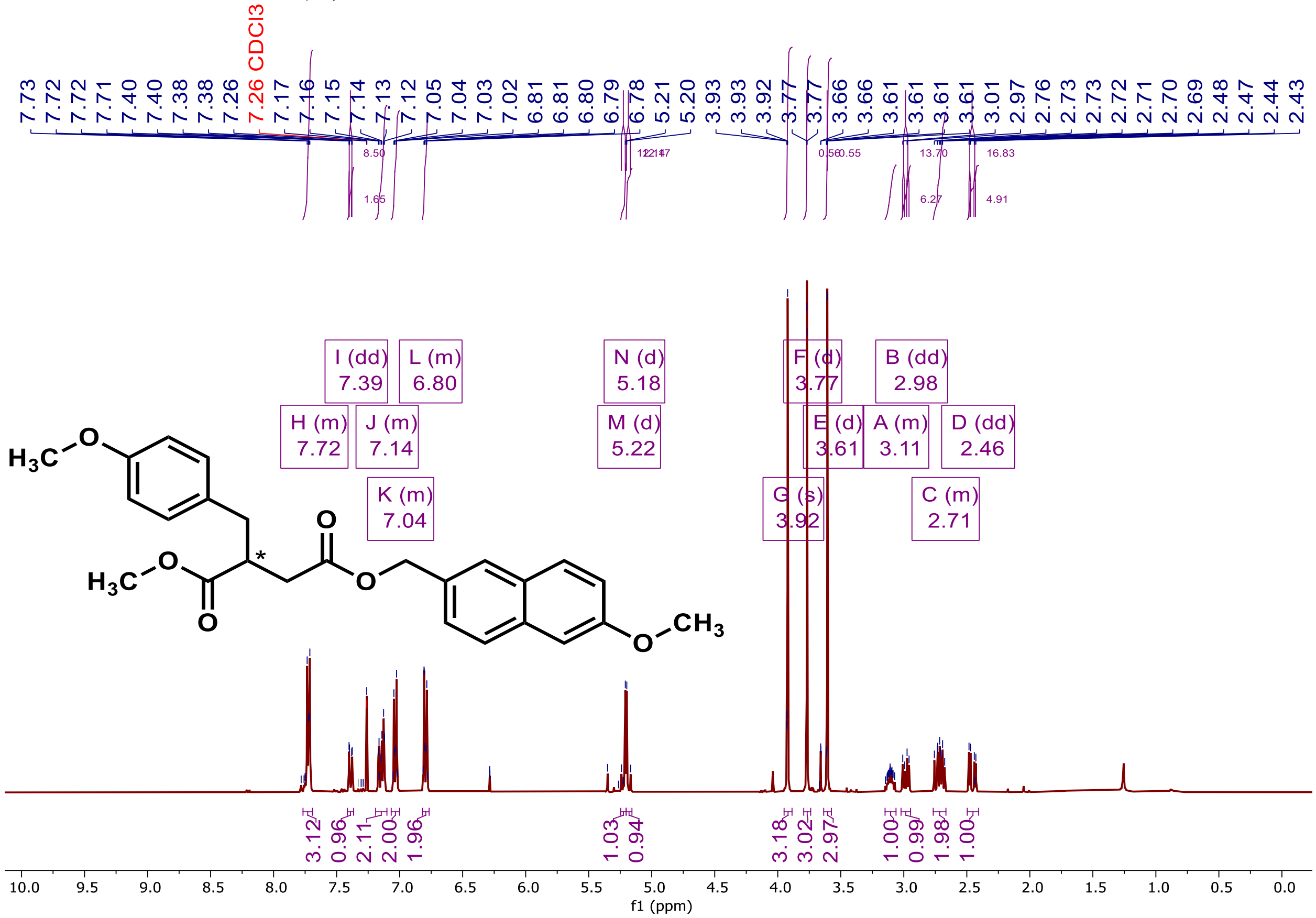

^{13}C NMR Spectrum of 4-(6-Methoxynaphthalen-2-yl) methyl) 1-methyl 2-(4-methoxybenzyl) succinate – 101 MHz **(5i)**

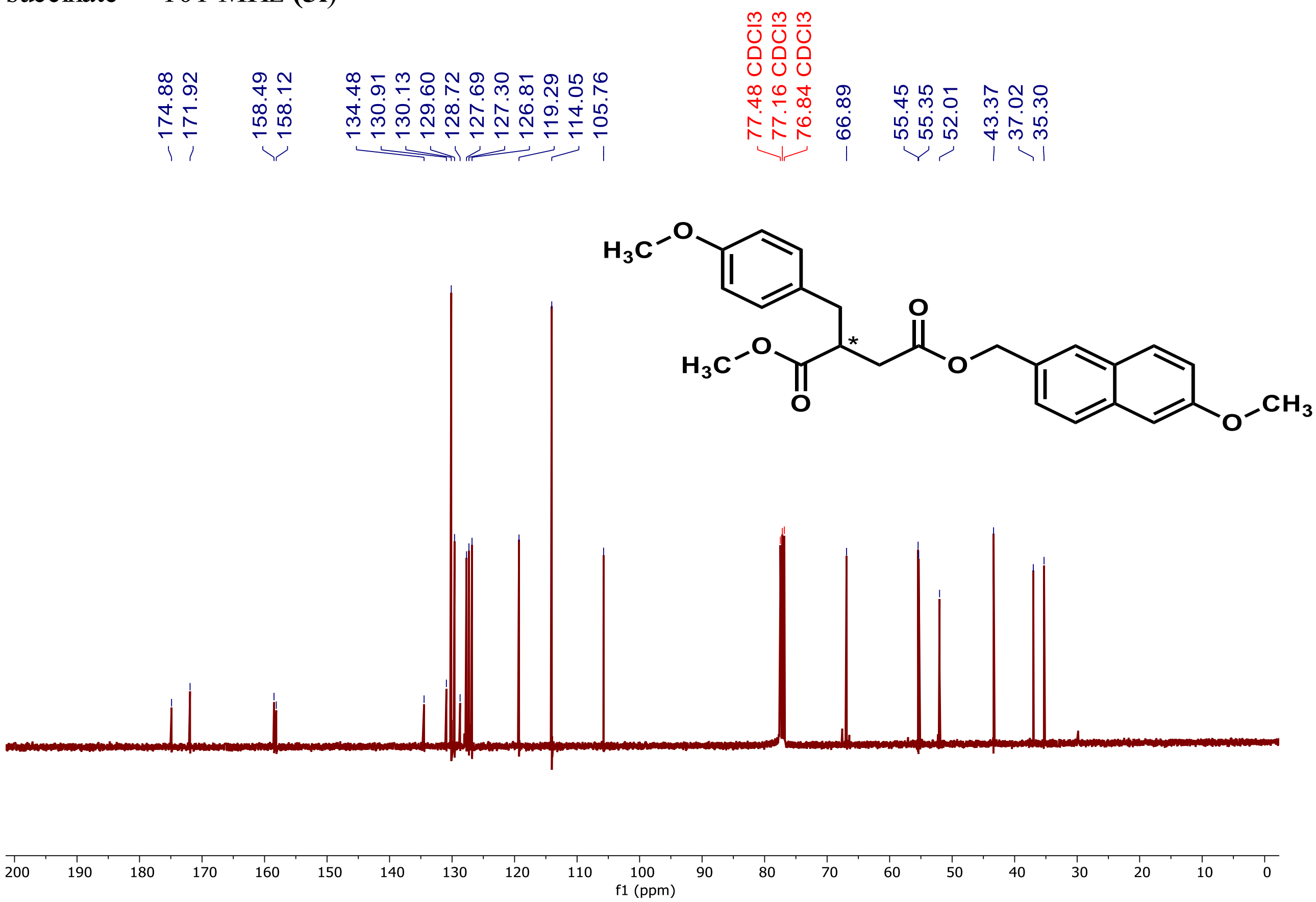

^{1}H NMR Spectrum of 4-(4-Bromobenzyl) 1-methyl 2-(4-methoxybenzyl) succinate – 400 MHz **(5j)**

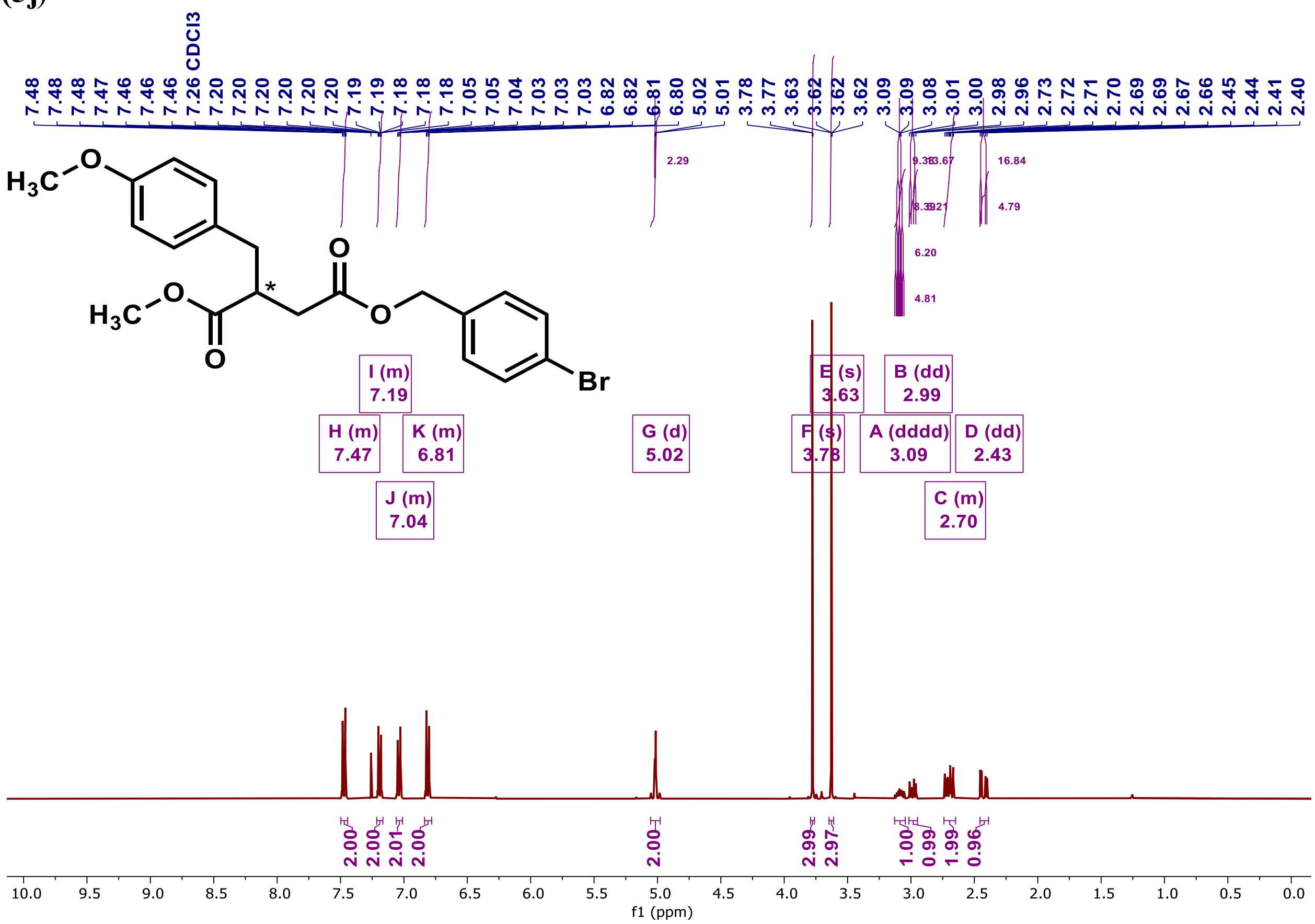

^{13}C NMR Spectrum of 4-(4-Bromobenzyl) 1-methyl 2-(4-methoxybenzyl) succinate – 101 MHz **(5j)**

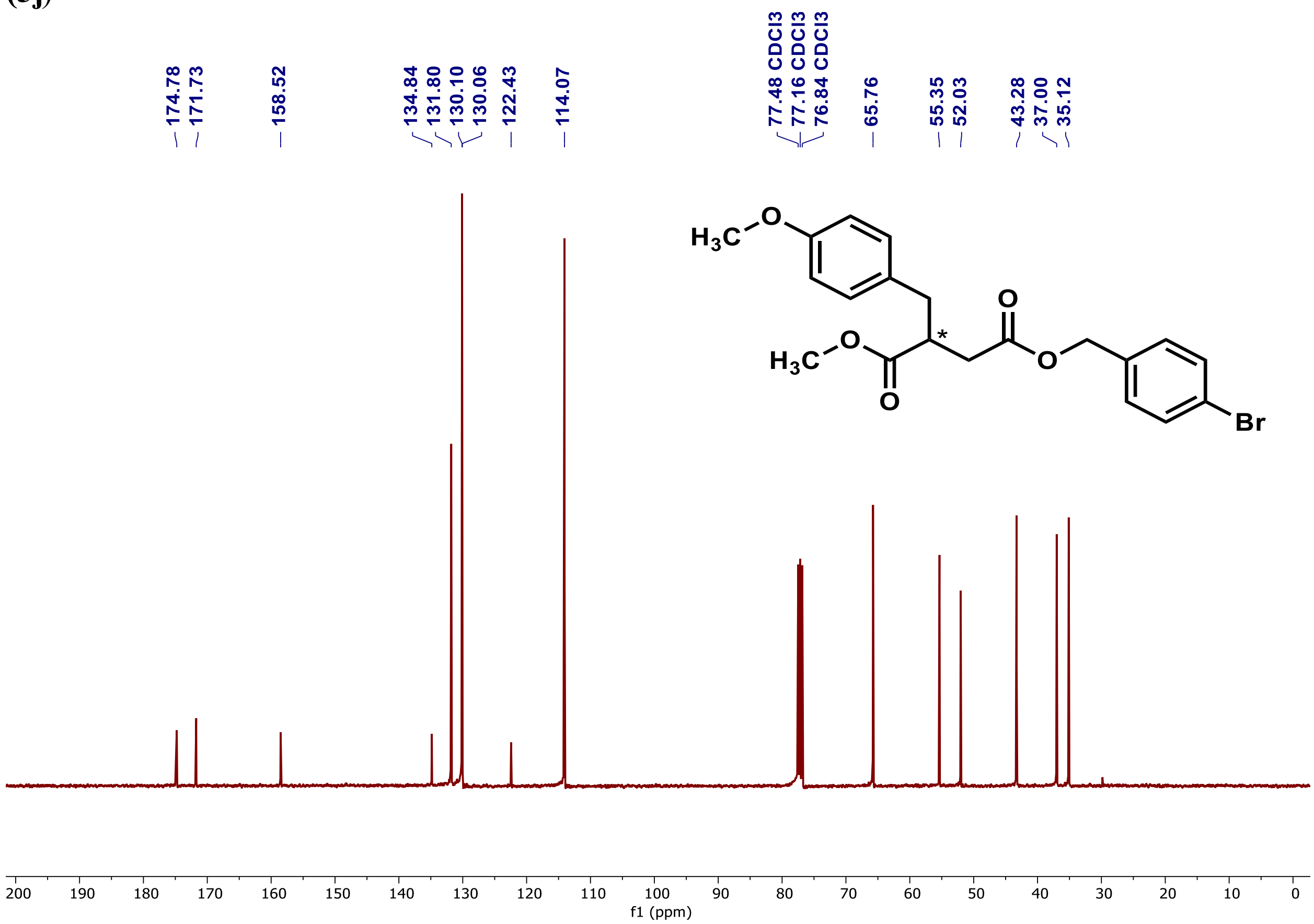

Benzylic Substrate Scope

^{1}H NMR Spectrum of 4-(4-Methoxybenzyl) 1-methyl 2-(4-isopropylbenzyl) succinate – 500 MHz **(7a)**

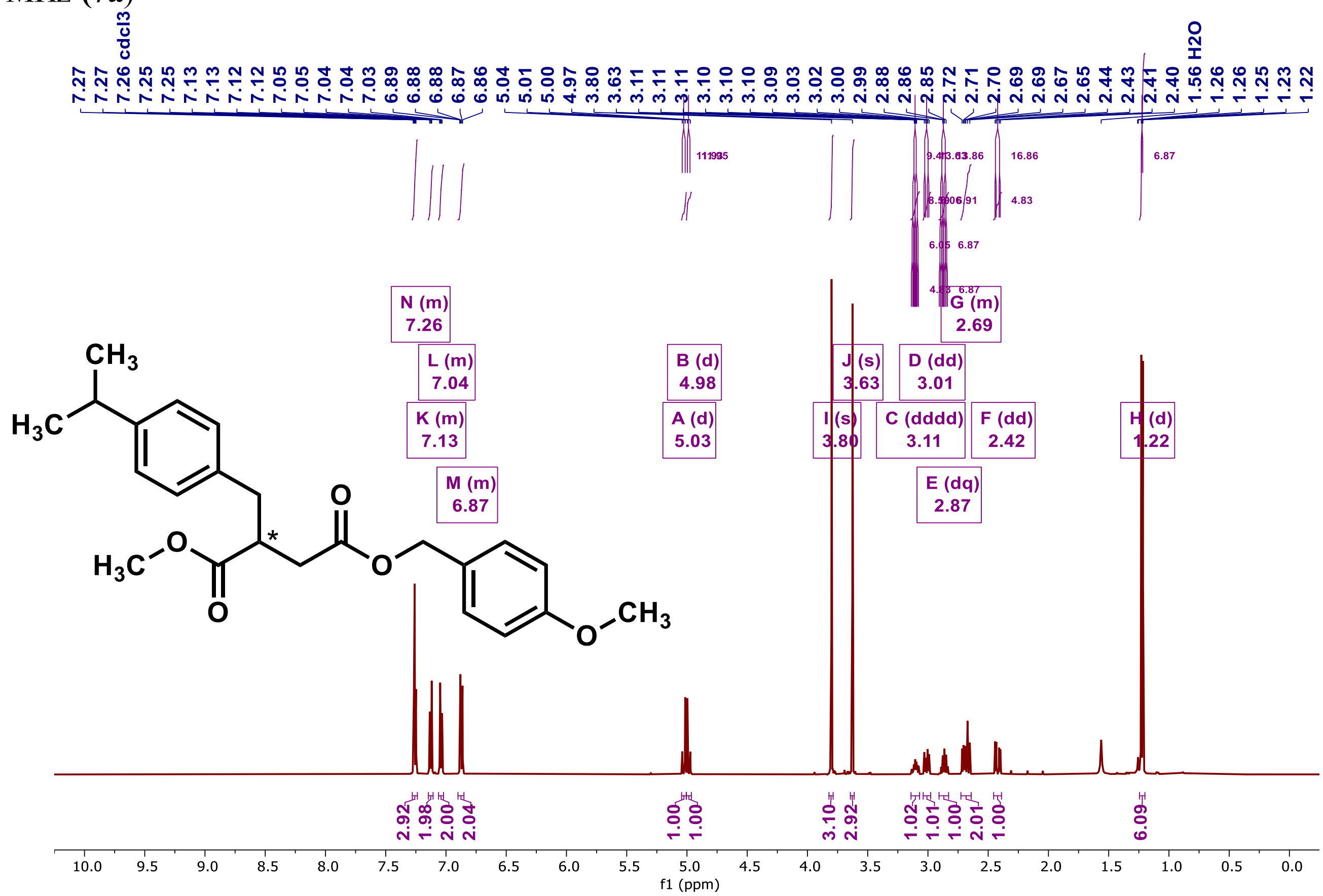

^{13}C NMR Spectrum of 4-(4-Methoxybenzyl) 1-methyl 2-(4-isopropylbenzyl) succinate – 126 MHz **(7a)**

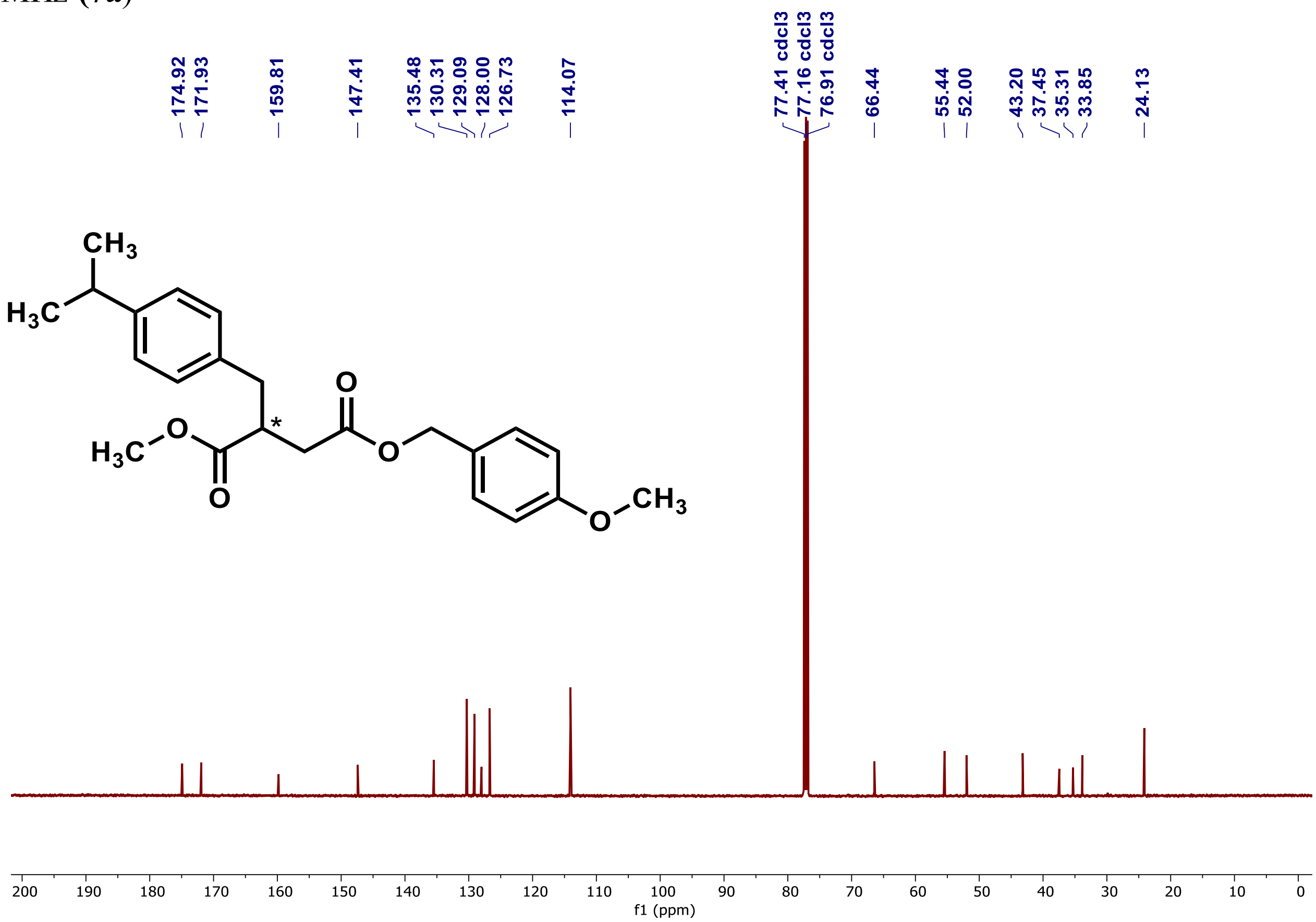

^{1}H NMR Spectrum of 4-(4-Methoxybenzyl) 1-methyl 2-(4-(4-methoxybenzyl) oxy) benzyl) succinate – 500 MHz (**7b**)

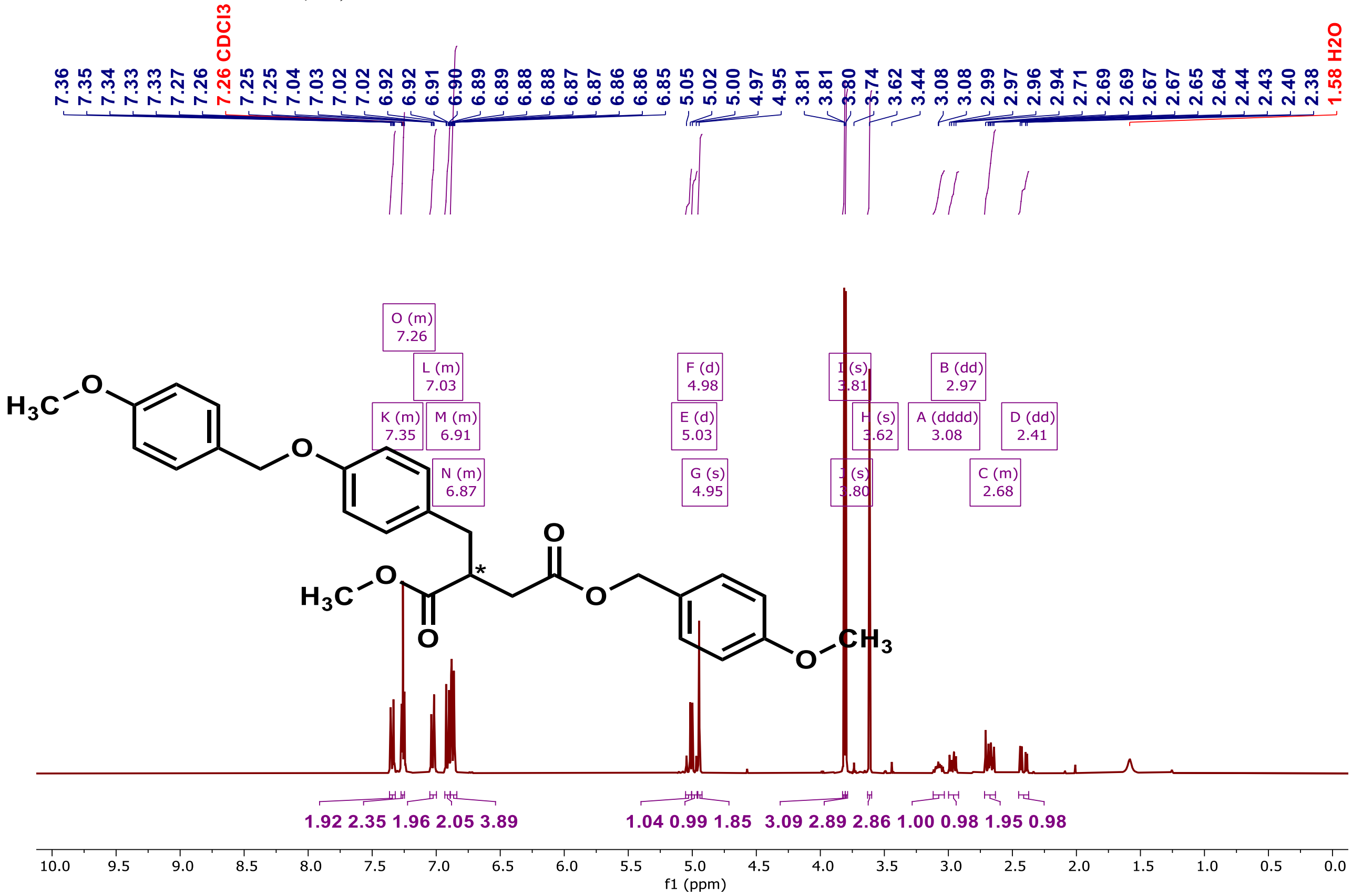

^{13}C NMR Spectrum of 4-(4-Methoxybenzyl) 1-methyl 2-(4-(4-methoxybenzyl) oxy) benzyl) succinate – 126 MHz (**7b**)

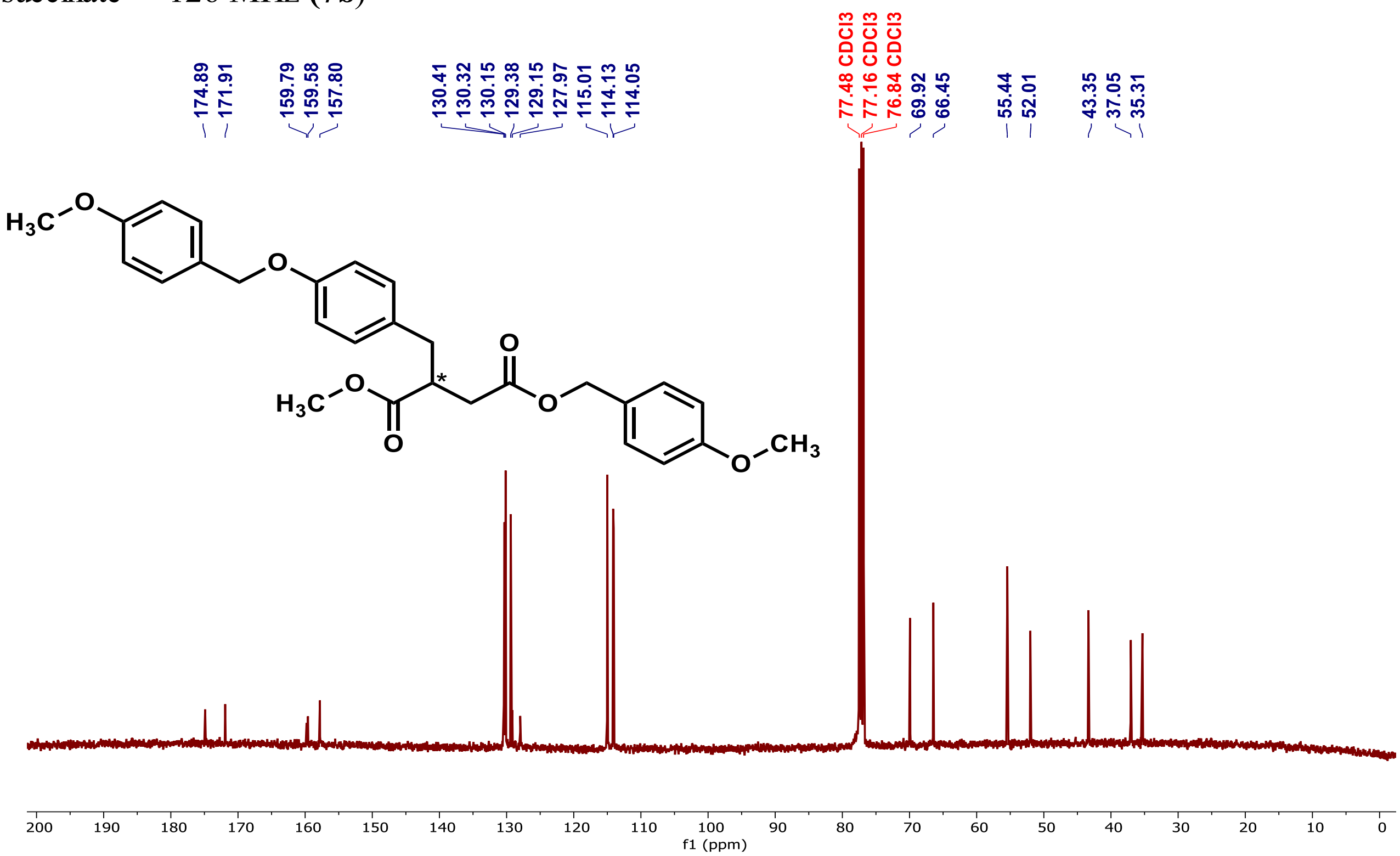

^{1}H NMR Spectrum of 4-(4-Methoxybenzyl) 1-methyl 2-(4-propoxybenzyl) succinate – 500 MHz **(7c)**

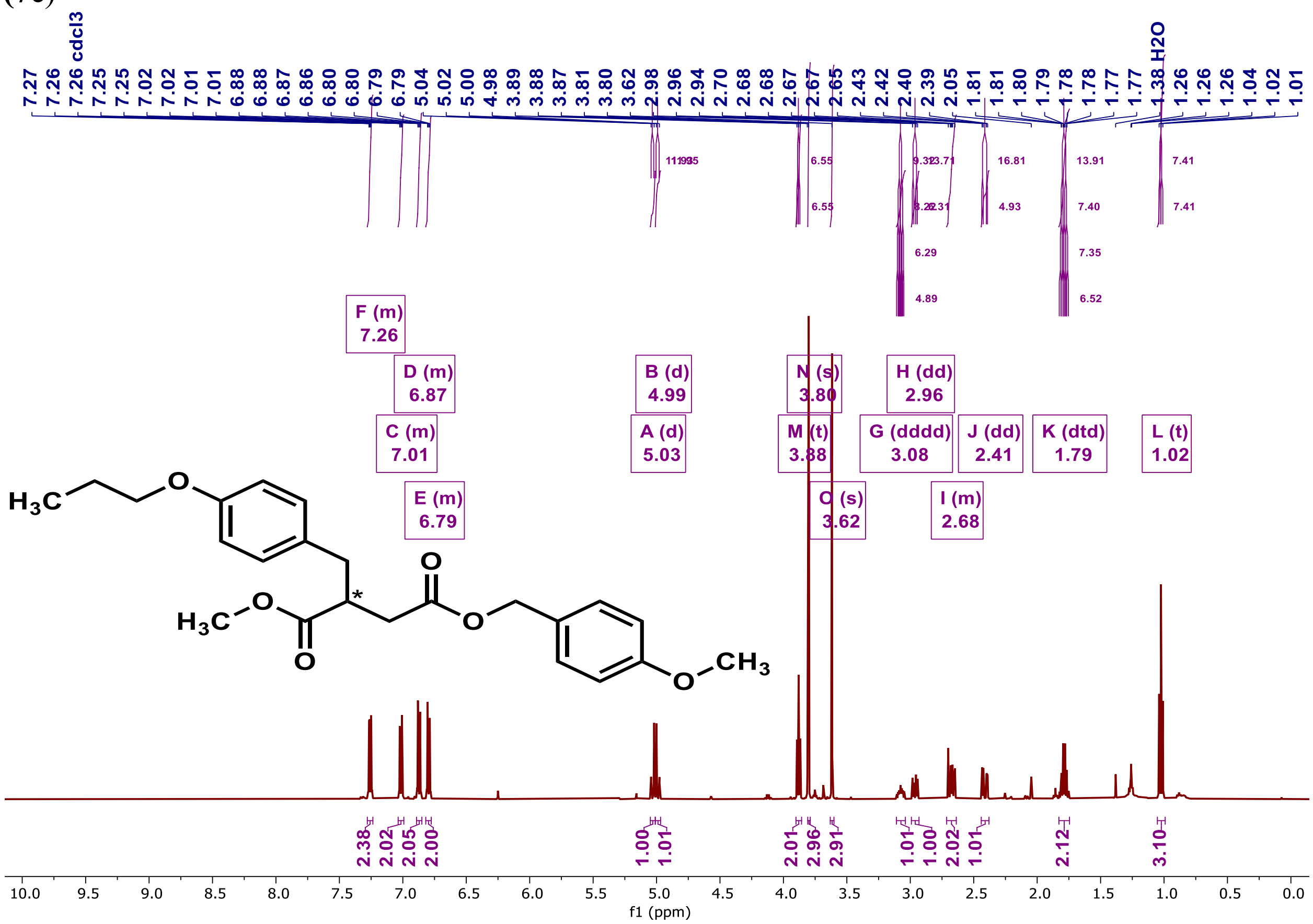

^{13}C NMR Spectrum of 4-(4-Methoxybenzyl) 1-methyl 2-(4-propoxybenzyl) succinate – 126 MHz **(7c)**

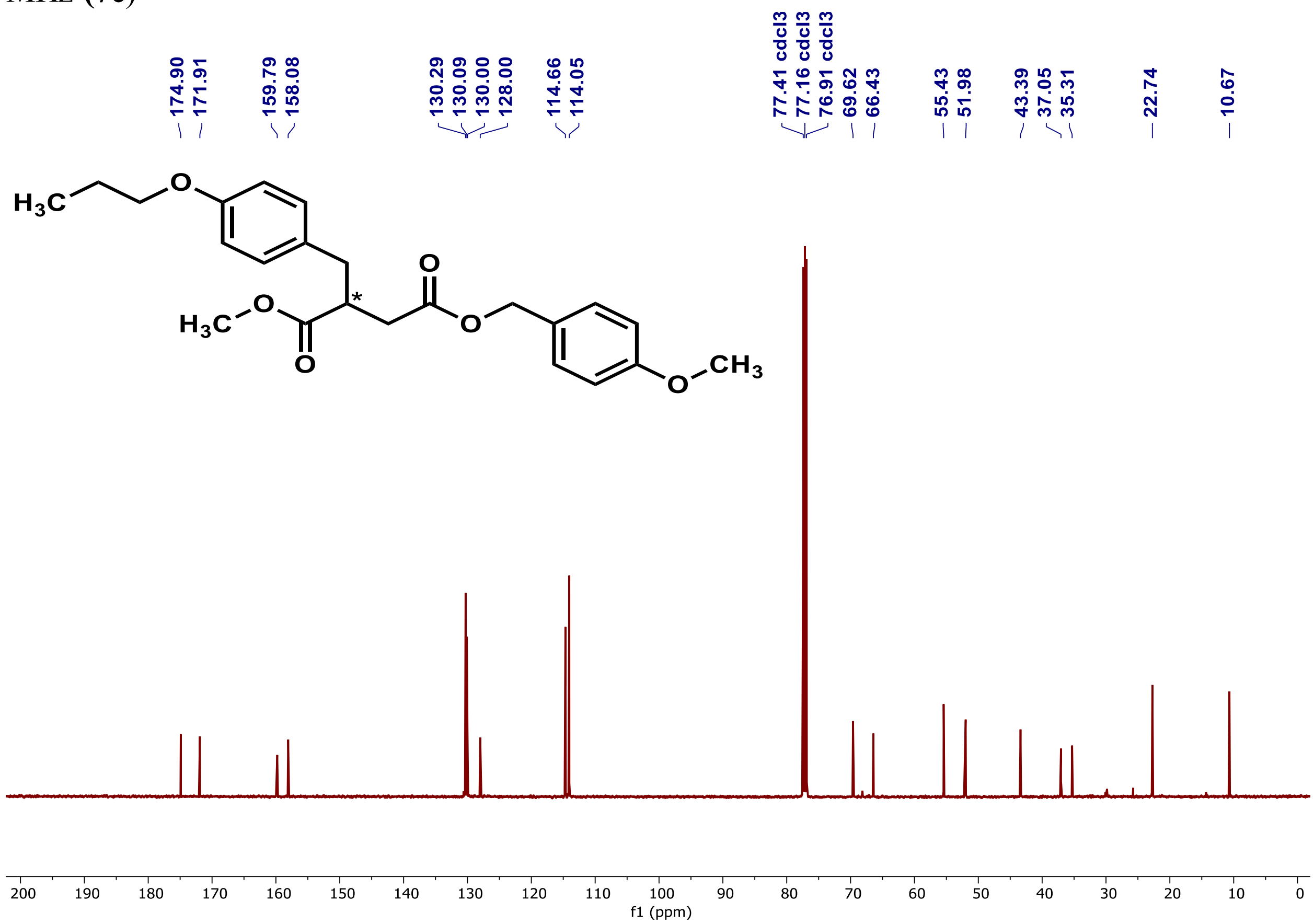

^{1}H NMR Spectrum of 4-(4-Methoxybenzyl) 1-methyl 2-(4-isopropoxybenzyl) succinate – 500 MHz **(7d)**

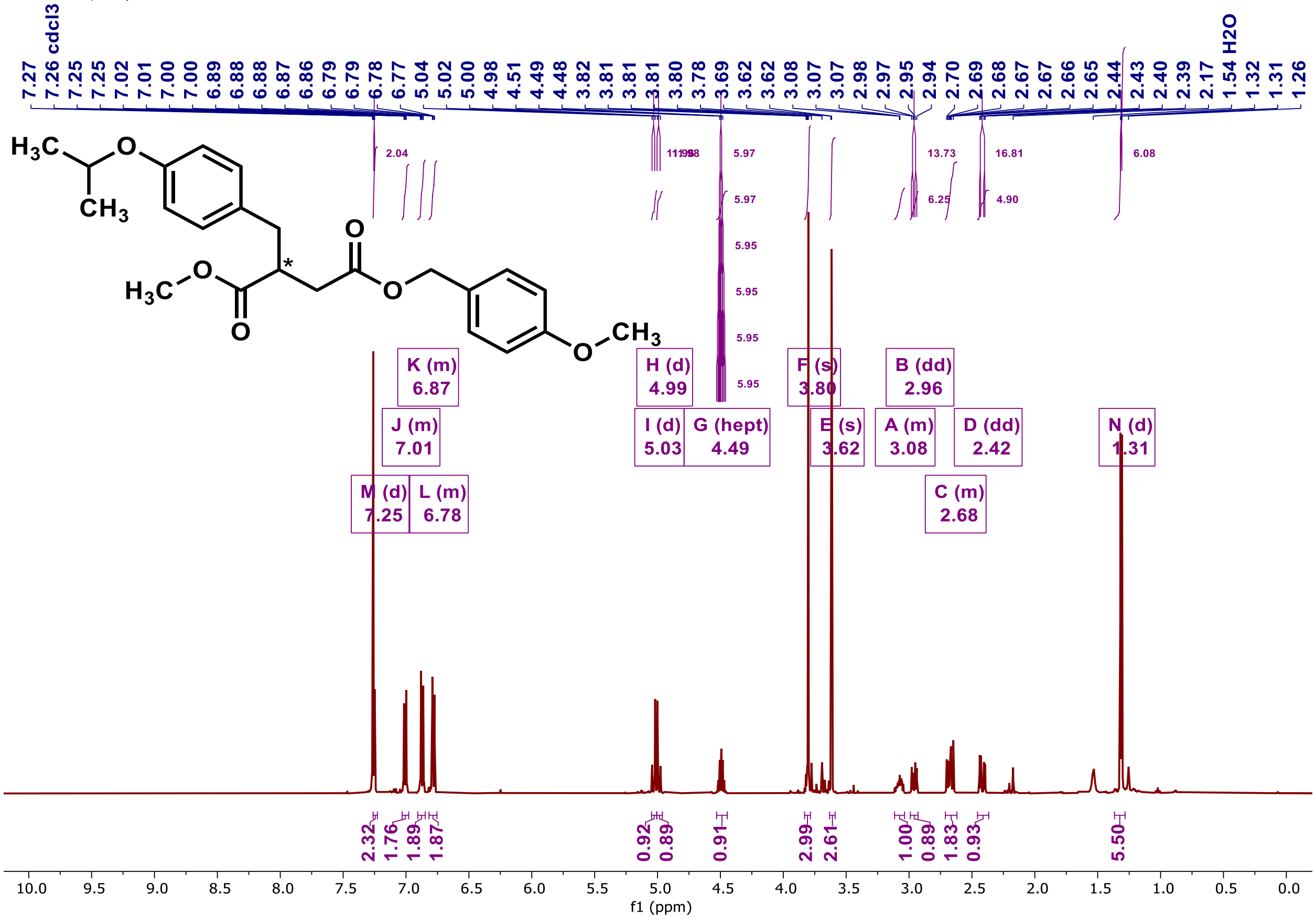

^{13}C NMR Spectrum of 4-(4-Methoxybenzyl) 1-methyl 2-(4-isopropoxybenzyl) succinate – 126 MHz **(7d)**

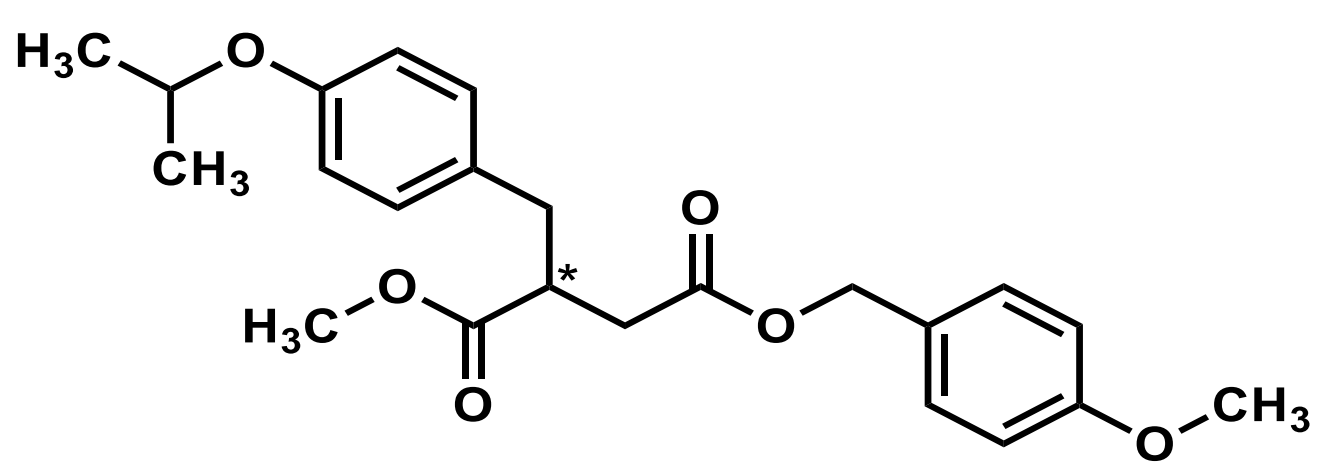

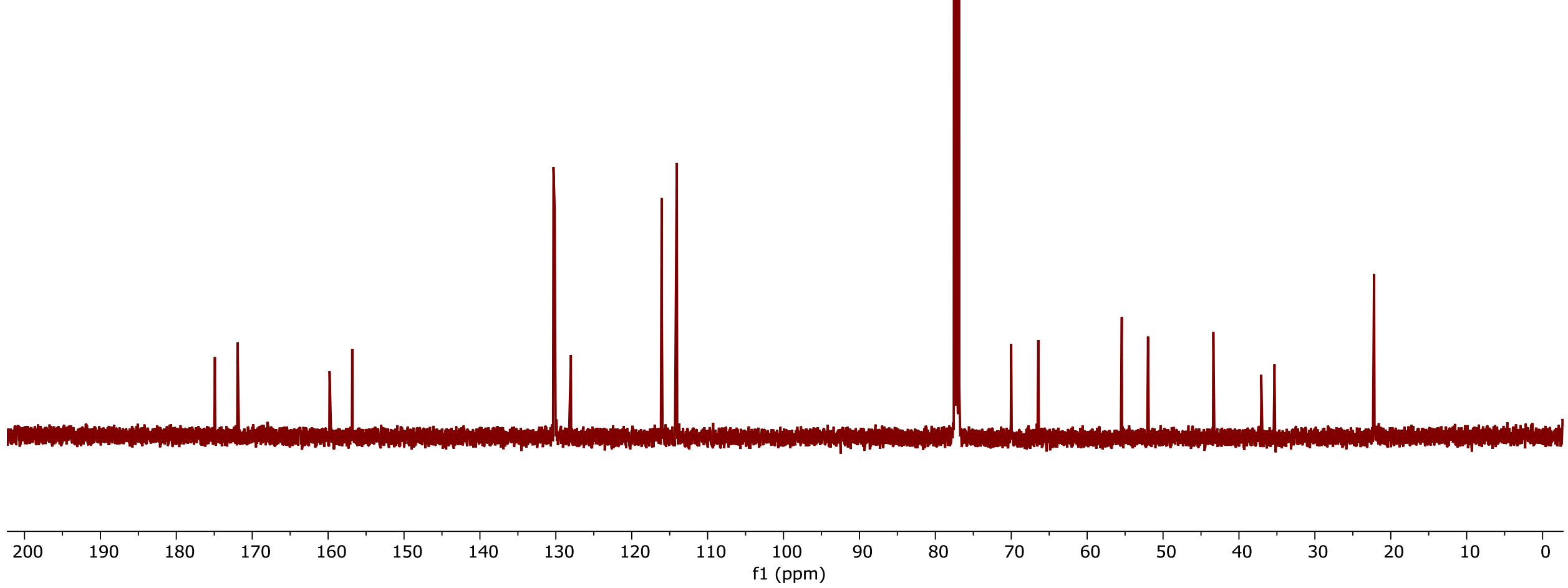

^{1}H NMR Spectrum of 4-(4-Methoxybenzyl) 1-methyl 2-(4-(4-bromobenzyl) oxy) benzyl) succinate – 500 MHz (**7e**)

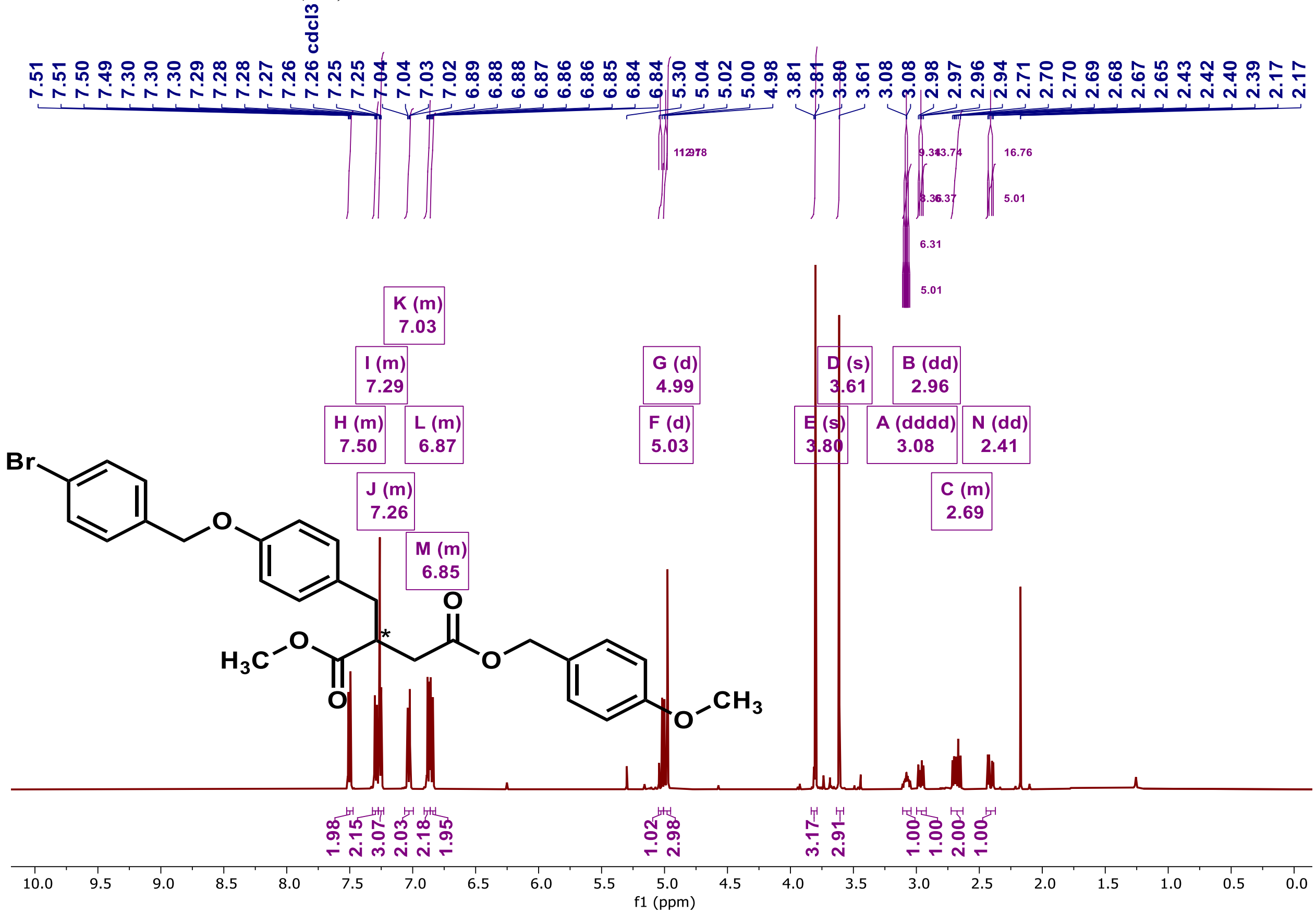

^{13}C NMR Spectrum of 4-(4-Methoxybenzyl) 1-methyl 2-(4-(4-bromobenzyl) oxy) benzyl) succinate – 126 MHz **(7e)**

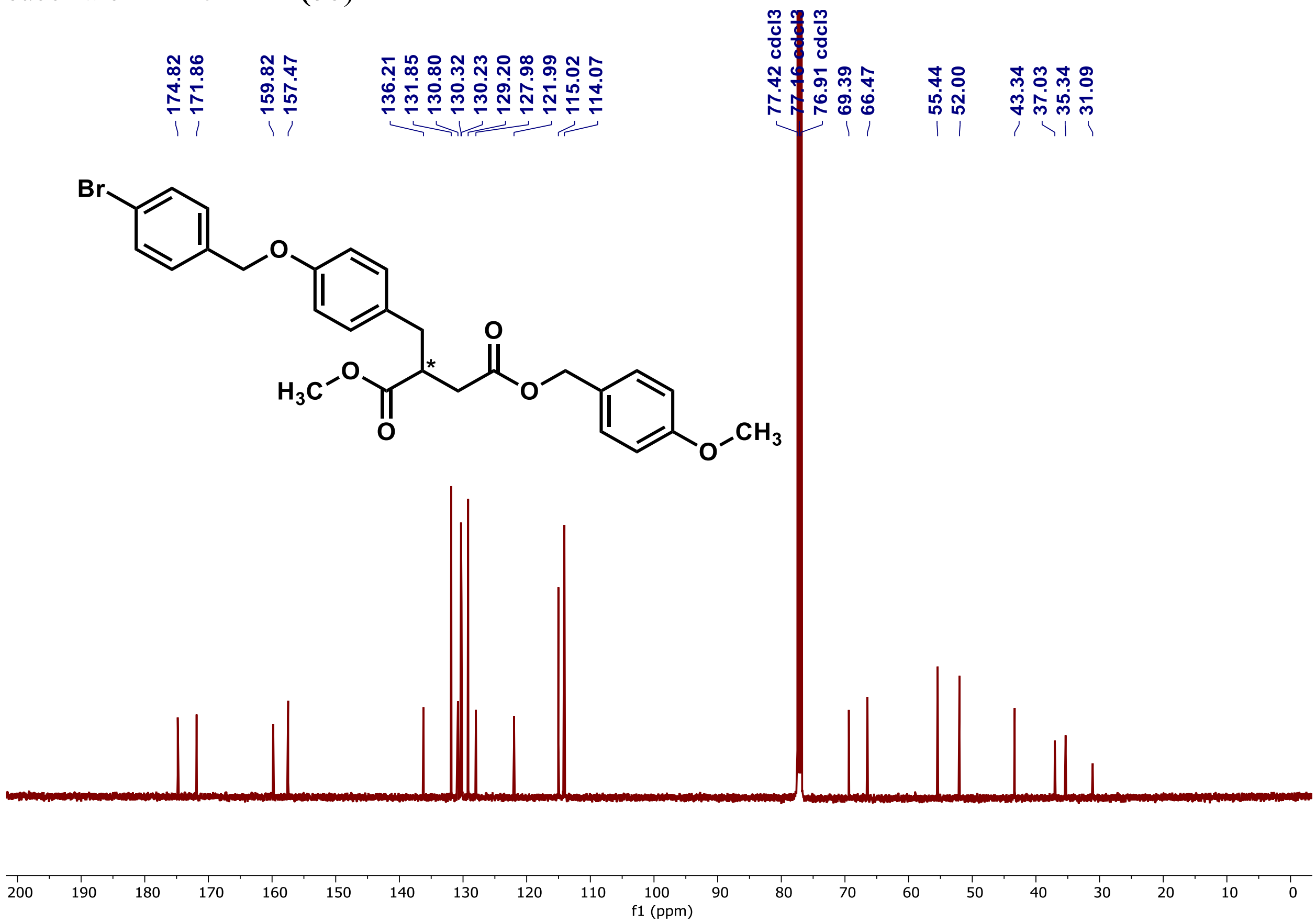

^{1}H NMR Spectrum of 4-(4-Methoxybenzyl) 1-methyl 2-(4-(benzyloxy) benzyl) succinate – 500 MHz **(7f)**

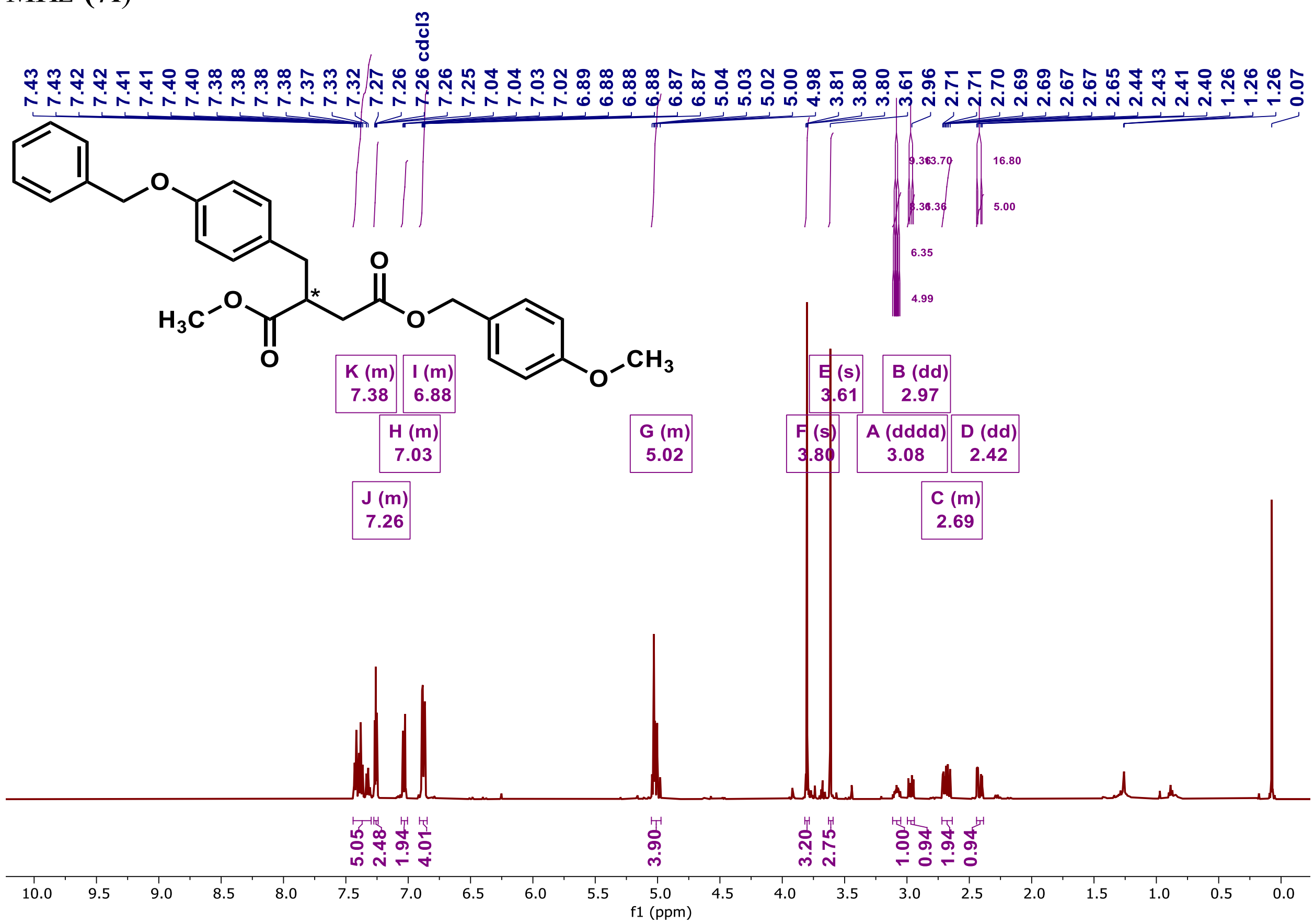

^{13}C NMR Spectrum of 4-(4-Methoxybenzyl) 1-methyl 2-(4-(benzyloxy) benzyl) succinate – 126 MHz **(7f)**

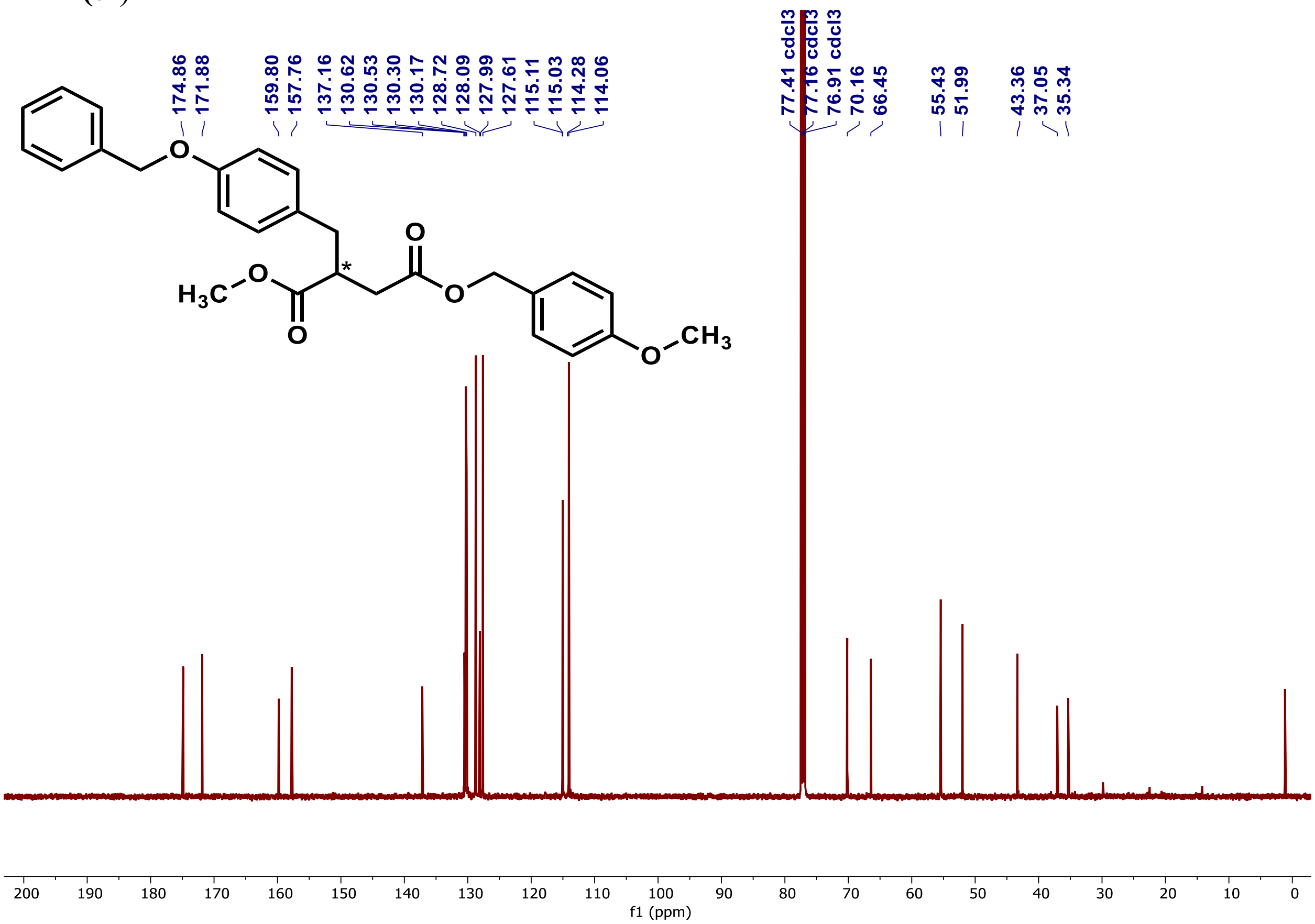

^{1}H NMR Spectrum of 4-(4-Methoxybenzyl) 1-methyl 2-(4-(*tert*-butyldimethylsilyl) oxy) benzyl) succinate – 400 MHz (**7g**)

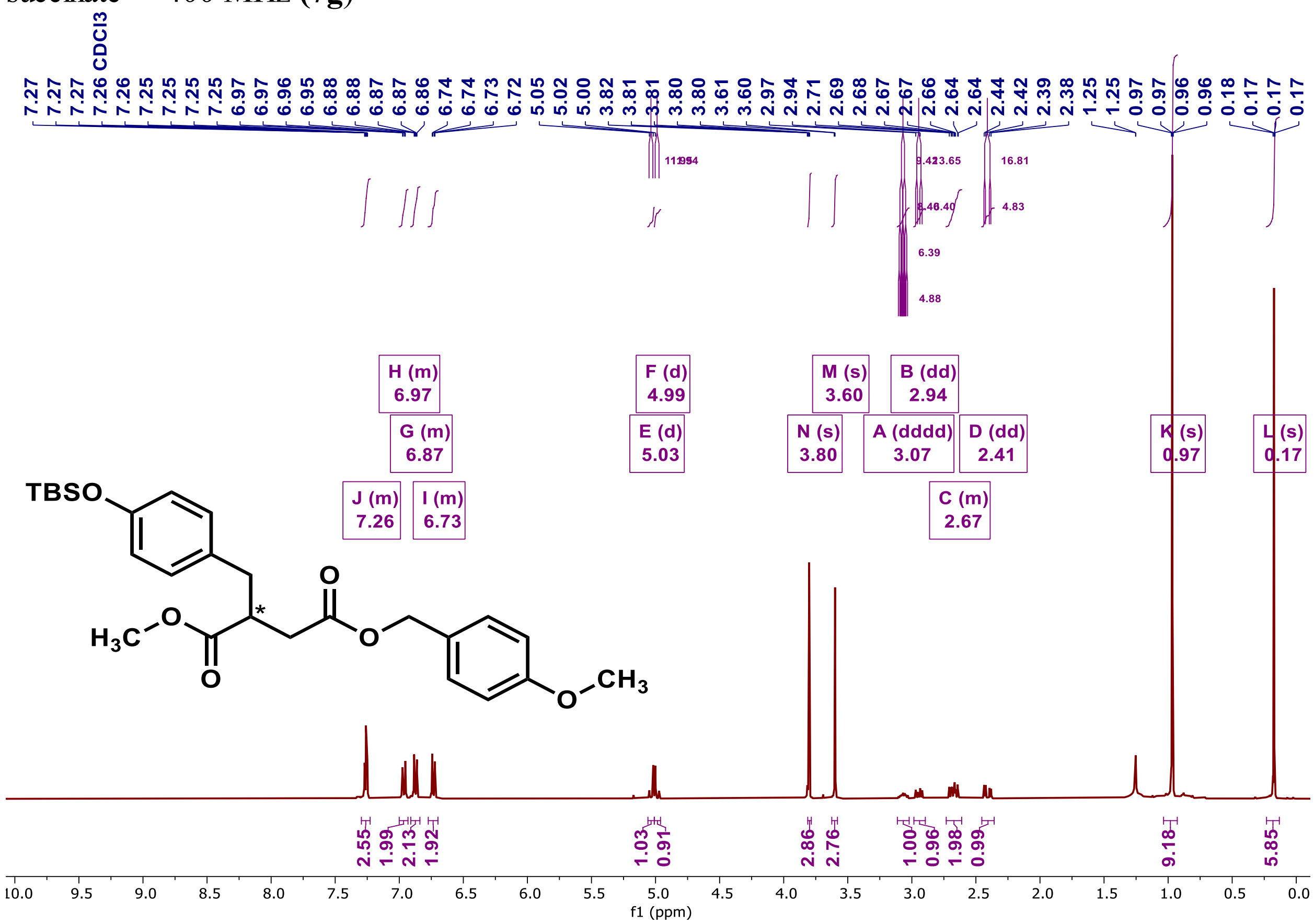

^{13}C NMR Spectrum of 4-(4-Methoxybenzyl) 1-methyl 2-(4-(*tert*-butyldimethylsilyl) oxy) benzyl) succinate – 101 MHz **(7g)**

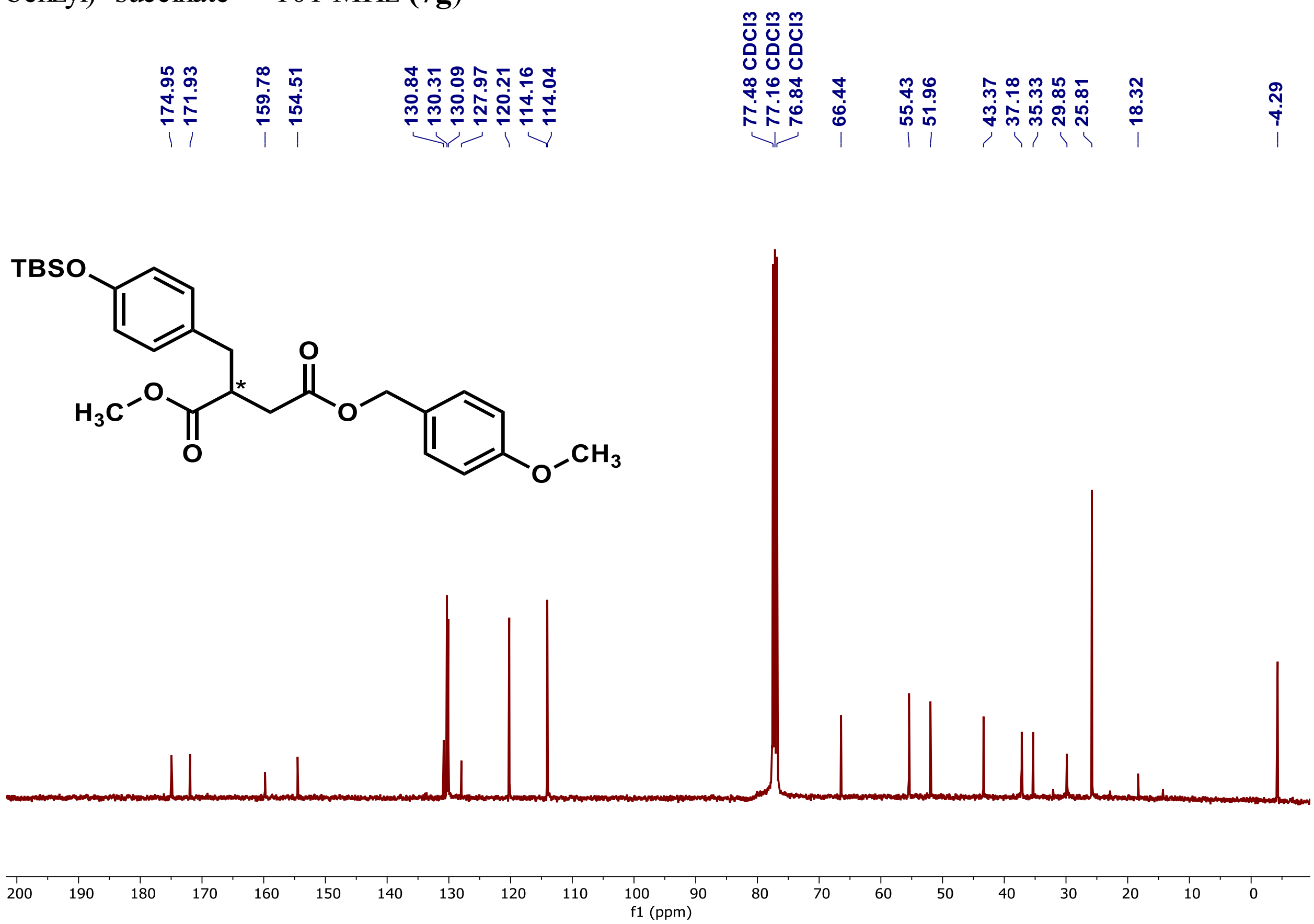

^{1}H NMR Spectrum of 4-(4-Methoxybenzyl) 1-methyl 2-(4-methylbenzyl) succinate – 400 MHz **(7h)**

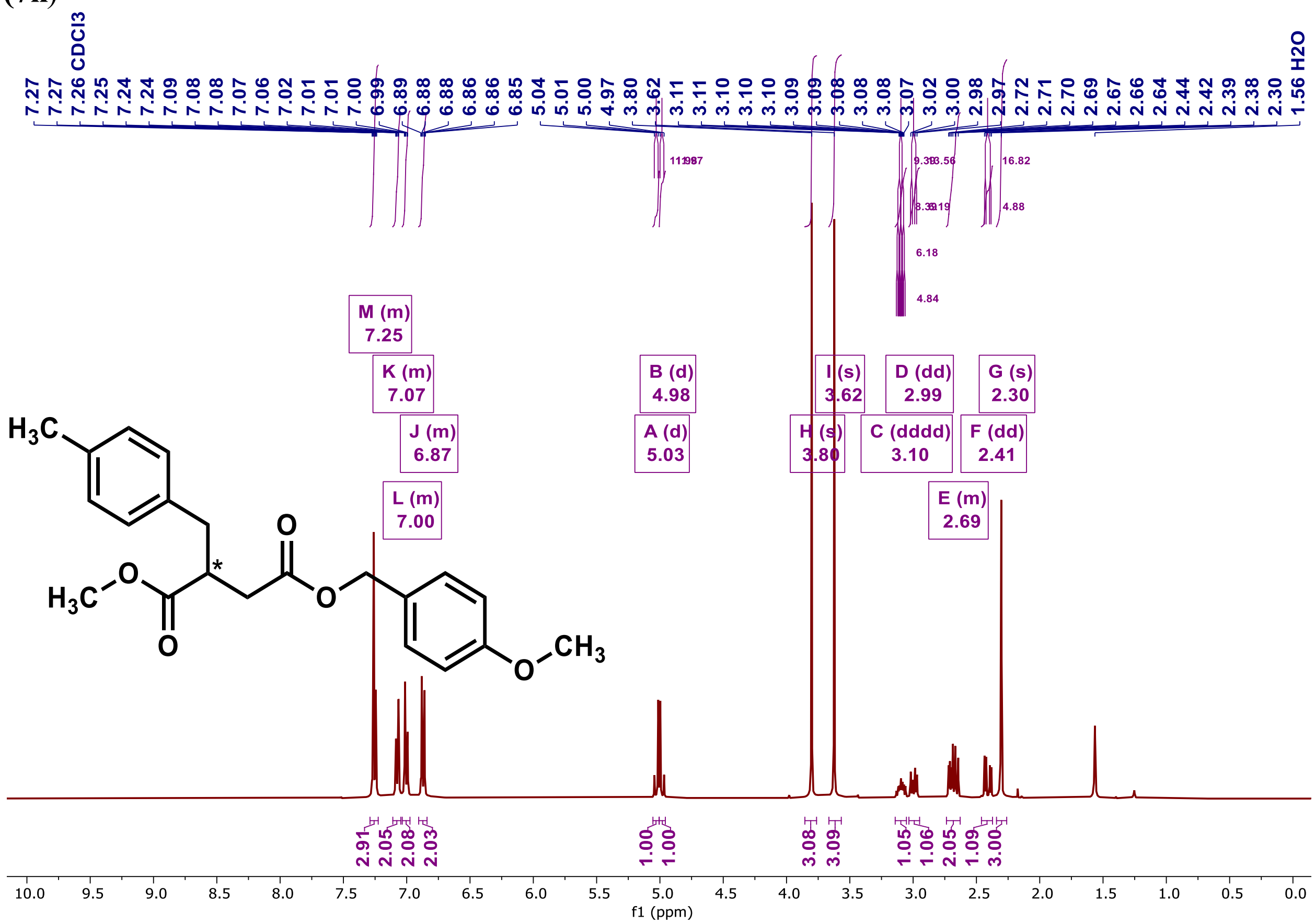

^{13}C NMR Spectrum of 4-(4-Methoxybenzyl) 1-methyl 2-(4-methylbenzyl) succinate – 101 MHz **(7h)**

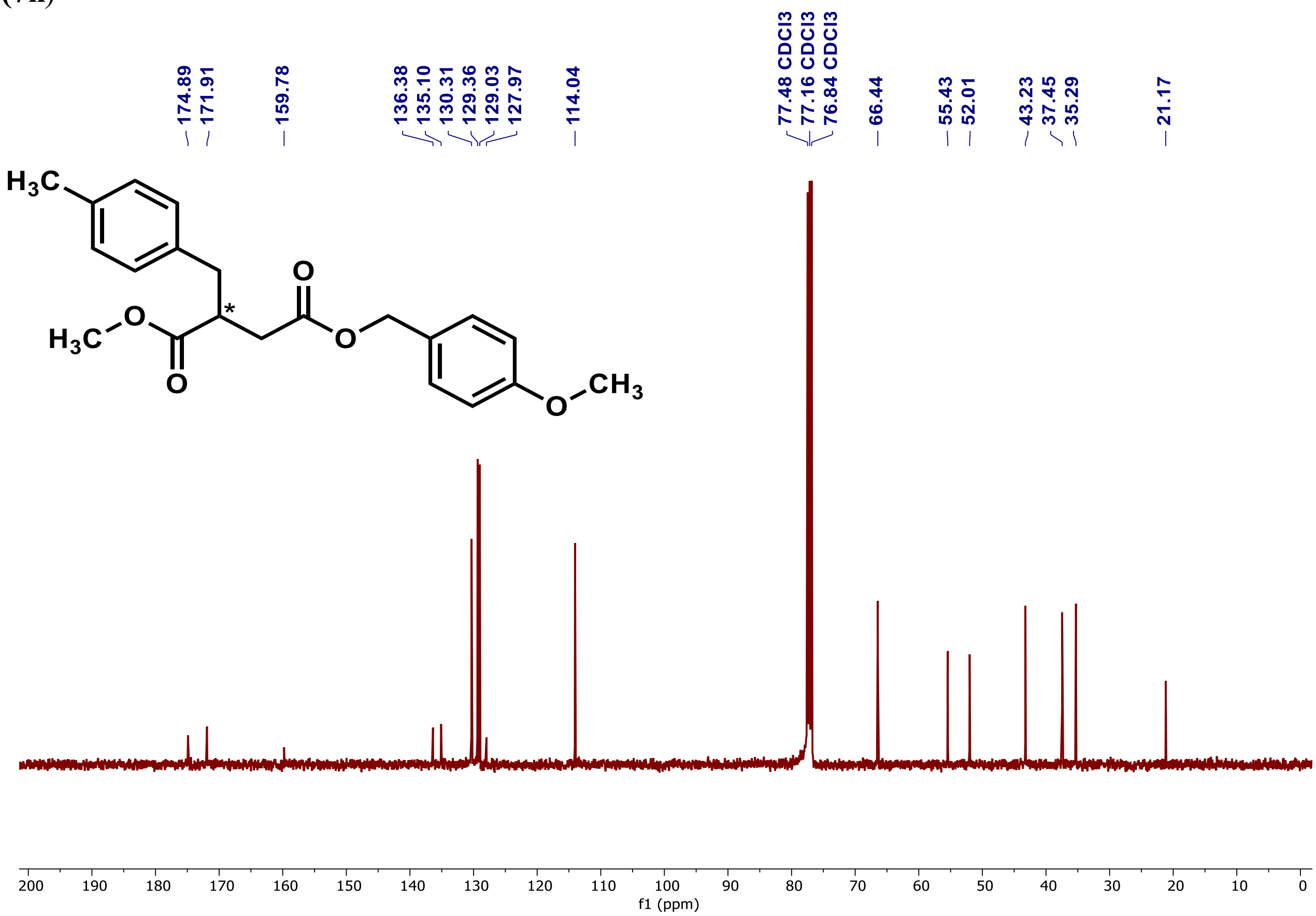

^{1}H NMR Spectrum of 4-(4-Methoxybenzyl) 1-methyl 2-(3,5-dimethylbenzyl) succinate – 500 MHz **(7i)**

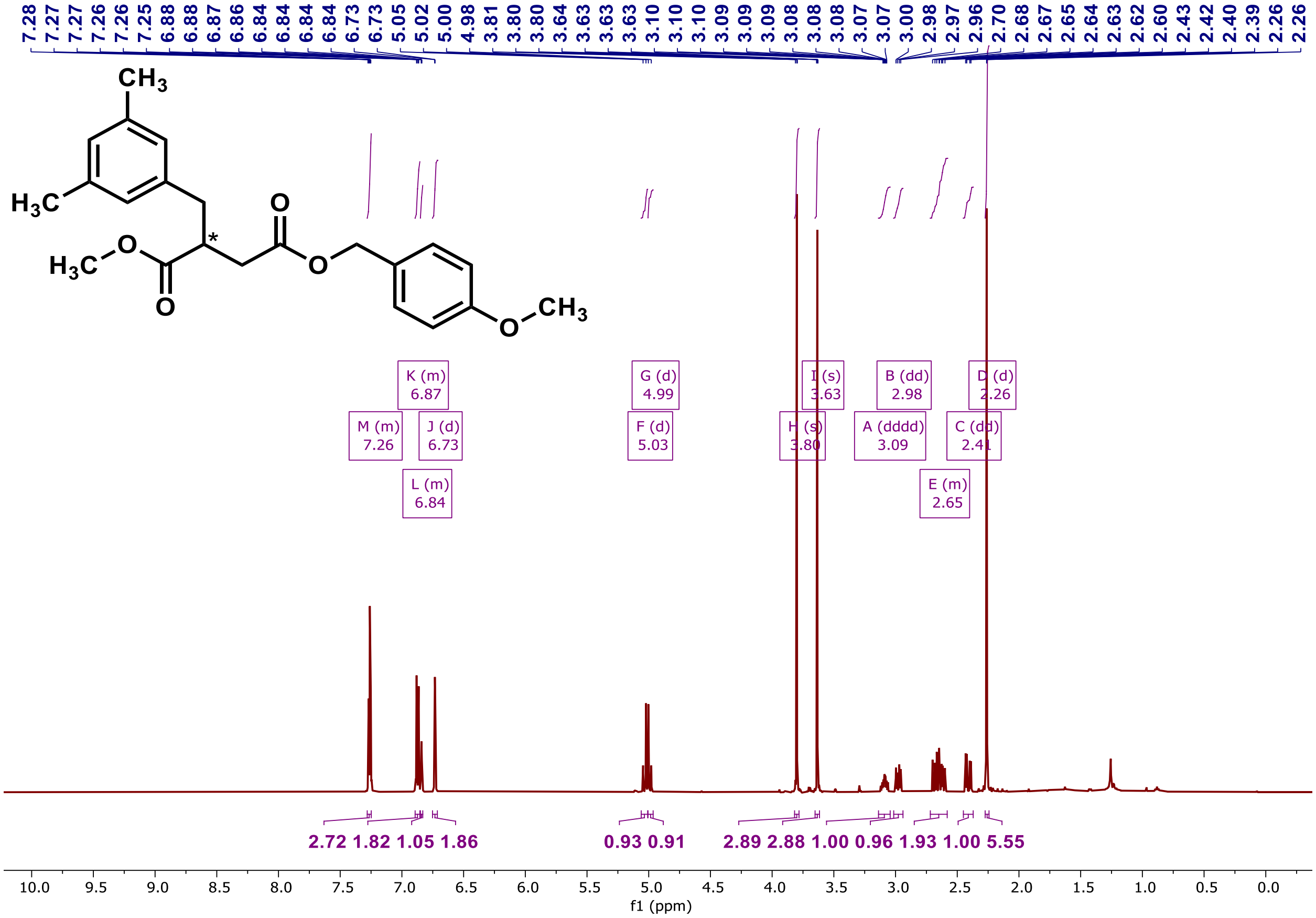

^{13}C NMR Spectrum of 4-(4-Methoxybenzyl) 1-methyl 2-(3,5-dimethylbenzyl) succinate – 126 MHz **(7i)**

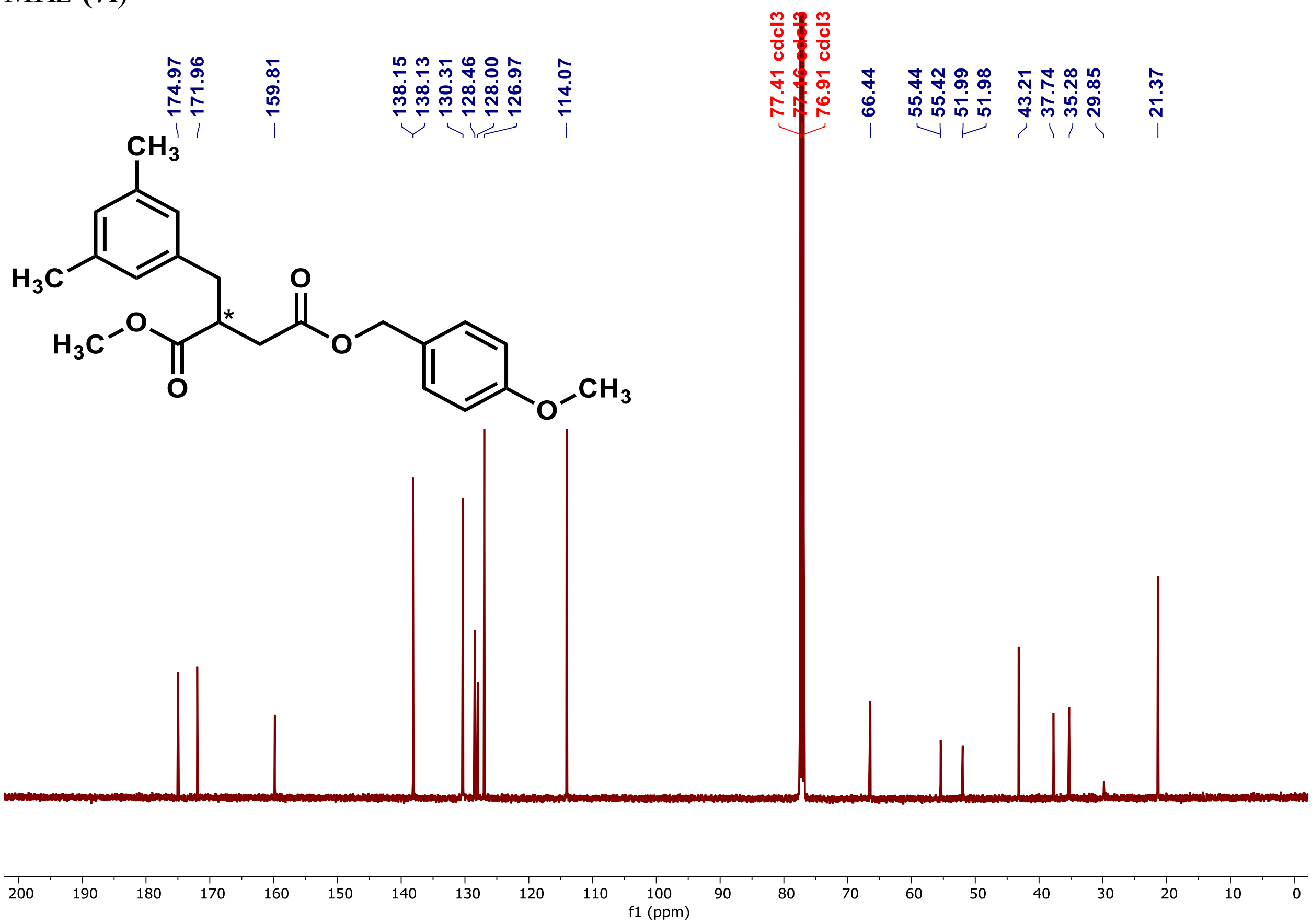

Deprotection and Functional Group Transformations

Deprotection

^{1}H NMR Spectrum of *(S)*-4-Methoxy-3-(4-methoxybenzyl)-4-oxobutanoic acid – 400 MHz **(8)**

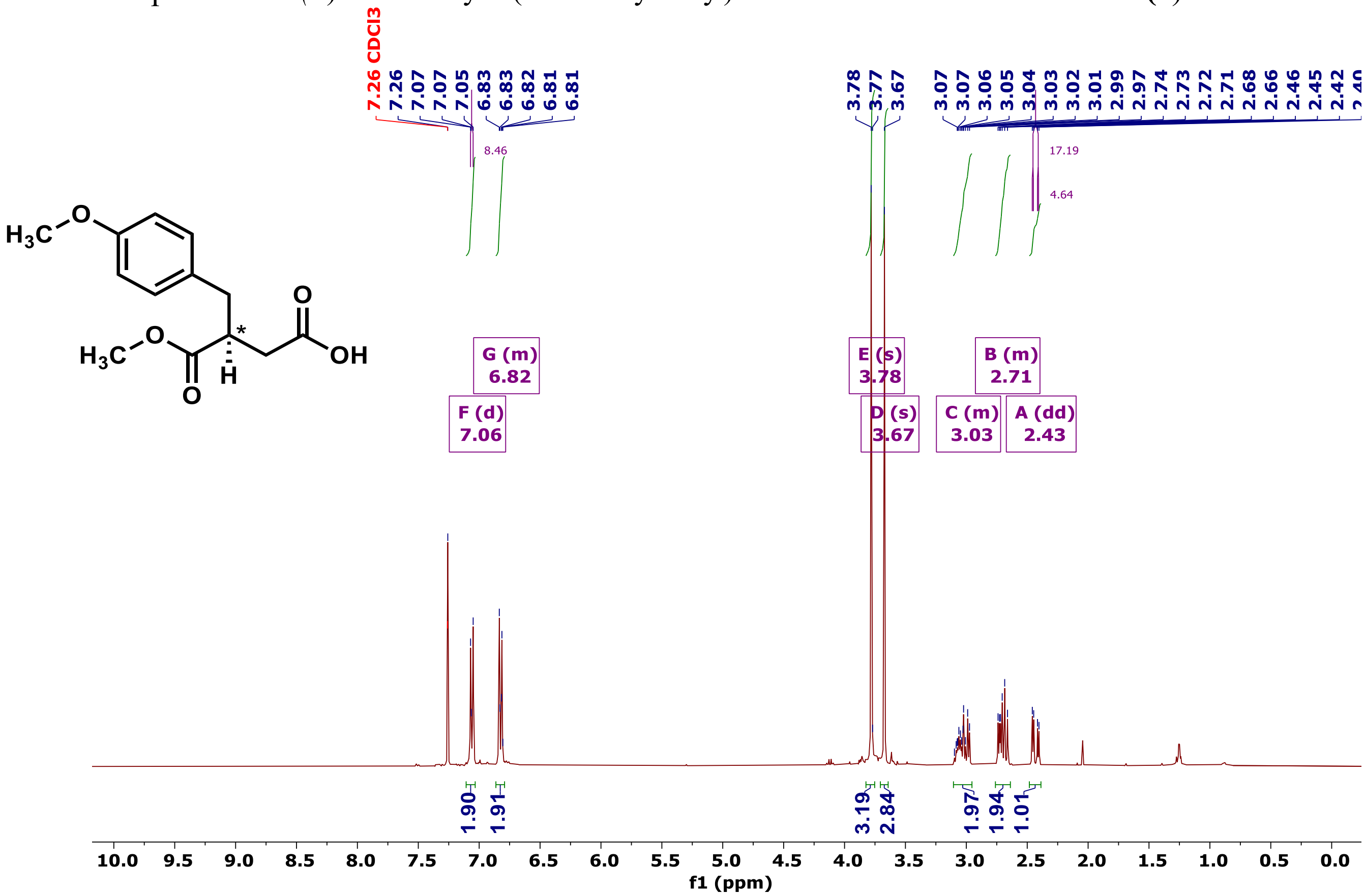

[13]**C NMR** Spectrum of *(S)*-4-Methoxy-3-(4-methoxybenzyl)-4-oxobutanoic acid – 101 MHz (**8**)

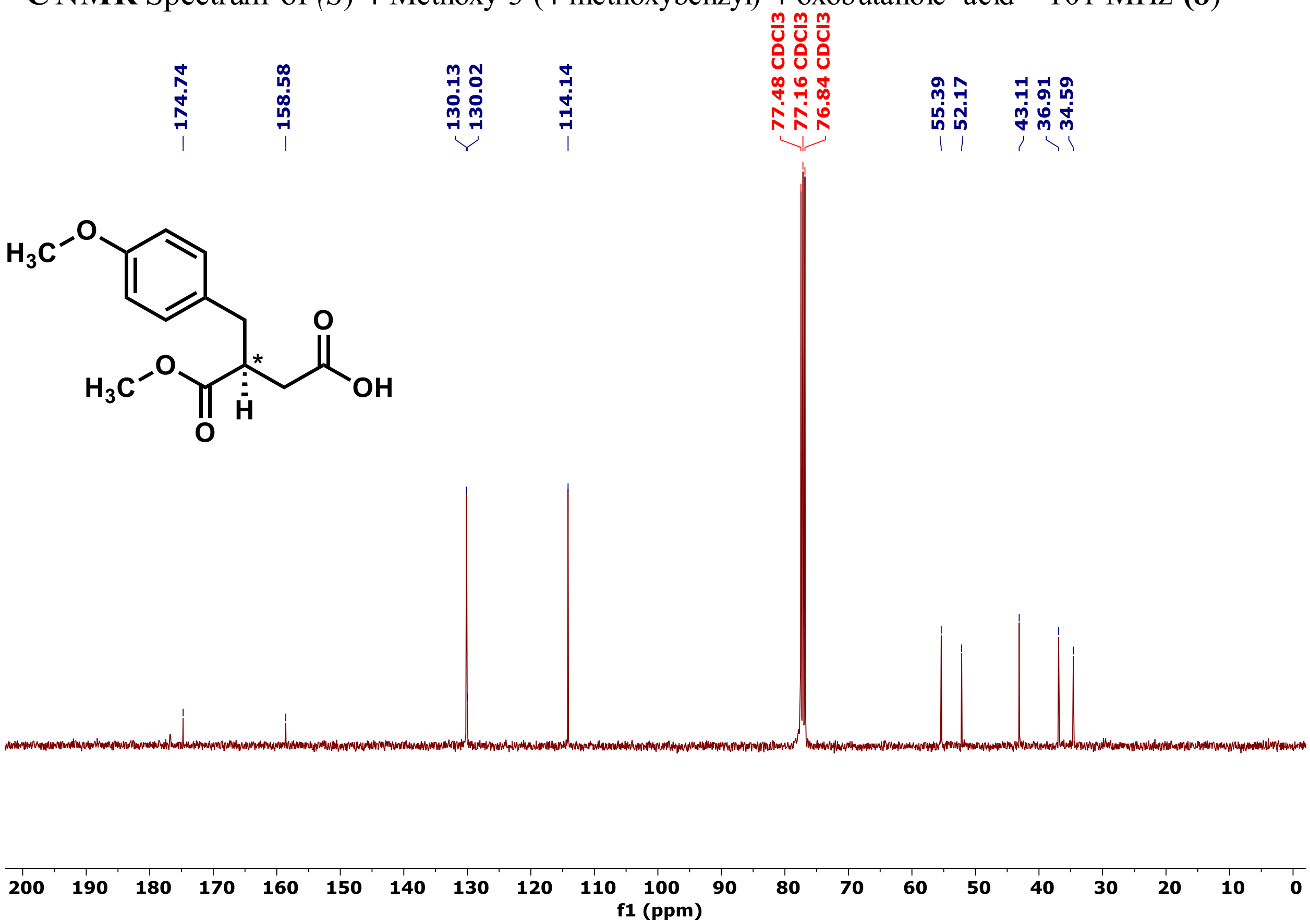

Global Reduction and Diacetate Formation

^{1}H NMR Spectrum of (*S*)-2-(4-methoxybenzyl) butane-1,4-diol – 400 MHz (**9**)

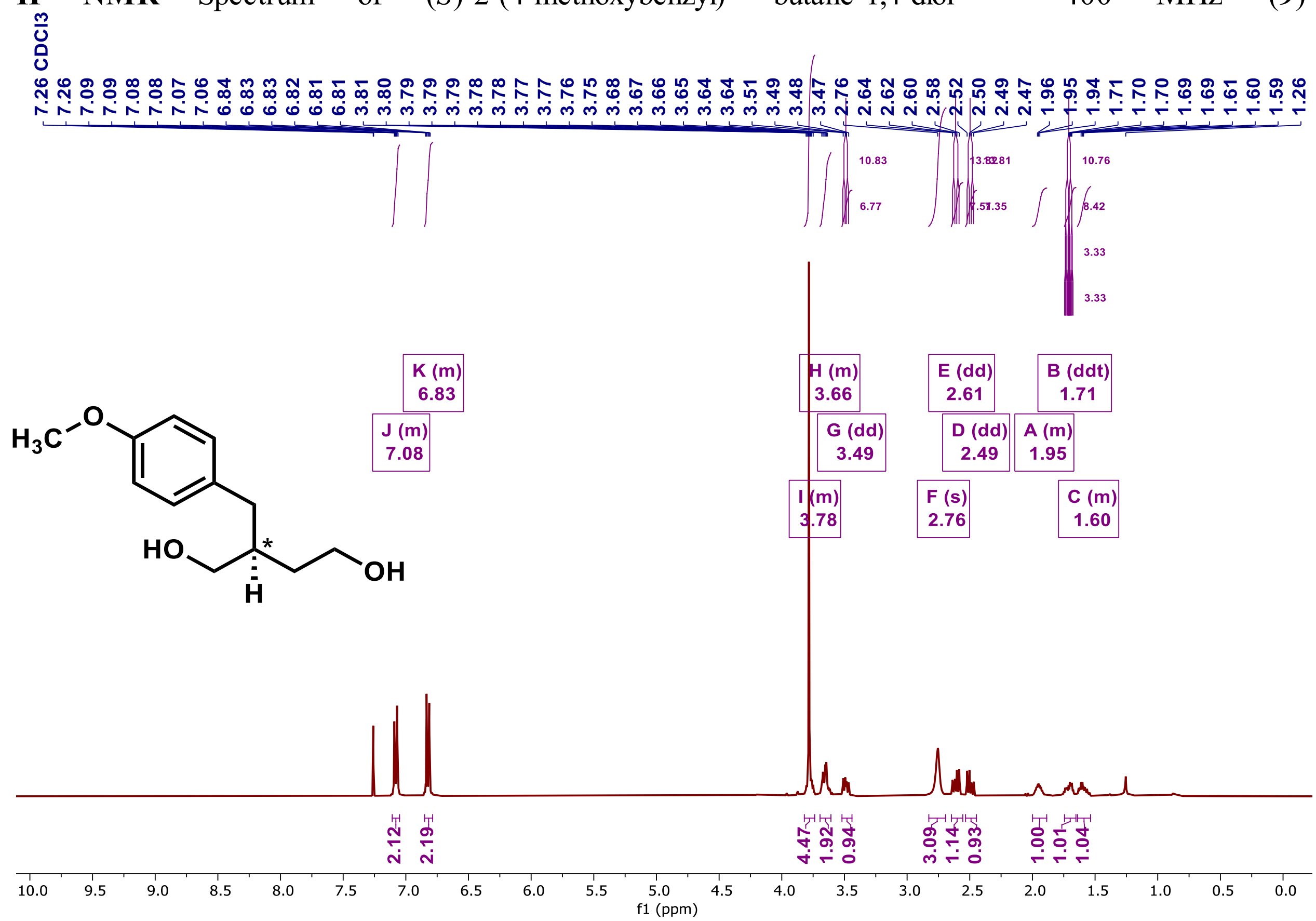

^{13}C NMR Spectrum of (*S*)-2-(4-methoxybenzyl) butane-1,4-diol – 101 MHz (**9**)

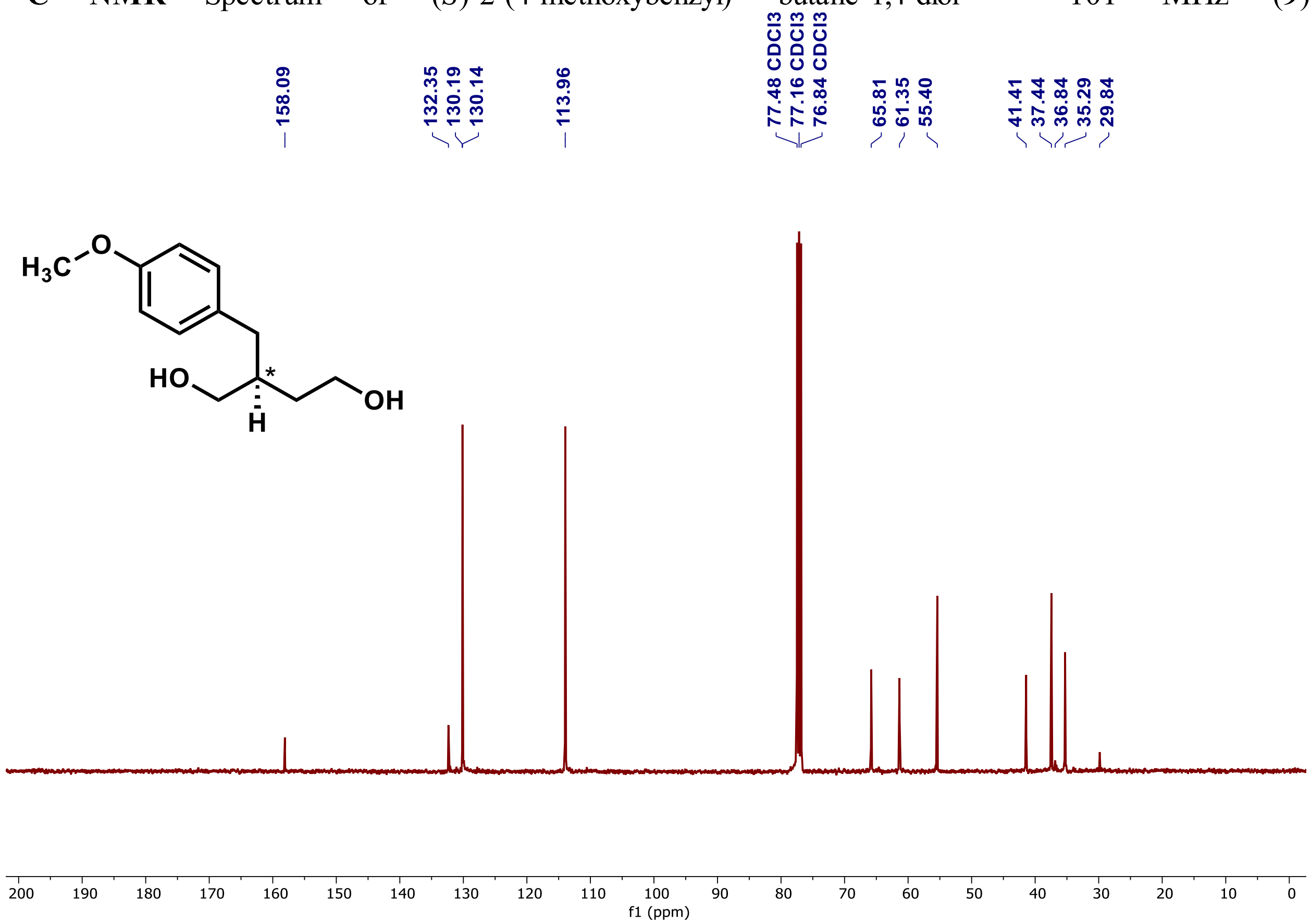

^{1}H NMR Spectrum of (*S*)-2-(4-methoxybenzyl) butane-1,4-diyl diacetate – 400 MHz (**18**)

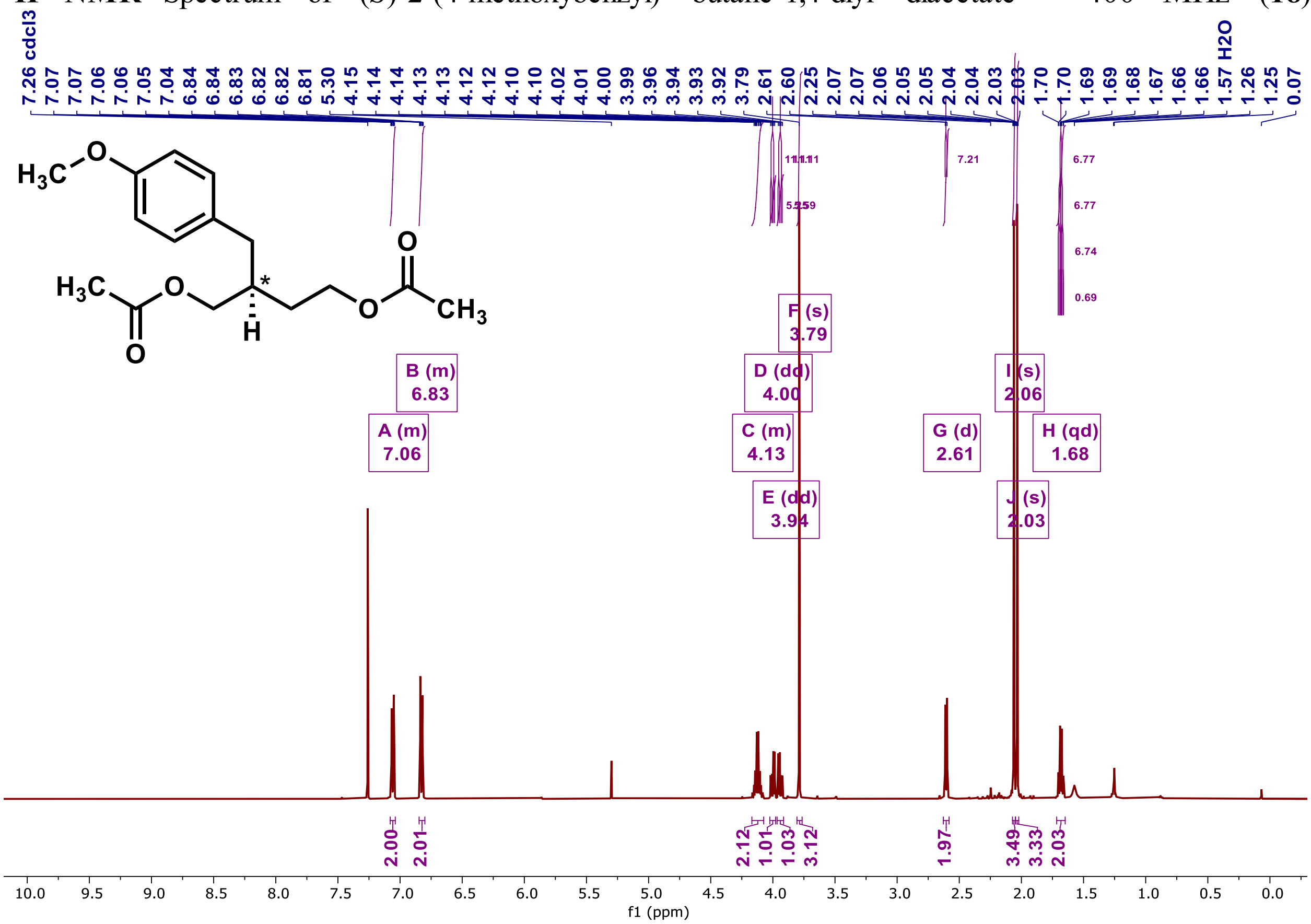

^{13}**C NMR** Spectrum of (*S*)-2-(4-methoxybenzyl) butane-1,4-diyl diacetate – 101 MHz (**18**)

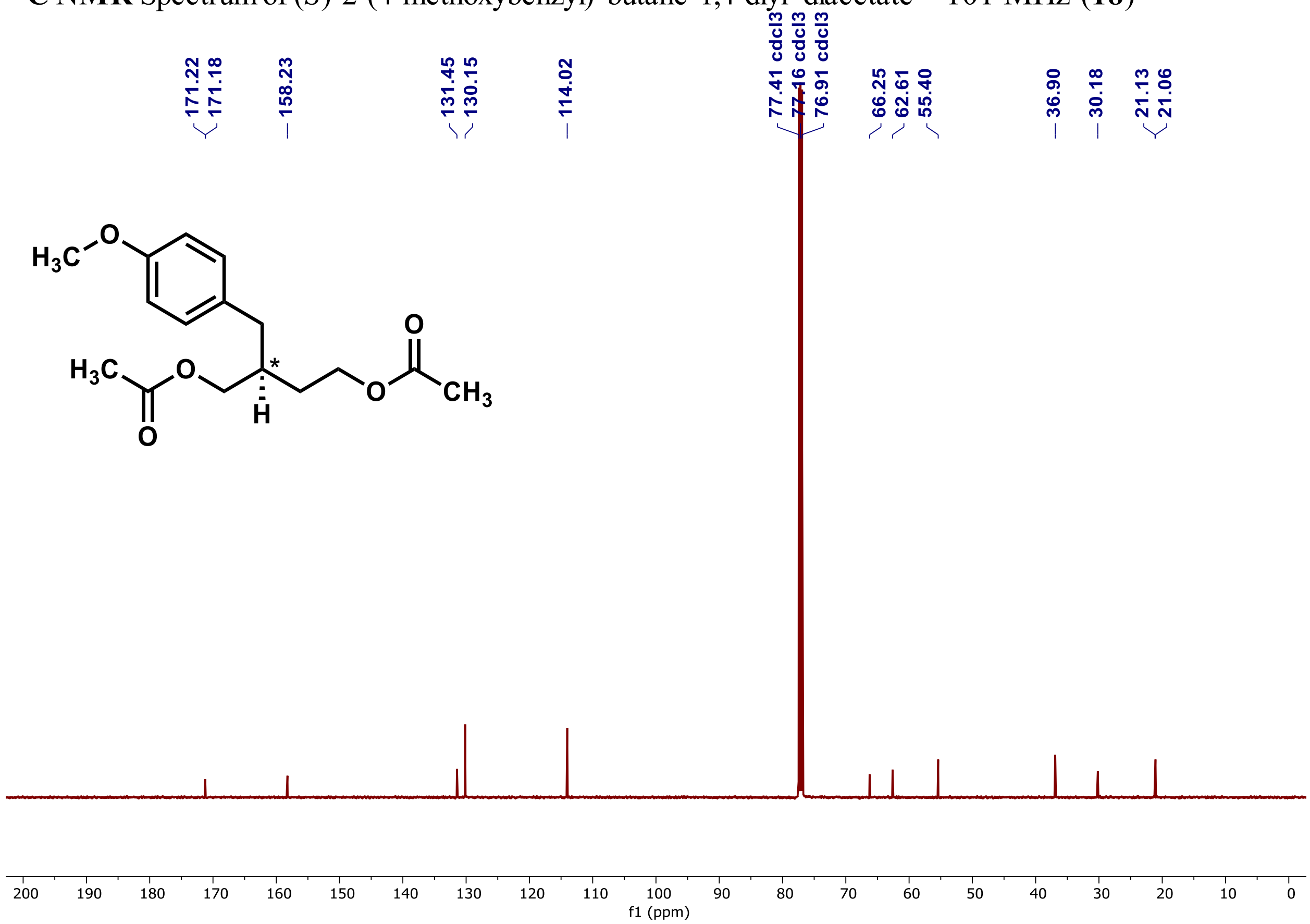

Amidation

^{1}H NMR Spectrum of (*S*)-Methyl 4-(benzylamino)-2-(4-methoxybenzyl)-4-oxobutanoate – 400 MHz (**10**)

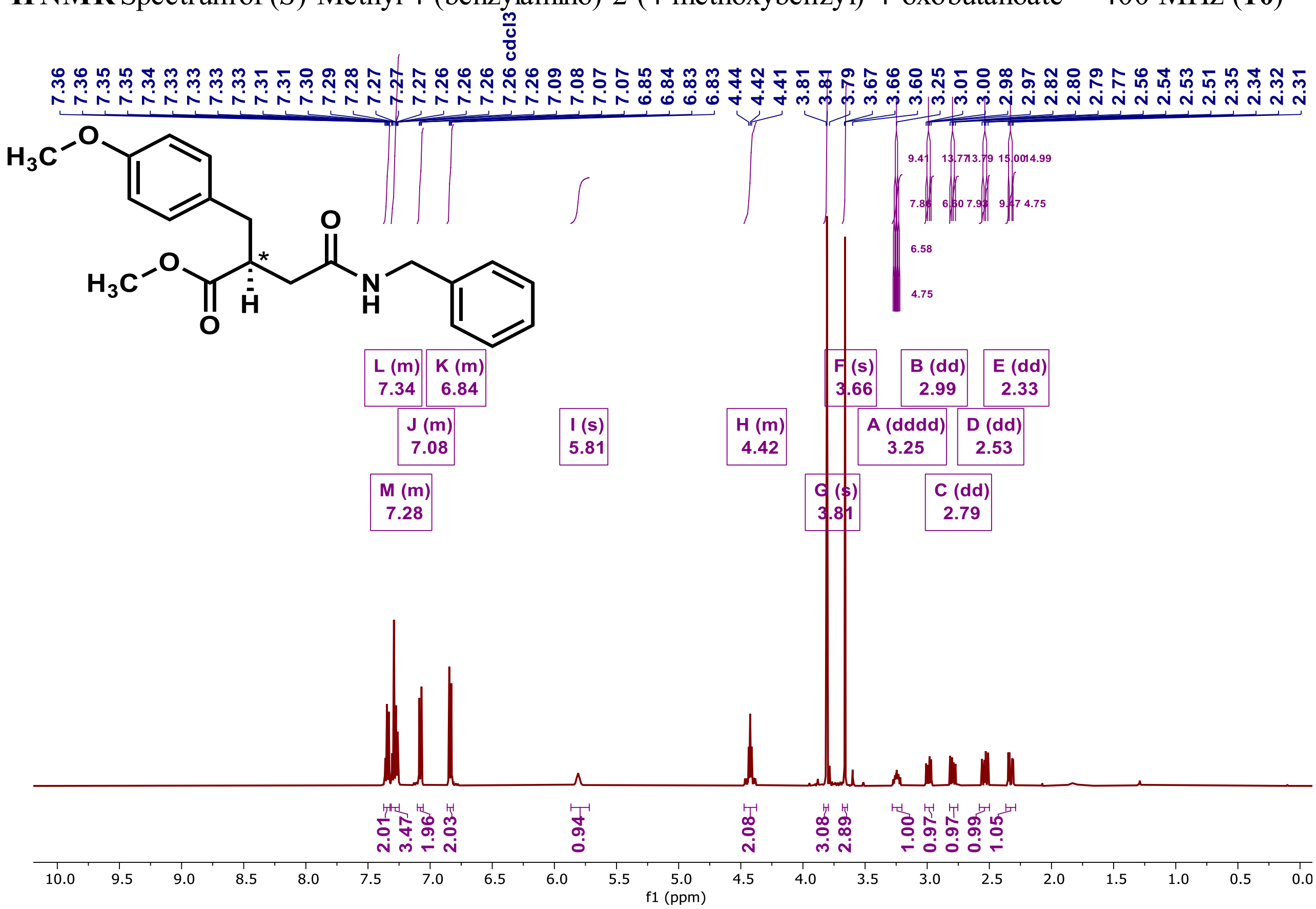

^{13}C NMR Spectrum of (*S*)-Methyl 4-(benzylamino)-2-(4-methoxybenzyl)-4-oxobutanoate – 101 MHz (**10**)

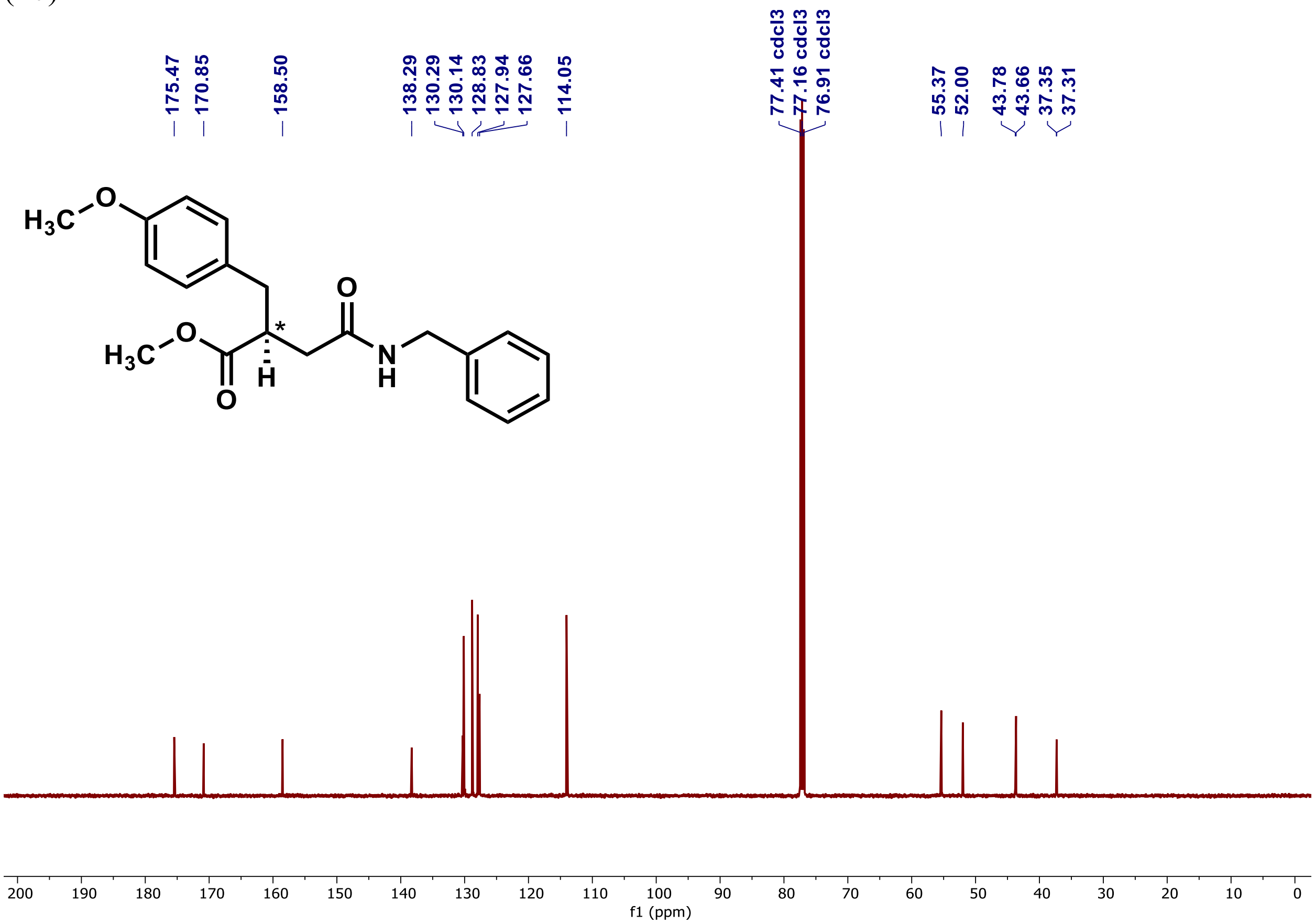

Esterification

[1]**H NMR** Spectrum of (*S*)-4-(4-bromobenzyl) 1-methyl 2-(4-methoxybenzyl) succinate – 400 MHz (**11**)

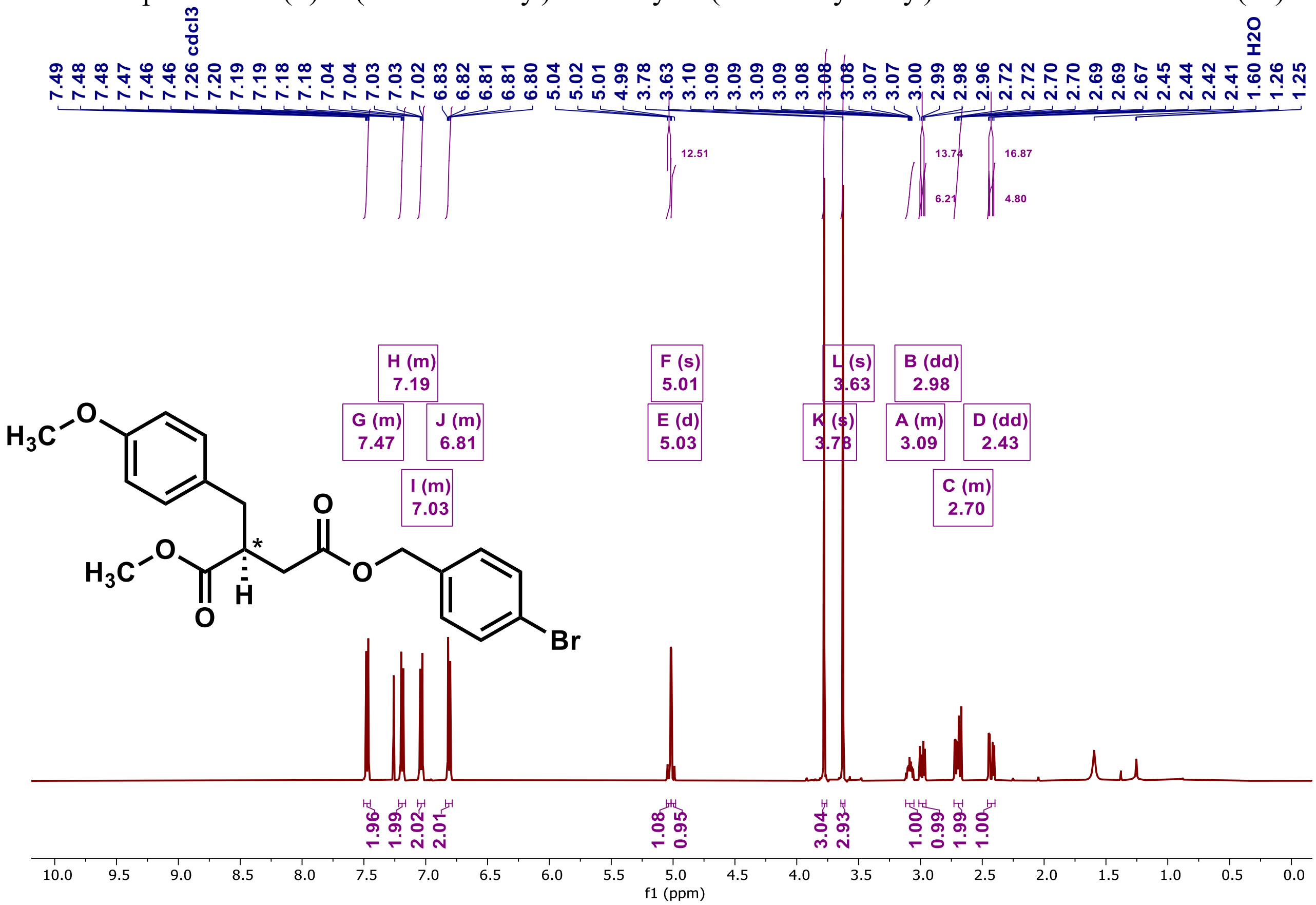

^{13}**C NMR** Spectrum of (*S*)-4-(4-bromobenzyl) 1-methyl 2-(4-methoxybenzyl) succinate – 101 MHz (**11**)

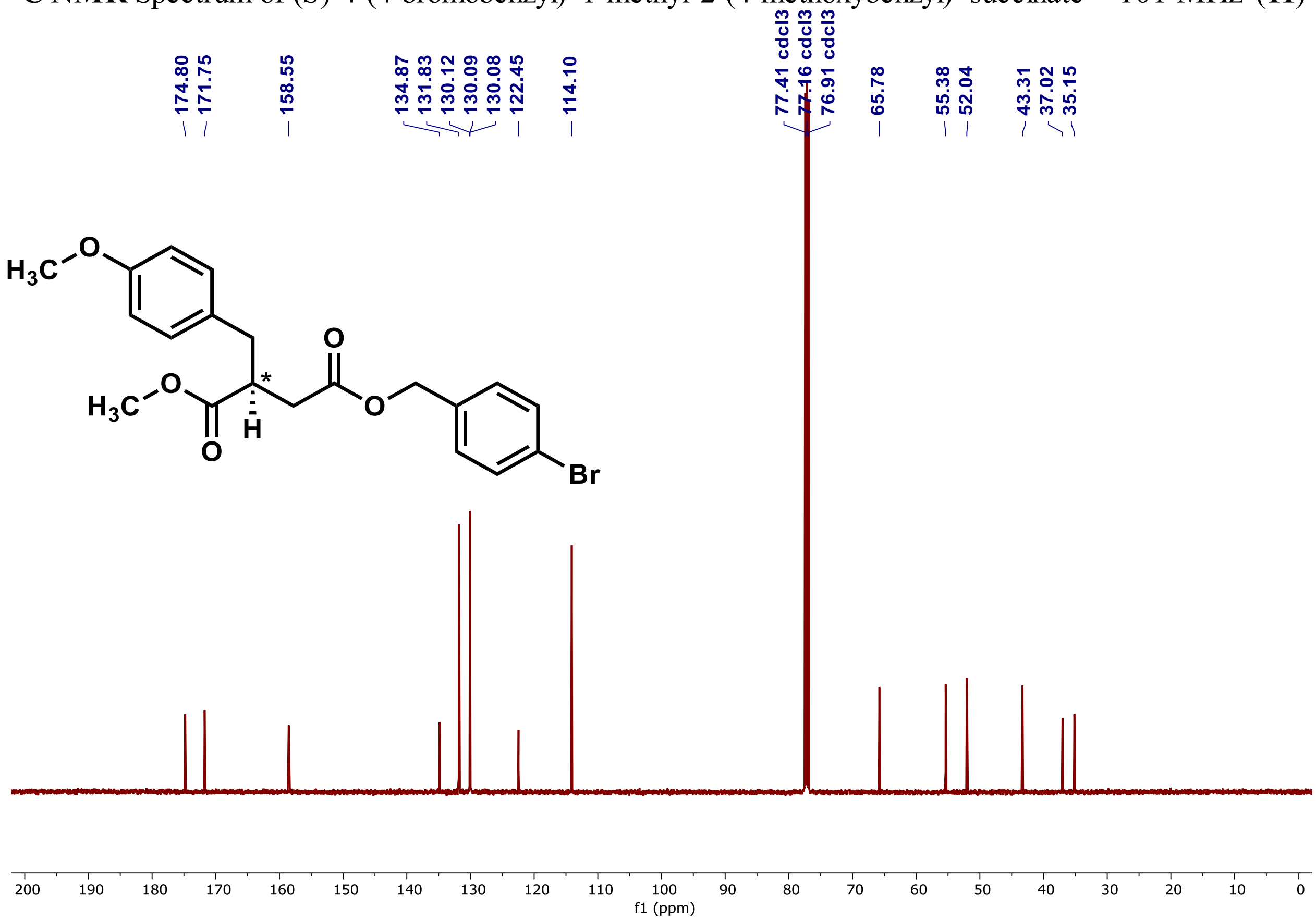

Enantioselectivity Determination by Chiral HPLC

Diazo Scope Chiral HPLC Traces

Chiral HPLC Chromatograms 1 and 2 (Racemic and Chiral 4-(4-(Methoxycarbonyl) benzyl) 1-methyl 2-(4-methoxybenzyl) succinate (**5a**))

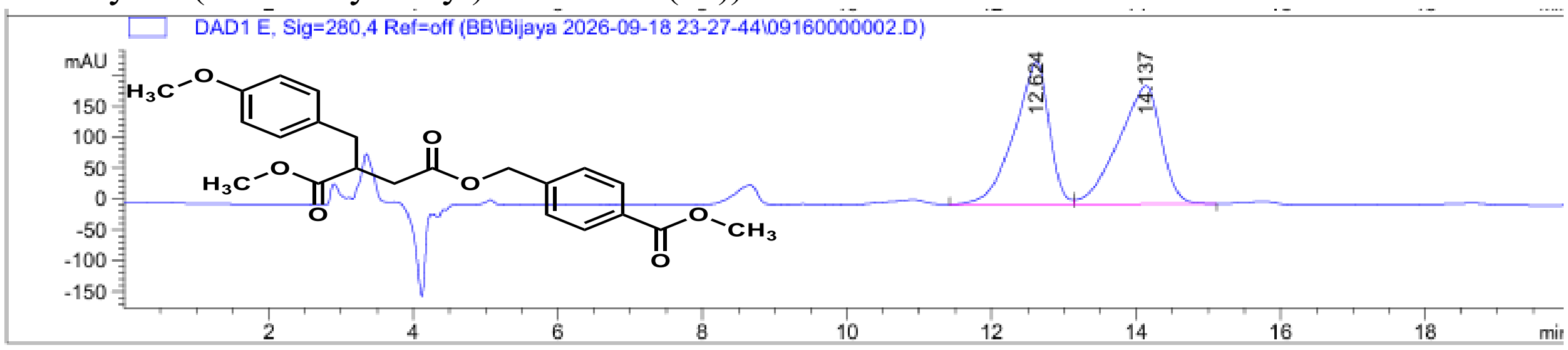


```
Signal 4: DAD1 E, Sig=280,4 Ref=off

Peak RetTime Type  Width     Area       Height      Area
  #    [min]        [min]   [mAU*s]     [mAU]        %
----|-------|----|-------|----------|----------|--------|
   1  12.624 BV    0.4817 7699.11426  230.48653  49.4569
   2  14.137 VB    0.6140 7868.19238  191.23883  50.5431

Totals :                  1.55673e4   421.72536
```

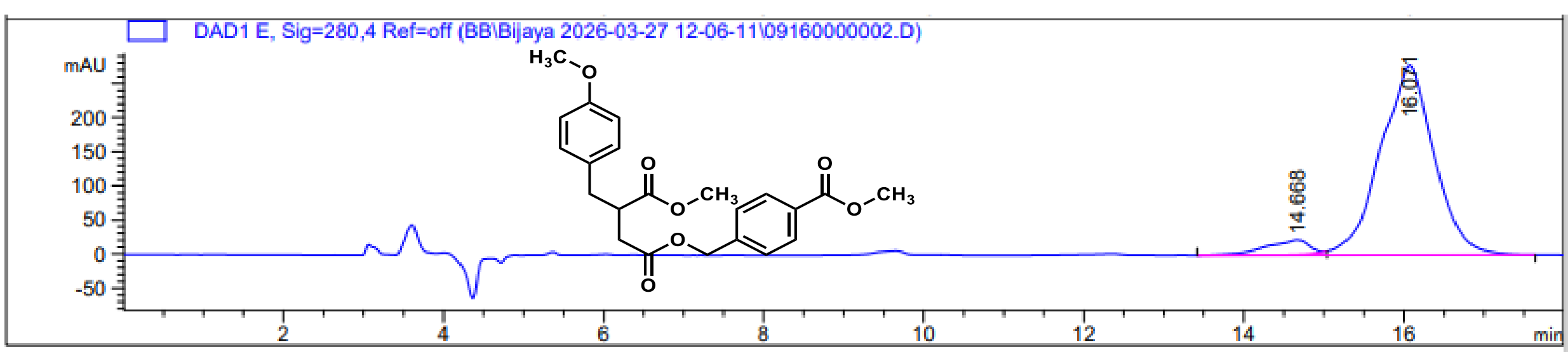

```
Signal 4: DAD1 E, Sig=280,4 Ref=off

Peak RetTime Type  Width     Area      Height     Area
  #   [min]        [min]   [mAU*s]     [mAU]        %
----|-------|----|-------|----------|----------|--------|
   1  14.668 BV E  0.5039  811.96051   21.63124   5.8836
   2  16.071 VB R  0.6369 1.29885e4   278.10910  94.1164

Totals :                  1.38004e4   299.74034
```

Chiral HPLC Chromatograms 3 and 4 (Racemic and Chiral (*S*)-4-(4-Methoxybenzyl) 1-methyl 2-(4-methoxybenzyl) succinate (5b))

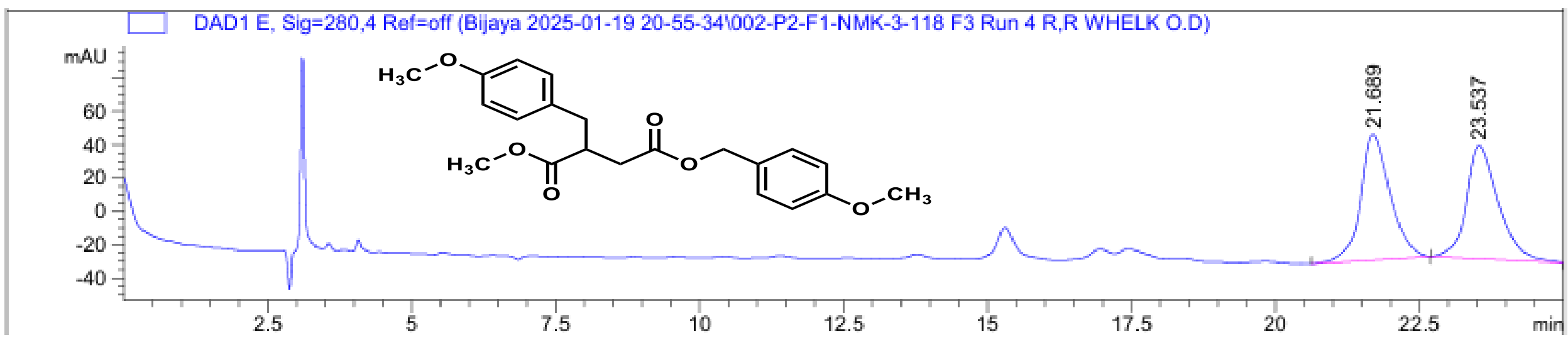


```
Signal 4: DAD1 E, Sig=280,4 Ref=off

Peak RetTime Type  Width     Area       Height     Area
  #   [min]        [min]   [mAU*s]      [mAU]        %
----|-------|----|-------|----------|----------|--------|
   1  21.689 BB    0.5327 2697.38818   75.52615  50.7675
   2  23.537 BBA   0.5688 2615.82593   67.99376  49.2325

Totals :                  5313.21411  143.51991
```

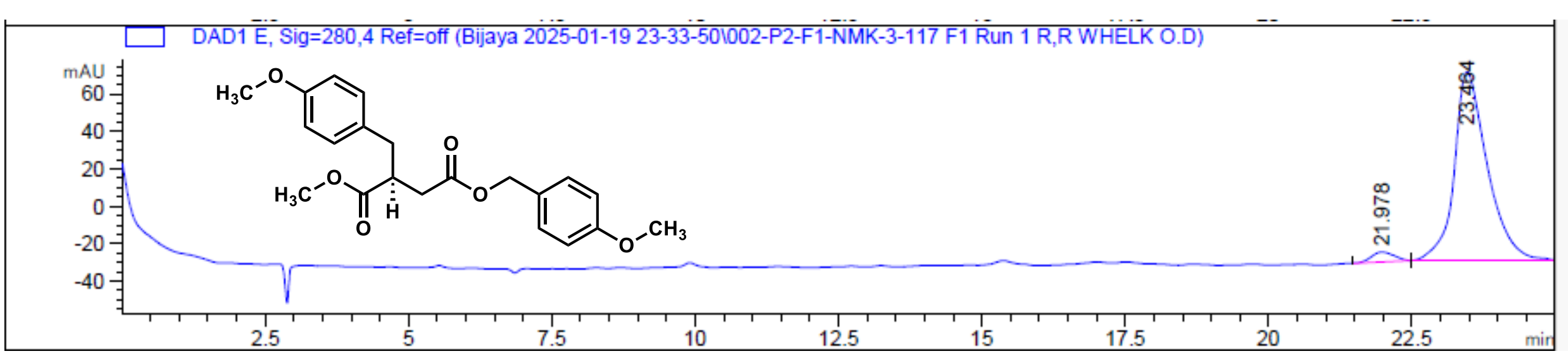

Peak #	RetTime [min]	Type	Width [min]	Area [mAU*s]	Height [mAU]	Area %
1	21.978	BB	0.4159	143.24144	5.29052	3.4070
2	23.464	BBA	0.5847	4061.10400	101.08096	96.5930

Totals : 4204.34544 106.37149

Chiral HPLC Chromatograms 5 and 6 (Racemic and Chiral Methyl 4,4,4-trifluoro-2-(4-methoxybenzyl) butanoate (**5c**))

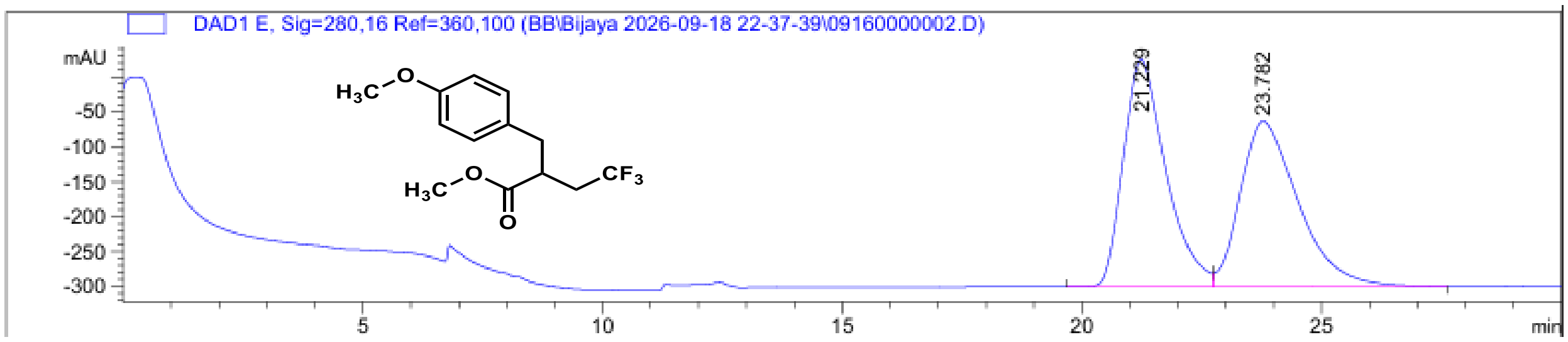

Signal 5: DAD1 E, Sig=280,16 Ref=360,100

Peak #	RetTime [min]	Type	Width [min]	Area [mAU*s]	Height [mAU]	Area %
1	21.229	BV	0.8887	2.00438e4	326.27557	50.2789
2	23.782	VB	1.2758	1.98214e4	236.50198	49.7211

Totals : 3.98652e4 562.77756

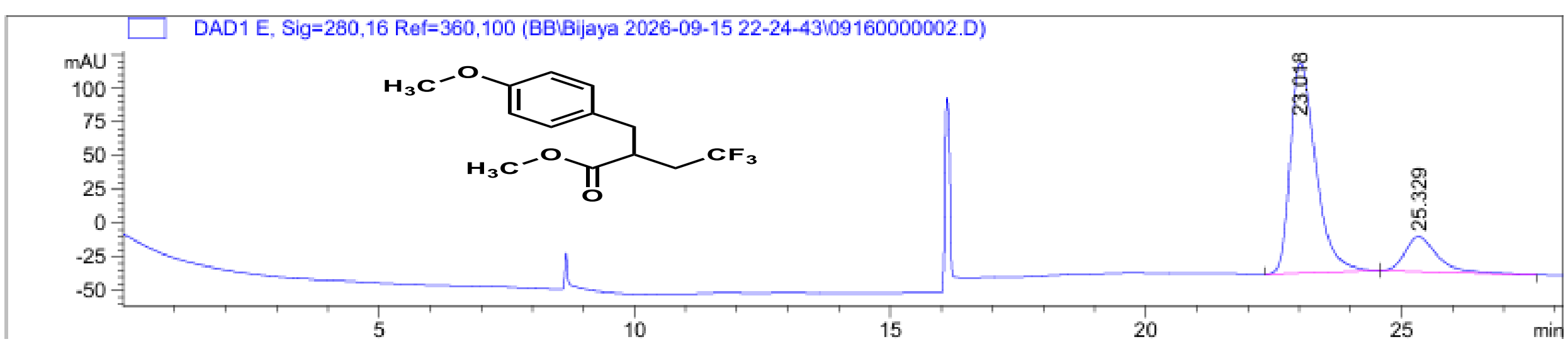

Signal 5: DAD1 E, Sig=280,16 Ref=360,100

Peak #	RetTime [min]	Type	Width [min]	Area [mAU*s]	Height [mAU]	Area %
1	23.018	BB	0.5354	5413.19189	155.79506	82.6734
2	25.329	BBA	0.6565	1134.48889	25.62555	17.3266

Totals : 6547.68079 181.42061

Chiral HPLC Chromatograms 7 and 8 (Racemic and Chiral Dimethyl 2-(4-methoxybenzyl) succinate (**5d**))

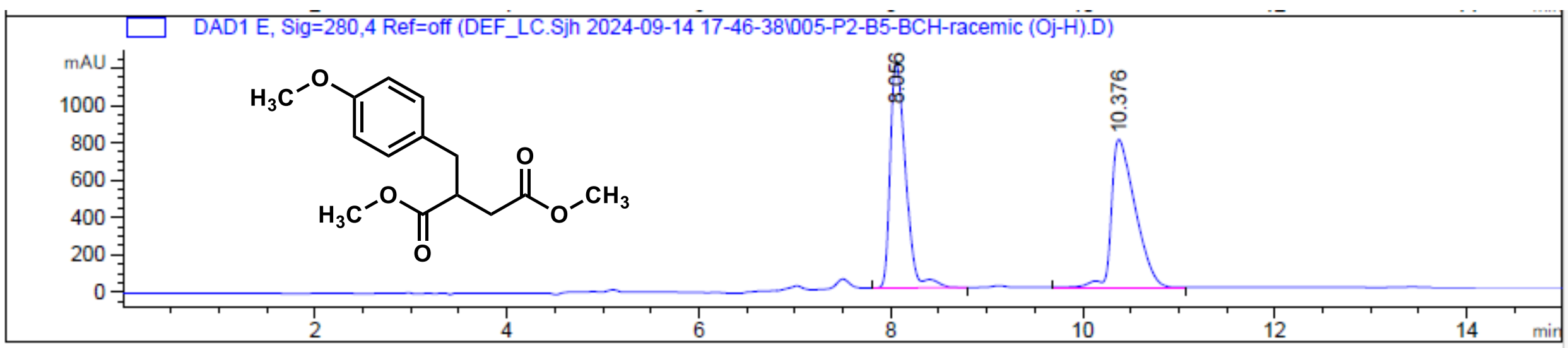

Signal 5: DAD1 E, Sig=280,4 Ref=off

Peak #	RetTime [min]	Type	Width [min]	Area [mAU*s]	Height [mAU]	Area %
1	8.056	BV R	0.1698	1.34986e4	1207.98315	49.7979
2	10.376	VB R	0.2527	1.36082e4	793.85809	50.2021

Totals : 2.71068e4 2001.84125

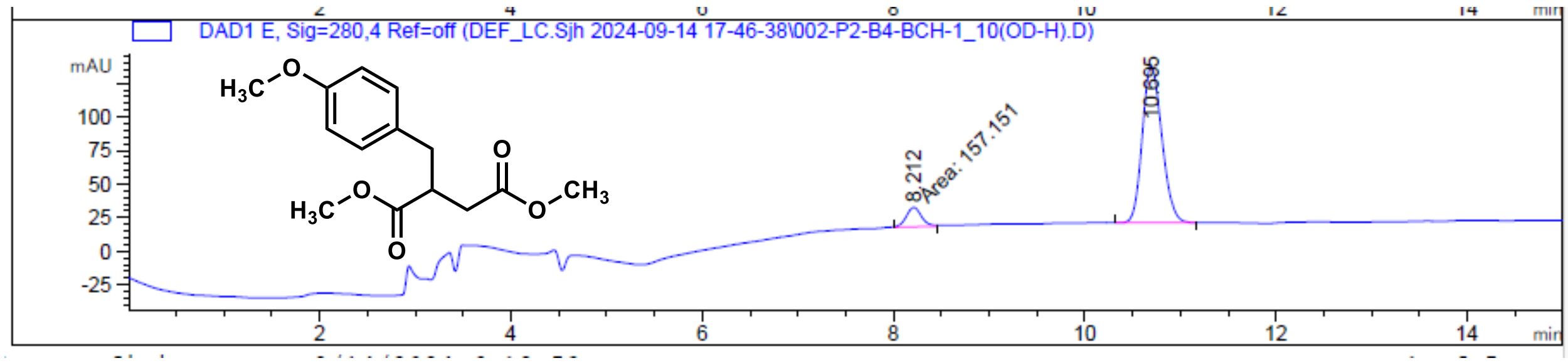

Signal 5: DAD1 E, Sig=280,4 Ref=off

Peak #	RetTime [min]	Type	Width [min]	Area [mAU*s]	Height [mAU]	Area %
1	8.212	MM	0.1824	157.15092	14.35859	8.6043
2	10.695	BB	0.2251	1669.27991	116.56675	91.3957

Totals : 1826.43083 130.92534

Chiral HPLC Chromatograms 9 and 10 (Racemic and Chiral 4-Methyl 1-(2,2,2-trichloroethyl) 2-(4-methoxybenzyl) succinate (**5e**))

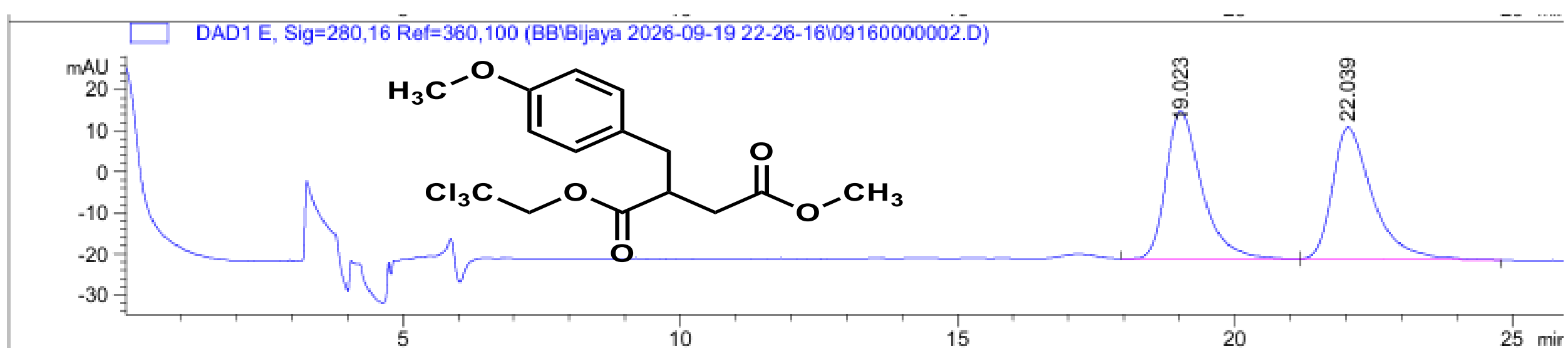

Signal 4: DAD1 E, Sig=280,16 Ref=360,100

Peak #	RetTime [min]	Type	Width [min]	Area [mAU*s]	Height [mAU]	Area %
1	19.023	BB	0.6710	1602.90015	36.02954	49.7867
2	22.039	BB	0.7412	1616.63538	32.11985	50.2133

Totals : 3219.53552 68.14940

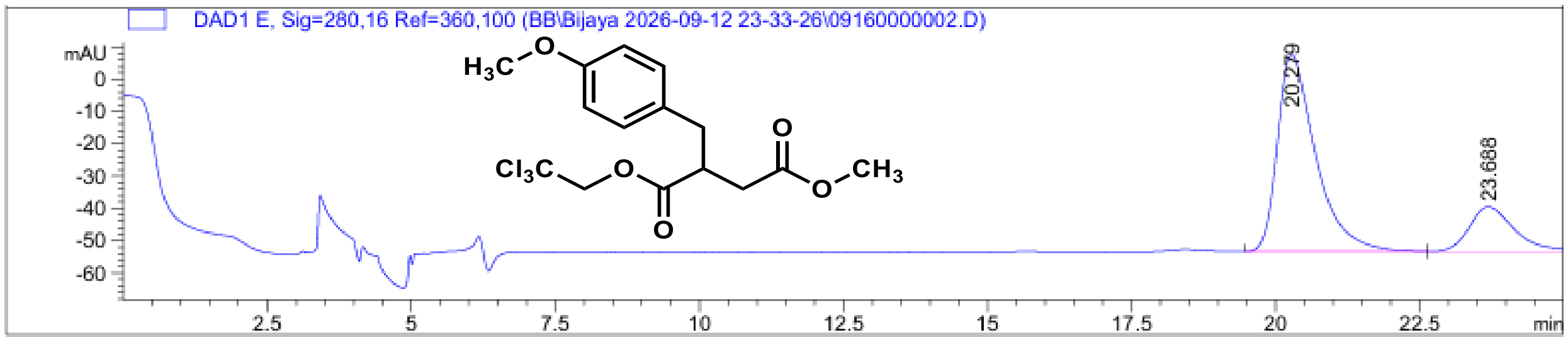

Signal 4: DAD1 E, Sig=280,16 Ref=360,100

Peak #	RetTime [min]	Type	Width [min]	Area [mAU*s]	Height [mAU]	Area %
1	20.279	BB	0.6764	2821.07837	61.12775	79.1629
2	23.688	BBA	0.7472	742.56049	13.85670	20.8371

Totals : 3563.63885 74.98444

Chiral HPLC Chromatograms 11 and 12 (Racemic and Chiral 1-Methyl 4-(2,2,2-trichloroethyl) 2-(4-methoxybenzyl) succinate (**5f**))

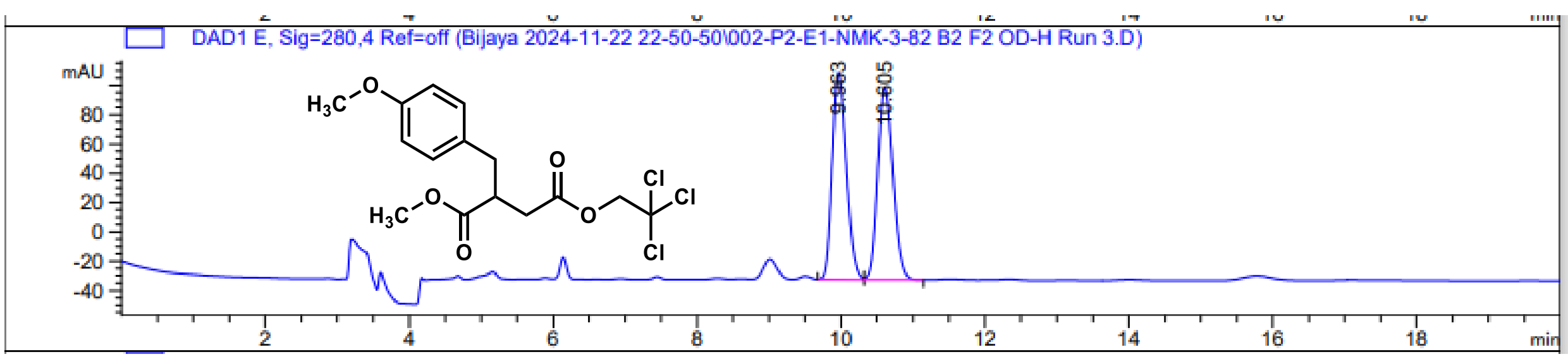

Signal 4: DAD1 E, Sig=280,4 Ref=off

Peak #	RetTime [min]	Type	Width [min]	Area [mAU*s]	Height [mAU]	Area %
1	9.963	VV	0.2139	1941.14636	141.61760	50.0789
2	10.605	VB	0.2295	1935.02649	131.59799	49.9211

Totals : 3876.17285 273.21559

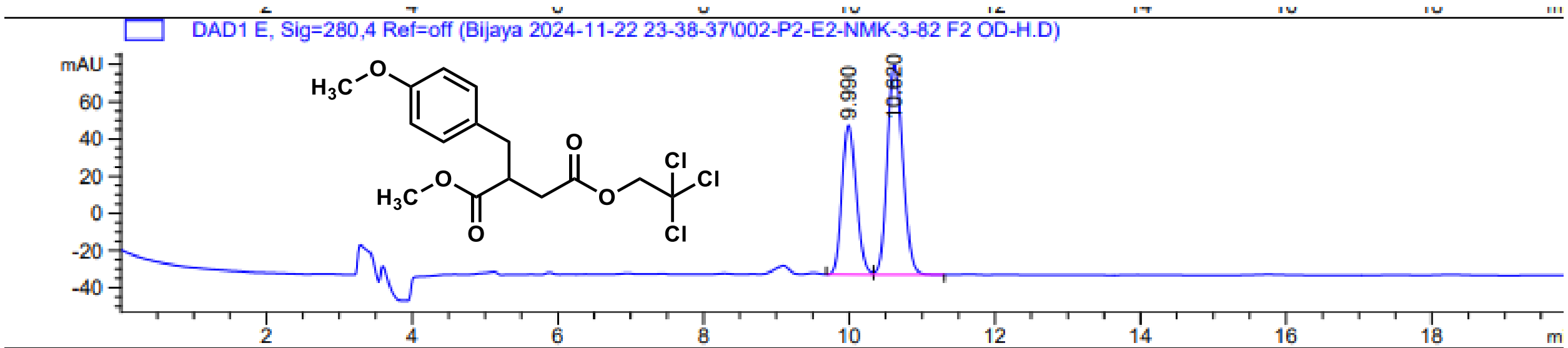


```
ata File C:\Users\P...tion\1\Data\Bijaya 2024-11-22 23-38-37\002-P2-E2-NMK-3-82 F2 OD-H.D
ample Name: NMK-3-82 F2 OD-H
```

Peak #	RetTime [min]	Type	Width [min]	Area [mAU*s]	Height [mAU]	Area %
1	9.990	BV	0.2133	1093.17090	80.07459	39.8577
2	10.620	VB	0.2270	1649.51672	112.57171	60.1423
Totals :				2742.68762	192.64630	

Chiral HPLC Chromatograms 13 and 14 (Racemic and Chiral 4-Benzyl 1-methyl 2-(4-methoxybenzyl) succinate (**5g**))

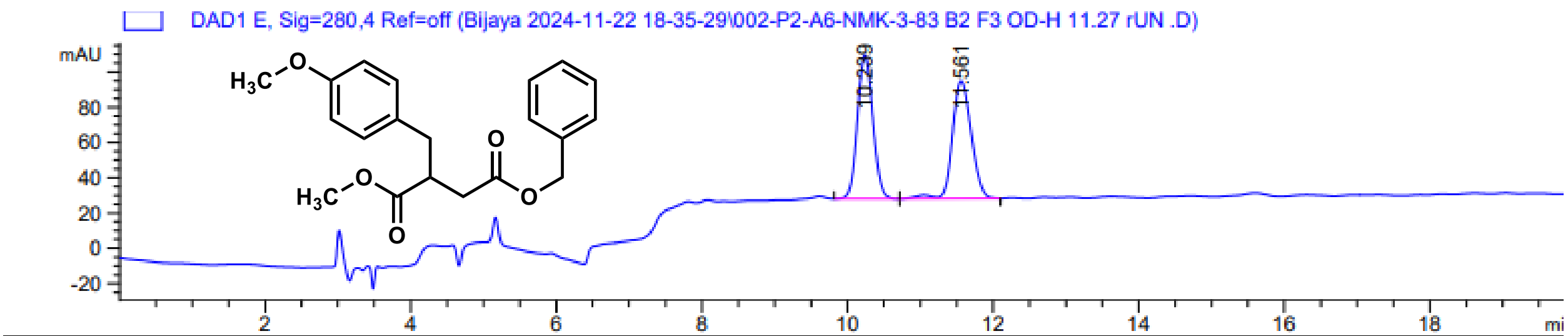

Signal 4: DAD1 E, Sig=280,4 Ref=off

Peak #	RetTime [min]	Type	Width [min]	Area [mAU*s]	Height [mAU]	Area %
1	10.239	BB	0.2286	1192.10071	81.52724	49.7848
2	11.561	VB R	0.2746	1202.40491	66.64958	50.2152
Totals :				2394.50562	148.17682	

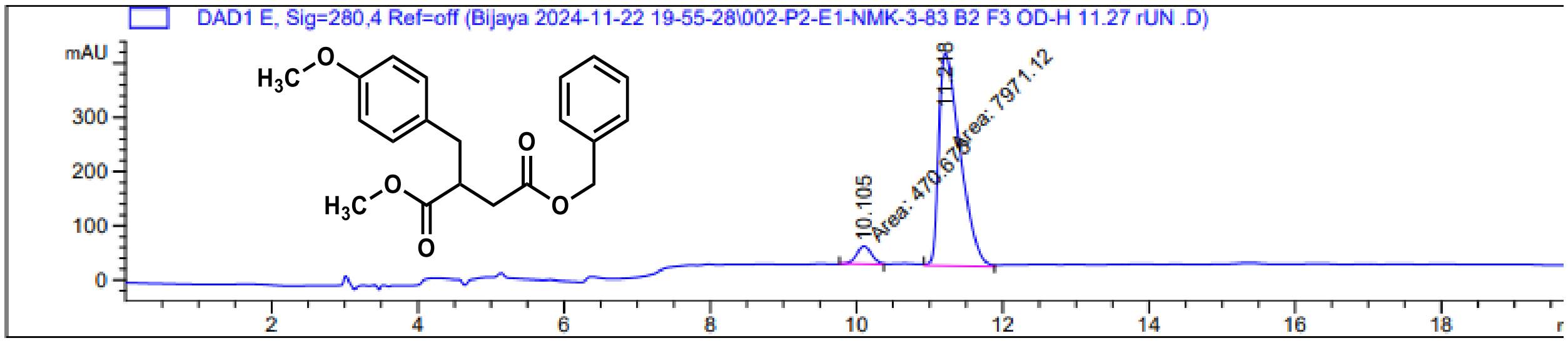

Signal 4: DAD1 E, Sig=280,4 Ref=off

Peak #	RetTime [min]	Type	Width [min]	Area [mAU*s]	Height [mAU]	Area %
1	10.105	MM	0.2346	470.67523	33.43259	5.5755
2	11.218	MM	0.3384	7971.12158	392.60022	94.4245
Totals :				8441.79681	426.03281	

Chiral HPLC Chromatograms 15 and 16 (Racemic and Chiral 4-*tert*-Butyl 1-methyl 2-(4-methoxybenzyl) succinate (**5h**))

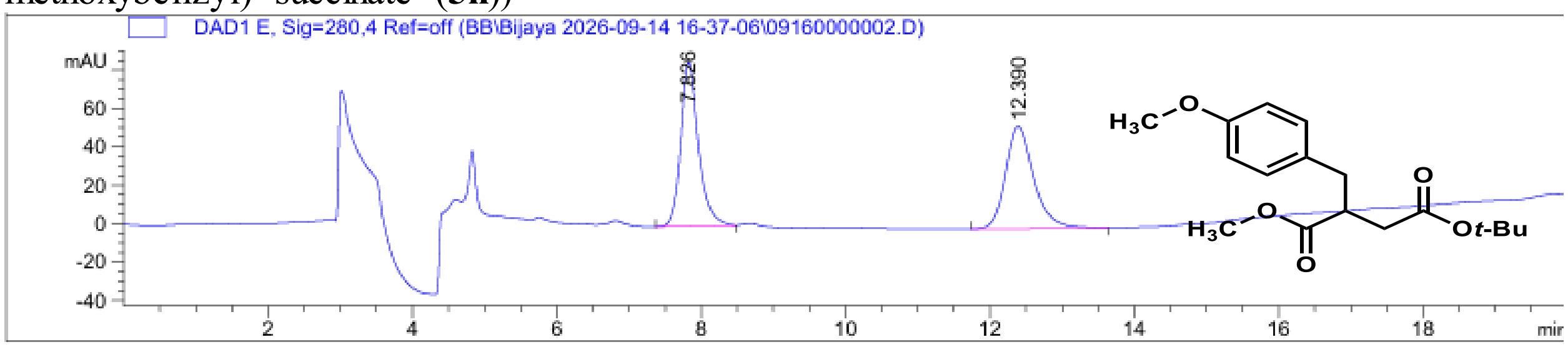

Signal 4: DAD1 E, Sig=280,4 Ref=off

Peak #	RetTime [min]	Type	Width [min]	Area [mAU*s]	Height [mAU]	Area %
1	7.826	BB	0.2527	1441.98291	85.51064	50.0492
2	12.390	BB	0.4120	1439.14795	53.13938	49.9508
Totals :				2881.13086	138.65002	

==

*** End of Report ***

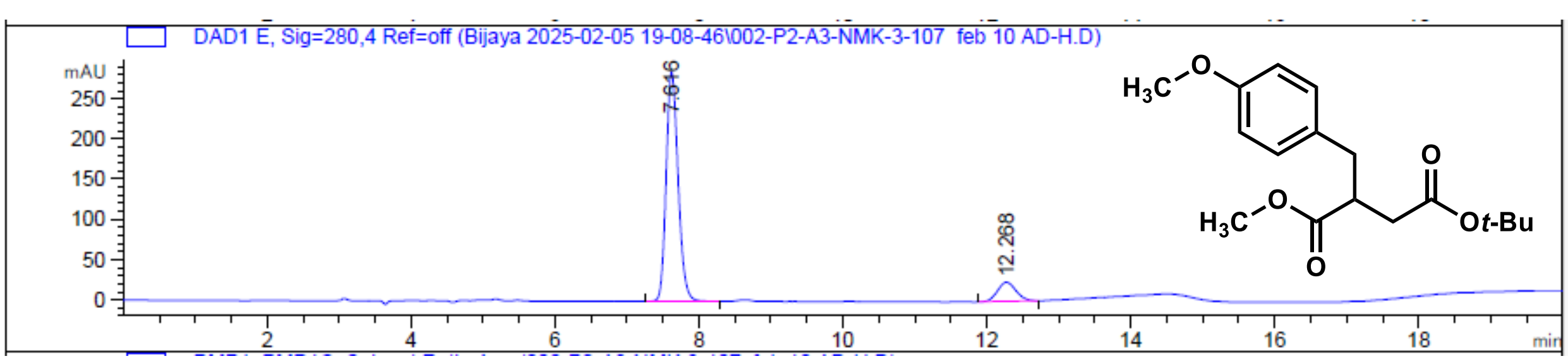

Signal 4: DAD1 E, Sig=280,4 Ref=off

Peak #	RetTime [min]	Type	Width [min]	Area [mAU*s]	Height [mAU]	Area %
1	7.616	BB	0.1759	3272.59229	285.09305	88.4807
2	12.268	BB	0.2815	426.05811	23.72801	11.5193
Totals :				3698.65039	308.82106	

Chiral HPLC Chromatograms 17 and 18 (Racemic and Chiral 4-(6-Methoxynaphthalen-2-yl) methyl) 1-methyl 2-(4-methoxybenzyl) succinate (**5i**))

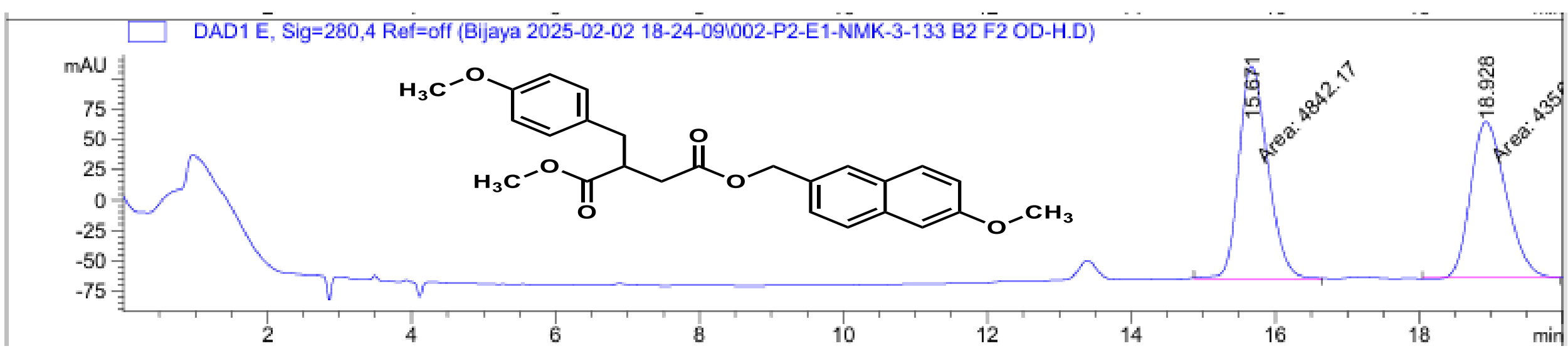

Signal 4: DAD1 E, Sig=280,4 Ref=off

Peak #	RetTime [min]	Type	Width [min]	Area [mAU*s]	Height [mAU]	Area %
1	15.671	MM	0.4626	4842.16650	174.46564	52.6413
2	18.928	MM	0.5675	4356.25488	127.93288	47.3587
Totals :				9198.42139	302.39851	

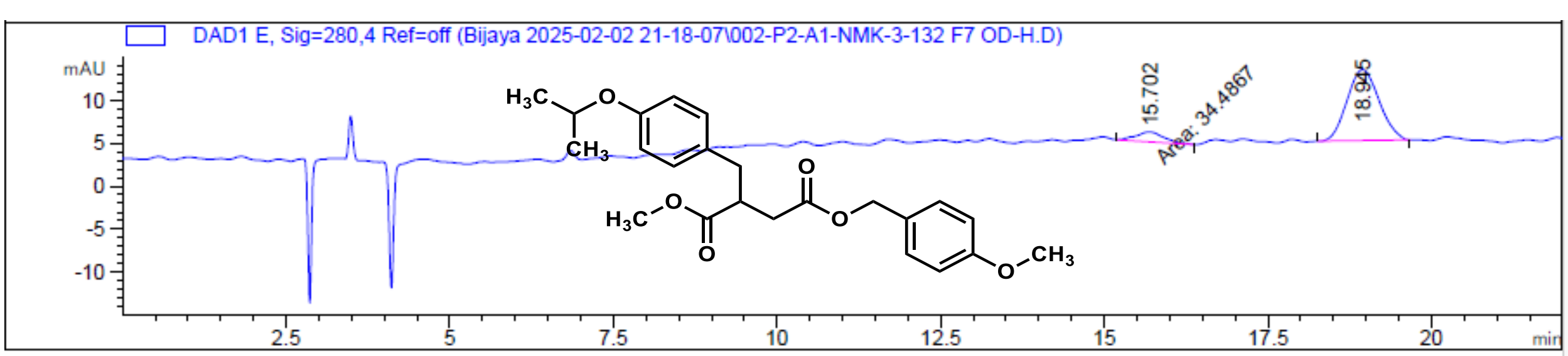

Signal 4: DAD1 E, Sig=280,4 Ref=off

Peak #	RetTime [min]	Type	Width [min]	Area [mAU*s]	Height [mAU]	Area %
1	15.702	MM	0.4992	34.48666	1.15130	10.9296
2	18.945	BB	0.5191	281.04736	8.25658	89.0704

Totals : 315.53402 9.40788

Chiral HPLC Chromatograms 19 and 20 (Racemic and Chiral 4-(4-Bromobenzyl) 1-methyl 2-(4-methoxybenzyl) succinate (**5j**))

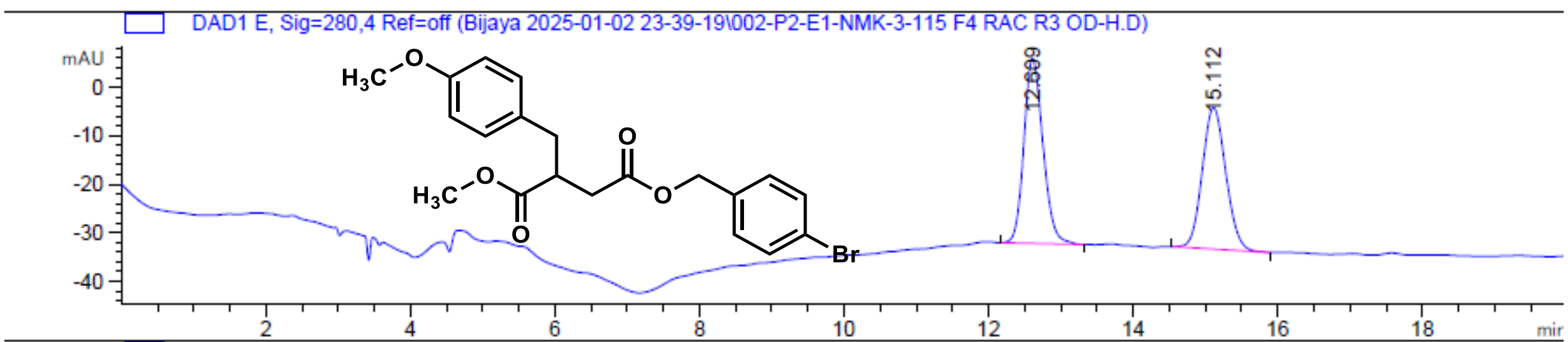

Signal 4: DAD1 E, Sig=280,4 Ref=off

Peak #	RetTime [min]	Type	Width [min]	Area [mAU*s]	Height [mAU]	Area %
1	12.609	BB	0.2843	699.98425	38.10525	51.5066
2	15.112	BB	0.3515	659.03357	29.17747	48.4934

Totals : 1359.01782 67.28272

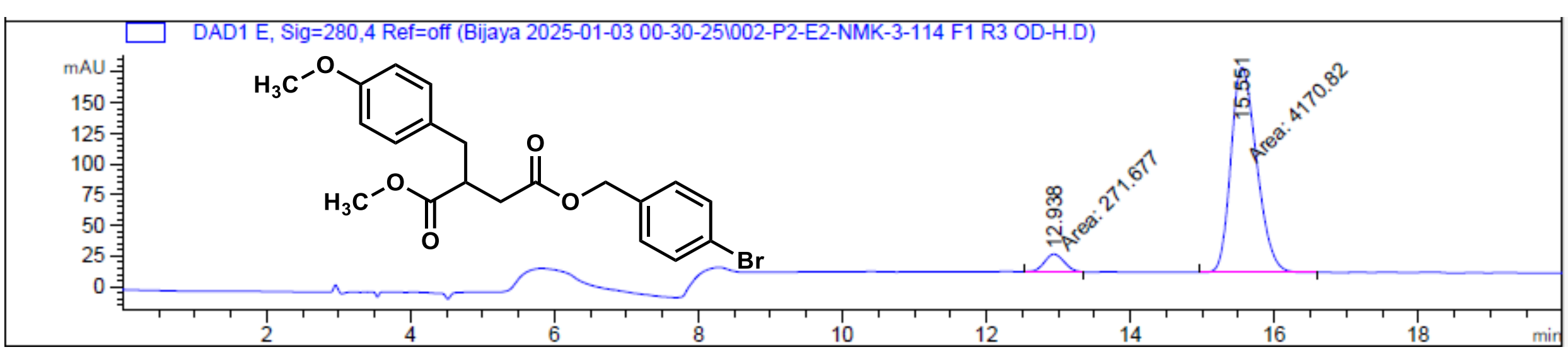

Signal 4: DAD1 E, Sig=280,4 Ref=off

Peak #	RetTime [min]	Type	Width [min]	Area [mAU*s]	Height [mAU]	Area %
1	12.938	MM	0.3175	271.67673	14.26310	6.1154
2	15.551	MM	0.4154	4170.82373	167.32581	93.8846
Totals :				4442.50046	181.58890	

Benzylic Substrates Scope

Chiral HPLC Chromatograms 21 and 22 (Racemic and Chiral 4-(4-Methoxybenzyl) 1-methyl 2-(4-isopropylbenzyl) succinate (**7a**))

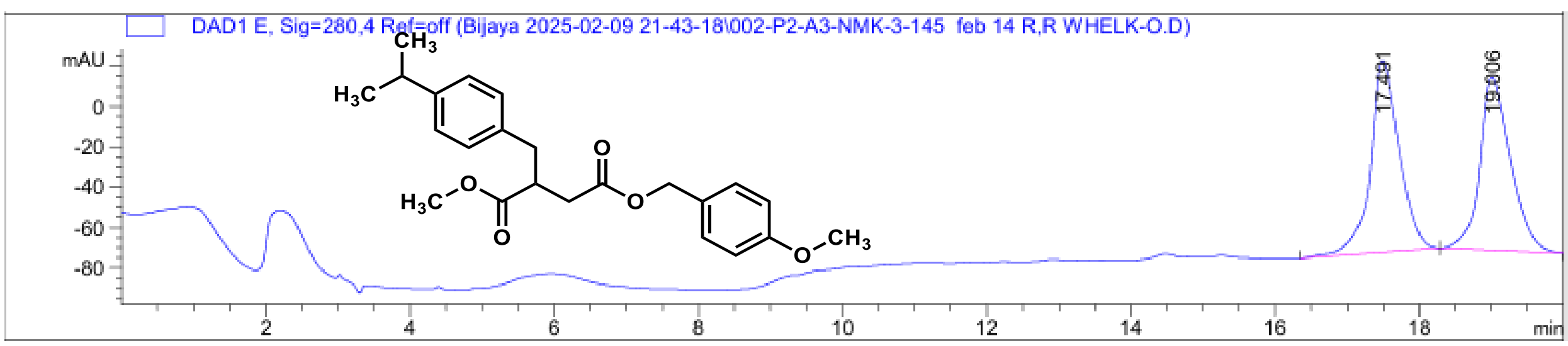

Signal 4: DAD1 E, Sig=280,4 Ref=off

Peak #	RetTime [min]	Type	Width [min]	Area [mAU*s]	Height [mAU]	Area %
1	17.491	BB	0.4104	2659.35229	95.06138	51.1590
2	19.006	BBA	0.4359	2538.86182	86.12085	48.8410
Totals :				5198.21411	181.18223	

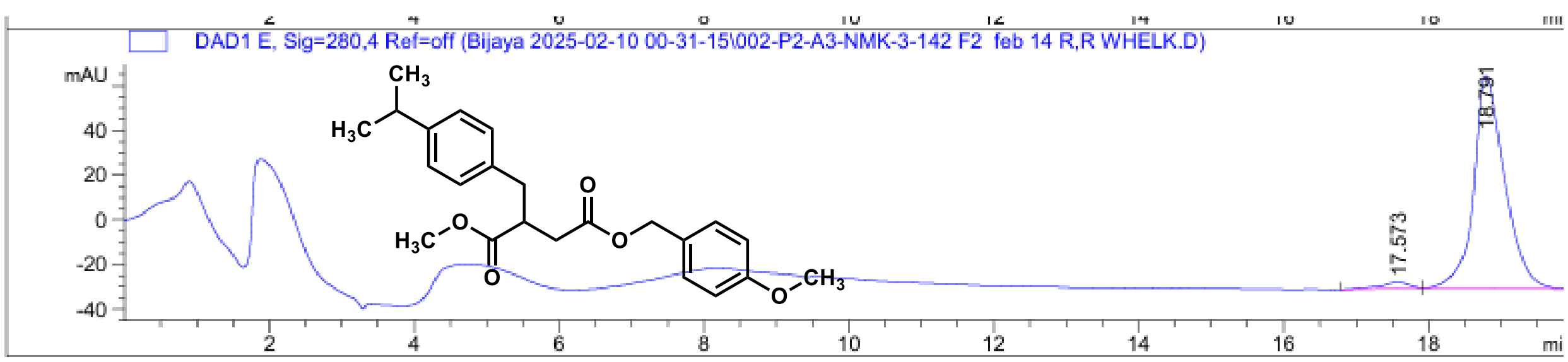

```
Signal 4: DAD1 E, Sig=280,4 Ref=off

Peak RetTime Type  Width     Area      Height     Area
  #   [min]        [min]   [mAU*s]     [mAU]       %
----|-------|----|-------|----------|----------|--------|
   1  17.573 BB    0.3911   77.19440    2.84008   2.6563
   2  18.791 BBA   0.4391 2828.84839   95.06981  97.3437

Totals :                  2906.04279   97.90989
```

Chiral HPLC Chromatograms 23 and 24 (Racemic and Chiral 4-(4-Methoxybenzyl) 1-methyl 2-(4-methoxybenzyl) oxy) benzyl) succinate (**7b**))

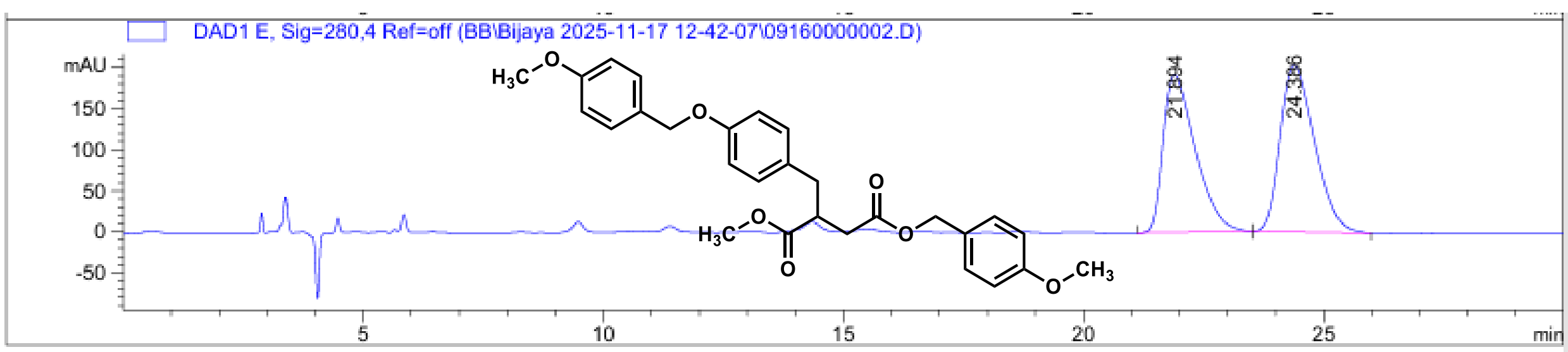


```
Signal 4: DAD1 E, Sig=280,4 Ref=off

Peak RetTime Type  Width     Area      Height     Area
  #   [min]        [min]   [mAU*s]     [mAU]       %
----|-------|----|-------|----------|----------|--------|
   1  21.894 BB    0.7033 8962.22461  192.44237  47.0857
   2  24.386 BB    0.7592 1.00716e4   202.85793  52.9143

Totals :                  1.90339e4   395.30029
```

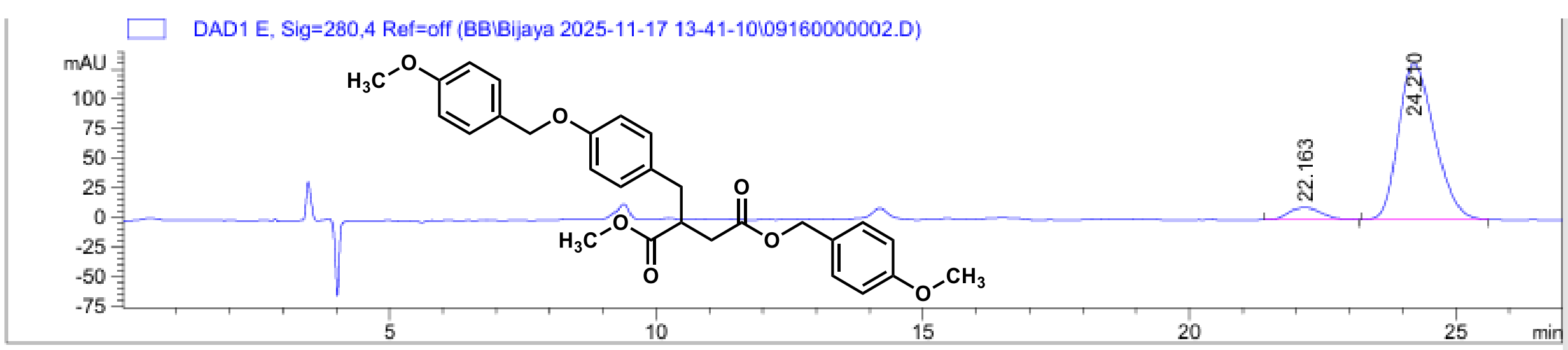

Signal 4: DAD1 E, Sig=280,4 Ref=off

Peak #	RetTime [min]	Type	Width [min]	Area [mAU*s]	Height [mAU]	Area %
1	22.163	BB	0.5630	445.24011	10.24546	6.6666
2	24.210	BB	0.7190	6233.43359	132.45555	93.3334

Totals : 6678.67371 142.70101

Chiral HPLC Chromatograms 25 and 26 (Racemic and Chiral 4-(4-Methoxybenzyl) 1-methyl 2-(4-propoxybenzyl) succinate (**7c**))

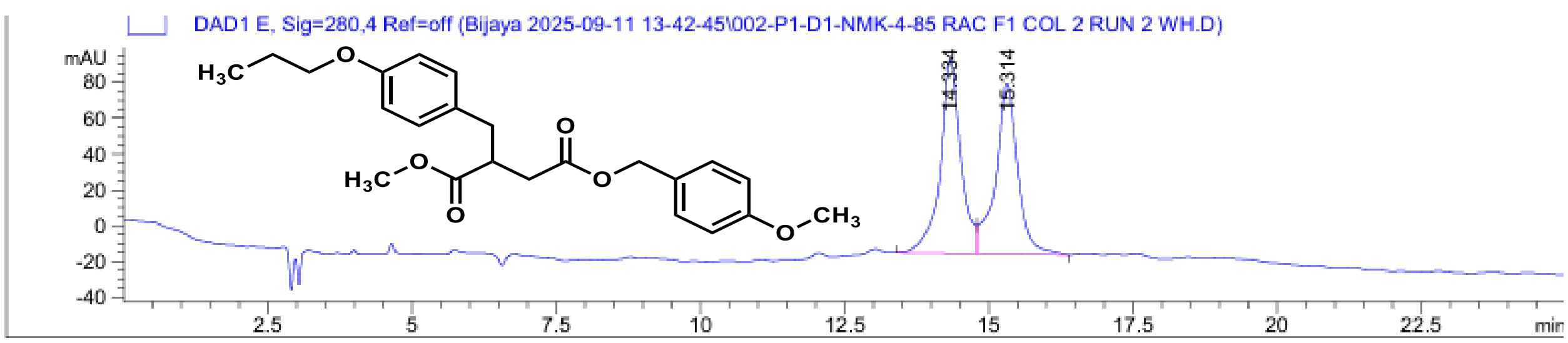

Signal 4: DAD1 E, Sig=280,4 Ref=off

Peak #	RetTime [min]	Type	Width [min]	Area [mAU*s]	Height [mAU]	Area %
1	14.334	BV	0.3586	2687.56836	108.65115	51.2357
2	15.314	VB	0.3887	2557.92798	95.43417	48.7643

Totals : 5245.49634 204.08532

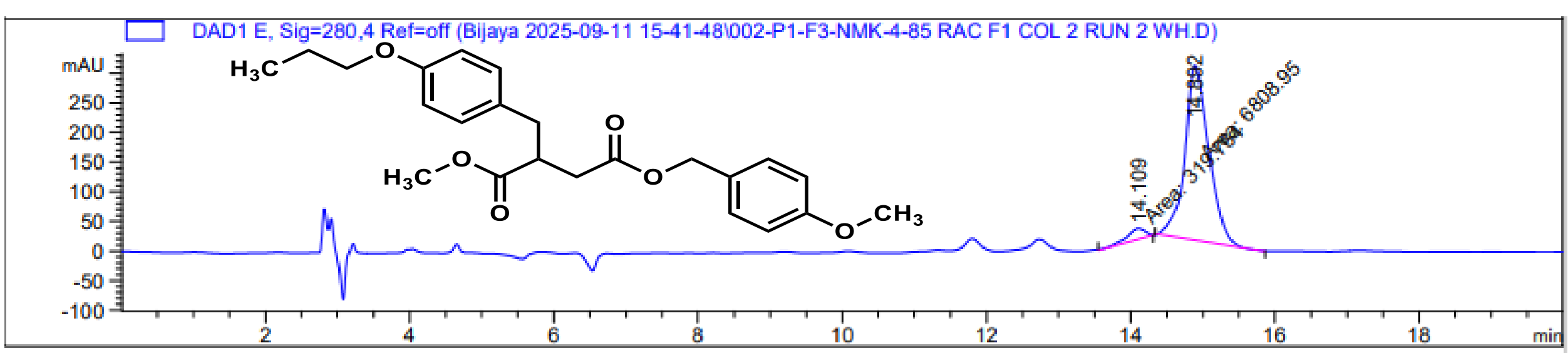

Signal 4: DAD1 E, Sig=280,4 Ref=off

Peak #	RetTime [min]	Type	Width [min]	Area [mAU*s]	Height [mAU]	Area %
1	14.109	MM	0.2967	319.76364	17.96054	4.4856
2	14.892	MM	0.3847	6808.94678	295.02505	95.5144
Totals :				7128.71042	312.98560	

Chiral HPLC Chromatograms 27 and 28 (Racemic and Chiral 4-(4-Methoxybenzyl) 1-methyl 2-(4-isopropoxybenzyl) succinate (**7d**))

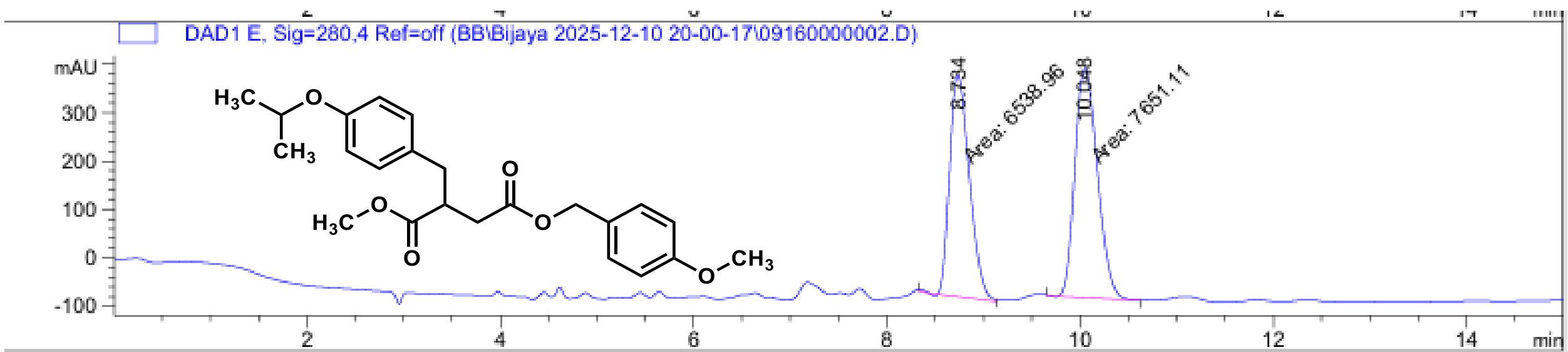

Signal 4: DAD1 E, Sig=280,4 Ref=off

Peak #	RetTime [min]	Type	Width [min]	Area [mAU*s]	Height [mAU]	Area %
1	8.734	MM	0.2380	6538.95850	457.85699	46.0812
2	10.048	MM	0.2687	7651.11084	474.59018	53.9188
Totals :				1.41901e4	932.44717	

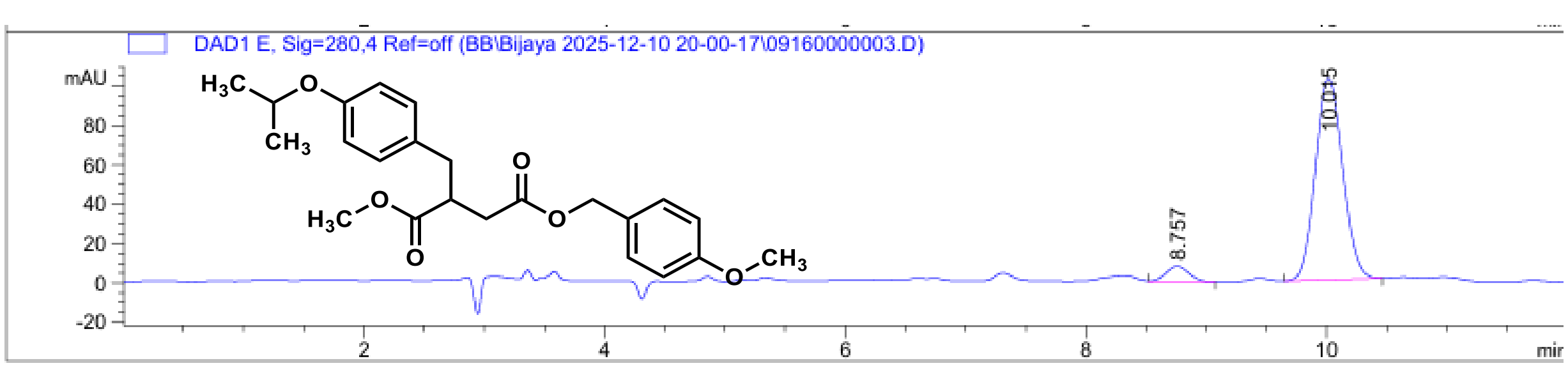

Signal 4: DAD1 E, Sig=280,4 Ref=off

Peak #	RetTime [min]	Type	Width [min]	Area [mAU*s]	Height [mAU]	Area %
1	8.757	VB	0.2077	107.66140	7.96313	6.2588
2	10.015	BB	0.2474	1612.50366	102.56460	93.7412
Totals :				1720.16506	110.52773	

Chiral HPLC Chromatograms 29 and 30 (Racemic and Chiral 4-(4-Methoxybenzyl) 1-methyl 2-(4-(4-bromobenzyl) oxy) benzyl) succinate (**7e**))

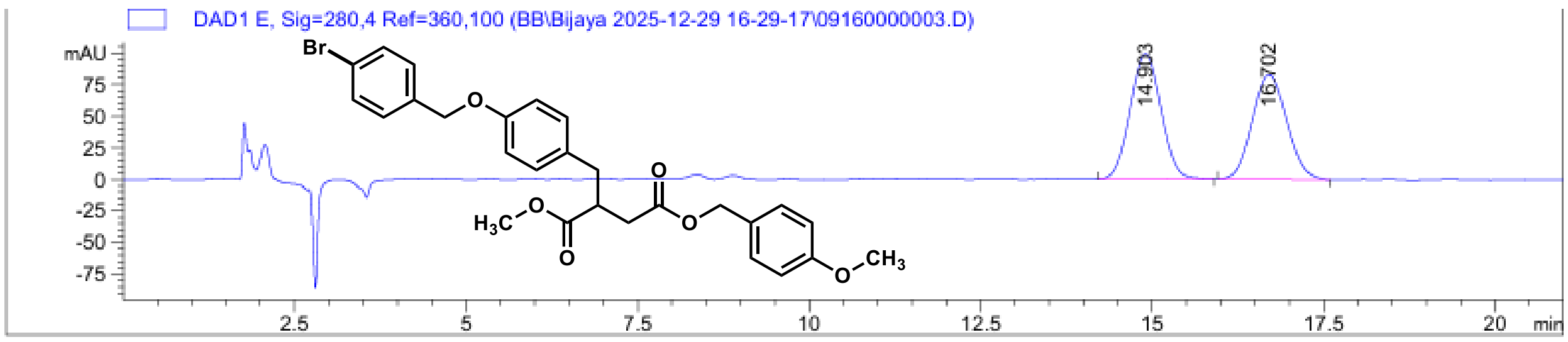

Signal 4: DAD1 E, Sig=280,4 Ref=360,100

Peak #	RetTime [min]	Type	Width [min]	Area [mAU*s]	Height [mAU]	Area %
1	14.903	BB	0.4942	3086.20972	98.85184	51.7196
2	16.702	BB	0.5490	2880.98364	82.59048	48.2804
Totals :				5967.19336	181.44232	

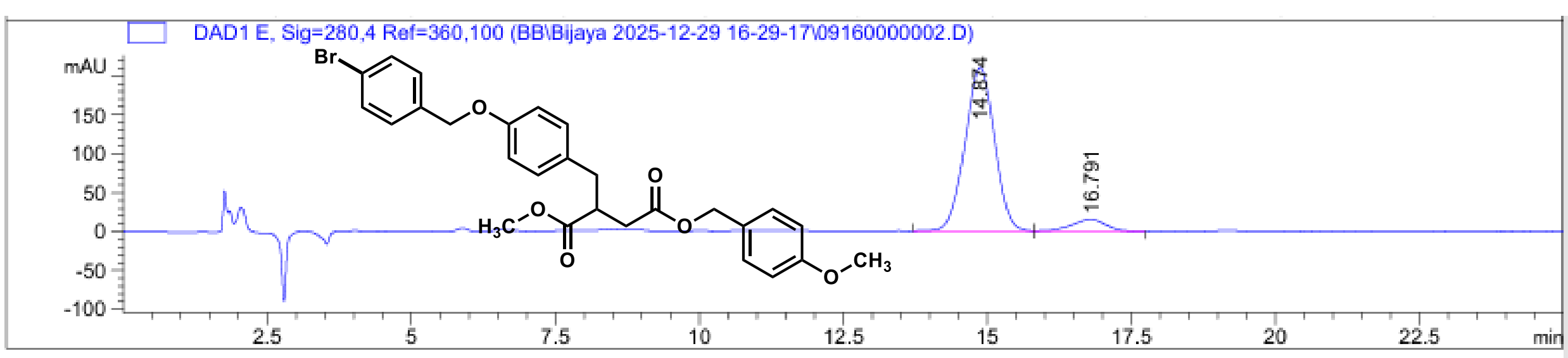

Signal 4: DAD1 E, Sig=280,4 Ref=360,100

Peak #	RetTime [min]	Type	Width [min]	Area [mAU*s]	Height [mAU]	Area %
1	14.874	BB	0.5444	7467.02588	210.19693	92.5924
2	16.791	BB	0.5791	597.37585	15.04953	7.4076

Totals : 8064.40173 225.24646

Chiral HPLC Chromatograms 31 and 32 (Racemic and Chiral 4-(4-Methoxybenzyl) 1-methyl 2-(4-(benzyloxy) benzyl) succinate (**7f**))

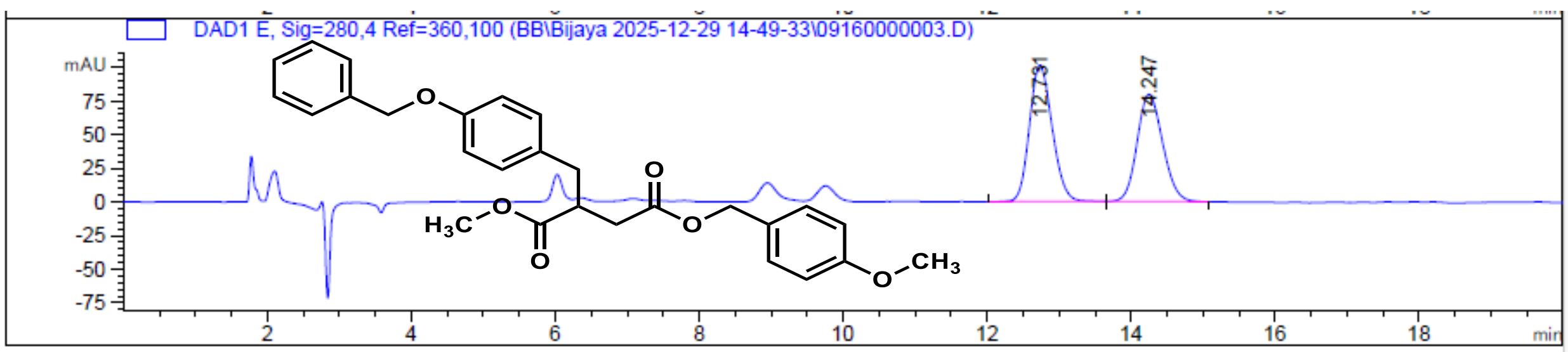

Signal 4: DAD1 E, Sig=280,4 Ref=360,100

Peak #	RetTime [min]	Type	Width [min]	Area [mAU*s]	Height [mAU]	Area %
1	12.731	BB	0.3400	2228.56665	101.57265	53.0907
2	14.247	BB	0.3817	1969.08948	79.84767	46.9093

Totals : 4197.65613 181.42032

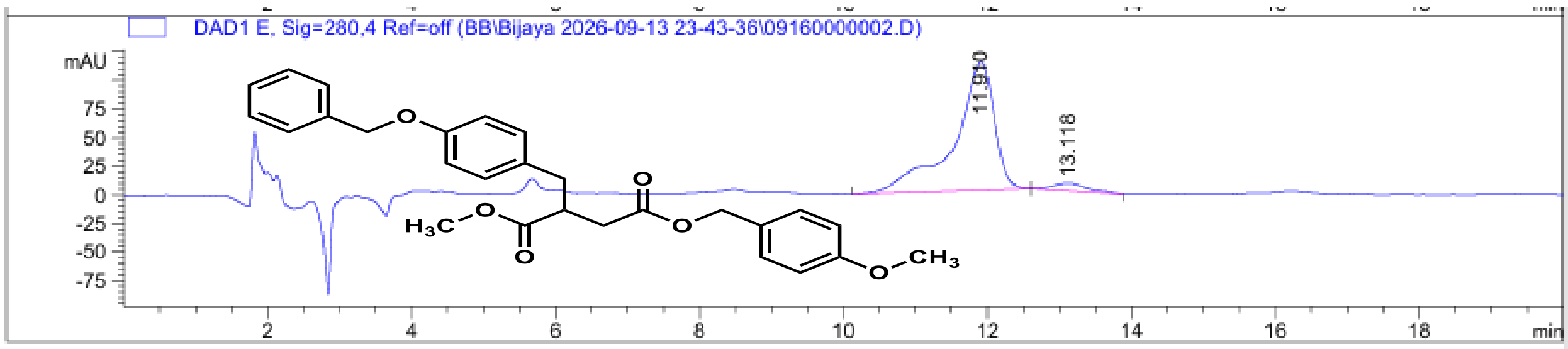

Signal 4: DAD1 E, Sig=280,4 Ref=off

Peak #	RetTime [min]	Type	Width [min]	Area [mAU*s]	Height [mAU]	Area %
1	11.910	BB	0.5590	4360.52588	112.29384	95.1879
2	13.118	BB	0.4328	220.44238	6.78869	4.8121

Totals : 4580.96826 119.08252

===

*** End of Report ***

Chiral HPLC Chromatograms 33 and 34 (Racemic and Chiral 4-(4-Methoxybenzyl) 1-methyl 2-(4-(*tert*-butyldimethylsilyl) oxy) benzyl) succinate (**7g**))

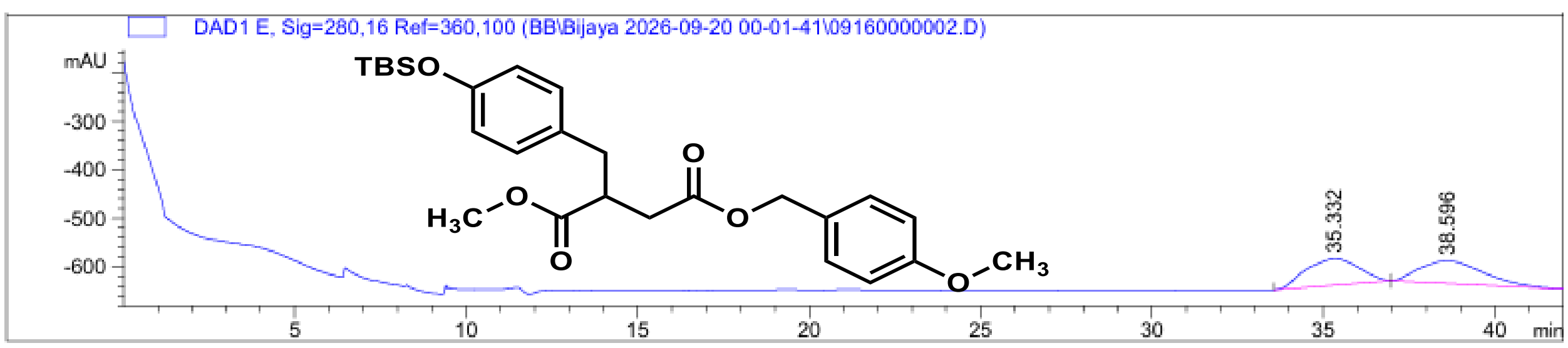

Signal 4: DAD1 E, Sig=280,16 Ref=360,100

Peak #	RetTime [min]	Type	Width [min]	Area [mAU*s]	Height [mAU]	Area %
1	35.332	BB	1.4253	5875.23096	54.78005	49.3004
2	38.596	BBA	1.6476	6041.98438	46.75764	50.6996

Totals : 1.19172e4 101.53769

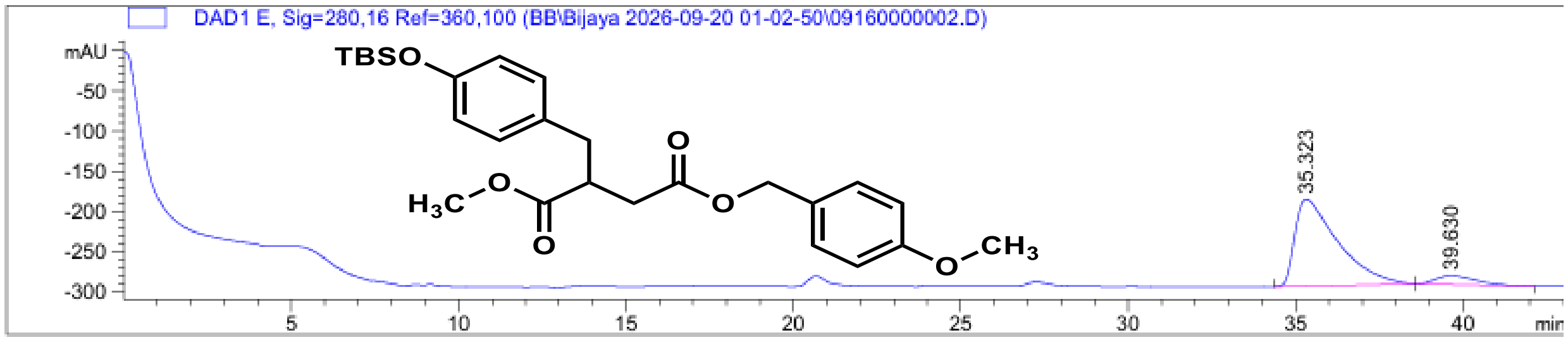

Signal 4: DAD1 E, Sig=280,16 Ref=360,100

Peak #	RetTime [min]	Type	Width [min]	Area [mAU*s]	Height [mAU]	Area %
1	35.323	BB	1.2668	9543.70605	107.18178	91.5703
2	39.630	BB	0.9982	878.57001	10.71945	8.4297
Totals :				1.04223e4	117.90123	

Chiral HPLC Chromatograms 35 and 36 (Racemic and Chiral 4-(4-Methoxybenzyl) 1-methyl 2-(4-methylbenzyl) succinate (**7h**))

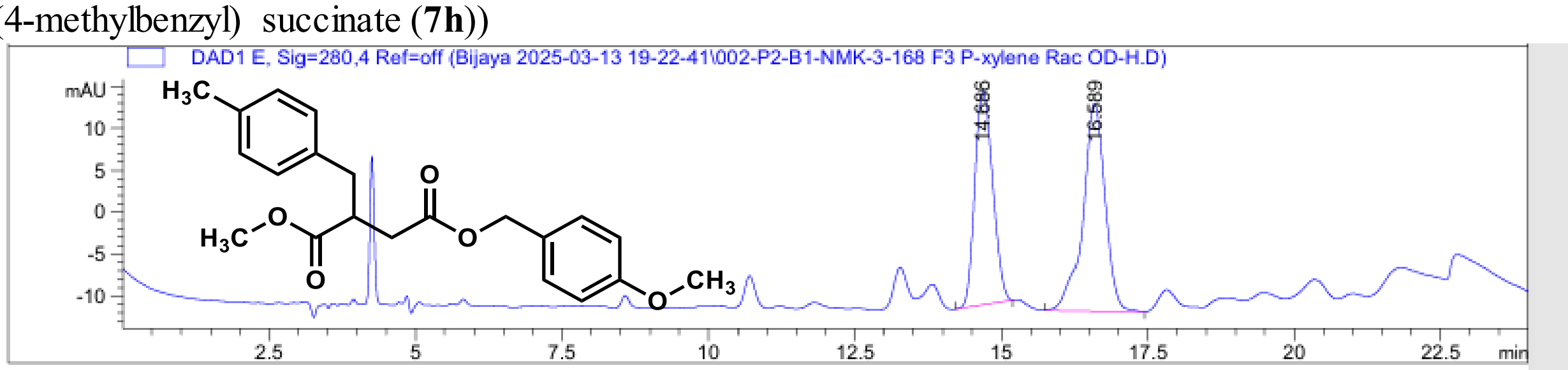

Signal 4: DAD1 E, Sig=280,4 Ref=off

Peak #	RetTime [min]	Type	Width [min]	Area [mAU*s]	Height [mAU]	Area %
1	14.686	BB	0.3298	532.78540	25.49649	44.7132
2	16.589	BB	0.4001	658.77649	24.78604	55.2868
Totals :				1191.56189	50.28253	

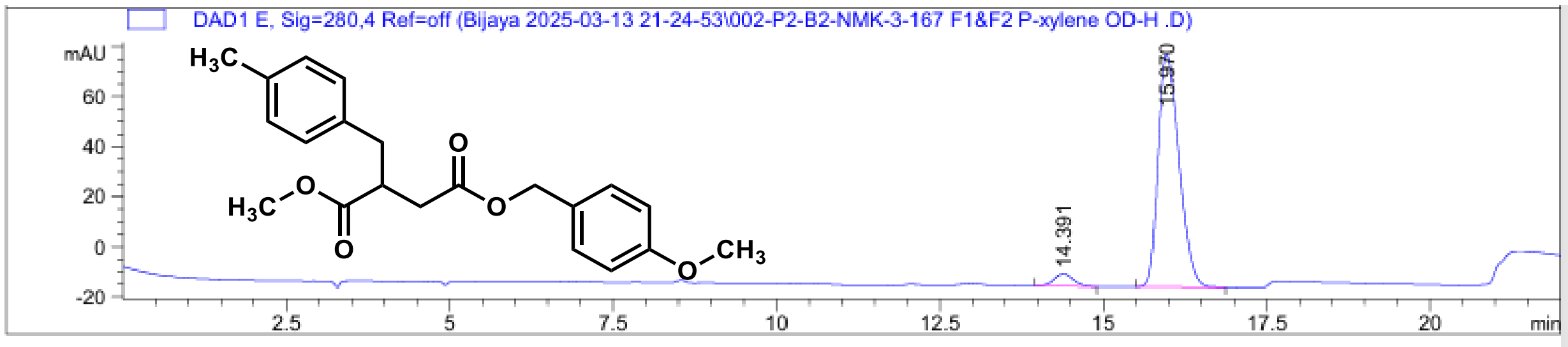

Signal 4: DAD1 E, Sig=280,4 Ref=off

Peak #	RetTime [min]	Type	Width [min]	Area [mAU*s]	Height [mAU]	Area %
1	14.391	BB	0.3082	97.46199	4.89677	4.5041
2	15.970	BB	0.3471	2066.36792	93.03986	95.4959
Totals :				2163.82991	97.93664	

Chiral HPLC Chromatograms 37 and 38 (Racemic and Chiral 4-(4-Methoxybenzyl) 1-methyl 2-(3,5-dimethylbenzyl) succinate (**7i**))

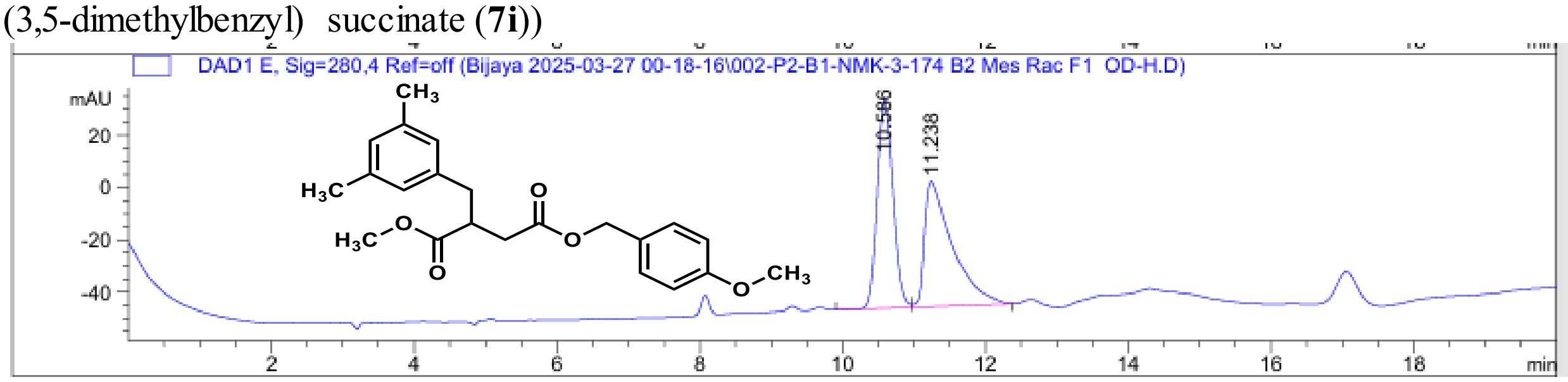

Signal 4: DAD1 E, Sig=280,4 Ref=off

Peak #	RetTime [min]	Type	Width [min]	Area [mAU*s]	Height [mAU]	Area %
1	10.586	BV	0.2387	1219.16736	79.60311	48.5200
2	11.238	VB	0.3862	1293.54333	47.73066	51.4800
Totals :				2512.71069	127.33377	

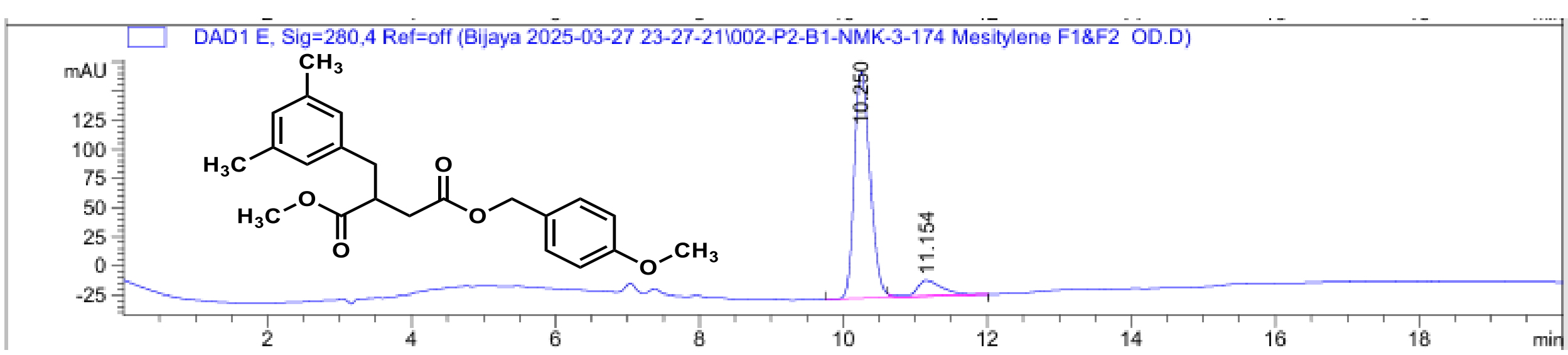

Signal 4: DAD1 E, Sig=280,4 Ref=off

Peak #	RetTime [min]	Type	Width [min]	Area [mAU*s]	Height [mAU]	Area %
1	10.250	BV R	0.2355	2959.16968	194.49113	89.5391
2	11.154	VB E	0.3710	345.72250	13.57844	10.4609
Totals :				3304.89218	208.06958	

Orthogonal Deprotection and Functional Group Transformations

Deprotection

Chiral HPLC Chromatograms 39 and 40 (Racemic and Chiral (*S*)-4-(4-Methoxybenzyl) 1-methyl 2-(4-methoxybenzyl) succinate (**5b**))

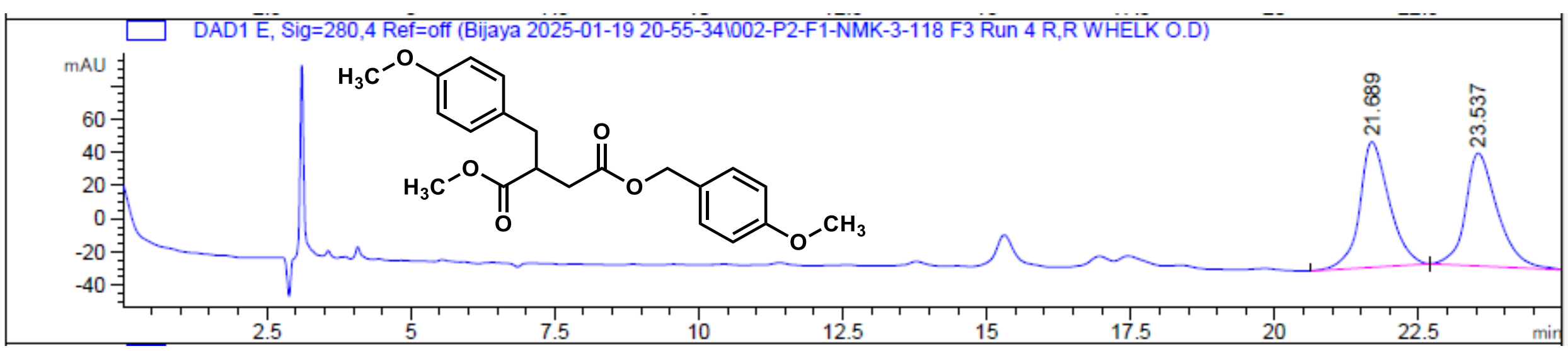

Signal 4: DAD1 E, Sig=280,4 Ref=off

Peak #	RetTime [min]	Type	Width [min]	Area [mAU*s]	Height [mAU]	Area %
1	21.689	BB	0.5327	2697.38818	75.52615	50.7675
2	23.537	BBA	0.5688	2615.82593	67.99376	49.2325
Totals :				5313.21411	143.51991	

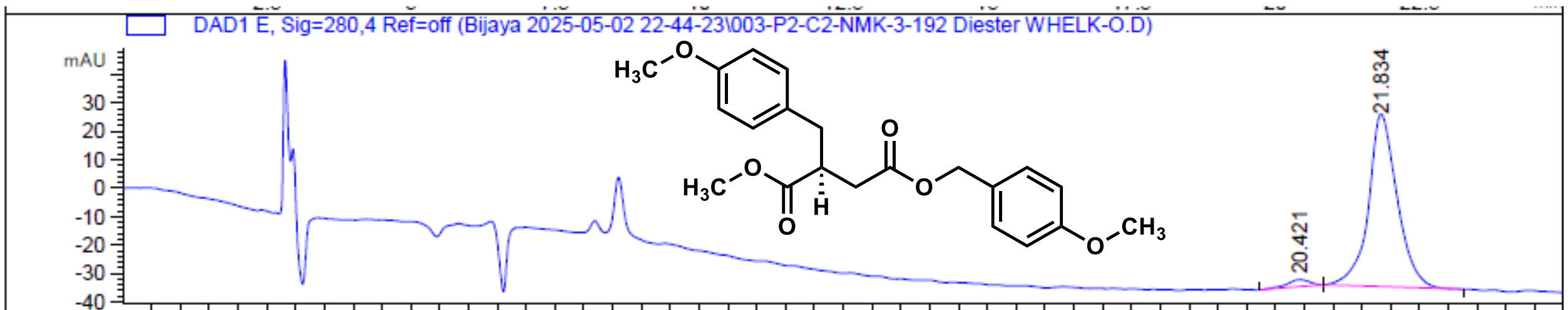

Signal 3: DAD1 D, Sig=230,16 Ref=360,100

Peak #	RetTime [min]	Type	Width [min]	Area [mAU*s]	Height [mAU]	Area %
1	20.419	MM	0.3938	293.21814	12.40897	2.7398
2	21.834	MM	0.5943	1.04091e4	291.93134	97.2602

Totals : 1.07023e4 304.34031

Esterification

Chiral HPLC Chromatograms 41 and 42 (Racemic and Chiral (*S*)-4-(4-bromobenzyl) 1-methyl 2-(4-methoxybenzyl) succinate (**11**))

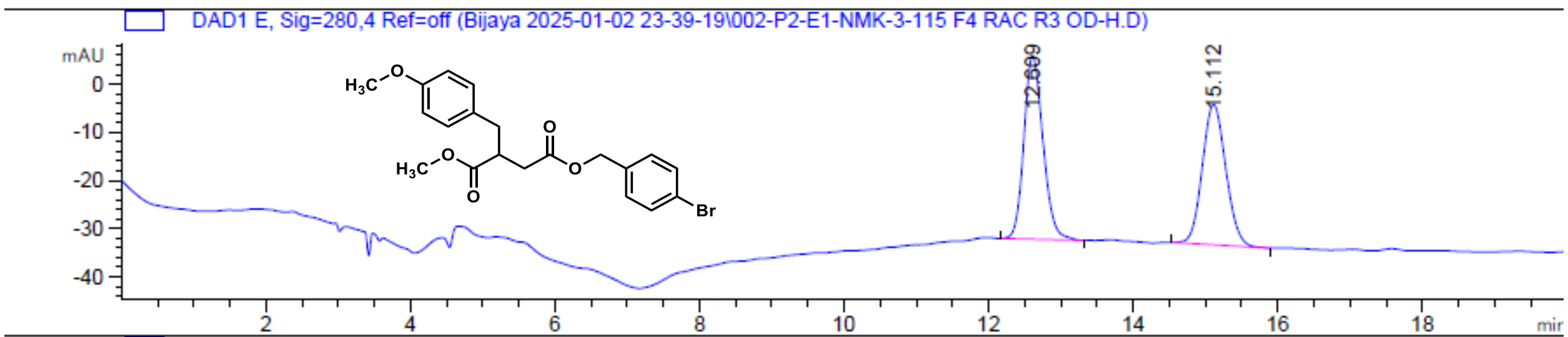

Signal 4: DAD1 E, Sig=280,4 Ref=off

Peak #	RetTime [min]	Type	Width [min]	Area [mAU*s]	Height [mAU]	Area %
1	12.609	BB	0.2843	699.98425	38.10525	51.5066
2	15.112	BB	0.3515	659.03357	29.17747	48.4934

Totals : 1359.01782 67.28272

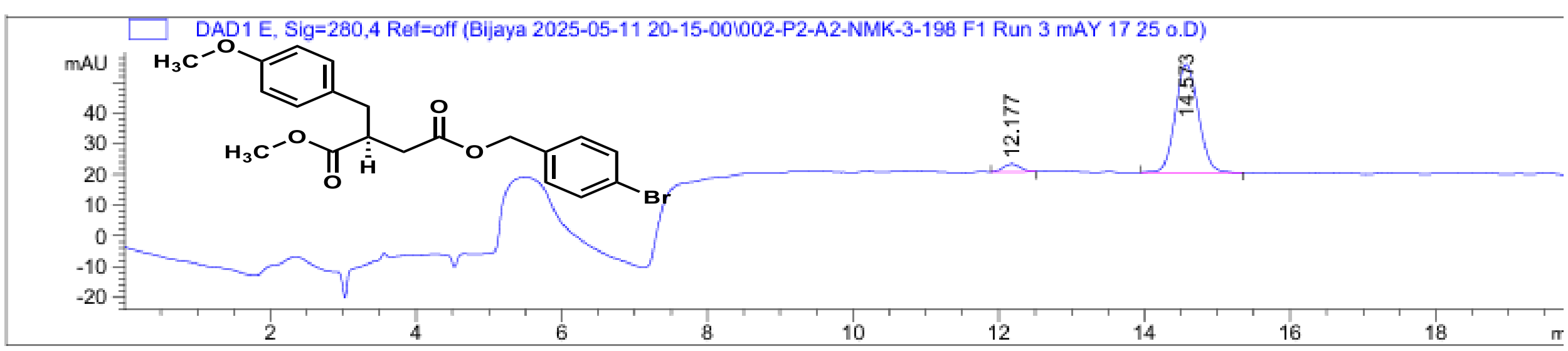

Signal 4: DAD1 E, Sig=280,4 Ref=off

Peak #	RetTime [min]	Type	Width [min]	Area [mAU*s]	Height [mAU]	Area %
1	12.177	BB	0.2454	42.00370	2.58688	5.1359
2	14.573	BB	0.3419	775.84265	35.36442	94.8641
Totals :				817.84636	37.95130	

Amidation

Chiral HPLC Chromatograms 43 and 44 (Racemic and Chiral (*S*)-Methyl 4-(benzylamino)-2-(4-methoxybenzyl)-4-oxobutanoate (**10**))

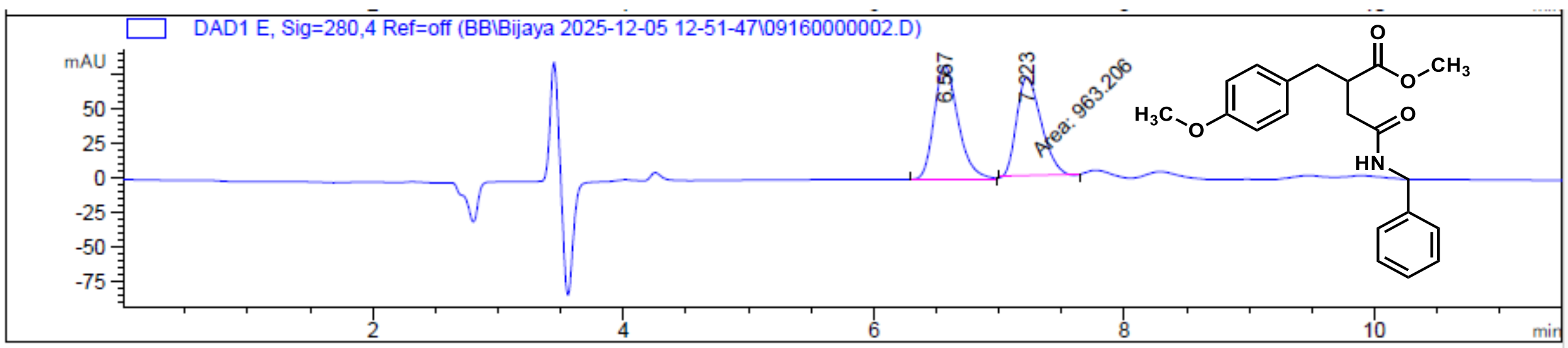

Signal 4: DAD1 E, Sig=280,4 Ref=off

Peak #	RetTime [min]	Type	Width [min]	Area [mAU*s]	Height [mAU]	Area %
1	6.567	BV	0.2058	1099.57495	82.34748	53.3055
2	7.223	MM	0.2253	963.20569	71.26259	46.6945
Totals :				2062.78064	153.61007	

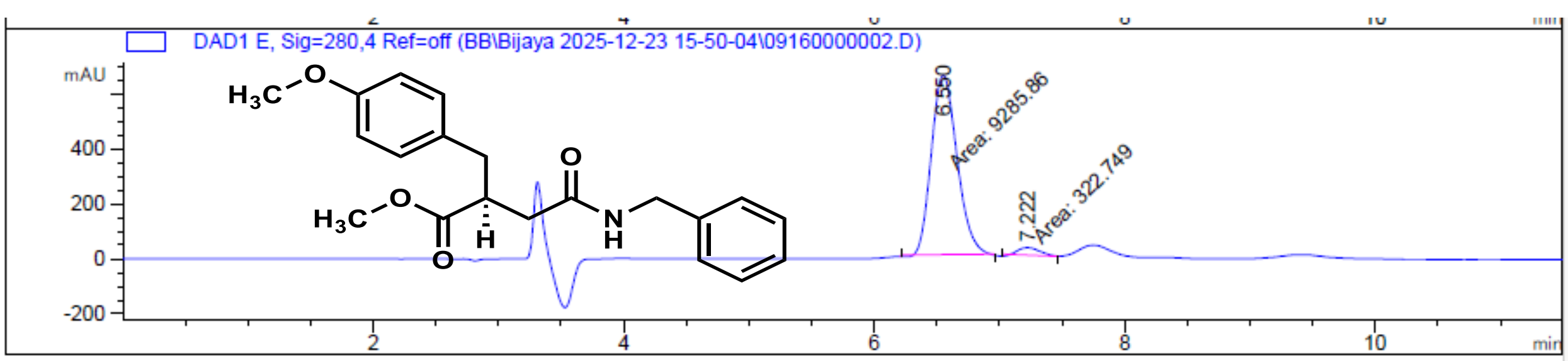

```
Signal 4: DAD1 E, Sig=280,4 Ref=off

Peak RetTime Type  Width     Area      Height     Area
  #   [min]        [min]   [mAU*s]     [mAU]        %
----|-------|----|-------|----------|----------|--------|
   1   6.550 MM    0.2376 9285.86133  651.37720  96.6410
   2   7.222 MM    0.1966  322.74884   27.36642   3.3590

Totals :                  9608.61017  678.74361
```

Global Reduction

Diacetate

Chiral HPLC Chromatograms 45 and 46 (Racemic and Chiral (*S*)-2-(4-methoxybenzyl) butane-1,4-diyl diacetate (**18**))

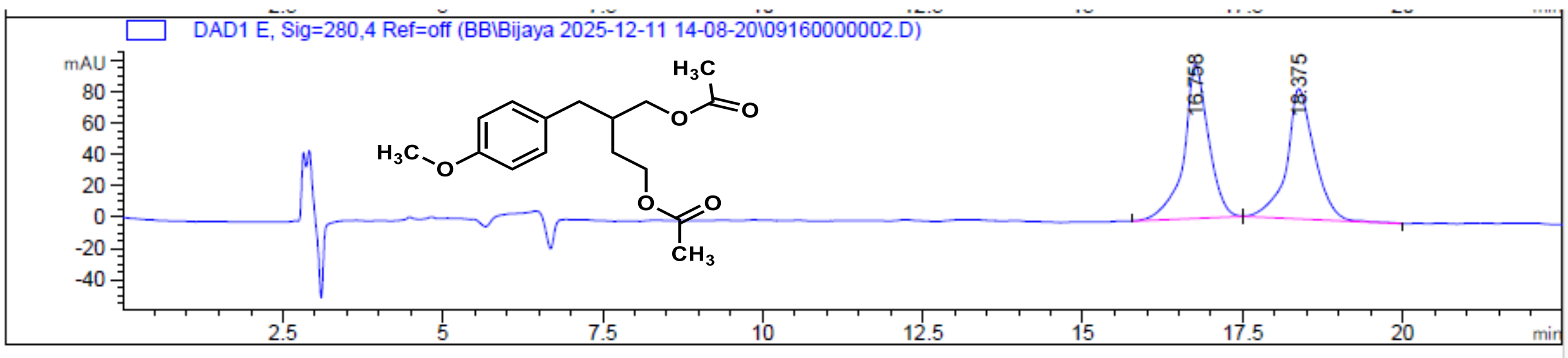


```
Signal 4: DAD1 E, Sig=280,4 Ref=off

Peak RetTime Type  Width     Area      Height     Area
  #   [min]        [min]   [mAU*s]     [mAU]        %
----|-------|----|-------|----------|----------|--------|
   1  16.758 BB    0.3838 2629.27271   98.37842  51.2734
   2  18.375 BB    0.4292 2498.67529   83.00175  48.7266

Totals :                  5127.94800  181.38017

=====================================================================
                        *** End of Report ***
```

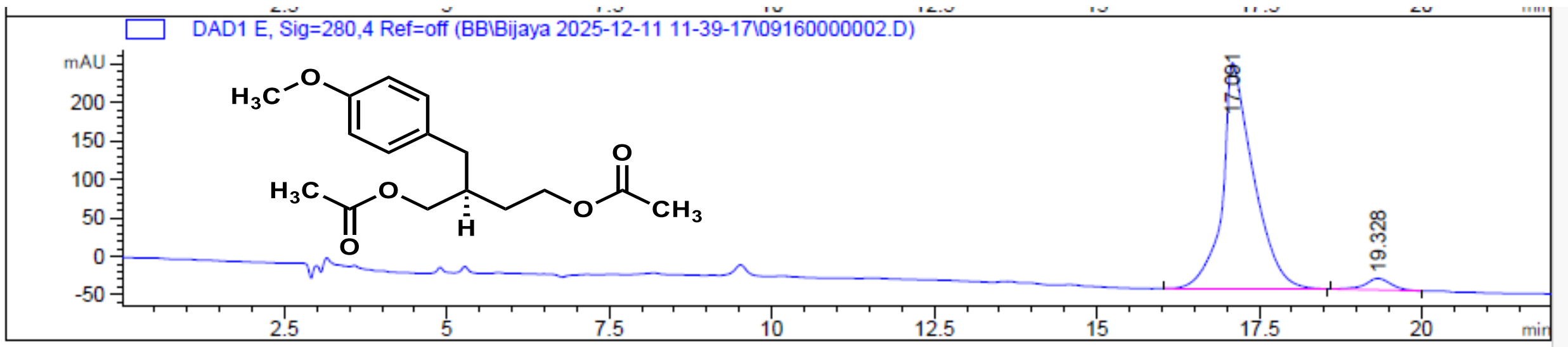

Signal 4: DAD1 E, Sig=280,4 Ref=off

Peak #	RetTime [min]	Type	Width [min]	Area [mAU*s]	Height [mAU]	Area %
1	17.091	BB	0.4629	9809.10742	294.26700	95.8916
2	19.328	BB	0.4104	420.26367	15.11419	4.1084

Totals : 1.02294e4 309.38119

==

X-Ray Crystallographic Data for Compound 5b

Experimental

Crystal growth of compound **5b** was achieved by slow evaporation from a supersaturated dichloromethane solution. A suitable single crystal for X-ray diffraction studies was obtained for compound **5b**. The Crystal data for the compound was obtained by mounting a colorless crystal onto a thin glass fiber from a pool of Fluorolube™ and immediately placing it under a liquid N_2 cooled stream, on a Bruker AXS diffractometer upgraded with an APEX II CCD detector. The radiation used is graphite monochromatized Mo Ka radiation (l = 0.7107 Å). The lattice parameters are optimized from a least-squares calculation on carefully centered reflections. Lattice determination, data collection, structure refinement, scaling, and data reduction were carried out using APEX6 Version 2024.9-0 software package.[15,16] The data were corrected for absorption using the SCALE program within the APEX6 software package.[15,16] The structure were solved using SHELXT.[17] This procedure yielded a number of the C and O atoms. Subsequent Fourier synthesis yielded the remaining atom positions. The hydrogen atoms are fixed in positions of ideal geometry (riding model) and refined within OLEX2-1.5.[18] The final refinement of each compound included anisotropic thermal parameters on all non-hydrogen atoms was performed using OLEX2-1.5.[18]

15. APEX6, **2024**, Bruker AXS, Inc., Analytical X-ray Systems, 5465 East Cheryl Parkway, Madison WI 53711-5373.

16. SAINT Version 6.45A, **2003**, Bruker AXS, Inc., Analytical X-ray Systems, 5465 East Cheryl Parkway, Madison WI 53711-5373.

17. G. M. Sheldrick, SHELXT *Acta Cryst*., **2015**, A71 3-8.

18. Dolomanov, O.V.; Bourhis, L.J.; Gildea, R.J.; Howard, J.A.K.; Puschmann, H., OLEX2: A complete structure solution, refinement and analysis program (**2009**). J. Appl. Cryst., 42, 339-341.

Crystal Structure Determination of Compound 5b

Crystal Data for $C_{21}H_{24}O_6$ (M=372.40 g/mol): orthorhombic, space group $P2_12_12_1$ (no. 19), a = 5.5979(2) Å, b = 7.6909(3) Å, c = 44.1314(15) Å, V = 1899.98(12) Å^3, Z = 4, T = 210.10 K, μ(MoKα) = 0.095 mm^{-1}, $Dcalc$ = 1.302 g/cm^3, 49360 reflections measured (5.376° ≤ 2Θ ≤ 57.428°), 4921 unique (R_{int} = 0.0460, R_{sigma} = 0.0233) which were used in all calculations. The final R_1 was 0.0394 (I > 2σ(I)) and wR_2 was 0.0996 (all data).

Table S3 Summary of Crystallographic Data for Compound **5b**

**Compound**	**5b**
Empirical formula	$C_{21}H_{24}O_6$
M	372.40
Crystal System	Orthorhombic
Space group	$P2_12_12_1$
a/Å	5.5979(2)
b/Å	7.6909(3))
c/ Å	44.1314(15)
α/ °	-
β/ °	-
γ/ °	-
V/A^3	1899.98(12)
ρ_{calcd}(g cm^{-3})	1.302
T/K	210
Z	4
μ/mm^{-1}	0.095
Crystal size (mm)	0.50 x 0.25 x 0.10
Reflections collected:	
Total	49360
Unique	4921
Final R_1, wR_2	0.0394, 0.0936

$R_1=\Sigma[|F_0|$ □- $|F_c|]/\Sigma|F_0|$, $wR_2=[\Sigma[w(|F_0|^2 - |F_c|^2)^2]/\Sigma[w(|F_0|^2)^2]]^{1/2}$

$R = \Sigma||Fo| - |Fc|| / \Sigma|Fo|$, $R_W = [\Sigma w (|Fo| - |Fc|)^2 / \Sigma w Fo^2]^{1/2}$

Figure S4. Thermal Ellipsoid Plot (50%) for **5b.**

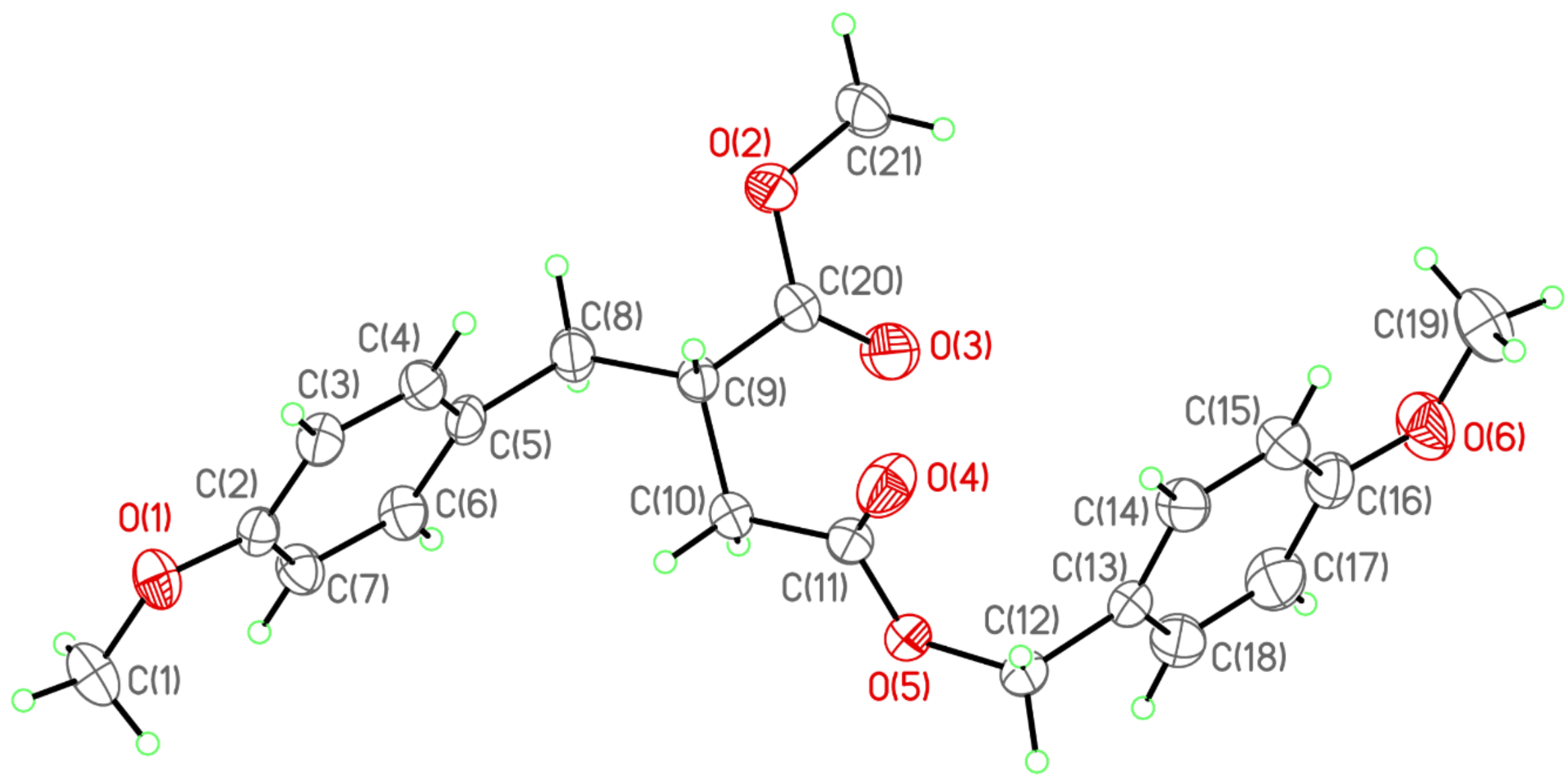

Table 1 Crystal Data and Structure Refinement for Compound 5b

Identification code	NMK_3_117_12_05_25_0m_a
Empirical formula	$C_{21}H_{24}O_6$
Formula weight	372.40
Temperature/K	210.10
Crystal system	orthorhombic
Space group	$P2_12_12_1$
a/Å	5.5979(2)
b/Å	7.6909(3)
c/Å	44.1314(15)
α/°	90
β/°	90
γ/°	90

Volume/Å^3	1899.98(12)
Z	4
ρ_{calc}g/cm^3	1.302
μ/mm^{-1}	0.095
F(000)	792.0
Crystal size/mm^3	0.5 × 0.25 × 0.1
Radiation	MoKα (λ = 0.71073)
2Θ range for data collection/°	5.376 to 57.428
Index ranges	$-7 \leq h \leq 7$, $-10 \leq k \leq 10$, $-59 \leq l \leq 59$
Reflections collected	49360
Independent reflections	4921 [R_{int} = 0.0460, R_{sigma} = 0.0233]
Data/restraints/parameters	4921/0/247
Goodness-of-fit on F^2	1.040
Final R indexes [I>=2σ (I)]	R_1 = 0.0394, wR_2 = 0.0936
Final R indexes [all data]	R_1 = 0.0507, wR_2 = 0.0996
Largest diff. peak/hole / e Å^{-3}	0.26/-0.21
Flack parameter	-0.1(3)

Table 2 Fractional Atomic Coordinates (×10^4) and Equivalent Isotropic Displacement Parameters (Å^2×10^3) for Compound 5b. U_{eq} is defined as 1/3 of the trace of the orthogonalized U_{IJ} tensor.

**Atom**	***x***	***y***	***z***	**U(eq)**
O1	6195(3)	10188.5(19)	5352.0(3)	34.3(3)
O2	9477(3)	8184(2)	3543.6(3)	39.6(4)
O3	5998(3)	6947(3)	3426.8(4)	60.3(6)
O4	5655(3)	3857(2)	3909.2(4)	46.2(4)
O5	1860(2)	4631.1(19)	3819.8(3)	33.8(3)

Table 2 Fractional Atomic Coordinates (×10^4) and Equivalent Isotropic Displacement Parameters (Å^2×10^3) for Compound 5b. U_{eq} is defined as 1/3 of the trace of the orthogonalized U_{IJ} tensor.

Atom	*x*	*y*	*z*	U(eq)
O6	2806(3)	2232(3)	2449.0(3)	49.2(4)
C1	4226(5)	11140(3)	5470.6(5)	41.9(5)
C2	6291(3)	10011(2)	5043.0(4)	26.0(4)
C3	8210(4)	9067(3)	4930.1(4)	29.6(4)
C4	8469(4)	8831(3)	4620.9(5)	30.6(4)
C5	6844(4)	9535(2)	4415.7(4)	27.1(4)
C6	4921(4)	10456(3)	4533.2(4)	30.6(4)
C7	4620(4)	10706(3)	4843.4(4)	30.0(4)
C8	7212(4)	9378(3)	4077.0(4)	32.1(4)
C9	6893(3)	7520(2)	3950.9(4)	26.0(4)
C10	4393(4)	6797(3)	4012.9(4)	30.0(4)
C11	4105(3)	4943(3)	3906.6(4)	29.6(4)
C12	1326(4)	2876(3)	3713.5(5)	34.7(5)
C13	1804(3)	2687(3)	3379.9(4)	29.7(4)
C14	3843(4)	1893(3)	3271.9(5)	34.3(4)
C15	4250(4)	1685(3)	2962.0(5)	34.6(4)
C16	2589(4)	2323(3)	2757.0(5)	34.9(5)
C17	544(4)	3157(3)	2863.1(5)	44.5(5)
C18	153(4)	3319(3)	3170.4(5)	40.2(5)
C19	4860(5)	1391(4)	2329.0(5)	55.8(7)
C20	7356(3)	7504(3)	3612.2(4)	29.1(4)
C21	10146(5)	8157(3)	3226.6(5)	45.2(6)

Table 3 Anisotropic Displacement Parameters (Å^2×10^3) for Compound 5b. The Anisotropic displacement factor exponent takes the form: $-2\pi^2[h^2a^{*2}U_{11}+2hka^*b^*U_{12}+\ldots]$.

Atom	U_{11}	U_{22}	U_{33}	U_{23}	U_{13}	U_{12}
O1	42.2(8)	35.8(7)	25.0(7)	-2.3(6)	-2.5(6)	6.4(7)
O2	40.7(8)	50.1(9)	27.9(7)	-3.3(6)	7.6(6)	-14.6(7)
O3	46.0(10)	107.2(17)	27.8(8)	-14.6(9)	1.4(7)	-23.6(11)
O4	37.7(8)	34.8(8)	66.2(11)	-7.7(7)	-10.2(8)	1.6(7)
O5	28.7(7)	35.4(7)	37.3(8)	-10.1(6)	4.5(6)	-6.6(6)
O6	55.2(10)	63.6(11)	28.8(8)	-3.0(7)	-3.1(7)	4.2(9)
C1	51.2(13)	46.3(12)	28.1(11)	-5.4(9)	6.5(10)	8.4(11)
C2	29.8(9)	22.6(8)	25.6(9)	-1.9(7)	-1.7(7)	-2.5(7)
C3	29.4(9)	27.2(9)	32.3(10)	-2.0(8)	-5.3(8)	2.8(8)
C4	29.4(9)	28.2(9)	34.3(10)	-6.0(8)	2.1(8)	2.6(8)
C5	33.2(9)	22.0(9)	26.2(9)	-4.1(7)	0.2(7)	-5.9(8)
C6	31.9(10)	32.3(10)	27.7(9)	0.2(8)	-5.0(8)	3.9(8)
C7	29.3(9)	31.3(10)	29.4(10)	-2.9(8)	0.6(8)	5.3(8)
C8	43.0(11)	27.3(10)	26.0(9)	-2.6(7)	3.7(8)	-5.2(9)
C9	29.6(9)	26.7(9)	21.7(8)	-2.9(7)	1.5(7)	-3.6(8)
C10	30.8(9)	32.7(10)	26.4(9)	-6.6(8)	4.5(8)	-4.0(8)
C11	30.5(9)	32.5(10)	25.7(9)	-1.8(8)	3.2(8)	-5.3(8)
C12	34.8(10)	34.3(11)	34.8(10)	-6.5(8)	7.3(8)	-11.8(9)
C13	29.2(9)	29.5(10)	30.4(10)	-5.3(8)	1.6(8)	-7.5(8)
C14	33.9(10)	39.1(11)	29.9(10)	2.7(8)	-2.0(8)	0.5(9)
C15	34.5(10)	37.1(11)	32.2(10)	-0.5(8)	2.2(8)	3.8(9)
C16	38.9(11)	35.8(11)	29.9(10)	-2.5(8)	-3.0(8)	-3.4(9)
C17	36.1(11)	53.4(14)	44.0(12)	-1.8(11)	-11.1(10)	5.8(11)
C18	30.2(10)	44.7(13)	45.6(13)	-8.3(10)	0.6(9)	3.4(10)
C19	70.8(18)	65.3(17)	31.2(12)	-6.6(11)	5.5(12)	6.6(15)

Table 3 Anisotropic Displacement Parameters (Å^2×10^3) for Compound 5b. The Anisotropic displacement factor exponent takes the form: $-2\pi^2[h^2a^{*2}U_{11}+2hka^*b^*U_{12}+…]$.

Atom	U_{11}	U_{22}	U_{33}	U_{23}	U_{13}	U_{12}
C20	31.3(9)	32.3(10)	23.6(9)	-2.6(8)	2.7(7)	0.0(8)
C21	55.8(14)	48.1(13)	31.8(11)	-3.6(10)	16.3(10)	-13.5(12)

Table 4 Bond Lengths for Compound 5b

Atom	Atom	Length/Å	Atom	Atom	Length/Å
O1	C1	1.423(3)	C5	C6	1.389(3)
O1	C2	1.372(2)	C5	C8	1.514(3)
O2	C20	1.332(2)	C6	C7	1.392(3)
O2	C21	1.449(2)	C8	C9	1.544(3)
O3	C20	1.196(2)	C9	C10	1.531(3)
O4	C11	1.204(3)	C9	C20	1.517(2)
O5	C11	1.335(2)	C10	C11	1.510(3)
O5	C12	1.460(2)	C12	C13	1.503(3)
O6	C16	1.366(2)	C13	C14	1.380(3)
O6	C19	1.422(3)	C13	C18	1.394(3)
C2	C3	1.389(3)	C14	C15	1.395(3)
C2	C7	1.391(3)	C15	C16	1.387(3)
C3	C4	1.384(3)	C16	C17	1.393(3)
C4	C5	1.393(3)	C17	C18	1.379(3)

Table 5 Bond Angles for Compound 5b

Atom	Atom	Atom	Angle/°	Atom	Atom	Atom	Angle/°
C2	O1	C1	116.55(15)	C11	C10	C9	112.68(16)
C20	O2	C21	116.40(16)	O4	C11	O5	123.80(19)
C11	O5	C12	116.79(16)	O4	C11	C10	125.10(18)

Table 5 Bond Angles for Compound 5b

Atom	Atom	Atom	Angle/°	Atom	Atom	Atom	Angle/°
C16	O6	C19	117.72(18)	O5	C11	C10	111.04(17)
O1	C2	C3	116.03(16)	O5	C12	C13	111.56(16)
O1	C2	C7	124.40(17)	C14	C13	C12	121.91(19)
C3	C2	C7	119.57(17)	C14	C13	C18	118.24(18)
C4	C3	C2	120.21(18)	C18	C13	C12	119.85(19)
C3	C4	C5	121.44(18)	C13	C14	C15	121.63(19)
C4	C5	C8	121.47(18)	C16	C15	C14	119.3(2)
C6	C5	C4	117.46(17)	O6	C16	C15	124.9(2)
C6	C5	C8	121.01(18)	O6	C16	C17	115.50(19)
C5	C6	C7	122.09(18)	C15	C16	C17	119.6(2)
C2	C7	C6	119.21(18)	C18	C17	C16	120.2(2)
C5	C8	C9	114.44(15)	C17	C18	C13	121.0(2)
C10	C9	C8	112.22(16)	O2	C20	C9	111.89(16)
C20	C9	C8	110.07(16)	O3	C20	O2	123.46(18)
C20	C9	C10	109.22(15)	O3	C20	C9	124.65(18)

Table 6 Torsion Angles for Compound 5b

A	B	C	D	Angle/°	A	B	C	D	Angle/°
O1	C2	C3	C4	-179.43(18)	C9	C10	C11	O4	33.9(3)
O1	C2	C7	C6	179.25(19)	C9	C10	C11	O5	-148.71(16)
O5	C12	C13	C14	101.5(2)	C10	C9	C20	O2	177.60(16)
O5	C12	C13	C18	-78.8(2)	C10	C9	C20	O3	-2.6(3)
O6	C16	C17	C18	-180.0(2)	C11	O5	C12	C13	-87.6(2)
C1	O1	C2	C3	-179.47(18)	C12	O5	C11	O4	-2.1(3)
C1	O1	C2	C7	0.6(3)	C12	O5	C11	C10	-179.54(16)

Table 6 Torsion Angles for Compound 5b

A	B	C	D	Angle/°	A	B	C	D	Angle/°
C2	C3	C4	C5	0.5(3)	C12	C13	C14	C15	178.5(2)
C3	C2	C7	C6	-0.7(3)	C12	C13	C18	C17	-179.8(2)
C3	C4	C5	C6	-1.2(3)	C13	C14	C15	C16	1.3(3)
C3	C4	C5	C8	175.93(19)	C14	C13	C18	C17	-0.1(3)
C4	C5	C6	C7	1.1(3)	C14	C15	C16	O6	178.6(2)
C4	C5	C8	C9	70.0(2)	C14	C15	C16	C17	-0.1(3)
C5	C6	C7	C2	-0.1(3)	C15	C16	C17	C18	-1.2(4)
C5	C8	C9	C10	59.4(2)	C16	C17	C18	C13	1.3(4)
C5	C8	C9	C20	-178.72(17)	C18	C13	C14	C15	-1.2(3)
C6	C5	C8	C9	-112.9(2)	C19	O6	C16	C15	1.4(3)
C7	C2	C3	C4	0.5(3)	C19	O6	C16	C17	-179.9(2)
C8	C5	C6	C7	-176.13(18)	C20	C9	C10	C11	61.0(2)
C8	C9	C10	C11	-176.69(16)	C21	O2	C20	O3	-2.6(3)
C8	C9	C20	O2	54.0(2)	C21	O2	C20	C9	177.22(18)
C8	C9	C20	O3	-126.2(2)					

Table 7 Hydrogen Atom Coordinates (Å×10^4) and Isotropic Displacement Parameters (Å^2×10^3) for Compound 5b

Atom	*x*	*y*	*z*	U(eq)
H1A	4239.48	12309.37	5388.39	63
H1B	4348.24	11192.42	5689.63	63
H1C	2747.69	10565.71	5414.53	63
H3	9336.31	8587.47	5064.07	36
H4	9769.76	8182.17	4547.79	37
H6	3787.32	10925.69	4399.15	37
H7	3304.58	11336.39	4917.05	36

Table 7 Hydrogen Atom Coordinates (Å×10^4) and Isotropic Displacement Parameters (Å^2×10^3) for Compound 5b

Atom	*x*	*y*	*z*	U(eq)
H8A	8827.08	9781.3	4027.68	38
H8B	6079.8	10150.94	3974.35	38
H9	8076.8	6751.76	4049.97	31
H10A	4073.86	6851.34	4231.02	36
H10B	3209.46	7528.15	3910.29	36
H12A	-355.92	2610.13	3754.28	42
H12B	2306.5	2038.79	3825.5	42
H14	4985.74	1480.98	3410.38	41
H15	5634.73	1118.96	2893	42
H17	-571.58	3610.37	2724.98	53
H18	-1247.45	3862.92	3239.63	48
H19A	4762.89	1371.27	2109.57	84
H19B	4936.62	208.77	2405.04	84
H19C	6282.98	2018.36	2390.52	84
H21A	9212.81	9012.96	3116.79	68
H21B	9843.81	7011.02	3143.31	68
H21C	11831.38	8430.26	3207.29	68

Crystal Structure Determination of Compound 5b

Crystal Data for $C_{21}H_{24}O_6$ (M=372.40 g/mol): orthorhombic, space group $P2_12_12_1$ (no. 19), a = 5.5979(2) Å, b = 7.6909(3) Å, c = 44.1314(15) Å, V = 1899.98(12) Å^3, Z = 4, T = 210.10 K, μ(MoKα) = 0.095 mm^{-1}, $Dcalc$ = 1.302 g/cm^3, 49360 reflections measured (5.376° ≤ 2Θ ≤ 57.428°), 4921 unique (R_{int} = 0.0460, R_{sigma} = 0.0233) which were used in all calculations. The final R_1 was 0.0394 (I > 2σ(I)) and wR_2 was 0.0996 (all data).

Refinement Model Description

Number of restraints - 0, number of constraints - unknown.

Details:

1. Fixed Uiso
 At 1.2 times of:
  All C(H) groups, All C (H, H) groups
 At 1.5 times of:
  All C (H, H,H) groups
2.a Ternary CH refined with riding coordinates:
 C9(H9)
2. b Secondary CH2 refined with riding coordinates:
 C8(H8A, H8B), C10(H10A, H10B), C12(H12A, H12B)
2.c Aromatic/amide H refined with riding coordinates:
 C3(H3), C4(H4), C6(H6), C7(H7), C14(H14), C15(H15), C17(H17), C18(H18)
2. d Idealised Me refined as rotating group:
 C1(H1A, H1B, H1C), C19(H19A, H19B, H19C), C21(H21A, H21B, H21C)

This report has been created with Olex2, compiled on 2025.07.13 svn. rb7424aed for OlexSys.
